# Supplementary material for: Text mining of CHO bioprocess bibliome: Topic modeling and document classification
Source: PLoS One. 2023 Apr 6;18(4):e0274042. doi: 10.1371/journal.pone.0274042 (PMC10079098; doi:10.1371/journal.pone.0274042)
Supplement: S1 Fig — (PDF) [file pone.0274042.s001.pdf]

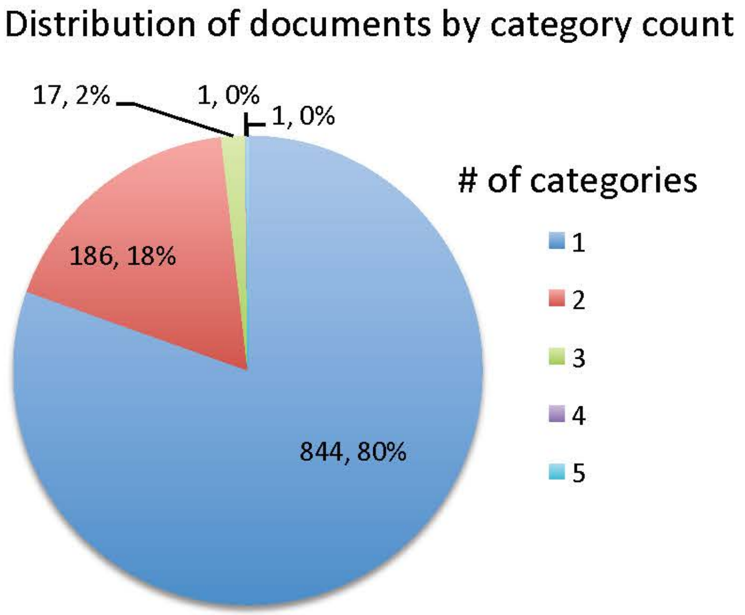

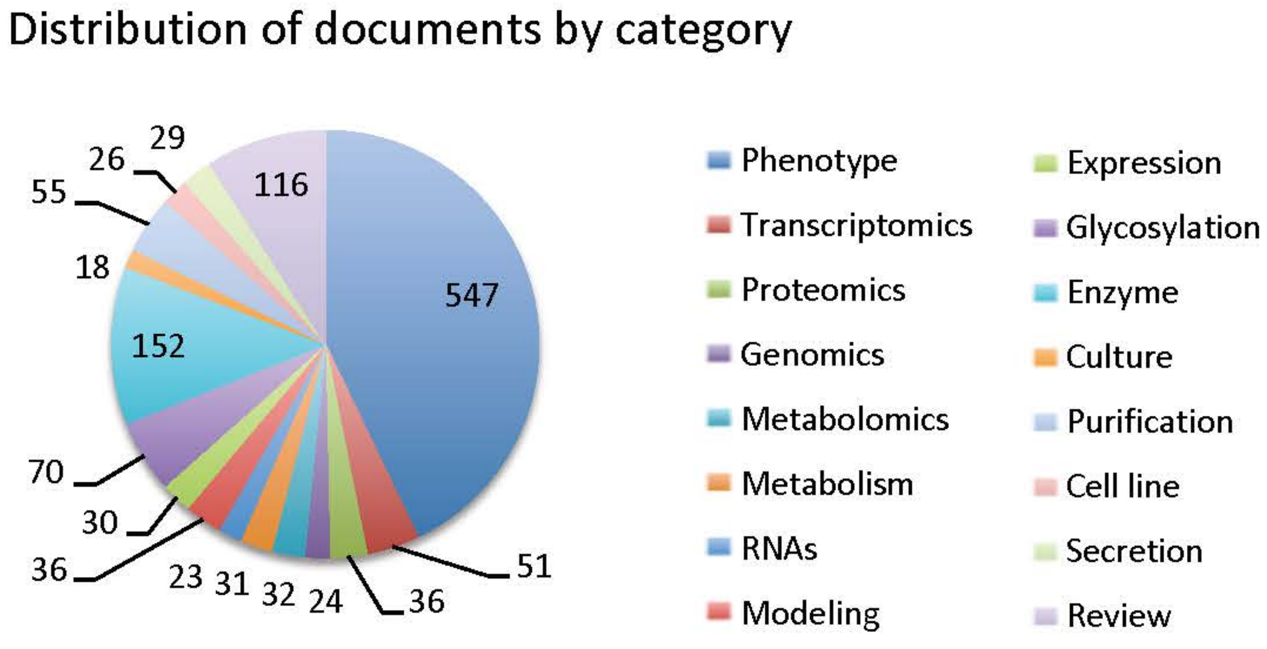

S1 Fig. Overview of CHO bibliome bioprocessing set with manual categories. Distribution of human labels for 16 categories, by category label count (top) and by document count (bottom), respectively.
