# Supplementary material for: Text mining of CHO bioprocess bibliome: Topic modeling and document classification
Source: PLoS One. 2023 Apr 6;18(4):e0274042. doi: 10.1371/journal.pone.0274042 (PMC10079098; doi:10.1371/journal.pone.0274042)
Supplement: S2 Fig — (PDF) [file pone.0274042.s002.pdf]

Index ||| PMID ||| (TopicID,score) ||| TopicID ||| Internal_TopicID ||| Document (Title & Abstract)

700 ||| 9043639 || (4, 0.49),(1, 0.37) ||| 4 ||| 5 ||| The effect of alpha-tocopherol and beta-tocopherol on proliferation, protein kinase C activity and gene expression in different cell lines. alpha-Tocopherol, but not beta-tocopherol, negatively regulates proliferation of A7r5 vascular smooth muscle cells at physiological concentration. The HeLa cell line was not affected whereas the Chinese hamster ovary cell line (CHO) was slightly inhibited by both alpha-tocopherol and beta-tocopherol. In A7r5 cells alpha-tocopherol inhibited protein kinase C activity, and this correlated with inhibition of proliferation. beta-Tocopherol did not inhibit either protein kinase C or proliferation. In HeLa cells no inhibition by alpha-tocopherol or beta-tocopherol of protein kinase C activity and cell proliferation was observed. In Chinese hamster ovary cells both tocopherols inhibited protein kinase C activity but not proliferation. Thus in the latter cells proliferation was not protein kinase C-dependent. In A7r5 cells alpha-tocopherol but not beta-tocopherol activated AP-1-mediated gene expression. In HeLa cells no change in gene expression was observed in agreement with the finding that also protein kinase C was not affected. In CHO cells gene-expression was activated by both alpha-tocopherol and beta-tocopherol. In this case also a positive correlation was found with similar inhibition of protein kinase C activity. In these cells, however, the changes at the level of protein kinase C activity and gene expression did not result in proliferation changes. The effect of alpha-tocopherol and beta-tocopherol on protein kinase C activity and gene expression suggest a cause-to-effect relationship. Inhibition of proliferation, however, correlates in the case of A7r5 and HeLa cells but not in the case of CHO suggesting a different proliferation pathway for these cells.

S2 Fig. Snip shot of Topic4Term.html in S3 File to show PMID 9043639 document with salient terms in color. It has probability of 0.49 for Topic-4 and 0.37 for Topic-1 according to the LDA model predictions. The color-coding of the words is based on their significance in the Topic-4. See details in Topic4Terms.html.
