# Supplementary material for: Text mining of CHO bioprocess bibliome: Topic modeling and document classification
Source: PLoS One. 2023 Apr 6;18(4):e0274042. doi: 10.1371/journal.pone.0274042 (PMC10079098; doi:10.1371/journal.pone.0274042)
Supplement: S3 Fig — (PDF) [file pone.0274042.s003.pdf]

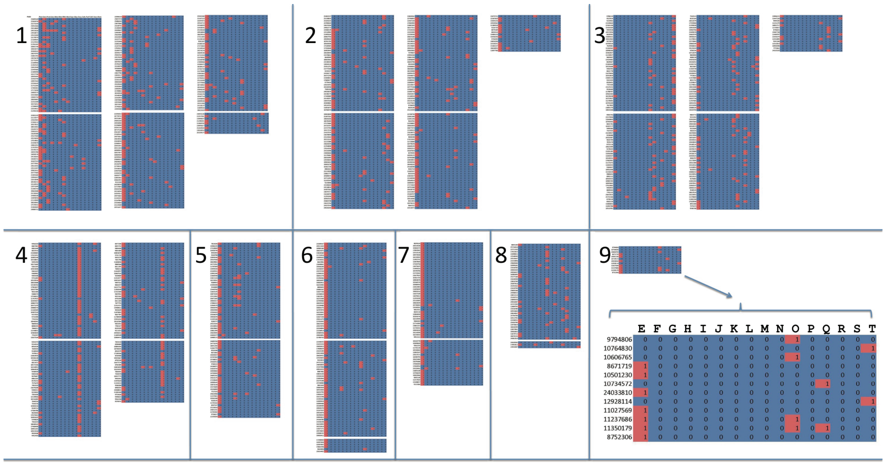

	E	F	G	H	I	J	K	L	M	N	O	P	Q	R	S	T
9794806	0	0	0	0	0	0	0	0	0	0	1	0	0	0	0	0
10764830	0	0	0	0	0	0	0	0	0	0	0	0	0	0	0	1
10606765	0	0	0	0	0	0	0	0	0	0	1	0	0	0	0	0
8671719	1	0	0	0	0	0	0	0	0	0	0	0	0	0	0	0
10501230	1	0	0	0	0	0	0	0	0	0	0	0	0	0	0	0
10734572	0	0	0	0	0	0	0	0	0	0	0	0	1	0	0	0
24033810	1	0	0	0	0	0	0	0	0	0	0	0	0	0	0	0
12928114	0	0	0	0	0	0	0	0	0	0	0	0	0	0	0	1
11027569	1	0	0	0	0	0	0	0	0	0	0	0	0	0	0	0
11237686	1	0	0	0	0	0	0	0	0	0	1	0	0	0	0	0
11350179	1	0	0	0	0	0	0	0	0	0	1	0	1	0	0	0
8752306	1	0	0	0	0	0	0	0	0	0	0	0	0	0	0	0

Figure S3. **Dsitribution of documents by LDA topic-1 to -9 and manual categories.** Heatmap for each topic shows the manual categories (columns) of the documents in the topic, each document (row with PMID shown on the left) with 1 (red) or 0 (blue) to indicate the manual assignment status for the manual category (column).
