# Supplementary material for: Text mining of CHO bioprocess bibliome: Topic modeling and document classification
Source: PLoS One. 2023 Apr 6;18(4):e0274042. doi: 10.1371/journal.pone.0274042 (PMC10079098; doi:10.1371/journal.pone.0274042)
Supplement: S1 Table — (PDF) [file pone.0274042.s004.pdf]

S1 Table. Human labels of categories for CHO Bioprocess set (cited from [1])

Category (label)	Document Dataset Size	Description
***Phenotype* and Production Characteristics**	547	As discussed in the main text, this category contains articles with relevant information on CHO cell metabolism, phenotype and productivity under different culture conditions. This category also includes studies that aimed to characterize CHO cell culture media, selection markers, effects of genetic modifications and methods for stable and transient expression of recombinant proteins.
Gene Expression and Transcriptomics	51	The earliest articles in this category (1995-2005) involve comparison of mRNA sequences across several CHO cell lines and hamster, characterization of transcription factors and sequencing of certain mRNA molecules. The latter period (2006-2015) include studies evaluating changes in gene expression in CHO cells different culture conditions and media as well as transcriptional profiling of relevant pathways (e.g. apoptosis) in CHO cells expressing recombinant proteins. Also, the latest articles include relevant information on CHO transcriptomes and development of high-throughput technologies (i.e. CHO-specific microarray chips) for transcriptomics.
Proteomics	36	This category includes studies that aimed to characterize the effects of gene expression on protein patterns as well as creating 2D maps of CHO cell proteomes via electrophoresis. Also, certain studies look at the influence of temperature, pH and other culture conditions on CHO cell proteome and levels of protein phosphorylation. Finally, those studies published as of 2008 are characterized by making ample use of high-throughput technologies for mapping the proteome of CHO cells under different culture conditions as well to try to understand the proteomic differences between high and low producing CHO cell lines.
***Genomics* and Epigenetics**	24	Some of the studies in this category that were published between 1995 and 2007 focused on characterizing the chromosomal localization of relevant genes in CHO cells as well as molecular mechanisms of DNA replication and effects of mutations on CHO cell phenotype. As of 2011, the focus of the studies in this category shifts towards sequencing the genomes of the Chinese hamster (*Cricetulus griseus*) and relevant CHO cell lines. Finally, this latter period in time includes the development of microarray and gene editing technologies for studying DNA methylation/epigenetic profiles and identifying targets for genetic engineering, respectively

Metabolomics* and *Fluxomics	32	The articles in this category focus on metabolomic profiling of intra- and extracellular metabolites in CHO cells under different culture conditions as well as on large-scale quantification of intracellular fluxes. Applications of metabolomics to identify targets for metabolic engineering are also included in this category
Metabolism* and *Metabolic Flux Analysis	31	This category contains articles that focus on quantifying the metabolic fluxes through specific pathways in CHO cells when varying the culture media, temperature or expression of relevant genes. This category also includes articles that aim to get a basic understanding of the dynamics and metabolic control of important pathways (e.g. lactate metabolism) in CHO cells during different growth phases and with different nutrient availabilities
***RNAs* and codon usage**	23	The studies in this category focus on the identification and application of RNA molecules in CHO cells during culture. For instance, profiling micro RNAs that might be involved in post-transcriptional control of recombinant protein productivity in CHO bioprocessing or using interference RNAs to regulate expression of target genes. Finally, this category also includes studies where codon usage was engineered in order to enhance CHO cell productivity
Modeling	36	This category includes in-silico biology techniques to simulate different aspects of CHO cell metabolism and/or growth dynamics during culture. Some of the approaches discussed in the articles in this category include kinetic modeling, steady state modeling, flux balance analysis, macroscopic bioreaction models, Markov Chain Monte Carlo method and multivariate analysis
Expression* and *Transfection Methods	30	This category includes articles where expression vectors are designed to silence, activate, overexpress or repress genes of interest in CHO cells. Also, techniques for transient and stable transfection of genes into CHO cells are discussed in several of these articles
Glycosylation	70	Articles where glycosylation profiles of recombinant proteins produced in CHO cells are studied are included in this category as well as articles where the effect of certain nutrients on glycosylation is assessed
***Enzyme* analysis**	152	The focus of the articles included in this category is 1) characterizing enzymes (e.g. structure, functions) in CHO cells, 2) studying the effects of overexpressing certain enzymatic genes on CHO cell productivity and growth and 3) analyzing the biochemistry of pathways in CHO cells with enzymatic mutations

Culture strategy and Bioreactor Design	18	Some of the culture strategies discussed in this category include artificial substrates for protein-free culture, microcarrier design for perfusion cultures and two-stage depth filter perfusion culture. Regarding bioreactor design approaches, this category includes solid-bed reactors for continuous flow, fluidized bed reactors and microfluidic devices
Purification and Separation Methods	55	The articles in this category focus on describing techniques to purify and separate recombinant and non-recombinant proteins in CHO cells. Several comparisons across various chromatography techniques are also included in this category
Cell line construction and characterization	26	As the name of this category implies, here we have included studies that describe novel CHO cell lines capable of producing several different recombinant proteins or with key genetic modifications that confer these cells interesting properties
Secretory Pathway and Product Secretion	29	The articles in this category look at the effects of culture conditions and/or genetic manipulations on the productivity and secretion capacity of CHO cells. Also, here we have included studies that look at bottlenecks of intracellular trafficking and folding of recombinant proteins. Finally, techniques for monitoring the unfolded protein response (UPR) are also discussed in some of these articles
Review article or other	116	Besides including review articles relevant to CHO cell bioprocessing and biology, this category includes several articles on topics that do not fit in any of the previous categories. For example, methods for expressing receptors in CHO cells, description of signaling pathways, techniques for characterizing recombinant proteins produced in CHO cells and strategies for studying/controlling apoptosis.

1. Golabgir A, Gutierrez JM, Hefzi H, Li S, Palsson BO, Herwig C, et al. Quantitative feature extraction from the Chinese hamster ovary bioprocess bibliome using a novel meta-analysis workflow. Biotechnology advances. 2016;34(5):621-33. doi: 10.1016/j.biotechadv.2016.02.011. PubMed PMID: 26948029.
