# Supplementary material for: Text mining of CHO bioprocess bibliome: Topic modeling and document classification
Source: PLoS One. 2023 Apr 6;18(4):e0274042. doi: 10.1371/journal.pone.0274042 (PMC10079098; doi:10.1371/journal.pone.0274042)
Supplement: S1 File — (PDF) [file pone.0274042.s005.pdf]

Supporting Information

Comparison of LDA Topics (subset with single topic, named “Collection-Single”) with manual categories

In an effort to directly compare topics from the LDA model and those research categories manually assigned in the CHO Bibliome [1], focus here is on the set of documents (n=336, named Collection-Single, see Figure S4A) with single prevalent topic assigned by the LDA model (NB: single prevalence is defined by an assignment of single topic with LDA probability Score > 0.5, meanwhile no other topics with probability score > 0.2). Among the 336 documents, 264 have a single category assigned manually, 66 with 2 categories, 5 with 3 categories, and 1 with 5 categories (supplemental material) [see S1 Table for the categories’ details; here for convenience, they are called Category E to T]. Overall, some manually assigned categories are represented more frequently in the Collection-Single when compared with the full Collection-BP (Figure S4A). For example, Gene Expression and Transcriptomics (Category F), RNAs and codon usage (Category K), Cell line construction and characterization (Category R) categories have >50% increase of representation. Meanwhile, categories of Metabolism and Metabolic Flux Analysis (Category J), Metabolomics and Fluxomics (Category I), and Phenotype and Production Characteristics (Category E) show a significant decrease of representation in the Collection-Single (50%, 32% and 22% respectively). The dominant category of Collection-BP (i.e., Phenotype and Production Characteristics (Category E), which is assigned to 547 documents in Collection-BP) continues to be the category with the highest number of documents in Collection-Single (Figure S4A).

The graph of weight by category (Figure S4B) shows that Topic #1 covers the majority of documents from 6 categories (F-K), Topic #2 for 3 categories (M,R,S), Topic #3 for 3 categories (N, T, Q), Topic #4 for category O. Distinctively, Category E (i.e., Phenotype and Production Characteristics) is the only one without single dominant topic (i.e., weight of > 50%), which implies that the category has diverse information that forks into a number of topics (e.g., it has significant presence in Topic #1, #2, #7).

From the top-30 most relevant terms in each topic (Table S2), several topics show distinctively coherent themes, with facilitation of weight normalization graphs (Figure S4 B, C).

Topic 1 includes top relevant terms such as cell line, protein, production and productivity; as well as miRNA, cellular, engineering, fed batch, titer, and development. Its comparison with the manual categories indicates its major coverage for: Phenotype and Production Characteristics (Category E), Gene Expression and Transcriptomics (Category F), Proteomics (Category G), RNAs and codon usage (Category K).

Topic 2 includes gene, expression, cell line, clone, protein, production; stable, promoter, sequence, vector, transfection, selection. They apparently correlate well with the manual categories: Expression and Transfection Methods (Category M), Cell line construction and characterization (Category R), Phenotype and Production Characteristics (Category E).

Topic 3 has coherent top terms such as glycosylation, glycoprotein, glycan, oligosaccharide, structure, purify, purification, core, complex, chain. It reflects concepts from manual categories: Glycosylation (Category N), Purification and Separation Methods (Category Q).

Topic 4 captures mutant, activity, synthesis, pathway, enzyme, membrane, cholesterol, inhibit, inhibition, inhibitor. It represents the manual category of Enzyme analysis (Category O).

Topic 7 has differentiating terms such as apoptosis, culture, medium, effect, concentration, bcl2, growth, nabu, cell death, viability, serum. They are understandable coherent concepts, and indeed mostly correlates with the manual category of Phenotype and Production Characteristics (Category E). Similarly does Topic 5 and 6, with significant overlap (Figure S4B).

Topic 8 features: model, process, mab, culture, method, rate, concentration, bioreactor, system, perfusion, control, yield, parameter, kinetic etc. Indeed, these words mirror well with the Modeling (Category L) and Culture strategy & Bioreactor Design (Category P) categories in the manual assignment (see Table S1).

Topic 9 is of little significance given its tiny percentage in the corpus (Figure S4B).

A

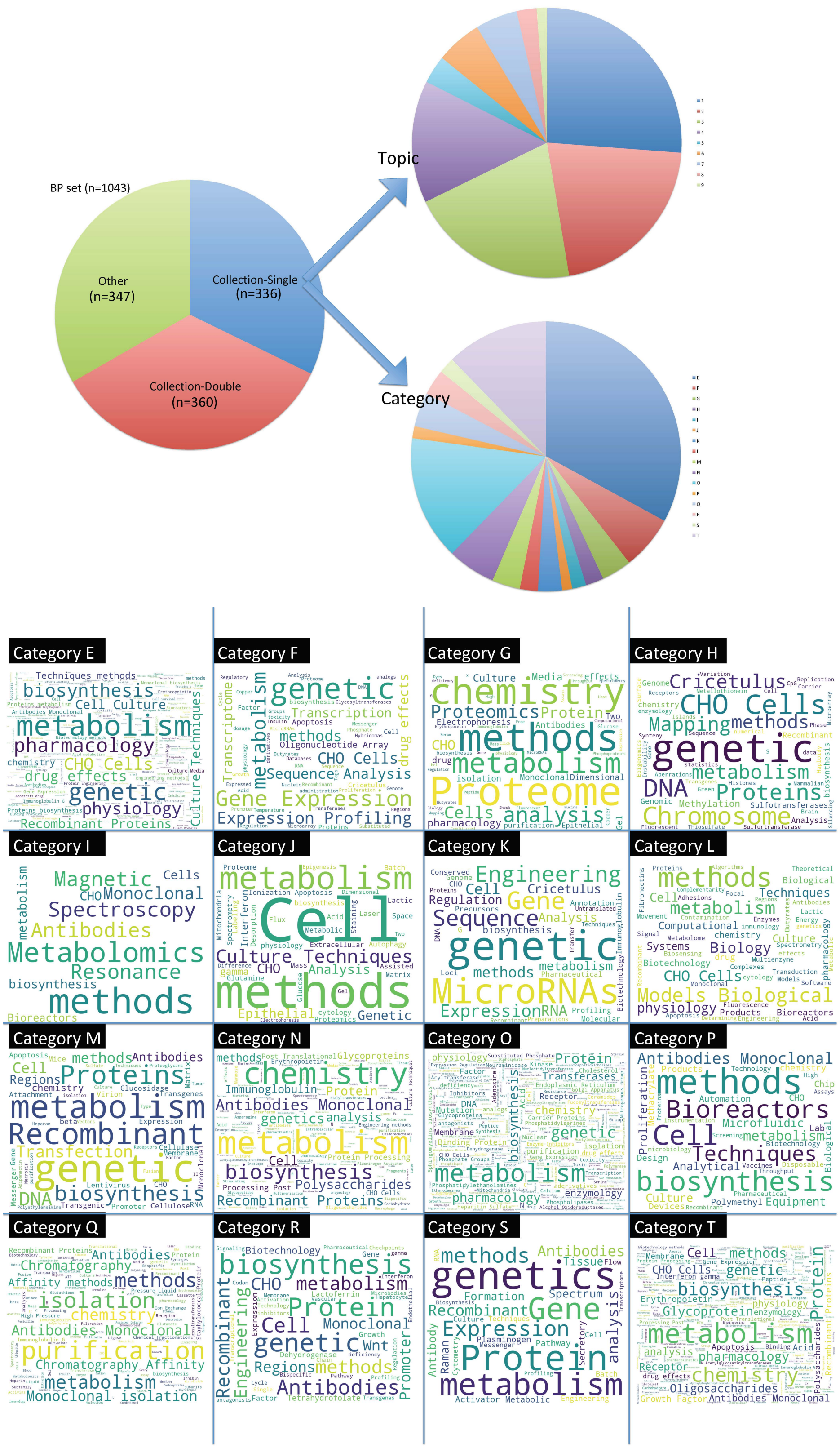
BP set (n=1043)
Other
(n=347)
Collection-Single
(n=336)
Collection-Double
(n=360)
Topic
Category
Category E
Category F
Category G
Category H
Category I
Category J
Category K
Category L
Category M
Category N
Category O
Category P
Category Q
Category R
Category S
Category T

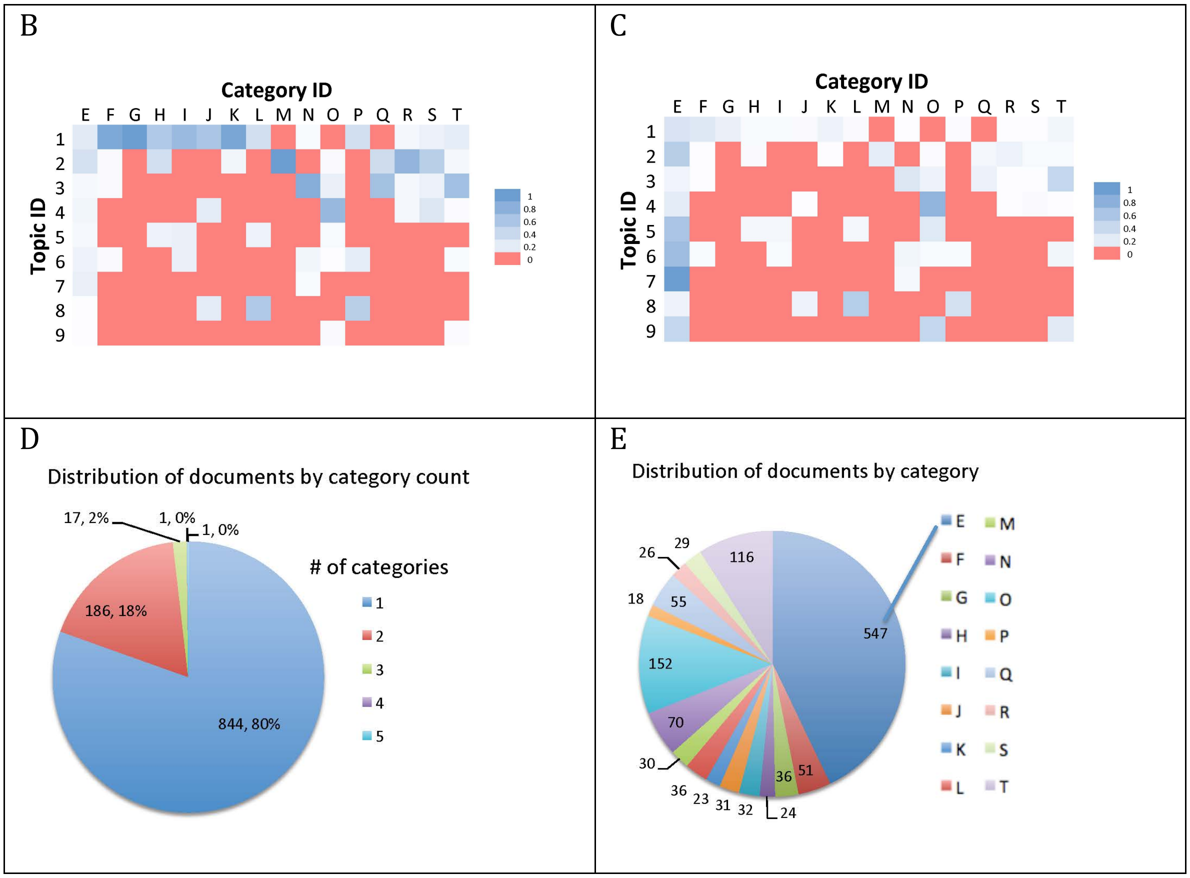

S4 Figure. Cross comparisons between LDA topics and manually assigned categories. (A) Distribution of 9 Topics of LDA model, and 16 Categories in CHO bibliome. The 16 categories in CHO Bibliome, with Wordcloud representations of MeSH term major topics (descriptors and qualifiers) for abstracts in a given category. MeSH contains several different types of terms. Descriptors (main headings): characterize the subject matter or content. Qualifiers: are used with descriptors and afford a means of grouping together those documents concerned with a particular aspect of a subject. (B) Normalized by Category count (column). (C) Normalized by Topic count (row).
