# Supplementary material for: Text mining of CHO bioprocess bibliome: Topic modeling and document classification
Source: PLoS One. 2023 Apr 6;18(4):e0274042. doi: 10.1371/journal.pone.0274042 (PMC10079098; doi:10.1371/journal.pone.0274042)
Supplement: S3 File — (ZIP) [file pone.0274042.s007.zip › S3_file/Topic2Terms.html]

Here is a list of 1049 bioprocessing BP documents (both title and abstract),
with the Top-30 most salient terms for a select Topic colored
according to the relevance order of the terms :
1-5 (most relevant) , 6-10, 11-15,
16-20,
21-25,
26-30 (least relevant among the Top-30).

Topic1

Topic2

Topic3

Topic4

Topic5

Topic6

Topic7

Topic8

Topic9

Topic NA

# Index ||| PMID ||| (TopicID,score) ||| TopicID ||| Internal\_TopicID ||| Document (Title & Abstract)

# ----------------------------- TOPIC1 -----------------------------

1 ||| 25689160 ||| (1, 0.99) ||| 1 ||| 8 ||| Annotation of additional evolutionary conserved microRNAs in CHO cells from updated genomic data. MicroRNAs are small non-coding RNAs that play a critical role in post-transcriptional control of gene expression. Recent publications of genomic sequencing data from the Chinese Hamster (CGR) and Chinese hamster ovary (CHO) cells provide new tools for the discovery of novel miRNAs in this important production system. Version 20 of the miRNA registry miRBase contains 307 mature miRNAs and 200 precursor sequences for CGR/CHO. We searched for evolutionary conserved miRNAs from miRBase v20 in recently published genomic data, derived from Chinese hamster and CHO cells, to further extend the list of known miRNAs. With our approach we could identify several hundred miRNA sequences in the genome. For several of these, the expression in CHO cells could be verified from multiple next-generation sequencing experiments. In addition, several hundred unexpressed miRNAs are awaiting further confirmation by testing for their transcription in different Chinese hamster tissues.

2 ||| 25583076 ||| (1, 0.99) ||| 1 ||| 8 ||| Metabolomics analysis of soy hydrolysates for the identification of productivity markers of mammalian cells for manufacturing therapeutic proteins. Soy hydrolysates are widely used as a nutrient supplement in mammalian cell culture for the production of recombinant proteins. The batch-to-batch variability of a soy hydrolysate often leads to productivity differences. This report describes our metabolomics platform, which includes a battery of LC-MS/MS modes of operation, and advanced data analysis software for automated data processing. The platform was successfully used for screening productivity markers in soy hydrolysates during the production of two therapeutic antibodies in two Chinese hamster ovary cell lines. A total of 123 soy hydrolysate batches were analyzed, from which 62 batches were used in the production runs of cell line #1 and 12 batches were used in the production runs of cell line #2. For cell line #1, out of 19 amino acids, 106 other metabolites and 4,131 peptides identified in the soy hydrolysate batches being used, several nucleosides and short hydrophobic peptides showed negative correlation with antibody titer, while ornithine, citrulline and several amino acids and organic acids correlated positively with titer. For cell line #2, only ornithine and citrulline showed strong positive correlation. When ornithine was spiked into the culture media, both cell lines demonstrated accelerated cell growth, indicating ornithine as a root cause of the performance difference. It is proposed that better soy hydrolysate performance resulted from better bacterial fermentation during the hydrolysate production. A few selected markers were used to predict the performance of other soy hydrolysate batches for cell line #1. The predicted titers agreed with the experimental values with good accuracy.

3 ||| 25271019 ||| (1, 0.99) ||| 1 ||| 8 ||| Hypoxia influences protein transport and epigenetic repression of CHO cell cultures in shake flasks. Shake flasks and bench-top bioreactors are widely used for cell culture process development, however, culture performances significantly differ between them. In order to apply the results received from small-scale cultures to production scale, it is important to understand the mechanisms underlying the differences between various culture systems. This study analyzes the expression patterns of Chinese hamster ovary (CHO) cells producing IgG-fusion protein B0 cultured in shake flasks and 5-L bench-top bioreactors by CHO-specific DNA microarrays. The data show that hypoxia was present in shake flask cultures but not in controlled, bench-top bioreactors. Hypoxic conditions appeared to be associated with epigenetic repression resulting in decreased cell culture performance and protein productivity, which is also present during large-scale bioreactor operations due to oxygen gradients. High protein productivity was associated with increased cellular machinery for protein transport and secretion in conjunction with decreased epigenetic repression in bench-top bioreactor cultivation. Metal ions could improve cell growth and protein production under hypoxia and this condition could be mimicked in small-scale bioreactors to facilitate cell culture process scale-up.

4 ||| 25052466 ||| (1, 0.99) ||| 1 ||| 8 ||| Identification of microRNAs specific for high producer CHO cell lines using steady-state cultivation. MicroRNAs are short non-coding RNAs that play an important role in the regulation of gene expression. Hence, microRNAs are considered as potential targets for engineering of Chinese hamster ovary (CHO) cells to improve recombinant protein production. Here, we analyzed and compared the microRNA expression patterns of high, low, and non-producing recombinant CHO cell lines expressing two structurally different model proteins in order to identify microRNAs that are involved in heterologous protein synthesis and secretion and thus might be promising targets for cell engineering to increase productivity. To generate reproducible and comparable data, the cells were cultivated in a bioreactor under steady-state conditions. Global microRNA expression analysis showed that mature microRNAs were predominantly upregulated in the producing cell lines compared to the non-producer. Several microRNAs were significantly differentially expressed between high and low producers, but none of them commonly for both model proteins. The identification of target messenger RNAs (mRNAs) is essential to understand the biological function of microRNAs. Therefore, we negatively correlated microRNA and global mRNA expression data and combined them with computationally predicted and experimentally validated targets. However, statistical analysis of the identified microRNA-mRNA interactions indicated a considerable false positive rate. Our results and the comparison to published data suggest that the reaction of CHO cells to the heterologous protein expression is strongly product- and/or clone-specific. In addition, this study highlights the urgent need for reliable CHO-specific microRNA target prediction tools and experimentally validated target databases in order to facilitate functional analysis of high-throughput microRNA expression data in CHO cells.

5 ||| 25014889 ||| (1, 0.99) ||| 1 ||| 8 ||| Advancing biopharmaceutical process science through transcriptome analysis. Global survey of transcriptome dynamics can provide molecular insights into cell physiology. In the past few years, DNA microarray for transcriptome analysis has been augmented by high-throughput sequencing methods; extending the reach of transcriptome analysis to the rodent species of biotechnological importance, for which the development of genomic tools has been lagging. The rapid accumulation of sequencing data for these species highlighted the need for more evidence-based annotation. Recent findings in the epigenetic regulation in human and mouse will inspire similar research in CHO and BHK cells. Transcriptome studies in these recombinant cells will likely lay the foundation for a systems-based genome engineering which can be used to develop superior producing cell lines. Herein, we summarized the recent findings and advances in transcriptome studies of cell culture bioprocesses. The potential impact of transcriptomics on biopharmaceutical process technology is also discussed.

6 ||| 24915626 ||| (1, 0.99) ||| 1 ||| 8 ||| Application of multi-omics techniques for bioprocess design and optimization in chinese hamster ovary cells. Significant improvements in the productivity and quality of therapeutic proteins produced in Chinese hamster ovary (CHO) cells have been reported since their establishment as host cells for biopharmaceutical production. Initial advances in the field focused on engineering strategies to manipulate genes associated with proliferation, apoptosis, and various metabolic pathways. Process engineering efforts to optimize culture media, batch-feeding strategies and culture conditions, including temperature and osmolarity, were also reported. More recently, focus has shifted toward enhancing process consistency and product quality using systems biology quality by design-based approaches during process development. Integration of different data generated using omics technologies, such as genomics, transcriptomics, proteomics and metabolomics, has facilitated a greater understanding of CHO cell biology. These techniques have enabled the provision of global information on dynamic changes in cellular components associated with different phenotypes. Using systems biology to understand these important host cells at the cellular level will undoubtedly result in further progression in the development and expression of biopharmaceutical products in CHO cells.

7 ||| 23916872 ||| (1, 0.99) ||| 1 ||| 8 ||| CHO microRNA engineering is growing up: recent successes and future challenges. microRNAs with their ability to regulate complex pathways that control cellular behavior and phenotype have been proposed as potential targets for cell engineering in the context of optimization of biopharmaceutical production cell lines, specifically of Chinese Hamster Ovary cells. However, until recently, research was limited by a lack of genomic sequence information on this industrially important cell line. With the publication of the genomic sequence and other relevant data sets for CHO cells since 2011, the doors have been opened for an improved understanding of CHO cell physiology and for the development of the necessary tools for novel engineering strategies. In the present review we discuss both knowledge on the regulatory mechanisms of microRNAs obtained from other biological models and proof of concepts already performed on CHO cells, thus providing an outlook of potential applications of microRNA engineering in production cell lines.

8 ||| 23523260 ||| (1, 0.99) ||| 1 ||| 8 ||| The emerging CHO systems biology era: harnessing the 'omics revolution for biotechnology. Chinese hamster ovary (CHO) cells are the primary factories for biopharmaceuticals because of their capacity to correctly fold and post-translationally modify recombinant proteins compatible with humans. New opportunities are arising to enhance these cell factories, especially since the CHO-K1 cell line was recently sequenced. Now, the CHO systems biology era is underway. Critical 'omics data sets, including proteomics, transcriptomics, metabolomics, fluxomics, and glycomics, are emerging, allowing the elucidation of the molecular basis of CHO cell physiology. The incorporation of these data sets into mathematical models that describe CHO phenotypes will provide crucial biotechnology insights. As 'omics technologies and computational systems biology mature, genome-scale approaches will lead to major innovations in cell line development and metabolic engineering, thereby improving protein production and bioprocessing.

9 ||| 23170974 ||| (1, 0.99) ||| 1 ||| 8 ||| Integrated miRNA, mRNA and protein expression analysis reveals the role of post-transcriptional regulation in controlling CHO cell growth rate. BACKGROUND: To study the role of microRNA (miRNA) in the regulation of Chinese hamster ovary (CHO) cell growth, qPCR, microarray and quantitative LC-MS/MS analysis were utilised for simultaneous expression profiling of miRNA, mRNA and protein. The sample set under investigation consisted of clones with variable cellular growth rates derived from the same population. In addition to providing a systems level perspective on cell growth, the integration of multiple profiling datasets can facilitate the identification of non-seed miRNA targets, complement computational prediction tools and reduce false positive and false negative rates. RESULTS: 51 miRNAs were associated with increased growth rate (35 miRNAs upregulated and 16 miRNAs downregulated). Gene ontology (GO) analysis of genes (n=432) and proteins (n=285) found to be differentially expressed (DE) identified biological processes driving proliferation including mRNA processing and translation. To investigate the influence of miRNA on these processes we combined the proteomic and transcriptomic data into two groups. The first set contained candidates where evidence of translational repression was observed (n=158). The second group was a mixture of proteins and mRNAs where evidence of translational repression was less clear (n=515). The TargetScan algorithm was utilised to predict potential targets within these two groups for anti-correlated DE miRNAs. CONCLUSIONS: The evidence presented in this study indicates that biological processes such as mRNA processing and protein synthesis are correlated with growth rate in CHO cells. Through the integration of expression data from multiple levels of the biological system a number of proteins central to these processes including several hnRNPs and components of the ribosome were found to be post-transcriptionally regulated. We utilised the expression data in conjunction with in-silico tools to identify potential miRNA-mediated regulation of mRNA/proteins involved in CHO cell growth rate. These data have allowed us to prioritise candidates for cell engineering and/or biomarkers relevant to industrial cell culture. We also expect the knowledge gained from this study to be applicable to other fields investigating the role of miRNAs in mammalian cell growth.

10 ||| 22189905 ||| (1, 0.99) ||| 1 ||| 8 ||| Profiling conserved microRNA expression in recombinant CHO cell lines using Illumina sequencing. MicroRNAs (miRNAs) are small, non-coding RNAs that regulate multiple aspects of cell physiology. The differential expression of conserved miRNAs in two Chinese hamster ovary (CHO) cell lines producing recombinant proteins was examined relative to the CHO-K1 cell line. A total of 190 conserved CHO miRNAs were identified through homology with known human and rodent miRNAs. More than 80% of these miRNAs showed differential expression in recombinant CHO cell lines. The small RNA sequencing data were analyzed in context of the CHO-K1 genome to examine miRNA organization and develop sequence-specific miRNA resources for CHO cells. The identification and characterization of CHO miRNAs will facilitate the use of miRNA tools in cell line engineering efforts to improve product yield and quality.

11 ||| 22147654 ||| (1, 0.99) ||| 1 ||| 8 ||| Microarray expression profiling identifies genes regulating sustained cell specific productivity (S-Qp) in CHO K1 production cell lines. Fed batch culture processes are often characterized by decreasing cell culture performance as the process continues, presumably through the depletion of vital nutrients and the accumulation of toxic byproducts. We have similarly observed that cellular productivity (Qp) often declines during the course of a fed batch process; however, it is not clear why some cell lines elicit this behavior, while others do not. We here present a transcriptomic profiling analysis of a phenotype of sustained Qp (S-Qp) in production Chinese hamster ovary (CHO) culture, in which a marked drop in Qp levels ("non-sustained" (NS) phenotype) in two cell lines irrespective of viability levels was compared to two cell lines that consistently displayed high Qp throughout the culture ("sustained" (S) phenotype). Statistical analysis of the microarray data resulted in the identification of 22 gene transcripts whose expression patterns were either significantly negatively or positively correlated with long-term maintenance of Qp over the culture lifespan. qPCR analysis of four of these genes on one of each (NS2, S2) of the cell lines examined by microarray analysis confirmed that two genes (CRYAB and MGST1) both replicated the microarray results and were differentially regulated between the NS and S phenotypes.

12 ||| 21781345 ||| (1, 0.99) ||| 1 ||| 8 ||| Sustained productivity in recombinant Chinese hamster ovary (CHO) cell lines: proteome analysis of the molecular basis for a process-related phenotype. BACKGROUND: The ability of mammalian cell lines to sustain cell specific productivity (Qp) over the full duration of bioprocess culture is a highly desirable phenotype, but the molecular basis for sustainable productivity has not been previously investigated in detail. In order to identify proteins that may be associated with a sustained productivity phenotype, we have conducted a proteomic profiling analysis of two matched pairs of monoclonal antibody-producing Chinese hamster ovary (CHO) cell lines that differ in their ability to sustain productivity over a 10 day fed-batch culture. RESULTS: Proteomic profiling of inherent differences between the two sets of comparators using 2D-DIGE (Difference Gel Electrophoresis) and LC-MS/MS resulted in the identification of 89 distinct differentially expressed proteins. Overlap comparisons between the two sets of cell line pairs identified 12 proteins (AKRIB8, ANXA1, ANXA4, EIF3I, G6PD, HSPA8, HSP90B1, HSPD1, NUDC, PGAM1, RUVBL1 and CNN3) that were differentially expressed in the same direction. CONCLUSION: These proteins may have an important role in sustaining high productivity of recombinant protein over the duration of a fed-batch bioprocess culture. It is possible that many of these proteins could be useful for future approaches to successfully manipulate or engineer CHO cells in order to sustain productivity of recombinant protein.

13 ||| 20091739 ||| (1, 0.99) ||| 1 ||| 8 ||| Microarray and proteomics expression profiling identifies several candidates, including the valosin-containing protein (VCP), involved in regulating high cellular growth rate in production CHO cell lines. A high rate of cell growth (micro) leading to rapid accumulation of viable biomass is a desirable phenotype during scale up operations and the early stages of production cultures. In order to identify genes and proteins that contribute to higher growth rates in Chinese hamster ovary (CHO) cells, a combined approach using microarray and proteomic expression profiling analysis was carried out on two matched pairs of CHO production cell lines that displayed either fast or slow growth rates. Statistical analysis of the microarray and proteomic data separately resulted in the identification of 118 gene transcripts and 58 proteins that were differentially expressed between the fast- and slow-growing cells. Overlap comparison of both datasets identified a priority list of 21 candidates associated with a high growth rate phenotype in CHO. Functional analysis (by siRNA) of five of these candidates identified the valosin-containing protein (VCP) as having a substantial impact on CHO cell growth and viability. Knockdown of HSPB1 and ENO1 also had an effect on cell growth (negative and positive, respectively). Further functional validation in CHO using both gene knockdown (siRNA) and overexpression (cDNA) confirmed that altered VCP expression impacted CHO cell proliferation, indicating that VCP and other genes and proteins identified here may play an important role in the regulation of CHO cell growth during log phase culture and are potential candidates for CHO cell line engineering strategies.

14 ||| 19003075 ||| (1, 0.99) ||| 1 ||| 8 ||| Toward genomic cell culture engineering. Genomic and proteomic based global gene expression profiling has altered the landscape of biological research in the past few years. Its potential impact on cell culture bioprocessing has only begun to emanate, partly due to the lack of genomic sequence information for the most widely used industrial cells, Chinese hamster ovary (CHO) cells. Transcriptome and proteome profiling work for species lacking extensive genomic resources must rely on information for other related species or on data obtained from expressed sequence tag (EST) sequencing projects, for which burgeoning efforts have only recently begun. This article discusses the aspects of EST sequencing in those industrially important, genomic resources-poor cell lines, articulates some of the unique features in employing microarray in the study of cultured cells, and highlights the infrastructural needs in establishing a platform for genomics based cell culture research. Recent experience has revealed that generally, most changes in culture conditions only elicit a moderate level of alteration in gene expression. Nevertheless, by broadening the conventional scope of microarray analysis to consider estimated levels of transcript abundance, much physiological insight can be gained. Examples of the application of microarray in cell culture are discussed, and the utility of pattern identification and process diagnosis are highlighted. As genomic resources continue to expand, the power of genomic tools in cell culture processing research will be amply evident. The key to harnessing the immense benefit of these genomic resources resides in the development of physiological understanding from their application.

15 ||| 20306541 ||| (1, 0.94) ||| 1 ||| 8 ||| Transcriptomic responses to sodium chloride-induced osmotic stress: a study of industrial fed-batch CHO cell cultures. The rapidly expanding market for monoclonal antibody and Fc-fusion-protein therapeutics has increased interest in improving the productivity of mammalian cell lines, both to alleviate capacity limitations and control the cost of goods. In this study, we evaluated the responses of an industrial CHO cell line producing an Fc-fusion-protein to hyperosmotic stress, a well-known productivity enhancer, and compared them with our previous studies of murine hybridomas (Shen and Sharfstein, Biotechnol Bioeng. 2006;93:132-145). In batch culture studies, cells showed substantially increased specific productivity in response to increased osmolarity as well as significant metabolic changes. However, the final titer showed no substantial increase due to the decrease in viable cell density. In fed batch cultures, hyperosmolarity slightly repressed the cellular growth rate, but no significant change in productivity or final titer was detected. To understand the transcriptional responses to increased osmolarity and relate changes in gene expression to increased productivity and repressed growth, proprietary CHO microarrays were used to monitor the transcription profile changes in response to osmotic stress. A set of osmotically regulated genes was generated and classified by extracting their annotations and functionalities from online databases. The gene list was compared with results previously obtained from similar studies of murine-hybridoma cells. The overall transcriptomic responses of the two cell lines were rather different, although many functional groups were commonly perturbed between them. Building on this study, we anticipate that further analysis will establish connections between productivity and the expression of specific gene(s), thus allowing rational engineering of mammalian cells for higher recombinant-protein productivity.

16 ||| 24874795 ||| (1, 0.93) ||| 1 ||| 8 ||| Toward product attribute control: developments from genome sequencing. Chinese hamster ovary (CHO) cells are important hosts for the production of therapeutic proteins. Recent genome sequencing studies provide an initial baseline of information useful for understanding cell line performance in terms of product quality attributes. However, the lack of a well-established reference genome together with concerns about genome stability have not yet permitted the community to define the detailed relationship between the genome and cell line performance. Emerging efforts to define a new reference genome, together with new data on genome stability, herald an era where cell line's with defined genomes can be combined with defined process parameters to yield product quality attribute control.

17 ||| 21801763 ||| (1, 0.93) ||| 1 ||| 8 ||| Large scale microarray profiling and coexpression network analysis of CHO cells identifies transcriptional modules associated with growth and productivity. Weighted gene coexpression network analysis (WGCNA) was utilised to explore Chinese hamster ovary (CHO) cell transcriptome patterns associated with bioprocess relevant phenotypes. The dataset set used in this study consisted of 295 microarrays from 121 individual CHO cultures producing a range of biologics including monoclonal antibodies, fusion proteins and therapeutic factors; non-producing cell lines were also included. Samples were taken from a wide range of process scales and formats that varied in terms of seeding density, temperature, medium, feed medium, culture duration and product type. Cells were sampled for gene expression analysis at various stages of the culture and bioprocess-relevant characteristics including cell density, growth rate, viability, lactate, ammonium and cell specific productivity (Qp) were determined. WGCNA identified six distinct clusters of co-expressed genes, five of which were found to have associations with bioprocess variables. Two coexpression clusters were found to be associated with culture growth rate (1 positive and 1 negative). In addition, associations between a further three coexpression modules and Qp were observed (1 positive and 2 negative). Gene set enrichment analysis (GSEA) identified a number of significant biological processes within coexpressed gene clusters including cell cycle, protein secretion and vesicle transport. In summary, the approach presented in this study provides a novel perspective on the CHO cell transcriptome.

18 ||| 23876478 ||| (1, 0.91) ||| 1 ||| 8 ||| Translatome analysis of CHO cells to identify key growth genes. We report the first investigation of translational efficiency on a global scale, also known as translatome, of a Chinese hamster ovary (CHO) DG44 cell line producing monoclonal antibodies (mAb). The translatome data was generated via combined use of high resolution and streamlined polysome profiling technology and proprietary Nimblegen microarrays probing for more than 13K annotated CHO-specific genes. The distribution of ribosome loading during the exponential growth phase revealed the translational activity corresponding to the maximal growth rate, thus allowing us to identify stably and highly translated genes encoding heterogeneous nuclear ribonucleoproteins (Hnrnpc and Hnrnpa2b1), protein regulator of cytokinesis 1 (Prc1), glucose-6-phosphate dehydrogenase (G6pdh), UTP6 small subunit processome (Utp6) and RuvB-like protein 1 (Ruvbl1) as potential key players for cellular growth. Moreover, correlation analysis between transcriptome and translatome data sets showed that transcript level and translation efficiency were uncoupled for 95% of investigated genes, suggesting the implication of translational control mechanisms such as the mTOR pathway. Thus, the current translatome analysis platform offers new insights into gene expression in CHO cell cultures by bridging the gap between transcriptome and proteome data, which will enable researchers of the bioprocessing field to prioritize in high-potential candidate genes and to devise optimal strategies for cell engineering toward improving culture performance.

19 ||| 23775295 ||| (1, 0.91) ||| 1 ||| 8 ||| Automated disposable small scale reactor for high throughput bioprocess development: a proof of concept study. The acceleration of bioprocess development for biologics and vaccines can be enabled by automated high throughput technologies. This will alleviate the significant resource burden from the multi-factorial statistical experimentation required for controlling product quality attributes of complex biologics. Recent technology advances have improved clone evaluation and screening, but have struggled to combine the scale down criteria required for both high cell density cell culture and microbial processes, with sufficient automation and disposable technologies to accelerate process development. This article describes the proof of concept evaluations of an automated disposable small scale reactor for high throughput upstream process development. Characterization studies established the small scale stirred tank disposable 250 mL reactor as similar to those of lab and pilot scale. The reactor generated equivalent process performance for industrial biologics processes for therapeutic protein and monoclonal antibody production using CHO cell culture, Pichia pastoris and E. coli. This included similar growth, cell viability, product titer, and product quality. The technology was shown to be robust across multiple runs and met the requirements for the ability to run high cell density processes (>400 g/L wet cell weight) with exponential feeds and sophisticated event triggered processes. Combining this reactor into an automated array of reactors will ultimately be part of a high throughput process development strategy. This will combine upstream, small scale purification with rapid analytics that will dramatically shorten timelines and costs of developing biological processes.

20 ||| 22189966 ||| (1, 0.91) ||| 1 ||| 8 ||| CGCDB: a web-based resource for the investigation of gene coexpression in CHO cell culture. UNLABELLED: Coexpression analysis is a powerful, widely used methodology for the investigation of underlying patterns in gene expression data. This "guilt-by-association" approach aims to find groups of genes with closely correlated expression profiles. Observation of consistent correlations across phenotypically diverse samples indicates that these genes have a shared function. We have recently described the application of weighted gene coexpression network analysis (WGCNA) to a 295 sample production CHO cell line microarray dataset and elucidated groups of genes related to growth rate and cell-specific productivity (Qp). In this study, we present the CHO gene coexpression database (CGCDB), a web-based system, designed specifically for researchers in the CHO community to provide user-friendly access to these gene-gene coexpression patterns. In addition to correlation between genes, the direct correlations between probesets and either growth rate or Qp are provided. Results are presented to the user via an interactive network diagram and in a downloadable tabular format. It is hoped that this resource will allow researchers to prioritize cell line engineering and/or biomarker candidates to enhance CHO-based cell culture for the production of biotherapeutics. AVAILABILITY: www.cgcdb.org.

21 ||| 21392545 ||| (1, 0.91) ||| 1 ||| 8 ||| Next-generation sequencing of the Chinese hamster ovary microRNA transcriptome: Identification, annotation and profiling of microRNAs as targets for cellular engineering. Chinese hamster ovary (CHO) cells are the predominant cell factory for the production of recombinant therapeutic proteins. Nevertheless, the lack in publicly available sequence information is severely limiting advances in CHO cell biology, including the exploration of microRNAs (miRNA) as tools for CHO cell characterization and engineering. In an effort to identify and annotate both conserved and novel CHO miRNAs in the absence of a Chinese hamster genome, we deep-sequenced small RNA fractions of 6 biotechnologically relevant cell lines and mapped the resulting reads to an artificial reference sequence consisting of all known miRNA hairpins. Read alignment patterns and read count ratios of 5' and 3' mature miRNAs were obtained and used for an independent classification into miR/miR\* and 5p/3p miRNA pairs and discrimination of miRNAs from other non-coding RNAs, resulting in the annotation of 387 mature CHO miRNAs. The quantitative content of next-generation sequencing data was analyzed and confirmed using qPCR, to find that miRNAs are markers of cell status. Finally, cDNA sequencing of 26 validated targets of miR-17-92 suggests conserved functions for miRNAs in CHO cells, which together with the now publicly available sequence information sets the stage for developing novel RNAi tools for CHO cell engineering.

22 ||| 23651948 ||| (1, 0.90) ||| 1 ||| 8 ||| Transcriptomic analysis of clonal growth rate variation during CHO cell line development. The selection of clones displaying a high rate of cell growth is an essential component of Chinese hamster ovary (CHO) cell line development. In recent years various "omics" technologies have been utilised to understand the mechanisms underlying bioprocess phenotypes. In this study, gene expression analysis using a CHO-specific microarray was conducted for a panel of CHO-K1 MAb-secreting cell lines spanning a range of growth rates that were derived from a single cell line development project. In-silico functional analysis of the resulting transcriptomic data revealed the overrepresentation of biological processes such as cell cycle and translation within those genes upregulated during fast growth, while genes associated with cellular homeostasis were downregulated. Using differential expression and correlation analysis we identified a high priority group of 416 transcripts (190 upregulated; 226 downregulated) associated with growth rate. Expression changes of eight of these genes were independently confirmed by qPCR. Finally, we demonstrate the enrichment of predicted mRNA targets of miR17-92, a microRNA (miRNA) cluster known to be upregulated during rapid proliferation, within downregulated transcripts.

23 ||| 22445466 ||| (1, 0.90) ||| 1 ||| 8 ||| Impact of miR-7 over-expression on the proteome of Chinese hamster ovary cells. MicroRNAs play critical roles in the regulation of biological processes such as growth, apoptosis, productivity and secretion thus representing a potential route toward enhancing desirable characteristics of mammalian cells for biopharmaceutical production. We have previously found that miR-7 over-expression significantly inhibits the growth of CHO-SEAP cells without impacting cellular viability, with an associated increase in normalised productivity. Understanding the biological basis of this effect might open the way to new strategies for bioprocess-relevant growth regulation. In this study we have carried out a quantitative label-free LC-MS profiling study of proteins exhibiting altered levels following over-expression of miR-7 to gain insights into potential mechanisms involved in the observed phenotype. From the analysis we found 93 proteins showing decreased levels and 74 proteins with increased levels following over-expression of miR-7. Pathway analysis suggests that proteins involved in protein translation (e.g. ribosomal proteins), RNA and DNA processing (including histones) are enriched in the list of proteins showing decreased expression. Proteins involved in protein folding and secretion were found to be up-regulated following miR-7 over-expression. In silico bioinformatic analysis using miRWalk, which combined the output from 6 selected miRNA target prediction algorithms, was used to evaluate if any of the down-regulated proteins were potential direct targets of miR-7. Two genes, stathmin and catalase, which both have known roles in the regulation of cellular growth, were found to overlap a number of the predictive target database searches in both mouse and rat, and are likely to be possible direct targets of miR-7 in CHO cells. This is the first report investigating the impact of a miRNA on the proteome of CHO cells.

24 ||| 22389098 ||| (1, 0.90) ||| 1 ||| 8 ||| Utilization and evaluation of CHO-specific sequence databases for mass spectrometry based proteomics. Recently released sequence information on Chinese hamster ovary (CHO) cells promises to not only facilitate our understanding of these industrially important cell factories through direct analysis of the sequence, but also to enhance existing methodologies and allow new tools to be developed. In this article we demonstrate the utilization of CHO specific sequence information to improve mass spectrometry (MS) based proteomic identification. The use of various CHO specific databases enabled the identification of 282 additional proteins, thus increasing the total number of identified proteins by 40-50%, depending on the sample source and methods used. In addition, a considerable portion of those proteins that were identified previously based on inter-species sequence homology were now identified by a larger number of peptides matched, thus increasing the confidence of identification. The new sequence information offers improved interpretation of proteomic analyses and will, in the years to come, prove vital to unraveling the CHO proteome.

25 ||| 24227746 ||| (1, 0.89) ||| 1 ||| 8 ||| Microarray platform affords improved product analysis in mammalian cell growth studies. High throughput (HT) platforms serve as a cost-efficient and rapid screening method for evaluating the effect of cell-culture conditions and screening of chemicals. We report the development of a HT cell-based microarray platform to assess the effect of culture conditions on Chinese hamster ovary (CHO) cells. Specifically, growth, transgene expression and metabolism of a GS/methionine sulphoximine (MSX) CHO cell line, which produces a therapeutic monoclonal antibody, was examined using a microarray system in conjunction with a conventional shake flask platform in a non-proprietary medium. The microarray system consists of 60-nL spots of cells encapsulated in alginate and separated in groups via an 8-well chamber system attached to the chip. Results show the non-proprietary medium developed allows cell growth, production, and normal glycosylation of recombinant antibody and metabolism of the recombinant CHO cells in both the microarray and shake flask platforms. In addition, 10.3 mM glutamate addition to the defined base medium results in lactate metabolism shift in the recombinant GS/MSX CHO cells in the shake flask platform. Ultimately, the results demonstrate that the HT microarray platform has the potential to be utilized for evaluating the impact of media additives on cellular processes, such as cell growth, metabolism, and productivity.

26 ||| 20945494 ||| (1, 0.89) ||| 1 ||| 8 ||| Maximizing productivity of CHO cell-based fed-batch culture using chemically defined media conditions and typical manufacturing equipment. A highly productive chemically defined fed-batch process was developed to maximize titer and volumetric productivity for Chinese hamster ovary cell-based recombinant protein manufacturing. Two cell lines producing a recombinant antibody (cell line A) and an Fc-fusion protein (cell line B) were used for development. Both processes achieved product titers of 10 g/L on day 18 under chemically defined conditions. For cell line B, the use of plant derived hydrolysates combined with the optimized chemically defined medium increased the titer to 13 g/L. Volumetric productivities were increased from a base line of about 200 mg/L/d to about 500 mg/L/d under chemically defined conditions and as high as 700 mg/L/d with cell line B using plant derived hydrolysates. Peak cell densities reached greater than 20E6 vc/mL, and cell viabilities were maintained above 80% on day 18 without the use of antiapoptotic genes or temperature shift. A rapid compound screening method was developed to effectively test positive factors within 72 h. Peak volumetric oxygen uptake rates (OUR) more than tripled from the baseline condition. Oxygen demand continued to increase after maximum cell density was reached with a maximal OUR of 3.7 mmol/L/h. The new process format was scaled up and verified at 100 L pilot scale using reactor equipment of similar configuration as used at manufacturing scale.

27 ||| 25450749 ||| (1, 0.87) ||| 1 ||| 8 ||| Global insights into the Chinese hamster and CHO cell transcriptomes. Transcriptomics is increasingly being used on Chinese hamster ovary (CHO) cells to unveil physiological insights related to their performance during production processes. The rich transcriptome data can be exploited to provide impetus for systems investigation such as modeling the central carbon metabolism or glycosylation pathways, or even building genome-scale models. To harness the power of transcriptome assays, we assembled and annotated a set of RNA-Seq data from multiple CHO cell lines and Chinese hamster tissues, and constructed a DNA microarray. The identity of genes involved in major functional pathways and their transcript levels generated in this study will serve as a reference for future studies employing kinetic models. In particular, the data on glycolysis and glycosylation pathways indicate that the variability of gene expression level among different cell lines and tissues may contribute to their differences in metabolism and glycosylation patterns. Thereby, these insights can potentially lead to opportunities for cell engineering. This repertoire of transcriptome data also enables the identification of potential sequence variants in cell lines and allows tracing of cell lineages. Overall the study is an illustration of the potential benefit of RNA-Seq that is yet to be exploited.

28 ||| 24997444 ||| (1, 0.87) ||| 1 ||| 8 ||| Exploiting the proteomics revolution in biotechnology: from disease and antibody targets to optimizing bioprocess development. Recent advancements in proteomics have enabled the generation of high-quality data sets useful for applications ranging from target and monoclonal antibody (mAB) discovery to bioprocess optimization. Comparative proteomics approaches have recently been used to identify novel disease targets in oncology and other disease conditions. Proteomics has also been applied as a new avenue for mAb discovery. Finally, CHO and Escherichia coli cells represent the dominant production hosts for biopharmaceutical development, yet the physiology of these cells types has yet to be fully established. Proteomics approaches can provide new insights into these cell types, aiding in recombinant protein production, cell growth regulation, and medium formulation. Optimization of sample preparations and protein database developments are enhancing the quantity and accuracy of proteomic results. In these ways, innovations in proteomics are enriching biotechnology and bioprocessing research across a wide spectrum of applications.

29 ||| 22556165 ||| (1, 0.87) ||| 1 ||| 8 ||| Analysis of dynamic changes in the proteome of a Bcl-XL overexpressing Chinese hamster ovary cell culture during exponential and stationary phases. Mammalian cell cultures used for biopharmaceutical production undergo various dynamic biological changes over time, including the transition of cells from an exponential growth phase to a stationary phase during cell culture. To better understand the dynamic aspects of cell culture, a quantitative proteomics approach was used to identify dynamic trends in protein expression over the course of a Chinese hamster ovary (CHO) cell culture for the production of a recombinant monoclonal antibody and overexpressing the antiapoptotic gene Bcl-xl. Samples were analyzed using a method incorporating iTRAQ labeling, two-dimensional LC/MS, and linear regression calculations to identify significant dynamic trends in protein abundance. Using this approach, 59 proteins were identified with significant temporal changes in expression. Pathway analysis tools were used to identify a putative network of proteins associated with cell growth and apoptosis. Among the differentially expressed proteins were molecular chaperones and isomerases, such as GRP78 and PDI, and reported cell growth markers MCM2 and MCM5. In addition, two proteins with growth-regulating properties, transglutaminase-2 and clusterin, were identified. These proteins are associated with tumor proliferation and apoptosis and were observed to be expressed at relatively high levels during stationary phase, which was confirmed by western blotting. The proteomic methodology described here provides a dynamic view of protein expression throughout a CHO fed-batch cell culture, which may be useful for further elucidating the biological processes driving mammalian cell culture performance.

30 ||| 21751394 ||| (1, 0.87) ||| 1 ||| 8 ||| Dynamic mRNA and miRNA profiling of CHO-K1 suspension cell cultures. In spite of the importance of Chinese hamster ovary (CHO) cells for recombinant protein production, very little is known about the molecular and gene regulatory mechanisms that control cellular phenotypes such as enhanced growth under serum-free conditions or high productivity. Most microarray analyses to this purpose are performed with samples taken during the exponential growth phase. However, the cellular transcriptome is dynamic, changing in response to external and internal stimuli and thus reflecting the current functional capacity of cells as well as their ability to adapt to a changing environment. Therefore, during batch or fed-batch cultivations it can be expected that the transcription pattern of genes will change and that such changes may give indications on the cellular state in terms of viability, growth, and productivity. In the current study we monitored the change in expression patterns of mRNAs and microRNAs (miRNA) during lag, exponential, and stationary phases in CHO-K1 suspension cell cultures. In total, over 1400 mRNAs and more than 100 miRNAs were differentially regulated (p<0.05) relative to the batch culture at the starting point. Functional clustering revealed groups of genes with similar expression patterns, which were subjected to functional pathway analysis. In addition, as miRNAs generally act as negative post-transcriptional regulators of mRNAs, we looked for changes in their expression that were inverse to those of their predicted target mRNAs.

31 ||| 17390381 ||| (1, 0.87) ||| 1 ||| 8 ||| A scaffold for the Chinese hamster genome. Chinese hamster ovary (CHO) cells are a prevalent tool in biological research and are among the most widely used host cell lines for production of recombinant therapeutic proteins. While research in other organisms has been revolutionized through the development of DNA sequence-based tools, the lack of comparable genomic resources for the Chinese hamster has impeded similar work in CHO cell lines. A comparative genomics approach, based upon the completely sequenced mouse genome, can facilitate genomic work in this important organism. Using chromosome synteny to define regions of conserved linkage between Chinese hamster and mouse chromosomes, a working scaffold for the Chinese hamster genome has been developed. Mapping CHO and Chinese hamster sequences to the mouse genome creates direct access to relevant information in public databases. Additionally, mapping gene expression data onto a chromosome scaffold affords the ability to interpret information in a genomic context, potentially revealing important structural and regulatory features in the Chinese hamster genome. Further development of this genomic scaffold will provide opportunities to use biomolecular tools for research in CHO cell lines today and will be an asset to future efforts to sequence the Chinese hamster genome.

32 ||| 17172658 ||| (1, 0.87) ||| 1 ||| 8 ||| Transcriptome and proteome profiling to understanding the biology of high productivity CHO cells. A combined transcriptome and proteome analysis was carried out to identify key genes and proteins differentially expressed in Chinese hamster ovary (CHO) cells producing high and low levels of dhfr-GFP fusion protein. Comparison of transcript levels was performed using a proprietary 15K CHO cDNA microarray chip, whereas proteomic analysis was performed using iTRAQ quantitative protein profiling technique. Microarray analysis revealed 77 differentially expressed genes, with 53 genes upregulated and 24 genes downregulated. Proteomic analysis gave 75 and 80 proteins for the midexponential and stationary phase, respectively. Although there was a general lack of correlation between mRNA levels and quantitated protein abundance, results from both datasets concurred on groups of proteins/genes based on functional categorization. A number of genes (20%) and proteins (45 and 23%) were involved in processes related to protein biosynthesis. We also identified three genes/proteins involved in chromatin modification. Enzymes responsible for opening up chromatin, Hmgn3 and Hmgb1, were upregulated whereas enzymes that condense chromatin, histone H1.2, were downregulated. Genes and proteins that promote cell growth (Igfbp4, Ptma, S100a6, and Lgals3) were downregulated, whereas those that deter cell growth (Ccng2, Gsg2, and S100a11) were upregulated. Other main groups of genes and proteins include carbohydrate metabolism, signal transduction, and transport. Our findings show that an integrated genomic and proteomics approach can be effectively utilized to monitor transcriptional and posttranscriptional events of mammalian cells in culture.

33 ||| 24995573 ||| (1, 0.86) ||| 1 ||| 8 ||| Enhancement of production of protein biopharmaceuticals by mammalian cell cultures: the metabolomics perspective. The new era of metabolic study is enabling a molecular rationalisation of cellular processes that support high production and quality of recombinant protein in bioprocessing. The completeness of, and confidence in, our understanding of culture metabolism has permitted the development of metabolic models for recombinant cell lines that will facilitate enhancement of bioprocess performance. For Chinese Hamster ovary (CHO) cell lines, key indicators of metabolic events linked to productivity have become clear in the past 2-3 years. In particular, the balance between glycolysis and the citric acid cycle appears as a determinant/indicator of both growth and productivity. The profiles observed offer potential to direct CHO cell function during culture through medium design and/or cell engineering.

34 ||| 23322664 ||| (1, 0.86) ||| 1 ||| 8 ||| An 'omics approach towards CHO cell engineering. Chinese hamster ovarian cells (CHO) cells have been extensively utilized for industrial production of biopharmaceutical products, such as monoclonal antibodies, human growth hormones, cytokines, and blood-products. Recent advances in recombinant DNA technology have resulted in the bioengineering of CHO cells that have robust gene amplification systems and can also be adapted to grow in suspension cultures. In parallel, recent advances in techniques and tools for decoding the CHO cell genome, transcriptome, proteome, and glycome have led to new areas of study for better understanding the metabolic pathways in CHO cells with the long-term goal of developing new biologics. This review paper discusses the recent advances in bioengineering strategies in CHO cell lines and the impact of the knowledge gained by CHO cell genomics, transcriptomics, and glycomics on the future of CHO-cell engineering.

35 ||| 23065714 ||| (1, 0.86) ||| 1 ||| 8 ||| Investigation of alginate-epsilon-poly-L-lysine microcapsules for cell microencapsulation. Cell microencapsulation is a promising approach for cell implantation, cell-based gene therapy, and large-scale cell culture. The well-studied alpha-AP (alginate-alpha-poly-L-lysine) microcapsules have been restricted to large-scale cell-culture and clinical applications because of high costs and cytotoxic effects in some cases. This study used epsilon-poly-L-lysine (epsilon-PLL), a high-biocompatible and low-cost food additives produced by fermentation, to prepare epsilon-AP (alginate-epsilon-PLL) microcapsules with various thickness membranes and swelling behaviors. epsilon-AP microcapsules were permeable to BSA, a standard protein of culture medium. epsilon-AP-microencapsulated Chinese hamster ovary (CHO) cells proliferated with culture time; no obvious difference with alpha-AP-microencapsulated CHO cells during the early 19 days. Whereas epsilon-AP-microencapsulated CHO cells kept higher viability (OD = 0.646 +- 0.012) on the 22nd day and microcapsule strength (integrity rate of 88%) on the 24th day than that of alpha-AP microcapsules (OD = 0.558 +- 0.025, integrity rate of 80%). epsilon-AP (alginate-epsilon-PLL) microcapsules exhibited more superior properties and could lower the costs to broaden the applications of microencapsulation technology.

36 ||| 20830683 ||| (1, 0.85) ||| 1 ||| 8 ||| Conserved microRNAs in Chinese hamster ovary cell lines. MicroRNAs (miRNAs), a class of short (20-24 nt) non-coding RNAs that direct post-transcriptional repression of messenger RNAs, increasingly have been shown to play a key role in regulating cellular physiology. We investigated the prevalence of miRNAs in Chinese hamster ovary (CHO) cells by high-throughput sequencing. Six cDNA libraries of small RNAs from four CHO cell lines were constructed and sequenced by Illumina sequencing. Three hundred fifty distinct miRNA and miRNA\* sequences were identified through homology with other species, including mouse, rat, and human. While the majority of the identified miRNAs appear to be expressed ubiquitously, many miRNAs were found to have a wide range of expression levels between cell lines. The identification of these miRNAs will facilitate investigations of their contribution to the hyperproductivity trait.

37 ||| 22252269 ||| (1, 0.81) ||| 1 ||| 8 ||| Combined in silico modeling and metabolomics analysis to characterize fed-batch CHO cell culture. The increasing demand for recombinant therapeutic proteins highlights the need to constantly improve the efficiency and yield of these biopharmaceutical products from mammalian cells, which is fully achievable only through proper understanding of cellular functioning. Towards this end, the current study exploited a combined metabolomics and in silico modeling approach to gain a deeper insight into the cellular mechanisms of Chinese hamster ovary (CHO) fed-batch cultures. Initially, extracellular and intracellular metabolite profiling analysis shortlisted key metabolites associated with cell growth limitation within the energy, glutathione, and glycerophospholipid pathways that have distinct changes at the exponential-stationary transition phase of the cultures. In addition, biomass compositional analysis newly revealed different amino acid content in the CHO cells from other mammalian cells, indicating the significance of accurate protein composition data in metabolite balancing across required nutrient assimilation, metabolic utilization, and cell growth. Subsequent in silico modeling of CHO cells characterized internal metabolic behaviors attaining physiological changes during growth and non-growth phases, thereby allowing us to explore relevant pathways to growth limitation and identify major growth-limiting factors including the oxidative stress and depletion of lipid metabolites. Such key information on growth-related mechanisms derived from the current approach can potentially guide the development of new strategies to enhance CHO culture performance.

38 ||| 19373452 ||| (1, 0.81) ||| 1 ||| 8 ||| Approaches to optimizing animal cell culture process: substrate metabolism regulation and protein expression improvement. Some high value proteins and vaccines for medical and veterinary applications by animal cell culture have an increasing market in China. In order to meet the demands of large-scale productions of proteins and vaccines, animal cell culture technology has been widely developed. In general, an animal cell culture process can be divided into two stages in a batch culture. In cell growth stage a high specific growth rate is expected to achieve a high cell density. In production stage a high specific production rate is stressed for the expression and secretion of qualified protein or replication of virus. It is always critical to maintain high cell viability in fed-batch and perfusion cultures. More concern has been focused on two points by the researchers in China. First, the cell metabolism of substrates is analyzed and the accumulation of toxic by-products is decreased through regulating cell metabolism in the culture process. Second, some important factors effecting protein expression are understood at the molecular level and the production ability of protein is improved. In pace with the rapid development of large-scale cell culture for the production of vaccines, antibodies and other recombinant proteins in China, the medium design and process optimization based on cell metabolism regulation and protein expression improvement will play an important role. The chapter outlines the main advances in metabolic regulation of cell and expression improvement of protein in animal cell culture in recent years.

39 ||| 17298905 ||| (1, 0.81) ||| 1 ||| 8 ||| Comparative transcriptional analysis of mouse hybridoma and recombinant Chinese hamster ovary cells undergoing butyrate treatment. DNA microarray based transcriptome analysis has become widely used in biomedical research; however, the lack of DNA sequence information available for Chinese hamster ovary (CHO) cells has hampered the application of microarrays for this cell line widely used for recombinant therapeutic protein production. We have constructed an expressed sequence tag (EST) based CHO DNA microarray and employed it for comparative transcriptome analysis of CHO cells and mouse hybridoma cells treated with sodium butyrate. Cross-species hybridization of CHO transcripts to mouse DNA microarrays was also performed to assess the utility of cross-species microarray. The average identity among probe sequences present on both the CHO and mouse microarray was 89.6%. Although cross-species hybridization yielded non-contradicting results when compared with the same-species arrays, decreased sensitivity was observed and resulted in fewer differentially expressed genes being confidently identified. The comparatively small number of genes probed using the CHO microarray and the low number of genes identified as differentially expressed in the cross-species hybridization limited physiological interpretation of the response of CHO cells to sodium butyrate treatment. Nevertheless, when all results are combined, mouse hybridoma and CHO cells can be seen to respond similarly to butyrate treatment, affecting histone modification, chaperones, lipid metabolism, and protein processing. To further develop the utility of microarray technology in cell culture process development, an expansion of current CHO cell sequencing efforts to increase the coverage of genes on available microarrays is warranted.

40 ||| 23554412 ||| (1, 0.80) ||| 1 ||| 8 ||| Improvement of mammalian cell culture performance through surfactant enabled concentrated feed media. The design of basal and feed media in mammalian cell culture is paramount towards ensuring acceptable upstream process performance in various operation modes, especially fed-batch culture. Mammalian cell culture media designs have evolved from the classical formulations designed by Eagle and Ham, to today's formulations designed from continuous improvement and statistical frameworks. Feed media is especially important for ensuring robust cell growth, productivity, and ensuring the product quality of recombinant therapeutics are within acceptable ranges. Numerous studies have highlighted the benefit of various media designs, supplements, and feed addition strategies towards the resulting cell culture process. In this work we highlight the use of a top-down level approach towards feed media design enabled by the use of select surfactants for the targeted enrichment of a chemically defined feed media. The use of the enriched media was able to improve product titers at g/L levels, without adversely impacting the growth of multiple Chinese Hamster Ovary cell lines or the product quality of multiple recombinant antibodies.

41 ||| 21290218 ||| (1, 0.80) ||| 1 ||| 8 ||| Advancing biopharmaceutical process development by system-level data analysis and integration of omics data. Development of efficient bioprocesses is essential for cost-effective manufacturing of recombinant therapeutic proteins. To achieve further process improvement and process rationalization comprehensive data analysis of both process data and phenotypic cell-level data is essential. Here, we present a framework for advanced bioprocess data analysis consisting of multivariate data analysis (MVDA), metabolic flux analysis (MFA), and pathway analysis for mapping of large-scale gene expression data sets. This data analysis platform was applied in a process development project with an IgG-producing Chinese hamster ovary (CHO) cell line in which the maximal product titer could be increased from about 5 to 8 g/L.Principal component analysis (PCA), k-means clustering, and partial least-squares (PLS) models were applied to analyze the macroscopic bioprocess data. MFA and gene expression analysis revealed intracellular information on the characteristics of high-performance cell cultivations. By MVDA, for example, correlations between several essential amino acids and the product concentration were observed. Also, a grouping into rather cell specific productivity-driven and process control-driven processes could be unraveled. By MFA, phenotypic characteristics in glycolysis, glutaminolysis, pentose phosphate pathway, citrate cycle, coupling of amino acid metabolism to citrate cycle, and in the energy yield could be identified. By gene expression analysis 247 deregulated metabolic genes were identified which are involved, inter alia, in amino acid metabolism, transport, and protein synthesis.

42 ||| 24381145 ||| (1, 0.79) ||| 1 ||| 8 ||| Addition of valproic acid to CHO cell fed-batch cultures improves monoclonal antibody titers. Improving the productivity of a biopharmaceutical Chinese hamster ovary (CHO) fed-batch cell culture can enable cost savings and more efficient manufacturing capacity utilization. One method for increasing CHO cell productivity is the addition of histone deacetylase (HDAC) inhibitors to the cell culture process. In this study, we examined the effect of valproic acid (VPA, 2-propylpentanoic acid), a branched-chain carboxylic acid HDAC inhibitor, on the productivity of three of our CHO cell lines that stably express monoclonal antibodies. Fed-batch shake flask VPA titrations on the three different CHO cell lines yielded cell line-specific results. Cell line A responded highly positively, cell line B responded mildly positively, and cell line C did not respond. We then performed factorial experiments to identify the optimal VPA concentration and day of addition for cell line A. After identifying the optimal conditions for cell line A, we performed verification experiments in fed-batch bioreactors for cell lines A and B. These experiments confirmed that a high dose of VPA late in the culture can increase harvest titer >20 % without greatly changing antibody aggregation, charge heterogeneity, and N-linked glycosylation profiles. Our results suggest that VPA is an attractive and viable small molecule enhancer of protein production for biopharmaceutical CHO cell culture processes.

43 ||| 21115077 ||| (1, 0.79) ||| 1 ||| 8 ||| Predicting cell-specific productivity from CHO gene expression. Improving the rate of recombinant protein production in Chinese hamster ovary (CHO) cells is an important consideration in controlling the cost of biopharmaceuticals. We present the first predictive model of productivity in CHO bioprocess culture based on gene expression profiles. The dataset used to construct the model consisted of transcriptomic data from 70 stationary phase, temperature-shifted CHO production cell line samples, for which the cell-specific productivity had been determined. These samples were utilised to investigate gene expression over a range of high to low monoclonal antibody and fc-fusion-producing CHO cell lines. We utilised a supervised regression algorithm, partial least squares (PLS) incorporating jackknife gene selection, to produce a model of cell-specific productivity (Qp) capable of predicting Qp to within 4.44 pg/cell/day root mean squared error in cross model validation (RMSE(CMV)). The final model, consisting of 287 genes, was capable of accurately predicting Qp in a further panel of 10 additional samples which were incorporated as an independent validation. Several of the genes constituting the model are linked with biological processes relevant to protein metabolism.

44 ||| 23563583 ||| (1, 0.78) ||| 1 ||| 8 ||| A high-throughput media design approach for high performance mammalian fed-batch cultures. An innovative high-throughput medium development method based on media blending was successfully used to improve the performance of a Chinese hamster ovary fed-batch medium in shaking 96-deepwell plates. Starting from a proprietary chemically-defined medium, 16 formulations testing 43 of 47 components at 3 different levels were designed. Media blending was performed following a custom-made mixture design of experiments considering binary blends, resulting in 376 different blends that were tested during both cell expansion and fed-batch production phases in one single experiment. Three approaches were chosen to provide the best output of the large amount of data obtained. A simple ranking of conditions was first used as a quick approach to select new formulations with promising features. Then, prediction of the best mixes was done to maximize both growth and titer using the Design Expert software. Finally, a multivariate analysis enabled identification of individual potential critical components for further optimization. Applying this high-throughput method on a fed-batch, rather than on a simple batch, process opens new perspectives for medium and feed development that enables identification of an optimized process in a short time frame.

45 ||| 22306111 ||| (1, 0.78) ||| 1 ||| 8 ||| Computational identification of microRNA gene loci and precursor microRNA sequences in CHO cell lines. MicroRNAs (miRNAs) have recently entered Chinese hamster ovary (CHO) cell culture technology, due to their severe impact on the regulation of cellular phenotypes. Applications of miRNAs that are envisioned range from biomarkers of favorable phenotypes to cell engineering targets. These applications, however, require a profound knowledge of miRNA sequences and their genomic organization, which exceeds the currently available information of ~400 conserved mature CHO miRNA sequences. Based on these recently published sequences and two independent CHO-K1 genome assemblies, this publication describes the computational identification of CHO miRNA genomic loci. Using BLAST alignment, 415 previously reported CHO miRNAs were mapped to the reference genomes, and subsequently assigned to a distinct genomic miRNA locus. Sequences of the respective precursor-miRNAs were extracted from both reference genomes, folded in silico to verify correct structures and cross-compared. In the end, 212 genomic loci and pre-miRNA sequences representing 319 expressed mature miRNAs (approximately 50% of miRNAs represented matching pairs of 5' and 3' miRNAs) were submitted to the miRBase miRNA repository. As a proof-of-principle for the usability of the published genomic loci, four likely polycistronic miRNA cluster were chosen for PCR amplification using CHO-K1 and DHFR (-) genomic DNA. Overall, these data on the genomic context of miRNA expression in CHO will simplify the development of tools employing stable overexpression or deletion of miRNAs, allow the identification of miRNA promoters and improve detection methods such as microarrays.

46 ||| 19557832 ||| (1, 0.78) ||| 1 ||| 8 ||| Using microarray technology to select housekeeping genes in Chinese hamster ovary cells. In the present study, we have identified species-specific housekeeping genes (HKGs) for Chinese Hamster Ovary (CHO) cells using data from microarray gene expression profiling. HKGs suitable for quantitative RT-PCR normalization should display relatively stable expression levels across experimental conditions. We analyzed transcription profiles of several IgG-producing recombinant CHO cell lines under numerous culture conditions using a custom CHO DNA microarray platform and calculated relative expression variability from 124 microarrays. We selected a novel panel of candidate HKGs based on their low variability in expression from the microarray data. Compared to three traditional HKGs (Gapdh, Actb, and B2m), the majority of genes on this panel demonstrated lower or equal variability. Each candidate HKG was then validated using qRT-PCR. Final selection of CHO-specific HKGs include Actr5, Eif3i, Hirip3, Pabpn1, Vezt, Cog1, and Yaf2. The results reported here provide a useful tool for gene expression analyses in CHO cells, a critical expression platform used in biotherapeutics.

47 ||| 18814282 ||| (1, 0.78) ||| 1 ||| 8 ||| Quality assessment of cross-species hybridization of CHO transcriptome on a mouse DNA oligo microarray. DNA microarray technology has been widely utilized for species with extensive genome sequence information available. Given the limited genomic information pertaining to Chinese hamster ovary (CHO) cell line, cross-species hybridization using mouse microarrays provides a viable alternative. In this study, the utility of mouse Affymetrix microarrays for transcriptome profiling in CHO cells was assessed by hybridizing identical sets of cRNA samples from CHO cells on both mouse and CHO Affymetrix microarrays. Expression level measured by probe sets for orthologous transcripts on the two microarrays was compared. Only a fraction of the orthologous probes which detected expression calls in same species hybridization were similarly called present in cross species hybridization. In further analysis at the 25-mer probe level, it was revealed that specific hybridization signals were detectable by the subset of mouse probes that have a high degree of homology to the corresponding CHO sequences. The feasibility of cross species hybridization for quantifying the extent of differential expression was assessed by comparing transcript levels of CHO cells cultivated with and without sodium butyrate. While same species hybridization gave consistent degree of differential expression calls in replicated runs, a much inferior ability in quantifying differential expression was seen with cross species hybridization. Our results demonstrate that through detailed analysis of homology at the probe pair level, a subset of probes on existing mouse Affymetrix oligo-array can be used successfully for transcriptome profiling of CHO cells.

48 ||| 21626722 ||| (1, 0.77) ||| 1 ||| 8 ||| Performance of high intensity fed-batch mammalian cell cultures in disposable bioreactor systems. The adoption of disposable bioreactor technology as an alternate to traditional nondisposable technology is gaining momentum in the biotechnology industry. Evaluation of current disposable bioreactors systems to sustain high intensity fed-batch mammalian cell culture processes needs to be explored. In this study, an assessment was performed comparing single-use bioreactors (SUBs) systems of 50-, 250-, and 1,000-L operating scales with traditional stainless steel (SS) and glass vessels using four distinct mammalian cell culture processes. This comparison focuses on expansion and production stage performance. The SUB performance was evaluated based on three main areas: operability, process scalability, and process performance. The process performance and operability aspects were assessed over time and product quality performance was compared at the day of harvest. Expansion stage results showed disposable bioreactors mirror traditional bioreactors in terms of cellular growth and metabolism. Set-up and disposal times were dramatically reduced using the SUB systems when compared with traditional systems. Production stage runs for both Chinese hamster ovary and NS0 cell lines in the SUB system were able to model SS bioreactors runs at 100-, 200-, 2,000-, and 15,000-L scales. A single 1,000-L SUB run applying a high intensity fed-batch process was able to generate 7.5 kg of antibody with comparable product quality.

49 ||| 25061012 ||| (1, 0.75) ||| 1 ||| 8 ||| A functional high-content miRNA screen identifies miR-30 family to boost recombinant protein production in CHO cells. The steady improvement of mammalian cell factories for the production of biopharmaceuticals is a key challenge for the biotechnology community. Recently, small regulatory microRNAs (miRNAs) were identified as novel targets for optimizing Chinese hamster ovary (CHO) production cells as they do not add any translational burden to the cell while being capable of regulating entire physiological pathways. The aim of the present study was to elucidate miRNA function in a recombinant CHO-SEAP cell line by means of a genome-wide high-content miRNA screen. This screen revealed that out of the 1, 139 miRNAs examined, 21% of the miRNAs enhanced cell-specific SEAP productivity mainly resulting in elevated volumetric yields, while cell proliferation was accelerated by 5% of the miRNAs. Conversely, cell death was diminished by 13% (apoptosis) or 4% (necrosis) of all transfected miRNAs. Besides these large number of identified target miRNAs, the outcome of our studies suggest that the entire miR-30 family substantially improves bioprocess performance of CHO cells. Stable miR-30 over expressing cells outperformed parental cells by increasing SEAP productivity or maximum cell density of approximately twofold. Our results highlight the application of miRNAs as powerful tools for CHO cell engineering, identified the miR-30 family as a critical component of cell proliferation, and support the notion that miRNAs are powerful determinants of cell viability.

50 ||| 23639388 ||| (1, 0.75) ||| 1 ||| 8 ||| Prediction of transcribed PIWI-interacting RNAs from CHO RNAseq data. Chinese hamster ovary (CHO) cells are currently the most important mammalian host for the manufacture of biopharmaceuticals. To enhance our understanding of cellular processes, pathways, and the genetic setup of CHO cell lines, we predicted PIWI interacting RNAs (piRNAs) from small RNA sequencing data. Although piRNAs are the least understood class of small non-coding RNAs that mediate RNA silencing, it is believed that they play a pivotal role in protecting genome integrity by repressing transposable elements. Since genomic integrity is the key to prolonged stability of recombinant CHO cell lines, we characterized piRNA sequences and expression in six CHO cell lines by computational analysis of an existing small RNA sequencing dataset using proTRAC and the published CHO genome as reference. Here we present the result of this analysis consisting of 25,626 piRNAs and 540 piRNA clusters. Moreover we provide first evidence for differential piRNA expression in adherent and suspension-adapted CHO-K1 and DUKXB11 host cell lines as well as their recombinant derivatives, indicating that piRNAs might be tools for cell line development and engineering.

51 ||| 25527440 ||| (1, 0.74) ||| 1 ||| 8 ||| Conserved microRNA function as a basis for Chinese hamster ovary cell engineering. The use of microRNAs (miRNAs) for improving the efficiency of recombinant protein production by CHO cells is gaining considerable interest for their ability to regulate entire molecular networks. Differential miRNA expression profiling and large-scale transient screening have been the prerequisite for the selection of miRNA candidates for stable manipulation, reported in CHO cells expressing a range of recombinant products. We selected a potent and well characterised tumour suppressor miRNA, miR-34a, as a high priority candidate for CHO cell engineering based on the conservation of both its sequence and function across species and cell type. Ectopic expression of miR-34a retained its functional conservation in CHO-SEAP cells by inhibiting growth by 90% in addition to decreasing the viable cell population by 30% when compared to controls. When the miR-34 family was stably depleted using a miRNA sponge decoy vector, the overall product yield was enhanced by ~2-fold in both fed-batch and small scale clonal batch cultures, despite having a negative impact on cell growth. These findings further strengthen the utility of miRNAs as engineering tools to modify and improve CHO cell performance.

52 ||| 24533650 ||| (1, 0.74) ||| 1 ||| 8 ||| The relationship between mTOR signalling pathway and recombinant antibody productivity in CHO cell lines. BACKGROUND: High recombinant protein productivity in mammalian cell lines is often associated with phenotypic changes in protein content, energy metabolism, and cell growth, but the key determinants that regulate productivity are still not clearly understood. The mammalian target of rapamycin (mTOR) signalling pathway has emerged as a central regulator for many cellular processes including cell growth, apoptosis, metabolism, and protein synthesis. This role of this pathway changes in response to diverse environmental cues and allows the upstream proteins that respond directly to extracellular signals (such as nutrient availability, energy status, and physical stresses) to communicate with downstream effectors which, in turn, regulate various essential cellular processes. RESULTS: In this study, we have performed a transcriptomic analysis using a pathway-focused polymerase chain reaction (PCR) array to compare the expression of 84 target genes related to the mTOR signalling in two recombinant CHO cell lines with a 17.4-fold difference in specific monoclonal antibody productivity (qp). Eight differentially expressed genes that exhibited more than a 1.5-fold change were identified. Pik3cd (encoding the Class 1A catalytic subunit of phosphatidylinositol 3-kinase [PI3K]) was the most differentially expressed gene having a 71.3-fold higher level of expression in the high producer cell line than in the low producer. The difference in the gene's transcription levels was confirmed at the protein level by examining expression of p110delta. CONCLUSION: Expression of p110delta correlated with specific productivity (qp) across six different CHO cell lines, with a range of expression levels from 3 to 51 pg/cell/day, suggesting that p110delta may be a key factor in regulating productivity in recombinant cell lines.

53 ||| 17914862 ||| (1, 0.74) ||| 1 ||| 8 ||| Novel orbital shake bioreactors for transient production of CHO derived IgGs. Large-scale transient gene expression in mammalian cells is being developed for the rapid production of recombinant proteins for biochemical and preclinical studies. Here, the scalability of transient production of a recombinant human antibody in Chinese hamster ovary (CHO) cells was demonstrated in orbitally shaken disposable bioreactors at scales from 50 mL to 50 L. First, a small-scale multiparameter approach was developed to optimize the poly(ethylenimine)-mediated transfection in 50 mL shake tubes. This study confirmed the benefit, both in terms of extended cell culture viability and increased product yield, of mild hypothermic cultivation conditions for transient gene expression in CHO cells. Second, the scalability of the process was demonstrated in disposable shake bioreactors having nominal volumes of 5, 20, and 50 L with final antibody yields between 30 and 60 mg L(-1). Thus, the combination of transient gene expression with disposable shake bioreactors allows for rapid and cost-effective production of recombinant proteins in CHO cells.

54 ||| 25194670 ||| (1, 0.73) ||| 1 ||| 8 ||| Gene expression measurements normalized to cell number reveal large scale differences due to cell size changes, transcriptional amplification and transcriptional repression in CHO cells. Conventional approaches to differential gene expression comparisons assume equal cellular RNA content among experimental conditions. We demonstrate that this assumption should not be universally applied because total RNA yield from a set number of cells varies among experimental treatments of the same Chinese Hamster Ovary (CHO) cell line and among different CHO cell lines expressing recombinant proteins. Conventional normalization strategies mask these differences in cellular RNA content and, consequently, skew biological interpretation of differential expression results. On the contrary, normalization to synthetic spike-in RNA standards added proportional to cell numbers reveals these differences and allows detection of global transcriptional amplification/repression. We apply this normalization method to assess differential gene expression in cell lines of different sizes, as well as cells treated with a cell cycle inhibitor (CCI), an mTOR inhibitor (mTORI), or subjected to high osmolarity conditions. CCI treatment of CHO cells results in a cellular volume increase and global transcriptional amplification, while mTORI treatment causes global transcriptional repression without affecting cellular volume. Similarly to CCI treatment, high osmolarity increases cell size, total RNA content and antibody expression. Furthermore, we show the importance of spike-in normalization for studies involving multiple CHO cell lines and advocate normalization to spike-in controls prior to correlating gene expression to specific productivity (qP). Overall, our data support the need for cell number specific spike-in controls for all gene expression studies where cellular RNA content differs among experimental conditions.

55 ||| 24861053 ||| (1, 0.73) ||| 1 ||| 8 ||| Precision genome editing: a small revolution for glycobiology. Precise and stable gene editing in mammalian cell lines has until recently been hampered by the lack of efficient targeting methods. While different gene silencing strategies have had tremendous impact on many biological fields, they have generally not been applied with wide success in the field of glycobiology, primarily due to their low efficiencies, with resultant failure to impose substantial phenotypic consequences upon the final glycosylation products. Here, we review novel nuclease-based precision genome editing techniques enabling efficient and stable gene editing, including gene disruption, insertion, repair, modification and deletion. The nuclease-based techniques comprised of homing endonucleases, zinc finger nucleases, transcription activator-like effector nucleases, as well as the RNA-guided clustered regularly interspaced short palindromic repeat/Cas nuclease system, all function by introducing single or double-stranded breaks at a defined genomic sequence. We here compare and contrast the different techniques and summarize their current applications, highlighting cases from the field of glycobiology as well as pointing to future opportunities. The emerging potential of precision gene editing for the field is exemplified by applications to xenotransplantation; to probing O-glycoproteomes, including differential O-GalNAc glycoproteomes, to decipher the function of individual polypeptide GalNAc-transferases, as well as for engineering Chinese Hamster Ovary host cells for production of improved therapeutic biologics.

56 ||| 22767053 ||| (1, 0.73) ||| 1 ||| 8 ||| Automated dynamic fed-batch process and media optimization for high productivity cell culture process development. Current industry practices for large-scale mammalian cell cultures typically employ a standard platform fed-batch process with fixed volume bolus feeding. Although widely used, these processes are unable to respond to actual nutrient consumption demands from the culture, which can result in accumulation of by-products and depletion of certain nutrients. This work demonstrates the application of a fully automated cell culture control, monitoring, and data processing system to achieve significant productivity improvement via dynamic feeding and media optimization. Two distinct feeding algorithms were used to dynamically alter feed rates. The first method is based upon on-line capacitance measurements where cultures were fed based on growth and nutrient consumption rates estimated from integrated capacitance. The second method is based upon automated glucose measurements obtained from the Nova Bioprofile FLEX autosampler where cultures were fed to maintain a target glucose level which in turn maintained other nutrients based on a stoichiometric ratio. All of the calculations were done automatically through in-house integration with a Delta V process control system. Through both media and feed strategy optimization, a titer increase from the original platform titer of 5 to 6.3 g/L was achieved for cell line A, and a substantial titer increase of 4 to over 9 g/L was achieved for cell line B with comparable product quality. Glucose was found to be the best feed indicator, but not all cell lines benefited from dynamic feeding and optimized feed media was critical to process improvement. Our work demonstrated that dynamic feeding has the ability to automatically adjust feed rates according to culture behavior, and that the advantage can be best realized during early and rapid process development stages where different cell lines or large changes in culture conditions might lead to dramatically different nutrient demands.

57 ||| 24249214 ||| (1, 0.72) ||| 1 ||| 8 ||| Cell line profiling to improve monoclonal antibody production. Mammalian cell culture performance is influenced by both intrinsic (genetic) and extrinsic (media and process) factors. In this study, intrinsic capacity of various monoclonal antibody-producing Chinese Hamster Ovary (CHO) cell lines was compared by exposing them to the same culture condition. Microarray-based transcriptomics and LC-MS/MS shotgun proteomics technologies were utilized to obtain expression landscape of different cell lines. Specific transcripts and proteins correlating with productivity, growth rate and cell size have been identified. The proteomics analysis results showed a strong correlation between the intracellular protein expression levels of the recombinant DHFR and productivity. In contrast, neither the light chain nor the heavy chain of the recombinant monoclonal antibody showed correlation to productivity. Other top ranked proteins which demonstrated positive correlation to productivity included the adaptor protein complex subunits AP3D1and AP2B2, DNA repair protein DDB1 and the ER translocation complex component, SRPR. The subunits of molecular chaperone T-complex protein 1 and the regulator of mitochondrial one-carbon metabolism MTHFD2 showed negative correlation to productivity. The transcriptomics analysis has identified the regulators of calcium signaling, Tmem20 and Rcan1, as the top ranked genes displaying positive and negative correlation to productivity, respectively. For the second part of the study, the principal component analysis (PCA) was generated to view the underlying global structure of the expression data. A clear division and expression polarity was observed between the two distinct clusters of cell lines, independent of link to productivity or any other traits examined. The primary component of the PCA generated from either transcriptomics or proteomics data displayed a strong correlation to cell size and doubling time, while none of the main principal components showed correlation to productivity. Our findings suggest that productivity is rather a minor feature in the context of global transcriptional or protein expression space.

58 ||| 24055788 ||| (1, 0.71) ||| 1 ||| 8 ||| Dynamic gene expression for metabolic engineering of mammalian cells in culture. Recombinant mammalian cells are the major hosts for the production of protein therapeutics. In addition to high expression of the product gene, a hyper-producer must also harbor superior phenotypic traits related to metabolism, protein secretion, and growth control. Introduction of genes endowing the relevant hyper-productivity traits is a strategy frequently used to enhance the productivity. Most of such cell engineering efforts have been performed using constitutive expression systems. However, cells respond to various environmental cues and cellular events dynamically according to cellular needs. The use of inducible systems allows for time dependent expression, but requires external manipulation. Ideally, a transgene's expression should be synchronous to the host cell's own rhythm, and at levels appropriate for the objective. To that end, we identified genes with different expression dynamics and intensity ranges using pooled transcriptome data. Their promoters may be used to drive the expression of the transgenes following the desired dynamics. We isolated the promoter of the Thioredoxin-interacting protein (Txnip) gene and demonstrated its capability to drive transgene expression in concert with cell growth. We further employed this Chinese hamster promoter to engineer dynamic expression of the mouse GLUT5 fructose transporter in Chinese hamster ovary (CHO) cells, enabling them to utilize sugar according to cellular needs rather than in excess as typically seen in culture. Thus, less lactate was produced, resulting in a better growth rate, prolonged culture duration, and higher product titer. This approach illustrates a novel concept in metabolic engineering which can potentially be used to achieve dynamic control of cellular behaviors for enhanced process characteristics.

59 ||| 24858576 ||| (1, 0.70) ||| 1 ||| 8 ||| Rapid high-throughput characterisation, classification and selection of recombinant mammalian cell line phenotypes using intact cell MALDI-ToF mass spectrometry fingerprinting and PLS-DA modelling. Despite many advances in the generation of high producing recombinant mammalian cell lines over the last few decades, cell line selection and development is often slowed by the inability to predict a cell line's phenotypic characteristics (e.g. growth or recombinant protein productivity) at larger scale (large volume bioreactors) using data from early cell line construction at small culture scale. Here we describe the development of an intact cell MALDI-ToF mass spectrometry fingerprinting method for mammalian cells early in the cell line construction process whereby the resulting mass spectrometry data are used to predict the phenotype of mammalian cell lines at larger culture scale using a Partial Least Squares Discriminant Analysis (PLS-DA) model. Using MALDI-ToF mass spectrometry, a library of mass spectrometry fingerprints was generated for individual cell lines at the 96 deep well plate stage of cell line development. The growth and productivity of these cell lines were evaluated in a 10L bioreactor model of Lonza's large-scale (up to 20,000L) fed-batch cell culture processes. Using the mass spectrometry information at the 96 deep well plate stage and phenotype information at the 10L bioreactor scale a PLS-DA model was developed to predict the productivity of unknown cell lines at the 10L scale based upon their MALDI-ToF fingerprint at the 96 deep well plate scale. This approach provides the basis for the very early prediction of cell lines' performance in cGMP manufacturing-scale bioreactors and the foundation for methods and models for predicting other mammalian cell phenotypes from rapid, intact-cell mass spectrometry based measurements.

60 ||| 24518263 ||| (1, 0.70) ||| 1 ||| 8 ||| Stable overexpression of miR-17 enhances recombinant protein production of CHO cells. miRNAs negatively regulate gene expression at post-transcriptional level, and consequently play an important role in the control of many cellular pathways. The use of miRNAs to engineer Chinese hamster ovary (CHO) cells is an emerging strategy to improve recombinant protein production. Here, we describe the effect of transient and stable miRNA overexpression on CHO cell phenotype. Using an established transient miRNA screening protocol, the effects of miR-17, miR-92a and cluster miR17-92a on CHO growth and protein productivity were studied and followed by analysis of cell pools with stable overexpression of these miRNAs. CHO cells stably engineered with miR-17 exhibited both enhanced growth performance and a 2-fold increase in specific productivity, which resulted in a 3-fold overall increase in EpoFc titer. While further studies of miRNA-mRNA interactions will be necessary to understand the molecular basis of this effect, these data provide valuable evidence for miR-17 as a cell engineering target to enhance CHO cell productivity.

61 ||| 23646721 ||| (1, 0.70) ||| 1 ||| 8 ||| Multiarray formation of CHO spheroids cocultured with feeder cells for highly efficient protein production in serum-free medium. Functional proteins like antibody, cytokine and growth factor have been widely used for basic biological research, diagnosis and cancer therapy. Particularly, antibody drugs as attractive biopharmaceuticals will be expected to create an enormous new market. Chinese hamster ovay (CHO) cells are being increasingly used in industry for the production of recombinant therapeutic proteins including antibody drugs. Although three-dimensional culture is preferred to two-dimensional monolayer culture for the efficient large scale culture of CHO cells and subsequent mass production of recombinant proteins, it has the limitation of low protein production. Therefore, a new cell culture em essentially required for an efficient protein production. Here we report on a new three-dimensional cell culture system as a spheroid cell culture on the micropattern array for efficient production of protein in CHO cells. Furthermore, cocultivation of CHO spheroids with feeder cells including bovine aortic endothelial cells (BAEC) and NIH 3T3 was essential to more increase a protein production. The results indicated that CHO heterospheroids cocultured with BAECs were much superior to either CHO monolayers or CHO homospheroids in protein production. Significantly, the above cocultured spheroids in the serum-free medium drastically enhanced protein expression level up to 3-fold compared with CHO spheroids in serum medium, suggesting that a coculture of spheroid system with feeder layer cells is a promising method to enhance protein production under serum-free condition. The spheroid array constructed here is highly usuful as a platform of biopharmaceutical manufacturing as well as tissue and cell based biosensors to detect a wide variety of clinically active compounds through a cellular physiological response.

62 ||| 16187333 ||| (1, 0.70) ||| 1 ||| 8 ||| Initial transcriptome and proteome analyses of low culture temperature-induced expression in CHO cells producing erythropoietin. Low culture temperature is known to enhance the specific productivity of Chinese hamster ovary (CHO) cells expressing erythropoietin (EPO) (LGE10-9-27). Genomic and proteomic approaches were taken to better understand the intracellular responses of these CHO cells resulting from use of low culture temperature (33 degrees C). For transcriptome analysis, commercially available rat and mouse cDNA microarrays were used. The data obtained from the rat and mouse cDNA chips were only somewhat informative in understanding the gene expression profile of CHO cells because of their different sequence homologies with CHO transcriptomes. Overall, transcriptome analysis revealed that low culture temperature could lead to changes in gene expression in various cellular processes such as metabolism, transport, and signaling pathways. Proteome analysis was carried out using 2-D PAGE. Based on spot intensity, 60 high intensity protein spots, from a total of more than 800, were chosen for MS analysis. Forty of the 60 protein spots, which represent 26 different kinds of proteins, were identified by MALDI-TOF-MS and validated by MS/MS. Compared to the reference temperature (37 degrees C), the expression levels of seven proteins (PDI, vimentin, NDK B, ERp57, RIKEN cDNA, phosphoglycerate kinase, and heat shock cognate 71 kDa protein) were increased over twofold at 33 degrees C and those of two proteins (HSP90-beta and EF2) were decreased over twofold at 33 degrees C. Taken together, the results demonstrate the potential of combined analysis of transcriptome and proteome analyses as a tool for the systematic comprehension of cellular mechanisms in CHO cells.

63 ||| 24777991 ||| (1, 0.69) ||| 1 ||| 8 ||| Modulation of mAb quality attributes using microliter scale fed-batch cultures. A high-throughput DoE approach performed in a 96-deepwell plate system was used to explore the impact of media and feed components on main quality attributes of a monoclonal antibody. Six CHO-S derived clonal cell lines expressing the same monoclonal antibody were tested in two different cell culture media with six components added at three different levels. The resulting 384 culture conditions including controls were simultaneously tested in fed-batch conditions, and process performance such as viable cell density, viability, and product titer were monitored. At the end of the culture, supernatants from each condition were purified and the product was analyzed for N-glycan profiles, charge variant distribution, aggregates, and low molecular weight forms. The screening described here provided highly valuable insights into the factors and combination of factors that can be used to modulate the quality attributes of a molecule. The approach also revealed specific intrinsic differences of the selected clonal cell lines - some cell lines were very responsive in terms of changes in performance or quality attributes, whereas others were less affected by the factors tested in this study. Moreover, it indicated to what extent the attributes can be impacted within the selected experimental design space. The outcome correlated well with confirmations performed in larger cell culture volumes such as small-scale bioreactors. Being fast and resource effective, this integrated high-throughput approach can provide information which is particularly useful during early stage cell culture development.

64 ||| 24613301 ||| (1, 0.69) ||| 1 ||| 8 ||| Transcriptome analyses of CHO cells with the next-generation microarray CHO41K: development and validation by analysing the influence of the growth stimulating substance IGF-1 substitute LongR(3.). The increasing importance of Chinese hamster ovary (CHO) cells for the production of pharmaceutical proteins has awakened the demand to understand the cellular metabolism of these cells. However, satisfactory gene expression studies have yet been impractical due to insufficient coverage of sequences. In this work, previously determined sequence information of CHO cells and newly derived data from 454 and Illumina sequencing was used to establish the CHO41K microarray which contains 41,304 probes. Self-hybridisation was performed for replica determination and samples were run in triplicates to increase statistical power. For determination of technical variance, confidence intervals at an M-value of +-0.6 for 95% and at +-2.3 for 99% of the probes were calculated. Intra-microarray and slide to slide variance was not detectable. In a first application, this microarray enabled an in-depth look inside the cellular transcriptome of CHO cells cultured in the presence or absence of the growth supporting substance "insulin like growth factor 1" (IGF-1) analogue LongR(3). Its effect on the cells ranged from enhanced growth to delay of cell death as well as cytoskeletal installation. Suggesting that under supplementation, a minimised cellular effort in installation of a large cytoskeleton occurs, possibly in favour of promoting faster cell division.

65 ||| 23511307 ||| (1, 0.69) ||| 1 ||| 8 ||| Cell electroporation by CNT-featured microfluidic chip. We present the application of carbon nanotubes (CNTs) for cell electroporation that is performed in a microfluidic device. Lab on a chip (LOC) developments have raised unique possibilities to scale down cell manipulation systems to a cellular level to achieve higher performance and accuracy. Among the systems employed for cell disruption, electroporation without chemical reagents provides many advantages but suffers from high voltage requirements. We have exploited the electric field enhancement by CNTs to realize low-voltage electroporation. A microchip with embedded aligned CNTs has been developed to test the effect of the enhanced electric field on electroporation of mammalian CHO cells. Fluorogenic Calcein AM dye is used to image the release of the intercellular medium as an indication of electroporation. The electroporation phenomenon is recorded in real-time and compared with that of a device without CNTs. The results show that at a voltage as low as 3 volts, the electroporation yield rate is increased by 72% with the incorporation of CNTs. This enhancement is a promising advancement towards integration of low-voltage electroporation with other low-voltage cell manipulation techniques.

66 ||| 19902412 ||| (1, 0.69) ||| 1 ||| 8 ||| Metabolomics profiling of extracellular metabolites in recombinant Chinese Hamster Ovary fed-batch culture. A metabolomics-based approach was used to time profile extracellular metabolites in duplicate fed-batch bioreactor cultures of recombinant Chinese Hamster Ovary (CHO) cells producing monoclonal IgG antibody. Culture medium was collected and analysed using a high-performance liquid chromatography (HPLC) system in tandem with an LTQ-Orbitrap mass spectrometer. An in-house software was developed to pre-process the LC/MS data in terms of filtering and peak detection. This was followed by principal component analysis (PCA) to assess variance amongst the samples, and hierarchical clustering to categorize mass peaks by their time profiles. Finally, LC/MS2 experiments using the LTQ-Orbitrap (where standard was available) and SYNAPT HDMS (where standard was unavailable) were performed to confirm the identities of the metabolites. Two groups of identified metabolites were of particular interest; the first consisted of metabolites that began to accumulate when the culture entered stationary phase. The majority of them were amino acid derivatives and they were likely to be derived from the amino acids in the feed media. Examples included acetylphenylalanine and dimethylarginine which are known to be detrimental to cell growth. The second group of metabolites showed a downward trend as the culture progressed. Two of them were medium components--tryptophan and choline, and these became depleted midway into the culture despite the addition of feed media. The findings demonstrated the potential of utilizing metabolomics to guide medium design for fed-batch culture to potentially improve cell growth and product titer.

67 ||| 19777580 ||| (1, 0.69) ||| 1 ||| 8 ||| BAK and BAX deletion using zinc-finger nucleases yields apoptosis-resistant CHO cells. Anoxic and metabolic stresses in large-scale cell culture during biopharmaceutical production can induce apoptosis. Strategies designed to ameliorate the problem of apoptosis in cell culture have focused on mRNA knockdown of pro-apoptotic proteins and over-expression of anti-apoptotic ones. Apoptosis in cell culture involves mitochondrial permeabilization by the pro-apoptotic Bak and Bax proteins; activity of either protein is sufficient to permit apoptosis. We demonstrate here the complete and permanent elimination of both the Bak and Bax proteins in combination in Chinese hamster ovary (CHO) cells using zinc-finger nuclease-mediated gene disruption. Zinc-finger nuclease cleavage of BAX and BAK followed by inaccurate DNA repair resulted in knockout of both genes. Cells lacking Bax and Bak grow normally but fail to activate caspases in response to apoptotic stimuli. When grown using scale-down systems under conditions that mimic growth in large-scale bioreactors they are significantly more resistant to apoptosis induced by starvation, staurosporine, and sodium butyrate. When grown under starvation conditions, BAX- and BAK-deleted cells produce two- to fivefold more IgG than wild-type CHO cells. Under normal growth conditions in suspension culture in shake flasks, double-knockout cultures achieve equal or higher cell densities than unmodified wild-type cultures and reach viable cell densities relevant for large-scale industrial protein production.

68 ||| 25495469 ||| (1, 0.68) ||| 1 ||| 8 ||| High-antibody-producing Chinese hamster ovary cells up-regulate intracellular protein transport and glutathione synthesis. Chinese hamster ovary (CHO) cells are the preferred production host for therapeutic monoclonal antibodies (mAb) due to their ability to perform post-translational modifications and their successful approval history. The completion of the genome sequence for CHO cells has reignited interest in using quantitative proteomics to identify markers of good production lines. Here we applied two different proteomic techniques, iTRAQ and SWATH, for the identification of expression differences between a high- and low-antibody-producing CHO cell lines derived from the same transfection. More than 2000 proteins were quantified with 70 of them classified as differentially expressed in both techniques. Two biological processes were identified as differentially regulated by both methods: up-regulation of glutathione biosynthesis and down-regulation of DNA replication. Metabolomic analysis confirmed that the high producing cell line displayed higher intracellular levels of glutathione. SWATH further identified up-regulation of actin filament processes and intracellular transport and down regulation of several growth-related processes. These processes may be important for conferring high mAb production and as such are promising candidates for targeted engineering of high-expression cell lines.

69 ||| 23994267 ||| (1, 0.68) ||| 1 ||| 8 ||| Breaking limitations of complex culture media: functional non-viral miRNA delivery into pharmaceutical production cell lines. MicroRNAs (miRNAs) are promising targets for cell engineering through modulation of crucial cellular pathways. An effective introduction of miRNAs into the cell is a prerequisite to reliably study microRNA function. Previously, non-viral delivery of nucleic acids has been demonstrated to be cell type as well as culture medium dependent. Due to their importance for biopharmaceutical research and manufacturing, Chinese hamster ovary (CHO) and Cevec's Amniocyte Production (CAP) cells were used as host cell lines to investigate transfection reagents with respect to successful delivery of small non-coding RNAs (ncRNAs) and their ability to allow for biological activity of miRNAs and small interfering RNAs (siRNAs) within the cell. In the present study, we screened numerous transfection reagents for their suitability to successfully deliver miRNA mimics into CHO DG44 and CAP cells. Our investigation revealed that the determination of transfection efficiency for a given transfection reagent alone is not sufficient to draw conclusions about its ability to maintain the functionality of the miRNA. We could show that independent from high transfection rates observed for several reagents only one was suitable for efficient introduction of functional miRNA mimics into cells cultured in complex protein production media. We provide evidence for the functionality of transferred ncRNAs by demonstrating siRNA-mediated changes in protein levels and cellular phenotype as well as decreased twinfilin-1 (twf-1) transcript levels by its upstream miR-1 regulator. Furthermore, the process could be shown to be scalable which has important implications for biotechnological applications.

70 ||| 21915848 ||| (1, 0.68) ||| 1 ||| 8 ||| RNA interference of cofilin in Chinese hamster ovary cells improves recombinant protein productivity. RNA interference (RNAi) has been recently applied to improve the yield and quality of recombinant proteins produced in Chinese hamster ovary (CHO) cells, the most commonly used mammalian cell line for production of complex biopharmaceuticals. Proteomic profiling of CHO cells undergoing gene amplification identified cofilin, a key regulatory protein of actin cytoskeletal dynamics, as a cellular target for genetic engineering studies. Transient reduction of cofilin by small interfering RNA (siRNA) enhanced specific productivity in recombinant CHO cells by up to 80%. CHO cell lines expressing cofilin-specific short hairpin RNA (shRNA) vectors showed up to a 65% increase in specific productivity. These results suggest that modulation of cofilin, and its regulatory pathways, may be a new approach to enhance recombinant protein productivity in CHO cells.

71 ||| 18546152 ||| (1, 0.68) ||| 1 ||| 8 ||| Proteomic profiling of CHO cells with enhanced rhBMP-2 productivity following co-expression of PACEsol. Chinese hamster ovary (CHO) cells are widely used for the production of recombinant protein biopharmaceuticals. The purpose of this study was to investigate differences in the proteome of CHO DUKX cells expressing recombinant human bone morphogenetic protein-2 (rhBMP-2) (G5 cells) compared to cells also expressing soluble exogenous paired basic amino acid cleaving enzyme soluble paired basic amino acid cleaving enzyme (PACEsol) (3C9 cells), which has been previously found to improve the post-translational processing of the mature rhBMP-2 dimer. PACEsol co-expression was also associated with a significant increase (almost four-fold) in cellular productivity of rhBMP-2 protein. Differential proteomic expression profiling using 2-D DIGE and MALDI-TOF MS was performed to compare 3C9 and G5 cells, and revealed a list of 60 proteins that showed differential expression (up/downregulated), with a variety of different cellular functions. A substantial number of these altered proteins were found to have chaperone activity, involved with protein folding, assembly and secretion, as well as a number of proteins involved in protein translation. These results support the use of proteomic profiling as a valuable tool towards understanding the biology of bioprocess cultures.

72 ||| 24144501 ||| (1, 0.67) ||| 1 ||| 8 ||| Stable microRNA expression enhances therapeutic antibody productivity of Chinese hamster ovary cells. MicroRNAs (miRNAs) are short non-coding RNAs that post-transcriptionally regulate the expression of different target genes and, thus, enable engineered gene networks to achieve complex phenotypic changes in mammalian cells. We hypothesized that exploiting this feature of miRNAs could improve therapeutic protein production processes by increasing viable cell densities and/or productivity of the mammalian cells used for manufacturing. To identify miRNAs that increase the productivity of producer cells, we performed a genome wide functional miRNA screen by transient transfection of Chinese hamster ovary (CHO) cells stably expressing an IgG1 antibody (CHO-IgG1). Using this approach, we identified nine human miRNAs that improved the productivities not only of the CHO-IgG1 cells but also of CHO cells expressing recombinant human serum albumin (HSA), demonstrating that the miRNAs act in a product-independent manner. We selected two miRNAs (miR-557 and miR-1287) positively impacting the viable cell density and the specific productivity, respectively, and then stably co-expressed them in IgG1 expressing CHO cells. In these cells, higher IgG1 titers were observed in fed-batch cultures whilst product quality was conserved, demonstrating that miRNA-based cell line engineering provides an attractive approach toward the genetic optimization of CHO producer cells for industrial applications.

73 ||| 22971049 ||| (1, 0.67) ||| 1 ||| 8 ||| Proteomic analysis of Chinese hamster ovary cells. To complement the recent genomic sequencing of Chinese hamster ovary (CHO) cells, proteomic analysis was performed on CHO cells including the cellular proteome, secretome, and glycoproteome using tandem mass spectrometry (MS/MS) of multiple fractions obtained from gel electrophoresis, multidimensional liquid chromatography, and solid phase extraction of glycopeptides (SPEG). From the 120 different mass spectrometry analyses generating 682,097 MS/MS spectra, 93,548 unique peptide sequences were identified with at most 0.02 false discovery rate (FDR). A total of 6164 grouped proteins were identified from both glycoproteome and proteome analysis, representing an 8-fold increase in the number of proteins currently identified in the CHO proteome. Furthermore, this is the first proteomic study done using the CHO genome exclusively, which provides for more accurate identification of proteins. From this analysis, the CHO codon frequency was determined and found to be distinct from humans, which will facilitate expression of human proteins in CHO cells. Analysis of the combined proteomic and mRNA data sets indicated the enrichment of a number of pathways including protein processing and apoptosis but depletion of proteins involved in steroid hormone and glycosphingolipid metabolism. Five-hundred four of the detected proteins included N-acetylation modifications, and 1292 different proteins were observed to be N-glycosylated. This first large-scale proteomic analysis will enhance the knowledge base about CHO capabilities for recombinant expression and provide information useful in cell engineering efforts aimed at modifying CHO cellular functions.

74 ||| 21286710 ||| (1, 0.67) ||| 1 ||| 8 ||| Proteomic understanding of intracellular responses of recombinant Chinese hamster ovary cells cultivated in serum-free medium supplemented with hydrolysates. In order to understand the intracellular responses in recombinant CHO (rCHO) cells producing antibody in serum-free medium (SFM) supplemented with optimized hydrolysates mixtures, yielding the highest specific growth rate (mu, SFM#S1) or the highest specific antibody productivity (q(Ab,) SFM#S2), differentially expressed proteins in rCHO cells are measured by two-dimensional gel electrophoresis combined with nano-LC-ESI-Q-TOF tandem MS. The comparative proteomic analysis with basal SFM without hydrolysates revealed that the addition of hydrolysate mixtures significantly altered the profiles of CHO proteome. In SFM#S1, the expression of metabolism-related proteins, cytoskeleton-associated proteins, and proliferation-related proteins was up-regulated. On the other hand, the expression of anti-proliferative proteins and pro-apoptotic protein was down-regulated. In SFM#S2, the expression of various chaperone proteins and proliferation-linked proteins was altered. 2D-Western blot analysis of differentially expressed proteins confirmed the proteomic results. Taken together, identification of differentially expressed proteins in CHO cells by a proteomic approach can provide insights into understanding the effect of hydrolysates on intracellular events and clues to find candidate genes for cell engineering to maximize the protein production in rCHO cells.

75 ||| 19663468 ||| (1, 0.67) ||| 1 ||| 8 ||| Proteomic profiling of a high-producing Chinese hamster ovary cell culture. The productivity of mammalian cell culture expression systems is critically important to the production of biopharmaceuticals. In this study, a high-producing Chinese hamster ovary cell culture which was transfected with the apoptosis inhibitor Bcl-X(L) gene was compared to a low-producing control that was not transfected. Shotgun proteomics was used to compare the high and low-producing fed-batch cell cultures at different growth time points. The goals of this study were twofold; it would be of value to find a biomarker that could predict cell lines with higher growth efficiency and to gain mechanistic insights into the effects of the introduction of a foreign gene that is known to have growth regulating properties in human cells. A total of 392 proteins were identified in this study, and 32 of these proteins were determined to be differentially expressed. In the high-producing cell culture, several proteins related to protein metabolism were upregulated, such as eukaryotic translation initiation factor 3 and ribosome 40S. In addition, several intermediate filament proteins such as vimentin and annexin, as well as histone H1.2 and H2A, were downregulated in the high producer. The expression of these proteins may be indicative of cellular productivity. A growth inhibitor, galectin-1, was downregulated in the high producer, which may be linked to the expression of Bcl-X(L). The molecular chaperone BiP was upregulated significantly in the high producer and may indicate an unfolded protein response due to endoplasmic reticulum (ER) stress. Several proteins involved in regulation of the cell cycle such as RACK1 and GTPase Ran were found to be differentially expressed, which may be due to a differentially controlled cell cycle between low- and high-producing cell cultures.

76 ||| 25218361 ||| (1, 0.65) ||| 1 ||| 8 ||| Adaptation for survival: phenotype and transcriptome response of CHO cells to elevated stress induced by agitation and sparging. In this work, the response and adaption of CHO cells to hydrodynamic stress in laboratory scale bioreactors originating from agitation, sparging and their combination is studied experimentally. First, the maximum hydrodynamic stress, tau(max), is characterized over a broad range of operating conditions using a shear sensitive particulate system. Separate stress regimes are determined, where tau(max) is controlled either by sparging, agitation, or their combination. Such conditions are consequently applied during cultivations of an industrial CHO cell line to determine the cellular responses to corresponding stresses. Our results suggest that the studied CHO cell line has different threshold values and response mechanisms for hydrodynamic stress resulting from agitation or sparging, respectively. For agitation, a characteristic local minimum in viability was found after stress induction followed by viability recovery, while at highest sparging stress a monotonic decrease in viability was observed. If both stresses were combined, also both characteristic stress responses could be observed, amplifying each other. On the other hand, cellular metabolism, productivity and product quality did not change significantly. Transcriptome analysis using mRNA microarrays confirmed that separate adaptation mechanisms are activated in the different stress situations studied, allowing identification of these stresses using a transcriptome fingerprinting approach. Functional analysis of the transcripts was consequently used to improve our understanding of the molecular mechanisms of shear stress response and adaptation.

77 ||| 24486028 ||| (1, 0.65) ||| 1 ||| 8 ||| Analysis of microRNA transcription and post-transcriptional processing by Dicer in the context of CHO cell proliferation. CHO cells are the mammalian cell line of choice for recombinant production of therapeutic proteins. However, their low rate of proliferation limits obtainable space-time yields due to inefficient biomass accumulation. We set out to correlate microRNA transcription to cell-specific growth-rate by microarray analysis of 5 CHO suspension cell lines with low to high specific growth rates. Global microRNA expression analysis and Pearson correlation studies showed that mature microRNA transcript levels are predominately up-regulated in a state of fast proliferation (46 positively correlated, 17 negatively correlated). To further validate this observation, the expression of three genes that are central to microRNA biogenesis (Dicer, Drosha and Dgcr8) was analyzed. The expression of Dicer, which mediates the final step in microRNA maturation, was found to be strongly correlated to growth rate. Accordingly, knockdown of Dicer impaired cell growth by reducing growth-correlating microRNA transcripts. Moderate ectopic overexpression of Dicer positively affected cell growth, while strong overexpression impaired growth, presumably due to the concomitant increase of microRNAs that inhibit cell growth. Our data therefore suggest that Dicer dependent microRNAs regulate CHO cell proliferation and that Dicer could serve as a potential surrogate marker for cellular proliferation.

78 ||| 22695856 ||| (1, 0.65) ||| 1 ||| 8 ||| Cell culture medium improvement by rigorous shuffling of components using media blending. A novel high-throughput methodology for the simultaneous optimization of many cell culture media components is presented. The method is based on the media blending approach which has several advantages as it works with ready-to-use media. In particular it allows precise pH and osmolarity adjustments and eliminates the need of concentrated stock solutions, a frequent source of serious solubility issues. In addition, media blending easily generates a large number of new compositions providing a remarkable screening tool. However, media blending designs usually do not provide information on distinct factors or components that are causing the desired improvements. This paper addresses this last point by considering the concentration of individual medium components to fix the experimental design and for the interpretation of the results. The extended blending strategy was used to reshuffle the 20 amino acids in one round of experiments. A small set of 10 media was specifically designed to generate a large number of mixtures. 192 mixtures were then prepared by media blending and tested on a recombinant CHO cell line expressing a monoclonal antibody. A wide range of performances (titers and viable cell density) was achieved from the different mixtures with top titers significantly above our previous results seen with this cell line. In addition, information about major effects of key amino acids on cell densities and titers could be extracted from the experimental results. This demonstrates that the extended blending approach is a powerful experimental tool which allows systematic and simultaneous reshuffling of multiple medium components.

79 ||| 22124969 ||| (1, 0.65) ||| 1 ||| 8 ||| Host cell protein dynamics in the supernatant of a mAb producing CHO cell line. The characterization of host cell protein (HCP) content during the production of therapeutic recombinant proteins is an important aspect in the drug development process. Despite this, key components of the HCP profile and how this changes with processing has not been fully investigated. Here we have investigated the supernatant HCP profile at different times throughout culture of a null and model GS-CHO monoclonal antibody producing mammalian cell line grown in fed-batch mode. Using 2D-PAGE and LC-MS/MS we identify a number of intracellular proteins (e.g., protein disulfide isomerise; elongation factor 2; calreticulin) that show a significant change in abundance relative to the general increase in HCP concentration observed with progression of culture. Those HCPs that showed a significant change in abundance across the culture above the general increase were dependent on the cell line examined. Further, our data suggests that the majority of HCPs in the supernatant of the cell lines investigated here arise through lysis or breakage of cells, associated with loss in viability, and are not present due to the secretion of protein material from within the cell. SELDI-TOF and principal components analysis were also investigated to enable rapid monitoring of changes in the HCP profile. SELDI-TOF analysis showed the same trends in the HCP profile as observed by 2D-PAGE analysis and highlighted biomarkers that could be used for process monitoring. These data further our understanding of the relationship between the HCP profile and cell viability and may ultimately enable a more directed development of purification strategies and the development of cell lines based upon their HCP profile.

80 ||| 18626961 ||| (1, 0.65) ||| 1 ||| 8 ||| Two-dimensional electrophoresis of proteins as a tool in the metabolic engineering of cell cycle regulation. Metabolic engineering of cell cycle regulation addresses important biotechnological problems about serum removal from animal cell culture systems. Chinese hamster ovary cells stimulated to grow by fetal calf serum, insulin, or basic fibroblast growth factor were studied by two-dimensional electrophoresis (2DE) and the resulting protein expression patterns were analyzed. Detailed 2DE protocols are provided and at least 24 gene products are identified which may play an important role in growth factor signaling. Moreover, a correlation between the expression of three proteins (cyclin D1, cyclin E, and E2F-1) and mitogenic strength was found.

81 ||| 17570552 ||| (1, 0.65) ||| 1 ||| 8 ||| Initial identification of low temperature and culture stage induction of miRNA expression in suspension CHO-K1 cells. This paper describes the first miRNA analysis carried out on hamster cells specifically Chinese hamster ovary (CHO) cells which are the most important cell line for the manufacture of human recombinant biopharmaceutical products. During biphasic culture, an initial phase of rapid cell growth at 37 degrees C is followed by a growth arrest phase induced through reduction of the culture temperature. Growth arrest is associated with many positive phenotypes including increased productivity, sustained viability and an extended production phase. Using miRNA bioarrays generated with probes against human, mouse and rat miRNAs, we have identified 26 differentially expressed miRNAs in CHO-K1 when comparing cells undergoing exponential growth at 37 degrees C to stationary phase cells at 31 degrees C. Five miRNAs were selected for qRT-PCR analysis using specific primer sets to isolate and amplify mature miRNAs. During this analysis, two known growth inhibitory miRNAs, miR-21 and miR-24 were identified as being upregulated during stationary phase growth induced either by temperature shift or during normal batch culture by both bioarray and qRT-PCR. Sequence data confirmed the identity of cgr-miR-21, a novel Cricetulus griseus ortholog of the known miRNA miR-21. This study offers a novel insight into the potential of miRNA regulation of CHO-K1 growth and may provide novel approaches to rational engineering of both cell lines and culture processes to ensure optimal conditions for recombinant protein production.

82 ||| 25384465 ||| (1, 0.64) ||| 1 ||| 8 ||| Evaluating the impact of high Pluronic F68 concentrations on antibody producing CHO cell lines. Pluronic F68 (P-F68) is an important component of chemically-defined cell culture medium because it protects cells from hydrodynamic and bubble-induced shear in the bioreactor. While P-F68 is typically used in cell culture medium at a concentration of 1 g/L (0.1%), higher concentrations can offer additional shear protection and have also been shown to be beneficial during cryopreservation. Recent industry experience with variability in P-F68-associated shear-protection has opened up the possibility of elevated P-F68 concentrations in cell culture media, a topic that has not been previously explored in the context of industrial cell culture processes. Recognizing this gap, we first evaluated the effect of 1-5 g/L P-F68 concentrations in shake flask cultures over ten 3-day passages for cell lines A and B. Increase in terminal cell density and cell size was seen over time at higher P-F68 concentrations but protein productivity was not impacted. Results from this preliminary screening study suggested no adverse impact of high P-F68 concentrations. Subsequently fed-batch bioreactor experiments were conducted at 1 and 5 g/L P-F68 concentrations with both cell lines where cell growth, viability, metabolism, and product quality were examined under process conditions reflective of a commercial process. Results from these bioreactor experiments confirmed findings from the preliminary screen and also indicated no impact of elevated P-F68 concentration on product quality. If additional shear protection is desired, either due to raw material variability, cell line sensitivity, or a high-shear cell culture process, our results suggest this can be accomplished by elevating the P-F68 concentration in the cell culture medium without impacting cell culture performance and product quality.

83 ||| 24888712 ||| (1, 0.64) ||| 1 ||| 8 ||| CHOPPI: a web tool for the analysis of immunogenicity risk from host cell proteins in CHO-based protein production. Despite high quality standards and continual process improvements in manufacturing, host cell protein (HCP) process impurities remain a substantial risk for biological products. Even at low levels, residual HCPs can induce a detrimental immune response compromising the safety and efficacy of a biologic. Consequently, advanced-stage clinical trials have been cancelled due to the identification of antibodies against HCPs. To enable earlier and rapid assessment of the risks in Chinese Hamster Ovary (CHO)-based protein production of residual CHO protein impurities (CHOPs), we have developed a web tool called CHOPPI, for CHO Protein Predicted Immunogenicity. CHOPPI integrates information regarding the possible presence of CHOPs (expression and secretion) with characterizations of their immunogenicity (T cell epitope count and density, and relative conservation with human counterparts). CHOPPI can generate a report for a specified CHO protein (e.g., identified from proteomics or immunoassays) or characterize an entire specified subset of the CHO genome (e.g., filtered based on confidence in transcription and similarity to human proteins). The ability to analyze potential CHOPs at a genomic scale provides a baseline to evaluate relative risk. We show here that CHOPPI can identify clear differences in immunogenicity risk among previously validated CHOPs, as well as identify additional "risky" CHO proteins that may be expressed during production and induce a detrimental immune response upon delivery. We conclude that CHOPPI is a powerful tool that provides a valuable computational complement to existing experimental approaches for CHOP risk assessment and can focus experimental efforts in the most important directions. Biotechnol. Bioeng. 2014;111: 2170-2182. 2014 Wiley Periodicals, Inc.

84 ||| 23596153 ||| (1, 0.64) ||| 1 ||| 8 ||| Resilient immortals, characterizing and utilizing Bax/Bak deficient Chinese hamster ovary (CHO) cells for high titer antibody production. Cell death due to apoptosis is frequently observed in large-scale manufacturing of therapeutic proteins, and can reduce product accumulation in bioreactors. Several different strategies that involve overexpression of antiapoptotic or downregulation of proapoptotic proteins have been designed in attempt to curb this problem in Chinese hamster ovary (CHO) cell culture. However, each of these designs has their own shortcomings and limits, rendering them ineffective for large-scale protein production. Recently, we have reported generation of a Bax and Bak deficient dhfr(-/-) CHO cell line using zinc-finger nucleases. Here we demonstrate that puromycin, but not methotrexate, selection can be used to generate antibody-expressing Bax and Bak deficient clones that are not only resistant to apoptosis, but that can also achieve higher titers relative to parental CHO cells due to higher cell density. Additionally, we show that Bax and Bak deficient cells have more mitochondria with healthy membrane potential, an attribute that perhaps contributes to their more potent growth compared to parental cells. Bax and Bak deficient cells do not readily apoptose, as shown by the ability to withstand high concentrations of apoptosis inducing agents, such as sodium butyrate, without a reduction in viability, growth, or titer. These traits render Bax and Bak deficient cells a potentially attractive host for production of therapeutic proteins at industrial scale.

85 ||| 25857574 ||| (1, 0.63) ||| 1 ||| 8 ||| A quantitative proteomic analysis of cellular responses to high glucose media in Chinese hamster ovary cells. A goal in recombinant protein production using Chinese hamster ovary (CHO) cells is to achieve both high specific productivity and high cell density. Addition of glucose to the culture media is necessary to maintain both cell growth and viability. We varied the glucose concentration in the media from 5 to 16 g/L and found that although specific productivity of CHO-DG44 cells increased with the glucose level, the integrated viable cell density decreased. To examine the biological basis of these results, we conducted a discovery proteomic study of CHO-DG44 cells grown under batch conditions in normal (5 g/L) or high (15 g/L) glucose over 3, 6, and 9 days. Approximately 5,000 proteins were confidently identified against an mRNA-based CHO-DG44 specific proteome database, with 2,800 proteins quantified with at least two peptides. A self-organizing map algorithm was used to deconvolute temporal expression profiles of quantitated proteins. Functional analysis of altered proteins suggested that differences in growth between the two glucose levels resulted from changes in crosstalk between glucose metabolism, recombinant protein expression, and cell death, providing an overall picture of the responses to high glucose environment. The high glucose environment may enhance recombinant dihydrofolate reductase in CHO cells by up-regulating NCK1 and down-regulating PRKRA, and may lower integrated viable cell density by activating mitochondrial- and endoplasmic reticulum-mediated cell death pathways by up-regulating HtrA2 and calpains. These proteins are suggested as potential targets for bioengineering to enhance recombinant protein production.

86 ||| 25086342 ||| (1, 0.63) ||| 1 ||| 8 ||| Discovery of transcription start sites in the Chinese hamster genome by next-generation RNA sequencing. Chinese hamster ovary (CHO) cell lines are one of the major production tools for monoclonal antibodies, recombinant proteins, and therapeutics. Although many efforts have significantly improved the availability of sequence information for CHO cells in the last years, forthcoming draft genomes still lack the information depth known from the mouse or human genomes. Many genes annotated for CHO cells and the Chinese hamster reference genome still are in silico predictions, only insufficiently verified by biological experiments. The correct annotation of transcription start sites (TSSs) is of special interest for CHO cells, as these directly define the location of the eukaryotic core promoter. Our study aims to elucidate these largely unexplored regions, trying to shed light on promoter landscapes in the Chinese hamster genome. Based on a 5' enriched dual library RNA sequencing approach 6547 TSSs were identified, of which over 90% were assigned to known genes. These TSSs were used to perform extensive promoter studies using a novel, modular bioinformatics pipeline, incorporating analyses of important regulatory elements of the eukaryotic core promoter on per-gene level and on genomic scale.

87 ||| 23873082 ||| (1, 0.63) ||| 1 ||| 8 ||| Genomic landscapes of Chinese hamster ovary cell lines as revealed by the Cricetulus griseus draft genome. Chinese hamster ovary (CHO) cells, first isolated in 1957, are the preferred production host for many therapeutic proteins. Although genetic heterogeneity among CHO cell lines has been well documented, a systematic, nucleotide-resolution characterization of their genotypic differences has been stymied by the lack of a unifying genomic resource for CHO cells. Here we report a 2.4-Gb draft genome sequence of a female Chinese hamster, Cricetulus griseus, harboring 24,044 genes. We also resequenced and analyzed the genomes of six CHO cell lines from the CHO-K1, DG44 and CHO-S lineages. This analysis identified hamster genes missing in different CHO cell lines, and detected >3.7 million single-nucleotide polymorphisms (SNPs), 551,240 indels and 7,063 copy number variations. Many mutations are located in genes with functions relevant to bioprocessing, such as apoptosis. The details of this genetic diversity highlight the value of the hamster genome as the reference upon which CHO cells can be studied and engineered for protein production.

88 ||| 23228731 ||| (1, 0.63) ||| 1 ||| 8 ||| Development of a Scale-Down Model of hydrodynamic stress to study the performance of an industrial CHO cell line under simulated production scale bioreactor conditions. The objective of this study was to develop a Scale-Down Model of a hydrodynamic stress present in large scale production bioreactors to investigate the performance of CHO cells under simulated production bioreactor conditions. Various levels of hydrodynamic stress were generated in 2L bioreactors mimicking those present in different locations of a large scale stirred tank bioreactor. In general, it was observed that tested cells are highly robust against the effect of hydrodynamic stress. However, at elevated hydrodynamic stress equivalent to an average energy dissipation rate, epsilon, equal to 0.4W/kg, the specific monoclonal antibody productivity, qmAb, decreased by 25% compared to the cultivation conditions corresponding to epsilon equal to 0.01W/kg. Even stronger decrease of qmAb, in the order of 30%, was observed when epsilon was periodically oscillating between 0.01 and 0.4W/kg to simulate the repeated passage of cells through the highly turbulent impeller discharge zone of a production scale bioreactor. Despite this effect, no changes in metabolite consumption or byproduct formation were observed. Furthermore, considering the experimental error product quality was independent of the applied epsilon. To achieve a molecular insight into the observed drop of cellular productivity, a transcriptome analysis using mRNA microarrays was performed. It was found that transcripts related to DNA damage and repair mechanisms were upregulated when high epsilon was applied for cultivation.

89 ||| 21269493 ||| (1, 0.63) ||| 1 ||| 8 ||| Genomic sequencing and analysis of a Chinese hamster ovary cell line using Illumina sequencing technology. BACKGROUND: Chinese hamster ovary (CHO) cells are among the most widely used hosts for therapeutic protein production. Yet few genomic resources are available to aid in engineering high-producing cell lines. RESULTS: High-throughput Illumina sequencing was used to generate a 1x genomic coverage of an engineered CHO cell line expressing secreted alkaline phosphatase (SEAP). Reference-guided alignment and assembly produced 3.57 million contigs and CHO-specific sequence information for ~ 18,000 mouse and ~ 19,000 rat orthologous genes. The majority of these genes are involved in metabolic processes, cellular signaling, and transport and represent attractive targets for cell line engineering. CONCLUSIONS: This demonstrates the applicability of next-generation sequencing technology and comparative genomic analysis in the development of CHO genomic resources.

90 ||| 24449637 ||| (1, 0.62) ||| 1 ||| 8 ||| High-throughput miniaturized bioreactors for cell culture process development: reproducibility, scalability, and control. Decreasing the timeframe for cell culture process development has been a key goal toward accelerating biopharmaceutical development. Advanced Microscale Bioreactors (ambr ) is an automated micro-bioreactor system with miniature single-use bioreactors with a 10-15 mL working volume controlled by an automated workstation. This system was compared to conventional bioreactor systems in terms of its performance for the production of a monoclonal antibody in a recombinant Chinese Hamster Ovary cell line. The miniaturized bioreactor system was found to produce cell culture profiles that matched across scales to 3 L, 15 L, and 200 L stirred tank bioreactors. The processes used in this article involve complex feed formulations, perturbations, and strict process control within the design space, which are in-line with processes used for commercial scale manufacturing of biopharmaceuticals. Changes to important process parameters in ambr resulted in predictable cell growth, viability and titer changes, which were in good agreement to data from the conventional larger scale bioreactors. ambr was found to successfully reproduce variations in temperature, dissolved oxygen (DO), and pH conditions similar to the larger bioreactor systems. Additionally, the miniature bioreactors were found to react well to perturbations in pH and DO through adjustments to the Proportional and Integral control loop. The data presented here demonstrates the utility of the ambr system as a high throughput system for cell culture process development.

91 ||| 12601807 ||| (1, 0.62) ||| 1 ||| 8 ||| Identification of cellular changes associated with increased production of human growth hormone in a recombinant Chinese hamster ovary cell line. A proteomics approach was used to identify the proteins potentially implicated in the cellular response concomitant with elevated production levels of human growth hormone in a recombinant Chinese hamster ovary (CHO) cell line following exposure to 0.5 mM butyrate and 80 microM zinc sulphate in the production media. This involved incorporation of two-dimensional (2-D) gel electrophoresis and protein identification by a combination of N-terminal sequencing, matrix-assisted laser desorption/ionisation-time of flight mass spectrometry, amino acid analysis and cross species database matching. From these identifications a CHO 2-D reference map and annotated database have been established. Metabolic labelling and subsequent autoradiography showed the induction of a number of cellular proteins in response to the media additives butyrate and zinc sulphate. These were identified as GRP75, enolase and thioredoxin. The chaperone proteins GRP78, HSP90, GRP94 and HSP70 were not up-regulated under these conditions.

92 ||| 25619381 ||| (1, 0.61) ||| 1 ||| 8 ||| Monensin, a small molecule ionophore, can be used to increase high mannose levels on monoclonal antibodies generated by Chinese hamster ovary production cell-lines. Asparagine-linked glycosylation of the constant region of monoclonal antibodies (mAbs) plays an important role in their stability and efficacy and is a critical product quality attribute that needs to be consistent between various process changes and production lots. Exact product quality match is also of the utmost importance for the development of biosimilar protein therapeutics. This poses a process development challenge since mAb glycosylation profiles can fluctuate easily with changes in process parameters. Therefore, there is a need to identify methods to modulate glycosylation levels on therapeutic antibodies during a production run in order to maintain consistent product quality profiles between different drug lots. Here, we demonstrate the use of a small molecule ionophore, monensin, to increase high mannose levels on multiple therapeutic human immunoglobulins (IgGs) in both plate-based small scale production models as well as in production bioreactors. This method is simple to implement and readily applicable for multiple production cell lines. Moreover, high mannose levels can be increased without significant negative impact on titer or cell culture performance. As such, monensin gives us a manipulable product quality lever.

93 ||| 24323929 ||| (1, 0.61) ||| 1 ||| 8 ||| Endogenous microRNA clusters outperform chimeric sequence clusters in Chinese hamster ovary cells. MicroRNAs (miRNAs) are small non-coding RNAs (~22 nucleotides) which regulate gene expression by silencing mRNA translation. MiRNAs are transcribed as long primary transcripts, which are enzymatically processed by Drosha/Dgcr8, in the nucleus, and by Dicer in the cytoplasm, into mature miRNAs. The importance of miRNAs for coordinated gene expression is commonly accepted. Consequentially, there is a growing interest in the application of miRNAs to improve phenotypes of mammalian cell factories such as Chinese hamster ovary (CHO) cells. Few studies have reported the targeted over-expression of miRNAs in CHO cells using vector-based systems. These approaches were hampered by limited sequence availability, and required the design of "chimeric" miRNA genes, consisting of the mature CHO miRNA sequence encompassed by murine flanking and loop sequences. Here we show that the substitution of chimeric sequences with CHO-specific sequences for expression of miRNA clusters yields significantly higher expression levels of the mature miRNA in the case of miR-221/222 and miR-15b/16. Our data suggest that the Drosha/Dgcr8-mediated excision from primary transcripts is reduced for chimeric miRNA sequences compared to the endogenous sequence. Overall, this study provides important guidelines for the targeted over-expression of clustered miRNAs in CHO cells. See accompanying commentary by Baik and Lee DOI: 10.1002/biot.201300503.

94 ||| 24157712 ||| (1, 0.61) ||| 1 ||| 8 ||| Understanding translational control mechanisms of the mTOR pathway in CHO cells by polysome profiling. The mammalian target of rapamycin (mTOR) pathway plays essential roles in the regulation of translational activity in many eukaryotes. Thus, from a bioprocessing point of view, understanding its molecular mechanisms may provide potential avenues for improving cell culture performance. Toward this end, the mTOR pathway of CHO cells in batch cultures was subjected to rapamycin treatment (inhibition) or nutrient supplementation (induction) and translational activities of CHO cells producing a monoclonal antibody (mAb) were evaluated with polysome profiling technology. Expectedly, rapamycin induced a shift of mRNAs from polysomes towards monosomes, thus reducing maximum cellular growth rate by 30%, while feeding additional nutrients extended mTOR pathway activity during the stationary growth phase in control batch culture, thereby contributing to an increase in global translation activity by up to 2-fold, and up to 5-fold higher specific translation of the heavy and light chains of the recombinant mAb. These increases in translation activity correlated with a 5-day extension in cellular growth and a 4-fold higher final product titer observed upon nutrient feeding. This first study of the relationship between the mTOR pathway and translational activity in CHO cultures provides key insights into the role of translational control in supporting greater productivity, which will lead to further enhancement of CHO cultures.

95 ||| 22252946 ||| (1, 0.61) ||| 1 ||| 8 ||| Differential in-gel electrophoresis (DIGE) analysis of CHO cells under hyperosmotic pressure: osmoprotective effect of glycine betaine addition. The use of glycine betaine combined with hyperosmolality is known to be an efficient means for achieving high protein production in recombinant Chinese hamster ovary (rCHO) cells. In order to understand the intracellular events and identify the key factors in rCHO cells cultivated with glycine betaine under hyperosmotic conditions, two-dimensional differential in-gel electrophoresis (2D-DIGE) followed by mass spectrometric analysis was applied. Differentially expressed 19 protein spots were selected and 16 different kinds of proteins were successfully identified. The identified proteins were associated with cellular metabolism (PEPCK, GAPDH, and PK), cellular architecture (beta-tubulin and beta-actin), protein folding (GRP78 and OSP94), mRNA processing (Rbm34, ACF, and IPMK), and protein secretion (gamma-COP). 2D-Western blot analysis of beta-tubulin, GAPDH, Peroxidoxin-1, and GRP78 confirmed the proteomic findings. The proteins identified from this study, which are related to cell growth and antibody production, can be applied to cell engineering for maximizing the efficacy of the use of glycine betaine combined with hyperosmolality in rCHO cells.

96 ||| 21692195 ||| (1, 0.61) ||| 1 ||| 8 ||| Profiling highly conserved microrna expression in recombinant IgG-producing and parental Chinese hamster ovary cells. MicroRNAs (miRNAs) play important roles in global gene regulation. Researchers in recombinant protein production have proposed miRNAs as biomarkers and cell engineering targets. However, miRNA expression remains understudied in Chinese Hamster Ovary cells, one of the most commonly used host cell systems for therapeutic protein production. To profile highly conserved miRNA expression, we used the miRCURY miRNA array for screening miRNAs in CHO cells. The selection criteria for further miRNA profiling included positive hybridization signals and experimentally validated predicted regulatory targets. On the basis of screening, we selected 16 miRNAs for quantitative RT-PCR profiling. We profiled miR expression in parental CHO DG44 and CHO K1 cell lines as well as four recombinant DG44-derived CHO lines producing a recombinant human IgG. We observed that miR-221 and miR-222 were significantly downregulated in all IgG-producing cell lines when compared with parental DG44, whereas miR-125b was significantly downregulated in one IgG-producing line. In another IgG-producing line, miR-19a was significantly upregulated. miRNA expression was also profiled in two of these lines that were amplified by stepwise increase of methotrexate. In both amplified cell lines, let-7b and miR-221 were significantly downregulated. In parental CHO K1, let-7b, miR-15b, and miR-17 were significantly downregulated when compared with DG44. The results reported here are the first steps toward profiling highly conserved miRNAs and studying the clonal difference in miRNA expression in CHO cells and may shed light on using miRNAs in cell engineering.

97 ||| 21337326 ||| (1, 0.61) ||| 1 ||| 8 ||| A novel microRNA mmu-miR-466h affects apoptosis regulation in mammalian cells. This study determined the changes in microRNA (miRs) expression in mammalian Chinese hamster ovary (CHO) cells undergoing apoptosis induced by exposing the cells to nutrient-depleted media. The apoptosis onset was confirmed by reduced cell viability and Caspase-3/7 activation. Microarray comparison of known mouse and rat miRs in CHO cells exposed to fresh or depleted media revealed up-regulation of the mouse miR-297-669 cluster in CHO cells subjected to depleted media. The mmu-miR-466h was chosen for further analysis as the member of this cluster with the highest overexpression and its up-regulation in depleted media was confirmed with qRT-PCR. Since miRs suppress mRNA translation, we hypothesized that up-regulated mmu-miR-466h inhibits anti-apoptotic genes and induces apoptosis. A combination of bioinformatics and experimental tools was used to predict and verify mmu-miR-466h anti-apoptotic targets. 8708 predicted targets were obtained from miRecords database and narrowed to 38 anti-apoptotic genes with DAVID NCBI annotation tool. Several genes were selected from this anti-apoptotic subset based on nucleotide pairing complimentarity between the mmu-miR-466h seed region and 3' UTR of the target mRNAs. The qRT-PCR analysis revealed reduced mRNA levels of bcl2l2, dad1, birc6, stat5a, and smo genes in CHO cells exposed to depleted media. The inhibition of the mmu-miR-466h increased the expression levels of those genes and resulted in increased cell viability and decreased Caspase-3/7 activation. The up-regulation of mmu-miR-466h in response to nutrients depletion causes the inhibition of several anti-apoptotic genes in unison. This suggests the pro-apoptotic role of mmu-miR-466h and its capability to modulate the apoptotic pathway in mammalian cells.

98 ||| 17929327 ||| (1, 0.61) ||| 1 ||| 8 ||| Genomic and proteomic exploration of CHO and hybridoma cells under sodium butyrate treatment. Sodium butyrate has been known to increase the specific productivity of recombinant proteins in mammalian cells. In quest of physiological mechanisms leading to the increased productivity, DNA microarray and two dimensional gel electrophoresis (2DE) were used to assess the response of Chinese hamster ovary (CHO) and a mouse hybridoma cell (MAK) to butyrate treatment at the transcriptome and proteome level. The expression of the orthologous genes represented on both CHO cDNA and mouse Affymetrix microarray, as well as genes in the same ontological class were compared. Only a relatively small number of orthologs changed their expression consistently between the two cell lines, however, at a functional class level many genes involved in cell cycle and apoptosis were affected in both cell lines. Furthermore, a large number of genes involved in protein processing, secretion and redox activity were upregulated in both CHO and MAK cells. More genes showed a consistent trend of change at both transcript and protein levels than those which showed opposite trend in MAK cells. Overall the results suggested that the changes arising in the protein processing machinery may be responsible for the increased productivity upon butyrate treatment in both CHO and MAK cells.

99 ||| 15300775 ||| (1, 0.61) ||| 1 ||| 8 ||| A two-dimensional electrophoresis map of Chinese hamster ovary cell proteins based on fluorescence staining. We report on the development of a detailed two-dimensional electrophoresis map of Chinese hamster ovary (CHO) cell proteins based on fluorescence staining and tandem time-of-flight (TOF/TOF)-mass spectrometry. We observed a 71% success rate in the identification of proteins even though the CHO genome is not sequenced. The map consists of 224 protein identifications present in 274 two-dimensional gel electrophoresis (2-DE) gel spots. We have also initiated a study of the phosphoproteome using a commercially available phosphoprotein-specific fluorescent stain. Using this stain, we observe 672 phosphorylated proteins, including many proteins known to be phosphorylated, which is 36% of the proteins we visualized with a total protein stain and consistent with expectations.

100 ||| 10344277 ||| (1, 0.61) ||| 1 ||| 8 ||| A two-dimensional protein map of Chinese hamster ovary cells. Chinese hamster ovary (CHO) cells are used extensively for the expression of biopharmaceutical protein products. As part of our effort to better understand CHO cell physiology and protein expression changes caused by modified culture conditions, we have begun to map CHO cell polypeptides. A parental cell line reference map was established using two-dimensional gel electrophoresis with immobilized pH gradients (pH 3-10) in the first dimension and a linear acrylamide gradient (9-18%T) in the second dimension. The map is composed of over 1000 silver-stained protein spots. Protein identification is proceeding using a combination of immunostaining, NH2-terminal sequencing, and mass spectrometric analyses. Among the proteins so far identified are glucose-regulated protein 78 (GRP78), protein disulfide isomerase (PDI), galectin-1, and several heat-shock proteins. The goal is to generate a database which emphasizes those proteins most relevant to the use of CHO cells as a host for recombinant protein expression.

101 ||| 25042542 ||| (1, 0.60) ||| 1 ||| 8 ||| Use of a small molecule cell cycle inhibitor to control cell growth and improve specific productivity and product quality of recombinant proteins in CHO cell cultures. The continued need to improve therapeutic recombinant protein productivity has led to ongoing assessment of appropriate strategies in the biopharmaceutical industry to establish robust processes with optimized critical variables, that is, viable cell density (VCD) and specific productivity (product per cell, qP). Even though high VCD is a positive factor for titer, uncontrolled proliferation beyond a certain cell mass is also undesirable. To enable efficient process development to achieve consistent and predictable growth arrest while maintaining VCD, as well as improving qP, without negative impacts on product quality from clone to clone, we identified an approach that directly targets the cell cycle G1-checkpoint by selectively inhibiting the function of cyclin dependent kinases (CDK) 4/6 with a small molecule compound. Results from studies on multiple recombinant Chinese hamster ovary (CHO) cell lines demonstrate that the selective inhibitor can mediate a complete and sustained G0/G1 arrest without impacting G2/M phase. Cell proliferation is consistently and rapidly controlled in all recombinant cell lines at one concentration of this inhibitor throughout the production processes with specific productivities increased up to 110 pg/cell/day. Additionally, the product quality attributes of the mAb, with regard to high molecular weight (HMW) and glycan profile, are not negatively impacted. In fact, high mannose is decreased after treatment, which is in contrast to other established growth control methods such as reducing culture temperature. Microarray analysis showed major differences in expression of regulatory genes of the glycosylation and cell cycle signaling pathways between these different growth control methods. Overall, our observations showed that cell cycle arrest by directly targeting CDK4/6 using selective inhibitor compound can be utilized consistently and rapidly to optimize process parameters, such as cell growth, qP, and glycosylation profile in recombinant antibody production cultures.

102 ||| 24574326 ||| (1, 0.60) ||| 1 ||| 8 ||| Perfusion seed cultures improve biopharmaceutical fed-batch production capacity and product quality. Volumetric productivity and product quality are two key performance indicators for any biopharmaceutical cell culture process. In this work, we showed proof-of-concept for improving both through the use of alternating tangential flow perfusion seed cultures coupled with high-seed fed-batch production cultures. First, we optimized the perfusion N-1 stage, the seed train bioreactor stage immediately prior to the production bioreactor stage, to minimize the consumption of perfusion media for one CHO cell line and then successfully applied the optimized perfusion process to a different CHO cell line. Exponential growth was observed throughout the N-1 duration, reaching >40 x 10(6) vc/mL at the end of the perfusion N-1 stage. The cultures were subsequently split into high-seed (10 x 10(6) vc/mL) fed-batch production cultures. This strategy significantly shortened the culture duration. The high-seed fed-batch production processes for cell lines A and B reached 5 g/L titer in 12 days, while their respective low-seed processes reached the same titer in 17 days. The shortened production culture duration potentially generates a 30% increase in manufacturing capacity while yielding comparable product quality. When perfusion N-1 and high-seed fed-batch production were applied to cell line C, higher levels of the active protein were obtained, compared to the low-seed process. This, combined with correspondingly lower levels of the inactive species, can enhance the overall process yield for the active species. Using three different CHO cell lines, we showed that perfusion seed cultures can optimize capacity utilization and improve process efficiency by increasing volumetric productivity while maintaining or improving product quality.

103 ||| 21853334 ||| (1, 0.60) ||| 1 ||| 8 ||| Proteomics analysis of chinese hamster ovary cells undergoing apoptosis during prolonged cultivation. The degradation of environmental conditions, such as nutrient depletion and accumulation of toxic waste products over time, often lead to premature apoptotic cell death in mammalian cell cultures and suboptimal protein yield. Although apoptosis has been extensively researched, the changes in the whole cell proteome during prolonged cultivation, where apoptosis is a major mode of cell death, have not been examined. To our knowledge, the work presented here is the first whole cell proteome analysis of non-induced apoptosis in mammalian cells. Flow cytometry analyses of various activated caspases demonstrated the onset of apoptosis in Chinese hamster ovary cells during prolonged cultivation was primarily through the intrinsic pathway. Differential in gel electrophoresis proteomic study comparing protein samples collected during cultivation resulted in the identification of 40 differentially expressed proteins, including four cytoskeletal proteins, ten chaperone and folding proteins, seven metabolic enzymes and seven other proteins of varied functions. The induction of seven ER chaperones and foldases is a solid indication of the onset of the unfolded protein response, which is triggered by cellular and ER stresses, many of which occur during prolonged batch cultures. In addition, the upregulation of six glycolytic enzymes and another metabolic protein emphasizes that a change in the energy metabolism likely occurred as culture conditions degraded and apoptosis advanced. By identifying the intracellular changes during cultivation, this study provides a foundation for optimizing cell line-specific cultivation processes, prolonging longevity and maximizing protein production.

104 ||| 16570314 ||| (1, 0.60) ||| 1 ||| 8 ||| Transcriptional profiling of apoptotic pathways in batch and fed-batch CHO cell cultures. Chinese Hamster ovary (CHO) cells are regarded as one of the "work-horses" for complex biotherapeutics production. In these processes, loss in culture viability occurs primarily via apoptosis, a genetically controlled form of cellular suicide. Using our "in-house" developed CHO cDNA array and a mouse oligonucleotide array for time profile expression analysis of batch and fed-batch CHO cell cultures, the genetic circuitry that regulates and executes apoptosis induction were examined. During periods of high viability, most pro-apoptotic genes were down-regulated but upon loss in viability, several early pro-apoptotic signaling genes were up-regulated. At later stages of viability loss, we detected late pro-apoptotic effector genes such as caspases and DNases being up-regulated. This sequential regulation of apoptotic genes showed that DNA microarrays could be used as a tool to study apoptosis. We found that in batch and fed-batch cultures, apoptosis signaling occurred primarily via death receptor- and mitochondria-mediated signaling pathways rather than endoplasmic reticulum-mediated signaling. These insights provide a greater understanding of the regulatory circuitry of apoptosis during cell culture and allow for subsequent targeting of relevant apoptosis signaling genes to prolong cell culture.

105 ||| 25701679 ||| (1, 0.59) ||| 1 ||| 8 ||| The DNA methylation landscape of Chinese hamster ovary (CHO) DP-12 cells. Chinese hamster ovary (CHO) cells represent the most commonly used production cell line for therapeutic proteins. By recent genome and transcriptome sequencing a basis was created for future investigations of genotype-phenotype relationships and for improvement of CHO cell productivity and product quality. In this context information is missing about DNA cytosine methylation as a crucial epigenetic modification and an important element in mammalian genome regulation and development. Here, we present the first DNA methylation map of a CHO cell line in single-base resolution that was generated by whole genome bisulfite sequencing combined with gene expression analysis by CHO microarrays. We show CHO DP-12 cells to exhibit global hypomethylation compared to a majority of mammalian methylomes and hypermethylation of CpG-dense regions at gene promoters called CpG islands. We also observed partially methylated domains that cover 62% of the CHO DP-12 cell genome and contain functional clusters of genes. Gene expression analysis showed these clusters to be either highly or weakly expressed with regard to CHO-specific characteristics and hence proves DNA methylation in CHO cells to be an important link between genomics and transcriptomics.

106 ||| 24146078 ||| (1, 0.59) ||| 1 ||| 8 ||| Establishment of a CpG island microarray for analyses of genome-wide DNA methylation in Chinese hamster ovary cells. Optimizing productivity and growth rates of recombinant Chinese hamster ovary (CHO) cells requires insight into the regulation of cellular processes. In this regard, the elucidation of the epigenetic process of DNA methylation, known to influence transcription by a differential occurrence in CpG islands in promoter regions, is increasingly gaining importance. However, DNA methylation has not yet been investigated on a genomic scale in CHO cells and suitable tools have not existed until now. Based on the genomic and transcriptomic CHO data currently available, we developed a customized oligonucleotide microarray covering 19598 CpG islands (89 % of total bioinformatically identified CpG islands) in the CHO genome. We applied our CHO-specific CpG island microarray to investigate the effect of butyrate treatment on differential DNA methylation in CHO cultures in a time-dependent approach. Supplementation of butyrate is known to enhance cell specific productivities in CHO cells and leads to alterations of epigenetic silencing events. Gene ontology clusters regarding, e.g., chromatin modification or DNA repair, were significantly overrepresented 24 h after butyrate addition. Functional classifications furthermore indicated that several major signaling systems such as the Wnt/beta-catenin pathway were affected by butyrate treatment. Our novel CHO-specific CpG island microarray will provide valuable information in future studies of cellular processes associated with productivity and product characteristics.

107 ||| 19306069 ||| (1, 0.59) ||| 1 ||| 8 ||| Automation of cell line development. An automated platform for development of high producing cell lines for biopharmaceutical production has been established in order to increase throughput and reduce development costs. The concept is based on the Cello robotic system (The Automation Partnership) and covers screening for colonies and expansion of static cultures. In this study, the glutamine synthetase expression system (Lonza Biologics) for production of therapeutic monoclonal antibodies in Chinese hamster ovary cells was used for evaluation of the automation approach. It is shown that the automated procedure is capable of producing cell lines of equal quality to the traditionally generated cell lines in terms of colony detection following transfection and distribution of IgG titer in the screening steps. In a generic fed-batch evaluation in stirred tank bioreactors, IgG titers of 4.7 and 5.0 g/L were obtained for best expressing cell lines. We have estimated that the number of completed cell line development projects can be increased up to three times using the automated process without increasing manual workload, compared to the manual process. Correlation between IgG titers obtained in early screens and titers achieved in fed-batch cultures in shake flasks was found to be poor. This further implies the benefits of utilizing a high throughput system capable of screening and expanding a high number of transfectants. Two concentrations, 56 and 75 muM, of selection agent, methionine sulphoximine (MSX), were applied to evaluate the impact on the number of colonies obtained post transfection. When applying selection medium containing 75 muM MSX, fewer low producing transfectants were obtained, compared to cell lines selected with 56 muM MSX, but an equal number of high producing cell lines were found. By using the higher MSX concentration, the number of cell line development projects run in parallel could be increased and thereby increasing the overall capacity of the automated platform process.

108 ||| 9608886 ||| (1, 0.58) ||| 1 ||| 8 ||| Innovative and economic potential of mammalian cell culture. Innovations for economic optimization of manufacturing processes of mammalian cell culture processes address new expression systems, optimized cell culture media and feeding systems, economy of scale, efficient harvest systems for viable cell separation, redesign of downstream processing and reduction of the overall number of quality control assays. A very efficient expression system in Chinese hamster ovary cells is the NEOSPLA expression system yielding 60-100 micrograms monoclonal antibodies per cell and day. Efficient supplements in nutrient feeding are insulin and amino acids which contribute to a high extent to the productivity of the mammalian cell culture process. Large scale manufacturing processes lower cost of goods by reduction of turn around cost for cleaning and steaming in place, media preparation, number of batches for annual campaign, in-process and quality control. In downstream processing the number of process steps and the step yield are responsible for the economics. Process control systems in a computer assisted manufacturing plant increase success rate, reduces man power and minimizes shifts. In the innovative process also alternative technologies such as transgenic animals should be considered to improve the economy of the manufacturing processes.

109 ||| 24827782 ||| (1, 0.58) ||| 1 ||| 8 ||| Accelerating genome editing in CHO cells using CRISPR Cas9 and CRISPy, a web-based target finding tool. Chinese hamster ovary (CHO) cells are widely used in the biopharmaceutical industry as a host for the production of complex pharmaceutical proteins. Thus genome engineering of CHO cells for improved product quality and yield is of great interest. Here, we demonstrate for the first time the efficacy of the CRISPR Cas9 technology in CHO cells by generating site-specific gene disruptions in COSMC and FUT8, both of which encode proteins involved in glycosylation. The tested single guide RNAs (sgRNAs) created an indel frequency up to 47.3% in COSMC, while an indel frequency up to 99.7% in FUT8 was achieved by applying lectin selection. All eight sgRNAs examined in this study resulted in relatively high indel frequencies, demonstrating that the Cas9 system is a robust and efficient genome-editing methodology in CHO cells. Deep sequencing revealed that 85% of the indels created by Cas9 resulted in frameshift mutations at the target sites, with a strong preference for single base indels. Finally, we have developed a user-friendly bioinformatics tool, named "CRISPy" for rapid identification of sgRNA target sequences in the CHO-K1 genome. The CRISPy tool identified 1,970,449 CRISPR targets divided into 27,553 genes and lists the number of off-target sites in the genome. In conclusion, the proven functionality of Cas9 to edit CHO genomes combined with our CRISPy database have the potential to accelerate genome editing and synthetic biology efforts in CHO cells.

110 ||| 23919117 ||| (1, 0.58) ||| 1 ||| 8 ||| The Role of Different Supplements in Expression Level of Monoclonal Antibody against Human CD20. BACKGROUND: Recombinant monoclonal antibodies have been marketed in last three decades as the major therapeutic proteins against different cancers. However choosing a proper medium and supplements to reach the high expression is a challenging step. Despite of commercial serum free and chemically defined media, there are still numerous researches seeking the optimum media to gain higher expression titer. Selecting the best basal media followed by proper supplementation to increase the cell density and expression titer needs proper and accurate investigation. METHODS: In this study, we have determined the expression titer of monoclonal antibody against human CD20 using soy extract, Essential Amino Acid, Non-Essential Amino Acid, Panexin NTS, Peptone, Yeast extract, Insulin-transferrin selenite, Human Serum Albumin, Bovine Serum Albumin, Lipid, and two commercially available supplements, Power and Xtreme feed. In each experiment, the expression level was compared with a well defined media, ProCHO5, RPMI 1640 and DMEM-F12. RESULTS: It has been shown that supplementing the ProCHO5 basal medium with 10% power feed or combination of 5% PanexinNTS,1.5 g/L yeast and 1.5g/L peptone results in the best production levels with 450 and 425 mg/L of anti CD20 mAb expression level, respectively. CONCLUSION: Panexin NTS, yeast and peptone cane be proper supplement for fed-batch cell culture instead of commercial Power feed supplement which is a cost effective way to increase expression level. And thereby ProCHO5 may be replaced with common media such as RPMI 1640 and DMEM-F12.

111 ||| 23140450 ||| (1, 0.58) ||| 1 ||| 8 ||| Identifying the CHO secretome using mucin-type O-linked glycosylation and click-chemistry. Chinese hamster ovary cells (CHO) are the most common cell line used in the production of therapeutic proteins. Understanding the complex pattern of secreted host cell proteins (HCP) that are released by CHO cells will facilitate the development of new recombinant protein production processes. In this study, we have adapted the N-azido-galactosamine (GalNAz) metabolic labeling method to enable the mass spectrometry identification and quantification of secreted proteins in cell culture media. CHO DG44 and CHO-S cells were cultured in media containing GalNAz, which was metabolically incorporated into mucin-type O-linked glycans of secreted proteins. These proteins were effectively enriched using click-chemistry from the cell culture media, allowing for the analysis of secreted proteins across multiple days of cell growth. When compared to the standard method for secretome analysis, the GalNAz method not only increased the total number of proteins identified but dramatically improved the quality of data by decreasing the number of background proteins (cytosolic or nuclear) to essentially zero.

112 ||| 22331235 ||| (1, 0.58) ||| 1 ||| 8 ||| Effects of high passage cultivation on CHO cells: a global analysis. Cell lines for industrial pharmaceutical protein production processes need to be robust, fast-growing, and high-producing. In order to find such cells, we performed a high passage cultivation of monoclonal antibody producing Chinese hamster ovary (CHO) cells in shaking flasks for more than 420 days. Examinations of cell growth, productivity, intracellular protein, and metabolite characteristics as well as product transcript and genomic integrate levels revealed substantial differences between subpopulations that were cryopreserved from long-term cultivation at different time points. Detected growth performance as well as intracellular adenylate energy charge increased during high passage cultivation. In addition, proteome analysis indicated an augmented utilization of glycolysis with higher passage number and an enhanced robustness based on anti-stress proteins. Interestingly, the product formation increased at first but decreased dramatically during the later subcultivations, although selection pressure was applied. Utilizing flow cytometry and quantitative real-time polymerase chain reaction, we further examined the translational, transcriptional, and genomic basis for the observed phenotypes. The detected reduction of antibody expression, in particular of the heavy chain, was ascribed to a decrease of antibody transcript, caused by loss of gene copy number and assumably a malfunctioning splicing mechanism of the dicistronic mRNA. To our knowledge, this is the first systematic approach using process analytics and targeted omic techniques to elucidate the effects of long-term cultivation of CHO cells expressing a therapeutic protein.

113 ||| 19003409 ||| (1, 0.58) ||| 1 ||| 8 ||| Engineering Chinese hamster ovary (CHO) cells to achieve an inverse growth - associated production of a foreign protein, beta-galactosidase. Protein synthesis in mammalian cells can be observed in two strikingly different patterns: 1) production of monoclonal antibodies in hybridoma cultures is typically inverse growth associated and 2) production of most therapeutic glycoproteins in recombinant mammalian cell cultures is found to be growth associated. Production of monoclonal antibodies has been easily maximized by culturing hybridoma cells at very low growth rates in high cell density fed- batch or perfusion bioreactors. Applying the same bioreactor techniques to recombinant mammalian cell cultures results in drastically reduced production rates due to their growth associated production kinetics. Optimization of such growth associated production requires high cell growth conditions, such as in repeated batch cultures or chemostat cultures with attendant excess biomass synthesis. Our recent research has demonstrated that this growth associated production in recombinant Chinese hamster ovary (CHO) cells is related to the S (DNA synthesis)-phase specific production due to the SV40 early promoter commonly used for driving the foreign gene expression. Using the stably transfected CHO cell lines synthesizing an intracellular reporter protein under the control of SV40 early promoter, we have recently demonstrated in batch and continuous cultures that the product synthesis is growth associated. We have now replaced this S-phase specific promoter in new expression vectors with the adenovirus major late promoter which was found to be active primarily in the G1-phase and is expected to yield the desirable inverse growth associated production behavior. Our results in repeated batch cultures show that the protein synthesis kinetics in this resulting CHO cell line is indeed inverse growth associated. Results from continuous and high cell density perfusion culture experiments also indicate a strong inverse growth associated protein synthesis. The bioreactor optimization with this desirable inverse growth associated production behavior would be much simpler than bioreactor operation for cells with growth associated production.

114 ||| 25529344 ||| (1, 0.57) ||| 1 ||| 8 ||| Determination of the maximum operating range of hydrodynamic stress in mammalian cell culture. Application of quality by design (QbD) requires identification of the maximum operating range for parameters affecting the cell culture process. These include hydrodynamic stress, mass transfer or gradients in dissolved oxygen and pH. Since most of these are affected by the impeller design and speed, the main goal of this work was to identify a maximum operating range for hydrodynamic stress, where no variation of cell growth, productivity and product quality can be ensured. Two scale-down models were developed operating under laminar and turbulent condition, generating repetitive oscillating hydrodynamic stress with maximum stress values ranging from 0.4 to 420Pa, to compare the effect of the different flow regimes on the cells behavior. Two manufacturing cell lines (CHO and Sp2/0) used for the synthesis of therapeutic proteins were employed in this study. For both cell lines multiple process outputs were used to determine the threshold values of hydrodynamic stress, such as cell growth, morphology, metabolism and productivity. They were found to be different in between the cell lines with values equal to 32.4+-4.4Pa and 25.2+-2.4Pa for CHO and Sp2/0, respectively. Below the measured thresholds both cell lines do not show any appreciable effect of the hydrodynamic stress on any critical quality attribute, while above, cells responded negatively to the elevated stress. To confirm the applicability of the proposed method, the obtained results were compared with data generated from classical small-scale reactors with a working volume of 3L.

115 ||| 24261865 ||| (1, 0.57) ||| 1 ||| 8 ||| MC3: a steady-state model and constraint consistency checker for biochemical networks. BACKGROUND: Stoichiometric models provide a structural framework for analyzing steady-state cellular behavior. Models are developed either through augmentations of existing models or more recently through automatic reconstruction tools. There is currently no standardized practice or method for validating the properties of a model before placing it in the public domain. Considerable effort is often required to understand a model's inconsistencies before its reuse within new research efforts. RESULTS: We present a review of common issues in stoichiometric models typically uncovered during pathway analysis and constraint-based optimization, and we detail succinct and efficient ways to find them. We present MC3, Model and Constraint Consistency Checker, a computational tool that can be used for two purposes: (a) identifying potential connectivity and topological issues for a given stoichiometric matrix, S, and (b) flagging issues that arise during constraint-based optimization. The MC3 tool includes three distinct checking components. The first examines the results of computing the basis for the null space for Sv = 0; the second uses connectivity analysis; and the third utilizes Flux Variability Analysis. MC3 takes as input a stoichiometric matrix and flux constraints, and generates a report summarizing issues. CONCLUSIONS: We report the results of applying MC3 to published models for several systems including Escherichia coli, an adipocyte cell, a Chinese Hamster Ovary cell, and Leishmania major. Several issues with no prior documentation are identified. MC3 provides a standalone MATLAB-based comprehensive tool for model validation, a task currently performed either ad hoc or implemented in part within other computational tools.

116 ||| 22002953 ||| (1, 0.57) ||| 1 ||| 8 ||| Controlled release of nutrients to mammalian cells cultured in shake flasks. Though cell culture-based protein production processes are rarely carried out under batch mode of operation, cell line and initial process development operations are usually carried out in batch mode due to simplicity of operation in widely used scale down platforms like shake flasks. Nutrient feeding, if performed, is achieved by bolus addition of concentrated feed solution at different intervals, which leads to large transient increases in nutrient concentrations. One negative consequence is increased waste metabolite production. We have developed a hydrogel-based nutrient delivery system for continuous feeding of nutrients in scale down models like shake flasks without the need for manual feed additions or any additional infrastructure. Continuous delivery also enables maintaining nutrient concentrations at low levels, if desired. The authors demonstrate the use of these systems for continuous feeding of glucose and protein hydrolysate to a suspension Chinese Hamster Ovary (CHO) culture in a shake flask. Glucose feeding achieved using the glucose-loaded hydrogel resulted in a 23% higher integral viable cell density and an 89% lower lactate concentration at the end of the culture when compared with a bolus-feed of glucose.

117 ||| 21769861 ||| (1, 0.57) ||| 1 ||| 8 ||| Metabolite profiling of recombinant CHO cells: designing tailored feeding regimes that enhance recombinant antibody production. Chinese hamster ovary (CHO) cells are the primary platform for commercial expression of recombinant therapeutic proteins. Obtaining maximum production from the expression platform requires optimal cell culture medium (and associated nutrient feeds). We have used metabolite profiling to define the balance of intracellular and extracellular metabolites during the production process of a CHO cell line expressing a recombinant IgG4 antibody. Using this metabolite profiling approach, it was possible to identify nutrient limitations, which acted as bottlenecks for antibody production, and subsequently develop a simple feeding regime to relieve these metabolic bottlenecks. This metabolite profiling-based strategy was used to design a targeted, low cost nutrient feed that increased cell biomass by 35% and doubled the antibody titer. This approach, with the potential for utilization in non-specialized laboratories, can be applied universally to the optimization of production of commercially important biopharmaceuticals.

118 ||| 18240028 ||| (1, 0.57) ||| 1 ||| 8 ||| Transcriptional profiling of gene expression changes in a PACE-transfected CHO DUKX cell line secreting high levels of rhBMP-2. Chinese hamster ovary (CHO) cells are widely used in the biopharmaceutical industry for the production of recombinant human proteins including complex polypeptides such as recombinant human bone morphogenic protein 2 (rhBMP-2). Large-scale manufacture of rhBMP-2 has associated production difficulties resulting from incomplete processing of the recombinant human protein due to insufficient endogenous levels of the paired basic amino acid cleaving enzyme (PACE) in CHO. In order to resolve this issue, CHO DUKX cells expressing rhBMP-2 were transfected with the soluble version of human PACE (PACEsol) resulting in improved amino-terminal homogeneity and a fourfold increase in rhBMP-2 productivity. In this article, we present a microarray expression profile analysis comparing the parental lineage to the higher producing subclone co-expressing PACEsol using a proprietary CHO-specific microarray. Using this technology we observed 1,076 significantly different genes in the high-productivity cells co-expressing PACEsol. Following further analysis of the differentially expressed genes, the Unfolded Protein Response (UPR) component of the endoplasmic reticulum stress response pathway was identified as a key candidate for effecting increased productivity in this cell system. Several additional ER- and Golgi-localised proteins were identified which may also contribute to this effect. The results presented here support the use of large-scale microarray expression profiling as a viable and valuable route towards understanding the behaviour of bioprocess cultures in vitro.

119 ||| 24148184 ||| (1, 0.56) ||| 1 ||| 8 ||| Monitoring CHO cell cultures: cell stress and early apoptosis assessment by mass spectrometry. Mammalian cells, especially CHO (Chinese Hamster Ovary), are an important host for the production of biopharmaceuticals. Early detection of cellular stress and the onset of apoptosis, ultimately leading to a reduced viability of the culture, are important with respect to process development and monitoring. In this work, intact cell MALDI mass spectrometry (ICM MS) biotyping was used to rapidly and sensitively detect cell stress and the onset of apoptosis at line in CHO cell cultures. We describe the identification of specific and highly reproducible stress and apoptosis related changes in m/z signal intensities that allowed prediction of upcoming cell viability changes approximately 24h earlier than standard culture monitoring. Furthermore, early identification of apoptosis onset was comparable to that using a sensitive, albeit offline, detection method. By comparison with ICM MS analysis of apoptosis induced cultures, many of the m/z values were identified as apoptosis-specific. A classification model for discrimination of unknown samples regarding their cellular viability/apoptosis status was developed based on a condensed set of 51 m/z values. The fast, robust and automated acquisition of cell state specific MS signatures could become a promising tool for CHO culture monitoring.

120 ||| 23142953 ||| (1, 0.56) ||| 1 ||| 8 ||| CHO glycosylation mutants as potential host cells to produce therapeutic proteins with enhanced efficacy. CHO glycosylation mutants, pioneered by Stanley and co-workers, have proven to be valuable tools in glycobiology and biopharmaceutical research. Here we aim to provide a summary of our efforts to isolate industrially applicable CHO glycosylation mutants, termed CHO-gmt cells, using cytotoxic lectins and zinc-finger nuclease technology. The genetic defects in the glycosylation machinery in these cells lead to the production of recombinant glycoproteins with consistent and unique glycan structures. In addition, these mutant cells can be easily adapted to serum-free medium in suspension cultures, the condition used by the biotech industry for large-scale production of recombinant therapeutics. In light of the critical impact of glycosylation on biopharmaceutical performances, namely, safety and efficacy, the CHO-gmt lines have enormous potential in producing glycoprotein therapeutics with optimal glycosylation profiles, thus, representing a panel of ideal host cell lines for producing recombinant biopharmaceuticals with improved safety profiles and enhanced efficacy.

121 ||| 23074084 ||| (1, 0.56) ||| 1 ||| 8 ||| Impact of aeration strategy on CHO cell performance during antibody production. Stirred tank bioreactors using suspension adapted mammalian cells are typically used for the production of complex therapeutic proteins. The hydrodynamic conditions experienced by cells within this environment have been shown to directly impact growth, productivity, and product quality and therefore an improved understanding of the cellular response is critical. Here we investigate the sub-lethal effects of different aeration strategies on Chinese hamster ovary cells during monoclonal antibody production. Two gas delivery systems were employed to study the presence and absence of the air-liquid interface: bubbled direct gas sparging and a non-bubbled diffusive silicone membrane system. Additionally, the effect of higher gas flow rate in the sparged bioreactor was examined. Both aeration systems were run using chemically defined media with and without the shear protectant Pluronic F-68 (PF-68). Cells were unable to grow with direct gas sparging without PF-68; however, when a silicone membrane aeration system was implemented growth was comparable to the sparged bioreactor with PF-68, indicating the necessity of shear protectants in the presence of bubbles. The cultures exposed to increased hydrodynamic stress were shown by flow cytometry to have decreased F-actin intensity within the cytoskeleton and enter apoptosis earlier. This indicates that these conditions elicit a sub-lethal physiological change in cells that would not be detected by the at-line assays which are normally implemented during cell culture. These physiological changes only result in a difference in continuous centrifugation performance under high flow rate conditions. Product quality was more strongly affected by culture age than the hydrodynamic conditions tested.

122 ||| 22974585 ||| (1, 0.56) ||| 1 ||| 8 ||| Multivariate analysis of cell culture bioprocess data--lactate consumption as process indicator. Multivariate analysis of cell culture bioprocess data has the potential of unveiling hidden process characteristics and providing new insights into factors affecting process performance. This study investigated the time-series data of 134 process parameters acquired throughout the inoculum train and the production bioreactors of 243 runs at the Genentech's Vacaville manufacturing facility. Two multivariate methods, kernel-based support vector regression (SVR) and partial least square regression (PLSR), were used to predict the final antibody concentration and the final lactate concentration. Both product titer and the final lactate level were shown to be predicted accurately when data from the early stages of the production scale were employed. Using only process data from the inoculum train, the prediction accuracy of the final process outcome was lower; the results nevertheless suggested that the history of the culture may exert significant influence on the final process outcome. The parameters contributing most significantly to the prediction accuracy were related to lactate metabolism and cell viability in both the production scale and the inoculum train. Lactate consumption, which occurred rather independently of the residual glucose and lactate concentrations, was shown to be a prominent factor in determining the final outcome of production-scale cultures. The results suggest possible opportunities to intervene in metabolism, steering it towards the type with a strong propensity towards high productivity. Such intervention could occur in the inoculum stage or in the early stage of the production-scale reactors. Overall, this study presents pattern recognition as an important process analytical technology (PAT). Furthermore, the high correlation between lactate consumption and high productivity can provide a guide to apply quality by design (QbD) principles to enhance process robustness.

123 ||| 20571937 ||| (1, 0.56) ||| 1 ||| 8 ||| Simultaneous targeting of Requiem & Alg-2 in Chinese hamster ovary cells for improved recombinant protein production. Apoptosis is known to be the main cause of cell death in the bioreactor environment, leading to the loss of recombinant protein productivity. In a previous study, transcriptional profiling was used to identify and target four early apoptosis-signaling genes: FADD, FAIM, Alg-2, and Requiem. The resulting cell lines had increased viable cell numbers and extended culture viability, which translated to increased protein productivity. Combinatorial targeting of two genes simultaneously has previously been shown to be more effective than targeting one gene alone. In this study, we sought to determine if targeting Requiem and Alg-2 was more effective than targeting Requiem alone. We found that targeting Requiem and Alg-2 did not result in extended culture viability, but resulted in an increase in maximum viable cell numbers and cumulative IVCD under fed-batch conditions. This in turn led to an approximately 1.5-fold increase in recombinant protein productivity.

124 ||| 25740626 ||| (1, 0.55) ||| 1 ||| 8 ||| Heat shock protein 27 overexpression in CHO cells modulates apoptosis pathways and delays activation of caspases to improve recombinant monoclonal antibody titre in fed-batch bioreactors. CHO cells are major production hosts for recombinant biologics including the rapidly expanding recombinant monoclonal antibodies (mAbs). Heat shock protein 27 (HSP27) expression was observed to be down-regulated towards the late-exponential and stationary phase of CHO fed-batch bioreactor cultures, whereas HSP27 was found to be highly expressed in human pathological cells and reported to have anti-apoptotic functions. These phenotypes suggest that overexpression of HSP27 is a potential cell line engineering strategy for improving robustness of CHO cells. In this work, HSP27 was stably overexpressed in CHO cells producing recombinant mAb and the effects of HSP27 on cell growth, volumetric production titer and product quality were assessed. Concomitantly, HSP27 anti-apoptosis functions in CHO cells were investigated. Stably transfected clones cultured in fed-batch bioreactors displayed 2.2-fold higher peak viable cell density, delayed loss of culture viability by two days and 2.3-fold increase in mAb titer without affecting the N-glycosylation profile, as compared to clones stably transfected with the vector backbone. Co-immunoprecipitation studies revealed HSP27 interactions with Akt, pro-caspase 3 and Daxx and caspase activity profiling showed delayed increase in caspase 2, 3, 8 and 9 activities. These results suggest that HSP27 modulates apoptosis signaling pathways and delays caspase activities to improve performance of CHO fed-batch bioreactor cultures.

125 ||| 21945585 ||| (1, 0.55) ||| 1 ||| 8 ||| Unraveling the Chinese hamster ovary cell line transcriptome by next-generation sequencing. The pyrosequencing technology from 454 Life Sciences and a novel assembly approach for cDNA sequences with the Newbler Assembler were used to achieve a major step forward to unravel the transcriptome of Chinese hamster ovary (CHO) cells. Normalized cDNA libraries originating from several cell lines and diverse culture conditions were sequenced and the resulting 1.84 million reads were assembled into 32,801 contiguous sequences, 29,184 isotigs, and 24,576 isogroups. A taxonomic classification of the isotigs showed that more than 70% of the assembled data is most similar to the transcriptome of Mus musculus, with most of the remaining isotigs being homologous to DNA sequences from Rattus norvegicus. Mapping of the CHO cell line contigs to the mouse transcriptome demonstrated that 9124 mouse transcripts, representing 6701 genes, are covered by more than 95% of their sequence length. Metabolic pathways of the central carbohydrate metabolism and biosynthesis routes of sugars used for protein N-glycosylation were reconstructed from the transcriptome data. All relevant genes representing major steps in the N-glycosylation pathway of CHO cells were detected. The present manuscript represents a data set of assembled and annotated genes for CHO cells that can now be used for a detailed analysis of the molecular functioning of CHO cell lines.

126 ||| 21595052 ||| (1, 0.55) ||| 1 ||| 8 ||| Cell culture and gene transcription effects of copper sulfate on Chinese hamster ovary cells. This study reports the effects of varying concentrations of copper sulfate on the metabolic and gene transcriptional profile of a recombinant Chinese hamster ovary (CHO) cell line producing an immunoglobulin G (IgG)-fusion protein (B0). Addition of 50 muM copper sulfate significantly decreased lactate accumulation in the cultures while increasing viable cell density and protein titer. These changes could be seen from day 6 and became increasingly evident with culture duration. Reducing the copper sulfate concentration to 5 muM retained all the above beneficial effects, but with the added benefit of reduced levels of the aggregated form of the B0 protein. To profile the cellular changes due to copper sulfate addition at the transcriptional level, Affymetrix CHO microarrays were used to identify differentially expressed genes related to reduced cellular stresses and facilitated cell cycling. Based on the microarray results, down-regulation of the transferrin receptor and lactate dehydrogenase, and up-regulation of a cytochrome P450 family-2 polypeptide were then confirmed by Western blotting. These results showed that copper played a critical role in cell metabolism and productivity on recombinant CHO cells and highlighted the usefulness of microarray data for better understanding biological responses on medium modification.

127 ||| 18778741 ||| (1, 0.55) ||| 1 ||| 8 ||| E2F-1 overexpression increases viable cell density in batch cultures of Chinese hamster ovary cells. The cell density is an inherent constraint in commercial mammalian cell cultures. Here, we describe a cell engineering strategy utilizing the overexpression of the E2F-1 cell cycle transcription factor in CHO DG44 cells that produce a monoclonal antibody in serum-free, suspension culture. Stable pools and cell lines expressing E2F-1 were isolated that attained viable cell densities 20% higher than control cell lines and continued proliferation for an additional day in batch culture. There were no significant changes in antibody production, apoptosis, and cell cycle compared to control cells, nor were the growth effects evident in fed-batch conditions. Overall, E2F-1 overexpression postponed entry into stationary phase in mammalian cells, but perhaps novel E2F-1 variants or combination cell cycle engineering strategies will be necessary to realize significant growth benefits in commercial applications.

128 ||| 18351681 ||| (1, 0.55) ||| 1 ||| 8 ||| Identification of novel small molecule enhancers of protein production by cultured mammalian cells. Small molecule additives to cell culture media (e.g., sodium butyrate) that are capable of enhancing the expression of recombinant proteins have significant utility in the production and manufacture of therapeutic polypeptides. To identify novel small molecule enhancers (SMEs) of recombinant protein expression in Chinese Hamster Ovary (CHO) cells, we screened two separate small molecule libraries for compounds capable of enhancing the expression of either a fluorescent reporter protein or a monoclonal antibody. Several compounds that increased recombinant protein expression were identified, and these compounds fell into three broad classes: (1) aromatic carboxylic acids, (2) hydroxamic acids, and (3) acetamides. We examined the impact of SME addition to CHO cell cultures expressing different classes of recombinant proteins including monoclonal antibodies (MAbs). For CHO cell pools or clones grown in production shake-flasks or bioreactors, recombinant protein titers up to 60% higher than control cultures were observed. Analysis of mRNA levels suggest that transcriptional activation plays a role in the expression enhancement seen for some SMEs, but other mechanisms may be involved for at least one compound. Finally, we tested many of the identified SMEs for their ability to increase MAb production by a hybridoma cell line. Hexanohydroxamic acid increased shake-flask MAb production by 40% relative to a control. Taken together, these data demonstrate the potential utility of the compounds in the production of therapeutically relevant proteins from diverse cell-based production systems.

129 ||| 24692260 ||| (1, 0.54) ||| 1 ||| 8 ||| Elucidating the effects of postinduction glutamine feeding on the growth and productivity of CHO cells. Inducible mammalian expression systems are increasingly being used for the production of valuable therapeutics. In such system, maximizing the product yield is achieved by carefully balancing the biomass concentration during the production phase and the specific productivity of the cells. These two factors are largely determined by the availability of nutrients and/or the presence of toxic waste metabolites in the culture environment. Glutamine is one of the most important components of cell culture medium, since this substrate is an important building block and source of energy for biomass and recombinant protein production. Its metabolism, however, ultimately leads to the formation of ammonia, a well known inhibitor of cellular growth and productivity. In this work, we show that nutrient feeding post-induction can greatly enhance the product yield by alleviating early limitations encountered in batch. Moreover, varying the amount of glutamine in the feed yielded two distinct culture behaviors post-induction; whereas excess glutamine allowed to reach greater cell concentrations, glutamine-limited fed-batch led to increased cell specific productivity. These two conditions also showed distinctive lactate metabolism. To further assess the physiological impact of glutamine levels on the cells, a comparative (13) C-metabolic flux analysis was conducted and a number of key intracellular fluxes were found to be affected by the amount of glutamine present in the feed during the production phase. Such information may provide useful clues for the identification of physiological markers of cell growth and productivity that could further guide the optimization of inducible expression systems.

130 ||| 24056080 ||| (1, 0.54) ||| 1 ||| 8 ||| Comparative analysis of amino acid metabolism and transport in CHO variants with different levels of productivity. Chinese hamster ovary (CHO) cells are widely used for the production of biopharmaceuticals; however, our understanding of several physiological elements that contribute to productivity is limited. One of these is amino acid transport and how its limitation and/or regulation might affect productivity. To further our understanding, we have examined the expression of 40 mammalian amino acid transporter genes during batch cultures of three CHO cell lines: a non-producer and two antibody-producing cell lines with different levels of productivity. In parallel, extracellular and intracellular levels of amino acids were quantified. The aim was to identify differences in gene regulation between cell lines and within culture. Our results show that three transporters associated with transport of taurine and beta-alanine, acidic amino acids and branched chain amino acids, are highly upregulated in both antibody-producing cell lines but not in the non-producer. Additionally, genes associated with the transport of amino acids related to the glutathione pathway (alanine, cysteine, cystine, glycine, glutamate) were found to be highly upregulated during the stationary phase of cell culture, correlating well with literature data on the importance of the pathway. Our analysis highlights potential markers for cell line selection and targets for process optimization.

131 ||| 23296898 ||| (1, 0.54) ||| 1 ||| 8 ||| Metabolic analysis of antibody producing CHO cells in fed-batch production. Chinese hamster ovary (CHO) cells are commonly used for industrial production of recombinant proteins in fed batch or alternative production systems. Cells progress through multiple metabolic stages during fed-batch antibody (mAb) production, including an exponential growth phase accompanied by lactate production, a low growth, or stationary phase when specific mAb production increases, and a decline when cell viability declines. Although media composition and cell lineage have been shown to impact growth and productivity, little is known about the metabolic changes at a molecular level. Better understanding of cellular metabolism will aid in identifying targets for genetic and metabolic engineering to optimize bioprocess and cell engineering. We studied a high expressing recombinant CHO cell line, designated high performer (HP), in fed-batch productions using stable isotope tracers and biochemical methods to determine changes in central metabolism that accompany growth and mAb production. We also compared and contrasted results from HP to a high lactate producing cell line that exhibits poor growth and productivity, designated low performer (LP), to determine intrinsic metabolic profiles linked to their respective phenotypes. Our results reveal alternative metabolic and regulatory pathways for lactate and TCA metabolite production to those reported in the literature. The distribution of key media components into glycolysis, TCA cycle, lactate production, and biosynthetic pathways was shown to shift dramatically between exponential growth and stationary (production) phases. We determined that glutamine is both utilized more efficiently than glucose for anaplerotic replenishment and contributes more significantly to lactate production during the exponential phase. Cells shifted to glucose utilization in the TCA cycle as growth rate decreased. The magnitude of this metabolic switch is important for attaining high viable cell mass and antibody titers. We also found that phosphoenolpyruvate carboxykinase (PEPCK1) and pyruvate kinase (PK) are subject to differential regulation during exponential and stationary phases. The concomitant shifts in enzyme expression and metabolite utilization profiles shed light on the regulatory links between cell metabolism, media metabolites, and cell growth.

132 ||| 20391528 ||| (1, 0.54) ||| 1 ||| 8 ||| Protein reference mapping of dihydrofolate reductase-deficient CHO DG44 cell lines using 2-dimensional electrophoresis. Chinese hamster ovary (CHO) cells are the major mammalian host for producing various therapeutic proteins. Among CHO cells, the dihydrofolate reductase-deficient CHO DG44 cell line has been used as a popular mammalian host because of the availability of a well-characterized genetic selection and amplification system. However, this cell line has not been studied at the proteome level. Here, the first detailed proteome analysis of the CHO DG44 cell line is described. A protein reference map of the CHO DG44 cell line was established by analyzing whole cellular proteins using 2-DE with various immobilized pH gradients (pHs 3-10, 5-8, and 3-6) in the first dimension and a 12% acrylamide gel in the second dimension. The map is composed of over 1400 silver-stained protein spots. Among them, 179 protein spots, which represent proteins associated with various biological processes and cellular compartments, were identified based on MALDI-TOF-MS and MS/MS. This proteome database should be valuable for better understanding of CHO cell physiology and protein expression patterns which may lead to efficient therapeutic protein production.

133 ||| 18727128 ||| (1, 0.54) ||| 1 ||| 8 ||| Enhancement of transient gene expression and culture viability using Chinese hamster ovary cells overexpressing Bcl-x(L). Transient gene expression (TGE) provides a method for quickly delivering protein for research using mammalian cells. While high levels of recombinant proteins have been produced in TGE experiments in HEK 293 cells, TGE efforts in the commercially prominent CHO cell line still suffer from inadequate protein yields. Here, we describe a cell-engineering strategy to improve transient production of proteins using CHO cells. CHO-DG44 cells were engineered to overexpress the anti-apoptotic protein Bcl-x(L) and transiently transfected using polyethylenimine (PEI) in serum-free media. Pools and cell lines stably expressing Bcl-x(L) showed enhanced viable cell density and increased production of a glycosylated, therapeutic fusion protein in shake flask TGE studies. The improved cell lines showed fusion protein production levels ranging from 12.6 to 27.0 mg/L in the supernatant compared to the control cultures which produced 6.3-7.3 mg/L, representing a 70-270% increase in yield after 14 days of fed-batch culture. All Bcl-xL-expressing cell lines also exhibited an increase in specific productivity during the first 8 days of culture. In addition to increased production, Bcl-x(L) cell lines maintained viabilities above 90% and less apoptosis compared to the DG44 host which had viabilities below 60% after 14 days. Product quality was comparable between a Bcl-xL-engineered cell line and the CHO host. The work presented here provides the foundation for using anti-apoptosis engineered CHO cell lines for increased production of therapeutic proteins in TGE applications.

134 ||| 25612871 ||| (1, 0.53) ||| 1 ||| 8 ||| 2D-DIGE screening of high-productive CHO cells under glucose limitation--basic changes in the proteome equipment and hints for epigenetic effects. CHO derivates (Chinese hamster ovary) belong to the most important mammalian cells for industrial recombinant protein production. Many efforts have been made to improve productivity and stability of CHO cells in bioreactor processes. Here, we followed up one barely understood phenomenon observed with process optimizations: a significantly increased cell-specific productivity in late phases of glucose-limited perfusion cultivations, when glucose (and lactate) reserves are exhausted. Our aim was to elucidate the cellular activities connected to the metabolic shift from glucose surplus to glucose limitation phase. With 2D-DIGE, we compared three stages in a perfusion culture of CHO cells: the initial growth with high glucose concentration and low lactate production, the second phase with glucose going to limitation and high lactate level, and finally the state of glucose limitation and also low lactate concentration but increased cell-specific productivity. With our proteomic approach we were able to demonstrate consequences of glucose limitation for the protein expression machinery which also could play a role for a higher recombinant protein production. Most interestingly, we detected epigenetic effects on the level of proteins involved in histone modification (HDAC1/-2, SET, RBBP7, DDX5). Together with shifts in the protein inventory of energy metabolism, cytoskeleton and protein expression, a picture emerges of basic changes in the cellular equipment under long-term glucose limitation of CHO cells.

135 ||| 25369564 ||| (1, 0.51) ||| 1 ||| 8 ||| Synchronized mammalian cell culture: part II--population ensemble modeling and analysis for development of reproducible processes. The consideration of inherent population inhomogeneities of mammalian cell cultures becomes increasingly important for systems biology study and for developing more stable and efficient processes. However, variations of cellular properties belonging to different sub-populations and their potential effects on cellular physiology and kinetics of culture productivity under bioproduction conditions have not yet been much in the focus of research. Culture heterogeneity is strongly determined by the advance of the cell cycle. The assignment of cell-cycle specific cellular variations to large-scale process conditions can be optimally determined based on the combination of (partially) synchronized cultivation under otherwise physiological conditions and subsequent population-resolved model adaptation. The first step has been achieved using the physical selection method of countercurrent flow centrifugal elutriation, recently established in our group for different mammalian cell lines which is presented in Part I of this paper series. In this second part, we demonstrate the successful adaptation and application of a cell-cycle dependent population balance ensemble model to describe and understand synchronized bioreactor cultivations performed with two model mammalian cell lines, AGE1.HNAAT and CHO-K1. Numerical adaptation of the model to experimental data allows for detection of phase-specific parameters and for determination of significant variations between different phases and different cell lines. It shows that special care must be taken with regard to the sampling frequency in such oscillation cultures to minimize phase shift (jitter) artifacts. Based on predictions of long-term oscillation behavior of a culture depending on its start conditions, optimal elutriation setup trade-offs between high cell yields and high synchronization efficiency are proposed.

136 ||| 24975601 ||| (1, 0.51) ||| 1 ||| 8 ||| Recent advances in the understanding of biological implications and modulation methodologies of monoclonal antibody N-linked high mannose glycans. With the prevalence of therapeutic monoclonal antibodies (mAbs) in the biopharmaceutical industry, the use of mammalian cell culture systems, particularly Chinese hamster ovary (CHO) cells, has become the main method for the production of therapeutics. Despite their similarity to human cells, one major challenge of mammalian cell based biopharmaceutical production is controlling aberrant glycosylation, especially glycans with five to nine mannose residues-high mannose glycoforms. Glycosylation plays a critical role in determining the therapeutic profile of therapeutic glycoproteins; high mannose glycoforms in particular have been shown to have a significant impact on clinical efficacy and pharmacokinetics. Thus, producing glycoform profiles with consistent levels of high mannose is necessary to reduce batch-to-batch therapeutic variability and to meet regulatory standards. Studies have shown that high mannose glycoforms can be modulated through the genetic engineering of cell lines, addition of inhibitors to key enzymes in the glycosylation pathways, and varying cell culture conditions. Focusing on these three types of techniques, this review will examine and critically assess current methods for high mannose glycosylation control and future developments in this area.

137 ||| 24819042 ||| (1, 0.51) ||| 1 ||| 8 ||| Overexpression of microRNAs enhances recombinant protein production in Chinese hamster ovary cells. MicroRNAs (miRNAs) are short, non-coding RNAs that can negatively regulate expression of multiple genes at post-transcriptional levels. Using miRNAs to target multiple genes and pathways is a promising cell-engineering strategy to increase recombinant protein production in mammalian cells. Here, we identified miRs-17, -19b, -20a, and -92a to be differentially expressed between high- and low- monoclonal antibody-producing Chinese hamster ovary (CHO) cell clones using next-generation sequencing and quantitative real-time PCR. These miRNAs were stably overexpressed individually and in combination in a high-producing clone to assess their effects on CHO cell growth, recombinant protein productivity and product quality. Stably transfected pools demonstrated 24-34% increases in specific productivity (qP) and 21-31% increases in titer relative to the parental clone, without significant alterations in proliferation rates. The highest protein-producing clones isolated from these pools exhibited 130-140% increases in qP and titer compared to the parental clone, without major changes in product aggregation and N-glycosylation profile. From our clonal data, correlations between enhanced qP/titer and increased levels of miRs-17, -19b, and -92a were observed. Our results demonstrate the potential of miRs-17, -19b, and -92a as cell-engineering targets to increase recombinant protein production in mammalian cells.

138 ||| 23636936 ||| (1, 0.51) ||| 1 ||| 8 ||| Differential response in downstream processing of CHO cells grown under mild hypothermic conditions. The manufacture of complex therapeutic proteins using mammalian cells is well established, with several strategies developed to improve productivity. The application of sustained mild hypothermic conditions during culture has been associated with increases in product titer and improved product quality. However, despite associated cell physiological effects, very few studies have investigated the impact on downstream processing (DSP). Characterization of cells grown under mild hypothermic conditions demonstrated that the stationary phase was prolonged by delaying the onset of apoptosis. This enabled cells to maintain viability for extended periods and increase volumetric productivity from 0.74 to 1.02 g L(-1) . However, host cell proteins, measured by ELISA, increased by ~50%, attributed to the extended time course and higher peak and harvest cell densities. The individual components making up this impurity, as determined by SELDI-TOF MS and 2D-PAGE, were shown to be largely comparable. Under mild hypothermic conditions, cells were less shear sensitive than those maintained at 37 C, enhancing the preliminary primary recovery step. Adaptive changes in membrane fluidity were further investigated by adopting a pronounced temperature shift immediately prior to primary recovery and the improvement observed suggests that such a strategy may be implementable when shear sensitivity is of concern. Early and late apoptotic cells were particularly susceptible to shear, at either temperature, even under the lowest shear rate investigated. These findings demonstrate the importance of considering the impact of cell culture strategies and cell physiology on DSP, by implementing a range of experimental methods for process characterization.

139 ||| 22068567 ||| (1, 0.51) ||| 1 ||| 8 ||| Improving the efficiency of CHO cell line generation using glutamine synthetase gene knockout cells. Although Chinese hamster ovary (CHO) cells, with their unique characteristics, have become a major workhorse for the manufacture of therapeutic recombinant proteins, one of the major challenges in CHO cell line generation (CLG) is how to efficiently identify those rare, high-producing clones among a large population of low- and non-productive clones. It is not unusual that several hundred individual clones need to be screened for the identification of a commercial clonal cell line with acceptable productivity and growth profile making the cell line appropriate for commercial application. This inefficiency makes the process of CLG both time consuming and laborious. Currently, there are two main CHO expression systems, dihydrofolate reductase (DHFR)-based methotrexate (MTX) selection and glutamine synthetase (GS)-based methionine sulfoximine (MSX) selection, that have been in wide industrial use. Since selection of recombinant cell lines in the GS-CHO system is based on the balance between the expression of the GS gene introduced by the expression plasmid and the addition of the GS inhibitor, L-MSX, the expression of GS from the endogenous GS gene in parental CHOK1SV cells will likely interfere with the selection process. To study endogenous GS expression's potential impact on selection efficiency, GS-knockout CHOK1SV cell lines were generated using the zinc finger nuclease (ZFN) technology designed to specifically target the endogenous CHO GS gene. The high efficiency (~2%) of bi-allelic modification on the CHO GS gene supports the unique advantages of the ZFN technology, especially in CHO cells. GS enzyme function disruption was confirmed by the observation of glutamine-dependent growth of all GS-knockout cell lines. Full evaluation of the GS-knockout cell lines in a standard industrial cell culture process was performed. Bulk culture productivity improved two- to three-fold through the use of GS-knockout cells as parent cells. The selection stringency was significantly increased, as indicated by the large reduction of non-producing and low-producing cells after 25 microM L-MSX selection, and resulted in a six-fold efficiency improvement in identifying similar numbers of high-productive cell lines for a given recombinant monoclonal antibody. The potential impact of GS-knockout cells on recombinant protein quality is also discussed.

140 ||| 20945491 ||| (1, 0.51) ||| 1 ||| 8 ||| Combining high-throughput screening of caspase activity with anti-apoptosis genes for development of robust CHO production cell lines. A set of anti-apoptotic genes were over-expressed, either singly or in combination, in an effort to develop robust Chinese Hamster Ovary host cell lines suitable for manufacturing biotherapeutics. High-throughput screening of caspase 3/7 activity enabled a rapid selection of transfectants with reduced caspase activity relative to the host cell line. Transfectants with reduced caspase 3/7 activity were then tested for improved integrated viable cell count (IVCC), a function of peak viable cell density and longevity. The maximal level of improvement in IVCC could be achieved by over-expression of either single anti-apoptotic genes, e.g., Bcl-2Delta (a mutated variant of Bcl-2) or Bcl-XL, or a combination of two or three anti-apoptotic genes, e.g., E1B-19K, Aven, and XIAPDelta. These cell lines yielded higher transient antibody production and a greater number of stable clones with high antibody yields. In a 5 L fed-batch bioreactor system, BDelta31-1, a stable clone expressing Bcl-2Delta, had a product titer that was 180% as compared to an optimal clone (Con-1) from the control cell line. Although lactate accumulated to more than 5 g/L in the control culture, its concentration was reduced in the anti-apoptotic BDelta31-1 cultures to below 1 g/L, confirming our earlier findings that cells over-expressing anti-apoptotic genes consume the lactate that would otherwise accumulate as a by-product in the culture medium. To the best of our knowledge, this is the first study to use the high throughput caspase screening method to identify CHO host cell lines with superior anti-apoptotic characteristics.

141 ||| 20521304 ||| (1, 0.51) ||| 1 ||| 8 ||| Profiling of N-glycosylation gene expression in CHO cell fed-batch cultures. One of the goals of recombinant glycoprotein production is to achieve consistent glycosylation. Although many studies have examined the changes in the glycosylation quality of recombinant protein with culture, very little has been done to examine the underlying changes in glycosylation gene expression as a culture progresses. In this study, the expression of 24 genes involved in N-glycosylation were examined using quantitative RT PCR to gain a better understanding of recombinant glycoprotein glycosylation during production processes. Profiling of the N-glycosylation genes as well as concurrent analysis of glycoprotein quality was performed across the exponential, stationary and death phases of a fed-batch culture of a CHO cell line producing recombinant human interferon-gamma (IFN-gamma). Of the 24 N-glycosylation genes examined, 21 showed significant up- or down-regulation of gene expression as the fed-batch culture progressed from exponential, stationary and death phase. As the fed-batch culture progressed, there was also an increase in less sialylated IFN-gamma glycoforms, leading to a 30% decrease in the molar ratio of sialic acid to recombinant IFN-gamma. This correlated with decreased expression of genes involved with CMP sialic acid synthesis coupled with increased expression of sialidases. Compared to batch culture, a low glutamine fed-batch strategy appears to need a 0.5 mM glutamine threshold to maintain similar N-glycosylation genes expression levels and to achieve comparable glycoprotein quality. This study demonstrates the use of quantitative real time PCR method to identify possible "bottlenecks" or "compromised" pathways in N-glycosylation and subsequently allow for the development of strategies to improve glycosylation quality.

142 ||| 18989903 ||| (1, 0.51) ||| 1 ||| 8 ||| Molecular engineering of exocytic vesicle traffic enhances the productivity of Chinese hamster ovary cells. A complex vesicle trafficking system manages the precise and regulated distribution of proteins, membranes and other molecular cargo between cellular compartments as well as the secretion of (heterologous) proteins in mammalian cells. Sec1/Munc18 (SM) proteins are key components of the system by regulating membrane fusion. However, it is not clear how SM proteins contribute to the overall exocytosis. Here, functional analysis of the SM protein Sly1 and Munc18c suggested a united, positive impact upon SNARE-based fusion of ER-to-Golgi- and Golgi-to-plasma membrane-addressed exocytic vesicles and increased the secretory capacity of different therapeutic proteins in Chinese hamster ovary cells up to 40 pg/cell/day. Sly1- and Munc18c-based vesicle traffic engineering cooperated with Xbp-1-mediated ER/Golgi organelle engineering. Our study supports a model for united function of SM proteins in stimulating vesicle trafficking machinery and provides a generic secretion engineering strategy to improve biopharmaceutical manufacturing of important protein therapeutics.

143 ||| 16937407 ||| (1, 0.51) ||| 1 ||| 8 ||| A novel function for selenium in biological system: selenite as a highly effective iron carrier for Chinese hamster ovary cell growth and monoclonal antibody production. As the market for biopharmaceuticals especially monoclonal antibodies (MAbs) rapidly grows, their manufacturing methods are coming under increasing regulatory scrutiny, particularly due to concerns about the potential introduction of adventitious agents from animal-sourced components in the media used for their production in mammalian cell culture. Chinese hamster ovary (CHO) cells are by far the most commonly used production vehicles for these recombinant glycoproteins. In developing animal-component free media for CHO and other mammalian cell lines, the iron-transporter function of serum or human/bovine transferrin is usually replaced by certain organic or inorganic chelators capable of delivering iron for cell respiration and metabolism, but few of them are sufficiently effective. Selenium is a well-known essential trace element (TE) for cell growth and development, and its positive role in biological system includes detoxification of free radicals by activating glutathione peroxidase. In cell culture, selenium in the form of selenite can help cells to detoxify the medium thus protect them from oxidative damage. In this presentation, we describe the discovery and application of a novel function of selenite, that is, as a highly effective carrier to deliver iron for cell growth and function. In our in-house-developed animal protein-free (APF) medium for CHO cells, using an iron-selenite compound to replace the well-established tropolone delivery system for iron led to comparable or better cell growth and antibody production. A high cell density of >10 x 10(6) viable cells/mL and excellent antibody titer of approximately 3 g/L were achieved in 14-day fed-batch cultures in shake flasks, followed by successful scale-up to stirred bioreactors. The preparation of the commercially unavailable iron-selenite compound from respective ions, and its effectiveness in cell-culture performance, were dependent on reaction time, substrates, and other conditions.

144 ||| 16860584 ||| (1, 0.51) ||| 1 ||| 8 ||| RNAi suppression of Bax and Bak enhances viability in fed-batch cultures of CHO cells. Bcl-2 family proteins play a crucial role in the regulation of the mitochondrial pathway that leads to apoptosis. Members of the Bcl-2 family can be divided into the anti-apoptotic proteins such as Bcl-2 and Bcl-X(L), and the pro-apoptotic proteins such as Bax and Bak and the BH3-only proteins. In this study, siRNA constructs to silence the Bax and Bak genes in Chinese hamster ovary (CHO) cells were generated. Stable CHO cell lines in which the expression of Bax and Bak were significantly knocked down were screened by Western blot analysis and confirmed by RT-PCR. CHO cells with both Bax and Bak knocked down showed a clear resistance against cytotoxic lectins and UV irradiation-induced apoptosis. Compared to original CHO-K1 cells, these cells also survived longer when cultured under extreme conditions such as complete nutrient depletion or in high-osmolality medium. CHO cells with both Bax and Bak genes knocked down displayed an extended lifespan as well as higher viable cell densities in fed-batch cultures, both in adherent form on microcarrier beads and in suspension. The IFN-gamma productivity by a rCHO IFN-gamma cell line in which both Bak and Bax were knocked down increased by 35% compared to the control cells. These results indicate that the genetic inactivation of Bax and Bak in recombinant CHO cells can be an effective strategy in delaying the onset of apoptosis in batch and fed-batch cultures.

145 ||| 16599582 ||| (1, 0.51) ||| 1 ||| 8 ||| Application of fractional factorial designs to screen active factors for antibody production by chinese hamster ovary cells. With ever increasing need for cost-effective large-scale production of monoclonal antibodies, it is essential to develop highly productive and commercially viable processes. Previous research showed that growth and production capacity of the culture media can be improved by micronutrient supplements, such as insulin, vitamins, and growth factors. Since these micronutrients may not act independently of one another, factorial designs can expose critical interactions between nutrients as opposed to a serial approach of changing one factor at a time. In this study, fractional factorial designs were applied to observe the effect of several micronutrients on antibody production and culture longevity in shake flasks. Response surface designs were used to investigate the factors in depth and confirm the results of the fractional factorial study. The results demonstrate that fractional factorial design is an effective tool for rapid development of antibody-producing Chinese hamster ovary (CHO) cells.

146 ||| 25880925 ||| (1, 0.50) ||| 1 ||| 8 ||| BioPreDyn-bench: a suite of benchmark problems for dynamic modelling in systems biology. BACKGROUND: Dynamic modelling is one of the cornerstones of systems biology. Many research efforts are currently being invested in the development and exploitation of large-scale kinetic models. The associated problems of parameter estimation (model calibration) and optimal experimental design are particularly challenging. The community has already developed many methods and software packages which aim to facilitate these tasks. However, there is a lack of suitable benchmark problems which allow a fair and systematic evaluation and comparison of these contributions. RESULTS: Here we present BioPreDyn-bench, a set of challenging parameter estimation problems which aspire to serve as reference test cases in this area. This set comprises six problems including medium and large-scale kinetic models of the bacterium E. coli, baker's yeast S. cerevisiae, the vinegar fly D. melanogaster, Chinese Hamster Ovary cells, and a generic signal transduction network. The level of description includes metabolism, transcription, signal transduction, and development. For each problem we provide (i) a basic description and formulation, (ii) implementations ready-to-run in several formats, (iii) computational results obtained with specific solvers, (iv) a basic analysis and interpretation. CONCLUSIONS: This suite of benchmark problems can be readily used to evaluate and compare parameter estimation methods. Further, it can also be used to build test problems for sensitivity and identifiability analysis, model reduction and optimal experimental design methods. The suite, including codes and documentation, can be freely downloaded from the BioPreDyn-bench website, https://sites.google.com/site/biopredynbenchmarks/ .

147 ||| 24604826 ||| (1, 0.50) ||| 1 ||| 8 ||| Enhanced transient recombinant protein production in CHO cells through the co-transfection of the product gene with Bcl-xL. Transient gene expression is gaining popularity as a method to rapidly produce recombinant proteins in mammalian cells. Although significant improvements have been made, in terms of expression, more improvements are needed to compete with the yields achievable in stable gene expression. Much progress has come from optimization of transfection media and parameters, as well as altering culturing conditions to enhance productivity. Recent studies have included cell lines engineered for apoptosis resistance through the constitutive expression of an anti-apoptotic protein, Bcl-xL. In this study, we examine an alternative method of using the benefits of anti-apoptotic gene expression to enhance the transient expression of biotherapeutics, namely, through the co-transfection of Bcl-xL and the product-coding gene. CHO-S cells were co-transfected with the product-coding gene and a vector containing Bcl-xL using polyethylenimine. Cells co-transfected with Bcl-xL showed reduced levels of apoptosis, increased specific productivity, and an overall increase in product yield of approximately 100%. Similar results were produced by employing another anti-apoptotic protein, Bcl-2 delta, in CHO cells, or through the co-transfection with Bcl-xL using HEK-293E cells. This work provides an alternative method for increasing yields of therapeutic proteins in TGE applications without generating a stable cell line and subsequent screening, which are both time- and resource-consuming. See accompanying commentary by Matthias Hackl and Nicole Borth DOI: 10.1002/biot.201400104.

148 ||| 24146795 ||| (1, 0.50) ||| 1 ||| 8 ||| Using molecular markers to characterize productivity in Chinese hamster ovary cell lines. Selection of high producing cell lines to produce maximum product concentration is a challenging and time consuming task for the biopharmaceutical industry. The identification of early markers to predict high productivity will significantly reduce the time required for new cell line development. This study identifies candidate determinants of high productivity by profiling the molecular and morphological characteristics of a panel of six Chinese Hamster Ovary (CHO) stable cell lines with varying recombinant monoclonal antibody productivity levels ranging between 2 and 50 pg/cell/day. We examined the correlation between molecular parameters and specific productivity (qp ) throughout the growth phase of batch cultures. Results were statistically analyzed using Pearson correlation coefficient. Our study revealed that, overall, heavy chain (HC) mRNA had the strongest association with qp followed by light chain (LC) mRNA, HC intracellular polypeptides, and intracellular antibodies. A significant correlation was also obtained between qp and the following molecular markers: growth rate, biomass, endoplasmic reticulum, and LC polypeptides. However, in these cases, the correlation was not observed at all-time points throughout the growth phase. The repeated sampling throughout culture duration had enabled more accurate predictions of productivity in comparison to performing a single-point measurement. Since the correlation varied from day to day during batch cultivation, single-point measurement was of limited use in making a reliable prediction.

149 ||| 23749410 ||| (1, 0.50) ||| 1 ||| 8 ||| Application of a nonradioactive method of measuring protein synthesis in industrially relevant Chinese hamster ovary cells. Due to the high medical and commercial value of recombinant proteins for clinical and diagnostic purposes, the protein synthesis machinery of mammalian host cells is the subject of extensive research by the biopharmaceutical industry. RNA translation and protein synthesis are steps that may determine the extent of growth and productivity of host cells. To address the problems of utilization of current radioisotope methods with proprietary media, we have focused on the application of an alternative method of measuring protein synthesis in recombinant Chinese hamster ovary (CHO) cells. This method employs puromycin as a nonradioactive label which incorporates into nascent polypeptide chains and is detectable by western blotting. This method, which is referred to as SUnSET, successfully demonstrated the expected changes in protein synthesis in conditions that inhibit and restore translation activity and was reproducibly quantifiable. The study of the effects of feed and sodium butyrate addition on protein synthesis by SUnSET revealed an increase following 1 h feed supplementation while a high concentration of sodium butyrate was able to decrease translation during the same treatment period. Finally, SUnSET was used to compare protein synthesis activity during batch culture of the CHO cell line in relation to growth. The results indicate that as the cells approached the end of batch culture, the global rate of protein synthesis declined in parallel with the decreasing growth rate. In conclusion, this method can be used as a "snapshot" to directly monitor the effects of different culture conditions and treatments on translation in recombinant host cells.

150 ||| 23436610 ||| (1, 0.50) ||| 1 ||| 8 ||| Cooverexpression of alanine aminotransferase 1 in Chinese hamster ovary cells overexpressing taurine transporter further stimulates metabolism and enhances product yield. Innovation in monoclonal antibody (mAb) production continues to be driven by cell engineering strategies to increase yield and improve product quality. In a previous study, to investigate the effectiveness of transporter overexpression strategies, we prepared a taurine transporter-overexpressing host cell line (DXB11/TAUT) that produced a higher proportion of high-mAb-titer strains than did the parent host cell line. In the current study, we selected a single DXB11/TAUT/mAb1 strain that remained viable for longer (up to 1 month) under common fed-batch culture conditions, and the improvement in viability could be attributed to its improved metabolic properties. It was also more productive (up to >100 pg/cell/day) and yielded more mAb1 (up to 8.1 g/L/31 days) than the parent cell line, and the mAb1 it produced was of comparable quality. These results suggested that this host cell engineering strategy has unique potential for the improvement of mAb-producing Chinese hamster ovary (CHO) cells; for example, it may be appropriate for high cell density perfusion culture. TAUT-overexpressing cell lines rapidly accumulated the byproduct alanine, and our challenge in the present study was to apply a strategy for modulating cell metabolism to utilize this byproduct to achieve a high mAb yield in a shorter culture period. To accomplish this, we genetically modified the DXB11/TAUT/mAb1 strain to cooverexpress alanine aminotransferase 1 (ALT1). The resulting DXB11/TAUT/mAb1/ALT1 cooverexpressing strain gave a higher mAb yield in a shorter culture period (5.9 g/L/14 days). It is usually difficult to drive the overexpression of two functional genes while balancing competing goals. However, forced cooverexpression of TAUT and ALT1 in our DXB11/TAUT/mAb1/ALT1 strain resulted in a higher proliferation than the DXB11/TAUT/mAb1 strain, with an ideal balance between cell viability and productivity. Therefore, we have demonstrated a strategy capable of achieving an optimum balance among the goals of cell viability, productivity, and proliferative capacity.

151 ||| 22907508 ||| (1, 0.50) ||| 1 ||| 8 ||| Rational development of a serum-free medium and fed-batch process for a GS-CHO cell line expressing recombinant antibody. A serum-free medium (CHO-SFM) together with a fed-batch process was developed for the cultivation of a recombinant GS-CHO cell line producing TNFR-Fc. According to the metabolic characteristics of GS-CHO cell, a basal medium was prepared by supplementing DMEM:F12:RPMI1640 (2:1:1) with amino acids, insulin, transferrin, Pluronic F68 and some other ingredients. Statistical optimization approaches based on Plackett-Burman and central composite designs were then adopted to identify additional positive determinants and determine their optimal concentrations, which resulted in the final CHO-SFM medium formulations. The maximum antibody titer reached was 90.95 mg/l in the developed CHO-SFM, which was a 18 % and 10 fold higher than that observed in the commercial EX-CELL 302 medium (76.95 mg/l) and basal medium (8.28 mg/l), respectively. Subsequently, a reliable, reproducible and robust fed-batch strategy was designed according to the offline measurement of glucose, giving a final antibody yield of 378 mg/l, which was a threefold improvement over that in conventional batch culture (122 mg/l) using CHO-SFM. In conclusion, the use of design of experiment (DoE) method facilitated the development of CHO-SFM medium and fed-batch process for the production of recombinant antibody using GS-CHO cells.

152 ||| 21404259 ||| (1, 0.50) ||| 1 ||| 8 ||| Ectopic expression of human mTOR increases viability, robustness, cell size, proliferation, and antibody production of chinese hamster ovary cells. Engineering of mammalian production cell lines to improve titer and quality of biopharmaceuticals is a top priority of the biopharmaceutical manufacturing industry providing protein therapeutics to patients worldwide. While many engineering strategies have been successful in the past decade they were often based on the over-expression of a single transgene and therefore limited to addressing a single bottleneck in the cell's production capacity. We provide evidence that ectopic expression of the global metabolic sensor and processing protein mammalian target of rapamycin (mTOR), simultaneously improves key bioprocess-relevant characteristics of Chinese hamster ovary (CHO) cell-derived production cell lines such as cell growth (increased cell size and protein content), proliferation (increased cell-cycle progression), viability (decreased apoptosis), robustness (decreased sensitivity to sub-optimal growth factor and oxygen supplies) and specific productivity of secreted human glycoproteins. Cultivation of mTOR-transgenic CHO-derived cell lines engineered for secretion of a therapeutic IgG resulted in antibody titers of up to 50 pg/cell/day, which represents a four-fold increase compared to the parental production cell line. mTOR-based engineering of mammalian production cell lines may therefore have a promising future in biopharmaceutical manufacturing of human therapeutic proteins.

153 ||| 16003777 ||| (1, 0.50) ||| 1 ||| 8 ||| EST sequencing for gene discovery in Chinese hamster ovary cells. Chinese hamster ovary (CHO) cells are one of the most important cell lines in biological research, and are the most widely used host for industrial production of recombinant therapeutic proteins. Despite their extensive applications, little sequence information is available for molecular based research. To facilitate gene discovery and genetic engineering, two cDNA libraries were constructed from three CHO cell lines grown under various conditions. The average insert size for both libraries is approximately 800-850 bp, and each library has comparable redundancy levels of 36%-38% for the sequences isolated. Random sequencing of 4,608 ESTs yielded 2,602 unique assemblies, 76% of which were annotated as orthologs of sequences in the GenBank database. A high abundance of mitochondrial genome transcripts facilitated the assembly of the complete mitochondrial genome by PCR walking. Comparative analysis of sequences from both mitochondrial and nuclear genomes with orthologous genes from other species shows that CHO sequences are generally most similar to mouse; however, examples with highest similarity to rat or human are common. A cDNA microarray, including all 4,608 ESTs, was constructed. The microarray results reveal a high level of consistency between transcript abundance in the libraries and fluorescence intensities. Inclusion of redundant clones in the microarray, additionally, allows small changes in abundant mRNAs to be discerned with a high degree of confidence. The information and tools generated provide access to genomic technology for this important cell line.

154 ||| 12892482 ||| (1, 0.50) ||| 1 ||| 8 ||| Effect of shear stress on intrinsic CHO culture state and glycosylation of recombinant tissue-type plasminogen activator protein. Shear stress in suspension culture was investigated as a possible manipulative parameter for the control of glycosylation of the recombinant tissue-type plasminogen activator protein (r-tPA) produced by recombinant Chinese hamster ovary (CHO) cell culture, grown in protein-free media. Resulting fractions of partially glycosylated, Type II, and fully glycosylated, Type I, r-tPA protein were monitored as a direct function of the shear characteristics of the culture environment. The shear-induced response of CHO culture to levels of low shear stress, where exponential growth was not obtained, and to higher levels of shear stress, which resulted in extensive cell death, were examined through manipulation of the bioreactor stirring velocity. Both apparent and intrinsic cell growth, metabolite consumption, byproduct and r-tPA production, and r-tPA glycosylation, from a variable site-occupancy standpoint, were monitored throughout. Kinetic analyses revealed a shear-stress-induced alteration of cellular homeostasis resulting in a nonlinear dependency of metabolic yield coefficients and an intrinsic cell lysis kinetic constant on shear stress. Damaging levels of shear stress were used to investigate the shear dependence of cell death and lysis, as well as the effects on the intrinsic growth rate of the culture. Kinetic models were also developed on the basis of the intrinsic state of the culture and compared to traditional models. Total r-tPA production was maximized under moderate shear conditions, as was the viable CHO cell density of the culture. However, Type II r-tPA production and the fraction of Type II glycoform production ratio was maximized under damaging levels of shear stress. Analyses of biomass production yield coefficients coupled with a plug-flow reactor model of glycan addition in the endoplasmic reticulum (ER) were used to propose an overall mechanism of decreased r-tPA protein site-occupancy glycosylation with increasing shear stress. Decreased residence time of r-tPA in the ER as a result of increased protein synthesis related to shear protection mechanisms is proposed to limit contact of site Asn184 with the membrane-bound oligosaccharyltransferase enzyme in the ER.

155 ||| 10662492 ||| (1, 0.49),(5, 0.31) ||| 1 ||| 8 ||| Improvement of CHO cell culture medium formulation: simultaneous substitution of glucose and glutamine. The formulation of the culture medium for a Chinese hamster ovary (CHO) cell line has been investigated in terms of the simultaneous replacement of glucose and glutamine, the most commonly employed carbon and nitrogen sources, pursuing the objective of achieving a more efficient use of these compounds, simultaneously avoiding the accumulation of lactate and ammonium in the medium. The key factor in this process is the selection of compounds that are slowly metabolized. Among the different compounds studied, galactose and glutamate provide the best results, allowing support of cell growth with an optimal balance between nutrient uptake and cell requirements and the generation of minimal quantities of lactate and ammonium. The attained results also highlight the capacity of the cells to redistribute their metabolism as a response to the changes in medium composition.

156 ||| 9212190 ||| (1, 0.49) ||| 1 ||| 8 ||| Cell growth on cordierite: an approach to the identification of reliable supports for continuous-flow solid-bed reactors. This study aimed to assess the biocompatibility of two cordierite ceramics (DF and Cord 1014), with similar chemical composition and different porosity, as a potential support for cell growth in a continuous-flow, solid-bed reactor. The Chinese hamster ovary (CHO) cell line transfected with HBV-DHFR recombinant plasmid was seeded on cordierite or polystyrene dishes and evaluated for cell growth and production of recombinant hepatitis B surface antigen. Proliferation of the CHO cells, in terms of cell number, was generally similar in polystyrene and Cord 1014 and always lower in DF. Flow cytometric analysis showed no difference in cell cycle distribution for cells grown on different supports, and showed a two-fold increase in percentage of debris for cells grown on DF than for those grown on Cord 1014 and polystyrene culture dishes. Moreover, the morphology of cells grown on Cord 1014 did not change during the experiment, and cells were well spread and organized. Finally, total recombinant hepatitis B surface antigen production was higher on Cord 1014 than on polystyrene and DF samples. Such evidence suggests that Cord 1014 could be a promising support for growing cells in a continuous-flow, solid-bed reactor.

157 ||| 24039059 ||| (1, 0.49) ||| 1 ||| 8 ||| Recovery of Chinese hamster ovary host cell proteins for proteomic analysis. Identification and characterization of Chinese hamster ovary (CHO) host cell protein (HCP) impurities by proteomic techniques can aid bioprocess design and lead to more efficient development and improved biopharmaceutical manufacturing operations. Recovery of extracellular CHO HCP for proteomic analysis is particularly challenging due to the relatively low protein concentration and complex composition of media. In this article, we report the development of optimized protocols that improve proteome capture for CHO HCP. Eleven precipitation protocols were screened for protein recovery and optimized for a subset of precipitants by a design of experiments (DOE) approach. Because total protein recovery does not fully replicate a proteomics experiment, or detect non-protein agents that may interfere with proteomic methods, a subset of precipitation conditions were compared by two-dimensional electrophoresis and liquid chromatography coupled with mass spectrometry, with optimized recovery shown to differ between the two proteomic methods. This work demonstrates broadly applicable methods that can be applied as initial steps to optimize sample preparation of any sample type for proteomic analysis, and presents optimized precipitation protocols for extracellular CHO HCP recovery, which can vary appreciably between gel-based and shotgun proteomic methods.

158 ||| 22727805 ||| (1, 0.48),(8, 0.41) ||| 1 ||| 8 ||| Full automation and validation of a flexible ELISA platform for host cell protein and protein A impurity detection in biopharmaceuticals. Monitoring host cell protein (HCP) and protein A impurities is important to ensure successful development of recombinant antibody drugs. Here, we report the full automation and validation of an ELISA platform on a robotic system that allows the detection of Chinese hamster ovary (CHO) HCPs and residual protein A of in-process control samples and final drug substance. The ELISA setup is designed to serve three main goals: high sample throughput, high quality of results, and sample handling flexibility. The processing of analysis requests, determination of optimal sample dilutions, and calculation of impurity content is performed automatically by a spreadsheet. Up to 48 samples in three unspiked and spiked dilutions each are processed within 24 h. The dilution of each sample is individually prepared based on the drug concentration and the expected impurity content. Adaptable dilution protocols allow the analysis of sample dilutions ranging from 1:2 to 1:2x10(7). The validity of results is assessed by automatic testing for dilutional linearity and spike recovery for each sample. This automated impurity ELISA facilitates multi-project process development, is easily adaptable to other impurity ELISA formats, and increases analytical capacity by combining flexible sample handling with high data quality.

159 ||| 25044769 ||| (1, 0.48),(8, 0.31) ||| 1 ||| 8 ||| Synchronized mammalian cell culture: part I--a physical strategy for synchronized cultivation under physiological conditions. Conventional analysis and optimization procedures of mammalian cell culture processes mostly treat the culture as a homogeneous population. Hence, the focus is on cell physiology and metabolism, cell line development, and process control strategy. Impact on cultivations caused by potential variations in cellular properties between different subpopulations, however, has not yet been evaluated systematically. One main cause for the formation of such subpopulations is the progress of all cells through the cell cycle. The interaction of potential cell cycle specific variations in the cell behavior with large-scale process conditions can be optimally determined by means of (partially) synchronized cultivations, with subsequent population resolved model analysis. Therefore, it is desirable to synchronize a culture with minimal perturbation, which is possible with different yield and quality using physical selection methods, but not with frequently used chemical or whole-culture methods. Conventional nonsynchronizing methods with subsequent cell-specific, for example, flow cytometric analysis, can only resolve cell-limited effects of the cell cycle. In this work, we demonstrate countercurrent-flow centrifugal elutriation as a useful physical method to enrich mammalian cell populations within different phases of a cell cycle, which can be further cultivated for synchronized growth in bioreactors under physiological conditions. The presented combined approach contrasts with other physical selection methods especially with respect to the achievable yield, which makes it suitable for bioreactor scale cultivations. As shown with two industrial cell lines (CHO-K1 and human AGE1.HN), synchronous inocula can be obtained with overall synchrony degrees of up to 82% in the G1 phase, 53% in the S phase and 60% in the G2/M phase, with enrichment factors (Ysync) of 1.71, 1.79, and 4.24 respectively. Cells are able to grow with synchrony in bioreactors over several cell cycles. This strategy, combined with population-resolved model analysis and parameter extraction as described in the accompanying paper, offers new possibilities for studies of cell lines and processes at levels of cell cycle and population under physiological conditions.

160 ||| 24254056 ||| (1, 0.48) ||| 1 ||| 8 ||| Cellular responses to individual amino-acid depletion in antibody-expressing and parental CHO cell lines. Depletion of two nonessential amino acids, asparagine (Asn) and glutamine (Gln), occurred during a fed-batch production process with a CHO cell line expressing a recombinant antibody. This depletion coincided with growth suppression and the onset of the stationary phase. Experimental withdrawal of Asn led to cell cycle arrest of cell line A in G0/G1 phase. On a mechanistic level, withdrawal of either Asn or Gln stimulated the amino-acid response (AAR) pathway, indicating that depletion of nonessential amino acids can induce AAR in this cell line. Compared to withdrawal of an essential amino acid, leucine (Leu), withdrawal of either Asn or Gln induced fewer changes in downstream effectors of mammalian target of rapamycin (mTOR) signaling involved in regulation of global protein synthesis. Global transcriptional analysis followed by pathway analysis revealed that the cultures experienced a down-regulation of cell-cycle progression, DNA replication and nucleotide biosynthesis in an E2F-dependent manner, as well as a down-regulation of lipid metabolism in a SREBP1/2-dependent manner as a result of individual amino-acid withdrawal. Timing and magnitude of observed phenotypic and transcriptional responses to amino-acid withdrawal differed between essential (Leu) and nonessential (Asn and Gln) amino acids examined. Observed responses were similar in parental (CS9 and CHOK-1) and two other antibody-producing CHO cell lines, but the magnitude of the transcriptional response was both cell-line and amino-acid dependent. Overall, these results suggest that depletion of nonessential amino acids in cell culture plays a role in the onset of the stationary phase of production process and offer mechanistic insights into the observed growth attenuation phenotype.

161 ||| 10397813 ||| (1, 0.47),(5, 0.39) ||| 1 ||| 8 ||| Influence of low temperature on productivity, proteome and protein phosphorylation of CHO cells. Proliferation of mammalian cells can be controlled by low cultivation temperature. However, depending on cell type and expression system, varying effects of a temperature shift on heterologous protein production have been reported. Here, we characterize growth behavior and productivity of the Chinese hamster ovary (CHO) cell line XM111-10 engineered to synthesize the model-product-secreted alkaline phosphatase (SEAP). Shift of cultivation temperature from 37 degrees C to 30 degrees C caused a growth arrest mainly in the G1 phase of the cell cycle concomitant with an up to 1.7-fold increase of specific productivity. A low temperature cultivation provided 3.4 times higher overall product yield compared to a standard cultivation at 37 degrees C. The cellular and molecular mechanisms underlying the effects of low temperature on growth and productivity of mammalian cells are poorly understood. Separation of total protein extracts by two-dimensional gel electrophoresis showed altered expression levels of CHO-K1 proteins after decrease in cultivation temperature to 30 degrees C. These changes in the proteome suggest that mammalian cells respond actively to low temperature by synthesizing specific cold-inducible proteins. In addition, we provide the first evidence that the cold response of mammalian cells includes changes in postranslational protein modifications. Two CHO proteins were found to be phosphorylated at tyrosine residues following downshift of cultivation temperature to 30 degrees C. Elucidating cellular events during cold exposure is necessary for further optimization of host-cell lines and expression systems and can provide new strategies for metabolic engineering.

162 ||| 24975682 ||| (1, 0.47),(5, 0.30) ||| 1 ||| 8 ||| A framework for the systematic design of fed-batch strategies in mammalian cell culture. A methodology to calculate the required amount of amino acids (a.a.) and glucose in feeds for animal cell culture from monitoring their levels in batch experiments is presented herein. Experiments with the designed feeds on an antibody-producing Chinese hamster ovary cell line resulted in a 3-fold increase in titer compared to batch culture. Adding 40% more nutrients to the same feed further increases the yield to 3.5 higher than in batch culture. Our results show that above a certain threshold there is no linear correlation between nutrient addition and the integral of viable cell concentration. In addition, although high ammonia levels hinder cell growth, they do not appear to affect specific antibody productivity, while we hypothesize that high extracellular lactate concentration is the cause for the metabolic shift towards lactate consumption for the cell line used. Overall, the performance of the designed feeds is comparable to that of a commercial feed that was tested in parallel. Expanding this approach to more nutrients, as well as changing the ratio of certain amino acids as informed by flux balance analysis, could achieve even higher yields.

163 ||| 21345355 ||| (1, 0.46),(2, 0.36) ||| 1 ||| 8 ||| Supplementation of serum free media with HT is not sufficient to restore growth properties of DHFR-/- cells in fed-batch processes - Implications for designing novel CHO-based expression platforms. DHFR-deficient CHO cells are the most commonly used host cells in the biopharmaceutical industry and over the years, individual substrains have evolved, some have been engineered with improved properties and platform technologies have been designed around them. Unexpectedly, we have observed that different DHFR-deficient CHO cells show only poor growth in fed-batch cultures even in HT supplemented medium, whereas antibody producer cells derived from these hosts achieved least 2-3 fold higher peak cell densities. Using a set of different expression vectors, we were able to show that this impaired growth performance was not due to the selection procedure possibly favouring fast growing clones, but a direct consequence of DHFR deficiency. Re-introduction of the DHFR gene reproducibly restored the growth phenotype to the level of wild-type CHO cells or even beyond which seemed to be dose-dependent. The requirement for a functional DHFR gene to achieve optimal growth under production conditions has direct implications for cell line generation since it suggests that changing to a selection system other than DHFR would require another CHO host which - especially for transgenic CHO strains and tailor-suited process platforms - this could mean significant investments and potential changes in product quality. In these cases, DHFR engineering of the current CHO-DG44 or DuxB11-based host could be an attractive alternative.

164 ||| 21167884 ||| (1, 0.46) ||| 1 ||| 8 ||| Metabolomics-based identification of apoptosis-inducing metabolites in recombinant fed-batch CHO culture media. A liquid chromatography-mass spectrometry (LC-MS) based metabolomics platform was previously established to identify and profile extracellular metabolites in culture media of mammalian cells. This presented an opportunity to isolate novel apoptosis-inducing metabolites accumulating in the media of antibody-producing Chinese hamster ovary (CHO mAb) fed-batch bioreactor cultures. Media from triplicate cultures were collected daily for the metabolomics analysis. Concurrently, cell pellets were obtained for determination of intracellular caspase activity. Metabolite profiles from the LC-MS data were subsequently examined for their degree of correlation with the caspase activity. A panel of extracellular metabolites, the majority of which were nucleotides/nucleosides and amino acid derivatives, exhibited good (R2 > 0.8) and reproducible correlation. Some of these metabolites, such as oxidized glutathione, AMP and GMP, were later shown to induce apoptosis when introduced to fresh CHO mAb cultures. Finally, metabolic engineering targets were proposed to potentially counter the harmful effects of these metabolites.

165 ||| 25504860 ||| (1, 0.45),(8, 0.41) ||| 1 ||| 8 ||| Cross-scale predictive modeling of CHO cell culture growth and metabolites using Raman spectroscopy and multivariate analysis. Multi-component, multi-scale Raman spectroscopy modeling results from a monoclonal antibody producing CHO cell culture process including data from two development scales (3 L, 200 L) and a clinical manufacturing scale environment (2,000 L) are presented. Multivariate analysis principles are a critical component to partial least squares (PLS) modeling but can quickly turn into an overly iterative process, thus a simplified protocol is proposed for addressing necessary steps including spectral preprocessing, spectral region selection, and outlier removal to create models exclusively from cell culture process data without the inclusion of spectral data from chemically defined nutrient solutions or targeted component spiking studies. An array of single-scale and combination-scale modeling iterations were generated to evaluate technology capabilities and model scalability. Analysis of prediction errors across models suggests that glucose, lactate, and osmolality are well modeled. Model strength was confirmed via predictive validation and by examining performance similarity across single-scale and combination-scale models. Additionally, accurate predictive models were attained in most cases for viable cell density and total cell density; however, these components exhibited some scale-dependencies that hindered model quality in cross-scale predictions where only development data was used in calibration. Glutamate and ammonium models were also able to achieve accurate predictions in most cases. However, there are differences in the absolute concentration ranges of these components across the datasets of individual bioreactor scales. Thus, glutamate and ammonium PLS models were forced to extrapolate in cases where models were derived from small scale data only but used in cross-scale applications predicting against manufacturing scale batches.

166 ||| 23124589 ||| (1, 0.45),(8, 0.34) ||| 1 ||| 8 ||| A simple eccentric stirred tank mini-bioreactor: mixing characterization and mammalian cell culture experiments. In industrial practice, stirred tank bioreactors are the most common mammalian cell culture platform. However, research and screening protocols at the laboratory scale (i.e., 5-100 mL) rely primarily on Petri dishes, culture bottles, or Erlenmeyer flasks. There is a clear need for simple-easy to assemble, easy to use, easy to clean-cell culture mini-bioreactors for lab-scale and/or screening applications. Here, we study the mixing performance and culture adequacy of a 30 mL eccentric stirred tank mini-bioreactor. A detailed mixing characterization of the proposed bioreactor is presented. Laser induced fluorescence (LIF) experiments and computational fluid dynamics (CFD) computations are used to identify the operational conditions required for adequate mixing. Mammalian cell culture experiments were conducted with two different cell models. The specific growth rate and the maximum cell density of Chinese hamster ovary (CHO) cell cultures grown in the mini-bioreactor were comparable to those observed for 6-well culture plates, Erlenmeyer flasks, and 1 L fully instrumented bioreactors. Human hematopoietic stem cells were successfully expanded tenfold in suspension conditions using the eccentric mini-bioreactor system. Our results demonstrate good mixing performance and suggest the practicality and adequacy of the proposed mini-bioreactor.

167 ||| 19551877 ||| (1, 0.45),(7, 0.44) ||| 1 ||| 8 ||| Mcl-1 overexpression leads to higher viabilities and increased production of humanized monoclonal antibody in Chinese hamster ovary cells. Bioreactor stresses, including nutrient deprivation, shear stress, and byproduct accumulation can cause apoptosis, leading to lower recombinant protein yields and increased costs in downstream processing. Although cell engineering strategies utilizing the overexpression of antiapoptotic Bcl-2 family proteins such as Bcl-2 and Bcl-x(L) potently inhibit apoptosis, no studies have examined the use of the Bcl-2 family protein, Mcl-1, in commercial mammalian cell culture processes. Here, we overexpress both the wild type Mcl-1 protein and a Mcl-1 mutant protein that is not degraded by the proteasome in a serum-free Chinese hamster ovary (CHO) cell line producing a therapeutic antibody. The expression of Mcl-1 led to increased viabilities in fed-batch culture, with cell lines expressing the Mcl-1 mutant maintaining approximately 90% viability after 14 days when compared with 65% for control cells. In addition to enhanced culture viability, Mcl-1-expressing cell lines were isolated that consistently showed increases in antibody production of 20-35% when compared with control cultures. The quality of the antibody product was not affected in the Mcl-1-expressing cell lines, and Mcl-1-expressing cells exhibited 3-fold lower caspase-3 activation when compared with the control cell lines. Altogether, the expression of Mcl-1 represents a promising alternative cell engineering strategy to delay apoptosis and increase recombinant protein production in CHO cells.

168 ||| 25502369 ||| (1, 0.45),(2, 0.41) ||| 1 ||| 8 ||| A high cell density transient transfection system for therapeutic protein expression based on a CHO GS-knockout cell line: process development and product quality assessment. Transient gene expression (TGE) is a rapid method for the production of recombinant proteins in mammalian cells. While the volumetric productivity of TGE has improved significantly over the past decade, most methods involve extensive cell line engineering and plasmid vector optimization in addition to long fed batch cultures lasting up to 21 days. Our colleagues have recently reported the development of a CHO K1SV GS-KO host cell line. By creating a bi-allelic glutamine synthetase knock out of the original CHOK1SV host cell line, they were able to improve the efficiency of generating high producing stable CHO lines for drug product manufacturing. We developed a TGE method using the same CHO K1SV GS-KO host cell line without any further cell line engineering. We also refrained from performing plasmid vector engineering. Our objective was to setup a TGE process to mimic protein quality attributes obtained from stable CHO cell line. Polyethyleneimine (PEI)-mediated transfections were performed at high cell density (4 x 10(6) cells/mL) followed by immediate growth arrest at 32 C for 7 days. Optimizing DNA and PEI concentrations proved to be important. Interestingly, found the direct transfection method (where DNA and PEI were added sequentially) to be superior to the more common indirect method (where DNA and PEI are first pre-complexed). Moreover, the addition of a single feed solution and a polar solvent (N,N dimethylacetamide) significantly increased product titers. The scalability of process from 2 mL to 2 L was demonstrated using multiple proteins and multiple expression volumes. Using this simple, short, 7-day TGE process, we were able to successfully produce 54 unique proteins in a fraction of the time that would have been required to produce the respective stable CHO cell lines. The list of 54 unique proteins includes mAbs, bispecific antibodies, and Fc-fusion proteins. Antibody titers of up to 350 mg/L were achieved with the simple 7-day process. Titers were increased to 1 g/L by extending the culture to 16 days. We also present two case studies comparing product quality of material generated by transient HEK293, transient CHO K1SV GS-KO, and stable CHO K1SV KO pool. Protein from transient CHO was more representative of stable CHO protein compared to protein produced from HEK293.

169 ||| 21167223 ||| (1, 0.45) ||| 1 ||| 8 ||| Engineering CHO cell growth and recombinant protein productivity by overexpression of miR-7. The efficient production of recombinant proteins by Chinese Hamster Ovary (CHO) cells in modern bioprocesses is often augmented by the use of proliferation control strategies. The most common method is to shift the culture temperature from 37 C to 28-33 C though genetic approaches to achieving the same effect are also of interest. In this work we used qRT-PCR-based expression profiling using TLDA cards to identify miRNAs displaying differential expression 24h after temperature-shift (TS) from 37 C to 31 C. Six miRNAs were found to be significantly up-regulated (mir-219, mir-518d, mir-126, mir-30e, mir-489 and mir-345) and four down-regulated (mir-7, mir-320, mir-101 and mir-199). Furthermore, qRT-PCR analysis of miR-7 expression over a 6 day batch culture, with and without TS, demonstrated decreased expression over time in both cultures but to a significantly greater extent in cells shifted to a lower culture temperature. Unexpectedly, when miR-7 levels were increased transiently by transfection with miR-7 mimic in CHO-K1 cells, cell proliferation at 37 C was effectively blocked over a 96 h culture period. On the other hand, transient inhibition of endogenous miR-7 levels using antagonists had no impact on cell growth. The exogenous overexpression of miR-7 also resulted in increased normalised (per cell) production at 37 C, though the yield was lower than cells grown at reduced temperature. This is the first report demonstrating a functional impact of specific miRNA disregulation on CHO cell behavior in batch culture and provides some evidence of the potential which these molecules may have in terms of engineering targets in CHO production clones. Finally, we report the cloning and sequencing of the hamster-specific cgr-miR-7.

170 ||| 10397873 ||| (1, 0.44),(8, 0.41) ||| 1 ||| 8 ||| Monitoring of intracellular ribonucleotide pools is a powerful tool in the development and characterization of mammalian cell culture processes. Efficient cell culture process development for the industrial production of recombinant therapeutics is characterized by constraints which pertain to issues such as costs, competitiveness and the meeting of project timelines. These constraints require tools which can help the developer learn as much as possible as quickly as possible about the cell at hand and identify features of a particular culture which are amenable to improvement. Current on- and off-line monitoring parameters, however useful, provide only late indications (cell concentration, viability) and circumstantial evidence (lactate, ammonia, etc.) with regard to the physiologic status of cells at the time of sampling. The relative intracellular content of purine to pyrimidine nucleotide triphosphates as well as the ratio of UTP to UDP-N-acetylhexosamines have been previously described as sensitive indicators of a cell's metabolic status, growth potential, and overall physiological condition. The sensitivity of such nucleotide ratios and their usefulness in commercially relevant process development and characterization were tested at Boehringer Ingelheim Pharma KG in a large number of fermentations (>80) with a variety of culture modes, cells, and products in scales up to 10,000 litres. Monitoring of these intracellular parameters allows a timely and reliable assessment of cell state and growth potential, which is possible neither by classical cell number and viability measurements nor by a variety of fermentation data typically monitored. The view inside the cell afforded by nucleotide monitoring enables prediction of the behavior of a culture up to 2 days before any hint of physiological changes is given by cell number and viability estimation. In this paper, data relating the growth behavior of CHO and hybridoma cell lines to their nucleotide pools are shown. Two very different processes for the production of recombinant tPA in 10,000-litre bioreactors are compared and characterized with respect to their nucleotide profiles. Examples from industrial process development cases in which intracellular nucleotide information is used to advantage are also presented and discussed.

171 ||| 20544713 ||| (1, 0.44),(8, 0.38) ||| 1 ||| 8 ||| Optimization of cultivation conditions in spin tubes for Chinese hamster ovary cells producing erythropoietin and the comparison of glycosylation patterns in different cultivation vessels. This article describes the optimization of cultivation factor settings, that is the shaking rate and working volume in 50 mL spin tubes for a Chinese hamster ovary cell line expressing recombinant human alpha-erythropoietin, using a response D-optimal surface method. The main objectives of the research were, firstly, to determine a setting in which the product titer and product quality attributes in spin tubes are equivalent to those in 250 mL shake flasks in a seven day batch and, secondly, to find a setting in which the product titer is maximal. The model for product titer prediction as a function of shaking rate and working volume in the defined design space was successfully applied to the optimization of cultivation conditions in spin tubes for the tested cell line. Subsequently, validation experiments were carried out simultaneously in spin tubes, shake flasks and bench scale bioreactors to compare cell culture performance parameters such as growth, productivity and product quality attributes in the form of isoform profiles and glycan antennarity structures. The results of the experiments showed that similar cell culture performance and product quality could be achieved in spin tubes when compared to shake flasks. Additionally, bioreactor titers could be reproduced in spin tubes at high shaking rates and low working volumes, but with differing product quality. Cultivation at lower shaking rates in spin tubes and shake flasks produced a glycoprotein with a product quality slightly comparable to that from bioreactors, but with titers being only two thirds.

172 ||| 24166820 ||| (1, 0.44),(2, 0.42) ||| 1 ||| 8 ||| CHO cell culture longevity and recombinant protein yield are enhanced by depletion of miR-7 activity via sponge decoy vectors. Improving the efficiency of recombinant protein production by CHO cells is highly desirable as more complex proteins (MAbs, fusion proteins, blood/clotting factors, etc.) go into development and come onto the market. Previous reports have shown that microRNA (miRNA)-7 overexpression arrests the growth of CHO cells and that its depletion increases the proliferation of various cell types. In this study we generated stable CHO clones that overexpressed a miR-7-specific decoy transcript (sponge) downstream of a green fluorescent protein reporter gene. The miR-7 sponge efficiently diverted miR-7 away from its endogenous targets as exemplified by the increased expression of CDC7. Although the sponge effectively sequestered miR-7, it also appeared to protect the bound miRNA sequence from degradation in the cell, as exemplified by the apparent increase in mature miR-7 levels without any change in primary transcription. Phenotypically, CHO clones with sequestered miR-7 displayed improved maximum cell density (40%), significantly improved viability and an almost two-fold increase in yield of secreted protein in a fed-batch culture. These findings demonstrate that miRNA sponge transcripts could potentially be used in cell line development projects to generate producer clones that grow to higher densities and last longer in the bioreactor - thereby improving product yield.

173 ||| 23436541 ||| (1, 0.44),(2, 0.39) ||| 1 ||| 8 ||| Non-invasive UPR monitoring system and its applications in CHO production cultures. Unfolded protein response (UPR) is the primary signaling network activated in response to the accumulation of unfolded and/or misfolded protein in the endoplasmic reticulum (ER). The expression of high levels of recombinant proteins in mammalian cell cultures has been linked to the increased UPR. However, the dynamics of different UPR-mediated events and their impact on cell performance and recombinant protein secretion during production remain poorly defined. Here, we have created a non-invasive UPR-responsive, fluorescence-based reporter system to detect and quantify specific UPR-mediated transcriptional activation of different intracellular signaling pathways. We have generated stable antibody-expressing CHO clones containing this UPR responsive system and established FACS-based methods for real-time, continuous monitoring of the endogenous UPR activation in live cultures. The results showed that the UPR activation is dynamically regulated during production culture. The clones differed in their UPR patterns; both the timing and the degree of UPR-induced transcriptional activation were linked to cell performance, such as growth, and viability. In addition, the cell culture environment, such as media composition and osmolarity, significantly impacted endogenous UPR activation. Taken together, these data demonstrate a utility of this UPR monitoring system in recombinant protein production processes and the observations increase our understanding of the critical role of UPR in regulating diverse phenotypes of the cells including growth, survival and recombinant protein secretion under different culture environments and processing conditions.

174 ||| 25150618 ||| (1, 0.44) ||| 1 ||| 8 ||| Proteomics analysis of altered cellular metabolism induced by insufficient copper level. Insufficient copper level in the mammalian cell culture medium resulted in lactate accumulation while maintaining similar growth and culture viability profiles. Label-free, LC-MS/MS-based shotgun proteomics method was applied to compare the protein expression profiles obtained from the cultures exposed to suboptimal copper level to those provided with sufficient amount of copper. Under copper deficient condition, a substantial reduction of the protein levels of the multiple subunits of Complex IV, also known as cytochrome c oxidase, of the mitochondrial electron transport chain was observed for all three different Chinese Hamster Ovary (CHO) cell lines expressing therapeutic monoclonal antibodies tested. Additional proteins affected by suboptimal copper level included peroxiredoxin (PRDX) and hepatocyte-derived growth factor (HDGF), which were affected during early phase of the fed-batch production, several days prior to initiation of lactate accumulation. In contrast, proteins such as syntenin (SDCBP) and integral membrane 2C (ITM2C) showed altered expression patterns toward the end of culture duration, after lactate divergence had occurred. For all conditions tested, time was the most predominant factor facilitating the direction of global protein expression trend, with substantial number of proteins subjected to time-dependent changes in expression, independent of copper.

175 ||| 19918894 ||| (1, 0.44) ||| 1 ||| 8 ||| A proteomic approach for identifying cellular proteins interacting with erythropoietin in recombinant Chinese hamster ovary cells. Identification of the cellular proteins interacting with incompletely folded and unfolded forms of erythropoietin (EPO) in recombinant CHO (rCHO) cells leads to better insight into the possible genetic manipulation approaches for increasing EPO production. To do so, a pull-down assay was performed with dual-tagged (N-terminal GST- and C-terminal hexahistidine-tagged) EPO expressed in E. coli as bait proteins and cell lysates of rCHO cells (DG44) as prey proteins. Cellular proteins interacting with dual-tagged EPO were then resolved by two-dimensional gel electrophoresis (2DE) and identified by MALDI-TOF MS/MS. A total of 27 protein spots including glucose-regulated protein 78 (GRP78) were successfully identified. Western blot analysis of GRP78 confirmed the results of the MS analyses. Taken together, a pull-down assay followed by a proteomic approach is found to be an efficient means to identify cellular proteins interacting with foreign protein in rCHO cells.

176 ||| 18629924 ||| (1, 0.43),(6, 0.32) ||| 1 ||| 8 ||| CHO DUKX cell lineages preadapted to growth in serum-free suspension culture enable rapid development of cell culture processes for the manufacture of recombinant proteins. Using an adaptive strategy, Chinese hamster ovary (CHO) cell lines were developed that are capable of robust growth in serum-free suspension culture. These preadapted derivatives of the commonly used strain of CHO cells (CHO DUKX), termed PA-DUKX, were used for the introduction and stable expression of several heterologous human genes. A significant advantage of recombinant PA-DUKX cells was their ability to readily resume growth in serum-free suspension culture after transfection and amplification of heterologous genes. Expression of recombinant human proteins in PA-DUKX cells was quantitatively similar to that of lineages generated using conventional CHO DUKX cells. In addition, recombinant human proteins expressed by transfected PA-DUKX lineages were shown to be biochemically and structurally similar to those expressed in CHO DUKX cells, PA-DUKX host cell technology provides an opportunity for reducing the time and resources required to develop large-scale, suspension culture-based manufacturing processes employing serum-free medium. (c) 1996 John Wiley & Sons, Inc.

177 ||| 17311405 ||| (1, 0.43),(6, 0.31) ||| 1 ||| 8 ||| Improving performance of mammalian cells in fed-batch processes through "bioreactor evolution". The amount of recombinant product obtained from mammalian cells grown in a bioreactor is in part limited by achievable cell densities and the ability of cells to remain viable over extended periods of time. In an attempt to generate cell lines capable of better bioreactor performance, we subjected the DG44 Chinese Hamster Ovary (CHO) host cell line and a recombinant production cell line to an iterative process whereby cells capable of surviving the harsh conditions in the bioreactor were selected. This selective process was termed "bioreactor evolution". Following the selective process, the "evolved" host cells attained a 2-fold increase in peak cell density and a 72% increase in integral cell area. Transient transfection experiments demonstrate that the evolved cells have the same transfection efficiency and the same secretory potential as the initial cells. The "evolved" host was also found to contain a large subpopulation of cells that did not require insulin for growth. From this, a new population of growth-factor-independent cells was obtained. These improvements in host properties should prove beneficial in the expression of recombinant proteins in fed-batch processes. The selective process was also applied to a recombinant production cell line. The evolved cells from this selection exhibited a 38% increase in peak cell density, a 30% increase in integral cell area, and a 36% increase in product titer. These increases were obtained without any appreciable impact on product quality, demonstrating the usefulness of this simple approach to improve the performance of recombinant cell lines.

178 ||| 21328318 ||| (1, 0.43),(5, 0.32) ||| 1 ||| 8 ||| High-end pH-controlled delivery of glucose effectively suppresses lactate accumulation in CHO fed-batch cultures. A simple method for control of lactate accumulation in suspension cultures of Chinese hamster ovary (CHO) cells based on the culture's pH was developed. When glucose levels in culture reach a low level (generally below 1 mM) cells begin to take up lactic acid from the culture medium resulting in a rise in pH. A nutrient feeding method has been optimized which delivers a concentrated glucose solution triggered by rising pH. We have shown that this high-end pH-controlled delivery of glucose can dramatically reduce or eliminate the accumulation of lactate during the growth phase of a fed-batch CHO cell culture at both bench scale and large scale (2,500 L). This method has proven applicable to the majority of CHO cell lines producing monoclonal antibodies and other therapeutic proteins. Using this technology to enhance a 12-day fed-batch process that already incorporated very high initial cell densities and highly concentrated medium and feeds resulted in an approximate doubling of the final titers for eight cell lines. The increase in titer was due to additional cell growth and higher cell specific productivity.

179 ||| 9592397 ||| (1, 0.43),(2, 0.38) ||| 1 ||| 8 ||| Controlled proliferation by multigene metabolic engineering enhances the productivity of Chinese hamster ovary cells. The eukaryotic cell cycle is regulated by a complex network of many proteins. Effective reprogramming of this complex regulatory apparatus to achieve bioprocess goals, such as cessation of proliferation at high cell density to allow an extended period of high production, can require coordinated manipulation of multiple genes. Previous efforts to establish inducible cell-cycle arrest of Chinese hamster ovary (CHO) cells by regulated expression of the cyclin-dependent kinase inhibitor (CDI) p21 failed. By tetracycline-regulated coexpression of p21 and the differentiation factor CCAAT/enhancer-binding protein alpha (which both stabilizes and induces p21), we have achieved effective cell-cycle arrest. Production of a model heterologous protein (secreted alkaline phosphatase; SEAP) has been increased 10-15 times, on a per cell basis, relative to an isogenic control cell line. Because activation of apoptosis response is a possible complication in a proliferation-arrested culture, the survival gene bcl-xL was coexpressed with another CDI, p27, found to enable CHO cell-cycle arrest predominantly in G1 phase. CHO cells stably transfected with a tricistronic construct containing the genes for these proteins and for SEAP showed 30-fold higher SEAP expression than controls.

180 ||| 23180219 ||| (1, 0.42),(7, 0.40) ||| 1 ||| 8 ||| Dual role of dextran sulfate 5000 Da as anti-apoptotic and pro-autophagy agent. Dextran sulfate 5,000 Da (DS), a sulfated polysaccharide, has been used in recombinant mammalian cell cultures to prevent cell aggregation, thereby increasing cell viability. Previous studies using Chinese hamster ovary (CHO) suspension cultures had shown that low concentrations of DS are related to an inhibition of apoptosis. In this study, DS was used on anchorage-dependent CHO cells producing erythropoietin (EPO), in order to investigate the effect of this molecule on anti-apoptotic and pro-survival cellular pathways. DS 5,000 Da treatment was shown to prolong the life of cells and increase productivity of EPO by 1.8-fold comparing with controls, in standard batch conditions. At a molecular level, we show that DS inhibits apoptosis by DNA fragmentation delay and decrease of annexin V-labeled cells, causes a G0/G1 cell cycle arrest, decreases p53 expression and increases the pro-survival factor Hsc70 expression. DS treatment also resulted in an enhanced LC3-I to LC3-II conversion and increased autophagosomes formation employing tagged-LC3. Our data show, for the first time, that low doses of DS may promote autophagy in different cell lines. These findings suggest that a better understanding and manipulation of phenomenon of autophagy could be of crucial importance in the bio-pharmaceutical industry, in particular in the field of protein production.

181 ||| 17172663 ||| (1, 0.42),(2, 0.34) ||| 1 ||| 8 ||| Development of transfection and high-producer screening protocols for the CHOK1SV cell system. To date, the FDA has approved 18 monoclonal antibody (MAb) therapeutic drugs with targets ranging from asthma and rheumatoid arthritis to leukemia. Many of these approved products are produced in Chinese hamster ovary cells (CHO) making CHO a significant and relevant host system. We studied the applicability of CHOK1SV cells as a potential host cell line for MAb production in terms of timelines, achievable titers, transfectant stability, and reproducibility. CHOK1SV, developed by Lonza Biologics, is a suspension, protein-free-adapted CHOK1-derivative utilizing the glutamine synthetase (GS) gene expression system. CHOK1SV expresses the GS enzyme endogenously; thus, positive transfectants were obtained under the dual selection of methionine sulfoximine (MSX) and glutamine-free media. We examined outgrowth efficiencies, specific productivities, and achievable batch titers of three different IgG MAbs transfected into CHOK1SV. Reducing the MSX concentration in the initial selection medium resulted in a decreased incubation time required for transfectant colonies to appear. Specific productivities of "high-producers" ranged between 11 and 49 pg/c/d with batch titers ranging from 105 to 519 mg/L. Transfectant stability and the effects of MSX also were investigated, which indicated that the addition of MSX was necessary to maintain stable MAb production. Cell growth was stable regardless of MSX concentration.

182 ||| 22140034 ||| (1, 0.42) ||| 1 ||| 8 ||| A mechanistic study on the effect of dexamethasone in moderating cell death in Chinese Hamster Ovary cell cultures. Dexamethasone (DEX) was previously shown (Jing et al., Biotechnol Bioeng. 2010;107:488-496) to play a dual role in increasing sialylation of recombinant glycoproteins produced by Chinese Hamster Ovary (CHO) cells. DEX addition increased sialic acid levels of a recombinant fusion protein through increased expression of alpha2,3-sialyltransferase and beta1,4-galactosyltransferase, but also decreased the sialidase-mediated, extracellular degradation of sialic acid through slowing cell death at the end of the culture period. This study examines the underlying mechanism for this cytoprotective action by studying the transcriptional response of the CHO cell genome upon DEX treatment using DNA microarrays and gene ontology term analysis. Many of those genes showing a significant transcriptional response were associated with the regulation of programmed cell death. The gene with the highest change in expression level, as validated by Quantitative PCR assays with TaqMan probes and confirmed by Western Blot analysis, was the antiapoptotic gene Tsc22d3, also referred to as GILZ (glucocorticoid-induced leucine zipper). The pathway by which DEX suppressed cell death towards the end of the culture period was also confirmed by showing involvement of glucocorticoid receptors and GILZ through studies using the glucocorticoid antagonist mifepristone (RU-486). These findings advance the understanding of the mechanism by which DEX suppresses cell death in CHO cells and provide a rationale for the application of glucocorticoids in CHO cell culture processes.

183 ||| 24574274 ||| (1, 0.41),(8, 0.37) ||| 1 ||| 8 ||| Efficiency improvement of an antibody production process by increasing the inoculum density. Increasing economic pressure is the main driving force to enhance the efficiency of existing processes. We developed a perfusion strategy for a seed train reactor to generate a higher inoculum density for a subsequent fed batch production culture. A higher inoculum density can reduce culture duration without compromising product titers. Hence, a better capacity utilization can be achieved. The perfusion strategy was planned to be implemented in an existing large scale antibody production process. Therefore, facility and process constraints had to be considered. This article describes the initial development steps. Using a proprietary medium and a Chinese hamster ovary cell line expressing an IgG antibody, four different cell retention devices were compared in regard to retention efficiency and reliability. Two devices were selected for further process refinement, a centrifuge and an inclined gravitational settler. A concentrated feed medium was developed to meet facility constraints regarding maximum accumulated perfundate volume. A 2-day batch phase followed by 5 days of perfusion resulted in cell densities of 1.6 x 10(10) cells L(-1) , a 3.5 fold increase compared to batch cultivations. Two reactor volumes of concentrated feed medium were needed to achieve this goal. Eleven cultivations were carried out in bench and 50 L reactors showing acceptable reproducibility and ease of scale up. In addition, it was shown that at least three perfusion phases can be combined within a repeated perfusion strategy.

184 ||| 10649230 ||| (1, 0.41),(7, 0.39) ||| 1 ||| 8 ||| Part II. Overexpression of bcl-2 family members enhances survival of mammalian cells in response to various culture insults. A number of bioreactor configurations have been developed for the manufacture of products from mammalian cell hosts. Even in the most efficient of these, however, problems such as nutrient exhaustion, growth factor deprivation, and toxin accumulations may arise. Consequently, the current effort focused on the feasibility of overexpressing anti-apoptosis genes in baby hamster kidney (BHK) and Chinese hamster ovary (CHO) cells as a means of limiting cell death upon exposure to three such insults. Extended periods of glucose deprivation, serum withdrawal, and treatment with ammonium chloride each caused significant damage, often apoptotic in nature, to BHK and CHO cells, typically rendering cultures completely nonviable. The overexpression of bcl-2 and bcl-x(L), however, was able to abrogate the cell death in BHK cultures, though to varying degrees. For instance, the presence of Bcl-2, which did little to suppress apoptosis upon glucose deprivation, significantly improved the viabilities of these cells during serum withdrawal. In contrast, bcl-x(L) overexpression provided BHK cells with enhanced protection in the absence of glucose, allowing cultures to remain viable throughout the entire three week study. CHO cultures, on the other hand, displayed similar trends in survival in response to both glucose and serum deprivation. During these studies, Bcl-x(L) was consistently able to afford cells the highest degree of protection, though Bcl-2 also enhanced culture viabilities and viable numbers. Death suppression following exposure to 50 mM ammonium chloride was observed to a limited extent in both BHK and CHO cells overexpressing bcl-2 and bcl-x(L). However, even during such harsh treatment, Bcl-x(L) was able to enhance the survival of both cultures, providing CHO cells with viable numbers that were nearly 20-fold that of the controls after five days of exposure. Furthermore, the extensions in cell survival provided by the anti-apoptosis gene products enabled the recovery of many of the cultures during rescue attempts in which the death-inducing stimulus was removed. Clearly, engineering cells to better withstand and recover from the insults common during the large scale cultivation of mammalian cells has a number of potential applications in the biopharmaceutical industries where cell death can limit culture productivities.

185 ||| 22711553 ||| (1, 0.41),(5, 0.31) ||| 1 ||| 8 ||| LC-MS-based metabolic characterization of high monoclonal antibody-producing Chinese hamster ovary cells. The selection of suitable mammalian cell lines with high specific productivities is a crucial aspect of large-scale recombinant protein production. This study utilizes a metabolomics approach to elucidate the key characteristics of Chinese hamster ovary (CHO) cells with high monoclonal antibody productivities (q(mAb)). Liquid chromatography-mass spectrometry (LC-MS)-based intracellular metabolite profiles of eight single cell clones with high and low q(mAb) were obtained at the mid-exponential phase during shake flask batch cultures. Orthogonal projection to latent structures discriminant analysis (OPLS-DA) subsequently revealed key differences between the high and low q(mAb) clones, as indicated by the variable importance for projection (VIP) scores. The mass peaks were further examined for their potential association with q(mAb) across all clones using Pearson's correlation analysis. Lastly, the identities of metabolites with high VIP and correlation scores were confirmed by comparison with standards through LC-MS-MS. A total of seven metabolites were identified-NADH, FAD, reduced and oxidized glutathione, and three activated sugar precursors. These metabolites are involved in key cellular pathways of citric acid cycle, oxidative phosphorylation, glutathione metabolism, and protein glycosylation. To our knowledge, this is the first study to identify metabolites that are associated closely with q(mAb). The results suggest that the high producers had elevated levels of specific metabolites to better regulate their redox status. This is likely to facilitate the generation of energy and activated sugar precursors to meet the demands of producing more glycosylated recombinant monoclonal antibodies.

186 ||| 23376592 ||| (1, 0.41),(2, 0.39) ||| 1 ||| 8 ||| Stable inhibition of mmu-miR-466h-5p improves apoptosis resistance and protein production in CHO cells. MiRNAs have been shown to be involved in regulation of multiple cellular processes including apoptosis. Since a single miRNA can affect the expression of several genes, the utilization of miRNAs for apoptosis engineering in mammalian cells can be more efficient than the conventional approach of manipulating a single gene. Mmu-miR-466h-5p was previously shown to have a pro-apoptotic role in CHO cells by reducing the expression of several anti-apoptotic genes and its transient inhibition delayed both the activation of Caspase-3/7 and the loss of cell viability. The present study evaluates the effect of stable inhibition of mmu-miR-466h-5p in CHO cells on their ability to resist apoptosis onset and their production properties. The expression of mmu-miR-466h-5p in the engineered anti-miR-466h CHO cell line was significantly lower than in the negative control and the parental CHO cells. These engineered cells reached higher maximum viable cell density and extended viability compared with negative control and parental CHO cells in batch cell cultures which resulted in the 53.8% and 41.6% increase of integral viable cells. The extended viability of anti-miR-466h CHO cells was the result of delayed Caspase-3/7 activation by more than 35h, and the increased levels of its anti-apoptotic gene targets (smo, stat5a, dad1, birc6, and bcl2l2) to between 2.1- and 12.5-fold compared with the negative control CHO in apoptotic conditions. The expression of secreted alkaline phosphatase (SEAP) increased 43% and the cell-specific productivity increased 11% in the stable pools of anti-miR-466h CHO compared with the stable pools of negative control CHO cells. The above results demonstrate the potential of this novel approach to create more productive cell lines through stable manipulation of specific miRNA expression.

187 ||| 23338789 ||| (1, 0.41),(2, 0.31) ||| 1 ||| 8 ||| Co-expression of two fibrolytic enzyme genes in CHO cells and transgenic mice. Cellulose is the main non-starch polysaccharides (NSP) in plant cell walls and acts as anti-nutritional factor in animal feed. However, monogastric animals do not synthesize enzymes that cleave such plant structural polysaccharides and thus waste of resources and pollute the environment. We described the vectors construction and co-expressions of a multi-functional cellulase EGX (with the activities of exo-beta-1,4-glucanase, endo-beta-1,4-glucanase, and endo-beta-1,4-xylanase activities) from mollusca, Ampullaria crossean and a beta-glucosidase BGL1 from Asperjillus niger in CHO cells and the transgenic mice. The recombinant enzymes were synthesised, secreted by the direction of pig PSP signal peptide and functionally active in the eukaryote systems including both of CHO cells and transgenic mice by RT-PCR analysis, western blot analysis and cellulolytic enzymes activities assays. Expressions were salivary glands-specific dependent under the control of pig PSP promoter in transgenic mice. 2A peptide was used as the self-cleaving sequence to mediate co-expression of the fusion genes and the cleavage efficiency was very high both in vitro and in vivo according to the western blot analysis. In summary, we have demonstrated that the single ORF containing EGX and BGL1 were co-expressed by 2A peptide in CHO cells and transgenic mice. It presents a viable technology for efficient disruption of plant cell wall and liberation of nutrients. To our knowledge, this is the first report using 2A sequence to produce multiple cellulases in mammalian cells and transgenic animals.

188 ||| 17099908 ||| (1, 0.41) ||| 1 ||| 8 ||| Enhanced cell culture performance using inducible anti-apoptotic genes E1B-19K and Aven in the production of a monoclonal antibody with Chinese hamster ovary cells. Mammalian cells are used for the production of numerous biologics including monoclonal antibodies. Unfortunately, mammalian cells can lose viability at later stages in the cell culture process. In this study, the effects of expressing the anti-apoptosis genes, E1B-19K and Aven, separately and in combination on cell growth, survival, and monoclonal antibody (MAb) production were investigated for a commercial Chinese Hamster Ovary (CHO) mammalian cell line. CHO cells were observed to undergo apoptosis following a model insult, glucose deprivation, and at later stages of batch cell culture. The CHO cell line was then genetically modified to express the anti-apoptotic proteins E1B-19K and/or Aven using an ecdysone-inducible expression system. Stable transfected pools induced to express Aven or E1B-19K alone were found to survive 1-2 days longer than the parent cell line following glucose deprivation while the expression of both genes in concert increased cell survival by 3 days. In spinner flask batch studies, a clonal isolate engineered to express both anti-apoptosis genes exhibited a longer operating lifetime and higher final MAb titer as a result of higher viable cell densities and viabilities. Interestingly, survival was increased in the absence of an inducer, most likely as a result of leaky expression of the anti-apoptosis genes confirmed in subsequent PCR studies. In fed-batch bioreactors, the expression of both anti-apoptosis genes resulted in higher growth rates and cell densities in the exponential phase and significantly higher viable cell densities, viabilities, and extended survival during the post-exponential phase. As a result, the integral of viable cells (IVC) was between 40 and 100% higher for cell lines engineered to express both Aven and E1B-19K in concert, and the operational lifetime of the fed-batch bioreactors was increased from 2 to 5 days. The maximum titers of MAb were also increased by 40-55% for bioreactors containing cells expressing Aven and E1B-19K. These increases in volumetric productivity arose primarily from enhancements in viable cell density over the course of the fed-batch culture period since the specific productivities for the cells expressing anti-apoptosis genes were comparable or slightly lower than the parental hosts. These results demonstrate that expression of anti-apoptosis genes can enhance culture performance and increase MAb titers for mammalian CHO cell cultures especially under conditions such as extended fed-batch bioreactor operation.

189 ||| 23589471 ||| (1, 0.40),(8, 0.31) ||| 1 ||| 8 ||| Near-infrared and two-dimensional fluorescence spectroscopy monitoring of monoclonal antibody fermentation media quality: aged media decreases cell growth. In biomanufacturing processes, the influence of feedstock components on product yield and quality is considerable and often poorly understood. Here we describe the capabilities of near-infrared spectroscopy (NIRS) and two dimensional (2D)-fluorescence spectroscopy in detecting chemical changes over time in two types of culture media (one basal media and one feed media) used in the production of monoclonal antibodies (mAbs) by Chinese hamster ovary (CHO) cells. Both spectroscopies were able to detect compositional changes in basal media over storage period of 12 weeks. NIRS was more effective in detecting changes in feed medium composition. The impact of storage time in process performance was evaluated by using aged media components in mAb cultivations. The study suggests that basal media aging results in a decrease of the integral of viable cells (IVC) (cell growth over time), while product titer is not significantly affected. Feed media appears to be less sensitive to storage and no correlation between the age of the media and cell culture performance was detected. Results obtained provide a basis on which to further improve cell culture raw material quality assessment using vibrational (e.g. NIRS) and optical (e.g. 2D-fluorescence) spectroscopic methods.

190 ||| 18189137 ||| (1, 0.40),(7, 0.32) ||| 1 ||| 8 ||| The role of Bcl-2 and its combined effect with p21CIP1 in adaptation of CHO cells to suspension and protein-free culture. The overexpression of the antiapoptotic gene Bcl-2 has been previously shown to protect cells from undergoing apoptosis during exposure to environmental stress. There is strong evidence that, in addition to its well-known effects on apoptosis, Bcl-2 is involved in antioxidant protection and regulation of cell cycle progression. To determine if the overexpression of Bcl-2 could improve the process of adaptation to suspension and protein-free growth environments, we have studied the growth and viability of anchorage-dependent Chinese hamster ovary cell lines that differ only in there expression of Bcl-2. In addition, we examined the effect of combining Bcl-2 and p21CIP1 expression during adaptation to suspension and protein-free environments. The results of this study provide evidence of a clear reduction in the overall time required for the process of adaptation to both suspension and protein-free environments in Bcl-2 expressing cultures and that through the combined expression of p21CIP1 and Bcl-2, it is possible to further reduce the time. The Bcl-2 results support the well-demonstrated concept that this protein plays an important role in apoptotic signaling pathways and suggest that it may also provide more diverse functions beside its death-inhibiting role.

191 ||| 23763710 ||| (1, 0.40),(6, 0.30) ||| 1 ||| 8 ||| Identification of autocrine growth factors secreted by CHO cells for applications in single-cell cloning media. Chinese hamster ovary (CHO) cell lines are widely used for the expression of therapeutic recombinant proteins, including monoclonal antibodies and other biologics. For manufacturing, cells derived from a single-cell clone are typically used to ensure product consistency. Presently, fetal bovine serum (FBS) is commonly used to support low cell density cultures to obtain clonal cell populations because cells grow slowly, or even do not survive at low cell densities in protein-free media. However, regulatory authorities have discouraged the use of FBS to reduce the risk of contamination by adventitious agents from animal-derived components. In this study, we demonstrated how a complementary mass spectrometry-based shotgun proteomics strategy enabled the identification of autocrine growth factors in CHO cell-conditioned media, which has led to the development of a fully defined single-cell cloning media that is serum and animal component-free. Out of 290 secreted proteins that were identified, eight secreted growth factors were reported for the first time from CHO cell cultures. By supplementing a combination of these growth factors to protein-free basal media, single cell growth of CHO cells was improved with cloning efficiencies of up to 30%, a 2-fold improvement compared to unsupplemented basal media. Complementary effects of these autocrine growth factors with other paracrine growth factors were also demonstrated when the mixture improved cloning efficiency to 42%, similar to that for the conditioned medium.

192 ||| 21373840 ||| (1, 0.40) ||| 1 ||| 8 ||| NMR-based metabolomics of mammalian cell and tissue cultures. NMR spectroscopy was used to evaluate growth media and the cellular metabolome in two systems of interest to biomedical research. The first of these was a Chinese hamster ovary cell line engineered to express a recombinant protein. Here, NMR spectroscopy and a quantum mechanical total line shape analysis were utilized to quantify 30 metabolites such as amino acids, Krebs cycle intermediates, activated sugars, cofactors, and others in both media and cell extracts. The impact of bioreactor scale and addition of anti-apoptotic agents to the media on the extracellular and intracellular metabolome indicated changes in metabolic pathways of energy utilization. These results shed light into culture parameters that can be manipulated to optimize growth and protein production. Second, metabolomic analysis was performed on the superfusion media in a common model used for drug metabolism and toxicology studies, in vitro liver slices. In this study, it is demonstrated that two of the 48 standard media components, choline and histidine are depleted at a faster rate than many other nutrients. Augmenting the starting media with extra choline and histidine improves the long-term liver slice viability as measured by higher tissues levels of lactate dehydrogenase (LDH), glutathione and ATP, as well as lower LDH levels in the media at time points out to 94 h after initiation of incubation. In both models, media components and cellular metabolites are measured over time and correlated with currently accepted endpoint measures.

193 ||| 21901863 ||| (1, 0.39),(8, 0.37) ||| 1 ||| 8 ||| Use of a Plackett-Burman statistical design to determine the effect of selected amino acids on monoclonal antibody production in CHO cells. Culture media design is central to the optimization of monoclonal antibody (mAb) production. Although general strategies do not currently exist for optimization of culture media, the combined use of statistical design and analysis of experiments and strategies based on simple material balances can facilitate culture media design. In this study, we evaluate the effect of selected amino acids on the growth rate and monoclonal antibody production of a Chinese hamster ovary DG-44 (CHO-DG44) cell line. These amino acids were selected based on their relative mass fraction in the specific mAb produced in this study, their consumption rate during bioreactor experiments, and also through a literature review. A Plackett-Burman statistical design was conducted to minimize the number of experiments needed to obtain statistically relevant information. The effect of this set of amino acids was evaluated during exponential cell culture (considering viable cell concentration and the specific growth rate as main output variables) and during the high cell-density stage (considering mAb final concentration and specific productivity as relevant output variables). For this particular cell line, leucine (Leu) and arginine (Arg) had the highest negative and positive effects on cell viability, respectively; Leu and threonine (Thr) had the highest negative effect on growth rate, and valine (Val) and Arg demonstrated the highest positive impact on mAb final concentration. Results suggest the pertinence of a two-stage strategy for amino acid supplementation, with a mixture optimized for cell growth and a different amino acid mixture for mAb production at high density.

194 ||| 23306891 ||| (1, 0.39),(8, 0.32) ||| 1 ||| 8 ||| Advances and drawbacks of the adaptation to serum-free culture of CHO-K1 cells for monoclonal antibody production. Currently, mammalian cell technology has become the focus of biopharmaceutical production, with strict regulatory scrutiny of the techniques employed. Major concerns about the presence of animal-derived components in the culture media led to the development of serum-free (SF) culture processes. However, cell adaptation to SF conditions is still a major challenge and limiting step of process development. Thus, this study aims to assess the impact of SF adaptation on monoclonal antibody (mAb) production, identify the most critical steps of cell adaptation to the SF EX-CELL medium, and create basic process guidelines. The success of SF adaptation was dependent on critical steps that included accentuated cell sensitivity to common culture procedures (centrifugation, trypsinization), initial cell concentration, time given at each step of serum reduction, and, most importantly, medium supplements used to support adaptation. Indeed, only one of the five supplement combinations assessed (rhinsulin, ammonium metavanadate, nickel chloride, and stannous chloride) succeeded for the Chinese hamster ovary-K1 cell line used. This work also revealed that the chemically defined EX-CELL medium benefits mAb production in comparison with the general purpose Dulbecco's Modified Eagle's Medium, but the complete removal of serum attenuates these positive effects.

195 ||| 23794452 ||| (1, 0.39),(5, 0.37) ||| 1 ||| 8 ||| Metabolic signatures of GS-CHO cell clones associated with butyrate treatment and culture phase transition. Chinese hamster ovary (CHO) cells are preferred hosts for the production of recombinant biopharmaceuticals. Efforts to optimize these bioprocesses have largely relied on empirical experience and our knowledge of cellular behavior in culture is incomplete. More recently, comprehensive investigations of metabolic network operation have started to be used to uncover traits associated with optimal growth and recombinant protein production. In this work, we used (1) H-nuclear magnetic resonance ((1) H-NMR) to analyze the supernatants of glutamine-synthetase (GS)-CHO cell clones expressing variable amounts of an IgG4 under control and butyrate-treated conditions. Exometabolomic data revealed accumulation of several metabolic by-products, indicating inefficiencies at different metabolic nodes. These data were contextualized in a detailed network and the cellular fluxomes estimated through metabolic flux analysis. This approach allowed comparing metabolic activity across different clones, growth phases and culture conditions, in particular the efficiency pertaining to carbon lost to glycerol and lactate accumulation and the characteristic nitrogen metabolism involving high asparagine and serine uptake rates. Importantly, this study shows that early butyrate treatment has a marked effect on sustaining high nutrient consumption along culture time, being more pronounced during the stationary phase when extra energy generation and biosynthetic activity is fueled to increase IgG formation. Collectively, the information generated contributes to deepening our understanding of CHO cells metabolism in culture, facilitating future design of improved bioprocesses.

196 ||| 25132658 ||| (1, 0.39),(3, 0.35) ||| 1 ||| 8 ||| Cell culture media supplementation of uncommonly used sugars sucrose and tagatose for the targeted shifting of protein glycosylation profiles of recombinant protein therapeutics. Protein glycosylation is an important post-translational modification toward the structure and function of recombinant therapeutics. The addition of oligosaccharides to recombinant proteins has been shown to greatly influence the overall physiochemical attributes of many proteins. It is for this reason that protein glycosylation is monitored by the developer of a recombinant protein therapeutic, and why protein glycosylation is typically considered a critical quality attribute. In this work, we highlight a systematic study toward the supplementation of sucrose and tagatose into cell culture media for the targeted modulation of protein glycosylation profiles on recombinant proteins. Both sugars were found to affect oligosaccharide maturation resulting in an increase in the percentage of high mannose N-glycan species, as well as a concomitant reduction in fucosylation. The latter effect was demonstrated to increase antibody-dependent cell-mediated cytotoxicity for a recombinant antibody. These aforementioned results were found to be reproducible at different scales, and across different Chinese hamster ovary cell lines. Through the selective supplementation of these described sugars, the targeted modulation of protein glycosylation profiles is demonstrated, as well as yet another tool in the cell culture toolbox for ensuring product comparability.

197 ||| 23777858 ||| (1, 0.39),(2, 0.32) ||| 1 ||| 8 ||| Engineering Chinese hamster ovary (CHO) cells for producing recombinant proteins with simple glycoforms by zinc-finger nuclease (ZFN)-mediated gene knockout of mannosyl (alpha-1,3-)-glycoprotein beta-1,2-N-acetylglucosaminyltransferase (Mgat1). While complex N-linked glycoforms are often desired in biotherapeutic protein production, proteins with simple, homogeneous glycan structure have implications for X-ray crystallography and for recombinant therapeutics targeted to the mannose receptor of antigen presenting cells. Mannosyl (alpha-1,3-)-glycoprotein beta-1,2-N-acetylglucosaminyltransferase (Mgat1, also called GnTI) adds N-acetylglucosamine to the Man5GlcNAc2 (Man5) N-glycan structure as part of complex N-glycan synthesis. Here, we report the use of zinc-finger nuclease (ZFN) genome editing technology to create Mgat1 disrupted Chinese hamster ovary (CHO) cell lines. These cell lines allow for the production of recombinant proteins with Man5 as the predominant N-linked glycosylation species. This method provides advantages over previously reported methods to create Mgat1-deficient cell lines. The use of ZFN-based genome editing eliminates potential regulatory concerns associated with random chemical mutagenesis, while retaining the robust growth and productivity characteristics of the parental cell lines. These Mgat1 disrupted cell lines may be used to produce mannose receptor-targeted therapeutic proteins. Cell line generation work can be performed in both Mgat1 disrupted and wild-type host cell lines to conduct X-ray crystallography studies of protein therapeutics in the same cell line used for production.

198 ||| 18726962 ||| (1, 0.39) ||| 1 ||| 8 ||| Comparative transcriptome analysis to unveil genes affecting recombinant protein productivity in mammalian cells. Low temperature culture (33 degrees C) has been shown to enhance the specific productivity of recombinant antibodies in Chinese hamster ovary (CHO) cells but did not affect antibody productivity in hybridoma (MAK) cells. We probed the transcriptional response of both cells undergoing temperature shift using cDNA microarrays. Among the orthologous gene probes, common trends in the expression changes between CHO and MAK are not prominent. Instead, many transcriptional changes were specific to only one cell line. Notably, oxidative phosphorylation and ribosomal genes were downregulated in MAK but not in CHO. Conversely, several protein trafficking genes and cytoskeleton elements were upregulated in CHO but remained unchanged in MAK. Interestingly, at 33 degrees C, immunoglobulin heavy and light chain showed no significant changes in CHO, but the immunoglobulin light chain was downregulated in MAK. Overall, a clear distinction in the transcriptional response to low temperature was seen in the two cell lines. To further elucidate the set of genes responsible for increased antibody productivity, the expression data of low temperature cultures was compared to that of butyrate treatment which increased specific antibody productivity in both cell lines. Genes which are commonly differentially expressed under conditions that increased productivity are likely to reflect functional classes that are important in the productivity changes. This comparative transcriptome analysis suggests that vesicle trafficking, endocytosis and cytoskeletal elements are involved in increased specific antibody productivity.

199 ||| 18163642 ||| (1, 0.39) ||| 1 ||| 8 ||| Proteomic profiling of secreted proteins from CHO cells using Surface-Enhanced Laser desorption ionization time-of-flight mass spectrometry. Chinese hamster ovary (CHO) cells are the most commonly used host cell line for the production of recombinant biopharmaceuticals. These biopharmaceuticals are typically secreted from CHO cells and purified from harvested cell culture media. The purpose of this study was to investigate changes in the secreted proteome of CHO cells over the various stages of the growth cycle using Surface Enhanced Laser desorption ionization time-of-flight mass spectrometry (SELDI-TOF MS). Conditioned media samples were collected each day over a 6 day growth period from CHO-K1 cells grown in low serum (0.5% FBS) conditions in monolayer culture. Samples were profiled on a number of ProteinChip arrays with different chromatographic surfaces. From this study, 24 proteins were found to be differentially regulated at different phases of the growth cycle in CHO-K1 cells, when profiled on two chromatographic surfaces, Q10 (anionic) and IMAC30 (metal affinity) ProteinChip arrays.

200 ||| 25846330 ||| (1, 0.38),(5, 0.36) ||| 1 ||| 8 ||| Benchmarking of commercially available CHO cell culture media for antibody production. In this study, eight commercially available, chemically defined Chinese hamster ovary (CHO) cell culture media from different vendors were evaluated in batch culture using an IgG-producing CHO DG44 cell line as a model. Medium adaptation revealed that the occurrence of even small aggregates might be a good indicator of cell growth performance in subsequent high cell density cultures. Batch experiments confirmed that the culture medium has a significant impact on bioprocess performance, but high amino acid concentrations alone were not sufficient to ensure superior cell growth and high antibody production. However, some key amino acids that were limiting in most media could be identified. Unbalanced glucose and amino acids led to high cell-specific lactate and ammonium production rates. In some media, persistently high glucose concentrations probably induced the suppression of respiration and oxidative phosphorylation, known as Crabtree effect, which resulted in high cell-specific glycolysis rates along with a continuous and high lactate production. In additional experiments, two of the eight basal media were supplemented with feeds from two different manufacturers in six combinations, in order to understand the combined impact of media and feeds on cell metabolism in a CHO fed-batch process. Cell growth, nutrient consumption and metabolite production rates, antibody production, and IgG quality were evaluated in detail. Concentrated feed supplements boosted cell concentrations almost threefold and antibody titers up to sevenfold. Depending on the fed-batch strategy, fourfold higher peak cell concentrations and eightfold increased IgG titers (up to 5.8 g/L) were achieved. The glycolytic flux was remarkably similar among the fed-batches; however, substantially different specific lactate production rates were observed in the different media and feed combinations. Further analysis revealed that in addition to the feed additives, the basal medium can make a considerable contribution to the ammonium metabolism of the cells. The glycosylation of the recombinant antibody was influenced by the selection of basal medium and feeds. Differences of up to 50 % in the monogalacto-fucosylated (G1F) and high mannose fraction of the IgG were observed.

201 ||| 25062658 ||| (1, 0.38),(5, 0.31) ||| 1 ||| 8 ||| Glycosylation-related genes in NS0 cells are insensitive to moderately elevated ammonium concentrations. NS0 and Chinese hamster ovary (CHO) cell lines are used to produce recombinant proteins for human therapeutics; however, ammonium accumulation can negatively impact cell growth, recombinant protein production, and protein glycosylation. To improve product quality and decrease costs, the relationship between ammonium and protein glycosylation needs to be elucidated. While ammonium has been shown to adversely affect glycosylation-related gene expression in CHO cells, NS0 studies have not been performed. Therefore, this study sought to determine if glycosylation in NS0 cells were ammonium-sensitive at the gene expression level. Using a DNA microarray that contained mouse glycosylation-related and housekeeping genes, these genes were analyzed in response to various culture conditions - elevated ammonium, elevated salt, and elevated ammonium with proline. Surprisingly, no significant differences in gene expression levels were observed between the control and these conditions. Further, the elevated ammonium cultures were analyzed using real-time quantitative reverse transcriptase PCR (qRT-PCR) for key glycosylation genes, and the qRT-PCR results corroborated the DNA microarray results, demonstrating that NS0 cells are ammonium-insensitive at the gene expression level. Since NS0 are known to have elevated nucleotide sugar pools under ammonium stress, and none of the genes directly responsible for these metabolic pools were changed, consequently cellular control at the translational or substrate-level must be responsible for the universally observed decreased glycosylation quality under elevated ammonium.

202 ||| 25565276 ||| (1, 0.38),(3, 0.33) ||| 1 ||| 8 ||| A robust method for increasing Fc glycan high mannose level of recombinant antibodies. High mannose (HM) glycoforms on antibody Fc glycan are recognized as critical quality attributes for therapeutic antibody products. Methods to control HM have been largely empirical, and it is challenging to target a desired HM level during antibody process development. A novel and robust method to increase antibody HM glycoforms is demonstrated in this study using multiple antibodies and cell lines without adversely impacting cell culture performance, including viable cell density, viability, and protein titer. This approach utilizes mannose as a carbon source and the ratio of mannose to total hexose (glucose and mannose) in feed media determines the extent of HM glycan content of an antibody expressed in cell culture. Scale-up of this strategy from 3 mL small scale plate to bioreactor (1.5 L) is also demonstrated with comparable results. Further full glycan map analysis shows that HM increase predominantly correlates with the decrease in G0F glycan, with minimum impact on other glycoforms. Possible hypotheses for the HM glycan modulation using mannose as carbon source are also discussed. Three pathways, including GDP-mannose biosynthesis, early protein glycosylation and UDP-N-acetylglucosamine biosynthesis, might be involved and contribute to this HM modulation.

203 ||| 19367714 ||| (1, 0.38),(3, 0.31) ||| 1 ||| 8 ||| 2-D DIGE to expedite downstream process development for human monoclonal antibody purification. Two-dimensional fluorescence difference gel electrophoresis (2-D DIGE) is an established method for assessing protein expression strategies, understanding pathogenesis mechanisms, characterizing biomarkers, and controlling therapeutic processes. We applied 2-D DIGE to facilitate the development of a purification process for a recombinant IgG1 antibody against Rhesus D antigen expressed by Chinese hamster ovary cells. The variability of two expression clones as well as the influence of cell viability on the host-cell protein pattern was assessed quantitatively. Up to 800 different spots were identified. 2-D DIGE showed that differences in cell viability had more influence on the protein expression pattern than did the expression clone itself. After purification of the IgG from different culture supernatants, the protein patterns on 2-D DIGE were identical, indicating the validity of purification scheme.

204 ||| 21446956 ||| (1, 0.38) ||| 1 ||| 8 ||| Increase in efficiency of media utilization for recombinant protein production in Chinese hamster ovary culture through dilution. Animal cells are extensively used for the large-scale production of recombinant proteins. Processes and genetically engineered cell lines have been developed to enhance longevity of the culture and increase protein productivity. In this study, we tested the effect of diluting a culture of Chinese hamster ovary (CHO) cells with phosphate-buffered saline (PBS) on cell growth and efficiency of media utilization. An immunoglobulin G-expressing CHO cell line was cultured in CD CHO media followed by dilution of the culture with PBS after the end of the exponential phase. A 28% and 61% increase in protein yield per milliliter of media was observed in the diluted culture in the batch and fed-batch mode with glucose and protein hydrolysate feeding, respectively. To aid in analyzing the potential causes of this observed increase, an unstructured mathematical model was constructed using previously reported kinetics to simulate cell growth, nutrient utilization, and protein production. The model predicts an increase in recombinant protein yield per milliliter of media in PBS diluted cultures under both batch and fed-batch conditions, and suggests that this observed increase could at least partly be due to a decrease in inhibitor concentration in the diluted culture.

205 ||| 10099544 ||| (1, 0.37) ||| 1 ||| 8 ||| Metabolism of peptide amino acids by Chinese hamster ovary cells grown in a complex medium. Metabolic flux analysis is a useful tool for unraveling relationships between metabolism and cell function. Material balancing can be used to provide estimates of major metabolic pathway fluxes, provided all significant metabolite uptake and production rates are measured. Potential sources of metabolizable material in many serum-free media formulations are low molecular weight digests of biological material such as yeast extracts and plant or animal tissue hydrolysates. These digests typically contain large amounts of peptides, which may be utilized as amino acids. This article demonstrates the need for accounting for amino acids liberated from peptides in order to accurately estimate pathway fluxes in Chinese hamster ovary cells grown in a complex (hydrolysate containing) medium. A simplified model of central carbon metabolism provides the framework for analyzing external metabolite measurements. Redundant measurements are included to ensure the consistency of data and assumed biochemistry by comparing redundant measurements with their predicted values from a minimum data set, and by expressing the degree of agreement using a statistical "consistency index." The consistency index tests whether redundancies are satisfied within expected experimental error. For chemostat steady states of CHO cultures grown in a hydrolysate-supplemented medium, consistent data were obtained only when amino acids liberated from peptides were taken into account.

206 ||| 24311306 ||| (1, 0.36),(2, 0.33) ||| 1 ||| 8 ||| Predicting the expression of recombinant monoclonal antibodies in Chinese hamster ovary cells based on sequence features of the CDR3 domain. Despite the development of high-titer bioprocesses capable of producing >10 g L(-1) of recombinant monoclonal antibody (MAb), some so called "difficult-to-express" (DTE) MAbs only reach much lower process titers. For widely utilized "platform" processes the only discrete variable is the protein coding sequence of the recombinant product. However, there has been little systematic study to identify the sequence parameters that affect expression. This information is vital, as it would allow us to rationally design genetic sequence and engineering strategies for optimal bioprocessing. We have therefore developed a new computational tool that enables prediction of MAb titer in Chinese hamster ovary (CHO) cells based on the recombinant coding sequence of the expressed MAb. Model construction utilized a panel of MAbs, which following a 10-day fed-batch transient production process varied in titer 5.6-fold, allowing analysis of the sequence features that impact expression over a range of high and low MAb productivity. The model identified 18 light chain (LC)-specific sequence features within complementarity determining region 3 (CDR3) capable of predicting MAb titer with a root mean square error of 0.585 relative expression units. Furthermore, we identify that CDR3 variation influences the rate of LC-HC dimerization during MAb synthesis, which could be exploited to improve the production of DTE MAb variants via increasing the transfected LC:HC gene ratio. Taken together these data suggest that engineering intervention strategies to improve the expression of DTE recombinant products can be rationally implemented based on an identification of the sequence motifs that render a recombinant product DTE.

207 ||| 25118137 ||| (1, 0.36) ||| 1 ||| 8 ||| Mammalian cell-produced therapeutic proteins: heterogeneity derived from protein degradation. Therapeutic glycoproteins, for example, antibodies (Abs) and Fc fusion proteins when produced in mammalian cells, such as Chinese hamster ovary (CHO) cells generally exhibit heterogeneity. Both the oligosaccharide moiety and the protein moiety contribute to this phenomenon. Non-enzymatic and enzymatic pathways of protein fragmentation generate heterogeneity in the polypeptide backbone. In the non-enzymatic pathway, physical and chemical events such as light, oxidation, and others can cause the protein moiety to become unstable leading to its fragmentation. Intracellular and secreted proteases are involved in the enzymatic degradation of proteins. This degradative process is modulated by the oligosaccharide moiety of the glycoprotein as well as glycosidases, including sialidases that are secreted in the culture medium. This review focuses on the factors that modulate heterogeneity of the protein moiety especially by the enzymatic methods. Availability of the CHO genome database will facilitate the development of host cell lines with minimal degradative properties.

208 ||| 22899543 ||| (1, 0.36) ||| 1 ||| 8 ||| Cell surface lectin array: parameters affecting cell glycan signature. Among the "omics", glycomics is one of the most complex fields and needs complementary strategies of analysis to decipher the "glycan dictionary". As an alternative method, which has developed since the beginning of the 21st century, lectin array technology could generate relevant information related to glycan motifs, accessibility and a number of other valuable insights from molecules (purified and non-purified) or cells. Based on a cell line model, this study deals with the key parameters that influence the whole cell surface glycan interaction with lectin arrays and the consequences on the interpretation and reliability of the results. The comparison between the adherent and suspension forms of Chinese Hamster Ovary (CHO) cells, showed respective glycan signatures, which could be inhibited specifically by neoglycoproteins. The modifications of the respective glycan signatures were also revealed according to the detachment modes and cell growth conditions. Finally the power of lectin array technology was highlighted by the possibility of selecting and characterizing a specific clone from the mother cell line, based on the slight difference determination in the respective glycan signatures.

209 ||| 18623424 ||| (1, 0.35),(6, 0.32) ||| 1 ||| 8 ||| Recombinant cyclin E expression activates proliferation and obviates surface attachment of chinese hamster ovary (CHO) cells in protein-free medium. Exogenous growth factors normally required in cell culture activate cell proliferation via the molecular controls of cell-cycle progression. Highly differing influences of mitogenic stimulation of Chinese hamster ovary (CHO) cells by insulin and basic fibroblast growth factor(bFGF) have been clearly observed in a defined protein-free medium. CHO K1 cells stimulated only with insulin grow with flattened cell morphology and extensive cell-cell contact, whereas stimulation with only bFGF or bFGF plus insulin results in loss of cell-cell contact and a transformed and rounded-up morphology. Compared with insulin-stimulated cells, bFGF-stimulated cells exhibit a relatively long G1, and short S phase, and contain higher levels of cyclin E. Observation of elevated levels of cyclin E in wild-type CHO K1 cells mitogenically stimulated by basic fibroblast growth factor motivated transfection of these cells by a cyclin E expression vector. These transfectants grew rapidly in protein-free basal medium and had similar cyclin b levels, distributions of nuclear cell-cycle times, and cell morphologies as bFGF-stimutated CHO K1 culture. Metabolic engineering of cell-cycle regulation thus bypasses exogenous growth factor requirements, addressing a priority objective in economical, reproducible, and safe biopharmaceutical manufacturing. (c) 1995 John Wiley & Sons Inc.

210 ||| 24093128 ||| (1, 0.35) ||| 1 ||| 8 ||| The challenge of applying Raman spectroscopy to monitor recombinant antibody production. UV resonance Raman (UVRR) spectroscopy combined with chemometric techniques was investigated as a physiochemical tool for monitoring secreted recombinant antibody production in cultures of Chinese hamster ovary (CHO) cells. Due to the enhanced selectivity of the UVRR, spectral variations arising from protein, small molecule substrates, and nucleic acid medium components could be measured simultaneously and we have successfully determined antibody titre. Medium samples were taken during culture of three CHO cell lines: two antibody-producing cell lines and a non-producing cell line, and analysed by UVRR spectroscopy using an excitation laser of 244 nm. Principal component analysis (PCA) was applied to the spectral sets and showed a linear trend over time for the antibody-producing cell lines that was not observed in the non-producing cell line. Partial least squares regression (PLSR) was used to predict antibody titres, glucose utilization and lactate accumulation, and compared very favourably with gold standard data acquired with the much slower techniques of ELISA and liquid chromatography. Further analysis of the UVRR spectral sets using two-dimensional correlation moving windows also revealed that spectral variations due to protein and nucleic acid concentrations in the medium during cell culture varied between each of the three cell lines investigated.

211 ||| 21557201 ||| (1, 0.35) ||| 1 ||| 8 ||| Effects of cell culture conditions on antibody N-linked glycosylation--what affects high mannose 5 glycoform. The glycosylation profile of therapeutic antibodies is routinely analyzed throughout development to monitor the impact of process parameters and to ensure consistency, efficacy, and safety for clinical and commercial batches of therapeutic products. In this study, unusually high levels of the mannose-5 (Man5) glycoform were observed during the early development of a therapeutic antibody produced from a Chinese hamster ovary (CHO) cell line, model cell line A. Follow up studies indicated that the antibody Man5 level was increased throughout the course of cell culture production as a result of increasing cell culture medium osmolality levels and extending culture duration. With model cell line A, Man5 glycosylation increased more than twofold from 12% to 28% in the fed-batch process through a combination of high basal and feed media osmolality and increased run duration. The osmolality and culture duration effects were also observed for four other CHO antibody producing cell lines by adding NaCl in both basal and feed media and extending the culture duration of the cell culture process. Moreover, reduction of Man5 level from model cell line A was achieved by supplementing MnCl2 at appropriate concentrations. To further understand the role of glycosyltransferases in Man5 level, N-acetylglucosaminyltransferase I GnT-I mRNA levels at different osmolality conditions were measured. It has been hypothesized that specific enzyme activity in the glycosylation pathway could have been altered in this fed-batch process.

212 ||| 22806637 ||| (1, 0.34) ||| 1 ||| 8 ||| The dynamics of the CHO host cell protein profile during clarification and protein A capture in a platform antibody purification process. Recombinant protein products such as monoclonal antibodies (mAbs) for use in the clinic must be clear of host cell impurities such as host cell protein (HCP), DNA/RNA, and high molecular weight immunogenic aggregates. Despite the need to remove and monitor HCPs, the nature, and fate of these during downstream processing (DSP) remains poorly characterized. We have applied a proteomic approach to investigate the dynamics and fate of HCPs in the supernatant of a mAb producing cell line during early DSP including centrifugation, depth filtration, and protein A capture chromatography. The primary clarification technique selected was shown to influence the HCP profile that entered subsequent downstream steps. MabSelect protein A chromatography removed the majority of contaminating proteins, however using 2D-PAGE we could visualize not only the antibody species in the eluate (heavy and light chain) but also contaminant HCPs. These data showed that the choice of secondary clarification impacts upon the HCP profile post-protein A chromatography as differences arose in both the presence and abundance of specific HCPs when depth filters were compared. A number of intracellularly located HCPs were identified in protein A elution fractions from a Null cell line culture supernatant including the chaperone Bip/GRP78, heat shock proteins, and the enzyme enolase. We demonstrate that the selection of early DSP steps influences the resulting HCP profile and that 2D-PAGE can be used for monitoring and identification of HCPs post-protein A chromatography. This approach could be used to screen cell lines or hosts to select those with reduced HCP profiles, or to identify HCPs that are problematic and difficult to remove so that cell-engineering approaches can be applied to reduced, or eliminate, such HCPs.

213 ||| 21298341 ||| (1, 0.34) ||| 1 ||| 8 ||| Bioprocess development for the production of mouse-human chimeric anti-epidermal growth factor receptor vIII antibody C12 by suspension culture of recombinant Chinese hamster ovary cells. The mouse-human chimeric anti-epidermal growth factor receptor vIII (EGFRvIII) antibody C12 is a promising candidate for the diagnosis of hepatocellular carcinoma (HCC). In this study, 3 processes were successfully developed to produce C12 by cultivation of recombinant Chinese hamster ovary (CHO-DG44) cells in serum-free medium. The effect of inoculum density was evaluated in batch cultures of shaker flasks to obtain the optimal inoculum density of 5 x 10(5) cells/mL. Then, the basic metabolic characteristics of CHO-C12 cells were studied in stirred bioreactor batch cultures. The results showed that the limiting concentrations of glucose and glutamine were 6 and 1 mM, respectively. The culture process consumed significant amounts of aspartate, glutamate, asparagine, serine, isoleucine, leucine, and lysine. Aspartate, glutamate, asparagine, and serine were particularly exhausted in the early growth stage, thus limiting cell growth and antibody synthesis. Based on these findings, fed-batch and perfusion processes in the bioreactor were successfully developed with a balanced amino acid feed strategy. Fed-batch and especially perfusion culture effectively maintained high cell viability to prolong the culture process. Furthermore, perfusion cultures maximized the efficiency of nutrient utilization; the mean yield coefficient of antibody to consumed glucose was 44.72 mg/g and the mean yield coefficient of glutamine to antibody was 721.40 mg/g. Finally, in small-scale bioreactor culture, the highest total amount of C12 antibody (1,854 mg) was realized in perfusion cultures. Therefore, perfusion culture appears to be the optimal process for small-scale production of C12 antibody by rCHO-C12 cells.

214 ||| 11460250 ||| (1, 0.34) ||| 1 ||| 8 ||| Comparative analysis of two controlled proliferation strategies regarding product quality, influence on tetracycline-regulated gene expression, and productivity. Overexpression of the cyclin-dependent kinase inhibitor p27 and exposure to low temperature (30 degrees C) represent two strategies to establish controlled proliferation processes for production of therapeutic proteins using Chinese hamster ovary (CHO) cells. Here we analyze the effect of growth inhibition on the quality of the human model glycoprotein SEAP (secreted alkaline phosphatase) for both strategies in monoclonal CHO-derived cell lines. Separation of purified SEAP samples using two-dimensional gel electrophoresis showed that production by proliferation-controlled CHO cultures did not alter the overall integrity of the product. Further, oligosaccharide profiles were compared using HPEC-PAD analysis. No differences were detectable between SEAP profiles obtained from p27 growth-arrested and proliferating cultures. However, production at 30 degrees C led to a significant increase in the degree of sialylation, an effect that is generally considered beneficial for the in vivo efficacy of protein therapeutics. In the production context presented here, SEAP expression is controlled by the tetracycline- (tet) repressible gene regulation system. Here we show low temperature-induced upregulation of the tetracycline-dependent transactivator (tTA). This induction has been shown by Northern blot analysis to occur at the mRNA level and is independent of the promoters driving the transactivator. We also describe a novel bottleneck in productivity at low temperature found in p27 growth-arrested CHO cells cultivated at 30 degrees C.

215 ||| 23995617 ||| (1, 0.32),(3, 0.30) ||| 1 ||| 8 ||| Production of stable bispecific IgG1 by controlled Fab-arm exchange: scalability from bench to large-scale manufacturing by application of standard approaches. The manufacturing of bispecific antibodies can be challenging for a variety of reasons. For example, protein expression problems, stability issues, or the use of non-standard approaches for manufacturing can result in poor yield or poor facility fit. In this paper, we demonstrate the use of standard antibody platforms for large-scale manufacturing of bispecific IgG1 by controlled Fab-arm exchange. Two parental antibodies that each contain a single matched point mutation in the CH3 region were separately expressed in Chinese hamster ovary cells and manufactured at 1000 L scale using a platform fed-batch and purification process that was designed for standard antibody production. The bispecific antibody was generated by mixing the two parental molecules under controlled reducing conditions, resulting in efficient Fab-arm exchange of>95% at kg scale. The reductant was removed via diafiltration, resulting in spontaneous reoxidation of interchain disulfide bonds. Aside from the bispecific nature of the molecule, extensive characterization demonstrated that the IgG1 structural integrity was maintained, including function and stability. These results demonstrate the suitability of this bispecific IgG1 format for commercial-scale manufacturing using standard antibody manufacturing techniques.

216 ||| 25202924 ||| (1, 0.30) ||| 1 ||| 8 ||| In situ cell retention of a CHO culture by a reverse-flow diafiltration membrane bioreactor. Heterogeneities occur in various bioreactor designs including cell retention devices. Whereas in external devices changing environmental conditions cannot be prevented, cells are retained in their optimal environment in internal devices. Conventional reverse-flow diafiltration utilizes an internal membrane device, but pulsed feeding causes temporal heterogeneities. In this study, the influence of conventional reverse-flow diafiltration on the yeast Hansenula polymorpha is investigated. Alternating 180 s of feeding with 360 s of non-feeding at a dilution rate of 0.2 h(-1) results in an oscillating DOT signal with an amplitude of 60%. Thereby, induced short-term oxygen limitations result in the formation of ethanol and a reduced product concentration of 25%. This effect is enforced at increased dilution rate. To overcome this cyclic problem, sequential operation of three membranes is introduced. Thus, quasi-continuous feeding is achieved reducing the oscillation of the DOT signal to an amplitude of 20% and 40% for a dilution rate of 0.2 h(-1) and 0.5 h(-1) , respectively. Fermentation conditions characterized by complete absence of oxygen limitation and without formation of overflow metabolites could be obtained for dilution rates from 0.1 h(-1) - 0.5 h(-1) . Thus, sequential operation of three membranes minimizes oscillations in the DOT signal providing a nearly homogenous culture over time.

217 ||| 23826910 ||| (1, 0.30) ||| 1 ||| 8 ||| Application of statistical techniques for elucidating flow cytometric data of batch and fed-batch cultures. The objective of this work is to develop structured, segregated stochastic models for bioprocesses using time-series flow cytometric (FC) data. To this end, mammalian CHO cells were grown in both batch and fed-batch cultures, and their viable cell numbers (VCDs), monoclonal antibody (MAb), cell cycle phases, mitochondria membrane potential/mitochondria mass, Golgi apparatus, and endoplasmic reticulum (ER) were analyzed. For the fed-batch mode, soy hydrolysate was introduced at 24-H intervals. The cytometric data were analyzed for early indicators of growth and productivity by multiple linear regression analysis, which involved taking into account multicollinearity diagnostics, Durbin-Watson statistics, and Houston tests to determine and refine statistically significant correlations between categorical variables (FC parameters) and response variables (yield parameters). The results indicate that the percentage of G1 cells and ER was significantly correlated with VCD and MAb in the case of batch culture, whereas for fed-batch culture, the percentage of G2 cells and ER was correlated significantly. There was a significant difference between cells in the batch and fed-batch cultures in their ER content, suggesting that the increase in protein synthesis as reflected by the ER content and consequent increase in growth rate and MAb productivity both can be monitored at the cellular level by FC analysis of ER content.

218 ||| 20159577 ||| (1, 0.30) ||| 1 ||| 8 ||| Evaluation of Chinese hamster ovary cell stability during repeated batch culture for large-scale antibody production. Pharmaceutical manufacturing plants can be operated continuously for several months. It is therefore important to use cells with long-term stability for the production of active ingredients. We investigated the reliability and long-term stability of an antibody-producing cell line. A recombinant Chinese hamster ovary (CHO) cell line was cultivated in spinner flasks and reactors, including a practical production-scale reactor (1600 L), for 109 days to produce monoclonal antibodies against the HM1.24 antigen. During cultivation, the cells remained stable and there was an increase in the rate of cell proliferation, yielding viable cells at high density. A decrease in cell-specific productivity was associated with this increase in the rate of cell proliferation. The cells were genetically stable and other measures of cellular function remained consistent throughout the cultivation period.

# ----------------------------- TOPIC2 -----------------------------

219 ||| 25482237 ||| (2, 0.99) ||| 2 ||| 2 ||| Toward stable gene expression in CHO cells. Maintaining high gene expression level during long-term culture is critical when producing therapeutic recombinant proteins using mammalian cells. Transcriptional silencing of promoters, most likely due to epigenetic events such as DNA methylation and histone modifications, is one of the major mechanisms causing production instability. Previous studies demonstrated that the core CpG island element (IE) from the hamster adenine phosphoribosyltransferase gene is effective to prevent DNA methylation. We generated one set of modified human cytomegalovirus (hCMV) promoters by insertion of one or two copies of IE in either forward or reverse orientations into different locations of the hCMV promoter. The modified hCMV with one copy of IE inserted between the hCMV enhancer and core promoter in reverse orientation (MR1) was most effective at enhancing expression stability in CHO cells without comprising expression level when compared with the wild type hCMV. We also found that insertion of IE into a chimeric murine CMV (mCMV) enhancer and human elongation factor-1alpha core (hEF) promoter in reverse orientation did not enhance expression stability, indicating that the effect of IE on expression stability is possibly promoter specific.

220 ||| 25158835 ||| (2, 0.99) ||| 2 ||| 2 ||| Accurate comparison of antibody expression levels by reproducible transgene targeting in engineered recombination-competent CHO cells. Over the years, Chinese hamster ovary (CHO) cells have emerged as the major host for expressing biotherapeutic proteins. Traditional methods to generate high-producer cell lines rely on random integration(s) of the gene of interest but have thereby left the identification of bottlenecks as a challenging task. For comparison of different producer cell lines derived from various transfections, a system that provides control over transgene expression behavior is highly needed. This motivated us to develop a novel "DUKX-B11 F3/F" cell line to target different single-chain antibody fragments into the same chromosomal target site by recombinase-mediated cassette exchange (RMCE) using the flippase (FLP)/FLP recognition target (FRT) system. The RMCE-competent cell line contains a gfp reporter fused to a positive/negative selection system flanked by heterospecific FRT (F) variants under control of an external CMV promoter, constructed as "promoter trap". The expression stability and FLP accessibility of the tagged locus was demonstrated by successive rounds of RMCE. As a proof of concept, we performed RMCE using cassettes encoding two different anti-HIV single-chain Fc fragments, 3D6scFv-Fc and 2F5scFv-Fc. Both targeted integrations yielded homogenous cell populations with comparable intracellular product contents and messenger RNA (mRNA) levels but product related differences in specific productivities. These studies confirm the potential of the newly available "DUKX-B11 F3/F" cell line to guide different transgenes into identical transcriptional control regions by RMCE and thereby generate clones with comparable amounts of transgene mRNA. This new host is a prerequisite for cell biology studies of independent transfections and transgenes.

221 ||| 25104235 ||| (2, 0.99) ||| 2 ||| 2 ||| It's time to regulate: coping with product-induced nongenetic clonal instability in CHO cell lines via regulated protein expression. Clonal instability and titer loss during Chinese hamster ovary (CHO) cell line development (CLD) has several underlying causes, the most prominent of which are DNA copy number loss and DNA silencing. However, in some cases, clonal instability is due to the toxicity of the therapeutic protein(s) that clones express. Unlike DNA copy number loss, which may occur in some clones or DNA silencing that is prevalent in certain regions of the genome, the hallmark of product induced clonal instability is its manifestation in all the selected clones. To circumvent such product induced clonal instability, we have developed a vector construct that utilizes a regulated protein expression system in which the constitutive expression of the target protein(s) is prevented unless doxycycline is added to the culture. We have then successfully used this system to express, at high titers, an antibody for which constitutive expression results in clonal instability perhaps due to intracellular accumulation of the antibody. Our data shows that unlike the constitutively expressed or continuously induced clones, uninduced clones do not display instability. Furthermore, maintaining the uninduced clones in culture for months or subjecting them to freeze-thaws did not have any effects on their titers. All together, our findings suggest that a regulated expression system could be suitable for production of difficult proteins that trigger instability.

222 ||| 24789630 ||| (2, 0.99) ||| 2 ||| 2 ||| Insertion of core CpG island element into human CMV promoter for enhancing recombinant protein expression stability in CHO cells. The human cytomegalovirus promoter (hCMV) is susceptible to gene silencing in CHO cells, most likely due to epigenetic events, such as DNA methylation and histone modifications. The core CpG island element (IE) from the hamster adenine phosphoribosyltransferase gene has been shown to prevent DNA methylation. A set of modified hCMV promoters was developed by inserting one or two copies of IE in either forward or reverse orientations either upstream of the hCMV enhancer, between the enhancer and core promoter (CP), or downstream of the CP. The modified hCMV with one copy of IE inserted between the enhancer and core promoter in reverse orientation (MR1) was most effective at enhancing expression stability without compromising expression level when compared with the wild-type (WT) hCMV. A third of 18 EGFP expressing clones generated using MR1 retained 70% of their starting expression level after 8 weeks of culture in the absence of selection pressure, while none of 18 WT hCMV generated clones had expression above 50%. MR1 also improved antibody expression stability of methotrexate (MTX) amplified CHO cell lines. Stably transfected pools generated using MR1 maintained 62% of their original monoclonal antibody titer after 8 weeks of culture in the absence of MTX, compared to only 37% for WT hCMV pools. Low levels of CpG methylation within both WT hCMV and MR1 were observed in all the analyzed cell lines and the methylation levels did not correlate to the expression stability, suggesting IE enhances expression stability by other mechanisms other than preventing methylation.

223 ||| 24615264 ||| (2, 0.99) ||| 2 ||| 2 ||| Synthetic promoters for CHO cell engineering. We describe for the first time the creation of a library of 140 synthetic promoters specifically designed to regulate the expression of recombinant genes in CHO cells. Initially, 10 common viral promoter sequences known to be active in CHO cells were analyzed using bioinformatic sequence analysis programs to determine the identity and relative abundance of transcription factor regulatory elements (TFREs; or transcription factor binding sites) they contained. Based on this, 28 synthetic reporters were constructed that each harbored seven repeats of a discrete TFRE sequence upstream of a minimal CMV core promoter element and secreted alkaline phosphatase (SEAP) reporter gene. After evaluation of the relative activity of TFREs by transient expression in CHO-S cells, we constructed a first generation library of 96 synthetic promoters derived from random ligation of six active TFREs inserted into the same reporter construct backbone. Comparison of the sequence and relative activity of first generation promoters revealed that individual TFRE blocks were either relatively abundant in active promoters (NFkappaB, E-box), equally distributed across promoters of varying activity (C/EBPalpha, GC-box) or relatively abundant in low activity promoters (E4F1, CRE). These data were utilized to create a second generation of 44 synthetic promoters based on random ligation of a fixed ratio of 4 TFREs (NFkappaB 5: E-box 3: C/EBPalpha 1: GC-box 1). Comparison of the sequence and relative activity of second generation promoters revealed that the most active promoters contained relatively high numbers of both NFkappaB and E-box TFREs in approximately equal proportion, with a correspondingly low number of GC-box and C/EBPalpha blocks. The most active second generation promoters achieved approximately twice the activity of a control construct harboring the human cytomegalovirus (CMV) promoter. Lastly, we evaluated the function of a subset of synthetic promoters exhibiting a broad range of activity in different CHO cell host cell lines (CHO-S, CHO-K1, and CHO-DG44) and across extended fed-batch transient expression in CHO-S cells. In general, the different synthetic promoters both maintained their relative activity and the most active promoters consistently and significantly exceeded the activity of the CMV control promoter. For advanced cell engineering strategies our synthetic promoter libraries offer precise control of recombinant transcriptional activity in CHO cells spanning over two orders of magnitude.

224 ||| 24063773 ||| (2, 0.99) ||| 2 ||| 2 ||| A streamlined implementation of the glutamine synthetase-based protein expression system. BACKGROUND: The glutamine synthetase-based protein expression system is widely used in industry and academia for producing recombinant proteins but relies on the cloning of transfected cells, necessitating substantial investments in time and handling. We streamlined the production of protein-producing cultures of Chinese hamster ovary cells using this system by co-expressing green fluorescent protein from an internal ribosomal entry site and selecting for high green fluorescent protein-expressing cells using fluorescence-activated cell sorting. RESULTS: Whereas other expression systems utilizing green fluorescent protein and fluorescence-activated cell sorting-based selection have relied on two or more sorting steps, we obtained stable expression of a test protein at levels >50% of that of an "average" clone and ~40% that of the "best" clone following a single sorting step. Versus clone-based selection, the principal savings are in the number of handling steps (reduced by a third), handling time (reduced by 70%), and the time needed to produce protein-expressing cultures (reduced by ~3 weeks). Coupling the glutamine synthetase-based expression system with product-independent selection in this way also facilitated the production of a hard-to-assay protein. CONCLUSION: Utilizing just a single fluorescence-activated cell sorting-based selection step, the new streamlined implementation of the glutamine synthetase-based protein expression system offers protein yields sufficient for most research purposes, where <10 mg/L of protein expression is often required but relatively large numbers of constructs frequently need to be trialed.

225 ||| 23468139 ||| (2, 0.99) ||| 2 ||| 2 ||| Unstable expression of recombinant antibody during long-term culture of CHO cells is accompanied by histone H3 hypoacetylation. The gradual loss of recombinant protein expression in CHO cell lines during prolonged subculture is a common issue, referred to as instability, which seriously affects the industrial production processes of therapeutic proteins. Loss of recombinant gene copies, due to the genetic instability of CHO cells, and epigenetic silencing of transgene sequences, are the main reported causes of production instability. To increase our understanding on the molecular mechanisms inherent to CHO cells involved in production instability, we explored the molecular features of stable and unstable antibody producing cell lines obtained without gene amplification, to exclude the genetic instability induced by the gene amplification process. The instability of recombinant antibody production during long-term culture was caused by a 48-53% decrease in recombinant mRNA levels without significant loss of recombinant gene copies, but accompanied by a ~45% decrease in histone H3 acetylation (H3ac). Thus, our results suggest a critical role of H3ac in the stability of recombinant protein production.

226 ||| 22510960 ||| (2, 0.99) ||| 2 ||| 2 ||| Development of a novel ER stress based selection system for the isolation of highly productive clones. Most biotherapeutic drugs are recombinant monoclonal antibodies which are mostly produced in monoclonal cell lines derived from Chinese hamster ovary (CHO) cells. Various clones expressing a monoclonal recombinant antibody were analyzed and a correlation of the antibody concentration and the relative mRNA level of calreticulin (CALR), glucose-regulated protein 78 and 94 kDa (GRP78, GRP94) and spliced X-box binding protein 1 (XPB1) was observed. By means of these results we were motivated to establish a novel selection system based on endoplasmic reticulum (ER) stress, which allows the rapid identification and isolation of high-expressing clones out of a pool mainly consisting of low- and medium-producing cells. Several ER stress responsive elements were tested with the aid of a recombinase mediated cassette exchange (RMCE) procedure. Very surprisingly, only GRP78 reporter constructs were strongly stimulated upon antibody expression. Furthermore we found that GRP78 reporter constructs are very suitable to reflect the level of antibody expression (IgG) in recombinant CHO cells. Based on these results, it is concluded, that the novel ER stress based selection system developed during this study is suitable to identify and isolate clones with a high level of antibody expression.

227 ||| 21495018 ||| (2, 0.99) ||| 2 ||| 2 ||| The PiggyBac transposon enhances the frequency of CHO stable cell line generation and yields recombinant lines with superior productivity and stability. Generating stable, high-producing mammalian cell lines is a major bottleneck in the manufacture of recombinant therapeutic proteins. Conventional gene transfer methods for cell line generation rely on random plasmid integration, resulting in unpredictable and highly variable levels of transgene expression. As a consequence, a large number of stably transfected cells must be analyzed to recover a few high-producing clones. Here we present an alternative gene transfer method for cell line generation based on transgene integration mediated by the piggyBac (PB) transposon. Recombinant Chinese hamster ovary (CHO) cell lines expressing a tumor necrosis factor receptor:Fc fusion protein were generated either by PB transposition or by conventional transfection. Polyclonal populations and isolated clonal cell lines were characterized for the level and stability of transgene expression for up to 3 months in serum-free suspension culture. Pools of transposed cells produced up to fourfold more recombinant protein than did the pools generated by standard transfection. For clonal cell lines, the frequency of high-producers was greater following transposition as compared to standard transfection, and these clones had a higher volumetric productivity and a greater number of integrated transgenes than did those generated by standard transfection. In general, the volumetric productivity of the cell pools and individual cell lines generated by transposition was stable for up to 3 months in the absence of selection. Our results indicate that the PB transposon supports the generation of cell lines with high and stable transgene expression at an elevated frequency relative to conventional transfection. Thus, PB-mediated gene delivery is expected to reduce the extent of recombinant cell line screening.

228 ||| 20967750 ||| (2, 0.99) ||| 2 ||| 2 ||| Generation of stable, high-producing CHO cell lines by lentiviral vector-mediated gene transfer in serum-free suspension culture. Lentivirus-derived vectors (LVs) were studied for the generation of stable recombinant Chinese hamster ovary (CHO) cell lines. Stable pools and clones expressing the enhanced green fluorescent protein (eGFP) were selected via fluorescence-activated cell sorting (FACS). For comparison, cell pools and cell lines were also generated by transfection, using the LV transfer plasmid alone. The level and stability of eGFP expression was greater in LV-transduced cell lines and pools than in those established by transfection. CHO cells were also infected at two different multiplicities of infection with an LV co-expressing eGFP and a tumor necrosis factor receptor:Fc fusion protein (TNFR:Fc). At 2-day post-infection, clonal cell lines with high eGFP-specific fluorescence were recovered by FACS. These clones co-expressed TNFR:Fc with yields of 50-250 mg/L in 4-day cultures. The recovered cell lines maintained stable expression over 3 months in serum-free suspension culture without selection. In conclusion, LV-mediated gene transfer provided an efficient alternative to plasmid transfection for the generation of stable and high-producing recombinant cell lines.

229 ||| 20949444 ||| (2, 0.99) ||| 2 ||| 2 ||| Improved recombinant protein yield using a codon deoptimized DHFR selectable marker in a CHEF1 expression plasmid. The CHEF1 expression plasmid utilizes regulatory domains of the Chinese Hamster Elongation Factor 1 (CHEF1) housekeeping gene to drive stable recombinant protein expression in Chinese Hamster Ovary (CHO) cells. Rapid development of stable CHEF1 cell lines is possible, in part, because high titer pools are produced in the absence of methotrexate-induced DHFR amplification. Inhibiting DHFR activity with methotrexate increases the selection pressure on transfected cells and can result in a compensatory event to ensure cell survival, such as dhfr gene amplification or integration into a transcriptionally active genomic site. Methotrexate amplification often results in improved cell line productivity but it is a time-consuming process. Herein, we describe a novel mechanism to increase selection stringency via codon deoptimization of the mouse dhfr selectable marker utilizing hamster codon preference that results in improved expression of a linked recombinant protein compared to wild-type DHFR selection. We show that deoptimizing the translatability of the dhfr gene reduces the expression level of the DHFR protein in CHO transfection pools and derived clones. Lower DHFR expression increases the transfection selection stringency, shown as lower transfection efficiency in pools, and correlates with increased recombinant protein expression in both pools and clones. These results demonstrate a new mechanism for increasing selection stringency and improving recombinant protein production during cell line development without time-consuming gene amplification steps.

230 ||| 18781700 ||| (2, 0.99) ||| 2 ||| 2 ||| Calcium phosphate transfection generates mammalian recombinant cell lines with higher specific productivity than polyfection. Transfection with polyethylenimine (PEI) was evaluated as a method for the generation of recombinant Chinese hamster ovary (CHO DG44) cell lines by direct comparison with calcium phosphate-DNA coprecipitation (CaPO4) using both green fluorescent protein (GFP) and a monoclonal antibody as reporter proteins. Following transfection with a GFP expression vector, the proportion of GFP-positive cells as determined by flow cytometry was fourfold higher for the PEI transfection as compared to the CaPO4 transfection. However, the mean level of transient GFP expression for the cells with the highest level of fluorescence was twofold greater for the CaPO4 transfection. Fluorescence in situ hybridization on metaphase chromosomes from pools of cells grown under selective pressure demonstrated that plasmid integration always occurred at a single site regardless of the transfection method. Importantly, the copy number of integrated plasmids was measurably higher in cells transfected with CaPO4. The efficiency of recombinant cell line recovery under selective pressure was fivefold higher following PEI transfection, but the average specific productivity of a recombinant antibody was about twofold higher for the CaPO4-derived cell lines. Nevertheless, no difference between the two transfection methods was observed in terms of the stability of protein production. These results demonstrated the feasibility of generating recombinant CHO-derived cell lines by PEI transfection. However, this method appeared inferior to CaPO4 transfection with regard to the specific productivity of the recovered cell lines.

231 ||| 18564058 ||| (2, 0.99) ||| 2 ||| 2 ||| Improving human interferon-beta production in mammalian cell lines by insertion of an intronic sequence within its naturally uninterrupted gene. Human beta-interferon is used extensively as a therapeutic agent in a wide variety of diseases, ranging from multiple sclerosis to viral infections. At present, the most common source of interferon-beta is derived from CHO (Chinese-hamster ovary) cells. Interestingly, however, the IFNB gene is characterized by a lack of intronic sequences and therefore does not undergo splicing during its expression pathway. As nuclear processing of pre-mRNA molecules has often been demonstrated to improve production yields of recombinant molecules, we have inserted a heterologous intronic sequence at different positions within the IFNB gene and analysed its effects on protein production. The results obtained in the present study show that the position of intron insertion has profound effects on the expression levels of the IFNB gene and on the nuclear/cytoplasm distribution levels of its mRNA as determined by FISH (fluorescent in situ hybridization) analysis of stably transfected clones. In conclusion, our results provide additional evidence that insertion of intronic sequences may be used to improve protein expression efficiency also in molecules that do not normally undergo any splicing process.

232 ||| 17428585 ||| (2, 0.99) ||| 2 ||| 2 ||| A novel RNA silencing vector to improve antigen expression and stability in Chinese hamster ovary cells. Chinese hamster ovary (CHO) cells and dihydrofolate reductase (dhfr)/methotrexate (MTX) gene amplification system are routinely used to generate stable producer CHO cell clones in biopharmaceutical industries. The present study proposes a novel method by the co-amplification of the silencing vector targeted to dhfr gene for improvements of selecting high-producing clones in dhfr-deficient and wild-type CHO cells. Using the silencing vector also resulted in improving the stability of the recombinant protein expression in the absence of MTX in the CHO/dhFr(-) and wild-type CHO cells. This new method is proposed to generate highly expressed stable cell clones of both dhfr-deficient and wild-type CHO cells for recombinant antigen production. Utilization of the silencing vector designed in this study can improve antigen expression through dhfr-directed gene amplification in other dhfr-competent cell lines for vaccine development.

233 ||| 17350648 ||| (2, 0.99) ||| 2 ||| 2 ||| An efficient and targeted gene integration system for high-level antibody expression. Random integration linking genomic amplification has been used to generate desired cell lines for stable and high-level expressing recombinant antibodies. But this technique is laborious, and the expression level is unpredictable due to position effects. Here, we have constructed a cell-vector system for high-level antibody expression using an FRT/FLP strategy to overcome position effects. The key is to target the FRT sequence to chromosomal locations where there is a high rate of transcription and gene amplification, and the amplified genes can be maintained. To screen desired loci with high transcriptional activity and amplifiable capacity, dual weakened markers (selectable galactosidase and amplifiable dihydrofolate reductase, DHFR) and the FRT sequence were synchronously cloned into a plasmid. After transfection of a Chinese hamster ovary host cell line with this plasmid, we selected 20 candidate cell lines from 721 individual clones. An antibody gene-targeting vector carrying an FRT-fused hygromycin gene was constructed to target antibody genes into the chromosomal FRT site by FLP recombinase. Three out of 20 cell lines can be used as host cells for site-specific recombination. By using southern blot and fluorescence in situ hybridization (FISH), a candidate engineered cell line, number 37, was chosen. It contains a single FRT-tagged locus in its genome. FISH analysis indicated that the antibody genes were all located at the original FRT-tagged locus in the genome of the gene-targeted and gene-amplified cell lines. Three kinds of recombinant antibodies were successfully expressed in candidate cell line 37. The highest producers produced more than 200 mug/ml of the antibody in 6 days of continuous culture in a spinner flask.

234 ||| 16403443 ||| (2, 0.99) ||| 2 ||| 2 ||| Genetic characterization of CHO production host DG44 and derivative recombinant cell lines. The dihydrofolate reductase-deficient Chinese hamster ovary (CHO) cell line DG44 is the dominant mammalian host for recombinant protein manufacturing, in large part because of the availability of a well-characterized genetic selection and amplification system. However, this cell line has not been studied at the cytogenetic level. Here, the first detailed karyotype analysis of DG44 and several recombinant derivative cell lines is described. In contrast to the 22 chromosomes in diploid Chinese hamster cells, DG44 has 20 chromosomes, only seven of which are normal. In addition, four Z group chromosomes, seven derivative chromosomes, and 2 marker chromosomes were identified. For all but one of the 16 DG44-derived recombinant cell lines analyzed, a single integration site was detected by fluorescence in situ hybridization regardless of the gene delivery method (calcium phosphate-DNA coprecipitation or microinjection), the topology of the DNA (circular or linear), or the integrated plasmid copy number (between 1 and 51). Chromosomal aberrations, observed in more than half of the cell lines studied, were mostly unbalanced with examples of aneuploidy, deletions, and complex rearrangements. The results demonstrate that chromosomal aberrations are frequently associated with the establishment of recombinant CHO DG44 cell lines. Noteworthy, there was no direct correlation between the stability of the genome and the stability of recombinant protein expression.

235 ||| 10618644 ||| (2, 0.99) ||| 2 ||| 2 ||| Homogeneous tetracycline-regulatable gene expression in mammalian fibroblasts. The expression of transfected genes in mammalian cells is rapidly repressed by epigenetic mechanisms such that, within a matter of weeks, only a fraction of the cells in most clonal populations still exhibit detectable expression. This problem can become prohibitive when one wants to express two ectopically introduced genes, as is necessary to establish cell lines that harbor genes regulated by the tetracycline-controlled transactivators. We describe an approach to establish Chinese hamster ovary (CHO) cell lines that stably induce a tet-responsive reporter gene in all cells of a transfected clonal population. Screening of more than 100 colonies resulting from a standard co-transfection of the tetracycline transactivator (tTA) with a green fluorescent protein (GFP) reporter plasmid failed to identify a single colony that could induce GFP in more than 20% of cells. The presence of chromatin insulator sequences, previously shown to protect some transfected genes from epigenetic silencing, moderately improved stability but was not sufficient to produce homogeneous transformants. However, when cell lines were first established in which selection could be maintained either for the expression of tTA activity (co-transfection with a tTA-responsive selectable marker) or the presence of tTA mRNA (bicistronic message encoding a selectable marker), these cell lines could be subsequently transfected with the GFP reporter construct, and nearly 10% of the resulting colonies exhibited stable homogeneous tet-responsive GFP expression in 100% of the expanded clonal cell population.

236 ||| 23376841 ||| (2, 0.98) ||| 2 ||| 2 ||| The isolation of CHO cells with a site conferring a high and reproducible transgene amplification rate. Co-amplification of transgenes using the dihydrofolate reductase/methotrexate (DHFR/MTX) system is a widely used method for the isolation of Chinese hamster ovary (CHO) cell lines that secrete high levels of recombinant proteins. A bottleneck in this process is the stepwise selection for MTX resistant populations; which can be slow, tedious and erratic. We sought to speed up and regularize this process by isolating dhfr(-) CHO cell lines capable of integrating a transgene of interest into a defined chromosomal location that supports a high rate of gene amplification. We isolated 100 independent transfectants carrying a gene for human adenosine deaminase (ada) linked to a phiC31 attP site and a portion of the dihydrofolate reductase (dhfr) gene. Measurement of the ada amplification rate in each transfectant using Luria-Delbruck fluctuation analysis revealed a wide clonal variation; sub-cloning showed these rates to be heritable. Site directed recombination was used to insert a transgene carrying a reporter gene for secreted embryonic alkaline phosphatase (SEAP) as well as the remainder of the dhfr gene into the attP site at this location in several of these clones. Subsequent selection for gene amplification of the reconstructed dhfr gene in a high ada amplification candidate clone (DG44-HA-4) yielded reproducible rates of seap gene amplification and concomitant increased levels of SEAP secretion. In contrast, random integrations of the dhfr gene into clone HA-4 did not yield these high levels of amplification. This cell line as well as this method of screening for high amplification rates may prove helpful for the reliable amplification of recombinant genes for therapeutically or diagnostically useful proteins.

237 ||| 8649999 ||| (2, 0.97) ||| 2 ||| 2 ||| High-level production of recombinant proteins in CHO cells using a dicistronic DHFR intron expression vector. We have constructed expression vectors for Chinese hamster ovary (CHO) cells that produce both selectable marker and recombinant cDNA from a single primary transcript via differential splicing. These vectors produce stable CHO cell clones that, when pooled, produce abundant amounts of secreted recombinant proteins compared with the amounts produced by conventional expression approaches that have selectable marker and the cDNA of interest under control of separate transcription units. Our vectors divert most of the transcript to product expression while linking it, at a fixed ratio, to dihydrofolate reductase (DHFR) expression to allow selection of stable transfectants. Pools of clones with increased expression of the product gene can be efficiently generated by selection in methotrexate. The high level of expression from pools allows convenient and rapid production of milligram amounts of recombinant proteins.

238 ||| 18602963 ||| (2, 0.95) ||| 2 ||| 2 ||| Short hairpin RNA targeted to dihydrofolate reductase enhances the immunoglobulin G expression in gene-amplified stable Chinese hamster ovary cells. The dihydrofolate reductase (dhfr)/methotrexate (MTX) selection is a common method to conduct gene amplification in stable clones of Chinese hamster ovary (CHO) cells. We previously reported the use of a short hairpin RNA (shRNA) vector targeted to the dhfr gene resulted in improving the intracellular antigen expression in gene-amplified stable CHO cells [Hong, W.W., Wu, S.C., 2007. A novel RNA silencing vector to improve antigen expression and stability in Chinese hamster ovary cells. Vaccine 25 (20), 4103-4111]. Here we investigated the use of the dhfr-targeted shRNA vector for immunoglobulin G (IgG) expression in gene-amplified stable CHO cells. With the use of the dhfr-targeted shRNA vector, the gene-amplified CHO/dhFr(-) cells were found to increase IgG expression at 1.0 microM MTX by more than 100% and to improve the genomic stability of IgG expression in MTX-free cultures by approximately 30%. The use of the dhfr-targeted shRNA vector can enhance the IgG expression in the gene-amplified stable CHO cells and uphold the IgG expression in MTX-free cultures. Utilizing the dhfr-targeted shRNA vector may provide an alternative way to maneuver CHO cell factories for IgG production in cultures.

239 ||| 21538334 ||| (2, 0.94) ||| 2 ||| 2 ||| A mechanistic understanding of production instability in CHO cell lines expressing recombinant monoclonal antibodies. One of the most significant problems in industrial bioprocessing of recombinant proteins using engineered mammalian cells is the phenomenon of cell line instability, where a production cell line suffers a loss of specific productivity (qP). This phenomenon occurs with unpredictable kinetics and has been widely observed in Chinese hamster ovary (CHO) cell lines and with all commonly used gene expression systems. The underlying causes (both genetic and physiological) and the precise molecular mechanisms underpinning cell line instability have yet to be fully elucidated, although recombinant gene silencing and loss of recombinant gene copies have been shown to cause qP loss. In this work we have investigated the molecular mechanisms underpinning qP instability over long-term sub-culture in CHO cell lines producing recombinant IgG1 and IgG2 monoclonal antibodies (Mab's). We demonstrate that production instability derives from two primary mechanisms: (i) epigenetic--methylation-induced transcriptional silencing of the CMV promoter driving Mab gene transcription and (ii) genetic--progressive loss of recombinant Mab gene copies in a proliferating CHO cell population. We suggest that qP decline resulting from loss of recombinant genes is a consequence of the inherent genetic instability of recombinant CHO cell lines.

240 ||| 11834126 ||| (2, 0.94) ||| 2 ||| 2 ||| High-level expression of human thyroid-stimulating hormone in Chinese hamster ovary cells by co-transfection of dicistronic expression vectors followed by a dual-marker amplification strategy. The utilization of dicistronic mRNA expression vectors, containing the gene of interest upstream of an amplifiable marker gene, has shown success in rapidly, efficiently and reproducibly obtaining stable cell lines that express high levels of the protein of interest. For this reason, human thyroid-stimulating hormone (hTSH), a heterodimeric glycoprotein composed of non-covalently linked alpha- and beta-subunits, was expressed in Chinese hamster ovary (CHO) cells using a system based on dicistronic expression vectors. These contained the genes of interest and the amplifiable gene markers dihydrofolate reductase (DHFR) and adenosine deaminase (ADA), separated by an internal ribosome entry site isolated from the encephalomyocarditis virus. After the cells (CHO-DHFR-) had been co-transfected with the expression vectors and submitted to gene amplification in culture medium containing stepwise increments of methotrexate, it was possible to isolate clones that presented a secretion level of up to 7.2+/-1.3 microg/10(6) cells per day, the highest ever reported for the expression of this glycoprotein hormone. A second treatment, involving the utilization of deoxycoformycin, directed to amplify the ADA marker gene, provided a clone with an additional 2-3-fold increase in hTSH secretion, reaching a secretion level of 17.8+/-7.6 microg/10(6) cells per day. Cell culture and hTSH production in a hollow-fibre bioreactor were set up in order to carry out a preliminary physico-chemical, immunological and biological characterization of this hormone in comparison with pituitary-extracted hTSH (from the National Institute of Diabetes and Digestive and Kidney Diseases) and the only recombinant hTSH now available (Thyrogen). The availability of recombinant hTSH is very important in the diagnosis and therapy of thyroid carcinoma, via stimulation of radioiodine uptake.

241 ||| 25451252 ||| (2, 0.92) ||| 2 ||| 2 ||| Optimization protein productivity of human interleukin-2 through codon usage, gene copy number and intracellular tRNA concentration in CHO cells. Transfer RNA (tRNA) abundance is one of the critical factors for the enhancement of protein productivity in prokaryotic and eukaryotic hosts. Gene copy number of tRNA and tRNA codon usage bias are generally used to match tRNA abundance of protein-expressing hosts and to optimize the codons of recombinant proteins. Because sufficient concentration of intracellular tRNA and optimized codons of recombinant proteins enhanced translation efficiency, we hypothesized that sufficient supplement of host's tRNA improved protein productivity in mammalian cells. First, the small tRNA sequencing results of CHO-K1 cells showed moderate positive correlation with gene copy number and codon usage bias. Modification of human interleukin-2 (IL-2) through codons with high gene copy number and high codon usage bias (IL-2 HH, modified on Leu, Thr, Glu) significantly increased protein productivity in CHO-K1 cells. In contrast, modification through codons with relatively high gene copy number and low codon usage bias (IL-2 HL, modified on Ala, Thr, Val), or relatively low gene copy number and low codon usage bias (IL-2 LH, modified on Ala, Thr, Val) did not increase IL-2 productivity significantly. Furthermore, supplement of the alanine tRNA or threonine tRNA increased IL-2 productivity of IL-2 HL. In summary, we revealed a potential strategy to enhance productivity of recombinant proteins, which may be applied in production of protein drug or design of DNA vaccine.

242 ||| 23777349 ||| (2, 0.90) ||| 2 ||| 2 ||| Human ubiquitin C promoter based expression of erythropoietin in CHO K1 cell lines: a simple transfectants screening approach. Erythropoietin (EPO), a glycoprotein hormone that regulates the production of erythrocytes in the human body, is of clinical importance in the treatment of anemia. Low expression levels of this recombinant hormone and time-consuming screening methods have made its commercial production expensive. Cloning of human EPO gene in a shuttle vector pUB6/V5-HisB driven by human ubiquitin C promoter and its transfection in CHO K1 cell lines by electroporation resulted in a moderate level of EPO expression. The limiting-dilution screening method required several months to obtain high expression stable transfectants but needed only short duration for selection in contrast to the present screening strategy. The supernatants of stably transfected cells were found to be biologically active by in vitro erythroid cluster forming activity.

243 ||| 9219237 ||| (2, 0.89) ||| 2 ||| 2 ||| High-level expression of a cDNA for human granulocyte colony-stimulating factor in Chinese hamster ovary cells. Effect of 3'-noncoding sequences. We compared the production of recombinant human granulocyte colony-stimulating factor (rhG-CSF) by Chinese hamster ovary (CHO) cells in a transient expression system, using different analogous vectors carrying a human G-CSF-encoding cDNA under the transcriptional control of the murine cytomegalovirus (CMV) major immediate early promoter. Comparison of two transcription units carrying a human (h)G-CSF cDNA deleted of 3'-untranslated (UTR) sequences containing AT-rich elements (ARE) and using 3'-UTR sequences for processing of transcripts from the SV40 early region or from the rabbit beta 1-globin gene showed that use of the sequences from the rabbit beta 1-globin gene resulted in 7- to 12-fold higher levels of rhG-CSF production. Deletion of ARE of hG-CSF cDNA resulted in increased rhG-CSF synthesis when transcription units using 3'-UTR sequences from the rabbit beta 1-globin gene were compared. By contrast, deletion of ARE did not appear to affect rhG-CSF production when 3'-UTR sequences from the SV40 early region were used. The most efficient G-CSF transcription unit, fused to a dihydrofolate reductase (DHFR) marker gene and transfected into a CHO cell line, yielded initial transfectant CHO cell lines secreting up to 21 micrograms rhG-CSF/1 x 10(6) cells in 24 h. After two rounds of DHFR gene amplification, a cell line was isolated that contains approx 12 copies of the vector and produces rhG-CSF at a rate of 90 micrograms/1 x 10(6) cells in 24 h.

244 ||| 24106171 ||| (2, 0.88) ||| 2 ||| 2 ||| A high-yielding CHO transient system: coexpression of genes encoding EBNA-1 and GS enhances transient protein expression. An efficient rapid protein expression system is crucial to support early drug development. Transient gene expression is an effective route, and to facilitate the use of the same host cells as for subsequent stable cell line development, we have created a high-yielding Chinese hamster ovary (CHO) transient expression system. Suspension-adapted CHO-K1 host cells were engineered to express the gene encoding Epstein-Barr virus (EBV) nuclear antigen-1 (EBNA-1) with and without the coexpression of the gene for glutamine synthetase (GS). Analysis of the transfectants indicated that coexpression of EBNA-1 and GS enhanced transient expression of a recombinant antibody from a plasmid carrying an OriP DNA element compared to EBNA-1-only transfectants. This was confirmed with the retransfection of an EBNA-1-only cell line with a GS gene. The retransfected cell lines showed an increase in transient expression when compared with that of the EBNA-1-only parent. The transient expression process for the best CHO transient cell line was further developed to enhance protein expression and improve scalability by optimizing the transfection conditions and the cell culture process. This resulted in a scalable CHO transient expression system that is capable of expressing 2 g/L of recombinant proteins such as antibodies. This system can now rapidly provide gram amounts of recombinant antibody to supply preclinical development studies that has comparable product quality to antibody produced from a stably transfected CHO cell line.

245 ||| 20371256 ||| (2, 0.88) ||| 2 ||| 2 ||| Generation of stable cell lines by site-specific integration of transgenes into engineered Chinese hamster ovary strains using an FLP-FRT system. Random integration linking genomic amplification is widely used to generate desired cell lines for stable and high-level expressing recombinant proteins. But this technique is laborious, and the expression level is unpredictable due to position effects. After reading many reports on gene amplification, we hypothesized that there should be several loci in the genome of Chinese hamster ovary (CHO) cells that allow not only high-level, but also stable gene expression. Based on this hypothesis, we constructed a plasmid pMCEscan, which introduces a site-specific recombinase-recognition sequence, FRT, for gene targeting into those sites. Another targeting vector, pcDNA5/FRT, has an FRT sequence fused to a promoterless hygromycin-resistance gene that can be expressed only when correct gene targeting occurs. Using the pMCEscan, which is a novel and stringent selection system used to create a few high protein-producing clones, we constructed engineered CHO strains that can be used for high-level production of foreign proteins by gene targeting. We selected 28 CHO strains that expressed a high-level of reporter genes and carried one copy of the pMCEscan in their chromosomes, and we treated these strains with methotrexate (MTX) to evaluate dihydrofolate reductase (DHFR)-mediated gene amplification. Nine clones showed high-level tissue plasminogen activator (tPA) production without amplification. We then targeted other genes (tPA, secreted alkaline phosphatase (SEAP), erythropoietin (EPO)) to test the basal expression ability of nine CHO strains. CHO strains 8-1 and 8-11 consistently expressed high basal levels of the recombinant genes. Using this cell-vector system, we obtained the tPA stable high producers by gene targeting and gene amplification. This system allows for rapid generation of recombinant proteins without cloning and greatly simplifies selection of cell lines for the production of potential therapeutic proteins.

246 ||| 23300841 ||| (2, 0.87) ||| 2 ||| 2 ||| Fusion of the Dhfr/Mtx and IR/MAR gene amplification methods produces a rapid and efficient method for stable recombinant protein production. Amplification of the dihydrofolate reductase gene (Dhfr) by methotrexate (Mtx) exposure is commonly used for recombinant protein expression in Chinese hamster ovary (CHO) cells. However, this method is both time- and labor-intensive, and the high-producing cells that are generated are frequently unstable in culture. Another gene amplification method is based on using a plasmid bearing a mammalian replication initiation region (IR) and a matrix attachment region (MAR), which result in the spontaneous initiation of gene amplification in transfected cells. The IR/MAR and Dhfr/Mtx methods of gene amplification are based on entirely different principles. In this study, we combine these two methods to yield a novel method, termed the IR/MAR-Dhfr fusion method, which was used to express three proteins, the Fc receptor, GFP, and recombinant antibody. The fusion method resulted in a dramatic increase in expression of all three proteins in two CHO sub-lines, DXB-11, and DG44. The IR/MAR-Dhfr fusion amplified the genes rapidly and efficiently, and produced larger amounts of antibody than the Dhfr/Mtx or IR/MAR methods alone. While the amplified structure produced by the Dhfr/Mtx method was highly unstable, and the antibody production rate rapidly decreased with the culture time of the cells, the IR/MAR-Dhfr fusion method resulted in stable amplification and generated clonal cells that produced large amounts of antibody protein over a long period of time. In summary, the novel IR/MAR-Dhfr fusion method enables isolation of stable cells that produce larger amounts of a target recombinant protein more rapidly and easily than either the Dhfr/Mtx or IR/MAR methods alone.

247 ||| 17680677 ||| (2, 0.87) ||| 2 ||| 2 ||| Accelerated cell line development using two-color fluorescence activated cell sorting to select highly expressing antibody-producing clones. The success of engineered monoclonal antibodies as biopharmaceuticals has generated considerable interest in strategies designed to accelerate development of antibody expressing cell lines. Stable mammalian cell lines that express therapeutic antibodies at high levels typically take 6-12 months to develop. Here we describe a novel method to accelerate selection of cells expressing recombinant proteins (e.g., antibodies) using multiparameter fluorescence activated cell sorting (FACS) in association with dual intracellular autofluorescent reporter proteins. The method is co-factor-independent and does not require complex sample preparation. Chinese hamster ovary (CHO) clones expressing high levels of recombinant antibody were selected on the basis of a two-color FACS sorting strategy using heavy and light chain-specific fluorescent reporter proteins. We were able to establish within 12 weeks of transfection cell lines with greater than a 38-fold increase in antibody production when compared to the pool from which they were isolated, following a single round of FACS. The method provides a robust strategy to accelerate selection and characterization of clones and builds a foundation for a predictive model of specific productivity based upon on two-color fluorescence.

248 ||| 9779420 ||| (2, 0.86) ||| 2 ||| 2 ||| Rapid establishment of high-producing cell lines using dicistronic vectors with glutamine synthetase as the selection marker. Recombinant proteins are useful tools in biological research, drug development, and drug screening. Specially designed expression vectors have been developed to introduce cDNA for recombinant protein expression in mammalian cells. We have combined a dicistronic mRNA design for expression of the recombinant protein, using glutamine synthetase (GS) for selection. A soluble form of human interleukin-4 receptor alpha chain was used as the model protein. The dicistronic vectors were compared to a standard expression vector in CHO-K1 cells in parallel experiments. Our data showed that a dicistronic vector containing an internal ribosome entry site (IRES) of the encephalomyocarditis virus (ECMV) was superior to a conventional expression vector in both levels of protein expression and amplification efficiency. The productivity of these clones was stable without selection pressure for an extended period of time. The GS selection system within a dicistronic vector design can achieve rapid and efficient gene amplification for protein production.

249 ||| 22623341 ||| (2, 0.86) ||| 2 ||| 2 ||| Albumin 3'untranslated region facilitates increased recombinant protein production from Chinese hamster ovary cells. Chinese hamster ovary (CHO) cells are used for recombinant protein production in the pharmaceutical industry but there is a need to improve expression levels. In the present work experiments were carried out to test the effectiveness of different 3'untranslated regions (3'UTRs) in promoting production of a naturally secreted luciferase. Seamless cloning was used to produce expression vectors in which Gaussia princeps luciferase coding sequences were linked to the human albumin, immunoglobulin or chymotrypsinogen 3'UTR. Stably transfected CHO cells expressing these constructs were selected. Luciferase activity in the culture medium was increased 2-3-fold by replacing the endogenous 3'UTR with the albumin 3'UTR and decreased by replacement with immunoglobulin or chymotrypsinogen 3'UTR. Replacement of the native 3'UTR with the albumin 3'UTR led to a 10-fold increase in luciferase mRNA levels. Deletion analysis of the albumin 3'UTR showed that loss of nucleotides 1-50, which removed an AU-rich complex stem loop region, caused significant reductions in both luciferase protein expression and luciferase mRNA levels. The results suggest that recombinant protein expression and yield could be improved by the careful selection of appropriate 3'UTR sequences.

250 ||| 20430058 ||| (2, 0.86) ||| 2 ||| 2 ||| DNA methylation contributes to loss in productivity of monoclonal antibody-producing CHO cell lines. Production instability currently limits the use of mammalian cells for industrial production of therapeutic proteins. We have previously reported that the loss of productivity in recombinant monoclonal antibody producing Chinese Hamster Ovary (CHO-mAb) cell lines is mainly due to a decrease in heavy chain (HC) and light chain (LC) transcripts. Molecular analysis indicates that the decreased mRNA levels are not due to a loss in gene copies and change of integration sites. In this work, we further demonstrate that impaired trans-acting factors and spontaneous mutations to the DNA are not responsible for the reduced HC and LC transcription. Examination of two CpG sites by methyl-assisted quantitative real-time PCR assay revealed an increase in methylation of the human cytomegalovirus major immediate-early enhancer and promoter (hCMV-MIE) controlling the expression of LC and HC in cells which exhibited loss in productivity. Treatment of these cells with a DNA methylation inhibitor, 5-aza-2'-deoxycytidine, partially restored the lost specific mAb productivity. The increase in productivity correlated to the increase in mRNA levels of HC and LC and the demethylation of hCMV-MIE promoter. This finding, which indicates that DNA methylation contributes to production instability, will be beneficial for generation of high-producing cell lines with stable productivity.

251 ||| 7599190 ||| (2, 0.85) ||| 2 ||| 2 ||| Cloning and characterization of the 5'-flanking region of metallothionein-I gene from Chinese hamster ovary cells. The 5'-flanking region of the Chinese hamster ovary metallothionein-I gene (MT-I) was isolated and sequenced. This region contains several cis-regulatory sequences, including metal-responsive element (MRE), AP1-binding site, Sp1-binding site and glucocorticoid-responsive element (GRE). These elements are located within 250 bases upstream of the transcription initiation site, as determined by primer extension. This region was fused with a reporter gene that could be activated by the challenge of cadmium only when the MREs were present. Thus, the isolated DNA is the promoter region of the CHO MT-I gene.

252 ||| 24929670 ||| (2, 0.84) ||| 2 ||| 2 ||| Improved elongation factor-1 alpha-based vectors for stable high-level expression of heterologous proteins in Chinese hamster ovary cells. BACKGROUND: Establishing highly productive clonal cell lines with constant productivity over 2-3 months of continuous culture remains a tedious task requiring the screening of tens of thousands of clonal colonies. In addition, long-term cultivation of many candidate lines derived in the absence of drug selection pressure is necessary. Expression vectors based on the elongation factor-1 alpha (EEF1A) gene and the dihydrofolate reductase (DHFR) selection marker (with separate promoters) can be used to obtain highly productive populations of stably transfected cells in the selection medium, but they have not been tested for their ability to support target gene amplification under gradually increasing methotrexate pressure. RESULTS: We have modified EEF1A-based vectors by linking the DHFR selection marker to the target gene in the bicistronic RNA, shortening the overall plasmid size, and adding an Epstein-Barr virus terminal repeat fragment (EBVTR) element. Presence of the EBVTR element increased the rate of stable transfection by the plasmid by 24 times that of the EBVTR-minus control and improved the rate of methotrexate-driven gene amplification. The mean expression level of the enhanced green fluorescent protein (eGFP) used herein as a model protein, increased up to eight-fold using a single round of amplification in the case of adherent colonies formation and up to 4.5-fold in the case of suspension polyclonal cultures. Several eGFP-expressing cell populations produced using vectors with antibiotic resistance markers instead of the DHFR marker were compared with each other. Stable transfection of Chinese hamster ovary (CHO) DG44 cells by the p1.2-Hygro-eGFP plasmid (containing a hygromycin resistance marker) generated highest eGFP expression levels of up to 8.9% of the total cytoplasmic protein, with less than 5% of the cell population being eGFP-negative. CONCLUSIONS: The p1.1 vector was very effective for stable transfection of CHO cells and capable of rapid MTX-driven target gene amplification, while p1.2-Hygro achieved similar eGFP expression levels as p1.1. The set of vectors we have developed should speed-up the process of generating highly productive clonal cell lines while substantially decreasing the associated experimental effort.

253 ||| 24248275 ||| (2, 0.84) ||| 2 ||| 2 ||| Overexpression of mutant cell division cycle 25 homolog B (CDC25B) enhances the efficiency of selection in Chinese hamster ovary cells. The effects of mutant cell division cycle 25 homolog B (CDC25B) overexpression on the generation of cells producing a monoclonal antibody were investigated in Chinese hamster ovary (CHO) cells. Mutant CDC25B (m-CDC25B) expression plasmids were transfected into CHO DG44-derived cells producing a monoclonal antibody, and the frequency of highly producing cells was assessed following gene amplification in the presence of 250 nM methotrexate. Most of the clones obtained from the m-CDC25B-overexpressing cells had higher antibody titers than did mock-transfected control cells. This arose from either higher transgene copy numbers or higher mRNA expression levels for the antibody. However, the high mRNA expression levels were not always accompanied by increases in transgene copy numbers. Our results suggest that cells producing high levels of a monoclonal antibody can be selected efficiently using m-CDC25B overexpression.

254 ||| 17269689 ||| (2, 0.82) ||| 2 ||| 2 ||| High-level recombinant protein production in CHO cells using an adenoviral vector and the cumate gene-switch. To facilitate and accelerate the production of eukaryotic proteins with correct post-translational modifications, we have developed a protein production system based on the transduction of Chinese hamster ovary (CHO) cells using adenovirus vectors (AdVs). We have engineered a CHO cell line (CHO-cTA) that stably expresses the transactivator (cTA) of our newly developed cumate gene-switch transcription system. This cell line is adapted to suspension culture and can grow in serum-free and protein-free medium. To increase the transduction level of AdVs, we have also generated a cell line (CHO-cTA-CAR) that expresses additional amounts of the coxackievirus and adenovirus receptor (CAR) on its surface. Recombinant protein production was tested using an AdV carrying the secreted alkaline phosphatase (SEAP) under the control of the CR5 promoter, which is strongly and specifically activated by binding to cTA. The SEAP expression was linked to the expression of the green fluorescent protein (GFP) through an internal ribosome entry site (IRES) to facilitate titration of the AdV. We monitored SEAP expression on a daily basis for 9 days after transduction of CHO-cTA and CHO-cTA-CAR using different quantities of AdVs at 37 and 30 degrees C. Incubation at the latter temperature increased the production of SEAP at least 10-fold, and the presence of CAR increased the transduction level of the AdV. Maximum SEAP production (63 mg/L) was achieved at 6-7 days post-infection at 30 degrees C by transducing CHO-cTA-CAR with 500 infectious particles/cell. Because numerous AdVs can now be generated within a few weeks and large-scale production of AdVs is now a routine procedure, this system could be used to produce rapidly milligram quantities of a battery of recombinant proteins as well as for large-scale protein production.

255 ||| 24140057 ||| (2, 0.81) ||| 2 ||| 2 ||| A rapid screening and production method using a novel mammalian cell display to isolate human monoclonal antibodies. Antibody display methods are increasingly being used to produce human monoclonal antibodies for disease therapy. Rapid screening and isolation of specific human antibody genes are valuable for producing human monoclonal antibodies showing high specificity and affinity. In this report, we describe a novel mammalian cell display method in which whole human IgG is displayed on the cell surface of CHO cells. Cells expressing antigen-specific human monoclonal IgGs with high affinity on the cell surface after normal folding and posttranscriptional modification were screened using a cell sorter. The membrane-type IgG-expressing CHO cells were then converted to IgG-secreting cells by transfection with a plasmid coding Cre recombinase. This mammalian cell display method was applied to in vitro affinity maturation of monoclonal C9 IgG specific to the human high-affinity IgE receptor (FcepsilonRIalpha). The CDR3 of the C9 heavy chain variable region gene was randomly mutated and inserted into pcDNA5FRT/IgG. A C9 IgG (CDRH3r)-expressing CHO cell display library consisting of 1.1x10(6) independent clones was constructed. IgG-displaying cells showing high reactivity to FcepsilonRIalpha antigen were screened by the cell sorter, resulting in the establishment of a CHO cell line producing with higher reactivity than the parent C9 IgG.

256 ||| 23047106 ||| (2, 0.81) ||| 2 ||| 2 ||| Establishment of tetracycline-inducible, survivin-expressing CHO cell lines by an optimized screening method. An optimized method based on tetracycline-inducible gene expression system T-REx was developed to screen and evaluate Tet repressor (TetR)-expressing cell lines using enhanced green fluorescence protein (EGFP) as reporter gene. To verify the effectiveness of the method, two TetR-expressing Chinese hamster ovary (CHO) cell lines, CHO-TR B2 (stringent) and B5 (less stringent), in which the EGFP genes were variantly controlled by tetracycline, were used to construct cell lines expressing the anti-apoptosis gene survivin upon induction with tetracycline. The resulting stable clones were analyzed for survivin expression. The analysis showed that all four B5-derived clones exhibited leaky survivin expression in the absence of tetracycline, while the B2-derived clones did not. DNA laddering and annexin V/PI staining assays further indicated that although tetracycline-inducible expression of survivin conferred resistance to NH4Cl- and staurosporine-induced apoptosis in both the B2- and the B5-derived stable cell lines, the B2-derived cell lines showed more stringent regulation in the absence of tetracycline. This represents successful utilization of the present screening method.

257 ||| 20300883 ||| (2, 0.80) ||| 2 ||| 2 ||| Utilization of site-specific recombination for generating therapeutic protein producing cell lines. The AttSite Recombinase Technology from Intrexon, Blacksburg, VA, utilizes specific DNA sequences and proprietary recombinase enzymes to catalyze the insertion of a gene of interest at a specific location in the host cell genome. Using this technology, we have developed Chinese Hamster Ovary (CHO) cell lines that have incorporated attB recombination sites at highly transcriptionally active loci or 'hot spots' within the cell genome. Subsequently, these attB site containing host cell lines could then be used for the expression of future Centocor products. Candidate production cell lines would be generated by a simple recombination event. Since the therapeutic gene of interest would preferentially integrate into the pre-selected high-expressing attB site, candidate cell lines would consistently express high levels of the gene of interest. We have been able to demonstrate that the AttSite Recombinase Technology could be a valid approach for the development of high-expressing production cell lines.

258 ||| 14524703 ||| (2, 0.80) ||| 2 ||| 2 ||| EASE vectors for rapid stable expression of recombinant antibodies. Over the past 10 years, monoclonal antibodies and antibody fragments have become an increasingly important source of therapeutic molecules in the biotechnology industry. Drug development strategies rely on screening large numbers of candidate molecules in search of an optimized drug candidate. This strategy requires efficient production of ten to a few hundred milligrams of candidate molecules for screening in bioassays and animal models. Typically, this amount of recombinant protein expression involves large numbers of transient transfections or cloning of a recombinant cell line. Both of these approaches are time-consuming and labor-intensive. In this report, we describe the application of an EASE vector system that is capable of generating stable pools of transfected Chinese hamster ovary cells. These pooled populations of cells produce high quantities of antibody candidates without labor-intensive cloning in a 3-5 week time frame. When an optimal drug candidate has been selected, pools generated with EASE-containing vectors can also be used in subsequent cloning steps to make cell lines with improved expression levels. We demonstrate that EASE increases expression in nonamplified pools in addition to increasing amplification and viability of clonal cell lines generated with the EASE-containing vectors compared with pools and cell lines generated without EASE.

259 ||| 11001873 ||| (2, 0.80) ||| 2 ||| 2 ||| High-level synthesis of human prolactin in Chinese-Hamster ovary cells. Two eukaryotic human prolactin (hPRL) expression vectors, based on a selectable dihydrofolate reductase (dhfr) marker, were used to transfect dhfr(-) Chinese- hamster ovary (CHO) cells. One vector, p658-hPRL, contains the hepatitis-B virus-X cDNA coding for a viral transactivator and sequences mediating dhfr mRNA degradation. The other, pEDdc-hPRL, carries the encephalomyocarditis virus leader sequence coupled to hPRL cDNA to provide high-level protein expression, possibly via a mechanism of internal translation initiation in dicistronic mRNA. Without methotrexate (MTX) amplification, p658-hPRL-transfected stable cell lines, secreting up to approximately 10 microg of hPRL/10(6) cells per day, could be rapidly obtained; production by pEDdc-hPRL-transfected cells was about 10-fold lower. However, a three-step MTX amplification of the latter led to clones secreting up to approximately 30 microg of hPRL/10(6) cells per day. A pilot production using a hollow-fibre bioreactor indicated that highly concentrated hormone levels in the medium could be obtained, with a production of up to 150 microg of hPRL/ml per day. SDS/PAGE analysis indicated that recombinant hPRL contained approximately 10% glycosylated PRL. Chromatographically purified non-glycosylated and glycosylated recombinant hPRL had bioactivities of 35 and 16 i.u./mg, respectively (Nb2 cell bioassay). This appears to be the first report describing production and purification of recombinant hPRL from CHO cells, secreted at levels higher than reported thus far in eukaryotic systems.

260 ||| 16766074 ||| (2, 0.79) ||| 2 ||| 2 ||| A rapid protein expression and purification system using Chinese hamster ovary cells expressing retrovirus receptor. Conventional stable protein expression systems using mammalian cells include a time-consuming step of antibiotic resistance-based cell cloning. Here, we report a rapid flow cytometry-based method for the collection of retrovirus vector-infected Chinese hamster ovary (CHO) cells that express desired proteins. The vector carries the genes for green fluorescent protein (GFP), as a marker, and glutathione-S-transferase (GST), to express the desired protein as a GST-fusion construct. To render CHO cells susceptible to retrovirus infection, they were forced to express EcoR, the receptor for retroviruses. After infection, cells expressing desired proteins were collected by flow cytometry as a GFP-positive population, and the desired proteins were purified by glutathione affinity chromatography. This method reduces the time required between infection of cells and purification of a desired protein from several months to approximately 2 weeks.

261 ||| 19002809 ||| (2, 0.78) ||| 2 ||| 2 ||| Evaluation of stable and highly productive gene amplified CHO cell line based on the location of amplified genes. In order to establish an easy and quick construction method for obtaining a stable and highly productive gene-amplified recombinant Chinese Hamster Ovary (CHO) cell line, variouskinds of stepwise methotrexate (MTX) selection were carriedout. The specific growth and production rates of the cell were compared with each other, and the distribution of the amplified gene location was determined using fluorescence in situ hybridization (FISH). The specific growth andproduction rates of the cell pool reached the highest levels under the selection condition in which the stepwise increase in the MTX concentration was most gradual; about 82% of amplified genes were observed near the telomeric region. During long-term cultivation without MTX, the percentage ofamplified genes near the telomeric region hardly changed, butthat of amplified genes at other regions decreased. Based on these results, stable and highly productive cell pools could be easily and quickly constructed and amplified and gradual stepwise increase of the MTX concentration. In addition, the FISH technique was powerful tool to evaluate highly productiveand stable gene-amplified cells based on the chromosomal location of the amplified gene.

262 ||| 25307181 ||| (2, 0.77) ||| 2 ||| 2 ||| Impact of using different promoters and matrix attachment regions on recombinant protein expression level and stability in stably transfected CHO cells. High expression level and long-term expression stability are required for therapeutic protein production in mammalian cells. Three commonly used promoters from the simian virus 40 (SV40), the CHO elongation factor 1alpha gene (EF1alpha), and the human cytomegalovirus major immediate early gene (CMV) and two matrix attachment regions from the chicken lysozyme gene (cMAR) and the human interferon beta (iMAR) were evaluated for enhancing recombinant gene expression level and stability in stably transfected CHO cells. In the absence of MAR elements, the SV40 promoter gave lower expression level but higher stability than the EF1alpha promoter and the CMV promoter. The inclusion of MAR elements did not increase the integrated gene copies for all promoters but did enhance expression level for only the SV40 promoter. The enhanced gene expression was due to an increase in mRNA levels. Neither MAR elements enhance gene expression stability during long-term culture. The combinations of SV40 promoter and MAR elements are the best for obtaining both high expression level and stability. The information presented here would be valuable to those developing vectors for generation of CHO cell lines with stable and high productivity.

263 ||| 19845036 ||| (2, 0.77) ||| 2 ||| 2 ||| Transient expression of human TorsinA enhances secretion of two functionally distinct proteins in cultured Chinese hamster ovary (CHO) cells. Cultured mammalian cells, particularly Chinese hamster ovary (CHO) cells, are widely exploited as hosts for the production of recombinant proteins, but often yields are limiting. Such limitations may be due in part to the misfolding and subsequent degradation of the heterologous proteins. Consequently we have determined whether transiently co-expressing yeast and/or mammalian chaperones that act to disaggregate proteins, in CHO cell lines, improve the levels of either a cytoplasmic (Fluc) or secreted (Gluc) form of luciferase or an immunoglobulin IgG4 molecule. Over-expression of the yeast 'protein disaggregase' Hsp104 in a CHO cell line increased the levels of Fluc more significantly than for Gluc although levels were not further elevated by over-expression of the yeast or mammalian Hsp70/40 chaperones. Over-expression of TorsinA, a mammalian protein related in sequence to yeast Hsp104, but located in the ER, significantly increased the level of secreted Gluc from CHO cells by 2.5-fold and to a lesser extent the secreted levels of a recombinant IgG4 molecule. These observations indicate that the over-expression of yeast Hsp104 in mammalian cells can improve recombinant protein yield and that over-expression of TorsinA in the ER can promote secretion of heterologous proteins from mammalian cells.

264 ||| 18078289 ||| (2, 0.77) ||| 2 ||| 2 ||| Improved production of recombinant human antithrombin III in Chinese hamster ovary cells by ATF4 overexpression. To improve the production of recombinant human antithrombin III (AT-III) in Chinese hamster ovary (CHO) cells, the genes encoding transcription factors, ATF4 (activating transcription factor 4) and XBP-1s (the spliced form of X-box binding protein 1), which were involved in the mammalian unfolded protein response (UPR), were cloned from CHO-K1 cells. Overexpression of ATF4 significantly enhanced the production of recombinant AT-III in CHO 13D-35D cells. The specific rate of AT-III production in the ATF4-overexpressed CHO 13D-35D cells reached approximately 23 pg/cell/day. After 144 h of incubation, the AT-III concentration in the culture supernatant was twofold greater compared to that observed with parental CHO 13D-35D cells. In contrast, ectopic expression of XBP-1s failed to enhance the production of recombinant AT-III in CHO 13D-35D cells. RT-PCR analysis revealed that high levels of XBP-1s mRNA were present in the CHO cells, regardless of ectopic expression of XBP-1s. Our results indicate that overexpression of the UPR transcription factor ATF4 is a promising means for improving the production of secreted protein pharmaceuticals in CHO cells.

265 ||| 17172660 ||| (2, 0.77) ||| 2 ||| 2 ||| Toward more efficient protein expression: keep the message simple. Optimization of gene coding-sequence, including preferred codon usage and removal of cryptic splice sites and mRNA-destabilizing motifs, has been shown to improve recombinant protein production of different proteins. Here, we present data to show that gene optimization can also be used to improve the production of a complex macromolecule, namely an antibody. When applied to the heavy and light chain genes of our model antibody, we found that greater numbers of high-producing transfectants as well as increased levels of protein production were observed (approximately 1.5-fold). In this test model, production was improved even though the antibody has previously been demonstrated to give high expression in stably transfected cells (up to 5 g/L in bioreactors). Because the parental heavy chain sequence contained introns, and the process of gene optimization is most efficiently performed on sequences without introns, we demonstrated that removal of introns in the coding sequence had no effect on the quantity of antibody produced. All constructs were evaluated using Lonza's glutamine synthetase gene expression vectors in Chinese hamster ovary cells. Our findings suggest that significant improvements in product yields can be achieved by gene optimization, which may facilitate the processing and translation of gene transcripts.

266 ||| 24725707 ||| (2, 0.76) ||| 2 ||| 2 ||| Optimization of heterologous protein production in Chinese hamster ovary cells under overexpression of spliced form of human X-box binding protein. BACKGROUND: The optimization of protein production is a complex and challenging problem in biotechnology. Different techniques for transcription, translation engineering and the optimization of cell culture conditions have been used to improve protein secretion, but there remain many open problems involving post-translational modifications of the secreted protein and cell line stability. RESULTS: In this work, we focus on the regulation of secreted protein specific productivity (using a recombinant human immunoglobulin G (IgG)) by controlling the expression of the spliced form of human X-box binding protein (XBP-(s)) in Chinese hamster ovary cells (CHO-K1) under doxycycline (DOX) induction at different temperatures. We observed a four-fold increase in specific IgG productivity by CHO cells under elevated concentrations of DOX at 30 C compared to 37 C, without detectable differences in binding activity in vitro or changes in the structural integrity of IgG. In addition, we found a correlation between the overexpression of human XBP-1(s) (and, as a consequence, endoplasmic reticulum (ER) size expansion) and the specific IgG productivity under DOX induction. CONCLUSIONS: Our data suggest the T-REx system overexpressing human XBP-1(s) can be successfully used in CHO-K1 cells for human immunoglobulin production.

267 ||| 23727793 ||| (2, 0.76) ||| 2 ||| 2 ||| Rapid establishment of CHO cell lines producing the anti-hepatocyte growth factor antibody SFN68. Anti-hepatocyte growth factor (anti-HGF) monoclonal antibodies (mAbs) are potential therapeutics against various cancers. Screening for high-producer clones is a time-consuming and complex process and is a major hurdle in the development of therapeutic mAbs. Here, we describe an efficient approach that allows the selection of high-producer Chinese hamster ovary (CHO) cell lines producing the novel anti-HGF mAb SFN68, which was generated previously by immunizing HGF bound to its receptor c-Met. We selected an SFN68-producing parental cell line via transfection of the dihydrofolate reductase-deficient CHO cell line DG44, which was preadapted to serum-free suspension culture, with an SFN68-expression vector. Subsequent gene amplification via multiple passages of the parental cell line in a methotrexate-containing medium over 4 weeks, followed by clonal isolation, enabled us to isolate two cell lines, 2F7 and 2H4, with 3-fold higher specific productivity. We also screened 72 different media formulated with diverse feed and basal media to develop a suboptimized medium. In the established suboptimized medium, the highest anti-HGF mAb yields of the 2F7 and 2H4 clones were 842 and 861 mg/l, respectively, which were about 10.5-fold higher than that of the parental cell line in a non-optimized basal medium. The selected CHO cell lines secreting high titers of SFN68 would be useful for the production of sufficient amounts of antibodies for efficacy evaluation in preclinical and early clinical studies.

268 ||| 10417224 ||| (2, 0.76) ||| 2 ||| 2 ||| Cytogenetic analysis of chimeric antibody-producing CHO cells in the course of dihydrofolate reductase-mediated gene amplification and their stability in the absence of selective pressure. Previously, the highest producing (HP) recombinant CHO subclones isolated at various methotrexate (MTX) levels showed different antibody production stability during long-term culture, although they were clonally derived from CS13 transformant. In this study, genetic basis for their difference in antibody production stability was investigated using southern blot hybridization and fluorescence in situ hybridization (FISH) techniques. Southern analysis of HP subclones revealed that light-chain (LC) and heavy-chain (HC) cDNAs were located closely within 23 kb on an amplification unit, and the configuration of LC and HC cDNAs within this amplification unit was not disrupted during long-term culture in the absence of MTX. However, when LC and HC genes were localized on the metaphase chromosomes of HP subclones using FISH, the amplified sequences were present as an extended array on diverse marker chromosomes. HP subclones selected at higher MTX level had more kinds of marker chromosomes. CS13\*-002 isolated at 0.02 microM MTX had only one marker chromosome (m002), whereas CS13\*-1.0 isolated at 1 microM MTX had five different ones (m10A, m10B, m10C, m10D, and m10E). Each marker chromosome showed different fate during long-term culture of HP subclones in the absence of MTX, resulting in different degrees of stability among the HP subclones. The m10A and m10B remained unchanged, whereas the others disappeared or evolved to variants with shortened amplified arrays. The cells containing stable marker chromosomes constituted dominant subpopulations in CS13\*-1.0, and thereby CS13\*-1.0 became most stable in regard to antibody production during long-term culture. Furthermore, our dual-color FISH showed that the telomeric ends of amplified arrays on the stable marker chromosomes were always surrounded by (TTAGGG)(n) sequences, indicating that (TTAGGG)(n) sequences are closely related to the stability and evolution of amplified sequences. Taken together, our data show that the assessment of genotypic stability of amplified CHO cells is a prerequisite for understanding their production stability during long-term culture in the absence of selection pressure.

269 ||| 22422519 ||| (2, 0.75) ||| 2 ||| 2 ||| Role of non-specific DNA in reducing coding DNA requirement for transient gene expression with CHO and HEK-293E cells. Transient gene expression (TGE) is a rapid method for the production of recombinant proteins in mammalian cells. While the TGE volumetric productivity has improved significantly over the past decade, the amount of plasmid DNA (pDNA) needed for transfection remains very high. Here, we examined the use of non-specific (filler) DNA to partially replace the transgene-bearing plasmid DNA (coding pDNA) in transfections of Chinese hamster ovary (CHO) and human embryo kidney (HEK-293E) cells. When the optimal amount of coding pDNA for either host was reduced by 67% and replaced with filler DNA, the recombinant protein yield decreased by only 25% relative to the yield in control transfections. Filler DNA did not affect the cellular uptake or intracellular stability of coding pDNA, but its presence lead to increases of the percentage of transfected cells and the steady-state level of transgene mRNA compared to control transfections. Studies of the physicochemical properties of DNA-polyethyleneimine (PEI) complexes with or without filler DNA did not reveal any differences in their size or surface charge. The results suggest that filler DNA allows the coding pDNA to be distributed over a greater number of DNA-PEI complexes, leading to a higher percentage of transfected cells. The co-assembly of filler DNA and coding pDNA within complexes may also allow the latter to be more efficiently utilized by the cell's transcription machinery, resulting in a higher level of transgene mRNA.

270 ||| 23676904 ||| (2, 0.74) ||| 2 ||| 2 ||| Engineering the cellular protein secretory pathway for enhancement of recombinant tissue plasminogen activator expression in Chinese hamster ovary cells: effects of CERT and XBP1s genes. Cell line development is the most critical and also the most time-consuming step in the production of recombinant therapeutic proteins. In this regard, a variety of vector and cell engineering strategies have been developed for generating high-producing mammalian cells; however, the cell line engineering approach seems to show various results on different recombinant protein producer cells. In order to improve the secretory capacity of a recombinant tissue plasminogen activator (t-PA)-producing Chinese hamster ovary (CHO) cell line, we developed cell line engineering approaches based on the ceramide transfer protein (CERT) and X-box binding protein 1 (XBP1) genes. For this purpose, CERT S132A, a mutant form of CERT that is resistant to phosphorylation, and XBP1s were overexpressed in a recombinant t-PA-producing CHO cell line. Overexpression of CERT S132A increased the specific productivity of t-PA-producing CHO cells up to 35%. In contrast, the heterologous expression of XBP1s did not affect the t-PA expression rate. Our results suggest that CERTS132A- based secretion engineering could be an effective strategy for enhancing recombinant t- PA production in CHO cells.

271 ||| 23124363 ||| (2, 0.74) ||| 2 ||| 2 ||| Optimized signal peptides for the development of high expressing CHO cell lines. Recombinant biotherapeutic proteins such as monoclonal antibodies are mostly produced in Chinese hamster ovary (CHO) cells and pharmaceutical companies are interested in an appropriate platform technology for the development of large-scale production processes. A major aim of our study was therefore to improve the secretion efficiency of a recombinant biotherapeutic antibody by optimizing signal peptides. Reporter molecules such as gaussia and vargula luciferase or secreted alkaline phosphatase are frequently used to this end. In striking contrast, we used a biotherapeutic antibody that was fused to 16 different signal peptides during our study. In this way, the secretion efficiency of the recombinant antibody has been analyzed by transient expression experiments in CHO cell lines. Compared to the control signal peptide, it was not possible to achieve higher efficiencies with signal peptides derived from a variety of species or even natural immunoglobulin G signal peptides. The best results were obtained with natural signal peptides derived from human albumin and human azurocidin. These results were confirmed by fed-batch experiments with stably transfected cell pools, in which cell-specific productivities up to 90 pg cell(-1) day(-1) and product concentrations up to 4 g L(-1) could be determined using the albumin signal peptide. Finally, the applicability of the identified signal peptides for both different antibodies and non-antibody products was demonstrated by transient expression experiments. In conclusion, it was found that signal peptides derived from human albumin and human azurocidin are most appropriate to generate cell lines with clearly improved production rates suitable for commercial purposes in a product-independent manner.

272 ||| 20222103 ||| (2, 0.74) ||| 2 ||| 2 ||| mRNA stability and antibody production in CHO cells: improvement through gene optimization. The productivity of stably transfected cell lines is of critical importance for the manufacturing of therapeutic proteins. Various methods have been successfully implemented to increase the production output of mammalian cell cultures. Increasing evidence suggests that optimization of the gene coding sequences of an expression vector can improve specific cell line yield of the recombinant protein. Here we demonstrate that gene optimization substantially enhances antibody production in Chinese hamster ovary cells. When gene optimization was applied to the heavy and light chain genes of a therapeutic antibody, we observed increased antibody production in transient transfection. Elevated heavy chain mRNA level was associated with the increase of antibody production. Further analysis suggested that the increased antibody expression is attributable to enhanced mRNA stability resulting from gene optimization. Gene optimization also led to increased antibody production in stable clones.

273 ||| 11115388 ||| (2, 0.74) ||| 2 ||| 2 ||| Efficiency of promoter and cell line in high-level expression of erythropoietin. Efficiency of viral promoters and various cell lines in directing high-level expression of human erythropoietin (Epo) was investigated. To investigate the effects of various viral promoters and cell lines on the Epo expression level, genomic Epo with the 5' and 3' untranslated regions (UTRs) deleted was cloned next to the simian virus 40 early promoter, cytomegalovirus early promoter or SRalpha promoter. These expression vectors were transfected into COS-7, BHK-21 and Chinese hamster ovary (CHO)/dhfr(-) cells, respectively. The COS-7 cells transfected with the vector containing the SRalpha promoter showed the highest expression level ( approximately 103 IU/ml) at 72 h post-transfection. For the development of Epo-producing stable cell lines, BHK-21 and CHO/dhfr(-) cells transfected with the 5',3'-UTR-deleted genomic Epo under the control of the SRalpha promoter were cultured with media containing zeocin. Several clones of zeocin-resistant BHK-21 and CHO/dhfr(-) cells were cultured in the presence of methotrexate (MTX). A BHK-21 clone selected in the presence of 500 nM MTX expressed and secreted approximately 490 IU/ml Epo into the medium. A CHO/dhfr(-) clone selected in the presence of 20 nM MTX expressed and secreted approximately 45 IU/ml Epo into the medium. Southern-blot analysis indicated that enhancement of Epo expression in the MTX-resistant stable cells might be related to the amplification of gene copy number.

274 ||| 25820359 ||| (2, 0.73) ||| 2 ||| 2 ||| Enhanced expression of bioactive recombinant VEGF-111 with insertion of intronic sequence in mammalian cell lines. Among all VEGF-A isoforms, VEGF-111 is particularly important in molecular biology research owing to its potent angiogenic properties and its remarkable resistance to proteolysis. These features make it a potential candidate for therapeutic use in ischemic diseases. VEGF-111 is not expressed in normal cells, but expression is induced by UV-B irradiation and exposure to genotoxic agents. Here, to increase expression at the transcriptional and translational levels, we synthesized and cloned recombinant VEGF-111 cDNA. Two fragments encoding exons 1-4 and intron 4/5 plus exon 8a were amplified and cloned into the pBud.CE4.1 vector using a class IIs restriction enzyme-based method. The expression of VEGF-111 in CHO-dhfr - and HEK 293 cell lines was evaluated with real-time PCR, dot blotting, and ELISA. VEGF expression was increased about 10- and 18-fold in transfected CHO-dhfr - and HEK 293 cells, respectively. Dot blotting and ELISA confirmed successful production of VEGF-111 in both cell lines.

275 ||| 23704898 ||| (2, 0.73) ||| 2 ||| 2 ||| Comparison of internal ribosome entry site (IRES) and Furin-2A (F2A) for monoclonal antibody expression level and quality in CHO cells. Four versions of tricistronic vectors expressing IgG1 light chain (LC), IgG1 heavy chain (HC), and dihydrofolate reductase (DHFR) in one transcript were designed to compare internal ribosome entry site (IRES) and furin-2A (F2A) for their influence on monoclonal antibody (mAb) expression level and quality in CHO DG44 cells. LC and HC genes are arranged as either the first or the second cistron. When using mAb quantification methods based on the detection antibodies against HC Fc region, F2A-mediated tricistronic vectors appeared to express mAb at higher levels than the IRES-mediated tricistronic vectors in both transient and stable transfections. Further analysis revealed that more than 40% of products detected in stably transfected pools generated using the two F2A-mediated tricistronic vectors were aggregates. LC and HC from the F2A stably transfected pools were not properly processed, giving rise to LC+F2A+HC or HC+F2A+LC fusion proteins, LC and HC polypeptides with F2A remnants, and incorrectly cleaved signal peptides. Both IRES-mediated tricistronic vectors express mAb with correct sizes and signal peptide cleavage. Arrangement of LC as the first cistron in the IRES-mediated tricistronic vectors exhibits increased mAb expression level, better growth, and minimized product aggregation, while arrangement of HC as first cistron results in low expression, slower growth, and high aggregation. The results obtained will be beneficial for designing vectors that enhance mAb expression level and quality in mammalian cells.

276 ||| 17614336 ||| (2, 0.73) ||| 2 ||| 2 ||| Effects of overexpression of X-box binding protein 1 on recombinant protein production in Chinese hamster ovary and NS0 myeloma cells. X-box binding protein 1 (XBP-1) is a key regulator of the cellular secretory pathway and unfolded protein response (UPR). It has been shown that the spliced form of XBP-1, XBP-1S, functions as a transcription activator and up-regulates many genes associated with protein secretion and biosynthesis of endoplasmic reticula. Since the production of some recombinant proteins is widely believed to be limited by the secretory capacity of the host cell, an increase in protein production may be achieved by overexpressing XBP-1S. In this study, the effects of XBP-1S on the productivity of monoclonal antibody (MAb), interferon gamma (IFNgamma), and erythropoietin (EPO) are examined in Chinese hamster ovary (CHO) and NS0 cell lines. Results show that XBP-1S may become a determinative factor only when accumulation of recombinant proteins exceeds the secretory capacity of the host cell. In transient transfection systems where a bottleneck in protein secretion was achieved, overexpression of XBP-1S improved protein titers by up to 2.5-fold. In contrast, overexpression of XBP-1S had no detectable effects on protein productivity of stable cell lines that did not exhibit any secretory bottleneck. We conclude that overexpression of XBP-1S is an effective strategy in enhancing recombinant protein production when the secretory pathway of the host cell is saturated by high-level synthesis of recombinant proteins.

277 ||| 25533398 ||| (2, 0.72) ||| 2 ||| 2 ||| Stability of single copy transgene expression in CHOK1 cells is affected by histone modifications but not by DNA methylation. Intraclonal heterogeneity of genetically modified mammalian cells has been observed as a phenomenon that has a strong impact on overall transgene expression levels and that limits the predictability of transgene expression in genetically modified cells, thereby hampering single cell based screening approaches. The underlying mechanism(s) leading to this variance are poorly understood. To study the dynamics and mechanisms of heterogeneity of early stage silencing we analyzed the expression in more than 100 independent clones of CHOK1 cells that harbour genetically stable integrates of single copy reporter cassettes driven by EF1alpha and CMV promoters. Single cell analysis showed intraclonal variability with heterogeneity in expression in genetically uniform populations. DNA methylation is a well known mechanism responsible for silencing of gene expression. Interestingly, loss of expression was not associated with DNA methylation of the CMV promoter. However, in most of the clonal populations expression could be increased by inhibitors of the histone deacetylases (HDACi) suggesting that heterogeneity of transgene expression is crucially governed by histone modifications. Further, to determine if the epigenetic status of transgene expression is governed by the chromosomal integration locus we targeted heterologous expression cassettes into two chromosomal sites using recombinase mediated cassette exchange (RMCE). The expression status of a particular clone was faithfully re-established when the same promoter used. In this way the problem of early stage cell clone instability can be bypassed. However, upon introduction of an unrelated promoter methylation-independent silencing was observed. Together, these results suggest that histone modifications are the relevant mechanisms by which epigenetic modulation of transgene expression cassettes is governed in the early phase of clone generation.

278 ||| 23606666 ||| (2, 0.72) ||| 2 ||| 2 ||| Chinese hamster ovary K1 host cell enables stable cell line development for antibody molecules which are difficult to express in DUXB11-derived dihydrofolate reductase deficient host cell. Therapeutic monoclonal antibodies (mAb) are often produced in Chinese hamster ovary (CHO) cells. Three commonly used CHO host cells for generating stable cell lines to produce therapeutic proteins are dihydrofolate reductase (DHFR) positive CHOK1, DHFR-deficient DG44, and DUXB11-based DHFR deficient CHO. Current Genentech commercial full-length antibody products have all been produced in the DUXB11-derived DHFR-deficient CHO host. However, it has been challenging to develop stable cell lines producing an appreciable amount of antibody proteins in the DUXB11-derived DHFR-deficient CHO host for some antibody molecules and the CHOK1 host has been explored as an alternative approach. In this work, stable cell lines were developed for three antibody molecules in both DUXB11-based and CHOK1 hosts. Results have shown that the best CHOK1 clones produce about 1 g/l for an antibody mAb1 and about 4 g/l for an antibody mAb2 in 14-day fed batch cultures in shake flasks. In contrast, the DUXB11-based host produced ~0.1 g/l for both antibodies in the same 14-day fed batch shake flask production experiments. For an antibody mAb3, both CHOK1 and DUXB11 host cells can generate stable cell lines with the best clone in each host producing ~2.5 g/l. Additionally, studies have shown that the CHOK1 host cell has a larger endoplasmic reticulum and higher mitochondrial mass.

279 ||| 11170482 ||| (2, 0.72) ||| 2 ||| 2 ||| Key determinants in the occurrence of clonal variation in humanized antibody expression of cho cells during dihydrofolate reductase mediated gene amplification. Recombinant Chinese hamster ovary (CHO) parental clones expressing a humanized antibody against S surface antigen of hepatitis B virus were obtained by cotransfection of heavy chain (HC) and light chain (LC) cDNA expression vectors into dihydrofolate reductase (DHFR)-deficient CHO cells. When 23 representative parental clones were subjected to stepwise selection for increasing methotrexate (MTX) resistance, such as 0.02, 0.08, 0.32, and 1.0 microM, their clonal variations in regard to antibody expression were found to be significant. Among 23 parental clones, only one clone (hu17) showed the significant increment of specific antibody productivity (q(Ab)) with increasing MTX concentration up to 0.32 microM. Compared with the parental clone (hu17), the q(Ab) of hu17 resistant at 0.32 microM MTX (hu17-0.32) was enhanced approximately 12.5-fold. To clarify the reason for the occurrence of clonal variations, Southern blot analyses of chromosomal DNAs derived from each amplified clone at 0.32 microM MTX were performed. Only the hu17-0.32 clone did not experience severe genetic rearrangement during gene amplification, and it had only one 49-kb amplification unit including the LC and HC cDNAs. A fluorescent MTX competition assay showed that the resistance against MTX toxicity of the other clones without enhanced q(Ab) at 0.32 microM MTX was obtained by mechanisms such as an impaired MTX transport system. Taken together, the data obtained here show that clonal variations in regard to antibody expression are found to be significant because clones can acquire MTX resistance by mechanisms other than DHFR-mediated gene amplification despite the stepwise selection.

280 ||| 26033090 ||| (2, 0.71) ||| 2 ||| 2 ||| Engineering selection stringency on expression vector for the production of recombinant human alpha1-antitrypsin using Chinese Hamster ovary cells. BACKGROUND: Expression vector engineering technology is one of the most convenient and timely method for cell line development to meet the rising demand of novel production cell line with high productivity. Destabilization of dihydrofolate reductase (dhfr) selection marker by addition of AU-rich elements and murine ornithine decarboxylase PEST region was previously shown to improve the specific productivities of recombinant human interferon gamma in CHO-DG44 cells. In this study, we evaluated novel combinations of engineered motifs for further selection marker attenuation to improve recombinant human alpha-1-antitrypsin (rhA1AT) production. Motifs tested include tandem PEST elements to promote protein degradation, internal ribosome entry site (IRES) mutations to impede translation initiation, and codon-deoptimized dhfr selection marker to reduce translation efficiency. RESULTS: After a 2-step methotrexate (MTX) amplification to 50 nM that took less than 3 months, the expression vector with IRES point mutation and dhfr-PEST gave a maximum titer of 1.05 g/l with the top producer cell pool. Further MTX amplification to 300 nM MTX gave a maximum titer of 1.15 g/l. Relative transcript copy numbers and dhfr protein expression in the cell pools were also analysed to demonstrate that the transcription of rhA1AT and dhfr genes were correlated due to the IRES linkage, and that the strategies of further attenuating dhfr protein expression with the use of a mutated IRES and tandem PEST, but not codon deoptimization, were effective in reducing dhfr protein levels in suspension serum free culture. CONCLUSIONS: Novel combinations of engineered motifs for further selection marker attenuation were studied to result in the highest reported recombinant protein titer to our knowledge in shake flask batch culture of stable mammalian cell pools at 1.15 g/l, highlighting applicability of expression vector optimization in generating high producing stable cells essential for recombinant protein therapeutics production. Our results also suggest that codon usage of the selection marker should be considered for applications that may involve gene amplification and serum free suspension culture, since the overall codon usage and thus the general expression and regulation of host cell proteins may be affected in the surviving cells.

281 ||| 22926643 ||| (2, 0.71) ||| 2 ||| 2 ||| Overexpression of CHOP alone and in combination with chaperones is effective in improving antibody production in mammalian cells. Secretory capacities including folding and assembly are believed to be limiting factors in the establishment of mammalian cell lines producing high levels of recombinant therapeutic proteins. To achieve industrial success, it is also important to improve protein folding, assembly, and secretory processes in combination with increasing transcription and translation. Here, we identified the expression of CHOP/Gadd153 and GRP78, which are unfolded protein response (UPR)-related genes, correlated with recombinant antibody production in stable CHO cells. Subsequently, CHOP overexpression resulted in increasing recombinant antibody production in some mammalian cell lines, and in addition a threefold further enhancement was obtained by combining expression with UPR-related genes or ER chaperones in transient assays. Overexpression of CHOP had no effect on the biochemical characteristics of the product. These results suggest overexpression of CHOP and its combinations may be an effective method to efficiently select a single cell line with a high level of antibody production in the development of cell lines for manufacturing.

282 ||| 19003402 ||| (2, 0.71) ||| 2 ||| 2 ||| Improved bicistronic mammalian expression vectors using expression augmenting sequence element (EASE). We have recently reported on the isolation of a 5.7 kb segment of Chinese hamster ovary cell genomic DNA, Expression Augmenting Sequence Element (EASE), which when used in bicistronic expression vectors allows the development of stable Chinese hamster ovary cell pools in a five to seven week time period that express high levels of recombinant protein (6-25 mug 10-6 cells/day depending on the protein). In the present study, we have mapped the activity of the EASE to a 2.1 kb region using colony forming assays and developed bicistronic expression vectors with the smaller EASE or control lambda DNA. The recovery of pools expressing the hematopoietic growth factor, FLT3 Ligand, in methotrexate-containing media took 1 to 4 weeks less when using EASE expression vectors compared with control vectors. The cell pools developed with the EASE and control vectors had similar final protein expression levels. Southern blot analysis suggested the expression cassette from the EASE containing vectors integrated in tandem arrays arranged in either head to head or head to tail fashion. By contrast, control vectors appeared to integrate with multiple interruptions to the expression vector. Thus, the EASE, within a bicistronic expression vector, appeared to facilitate tandem vector integration and reduce the time required to develop cell pools for protein expression.

283 ||| 19003276 ||| (2, 0.71) ||| 2 ||| 2 ||| Availability of oncogene activated production system for mass production of light chain of human antibody in CHO cells. We previously established a ras-oncogene amplified Chinesehamster ovary (CHO) cell line, named ras clone I, as anuniversal host cell line for oncogene activated production(OAP) system to mass-produce recombinant protein by activationof the cytomegalovirus immediate early (CMV) promoter with ras protein. The lambda light chain(C5lambda) of human monoclonal antibody HB4C5 is expected tobe potentially useful for lung cancer targeting. We generated aC5lambda hyper-producing cell line by transfecting ras cloneI with the C5lambda gene expression plasmid regulated by theCMV promoter, of which productivity was 5.3 times greater thanthe hyper productive CHO cell line generated by using conventional CHO cells. Introduction of the adenovirus E1A geneinto the hyper-producing cell line derived from ras clone I resulted in further 9.5 times enhancement of the productivity,suggesting the synergistic effect of E1A and ras oncogenes on the recombinant protein production driven by the CMV promoter. In addition, intracellular accumulation of C5lambda andupregulation of BiP was found in hyper-producing cell lineswhich were introduced E1A and ras oncogene. This resultsuggests that excessive intracellular accumulation ofC5lambda protein, which might be caused by that the amount of produced C5lambda in ER is beyond the ability of CHO cells to secrete, might signal the BiP promoter. Our data imply that ras clone I is available as a general host cell for establishing the recombinant protein hyper-producing CHOcells by the OAP system, and suggest that further mass production of recombinant proteins in the OAP system can be possible by clarifying the accurate role of upregulated BiP protein.

284 ||| 10361728 ||| (2, 0.71) ||| 2 ||| 2 ||| Changes during subclone development and ageing of human antibody-producing recombinant CHO cells. Some of the problems encountered with human or human-mouse heterohybridomas, such as low growth rates and high serum requirements, have led to the increased use of recombinant cell lines for production of human antibodies. To evaluate the suitability of such alternative cell lines for the production of human antibodies we have analysed several subclones with differing specific production rates of a recombinant CHO cell line. Gene copy number and site of chromosomal integration for the light and heavy chain and the dhfr gene were determined by in-situ hybridisation. Specific mRNA content was analysed by Northern blot. In addition the intracellular content in light and heavy chain was measured by flow cytometry and the specific secretion rates were determined. The stability of gene expression was followed in the highest producing subclone for over a year. As previously seen in heterohybridoma cells a high expression rate of light chain is beneficial in speeding up secretion rates of whole antibody. When grown in the presence of G418 and methotrexate the amplified gene copies in the genome of recombinant CHO cells were stable over more than 100 passages. However, the expression of light chain, and with it the secretion rate, decreased with time. The low intracellular concentration of light chain resulted in accumulation of heavy chain in the endoplasmic reticulum due to retention by chaperones. The specific secretion rate decreased by 50% after 100 passages. When no G418 or methotrexate were present 75% of the gene copies were lost after 100 passages.

285 ||| 8983022 ||| (2, 0.70) ||| 2 ||| 2 ||| Production of recombinant proteins in Chinese hamster ovary cells overexpressing the subtilisin-like proprotein converting enzyme furin. The proprotein processing enzyme furin is the mammalian prototype of a novel family of subtilisin-like serine endoproteases which possess cleavage specificity for sites involving multiple basic amino acid residues and are involved in the processing of precursor proteins of a variety of regulatory peptides and proteins. One of the limiting steps in the engineering of mammalian cells designed for the overproduction of secreted proteins is the endoproteolytic cleavage of the precursor molecule to its mature biologically active form. The extremely low level of endogenous furin is likely the reason why cells are not able to fully mature overexpressed precursor proteins to their mature form. Here, we report a CHO-derived cell line genetically engineered for the production of high levels of recombinant proteins that need such endoproteolytic maturation. First, the human furin cDNA under the control of the cytomegalovirus early promoter and enhancer was introduced and overexpressed in a DHFR-deficient CHO cell line. A permanent cell line CHO-D3-FUR was established that expressed biologically active furin. Subsequently, to demonstrate the capacity of CHO-D3-FUR cells to produce recombinant proteins in a fully matured form, two derivative cell lines were established that overexpressed the von Willebrand factor (vWF) and transforming growth factor beta 1 (TGF beta 1); CHO-D3-vWF and CHO-D3-TGF beta 1, respectively. Both derivative cell lines were able to produce relatively high levels of recombinant protein in a fully matured and biologically active form. Our results illustrate the potential of the CHO-D3-FUR cell line in the production of recombinant secretory proteins that need endoproteolytic activation at the consensus furin cleavage sequence Arg-X-Lys/Arg-Arg.

286 ||| 25215936 ||| (2, 0.70) ||| 2 ||| 2 ||| A robust transfection reagent for the transfection of CHO and HEK293 cells and production of recombinant proteins and lentiviral particles - PTG1. Bioproduction of recombinant proteins (r-proteins) and recombinant lentiviral particles (r-lentiviral particles) requires robust transfections consisting of efficient protocols that are easy to implement, with good reproducibility for a maximum production of proteins and lentiviral particles in a short time with low cytotoxicity. This study evaluates the capacity of histidinylated polyethyleneimine I (PTG1) to facilitate robust DNA transfection, with low cytotoxicity, of Chinese hamster ovary (CHO) and human embryonic kidney (HEK293T) cells for the production of r-proteins and r-lentiviral particles. We report that PTG1 transfection of cells in suspension with a plasmid DNA encoding enhanced green fluorescent protein leads to 72 and 97% of transfected CHO and HEK293T cells respectively, and does not significantly affect cell viability. PTG1 transfection of 100 mL of CHO-S cell culture in suspension at a cell density of 2 x 10(6) cells /mL resulted in a high level of transfected cells and protein expression after transfection with 0.75 mug/mL plasmid DNA. Transfection with PTG1 is more efficient than LipofectAmine2000 , and gene expression is higher than observed with FreeStyle and JetPEI . Tri-transfection of HEK293T packaging cells leads to the production of a higher level of r-lentiviral particles compared to the calcium phosphate method, and permits two harvests of viral particles within three days. These results show that PTG1 is a powerful new transfection reagent for cell lines frequently used for recombinant protein and lentiviral particle production. PTG1 could be used in protocols for bioproduction of therapeutic proteins such as antibodies for cancer treatments and viral vectors for gene therapy applications.

287 ||| 23081777 ||| (2, 0.70) ||| 2 ||| 2 ||| Exploration of BAC versus plasmid expression vectors in recombinant CHO cells. Vector engineering approaches are commonly used to increase recombinant protein production in mammalian cells, and among various concepts, bacterial artificial chromosomes (BAC) have been proposed to serve as open chromatin regions to omit chromosome positional effects. For proof of concept, we developed stable recombinant Chinese hamster ovary (CHO) cell lines using different expression vector systems: the plasmid vectors contained the identical expression cassette as the BAC constructs. Two anti-HIV1 antibody derivates served as model proteins (3D6scFc and 2F5scFc) for generation of four stable recombinant CHO cell lines. The BAC-derived clones showed three to four times higher specific productivity, and therefore, gene copy numbers and transcript level were quantified. The active chromatin region provided with the BAC environment significantly improved transcription evidenced with both model proteins. Specific transcription was approximately six times higher from BAC-based vectors compared to the corresponding plasmid vectors for both single-chain fragment crystallizable (scFc) proteins. Our accurate investigations elucidated also differences between translational activities related to the protein of choice. 3D6scFc expressed specifically three to four times more product than 2F5scFc indicating that the product by itself also contributes to enhanced productivity. This study indicated comparable increase of transcription level for both scFc proteins when using the BAC system, but translation, maturation, and secretion of individual proteins seem to be protein specific.

288 ||| 20589672 ||| (2, 0.70) ||| 2 ||| 2 ||| Cell line-specific control of recombinant monoclonal antibody production by CHO cells. In this study we compare the cellular control of recombinant human IgG(4) monoclonal antibody (Mab) synthesis in different CHO cell lines. Based on comprehensive empirical analyses of mRNA and polypeptide synthetic intermediates we constructed cell line-specific mathematical models of recombinant Mab manufacture in seven GS-CHO cell lines varying in specific production rate (qMab) over 350-fold. This comparative analysis revealed that control of qMab involved both genetic construct and cell line-specific factors. With respect to the former, all cell lines exhibited excess production of light chain (LC) mRNA and polypeptide relative to heavy chain (HC) mediated by more rapid LC transcription and enhanced LC mRNA stability. Downstream of this, cell lines differed markedly in their relative rates of recombinant mRNA translation, Mab assembly and secretion although HC mRNA abundance and the rate of HC translation generally exerted most control over qMab--the latter being directly proportional to qMab. This study shows that (i) cell lines capable of high qMab exceed a threshold functional competency in all synthetic processes, (ii) the majority of cells in parental and transfected cell populations are functionally limited and (iii) cell engineering strategies to increase Mab production should be cell line specific.

289 ||| 14711493 ||| (2, 0.69) ||| 2 ||| 2 ||| Improved recombinant gene expression in CHO cells using matrix attachment regions. The use of animal cells such as Chinese hamster ovary (CHO) cells for recombinant gene expression provides many advantageous features such as proper folding and post-translational modification of the recombinant protein. However, recombinant genes introduced into animal cells are often expressed at low levels mainly due to position effects from the neighboring chromatin context. The tedious and time-consuming selection and amplification procedure has been the major hurdle for using animal cell line such as CHO cells. To improve mammalian cell expression systems, we screened a variety of matrix/scaffold attachment region (MAR/SAR) elements for their ability to insulate transgene expression from the position effects in CHO cells. We found that the human beta-globin MAR element is particularly effective as the frequency of beta-Gal positive colonies was increased by up to 80%. The expression levels of these colonies were also enhanced about seven-fold. These improvements appear to be related to the increased copy numbers and a higher efficiency of expression of the integrated genes. When this element was used to express soluble TGF-beta type II receptor (sTbetaRII) through the gene amplification system, the frequency of colonies expressing detectable amounts of sTbetaRII was much higher than that of the control vector. We could also generate high sTbetaRII producers with uniform growth properties by a simple two-step amplification process involving two concentrations of methotrexate. This eliminates the need to isolate individual colonies followed by multi-step treatments of methotrexate and thereby greatly simplifies this mammalian expression system.

290 ||| 11242543 ||| (2, 0.69) ||| 2 ||| 2 ||| Gene structure for adenosine kinase in Chinese hamster and human: high-frequency mutants of CHO cells involve deletions of several introns and exons. The structure for the adenosine kinase (AK) gene has been determined from Chinese hamster (CH) and human cells. The AK gene in CH is comprised of 11 exons ranging in length from 36 to 765 nt, with the majority <100 nt. The exact lengths of the intervening introns have not been determined, but most of them are indicated to be very large (>15 kb). A 6.6-kb fragment from human cells was also sequenced, and it contained only a single exon corresponding to exon 10 in CH. The BLAST searches of the subsequently released draft human genome sequence have revealed that the AK gene structure in human is identical to that in CH. In the human genome, the AK exons are distributed over four genomic clones totaling 752 kb, providing direct evidence that the AK gene in mammalian species is unusually large. In contrast to CH and human, the AK genes from several other eukaryotic organisms whose complete genomes are now known are quite small (between 1.2 and 2.5 kb) and either contain no introns (Saccharomyces cerevisiae and Schizosaccharomyces pombe) or various numbers of introns (Drosophila melanogaster [2], Caenorhabditis elegans [4], Arabidopsis thaliana [10]). Some of the intron-exon junctions in these species are in the same positions as in mammals. The AK gene in CH and human, as well as mouse, is linked upstream in a head-to-head fashion with the gene for the clathrin adaptor mu3 protein (or beta 3A subunit of the AP-3 protein complex), which is affected in type 2 Hermansky-Pudlak syndrome. These two genes are separated by <200 nt, and it is possible that they have a common or overlapping promoter(s). We have also determined the nature of the genetic alterations in two of the class A AK(-) mutants of CHO cells, which are obtained at a very high spontaneous frequency (10(-3)-10(-4)) in this cell line. Both mutants contained large deletions within the AK gene and greatly shortened AK transcripts. The cloning and sequencing of the transcripts from these mutants showed that the deletion in one of them led to the loss of exons 5 through 8, whereas in the other, all exons from 2 through 8 are deleted. The endpoints of these deletions lie in the large introns within the AK gene.

291 ||| 9234898 ||| (2, 0.68) ||| 2 ||| 2 ||| A generic method for the production of cell lines expressing high levels of 7-transmembrane receptors. G-protein-coupled or 7-transmembrane receptors (7TMRs) are often studied after heterologous expression in mammalian cells such as COS-7, CHO-K1, or HEK-293s. In this paper, we describe the development of a rapid and generic method for producing stable Chinese hamster ovary cell lines expressing high levels of recombinant 7TMRs by N-terminal tagging these proteins with the hemagglutinin (HA) sequence. To illustrate the broad applicability of this technique, we have presented data from cell lines expressing a glycoprotein hormone receptor for follicle-stimulating hormone (FSHR), CXC- (CXCR-2), and CC-chemokine (CCR-1) receptors and peptide receptors from the somatostatin (SSTR1, 2, 5) and neuropeptide Y (NPY-Y2, -Y4 Rs) families. Typically, cell lines with a receptor density of 1 to 15 pmol/mg protein are produced with this method. The presence of the HA tag does not adversely affect the binding or functional activity of the receptors.

292 ||| 24108600 ||| (2, 0.68) ||| 2 ||| 2 ||| Transcriptome dynamics of transgene amplification in Chinese hamster ovary cells. Dihydrofolate reductase (DHFR) system is used to amplify the product gene to multiple copies in Chinese Hamster Ovary (CHO) cells for generating cell lines which produce the recombinant protein at high levels. The physiological changes accompanying the transformation of the non-protein secreting host cells to a high producing cell line is not well characterized. We performed transcriptome analysis on CHO cells undergoing the selection and amplification processes. A host CHO cell line was transfected with a vector containing genes encoding the mouse DHFR (mDHFR) and a recombinant human IgG (hIgG). Clones were isolated following selection and subcloned following amplification. Control cells were transfected with a control plasmid which did not have the hIgG genes. Although methotrexate (MTX) amplification increased the transcript level of the mDHFR gene significantly, its effect on both hIgG heavy and light chain genes was more modest. The subclones appeared to retain the transcriptome signatures of their parental clones, however, their productivity varied among those derived from the same clone. The transcript levels of hIgG transgenes of all subclones fall in a narrower range than the product titer, alluding to the role of many functional attributes, other than transgene transcript, on productivity. We cross examined functional class enrichment during selection and amplification as well as between high and low producers and discerned common features among them. We hypothesize that the role of amplification is not merely increasing transcript levels, but also enriching survivors which have developed the cellular machinery for secreting proteins, leading to an increased frequency of isolating high-producing clones. We put forward the possibility of assembling a hyper-productivity gene set through comparative transcriptome analysis of a wide range of samples.

293 ||| 24518824 ||| (2, 0.67) ||| 2 ||| 2 ||| High-throughput ClonePix FL analysis of mAb-expressing clones using the UCOE expression system. Therapeutic recombinant monoclonal antibodies (mAbs) are commonly produced by high-expressing, clonal, mammalian cells. Creation of these clones for manufacturing remains heavily reliant on stringent selection and gene amplification, which in turn can lead to genetic instability, variable expression, product heterogeneity and prolonged development timelines. Inclusion of cis-acting ubiquitous chromatin opening elements (UCOE ) in mammalian expression vectors has been shown to improve productivity and facilitate high-level gene expression irrespective of the chromosomal integration site without lengthy gene amplification protocols. In this study we have used high-throughput robotic clone selection in combination with UCOE containing expression vectors to develop a rapid, streamlined approach for early-stage cell line development and isolation of high-expressing clones for mAb production using Chinese hamster ovary (CHO) cells. Our results demonstrate that it is possible to go from transfection to stable clones in only 4 weeks, while achieving specific productivities exceeding 20 pg/cell/day. Furthermore, we have used this approach to quickly screen several process-crucial parameters including IgG subtype, enhancer-promoter combination and UCOE length. The use of UCOE -containing vectors in combination with automated robotic selection provides a rapid method for the selection of stable, high-expressing clones.

294 ||| 24397363 ||| (2, 0.67) ||| 2 ||| 2 ||| Scaffold/matrix attachment regions from CHO cell chromosome enhanced the stable transfection efficiency and the expression of transgene in CHO cells. Two scaffold/matrix attachment regions (S/MARs) from Chinese hamster ovary (CHO) cell chromosomes were identified computationally and cloned as the domain boundaries regions. As partition chromosomes in independently regulated units to increase the exogenous gene expression, these two S/MARs (MAR1 and MAR6) flanked expression vectors were respectively transfected into CHO cells. The results showed that MAR1 and MAR6 were much more potent to increase the stable transfection efficiency than the chicken lysozyme gene 5' S/MARs, which had been experimentally identified to increasing the expression of heterologous gene. Moreover, MAR6 is more active in mediating high and consistent gene expression in CHO cells than the chicken lysozyme gene 5' S/MARs.

295 ||| 23874717 ||| (2, 0.67) ||| 2 ||| 2 ||| Multi-host expression system for recombinant production of challenging proteins. Recombinant production of complex eukaryotic proteins for structural analyses typically requires a profound screening process to identify suitable constructs for the expression of ample amounts of properly folded protein. Furthermore, the evaluation of an optimal expression host has a major impact on protein yield and quality as well as on actual cost of the production process. Here we present a novel fast expression system for multiple hosts based on a single donor vector termed pFlp-Bac-to-Mam. The range of applications of pFlp-Bac-to-Mam comprises highly efficient transient transfection of HEK293-6E in serum-free suspension culture and subsequent large-scale production of challenging proteins expressing in mg per Liter level using either the baculoviral expression vector system or stable CHO production cell lines generated by Flp-mediated cassette exchange. The success of the multi-host expression vector to identify the optimal expression strategy for efficient production of high quality protein is demonstrated in a comparative expression study of three model proteins representing different protein classes: intracellular expression using a fluorescent protein, secretion of a single-chain-Fv-hIgG1Fc fusion construct and production of a large amount of highly homogeneous protein sample of the extracellular domain of a Toll-like receptor. The evaluation of the production efficiency shows that the pFlp-Bac-to-Mam system allows a fast and individual optimization of the expression strategy for each protein class.

296 ||| 21618470 ||| (2, 0.67) ||| 2 ||| 2 ||| Promoter methylation and transgene copy numbers predict unstable protein production in recombinant Chinese hamster ovary cell lines. Mammalian cell lines for recombinant protein production need to maintain productivity over extended cultivation times. Long-term stability studies are time and resource intensive, but are widely performed to identify and eliminate unstable candidates during cell line development. Production instability of manufacturing cell lines can be associated with methylation and silencing of the heterologous promoter. We have identified CpG dinucleotides within the human cytomegalovirus major immediate early promoter/enhancer (hCMV-MIE) that are frequently methylated in unstable antibody-producing Chinese hamster ovary (CHO) cell lines. We have established methylation-specific real-time qPCR for the rapid and sensitive measurement of hCMV-MIE methylation in multiple cell lines and provide evidence that hCMV-MIE methylation and transgene copy numbers can be used as early markers to predict production instability of recombinant CHO cell lines. These markers should provide the opportunity to enrich stable producers early in cell line development and allow developers to put more emphasis on other criteria, such as product quality and bioprocess robustness.

297 ||| 11291036 ||| (2, 0.67) ||| 2 ||| 2 ||| Efficient selection of high-producing subclones during gene amplification of recombinant Chinese hamster ovary cells by flow cytometry and cell sorting. The screening procedure for high-producing cell lines is extremely time- and labor-intensive and costly, and is at present guided by an empirical approach based on individual experience. Flow cytometry and cell sorting, with its ability to analyze and separate single cells, an ideal method in the selection of such rare cells. The isolation of recombinant cell lines is especially difficult due to repeated gene amplification, which introduces high mutational variation into the population. We have established and evaluated a modification of a previous method that traps secreted product on the surface of the secreting cell, thus allowing direct analysis of single cell specific production rates. This method was used to select for high-producing subclones of a recombinant Chinese hamster ovary (CHO) cell line producing a human antibody against HIV-1 by repeated rounds of gene amplification and cell sorting. This cell line has been amplified in previous investigations, so that the amount of work and testing required by traditional methods can be compared with the protocol described herein. Forty-five 96-well plates were necessary to obtain a high-producing subclone by limited dilution methods, whereas only five plates were required when cell sorting was used. The specific production rate of the best clone obtained by sorting, however, was five times that of the clone obtained by traditional methods. In contrast to the clones obtained by limited dilution, which consisted of several populations of low- and high-producing cells even at high methotrexate concentrations (6.4 microM), the clones isolated by sorting were already homogeneous at 0.8 microM methotrexate.

298 ||| 10592510 ||| (2, 0.67) ||| 2 ||| 2 ||| Engineering CHO cells to overexpress a secreted reporter protein upon induction from mouse mammary tumor virus promoter. The mouse mammary tumor virus (MMTV) promoter is induced by the addition of a glucocorticoid hormone or analog such as dexamethasone. The hormone binds to its specific transcription factor, glucocorticoid receptor (GR), and the activated complex then binds to the glucocorticoid response elements (GREs) in the enhancer region of the MMTV promoter to induce the overexpression of downstream genes. We have constructed an expression vector for a reporter protein, secreted alkaline phosphatase (SEAP),controlled by the MMTV promoter and co-transfected this vector along with a GR expression cassette into Chinese hamster ovary (CHO) cells. High producers were cloned and grown in suspension cultures. A very high titer, over 0.4 mg/mL, of SEAP was obtained from this inducible overexpression system, about ten times that achievable with the same reporter protein using the strong constitutive SV40 promoter in CHO cells. A peak production rate of 187 pg SEAP per cell per day was observed within 3 days after induction, compared to the peak rate of 23 pg SEAP per cell per day expressed using the constitutive SV40 promoter. With the reduced or zero growth rate during the protein production phase, this novel MMTV overexpression system is highly suited for optimizing glycoprotein synthesis rates in high cell density fed-batch or perfusion bioreactors.

299 ||| 25683738 ||| (2, 0.66) ||| 2 ||| 2 ||| Transcriptional and post-transcriptional limitations of high-yielding, PEI-mediated transient transfection with CHO and HEK-293E cells. Transient gene expression (TGE) in human embryonic kidney (HEK-293) and Chinese hamster ovary (CHO) cells is a well-established technology for the rapid generation of recombinant proteins. Although the maximum TGE yields have reached 1 g/L or more, the amount of plasmid DNA (pDNA) required for transfection remains high. Although greater than 10(3) copies of pDNA are present per transfected cell, protein yields are still lower than those achieved in recombinant cell lines with only one or a few copies of the transgene. This indicates a clear limitation to TGE in terms of the maximum level of recombinant protein production. In this study, we investigated the limitations to high-yielding TGE processes with CHO and HEK-293E cells using a monoclonal antibody as a model protein. For either cell host, both the intracellular and intranuclear pDNA levels increased linearly with the amount of pDNA added to the culture. In contrast, transgene mRNA accumulation reached a plateau as the intranuclear pDNA amount increased, suggesting a limitation in pDNA transcription. A post-transcriptional limitation to TGE yields was revealed by calculating the amount of antibody produced per transgene mRNA (mRNA utilization). For both hosts the transgene mRNA utilization decreased dramatically when transfected pDNA amounts increased beyond the level giving the maximum protein yield. The post-transcriptional limitation did not appear to be due to bottlenecks in antibody assembly or secretion, suggesting that transgene mRNA translation may be limiting. The results show that TGE yields are not limited by pDNA delivery into the nuclei, but in pDNA and transgene mRNA utilization.

300 ||| 23615744 ||| (2, 0.66) ||| 2 ||| 2 ||| Rapid construction of transgene-amplified CHO cell lines by cell cycle checkpoint engineering. The process of establishing high-producing cell lines for the manufacture of therapeutic proteins is usually both time-consuming and laborious due to the low probability of obtaining high-producing clones from a pool of transfected cells and slow cell growth under the strong selective pressure of screening to identify high-producing clones. We present a novel method to rapidly generate more high-producing cells by accelerating transgene amplification. A small interfering RNA (siRNA) expression vector against ataxia telangiectasia and Rad3 related (ATR), a cell cycle checkpoint kinase, was transfected into Chinese hamster ovary (CHO) cells. The influences of ATR downregulation on gene amplification and the productivity were investigated in CHO cells producing green fluorescent protein (GFP) and secreting monoclonal antibody (mAb). The ATR-downregulated cells showed up to a 6-fold higher ratio of GFP-positive cells than that of the control cell pool. Moreover, the downregulated mAb-producing cells had about a 4-fold higher specific production rate and a 3-fold higher volumetric productivity as compared with the mock cells. ATR-downregulated cells showed a much faster increase in transgene copy numbers during the gene amplification process via methotrexate (MTX) treatment in both GFP- and mAb-producing cells. Our results suggest that a pool of high-producing cells can be more rapidly generated by ATR downregulation as compared with conventional gene amplification by MTX treatment. This novel method may be a promising approach to reduce time and labor in the process of cell line development.

301 ||| 24557570 ||| (2, 0.65) ||| 2 ||| 2 ||| In search of expression bottlenecks in recombinant CHO cell lines--a case study. The efficient production of recombinant proteins such as antibodies typically involves the screening of an extravagant number of clones in order to finally select a stable and high-producing cell line. Thereby, the underlying principles of a powerful protein machinery, but also potential expression limitations, often remain poorly understood. To shed more light on this topic, we applied several different techniques to investigate a previously generated cell line (4B3-IgA), which expressed recombinant immunoglobulin A (IgA) with an unusually low specific productivity. Results were compared to the host cell line and to another recombinant CHO cell line (3D6-IgA) expressing another IgA that binds to an overlapping epitope. The low specific productivity of clone 4B3-IgA could not be explained by GCN or mRNA levels, but insufficiencies in protein maturation and/or secretion were determined. Despite the presence of free light chain polypeptides, they occasionally failed to associate with their heavy chain partners. Consequently, heavy chains were misassembled and accumulated to form intracellular aggregates, so-called Russell bodies. These protein deposits evoked the expression of increased amounts of ER-resident chaperones to combat the induced stress. Despite bottlenecks in protein processing, the cells' quality checkpoints remained intact, and predominantly correctly processed IgA was exported into the culture medium. The results of our study demonstrated that recombinant protein expression was impaired by heavy chain aggregation despite the presence of a disposable light chain and revealed elevated chaperone formation in combination with limited antibody assembly. Our studies suggest that the primary amino acid sequence and consequently the resulting structure of an expressed protein need to be considered as a factor influencing a cell's productivity.

302 ||| 24026344 ||| (2, 0.65) ||| 2 ||| 2 ||| Improved antibody production in Chinese hamster ovary cells by ATF4 overexpression. To improve antibody production in Chinese hamster ovary (CHO) cells, the humanized antibody-producing CHO DP-12-SF cell line was transfected with the gene encoding activating transcription factor 4 (ATF4), a central factor in the unfolded protein response. Overexpression of ATF4 significantly enhanced the production of antibody in the CHO DP-12-SF cell line. The specific IgG production rate of in the ATF4-overexpressing CHO-ATF4-16 cells was approximately 2.4 times that of the parental host cell line. Clone CHO-ATF4-16 did not show any change in growth rate compared with the parental cells or mock-transfected CHO-DP12-SF cells. The expression levels of mRNAs encoding both the antibody heavy and light chains in the CHO-ATF4-16 clone were analyzed. This analysis showed that ATF4 overexpression improved the total production and specific production rate of antibody without affecting the mRNA transcription level. These results indicate that ATF4 overexpression is a promising method for improving recombinant IgG production in CHO cells.

303 ||| 21804562 ||| (2, 0.65) ||| 2 ||| 2 ||| The genomic sequence of the Chinese hamster ovary (CHO)-K1 cell line. Chinese hamster ovary (CHO)-derived cell lines are the preferred host cells for the production of therapeutic proteins. Here we present a draft genomic sequence of the CHO-K1 ancestral cell line. The assembly comprises 2.45 Gb of genomic sequence, with 24,383 predicted genes. We associate most of the assembled scaffolds with 21 chromosomes isolated by microfluidics to identify chromosomal locations of genes. Furthermore, we investigate genes involved in glycosylation, which affect therapeutic protein quality, and viral susceptibility genes, which are relevant to cell engineering and regulatory concerns. Homologs of most human glycosylation-associated genes are present in the CHO-K1 genome, although 141 of these homologs are not expressed under exponential growth conditions. Many important viral entry genes are also present in the genome but not expressed, which may explain the unusual viral resistance property of CHO cell lines. We discuss how the availability of this genome sequence may facilitate genome-scale science for the optimization of biopharmaceutical protein production.

304 ||| 25706993 ||| (2, 0.64) ||| 2 ||| 2 ||| Optimization of heavy chain and light chain signal peptides for high level expression of therapeutic antibodies in CHO cells. Translocation of a nascent protein from the cytosol into the ER mediated by its signal peptide is a critical step in protein secretion. The aim of this work was to develop a platform technology to optimize the signal peptides for high level production of therapeutic antibodies in CHO cells. A database of signal peptides from a large number of human immunoglobulin (Ig) heavy chain (HC) and kappa light chain (LC) was generated. Most of the HC signal peptides contain 19 amino acids which can be divided into three domains and the LC signal peptides contain 22 amino acids. The signal peptides were then clustered according to sequence similarity. Based on the clustering, 8 HC and 2 LC signal peptides were analyzed for their impacts on the production of 5-top selling antibody therapeutics, namely, Herceptin, Avastin, Remicade, Rituxan, and Humira. The best HC and LC signal peptides for producing these 5 antibodies were identified. The optimized signal peptides for Rituxan is 2-fold better compared to its native signal peptides which are available in the public database. Substitution of a single amino acid in the optimized HC signal peptide for Avastin reduced its production significantly. Mass spectrometry analyses revealed that all optimized signal peptides are accurately removed in the mature antibodies. The results presented in this report are particularly important for the production of these 5 antibodies as biosimilar drugs. They also have the potential to be the best signal peptides for the production of new antibodies in CHO cells.

305 ||| 22467228 ||| (2, 0.64) ||| 2 ||| 2 ||| Identifying bottlenecks in transient and stable production of recombinant monoclonal-antibody sequence variants in Chinese hamster ovary cells. The increasing demand for antibody-based therapeutics has emphasized the need for technologies to improve recombinant antibody titers from mammalian cell lines. Moreover, as antibody therapeutics address an increasing spectrum of indications, interest has increased in antibody engineering to improve affinity and biological activity. However, the cellular mechanisms that dictate expression and the relationships between antibody sequence and expression level remain poorly understood. Fundamental understanding of how mammalian cells handle high levels of transgene expression and of the relationship between sequence and expression are vital to the development of new antibodies and for increasing recombinant antibody titers. In this work, we analyzed a pair of mutants that vary by a single amino acid at Kabat position 49 (heavy-chain framework), resulting in differential transient and stable titers with no apparent loss of antigen affinity. Through analysis of mRNA, gene copy number, intracellular antibody content, and secreted antibody, we found that while translational/post-translational mechanisms are limiting in transient systems, it appears that the amount of available transgenic mRNA becomes the limiting event on stable integration of the recombinant genes. We also show that amino acid substitution at residue 49 results in production of a non-secreted HC variant and postulate that stable antibody expression is maintained at a level which prevents toxic accumulation of this HC-related protein. This study highlights the need for proper sequence engineering strategies when developing therapeutic antibodies and alludes to the early analysis of transient expression systems to identify the potential for aberrant stable expression behavior.

306 ||| 17083990 ||| (2, 0.64) ||| 2 ||| 2 ||| High level expression of proteins using sequences from the ferritin heavy chain gene locus. An expression vector has been generated using a gene highly expressed under conditions found in a typical fed-batch bioreactor process. The ferritin heavy chain (HC) gene exhibits higher levels of expression in the late stages of a fed-batch bioreactor than in the early stages. This property was considered advantageous for an expression vector, since the maximal cell density would coincide with maximal expression. The rat ferritin HC genomic region was isolated and converted into an expression vector where large segments of 5' and 3' flanking regions were included in an attempt to recreate the same high level of expression in stably transfected cells. Expression from the resulting ferritin HC vector was compared to vectors containing the commonly used strong promoters, CMV IE, and SV40 early promoter/enhancer, in the generation of stable transfectants. The ferritin HC vector was able to generate cell lines with significantly higher expression levels than those under the control of the viral promoters.

307 ||| 21882365 ||| (2, 0.63) ||| 2 ||| 2 ||| Impact of gene vector design on the control of recombinant monoclonal antibody production by Chinese hamster ovary cells. In this study, we systematically compare two vector design strategies for recombinant monoclonal antibody (Mab) synthesis by Chinese hamster ovary (CHO) cells; a dual open reading frame (ORF) expression vector utilizing separate cytomegalovirus (CMV) promoters to drive heavy chain (HC) and light chain (LC) expression independently, and a single ORF vector design employing a single CMV promoter to drive HC and LC polypeptide expression joined by a foot and mouth disease virus F2A polypeptide self-cleaving linker sequence. Initial analysis of stable transfectants showed that transfectants utilizing the single ORF vector designs exhibited significantly reduced Mab production. We employed an empirical modeling strategy to quantitatively describe the cellular constraints on recombinant Mab synthesis in all stable transfectants. In all transfectants, an intracellular molar excess of LC polypeptide over HC polypeptide was observed. For CHO cells transfected with the single ORF vectors, model-predicted, and empirical intracellular intermediate levels could only be reconciled by inclusion of nascent HC polypeptide degradation. Whilst a local sensitivity analysis showed that qMab of all transfectants was primarily constrained by recombinant mRNA translation rate, our data indicated that all single ORF transfectants exhibited a reduced level of recombinant gene transcription and that Mab folding and assembly reactions generically exerted greater control over qMab. We infer that the productivity of single ORF transfectants is limited by ER processing/degradation "capacity" which sets a limit on transcriptional input. We conclude that gene vector design for oligomeric recombinant proteins should be based on an understanding of protein-specific synthetic kinetics rather than polypeptide stoichiometry.

308 ||| 18327552 ||| (2, 0.63) ||| 2 ||| 2 ||| Transient transfection factors for high-level recombinant protein production in suspension cultured mammalian cells. The efficient transfection of cloned genes into mammalian cells system plays a critical role in the production of large quantities of recombinant proteins (r-proteins). In order to establish a simple and scaleable transient protein production system, we have used a cationic lipid-based transfection reagent-FreeStyle MAX to study transient transfection in serum-free suspension human embryonic kidney (HEK) 293 and Chinese hamster ovary (CHO) cells. We used quantification of green fluorescent protein (GFP) to monitor transfection efficiency and expression of a cloned human IgG antibody to monitor r-protein production. Parameters including transfection reagent concentration, DNA concentration, the time of complex formation, and the cell density at the time of transfection were analyzed and optimized. About 70% GFP-positive cells and 50-80 mg/l of secreted IgG antibody were obtained in both HEK-293 and CHO cells under optimal conditions. Scale-up of the transfection system to 1 l resulted in similar transfection efficiency and protein production. In addition, we evaluated production of therapeutic proteins such as human erythropoietin and human blood coagulation factor IX in both HEK-293 and CHO cells. Our results showed that the higher quantity of protein production was obtained by using optimal transient transfection conditions in serum-free adapted suspension mammalian cells.

309 ||| 17310326 ||| (2, 0.63) ||| 2 ||| 2 ||| Scalable transient gene expression in Chinese hamster ovary cells in instrumented and non-instrumented cultivation systems. Cell expansion, gene transfer and protein production were all executed with a single serum-free, animal protein-free commercial medium designed for suspension-adapted Chinese hamster ovary cells (CHO DG44). This is a most important process to consider for clinical production of recombinant proteins. The transfection with polyethylenimine (PEI) was shown here to be scalable using both stirred-tank bioreactors of 3- and 150-l and novel agitated cultivation vessels (50 ml ventilated centrifuge tubes and 1-l square-shaped glass bottles) that lack any instrumentation. The transient transfections spanned a range of working volumes from 2 ml to 80 l. The maximum transient recombinant antibody yield was 22 mg/l, the highest ever reported for a multiliter transfection in CHO. The transiently expressed protein had the same extent of glycosylation as the same antibody produced from a stably transfected recombinant CHO cell line.

310 ||| 23335490 ||| (2, 0.62) ||| 2 ||| 2 ||| A CHO cell line engineered to express XBP1 and ERO1-Lalpha has increased levels of transient protein expression. Transient gene expression (TGE) systems currently provide rapid and scalable (up to 100 L) methods for generating multigram quantities of recombinant heterologous proteins. Product titers of up to 1 g/L have been demonstrated in HEK293 cells but reported yields from Chinese hamster ovary (CHO) cells are lower at ~300 mg/L. We report on the establishment of an engineered CHOS cell line, which has been developed for TGE. This cell line has been engineered to express both X-box binding protein (XBP-1S) and endoplasmic reticulum oxidoreductase (ERO1-Lalpha) and has been named CHOS-XE. CHOS-XE cells produced increased antibody (MAb) yields (5.3- 6.2 fold) in comparison to CHOS cells. Product quality was unchanged as assessed by size, charge, propensity to aggregate, major glycosylation species, and thermal stability. To further develop and test this TGE system, five commercial media were assessed, and one was shown to offer the greatest increase in antibody yields. With the addition of a commercial feed, MAb titers reached 875 mg/L.

311 ||| 22359058 ||| (2, 0.62) ||| 2 ||| 2 ||| Quantitative analysis of transcription and translation in gene amplified Chinese hamster ovary cells on the basis of a kinetic model. The elevation of expression levels for secreted glycoproteins by gene amplification in mammalian cells shows a saturation behavior at high levels of gene amplification. At high expression levels a drop in the secretion efficiency for the recombinant protein occurs (Schroder and Friedl, 1997), coinciding with the appearance of misfolded protein in the cell. In this communication we investigated whether additional limitations exist at the levels of transcription and translation. Four Chinese hamster ovary (CHO) cell lines expressing different amounts of human antithrombin III (ATIII) were used as a model system. A tenfold increase in the ATIII cDNA copy number from the lowest to the highest producing cell line coincided with a 38-fold increase in ATIII mRNA levels, and an 80-fold increase in the amount of intracellular ATIII levels. The data was analyzed using a simple kinetic model. The following conclusions were derived: I. The transcriptional activity for the recombinant protein is not saturated. II. Translation itself is not saturated either, but may be downregulated as secretion efficiency drops. III. Two explanations for the previously reported drop in secretion efficiency for the recombinant protein with increasing expression level are possible: A. Protein degradation is an alternative fate for translated ATIII and the fraction of ATIII degraded after translation increases as expression level is increased. B. Translation is downregulated as the secretory apparatus becomes exhausted to maintain cell viability.

312 ||| 20625180 ||| (2, 0.62) ||| 2 ||| 2 ||| Meganuclease-driven targeted integration in CHO-K1 cells for the fast generation of HTS-compatible cell-based assays. The development of cell-based assays for high-throughput screening (HTS) approaches often requires the generation of stable transformant cell lines. However, these cell lines are essentially created by random integration of a gene of interest (GOI) with no control over the level and stability of gene expression. The authors developed a targeted integration system in Chinese hamster ovary (CHO) cells, called the cellular genome positioning system (cGPS), based on the stimulation of homologous gene targeting by meganucleases. Five different GOIs were knocked in at the same locus in cGPS CHO-K1 cells. Further characterization revealed that the cGPS CHO-K1 system is more rapid (2-week protocol), efficient (all selected clones expressed the GOI), reproducible (GOI expression level variation of 12%), and stable over time (no change in GOI expression after 23 weeks of culture) than classical random integration. Moreover, in all cGPS CHO-K1 targeted clones, the recombinant protein was biologically active and its properties similar to the endogenous protein. This fast and robust method opens the door for creating large collections of cell lines of better quality and expressing therapeutically relevant GOIs at physiological levels, thereby enhancing the potential scope of HTS.

313 ||| 23876479 ||| (2, 0.61) ||| 2 ||| 2 ||| Enhanced expression of codon optimized interferon gamma in CHO cells. The human interferon-gamma (IFN-gamma) is a potential drug candidate for treating various diseases due to its immunomodulatory properties. The efficient production of this protein can be achieved through a popular industrial host, Chinese hamster ovary (CHO) cells. However, recombinant expression of foreign proteins is typically suboptimal possibly due to the usage of non-native codon patterns within the coding sequence. Therefore, we demonstrated the application of a recently developed codon optimization approach to design synthetic IFN-gamma coding sequences for enhanced heterologous expression in CHO cells. For codon optimization, earlier studies suggested to establish the target usage distribution pattern in terms of selected design parameters such as individual codon usage (ICU) and codon context (CC), mainly based on the host's highly expressed genes. However, our RNA-Seq based transcriptome profiling indicated that the ICU and CC distribution patterns of different gene expression classes in CHO cell are relatively similar, unlike other microbial expression hosts, Escherichia coli and Saccharomyces cerevisiae. This finding was further corroborated through the in vivo expression of various ICU and CC optimized IFN-gamma in CHO cells. Interestingly, the CC-optimized genes exhibited at least 13-fold increase in expression level compared to the wild-type IFN-gamma while a maximum of 10-fold increase was observed for the ICU-optimized genes. Although design criteria based on individual codons, such as ICU, have been widely used for gene optimization, our experimental results suggested that codon context is relatively more effective parameter for improving recombinant IFN-gamma expression in CHO cells.

314 ||| 23018810 ||| (2, 0.61) ||| 2 ||| 2 ||| Identification of a single base-pair mutation of TAA (Stop codon) GAA (Glu) that causes light chain extension in a CHO cell derived IgG1. We describe here the identification of a stop codon TAA (Stop) GAA (Glu) = Stop221E mutation on the light chain of a recombinant IgG1 antibody expressed in a Chinese hamster ovary (CHO) cell line. The extended light chain variants, which were caused by translation beyond the mutated stop codon to the next alternative in-frame stop codon, were observed by mass spectra analysis. The abnormal peptide peaks present in tryptic and chymotryptic LC-MS peptide mapping were confirmed by N-terminal sequencing as C-terminal light chain extension peptides. Furthermore, LC-MS/MS of Glu-C peptide mapping confirmed the stop221E mutation, which is consistent with a single base-pair mutation in TAA (stop codon) to GAA (Glu). The light chain variants were approximately 13.6% of wild type light chain as estimated by RP-HPLC analysis. DNA sequencing techniques determined a single base pair stop codon mutation, instead of a stop codon read-through, as the cause of this light chain extension. To our knowledge, the stop codon mutation has not been reported for IgGs expressed in CHO cells. These results demonstrate orthogonal techniques should be implemented to characterize recombinant proteins and select appropriate cell lines for production of therapeutic proteins because modifications could occur at unexpected locations.

315 ||| 19134553 ||| (2, 0.61) ||| 2 ||| 2 ||| Overexpression of GADD34 enhances production of recombinant human antithrombin III in Chinese hamster ovary cells. To improve the production of recombinant human antithrombin III (AT-III) in Chinese hamster ovary (CHO) cells, the gene encoding growth arrest and DNA damage inducible protein 34 (GADD34), which is a transcription factor involved in the unfolded protein response (UPR), was cloned from CHO-K1 cells. Overexpression of GADD34 significantly enhanced the production of recombinant AT-III in CHO 13D-35D cells. The specific rate of AT-III production in the GADD34-overexpressing CHO 13D-35D cells reached approximately 28 pg/cell/d. After 144 h of incubation, the AT-III concentration in the culture supernatant was approximately 40% higher than that observed in the case of the parental CHO 13D-35D cells. The mRNA expression, specific activity, and fucosylation of AT-III were not affected by GADD34 overexpression. Overexpression of GADD34 is a promising method of improving the production of secreted protein pharmaceuticals in CHO cells.

316 ||| 25219685 ||| (2, 0.60) ||| 2 ||| 2 ||| Characterization and expression of proprotein convertases in CHO cells: Efficient proteolytic maturation of human bone morphogenetic protein-7. Bone morphogenetic protein-7 (BMP-7) is synthesized as a precursor that requires proteolytic cleavage of the propeptide by proprotein convertases (PCs) for its functional activity. A high-level expression of BMP-7 in CHO cells (CHO-BMP-7) resulted in secretion of a mixture of inactive precursor and active BMP-7. In an effort to achieve efficient processing of BMP-7 in CHO cells, PCs responsible for cleavage of the precursors in CHO cells were characterized. Analysis of the mRNA expression levels of four PCs (furin, PACE4, PC5/6, and PC7) revealed that only furin and PC7 genes are expressed in CHO-BMP-7 cells. Specific inhibition of the PCs by hexa-D-arginine (D6R) or decanoyl-RVKR-chloromethyl ketone (RVKR-CMK) further revealed that furin is mainly responsible for the proteolytic processing of BMP-7. To identify a more efficient PC for BMP-7 processing, the four PC genes were transiently expressed in CHO-BMP-7 cells, respectively. Among these, PC5/6 was found to be the most efficient in BMP-7 processing. Stable overexpression of PC5/6DeltaC, a secreted form of PC5/6, significantly improved mature BMP-7 production in CHO-BMP-7 cells. When the maximum BMP-7 concentration was obtained in the culture of CHO-BMP-7 cells, approximately 88% of BMP-7 was unprocessed. In contrast, no precursor was found in the culture of PC5/6DeltaC-overexpressing cells (clone #97). Furthermore, the in vitro biological activity of the mature BMP-7 from PC5/6DeltaC-overexpressing cells was comparable to that from CHO-BMP-7 cells. Taken together, the present results indicate that overexpression of PC5/6DeltaC in CHO-BMP-7 cells is an efficient means of increasing the yield of BMP-7.

317 ||| 19041912 ||| (2, 0.60) ||| 2 ||| 2 ||| The 3'UTR of HIC mRNA improves the production of recombinant proteins in Chinese hamster ovary cells. Positive transcription elongation factor b (P-TEFb) is an important transcriptional regulator which controls 70-80% of RNA polymerase II transcription. It has been reported that the human I-mfa (inhibitor of MyoD family a) domain-containing protein (HIC) interacts with P-TEFb and that expression of HIC cDNA stimulates P-TEFb-dependent transcription. Interestingly, our recent study shows that transcriptional stimulation by HIC is predominately due to the 3' untranslated region (3'UTR) of HIC mRNA rather than its coding region. In this report, we investigate the effects of HIC 3'UTR on recombinant protein expression in mammalian cells. In transient transfections, overexpression of HIC 3'UTR stimulates transgene expression in several mammalian cell lines and significantly increases the production of human erythropoietin and interferon-gamma in Chinese hamster ovary (CHO) cells. This is the first report that demonstrates the improvement of expression of biopharmaceutical proteins by overexpressing a non-coding 3'UTR in CHO cells.

318 ||| 19003083 ||| (2, 0.59) ||| 2 ||| 2 ||| Increased production of a secreted glycoprotein in engineered CHO cells through amplification of a transcription factor. Expression levels of reporter protein driven by Mouse Mammary Tumor Virus Promoter system were improved by expressing its specific transcription factor (glucocorticoid receptor) from a different expression vector. The vector that expresses glucocorticoid receptor (GR) also contained dihydrofolate reductase (dhfr) gene as a selection marker. In the presentstudy we amplified the glucocorticoid receptor gene (gr)along with the dhfr gene by adapting the cell lines to increasing concentrations of methotrexate, an antifolate analog. Stepwise increases in the volumetric titers of a secreted reporter glycoprotein, Secreted Alkaline Phosphatase (SEAP), were observed in recombinant Chinese hamster ovary (CHO) cellsgrowing in increased concentrations of methotrexate. Western andRT-PCR analysis showed that this increase in volumetric titers is associated with higher levels of GR expressed in CHO cellsgrowing in increased concentration of methotrexate. A stablytransfected cell line growing in 10(-6) M methotrexate wasgrown in suspension culture and induced with 10(-7) Mdexamethasone. The SEAP volumetric titers reached a peak of approximately 23 mug ml(-1) on the 5th day after induction.Inducing these cells with increasing concentrations of dexamethasone resulted in increased specific productivity. These high volumetric productivities were further increased in fed-batch bioreactors.

319 ||| 7579667 ||| (2, 0.58) ||| 2 ||| 2 ||| Production of monoclonal antibodies in COS and CHO cells. Recent advances in the generation of genetically engineered monoclonal antibodies have enhanced the importance of COS cells as expression systems for rapidly producing sufficient quantities of these proteins for preliminary biochemical and biophysical analysis. In order to meet the demand for clinical supplies, a gradual increase has occurred in the usage of dihydrofolate reductase negative (DHFR-) Chinese hamster ovary (CHO) cells for large-scale antibody production. Using a variety of mammalian expression vectors and selection/amplification protocols, CHO cell lines capable of producing monoclonal antibodies at levels exceeding 1 gl-1 can now be obtained in an almost routine fashion. For the applications of monoclonal antibodies to expand into additional therapeutic areas, however, a 5-10-fold increase over current highest expression levels may still need to be achieved.

320 ||| 23994266 ||| (2, 0.58) ||| 2 ||| 2 ||| Development of a highly-efficient CHO cell line generation system with engineered SV40E promoter. Chinese hamster ovary (CHO) cells have been one of the most widely used host cells for the manufacture of therapeutic recombinant proteins. An effective and efficient clinical cell line development process, which could quickly identify those rare, high-producing cell lines among a large population of low and non-productive cells, is of considerable interest to speed up biological drug development. In the glutamine synthetase (GS)-CHO expression system, selection of top-producing cell lines is based on controlling the balance between the expression level of GS and the concentration of its specific inhibitor, l-methionine sulfoximine (MSX). The combined amount of GS expressed from plasmids that have been introduced through transfection and the endogenous CHO GS gene determine the stringency and efficiency of selection. Previous studies have shown significant improvement in selection stringency by using GS-knockout CHO cells, which eliminate background GS expression from the endogenous GS gene in CHOK1SV cells. To further improve selection stringency, a series of weakened SV40E promoters have been generated and used to modulate plasmid-based GS expression with the intent of manipulating GS-CHO selection, finely adjusting the balance between GS expression and GS inhibitor (MSX) levels. The reduction of SV40E promoter activities have been confirmed by TaqMan RT-PCR and GFP expression profiling. Significant productivity improvements in both bulk culture and individual clonal cell line have been achieved with the combined use of GS-knockout CHOK1SV cells and weakened SV40E promoters driving GS expression in the current cell line generation process. The selection stringency was significantly increased, as indicated by the shift towards higher distribution of producing-cell populations, even with no MSX added into cell culture medium. The potential applications of weakened SV40E promoter and GS-knockout cells in development of targeted integration and transient CHO expression systems are also discussed.

321 ||| 23881539 ||| (2, 0.58) ||| 2 ||| 2 ||| Stable expression of H1C2 monoclonal antibody in NS0 and CHO cells using pFUSE and UCOE expression system. From our recent publications, it was found that the deimmunization method (Dharshanan et al. (2012) Sci Res Essays 7:2288-2299) should be applied for the development of humanized anti-C2 monoclonal antibody (H1C2 mAb). However, the overlapping-PCR mutagenesis procedure used to insert the variable regions into cloning vectors was laborious and time-consuming. Additionally, the expression of H1C2 mAb in NS0 cells was low in static culture vessels. Therefore H1C2 mAb was redeveloped by deimmunization method with the following modifications in order to optimize the production of H1C2 mAb. First, instead of the overlapping-PCR mutagenesis procedure, synthetic DNA coding the variable regions were used to express the mAb. Second, two expression vectors, pFUSE and UCOE, were used to express H1C2 mAb in NS0 cells and CHO cells in order to investigate the combination that gave the highest number of high producing stable clones. This will provide the highest chance of finding clones with the requisite high productivity and stability required for manufacturing. We found that transfection of UCOE in CHO cells generated the highest number of high producing stable clones. To our knowledge, this is the first time that H1C2 mAb has been expressed in CHO cells.

322 ||| 22896849 ||| (2, 0.58) ||| 2 ||| 2 ||| Determination of Chinese hamster ovary cell line stability and recombinant antibody expression during long-term culture. Chinese hamster ovary (CHO) cell lines are frequently used as hosts for the production of recombinant therapeutics, such as monoclonal antibodies, due to their ability to perform correct post-translational modifications. A potential issue when utilizing CHO cells for therapeutic protein production is the selection of cell lines that do not retain stable protein expression during long-term culture (LTC). Instability of expression impairs process yields, effective usage of time and money, and regulatory approval for the desired therapeutic. In this study, we investigated a model unstable GS-CHO cell line over a continuous period of approximately 100 generations to determine markers of mechanisms that underlie instability. In this cell line, stability of expression was retained for 40-50 generations after which time a 40% loss in antibody production was detected. The instability observed within the cell line was not due to a loss in recombinant gene copy number or decreased expression of mRNA encoding for recombinant antibody H or L chain, but was associated with lower cumulative cell time values and an apparent increased sensitivity to cellular stress (exemplified by increased mRNA expression of the stress-inducible gene GADD153). Changes were also noted in cellular metabolism during LTC (alterations to extracellular alanine accumulation, and enhanced rates of glucose and lactate utilization, during the exponential and decline phase of batch culture, respectively). Our data indicates the breadth of changes that may occur to recombinant CHO cells during LTC ranging from instability of recombinant target production at a post-mRNA level to metabolic events. Definition of the mechanisms, regulatory events, and linkages underpinning cellular phenotype changes require further detailed analysis at a molecular level.

323 ||| 16635796 ||| (2, 0.58) ||| 2 ||| 2 ||| Xbp1-based engineering of secretory capacity enhances the productivity of Chinese hamster ovary cells. A variety of successful transcription and translation engineering strategies implemented during the past decade have driven the specific productivity of mammalian cells to an apparent limit. Restricted post-translation competence has since been considered the major bottleneck preventing mammalian cells from fully exploiting their physiologic production capacity in a biopharmaceutical manufacturing scenario. Through ectopic expression of the human transcription factor Xbp1 (X-box-binding-protein 1), evolved to manage plasma cell differentiation and coordinate the unfolded protein response, we have specifically expanded the endoplasmic reticulum and the Golgi of transgenic Chinese hamster ovary (CHO-K1)-derived cell lines with a resulting increase in overall production capacity. Xbp-1-based engineering of secretory bottlenecks was compatible with a variety of different promoter-product gene configurations suggesting that Xbp-1 induces generic production increases in CHO-K1 cell derivatives. Secretion engineering, illustrated here by Xbp1-based reprogramming of the post-translational processing machinery, provides a first insight into mastering a major system bottleneck which impacts biopharmaceutical manufacturing of secreted protein therapeutics.

324 ||| 9919309 ||| (2, 0.57) ||| 2 ||| 2 ||| Targeted recombination at the Chinese hamster APRT locus using insertion versus replacement vectors. In this study, we have examined the effects of targeting vector configuration and site of vector linearization on the frequency of targeted recombination at the endogenous CHO APRT locus, and have analyzed the types and class distributions of APRT+ recombinants obtained in APRT targeting experiments employing uncut circular, insertion-type (ends-in), and replacement-type (ends-out) configurations of the same pAG7 targeting vector, including configurations produced by introduction of a double-strand break (DSB) at sites either within, or at the 5' or 3' boundaries of APRT targeting homology. Our results suggest that: 1) plasmid-chromosome targeted recombination in mammalian cells may not be stimulated to the same degree by a DSB in the targeting vector as by a DSB in the chromosomal target; 2) recombinant class distributions are highly dependent upon targeting vector configuration; and 3) one-sided invasion mechanisms may play a significant role in homologous recombination in mammalian cells.

325 ||| 22068683 ||| (2, 0.57) ||| 2 ||| 2 ||| Early prediction of instability of Chinese hamster ovary cell lines expressing recombinant antibodies and antibody-fusion proteins. One of the most important criteria for the successful manufacture of a therapeutic protein (e.g., an antibody) is to develop a mammalian cell line that maintains stability of production. Problems with process yield, lack of effective use of costly resources, and a possible delay in obtaining regulatory approval of the product may ensue otherwise. Therefore the stability of expression in a number of Chinese hamster ovary (CHO) derived production cell lines that were isolated using the glutamine synthetase (GS) selection system was investigated by defining a culture as unstable if the titer (which is a measure of productivity) of a cell line expressing an antibody or antibody-fusion protein declined by 20-30% or more as it underwent 55 population doublings. Using this criterion, a significant proportion of the GS-selected CHO production cell lines were observed to be unstable. Reduced antibody titers correlated with the gradual appearance of a secondary, less productive population of cells as detected with flow cytometric analysis of intracellular antibody content. Where tested, it was observed that the secondary population arose spontaneously from the parental population following multiple passages, which suggested inherent clonal instability. Moreover, the frequency of unstable clones decreased significantly if the host cell line from which the candidate production cell lines were derived was apoptotic-resistant. This data suggested that unstable cell lines were more prone to apoptosis, which was confirmed by the fact that unstable cell lines had higher levels of Annexin V and caspase 3 activities. This knowledge has been used to develop screening protocols that identify unstable CHO production cell lines at an early stage of the cell line development process, potentially reducing the cost of biotherapeutic development.

326 ||| 18636602 ||| (2, 0.57) ||| 2 ||| 2 ||| A novel cytostatic process enhances the productivity of Chinese hamster ovary cells. We have established a novel production process which allows up to fourfold higher production of a model secreted protein, the human secreted alkaline phosphatase (SEAP), in Chinese hamster ovary (CHO) cells. A cytostatic production phase is established in which cell proliferation is inhibited or completely abolished. Such a cytostatic production phase is established by overexpression of the tumor suppressor genes p21, p27, or p53175P (a p53 mutant showing specific loss of apoptotic function) under transcriptional control of a tetracycline-repressible promoter (P(hCMV\*-1)). In order to minimize complications due to possible clonal variation of selected, stable cell lines, our investigations are based on transiently transfected subpopulations, that have become a useful tool in industrial R&D. These subpopulations have been selected by flow cytometry for the expression of genes encoded on a dicistronic expression vector. These vectors contain a dicistronic expression unit consisting of the genes encoding the green fluorescent protein (GFP) or SEAP, followed by one of the cytostatic genes p21, p27 or p53175P encoded by the second cistron. p21, p27 as well as p53175P block the cell cycle of CHO cells in the G1-phase for a prolonged period. However, these G1-arrested cells remain viable and proliferation proficient upon repression of expression of the cytostatic gene. All three of the cytostatic genes studied provided similar regulation of proliferation, and also similar enhancements in SEAP production, suggesting that higher productivity may be a general and intrinsic feature of G1-phase arrested CHO cells. Overall productivity is most likely enhanced because growth-arrested cells do not need to devote cellular resources to biomass production.

327 ||| 18220408 ||| (2, 0.57) ||| 2 ||| 2 ||| Mild hypothermia improves transient gene expression yields several fold in Chinese hamster ovary cells. Large-scale transient gene expression (TGE) in mammalian cells is a rapid method to generate recombinant proteins, but the volumetric productivity for secreted proteins is still more than an order of magnitude lower than the yields typically achieved with recombinant cell lines. Here transient recombinant protein production in Chinese hamster ovary cells transfected with linear 25 kDa polyethylenimine was significantly enhanced by incubation of the cells at temperatures ranging from 29 to 33 degrees C after DNA delivery. With this approach, transient recombinant antibody yields of 60-80 mg/L were achieved within 6 days of transfection. The increase in TGE correlated with the accumulation of cells in the G1 phase of the cell cycle, increased cell size, higher cell viability, higher steady-state levels of transgene mRNA, reduced consumption of nutrients, and decreased accumulation of waste products. The enhancement of TGE was not vector-dependent, but the presence of the woodchuck hepatitis virus post-transcriptional regulatory element in the 3' untranslated region of the transgene mRNA increased transient recombinant antibody expression more than 3-fold at 31 degrees C as compared to expression at 37 degrees C. The yields achieved by the low-temperature enhancement of TGE in CHO cells makes this technology feasible for the rapid production of gram amounts of secreted recombinant proteins at large scale (up to 100 L).

328 ||| 10099478 ||| (2, 0.57) ||| 2 ||| 2 ||| Clonal variability within dihydrofolate reductase-mediated gene amplified Chinese hamster ovary cells: stability in the absence of selective pressure. Recombinant Chinese hamster ovary (rCHO) cells expressing a high level of chimeric antibody were obtained by cotransfection of heavy- and light-chain cDNA expression vectors into dihydrofolate reductase-deficient CHO cells and subsequent gene amplification in medium containing stepwise increments in methotrexate (MTX) level up to 1.0 microM. To determine the clonal variability within the amplified cell population in regard to antibody production stability, 20 subclones were randomly isolated from the amplified cell population at 1.0 microM MTX (CS13-1.0 cells). Clonal analysis showed that CS13-1.0 cells were heterogeneous with regard to specific growth rate (mu) and specific antibody productivity (qAb), although they were derived from a single clone. The mu and qAb of 20 subclones were in the range of 0.51 to 0.72 day-1 and 10.9 to 19.1 microgram/10(6) cells/day, respectively. During 8 weeks of cultivation in the absence of selective pressure, the mu of most subclones did not change significantly. On the other hand, their qAb decreased significantly. Furthermore, the relative decrease in qAb varied among subclones, ranging from 30% to 80%. Southern and Northern blot analyses showed that this decreased qAb resulted mainly from the loss of amplified immunoglobulin (Ig) gene copies and their respective cytoplasmic mRNAs. For the sake of screening convenience, an attempted was made to correlate the initial properties of subclones (such as mu, qAb, and Ig gene copies) with their antibody production stability during long-term culture. Among these initial properties examined, only qAb of subclones could help to predict their stability to some extent. The subclones with high qAb were relatively stable with regard to antibody production during long-term culture in the absence of selective pressure (P < 0. 005, ANOVA). Taken together, the clonal heterogeneity in an amplified CHO cell population necessitates clonal analysis for screening stable clones with high qAb.

329 ||| 25444873 ||| (2, 0.56) ||| 2 ||| 2 ||| Overexpression of Serpinb1 in Chinese hamster ovary cells increases recombinant IgG productivity. We report the discovery and validation of a novel CHO cell engineering target for improving IgG expression, serpin peptidase inhibitor, clade B, member 1 (Serpinb1). Transcriptomic studies using microarrays revealed that Serpinb1 was up-regulated in cultures with IgG heavy and light chain transcription transiently repressed compared with cultures treated with non-targeting siRNA. As proof of concept, a lentiviral vector was employed to overexpress the Chinese Hamster Serpinb1 in a CHOZN( ) Glutamine Synthetase (-/-) recombinant IgG producing CHO line. The lentiviral stable pool demonstrated 4.2-fold SERPINB1 overexpression compared with the non-transduced control. The peak viable cell density (VCD) and peak IgG volumetric productivity of the lentiviral stable pool increased 1.3 and 2.0 fold, respectively, compared with the non-transduced control. For host cell engineering, a plasmid encoding SERPINB1 was transfected into the CHOZN( ) GS (-/-) host cell line to create several stable pools. Single-cell clones isolated from the pools were characterized for their SERPINB1 expression levels and growth. The clone (SERPINB1\_OE\_27) with the highest SERPINB1 expression had decreased peak viable cell density and exponential phase growth rate. Selected SERPINB1 OE clones were subsequently evaluated for their IgG expression capabilities using GS selection. Clone SERPINB1\_OE\_42 with moderate SERPINB1 overexpression demonstrated increased IgG productivity in "bulk" selection. We conclude that manipulating Serpinb1 expression can lead to increased recombinant IgG productivity, but the effect in host cell lines may vary by clone and by overexpression level. This work represents the ongoing effort in applying "-omics" findings to novel CHO host cell line engineering.

330 ||| 25887056 ||| (2, 0.55) ||| 2 ||| 2 ||| Sequencing the CHO DXB11 genome reveals regional variations in genomic stability and haploidy. BACKGROUND: The DHFR negative CHO DXB11 cell line (also known as DUX-B11 and DUKX) was historically the first CHO cell line to be used for large scale production of heterologous proteins and is still used for production of a number of complex proteins. RESULTS: Here we present the genomic sequence of the CHO DXB11 genome sequenced to a depth of 33x. Overall a significant genomic drift was seen favoring GC AT point mutations in line with the chemical mutagenesis strategy used for generation of the cell line. The sequencing depth for each gene in the genome revealed distinct peaks at sequencing depths of 0x, 16x, 33x and 49x coverage corresponding to a copy number in the genome of 0, 1, 2 and 3 copies. This indicate that 17% of the genes are haploid revealing a large number of genes which can be knocked out with relative ease. This tendency of haploidy was furthermore shown to be present in eight additional analyzed CHO genomes (15-20% haploidy) but not in the genome of the Chinese hamster. The dhfr gene is confirmed to be haploid in CHO DXB11; transcriptionally active and the remaining allele contains a G410C point mutation causing a Thr137Arg missense mutation. We find ~2.5 million single nucleotide polymorphisms (SNP's), 44 gene deletions in the CHO DXB11 genome and 9357 SNP's, which interfere with the coding regions of 3458 genes. Copy number variations for nine CHO genomes were mapped to the chromosomes of the Chinese hamster showing unique signatures for each chromosome. The data indicate that chromosome one and four appear to be more stable over the course of the CHO evolution compared to the other chromosomes thus might presenting the most attractive landing platforms for knock-ins of heterologous genes. CONCLUSIONS: Our studies reveal an unexpected degree of haploidy in CHO DXB11 and CHO cells in general and highlight the chromosomal changes that have occurred among the CHO cell lines sequenced to date.

331 ||| 23380542 ||| (2, 0.55) ||| 2 ||| 2 ||| CHO cell engineering to prevent polypeptide aggregation and improve therapeutic protein secretion. The ability to efficiently produce recombinant proteins in a secreted form is highly desirable and cultured mammalian cells such as CHO cells have become the preferred host as they secrete proteins with human-like post-translational modifications. However, attempts to express high levels of particular proteins in CHO cells may consistently result in low yields, even for non-engineered proteins such as immunoglobulins. In this study, we identified the responsible faulty step at the stage of translational arrest, translocation and early processing for such a "difficult-to-express" immunoglobulin, resulting in improper cleavage of the light chain and its precipitation in an insoluble cellular fraction unable to contribute to immunoglobulin assembly. We further show that proper processing and secretion were restored by over-expressing human signal receptor protein SRP14 and other components of the secretion pathway. This allowed the expression of the difficult-to-express protein to high yields, and it also increased the production of an easy-to-express protein. Our results demonstrate that components of the secretory and processing pathways can be limiting, and that engineering of the secretory pathway may be used to improve the secretion efficiency of therapeutic proteins from CHO cells.

332 ||| 23096947 ||| (2, 0.55) ||| 2 ||| 2 ||| IL-17F co-expression improves cell growth characteristics and enhances recombinant protein production during CHO cell line engineering. The generation of a high productivity cell line is a critical step in the production of a therapeutic protein. Many innovative engineering strategies have been devised in order to maximize the expression rate of production cells for increased process efficiency. Less effort has focused on improvements to the cell line generation process, which is typically long and laborious when using mammalian cells. Based on unexpected findings when generating stable CHO cell lines expressing human IL-17F, we studied the benefit of expressing this protein during the establishment of production cell lines. We demonstrate that IL-17F expression enhances the rate of selection and overall number of selected cell lines as well as their transgene expression levels. We also show that this benefit is observed with different parental CHO cell lines and selection systems. Furthermore, IL-17F expression improves the efficiency of cell line subcloning processes. IL-17F can therefore be exploited in a standard manufacturing process to obtain higher productivity clones in a reduced time frame.

333 ||| 22210617 ||| (2, 0.55) ||| 2 ||| 2 ||| Combined TGE-SGE expression of novel PAI-1-resistant t-PA in CHO DG44 cells using orbitally shaking disposable bioreactors. An important modification of thrombolytic agents is resistance to plasminogen activator inhibitor-1 (PAI-1). In previous studies, a new truncated PAI-1-resistant variant was developed based on deletion of the first three domains in t-PA and the substitution of KHRR 128-131 amino acids with AAAA in the truncated t-PA. The novel variant expressed in a static culture system of Chinese Hamster Ovary (CHO) DG44 cells exhibited a higher resistance to PAI-1 when compared with the full-length commercial drug; Actylase. In the present study, the truncatedmutant protein was expressed in CHO DG44 cells in 50 ml orbital shaking bioreactors. The final yield of the truncatedmutant in the culture was 752 IU/ml, representing a 63% increase compared with the static culture system. Therefore, these results suggest that using the combined features of a transient and stable expression system is feasible for the production of novel recombinant proteins in the quantities needed for preclinical studies.

334 ||| 10777536 ||| (2, 0.55) ||| 2 ||| 2 ||| Transcriptional regulation of the ATP citrate-lyase gene by sterol regulatory element-binding proteins. In an attempt to identify unknown target genes for SREBP-1, total RNA from a stable Chinese hamster ovary cell line (CHO-487) expressing a mature form of human SREBP-1a (amino acids 1-487) with a LacSwitch Inducible Mammalian Expression System was subjected to a polymerase chain reaction subtraction method. One of the fragments was found to have 90 and 86% homology with rat and human ATP citrate-lyase (ACL) cDNA, respectively. When Hep G2 cells are cultured under either sterol-loaded or -depleted conditions, expression of the gene is induced approximately 2-3-fold by sterol depletion. To investigate the direct effect of SREBP-1a on transcription, luciferase assays using the promoter of the human ACL gene were performed. These deletion studies indicated that a minimum 160-base pair segment contains the information required for the transcriptional regulation brought about by enforced expression of SREBP-1a. Luciferase assays using mutant reporter genes revealed that SREBP-dependent transcriptional regulation is mediated by two nearby motifs, the SREBP-binding site (a TCAGGCTAG sequence) and the NF-Y-binding site (a CCAAT box). It was confirmed by gel mobility shift assays that recombinant SREBP-1a binds to the sequence. Data from studies with transgenic mice and reporter assays show that the ACL gene promoter is activated by SREBP-1a more strongly than SREBP-2 in contrast to the HMG CoA synthase and LDL receptor gene promoters, which exhibit the same preference for the two factors. Therefore, SREBPs transcriptionally regulates ACL enzyme activity, which generates the cytosolic acetyl CoA required for both cholesterol and fatty acid synthesis.

335 ||| 23583871 ||| (2, 0.54) ||| 2 ||| 2 ||| Control of IgG LC:HC ratio in stably transfected CHO cells and study of the impact on expression, aggregation, glycosylation and conformational stability. Immunoglobulin G (IgG), the most common class of commercial monoclonal antibodies (mAbs), exists as multimers of two identical light chains (LC) and two identical heavy chains (HC) assembled together by disulfide bridges. Due to the kinetics of mAb assembly, it is suggested that expression of LC and HC in equal amounts is not optimal for IgG production. We designed a set of vectors using internal ribosome entry site (IRES) elements to control LC and HC expression. The intracellular LC:HC ratio of stable IgG expressing Chinese hamster ovary (CHO) cell pools can be controlled effectively at four different ratios of 3.43, 1.24, 1.12, and 0.32. The stable pools were used to study the impact of LC:HC ratio on mAb expression and quality. Gene amplification was most effective for pools with excess LC and generated the highest mAb titers among the transfected pools. When LC:HC ratio was greater than one, more than 97% of the secreted products were IgG monomers. The products also have similar N-glycosylation profiles and conformational stabilities at those ratios. For pools presented a lower LC:HC ratio of 0.32, monomers only constituted half of the product with the other half being aggregates and mAb fragments. High mannose-type N-glycans increased while fucosylated and galactosylated glycans decreased significantly at the lowest LC:HC ratio. Product stability was also adversely affected. The results obtained provide insights to the impact of different LC:HC ratios on stable mAb production and useful information for vector design during generation of mAb producing cell lines.

336 ||| 23300776 ||| (2, 0.54) ||| 2 ||| 2 ||| Production of functional soluble Dectin-1 glycoprotein using an IRES-linked destabilized-dihydrofolate reductase expression vector. Dectin-1 (CLEC7A) is a C-type lectin receptor that binds to beta-glucans found in fungal cell walls to act as a major pattern recognition receptor (PRR). Since beta-glucans epitope is not present in human cells, we are of the opinion that Dectin-1 can have therapeutic functions against fungal infections. We thus set out to produce a soluble extracellular domain of murine Dectin-1 (called sDectin-1) in sufficient titers to facilitate such studies in mouse models. Since sDectin-1 has previously been shown to be glycosylated, we chose to produce this protein using Chinese Hamster Ovary (CHO) cells, a mammalian host cell line suitable for the high-titer production of recombinant glycoproteins. To ensure a high titer production of sDectin-1 and minimize the effects of gene fragmentation, we constructed a mammalian expression vector with a PEST-destabilized dhfr amplifiable marker downstream of an attenuated IRES element, which was in turn downstream of the sDectin-1 gene and a CMV IE promoter. Stably transfected and MTX-amplified cell pools were generated using this vector, and maximum sDectin-1 titers of 246 mg/l and 598 mg/l were obtained in shake flask batch culture and bioreactor fed-batch culture respectively. The purified recombinant sDectin-1 was shown to be glycosylated. Protein functionality was also demonstrated by its ability to bind to zymosan particles and to the cell wall of Saccharomyces cerevisiae. We describe for the first time the use of an attenuated IRES-linked PEST-destabilized dhfr amplifiable marker for the production of recombinant proteins with stably amplified cell pools. With our process, we reached the highest reported titer for producing recombinant proteins smaller than 50 kDa in cell pools. sDectin-1 protein produced is glycosylated and functional. This vector design can thus be used efficiently for the high-titer production of functional recombinant proteins.

337 ||| 25641927 ||| (2, 0.53) ||| 2 ||| 2 ||| Chinese hamster ovary (CHO) host cell engineering to increase sialylation of recombinant therapeutic proteins by modulating sialyltransferase expression. N-Glycans of human proteins possess both alpha2,6- and alpha2,3-linked terminal sialic acid (SA). Recombinant glycoproteins produced in Chinese hamster overy (CHO) only have alpha2,3-linkage due to the absence of alpha2,6-sialyltransferase (St6gal1) expression. The Chinese hamster ST6GAL1 was successfully overexpressed using a plasmid expression vector in three recombinant immunoglobulin G (IgG)-producing CHO cell lines. The stably transfected cell lines were enriched for ST6GAL1 overexpression using FITC-Sambucus nigra (SNA) lectin that preferentially binds alpha2,6-linked SA. The presence of alpha2,6-linked SA was confirmed using a novel LTQ Linear Ion Trap Mass Spectrometry (LTQ MS) method including MSn fragmentation in the enriched ST6GAL1 Clone 27. Furthermore, the total SA (mol/mol) in IgG produced by the enriched ST6GAL1 Clone 27 increased by 2-fold compared to the control. For host cell engineering, the CHOZN( ) GS host cell line was transfected and enriched for ST6GAL1 overexpression. Single-cell clones were derived from the enriched population and selected based on FITC-SNA staining and St6gal1 expression. Two clones ("ST6GAL1 OE Clone 31 and 32") were confirmed for the presence of alpha2,6-linked SA in total host cell protein extracts. ST6GAL1 OE Clone 32 was subsequently used to express SAFC human IgG1. The recombinant IgG expressed in this host cell line was confirmed to have alpha2,6-linked SA and increased total SA content. In conclusion, overexpression of St6gal1 is sufficient to produce recombinant proteins with increased sialylation and more human-like glycoprofiles without combinatorial engineering of other sialylation pathway genes. This work represents our ongoing effort of glycoengineering in CHO host cell lines for the development of "bio-better" protein therapeutics and cell culture vaccine production.

338 ||| 21169054 ||| (2, 0.53) ||| 2 ||| 2 ||| Detailed understanding of enhanced specific productivity in Chinese hamster ovary cells at low culture temperature. The specific productivity of tumor necrosis factor receptor-immunoglobulin G1 Fc fusion (TNFR-Fc) (q(TNFR-Fc)) in Chinese hamster ovary (CHO) cells at 30 C was approximately 5-fold higher than that at 37 C. To investigate reasons for increased q(TNFR-Fc) at low culture temperature, TNFR-Fc mRNA levels were determined by real-time PCR. It was found that like q(TNFR-Fc), the relative TNFR-Fc mRNA level was increased by lowering culture temperature, and more importantly, the kinetics of the increase in TNFR-Fc mRNA levels were in accordance with the changes in q(TNFR-Fc). The results demonstrated that the increased transcriptional level of TNFR-Fc was responsible for the increased q(TNFR-Fc) at low culture temperature. Enhanced levels of mRNA could derive from increased gene copy number, improved mRNA stability, or enhanced transcriptional rate. There was not a big change of gene copy number by lowering culture temperature. The transcriptional rate of TNFR-Fc was slightly decreased at 30 C, compared to 37 C. However, mRNA stability of TNFR-Fc was significantly improved by lowering culture temperature. The half-life of TNFR-Fc mRNA was 5.55 h at 30 C, whereas that was 3.69h at 37 C. Taken together, the reasons for the increased q(TNFR-Fc) in CHO cells at low culture temperature were mainly the enhanced TNFR-Fc mRNA levels, which resulted from the improved mRNA stability, rather than the changes in the gene copy number or the transcriptional rate.

339 ||| 11853559 ||| (2, 0.53) ||| 2 ||| 2 ||| The unfolded protein response in a dolichyl phosphate mannose-deficient Chinese hamster ovary cell line points out the key role of a demannosylation step in the quality-control mechanism of N-glycoproteins. The CHO (Chinese hamster ovary) glycosylation mutant cell line, B3F7, transfers the truncated glycan Glc(3)Man(5)GlcNAc(2) on to nascent proteins. After deglucosylation, the resulting Man(5)GlcNAc(2) glycan is subjected to two reciprocal enzymic processes: the action of an endoplasmic-reticulum (ER) kifunensine-sensitive alpha1,2-mannosidase activity to yield a Man(4)GlcNAc(2) glycan, and the reglucosylation involved in the quality-control system which ensures that only correctly folded glycoproteins leave the ER. We show that the recombinant secreted alkaline phosphatase (SeAP) produced in stably transfected B3F7 cells, is co-immunoprecipitated with the GRP78 (glucose-regulated protein 78), a protein marker of the unfolded protein response (UPR). The level of GRP78 transcription has been evaluated by reverse transcription-PCR (RT-PCR) and we demonstrate that B3F7 cells present a constitutively higher level of UPR in the absence of inductors, compared with Pro(-5) cells. Interestingly, a decrease was observed in the UPR and an increase in SeAP secretion in the kifunensine-treated B3F7 cells. Altogether, these data highlight the relationships between the glycan structure, the quality control system and the UPR. Moreover, they support the idea that a specific demannosylation step is a key event of the glycoprotein quality control in B3F7 cells.

340 ||| 25215178 ||| (2, 0.52) ||| 2 ||| 2 ||| Effect of Peptone Feeding on Transient Gene Expression Process in CHO DG44. BACKGROUND: Transient Gene Expression (TGE) gained popularity over the last decade as a rapid method for the production of milligram to gram quantities of recombinant proteins for preclinical studies in biophama industry. Thereby, the optimization of the TGE technique for Chinese hamster ovary (CHO) as the dominant host for the production of biotherapeutics is of great interest to reach the values for Human Embryo Kidney-293 (HEK-293) cells in terms of transfection efficiencies and production titers. TGE efficiencies are cell line and vector dependant. METHODS: In transfection efficiency optimization experiments, different starting cell densities, different amounts of plasmid DNA and PEI transfection reagent were investigated to achieve the best conditions leading to maximum transfection efficiencies. Furthermore, in order to investigate the effect of peptone feeding on transfection efficiency, three different sources of peptones with the greatest effect in the CD DG44 basal media were selected; Casein Tryptone N1, Soy petone A2SC and Soy peptone E110. RESULTS: The transfection strategy performed here was able to make an outstanding increase in transfection efficiency of CHO DG44 cell line transfected with pTracer-SV40-mutated t-PA plasmid from 3.6% in our starting non-optimized condition to 66.93% in finally optimized situation. Moreover, peptone feeding strategy used here was successful to increase volumetric productivities up to 37%. In addition, the amounts of both PEI and plasmid DNA were reduced up to 66% and 25%, respectively compared to our previous protocol. CONCLUSION: Here we described an optimization process for TGE in suspension-adapted CHO cells based on Polyethylenimine (PEI)/DNA concentration, DNA: PEI ratio, starting cell densities and peptone feeding strategy.

341 ||| 21477625 ||| (2, 0.52) ||| 2 ||| 2 ||| Insect cells for antibody production: evaluation of an efficient alternative. In recent years there has been an increase in both availability and demand for therapeutic monoclonal antibodies. Currently, most of these antibodies are produced by stably transfected mammalian cells. In this study we evaluated the use of different baculoviral insect cell systems as an alternative for commonly used production schemes. We expressed the human anti-gp41 antibody 3D6 in Spodoptera frugiperda Sf9, Trichoplusia ni BTI-TN5B1-4 "High Five", and Spodoptera frugiperda SfSWT-1 "Mimic " insect cells and compared product yield, specificity and glycosylation patterns with a 3D6 antibody expressed in Chinese hamster ovary cells. Using "High Five" cells we achieved amounts of secreted antibody comparable to those resulting from transient expression in mammalian cells. We determined the N-linked oligosaccharide structures present on asparagine-297 in IgG1 heavy chains and tested the functionality in terms of antigen binding and the ability to elicit effector functions. Antibodies expressed in all insect cell lines displayed highly specific antigen binding. In general, the insect-produced antibodies carried, as the CHO-produced form, fucosylated N-glycans, including, in the case of "High Five" cells, high levels of core alpha1,3-fucose. This indicates that in all systems glycoengineering may be required in order to produce optimal glycoforms of this antibody.

342 ||| 9758659 ||| (2, 0.51) ||| 2 ||| 2 ||| Higher productivity of growth-arrested Chinese hamster ovary cells expressing the cyclin-dependent kinase inhibitor p27. We constructed stable Chinese hamster ovary (CHO) cell lines which conditionally and coordinately express the model product gene secreted alkaline phosphatase (SEAP) and one of the cytostatic genes p21, p27, and p53175P, a p53 mutant deficient in apoptotic but not cell-cycle arrest function. The use of dicistronic expression technology allowed the conditional expression of the model product gene and the cytostatic gene in a coordinated fashion from a single expression unit under the control of the tetracycline-responsive promoter PhCMV-1. Due to the presence of a cytostatic gene in the multicistronic expression unit, the growth behavior of the engineered CHO cell lines could be controlled by the addition or withdrawal of the exogenous agent tetracycline to or from the cell culture medium. Withdrawal of tetracycline resulted in sustained growth arrest of the stable cell lines for a prolonged period. The growth arrest of such cell lines was found to be accompanied by a 10-15-fold increase in their production of SEAP per cell. This controlled proliferation technology allows the design of a novel two-stage production process which consists of a proliferation phase leading to the desired cell density, followed by an extended production phase during which the cells remain growth-arrested and increase cell-specific production of a heterologous protein.

343 ||| 8634140 ||| (2, 0.51) ||| 2 ||| 2 ||| Plasmid insertional mutation may confer glucocorticoid responsiveness of cell growth. Culture of CHO.K1, a Chinese hamster ovary cell line, requires no particular care about the glucocorticoid level in media. Cell growth of CHO.K1 is little affected by dexamethasone at concentrations up to 3 microM. A clone of CHO.K1 stably transfected with an expression vector displayed a favored growth in dexamethasone-containing media. Ironically, dexamethasone was used to trigger the expression of antisense PCNA from the expression vector to impede the cell growth. Proliferating cell nuclear antigen (PCNA), an auxiliary factor of DNA polymerase delta, is required for cell proliferation. The stable cell clone, designated as B11 had a retarded growth rate as compared to its parental cell. However, the B11 cell growth rate and the cell cycle progression were increased by dexamethasone. The glucocorticoid produced no similar effect on the parental cell or other stable transfectants of the same plasmid. Thus, the stimulatory effect of dexamethasone on B11 has little connection with the expression of antisense PCNA and possibly involves a relevant gene in the B11 genome that was mutated due to the random plasmid insertion. A preliminary effort in identifying the targeted gene was made by using plasmid rescue method, and two plasmids were obtained. The rescued DNA of both plasmids specifically hybridized genomic DNA of the parental cells, and one of these plasmids detected a cellular transcript that was absent in B11 cells, suggesting its potential for further investigation.

344 ||| 25123359 ||| (2, 0.51) ||| 2 ||| 2 ||| Reduction in C-terminal amidated species of recombinant monoclonal antibodies by genetic modification of CHO cells. BACKGROUND: During development of recombinant monoclonal antibodies in Chinese hamster ovary (CHO) cells, C-terminal amidated species are observed. C-terminal amidation is catalysed by peptidylglycine alpha-amidating monooxygenase (PAM), an enzyme known to be expressed in CHO cells. The significant variations between clones during clone selection, and the relatively high content of amidated species (up to 15%) in comparison to reference material (4%), led us to develop a cell line with reduced production of C-terminal amidated monoclonal antibodies using genetic manipulation. RESULTS: Initial target validation was performed using the RNA interference approach against PAM, which resulted in a CHO cell line with C-terminal amidation decreased to 3%. Due to the transient effects of small-interfering RNAs, and possible stability problems using short-hairpin RNAs, we knocked-down the PAM gene using zinc finger nucleases. Plasmid DNA and mRNA for zinc finger nucleases were used to generate a PAM knock-out, which resulted in two CHO cell lines with C-terminal amidation decreased to 6%, in CHO Der2 and CHO Der3 cells. CONCLUSION: Two genetically modified cell lines were generated using a zinc finger nuclease approach to decrease C-terminal amidation on recombinant monoclonal antibodies. These two cell lines now represent a pool from which the candidate clone with the highest comparability to the reference molecule can be selected, for production of high-quality and safe therapeutics.

345 ||| 23748758 ||| (2, 0.51) ||| 2 ||| 2 ||| Maximizing antibody production in suspension-cultured mammalian cells by the customized transient gene expression method. Due to the great diversity in protein expression productivity, a customized transient gene expression (TGE) method was used in the present study to optimize transient expression of three antibodies. Several factors, including host cells, temperature, valproic acid (VPA) treatment, various vectors, and additives were optimized independently and then combined to form a customized TGE protocol for each antibody. In the event, the optimized TGE conditions for three antibodies were different from each other. Compared with the TGE in CHO-S cells by pCDNA3.1 expression vector, the expression productivities of 8C11 cAb, 37 hAb, and 10F7 cAb showed 16-fold, 293-fold, and 19-fold increases respectively by the customized TGE method. For 8C11 cAb, coexpressing L-chain and H-chain on different plasmids led to higher yields. The customized TGE method is an alternative approach that can greatly improve the expression productivity of a variety of recombinant proteins.

346 ||| 23091360 ||| (2, 0.51) ||| 2 ||| 2 ||| Generation of a Chinese hamster ovary cell line producing recombinant human glucocerebrosidase. Impaired activity of the lysosomal enzyme glucocerebrosidase (GCR) results in the inherited metabolic disorder known as Gaucher disease. Current treatment consists of enzyme replacement therapy by administration of exogenous GCR. Although effective, it is exceptionally expensive, and patients worldwide have a limited access to this medicine. In Brazil, the public healthcare system provides the drug free of charge for all Gaucher's patients, which reaches the order of $ 84 million per year. However, the production of GCR by public institutions in Brazil would reduce significantly the therapy costs. Here, we describe a robust protocol for the generation of a cell line producing recombinant human GCR. The protein was expressed in CHO-DXB11 (dhfr(-)) cells after stable transfection and gene amplification with methotrexate. As expected, glycosylated GCR was detected by immunoblotting assay both as cell-associated (~64 and 59 kDa) and secreted (63-69 kDa) form. Analysis of subclones allowed the selection of stable CHO cells producing a secreted functional enzyme, with a calculated productivity of 5.14 pg/cell/day for the highest producer. Although being laborious, traditional methods of screening high-producing recombinant cells may represent a valuable alternative to generate expensive biopharmaceuticals in countries with limited resources.

347 ||| 11091196 ||| (2, 0.51) ||| 2 ||| 2 ||| CHO expression of a novel human recombinant IgG1 anti-RhD antibody isolated by phage display. Replacement of the hyperimmune anti-Rhesus (Rh) D immunoglobulin, currently used to prevent haemolytic disease of the newborn, by fully recombinant human anti-RhD antibodies would solve the current logistic problems associated with supply and demand. The combination of phage display repertoire cloning with precise selection procedures enables isolation of specific genes that can then be inserted into mammalian expression systems allowing production of large quantities of recombinant human proteins. With the aim of selecting high-affinity anti-RhD antibodies, two human Fab libraries were constructed from a hyperimmune donor. Use of a new phage panning procedure involving bromelin-treated red blood cells enabled the isolation of two high-affinity Fab-expressing phage clones. LD-6-3 and LD-6-33, specific for RhD. These showed a novel reaction pattern by recognizing the D variants D(III), D(IVa), D(IVb), D(Va), D(VI) types I and II. D(VII), Rh33 and DFR. Full-length immunoglobulin molecules were constructed by cloning the variable regions into expression vectors containing genomic DNA encoding the immunoglobulin constant regions. We describe the first, stable, suspension growth-adapted Chinese hamster ovary (CHO) cell line producing a high affinity recombinant human IgG1 anti-RhD antibody adapted to pilot-scale production. Evaluation of the Fc region of this recombinant antibody by either chemiluminescence or antibody-dependent cell cytotoxicity (ADCC) assays demonstrated macrophage activation and lysis of red blood cells by human lymphocytes. A consistent source of recombinant human anti-RhD immunoglobulin produced by CHO cells is expected to meet the stringent safety and regulatory requirements for prophylactic application.

348 ||| 9634782 ||| (2, 0.50) ||| 2 ||| 2 ||| Production of recombinant proteins in Chinese hamster ovary cells using a protein-free cell culture medium. The growth-factor prototrophic Chinese hamster ovary (CHO) SSF3 cell line was previously adapted for growth in serum-free media. Here we present a newly designed medium which allows these cells to grow in the absence of any exogenously added growth factors. To investigate the capacity of CHO SSF3 cells for the efficient production of recombinant proteins in protein-free media, expression plasmids containing either a human single chain urokinase-type plasminogen activator (uPA)-encoding cDNA or a humanized immunoglobulin G (IgG) kappa light chain cDNA were introduced by transfection. The tryptophan synthase (trpB) gene of Escherichia coli was used as a dominantly acting selection marker allowing the cells to survive in a medium containing indole in place of tryptophan. Some of the clones obtained exhibited a stable uPA expression over a period of several months under selective conditions and the yields were up to 74 mg of uPA/l in a bioreactor and the productivity was around 40 mg/day per 10(9) cells. The yields of IgG light chains were up to 118 mg/l and the productivity was in the order of 56 mg/day per 10(9) cells in a bioreactor. These results demonstrate the potential of CHO SSF3 cells for the efficient production of recombinant proteins under protein-free conditions.

349 ||| 22358882 ||| (2, 0.50) ||| 2 ||| 2 ||| Erythropoietin enhancer stimulates production of a recombinant protein by Chinese hamster ovary (CHO) cells under hypoxic condition. Oxygen is a limiting nutrient in animal cell culture and its supply is still worthy of improvement for production of useful proteins with a high efficiency. From a different point of view, development of the system by which a high productivity can be maintained even under hypoxic condition as well as under normoxic condition may be important. A number of hypoxia-inducible genes have been found in eucaryotic cells and the induction in most cases, if not all, is due to hypoxic activation of the gene transcription. Transcription of erythropoietin gene is highly hypoxia-inducible and the induction is achieved by binding of a protein, which is widely distributed in animal cells, to a short DNA stretch (erythropoietin enhancer) in the 3' flanking region of erythropoietin gene. Using a hepatoma cell line (Hep3B) that produces the endogenous erythropoietin in an oxygen-dependent manner and Chinese hamster ovary cells that have been widely used for production of recombinant proteins, we show that, under hypoxic condition, the erythropoietin enhancer can activate not only the promoter of erythropoietin gene but also promoters of cytomegalovirus early genes and eucaryotic polypeptide chain elongation factor gene, both of which are very active in animal cells under normoxic condition.

350 ||| 19003404 ||| (2, 0.50) ||| 2 ||| 2 ||| Isolation, characterization and recombinant protein expression in Veggie-CHO: A serum-free CHO host cell line. The dihydrofolate reductase-deficient Chinese hamster ovary cell line, DXB11-CHO, commonly used as a host cell for the production of recombinant proteins requires 7.5% serum-supplementation for optimal growth. Regulatory issues surrounding the use of serum in clinical production processes and the direct and indirect costs of using serum in large-scale production and recovery processes have triggered efforts to derive serum-independent host cell lines. We have successfully isolated a serum-free host that we named Veggie- CHO. Veggie-CHO was generated by adapting DXB11-CHO cells to growth in serum-free media in the absence of exogenous growth factors such as Transferrin and Insulin-like growth factor, which we have previously shown to be essential for growth and viability of DXB11- CHO cells. Veggie-CHO cells have been shown to maintain an average doubling time of 22 hr in continuous growth cultures over a period of three months and have retained the dihydrofolate reductase -deficient phenotype of their parental DXB11-CHO cells. These properties and the stability of its serum-free phenotype have allowed the use of Veggie- CHO as host cells for transfection and amplified expression of recombinant proteins. We describe the derivation a serum-free recombinant cell line with an average doubling time of 20 hr and specific productivity of 2.5 Units recombinant Flt-3L protein per 10e6 cells per day.

351 ||| 10677373 ||| (2, 0.49),(4, 0.45) ||| 2 ||| 2 ||| Mutations in the reduced-folate carrier affect protein localization and stability. The reduced-folate-carrier (rfc) gene has been shown to be functionally important for reduced-folate transport in mammalian cells. In the present paper we describe the identification of alterations in both alleles of the rfc gene in a mutant Chinese-hamster ovary cell line deficient in methotrexate transport. One allele of the rfc gene contains a point mutation resulting in a Gly(345)-->Arg substitution in the predicted amino acid sequence. In this case, a protein of similar size to the wild-type protein is produced, although it remains as an immature, core-glycosylated, form. The second allele contains a point mutation in the last base of intron 5 that results in the utilization of a cryptic splice site leading to a seven-base deletion in the mRNA. The use of an alternate splice site changes the reading frame to yield a truncated protein with 68 different C-terminal amino acids as compared with the wild-type. Both of these altered gene products were monitored by fusion with green fluorescent protein and found to be non-functional with an increased rate of turnover. The protein with the point mutation is trapped in the endoplasmic reticulum with subsequent degradation, whereas the product of the splice mutation is not membrane-associated and is partially degraded. Thus mutations in both alleles of the rfc gene in this resistant cell line account for the loss of reduced-folate transport. The observations made regarding the degradation of these mutant gene products also provide support for putative checkpoints in the endoplasmic reticulum.

352 ||| 21569433 ||| (2, 0.49),(1, 0.40) ||| 2 ||| 2 ||| Identification of a novel temperature sensitive promoter in CHO cells. BACKGROUND: The Chinese hamster ovary (CHO) expression system is the leading production platform for manufacturing biopharmaceuticals for the treatment of numerous human diseases. Efforts to optimize the production process also include the genetic construct encoding the therapeutic gene. Here we report about the successful identification of an endogenous highly active gene promoter obtained from CHO cells which shows conditionally inducible gene expression at reduced temperature. RESULTS: Based on CHO microarray expression data abundantly transcribed genes were selected as potential promoter candidates. The S100a6 (calcyclin) and its flanking regions were identified from a genomic CHO-K1 lambda-phage library. Computational analyses showed a predicted TSS, a TATA-box and several TFBSs within the 1.5 kb region upstream the ATG start signal. Various constructs were investigated for promoter activity at 37 C and 33 C in transient luciferase reporter gene assays. Most constructs showed expression levels even higher than the SV40 control and on average a more than two-fold increase at lower temperature. We identified the core promoter sequence (222 bp) comprising two SP1 sites and could show a further increase in activity by duplication of this minimal sequence. CONCLUSIONS: This novel CHO promoter permits conditionally high-level gene expression. Upon a shift to 33 C, a two to three-fold increase of basal productivity (already higher than SV40 promoter) is achieved. This property is of particular advantage for a process with reduced expression during initial cell growth followed by the production phase at low temperature with a boost in expression. Additionally, production of toxic proteins becomes feasible, since cell metabolism and gene expression do not directly interfere. The CHO S100a6 promoter can be characterized as cold-shock responsive with the potential for improving process performance of mammalian expression systems.

353 ||| 21618471 ||| (2, 0.49),(1, 0.34) ||| 2 ||| 2 ||| Transforming growth factor-beta1 is constitutively secreted by Chinese hamster ovary cells and is functional in human cells. Chinese hamster ovary (CHO) cells are widely used for the production of recombinant proteins for clinical use as well as academic research. They are particularly important for the production of glycoproteins where bacteria cannot be used. TGFbeta1 is a potent cytokine highly conserved across species with multiple immunological and non-immunological effects. We have discovered that CHOK1, the CHO clone most commonly used by the pharmaceutical industry, constitutively secretes latent TGFbeta1 and that this hamster TGFbeta1 is active on human cells inducing profound immunological effects. As far as we are aware, the production of TGFbeta1 by CHOK1 cells has not been reported before in the literature. As TGFbeta1 exerts powerful and pleiotropic effects on diverse cell types, and as CHO cells are used to produce a large number of clinical and non-clinical products, our findings are highly relevant to studies that rely on recombinant proteins.

354 ||| 24216459 ||| (2, 0.49),(1, 0.30) ||| 2 ||| 2 ||| Single-cell-based breeding: Rational strategy for the establishment of cell lines from a single cell with the most favorable properties. For efficient biomolecule production (e.g., antibodies, recombinant proteins), mammalian cells with high expression rates should be selected from cell libraries, propagated while maintaining a homogenous expression rate, and subsequently stabilized at their high expression rate. Clusters of isogenic cells (i.e., colonies) have been used for these processes. However, cellular heterogeneity makes it difficult to obtain cell lines with the highest expression rates by using single-colony-based breeding. Furthermore, even among the single cells in an isogenic cell population, the desired cell properties fluctuate stochastically during long-term culture. Therefore, although the molecular mechanisms underlying stochastic fluctuation are poorly understood, it is necessary to establish excellent cell lines in order to breed single cells to have higher expression, higher stability, and higher homogeneity while suppressing stochastic fluctuation (i.e., single-cell-based breeding). In this review, we describe various methods for manipulating single cells and facilitating single-cell analysis in order to better understand stochastic fluctuation. We demonstrated that single-cell-based breeding is practical and promising by using a high-throughput automated system to analyze and manipulate single cells.

355 ||| 9838208 ||| (2, 0.49) ||| 2 ||| 2 ||| Stable expression of human alpha-2,6-sialyltransferase in Chinese hamster ovary cells: functional consequences for human erythropoietin expression and bioactivity. Hamster cell lines are common hosts for recombinant protein production, e.g. erythropoietin (Epo). Terminal sialylation of native human proteins is characteristically in both alpha-2,3 and alpha-2,6 linkage to galactose at the termini of N-linked oligosaccharides but only in alpha-2,3 linkage in recombinant proteins expressed in hamster cells which do not express alpha-2, 6-sialyltransferase (ST6GalI) (EC 2.4.99.1). This difference could alter the bioactivity of certain recombinant proteins. Chinese hamster ovary (CHO) cells stably transfected with human ST6GalI cDNA linked to the hamster metallothionein II promoter expressed highly inducible authentic ST6GalI activity. Untransfected CHO cells and CHO cells stably expressing ST6GalI cDNA when transfected with a human Epo cDNA expression cassette secreted immunoreactive Epo. Human Epo from singly transfected Pro-5 CHO cells induced significant reticulocytosis (7.00+/-1.58%; mean+/-S.D. % reticulocytes; control conditioned medium 3.04+/-1.29%; P<0.0024), whereas Epo from Pro-5 cells coexpressing ST6GalI elicited a more modest reticulocytosis (4.62+/-1.02%). Thus for recombinant human Epo, engineering CHO cells to express ST6GalI activity does not enhance Epo bioactivity in vivo in mice. The availability of CHO cells that express high levels of ST6GalI activity now enables systematic studies to determine the functional requirement for this form of sialylation in recombinant human proteins.

356 ||| 17421043 ||| (2, 0.49) ||| 2 ||| 2 ||| Effect of doxycycline-regulated protein disulfide isomerase expression on the specific productivity of recombinant CHO cells: thrombopoietin and antibody. Protein disulfide isomerase (PDI), one of the ER-resident molecular chaperones, forms and isomerizes disulfide bonds. This study attempts to investigate the effect of PDI expression level on specific productivity (q) of recombinant Chinese hamster ovary (rCHO) cells producing thrombopoietin (TPO) and antibody (Ab). To regulate the PDI expression level, the Tet-Off system was introduced in TPO and Ab producing CHO cells, and stable Tet-Off cells (TPO-Tet-Off and Ab-Tet-Off) were screened using the luciferase assay. The doxycycline-regulated PDI expression system in Tet-Off rCHO cells (Tet-TPO-PDI and Tet-Ab-PDI) was established by the cotransfection of pTRE-PDI and pTK-Hyg expression vector into TPO-Tet-Off and Ab-Tet-Off cells, respectively. Subsequent screening was done by Western blot analysis of PDI and an enzyme-linked immunosorbent assay of the secreted TPO and antibody. We cultured two Tet-TPO-PDI and two Tet-Ab-PDI clones, and all these clones showed an average of 2.5-fold increase in PDI expression when compared to the basal level. In both these cell lines the PDI expression was tightly controlled by various concentrations of doxycycline. The q of TPO (q(TPO)) was unaffected but that of antibody producing cells was increased by 15-27% due to the PDI expression level.

357 ||| 11716690 ||| (2, 0.49) ||| 2 ||| 2 ||| Efficient gene transfer using reversibly cross-linked low molecular weight polyethylenimine. Polyethylenimine (PEI) is a polycation with potential application as a nonviral vector for gene delivery. Here we show that after conjugation with homobifunctional amine reactive reducible cross-linking reagents, low molecular weight polyethylenimine efficiently mediates in vitro gene delivery to Chinese hamster ovary (CHO) cells. Two cross-linking reagents, dithiobis(succinimidylpropionate) (DSP) and dimethyl.3,3'-dithiobispropionimidate\*2HCl (DTBP), were utilized based on their reactivity and chemical properties. Both reagents react with primary amines to form reducible cross-links; however, unlike DSP, the DTBP cross-linker maintains net polymer charge through amidine bond formation. PEI with a reported weight-average molecular weight (M(w)) of 800 Da was reacted with either DSP or DTBP at PEI primary amine:cross-link reactive group ratios of 1:1 and 2:1. The transfection efficiencies of the resulting cross-linked products were evaluated in CHO cells using a luciferase reporter gene under a cytomegalovirus (CMV) promoter. Our results show that cross-linked polymers mediate variable levels of transfection depending on the cross-linking reagent, the extent of conjugation, and the N/P ratio. In general, we found conjugate size to be proportional to gene transfer efficiency. Using gel retardation analysis, we also evaluate the capacity of the cross-linked polymers to condense plasmid DNA before and after reduction with 45 mM dithiothreitol (DTT). DTT mediated reduction of intra-cross-link disulfide bonds and inhibited condensation of DNA by conjugates cross-linked with DSP at a ratio of 1:1, but had little effect on the remaining polymers. Analogous intracellular reduction of transfection complexes by reduced glutathione could facilitate uncoupling of PEI from DNA to enhance gene expression.

358 ||| 10779678 ||| (2, 0.49) ||| 2 ||| 2 ||| A new Chinese hamster ovary cell line expressing alpha2,6-sialyltransferase used as universal host for the production of human-like sialylated recombinant glycoproteins. Chinese hamster ovary (CHO) cells are widely employed to produce glycosylated recombinant proteins. Our group as well as others have demonstrated that the sialylation defect of CHO cells can be corrected by transfecting the alpha2,6-sialyltransferase (alpha2,6-ST) cDNA. Glycoproteins produced by such CHO cells display both alpha2,6- and alpha2,3-linked terminal sialic acid residues, similar to human glycoproteins. Here, we have established a CHO cell line stably expressing alpha2,6-ST, providing a universal host for further transfections of human genes. Several relevant parameters of the universal host cell line were studied, demonstrating that the alpha2,6-ST transgene was stably integrated into the CHO cell genome, that transgene expression was stable in the absence of selective pressure, that the recombinant sialyltransferase was correctly localized in the Golgi and, finally, that the bioreactor growth parameters of the universal host were comparable to those of the parental cell line. A second step consisted in the stable transfection into the universal host of cDNAs for human glycoproteins of therapeutic interest, i.e. interferon-gamma and the tissue inhibitor of metalloproteinases-1. Interferon-gamma purified from the universal host carried 40.4% alpha2,6- and 59.6% alpha2,3-sialic acid residues and showed improved pharmacokinetics in clearance studies when compared to interferon-gamma produced by normal CHO cells.

359 ||| 10649229 ||| (2, 0.49) ||| 2 ||| 2 ||| Part I. Bcl-2 and Bcl-x(L) limit apoptosis upon infection with alphavirus vectors. Viral expression systems offer the ability to generate high levels of a particular protein within a relatively short period of time. In particular, alphavirus constructs based on Sindbis virus (SV) and Semliki Forest virus (SFV) are promising vehicles as they are cytoplasmic vectors with the potential for high expression levels. Two such alphavirus vectors were utilized during the current study to infect two commercially relevant cell lines, baby hamster kidney (BHK) and Chinese hamster ovary (CHO); the first was a fully competent SV derivative carrying the gene for chloramphenicol acetyltransferase (dsSV-CAT), while the second was a replication deficient SFV construct containing the human interleukin-12 (IL-12) p35 and p40 genes (SFV-IL-12). Since infection with these vectors induced apoptosis in both cell lines, the present effort was dedicated to determining the ability of anti-apoptosis genes to limit the cell death associated with these virus constructs. Infection with the dsSV-CAT vector resulted in the rapid death of BHK and CHO cells within 4 days, a phenomenon which was considerably delayed by stably overexpressing bcl-2 or bcl-x(L). In fact, cellular lifespans were doubled in both BHK-bcl2 and CHO-bclx(L) cells relative to the parental cell lines. Furthermore, the presence of these gene products provided increases of up to 2-fold in recombinant CAT production. Overexpression of bcl-2 and bcl-x(L) also altered the response of these cells upon infection with SFV-IL-12. While the parental cell lines were completely nonviable within 1 week, the BHK-bcl2, BHK-bclx(L), and CHO-bclx(L) cells each recovered from the infection, resuming exponential growth and regaining viabilities of over 90% by 9 days post-infection. Total IL-12 productivities were nearly doubled by Bcl-2 and Bcl-x(L) in the CHO cells, although this effect was apparently cell-line specific, as the native BHK cells were able to secrete more IL-12 than either of its transfected derivatives. Regardless, the presence of the anti-apoptosis genes allowed the production of IL-12 to be maintained, albeit at low levels, from each of the cell lines for the duration of the culture process. Therefore, overexpression of bcl-2 family members can have a significant impact on culture viabilities and recombinant protein production during alphavirus infections of mammalian cells.

360 ||| 10592513 ||| (2, 0.49) ||| 2 ||| 2 ||| A new biological strategy for high productivity of recombinant proteins in animal cells by the use of hypoxia-response enhancer. Oxygen supply is one of the major problems in the production of useful proteins by cultured animal cells and therefore it is of importance to devise a system by which a high productivity of human therapeutic recombinant proteins can be maintained or enhanced under low oxygen concentrations. A number of hypoxia-inducible genes have been found in animal cells and the induction in most cases is due to hypoxic activation of the gene transcription. A consensus sequence (HRE = hypoxia-response enhancer) responsible for the hypoxic activation exists in these genes and the binding of a protein, which is widely distributed in animal cells, to this sequence responding to hypoxia activates the promoter activity. The promoter of lactate dehydrogenase A gene is active in Chinese hamster ovary (CHO) cells and the vicinal HRE stimulates the promoter activity efficiently in hypoxia. We have prepared a number of permanent CHO cell lines producing recombinant human erythropoietin (Epo) under control of this promoter/HRE. Epo production was highly hypoxia-inducible when the wild-type of HRE was used but uninducible when the mutant HRE was used. There was little difference in the in vitro and in vivo activities, and glycosylation between Epo produced by the cells cultured in 21% and 2% oxygen. Furthermore, forced expression of hypoxia-inducible factor-1alpha (HIF-1alpha) enhanced Epo production in all oxygen concentrations. These results indicate that a biological strategy based on the hypoxic induction of gene transcription provides a novel system which guarantees a high productivity even uner low oxygen concentrations.

361 ||| 10187834 ||| (2, 0.48),(4, 0.33) ||| 2 ||| 2 ||| The gain-of-function Chinese hamster ovary mutant LEC11B expresses one of two Chinese hamster FUT6 genes due to the loss of a negative regulatory factor. The LEC11 Chinese hamster ovary (CHO) gain-of-function mutant expresses an alpha(1,3)fucosyltransferase (alpha(1,3)Fuc-T) activity that generates the LeX, sialyl-LeX, and VIM-2 glycan determinants and has been extensively used for studies of E-selectin ligand specificity. In order to identify regulatory mechanisms that control alpha(1,3)Fuc-T expression in mammals, mechanisms of FUT gene expression were investigated in LEC11 cells and two new, independent mutants, LEC11A and LEC11B. Northern and ribonuclease protection analyses, using probes that span the coding region of a cloned CHO FUT gene, detected transcripts in each LEC11 mutant but not in CHO cells or other gain-of-function CHO mutants that express a different alpha(1,3)Fuc-T activity. Coding region sequence analysis and alpha(1,3)Fuc-T acceptor specificity comparisons with recombinant human Fuc-TV and Fuc-TVI showed that the cloned FUT gene is orthologous to the human FUT6 gene. Southern analyses identified two closely related FUT6 genes in the Chinese hamster, whose evolutionary relationships are discussed. The blots showed that rearrangements had occurred in LEC11A and LEC11 genomic DNA, consistent with a cis mechanism of FUT6 gene activation in these mutants. By contrast, somatic cell hybrid analyses revealed that LEC11B cells express FUT6 gene transcripts due to the loss of a trans-acting, negative regulatory factor. Sequencing of reverse transcriptase-polymerase chain reaction products identified unique 5'- and 3'-untranslated region sequences in FUT6 gene transcripts from each LEC11 mutant. Northern and Southern analyses with gene-specific probes showed that LEC11A cells express only the cgFUT6A gene (where cg is Cricetulus griseus), whereas LEC11 and LEC11B cells express only the cgFUT6B gene. In LEC11A x LEC11B hybrid cells, the cgFUT6A gene was predominantly expressed, as predicted if a trans-acting negative regulatory factor functions to suppress cgFUT6B gene expression in CHO cells. This factor is predicted to be a cell type-specific regulator of FUT6 gene expression in mammals.

362 ||| 8579595 ||| (2, 0.48) ||| 2 ||| 2 ||| High-level expression of human inducible nitric oxide synthase in Chinese hamster ovary cells and characterization of the purified enzyme. Chinese hamster ovary cells were transfected with the human inducible nitric oxide synthase (iNOS) cDNA under the control of the hCMV promoter. The NOS inhibitor, S-ethyl isothiourea, was utilized to minimize toxicity from NO production. Selected colonies were grown in the absence of S-ethyl isothiourea, after which nitrite levels in the medium were measured. NOS activities in cell lysates were determined, and 9 colonies had iNOS activities of over 1 nmol of citrulline formed/min/mg protein. iNOS expression was further increased by gene amplification and the use of sodium butyrate, resulting in two cell lines with stable activity of greater than 3 nmol/min/mg. The iNOS purified from these cells had a specific activity of over 500 nmol/min/mg, and its properties were similar to native iNOS purified from cytokine-induced DLD-1 cells. This is the most efficient expression system reported to date for iNOS.

363 ||| 10359125 ||| (2, 0.48) ||| 2 ||| 2 ||| Recombinant expression of polymeric IgA: incorporation of J chain and secretory component of human origin. Mucosal J (joining) chain-expressing IgA immunocytes produce dimeric IgA that is actively transported by the epithelial polymeric Ig receptor (pIgR) to exocrine secretions. Release of secretory IgA (SIgA) occurs by cleavage of the covalently linked pIgR ectodomain, also known as bound secretory component. We have identified the human J-chain cDNA sequence through database screening, and isolated it from B cells for recombinant expression. Co-expression of this cDNA with an alpha heavy chain and a lambda light chain in Chinese hamster ovary (CHO) cells resulted in a mixture of recombinant monomeric and dimeric IgA in culture supernatants. This dimeric IgA was transported by the pIgR-mediated mechanism in vitro. Furthermore, expression of the human pIgR ectodomain together with the dimeric IgA, resulted in production of complete SIgA by the CHO cells. These results demonstrated that co-expression of the necessary polypeptide components allows a single mammalian cell to produce SIgA. Development of production systems for human antigen-specific recombinant SIgA may be important for applications in passive mucosal vaccination.

364 ||| 10700388 ||| (2, 0.47),(4, 0.31) ||| 2 ||| 2 ||| alpha(1,3)fucosyltransferases expressed by the gain-of-function Chinese hamster ovary glycosylation mutants LEC12, LEC29, and LEC30. Gain-of-function glycosylation mutants provide access to glycosylation pathways, glycosylation genes, and mechanisms that regulate expression of a glycotype. Previous studies have shown that the gain-of-function Chinese hamster ovary (CHO) mutants LEC12, LEC29, and LEC30 express an N-ethylmaleimide-resistant alpha(1, 3)fucosyltransferase (alpha(1,3)Fuc-T) activity that is not detected in CHO cells and that generates the Lewis(X) but not the sialyl-Lewis(X) determinant. The three mutants differ, however, in lectin resistance properties, expression of fucosylated antigens, and in vitro alpha(1,3)Fuc-T activities. In this paper we show that each mutant expresses Fuc-TIX, but only LEC30 cells express Fuc-TIV. Using genomic PCR and reverse-transcriptase (RT)-PCR strategies, we isolated coding portions of the CHO Fut4 and Fut9 genes. Each gene is present in a single copy in the CHO and mutant genomes. The Fut4 gene is expressed only in LEC30 cells, while all three mutants express the Fut9 gene. Interestingly, the fucosylation phenotypes of LEC12 and LEC29 cells do not correlate with the relative abundance of their Fut9 gene transcripts (LEC29 >> LEC12). Compared to LEC29 cells, LEC12 cells have an approximately 40-fold higher in vitro alpha(1,3)Fuc-T activity and bind the VIM-2 monoclonal antibody, whereas LEC29 cells do not bind VIM-2. Mixing experiments did not detect Fuc-TIX inhibitory activity in LEC29 cell extracts, and CHO cells expressing a transfected Fut9 gene behaved like LEC12 cells. Therefore, it seems that LEC29 cells may not translate their more abundant Fut9 gene transcripts efficiently or may not synthesize appropriate acceptors for internal alpha(1,3)fucosylation. Alternatively, LEC12 cells may possess, in addition to Fuc-TIX, a novel alpha(1,3)Fuc-T activity.

365 ||| 22835852 ||| (2, 0.47) ||| 2 ||| 2 ||| Increased disulphide dimer formation of latent associated peptide fusions of TGF-beta by addition of L-cystine. The development of novel protein therapeutics relies on the ability to express appreciable amounts of correctly folded recombinant proteins. Latent IFN-beta is engineered using the latency-associated peptide (LAP) of transforming growth factor beta1 (TGF-beta1) to maintain IFN-beta in a biologically inactive form until such time as it is released at sites of inflammation by matrix metalloproteinase activity (see Adams et al., 2003). CHO cells cultured in suspension were used for expression of latent IFN-beta to allow medium scale transient transfection. However, the recombinant protein expressed in this system consisted of a mixture of properly linked disulphide dimers and monomers. The ratio of dimer:monomer produced could be significantly altered towards increased dimer production by the addition of L-cystine to the CHO culture medium. The total yield of latent IFN-beta was increased by co-transfection of plasmid coding for the simian virus (SV) 40 large T antigen to the plasmid with the SV40 origin of replication expressing latent IFN-beta DNA. These results provide valuable new insights for developing protocols to produce substantial quantities of latent cytokine dimers in CHO cells in suspension.

366 ||| 19199356 ||| (2, 0.46),(1, 0.36) ||| 2 ||| 2 ||| High-level protein expression in scalable CHO transient transfection. Chinese hamster ovary cells (CHO) have been extensively utilized as the production platform for therapeutic proteins including monoclonal antibodies in pharmaceutical industry. For early development, it would be advantageous to rapidly produce large amounts of protein in the same cell line; therefore, development of a CHO transient transfection platform with high protein expression level is highly desirable. Here, we describe the development of such a platform in CHO cells. Polyethylenimine (PEI) was used as the transfection reagent. Different media were screened for the best transfection and expression performance, and UltraCHO was chosen as the best performer. DMSO and lithium acetate (LiAc) were discovered to improve CHO transient transfection expression levels significantly. A 14-day fed-batch process was successfully developed to further increase production yield. With an optimized transient transfection process, we were able to express monoclonal antibody (Mab) in CHO cells at a high level, averaging 80 mg/L. The process was successfully scaled up to 10 L working volume in a 20 L wave bioreactor. As expected, the Mabs had similar glycosylation patterns in comparison to the Mabs produced from a stably transfected CHO cell line, while in contrast Mabs expressed transiently from HEK293EBNA cells differed.

367 ||| 18448183 ||| (2, 0.45),(1, 0.45) ||| 2 ||| 2 ||| An XBP-1 dependent bottle-neck in production of IgG subtype antibodies in chemically defined serum-free Chinese hamster ovary (CHO) fed-batch processes. The optimization of production processes for therapeutic antibodies is a continuing challenge in pharmaceutical biotechnology. Although it could be demonstrated that vector design and host cell engineering can improve transcriptional and translational efficiency and thereby result in generation of high producer cell lines, it is not clear whether introduction of transgenes that regulate protein transport or affect post-translational modifications could further improve such industrial processes. Here, we show that heterologous expression of the transcription factor X-box binding protein-1 (XBP-1) can lead to an increase in endoplasmic reticulum (ER) content and specific therapeutic antibody productivity of Chinese hamster ovary (CHO)-DG44 cells in inoculum suspension cultures. This effect translates into 40% increased overall antibody titers in a fed-batch format where cells are grown in chemically defined serum-free media. Protein-A purified antibody products from mock-transfected cells and XBP-1 transfected cells were found to be of comparable quality with regard to glycosylation pattern and physicochemical characteristics. The data demonstrate the potential of XBP-1 engineering to improve mammalian cell culture production processes to yield high amounts of a therapeutic protein product of desired quality.

368 ||| 24166598 ||| (2, 0.45) ||| 2 ||| 2 ||| Salt ions and related parameters affect PEI-DNA particle size and transfection efficiency in Chinese hamster ovary cells. Transfection efficiency is directly associated with the expression level and quantity of recombinant protein after the transient transfection of animal cells. The transfection process can be influenced by many still-unknown factors, so it is valuable to study the precise mechanism and explore these factors in gene delivery. Polyethylenimine (PEI) is considered to have high transfection efficiency and endosome-disrupting capacity. Here we aimed to investigate optimal conditions for transfection efficiency by setting different parameters, including salt ion concentration, DNA/PEI ratio, and incubation time. We examined the PEI-DNA particle size using a Malvern particle size analyzer and assessed the transfection efficiency using flow cytometry in Chinese hamster ovary-S cells. Salt ions, higher amounts of PEI tended to improve the aggregation of PEI-DNA particles and the particle size of PEI-DNA complexes and the transfection efficiency were increased. Besides, the particle size was also found to benefit from longer incubation time. However, the transfection efficiency increased to maximum of 68.92 % at an incubation time of 10 min, but decreased significantly thereafter to 23.71 %, when incubating for 120 min (P < 0.05). Besides, PEI-DNA complexes formed in salt-free condition were unstable. Our results suggest DNA and PEI incubated in 300 mM NaCl at a ratio of 1:4 for 10 min could achieve the optimal transfection efficiency. Our results might provide guidance for the optimization of transfection efficiency and the industrial production of recombinant proteins.

369 ||| 9202714 ||| (2, 0.44),(6, 0.37) ||| 2 ||| 2 ||| CHO transfectants produce large amounts of recombinant protein in suspension culture. Chinese hamster ovary (CHO) cells transfected with various genes are widely used as adherent cell monolayers to produce recombinant proteins. In this report we present a new culture technique for CHO cells transfected with the vector pPOL-DHFR-CD14 using a minifermenter (miniPERM, Heraeus) for the production of recombinant human endotoxin receptor CD14 (rCD14). The transfectants were cultured for 12-17 days under serum-free conditions and formed spheroids. From this system we harvested supernatants containing up to 3.1 mg/ml recombinant CD14 (rCD14). This represents a 200-fold increase of rCD14 yield compared to conventional adherent CHO cell culture.

370 ||| 22465547 ||| (2, 0.44),(1, 0.34) ||| 2 ||| 2 ||| Evaluation of the OSCAR system for the production of monoclonal antibodies by CHO-K1 cells. Biopharmaceutical production of complex recombinant protein therapeutics currently relies on mammalian cells. The development of high-yielding stable cell lines requires processes of transfection, selection and adaptation. With several technologies available, selection has been most frequently based on dihydrofolate reductase or glutamine synthetase systems, which can be very time-consuming. Due to the pressure to reduce development costs and speed up time to market, new technologies are emerging, as the promising OSCAR expression system that could provide more rapid development of high-yielding stable cell lines than the traditional systems. However, further evaluation of its application in a wider range of cell types and media is still necessary. In this study, application of OSCAR for the transfection of a CHO-K1 cell line with a monoclonal antibody was evaluated. OSCAR was reasonably fast and simple, without negative impact on cell growth characteristics. However, minigene selection was critical, with only pDWM128 working for the cell line assessed. Initial relatively high levels of production decreased significantly in the first few weeks of passing, remaining relatively stable although with low yield thereafter. The results suggest that more work is required to develop methodologies and prove that OSCAR has significant value to the bioproduction industry.

371 ||| 8552676 ||| (2, 0.44) ||| 2 ||| 2 ||| High-level production of recombinant human lysosomal acid alpha-glucosidase in Chinese hamster ovary cells which targets to heart muscle and corrects glycogen accumulation in fibroblasts from patients with Pompe disease. Infantile Pompe disease is a fatal genetic muscle disorder caused by a deficiency of acid alpha-glucosidase, a glycogen-degrading lysosomal enzyme. We constructed a plasmid containing a 5'-shortened human acid alpha-glucosidase cDNA driven by the cytomegalovirus promoter, as well as the aminoglycoside phosphotransferase and dihydrofolate reductase genes. Following transfection in dihydrofolate reductase-deficient Chinese hamster ovary cells, selection with Geneticin, and amplification with methotrexate, a cell line producing high levels of the alpha-glucosidase was established. In 48 hr, the cells cultured in Iscove's medium with 5 mM butyrate secreted 110-kDa precursor enzyme that accumulated to 91 micrograms.ml-1 in the medium (activity, > 22.6 mumol.hr-1.ml-1). This enzyme has a pH optimum similar to that of the mature form, but a lower Vmax and Km for 4-methylumbelliferyl-alpha-D-glucoside. It is efficiently taken up by fibroblasts from Pompe patients, restoring normal levels of acid alpha-glucosidase and glycogen. The uptake is blocked by mannose 6-phosphate. Following intravenous injection, high enzyme levels are seen in heart and liver. An efficient production system now exists for recombinant human acid alpha-glucosidase targeted to heart and capable of correcting fibroblasts from patients with Pompe disease.

372 ||| 22833427 ||| (2, 0.44) ||| 2 ||| 2 ||| Functional heterogeneity and heritability in CHO cell populations. In this study, we address the hypothesis that it is possible to exploit genetic/functional variation in parental Chinese hamster ovary (CHO) cell populations to isolate clonal derivatives that exhibit superior, heritable attributes for biomanufacturing--new parental cell lines which are inherently more "fit for purpose." One-hundred and ninety-nine CHOK1SV clones were isolated from a donor CHOK1SV parental population by limiting dilution cloning and microplate image analysis, followed by primary analysis of variation in cell-specific proliferation rate during extended deep-well microplate suspension culture of individual clones to accelerate genetic drift in isolated cultures. A subset of 100 clones were comparatively evaluated for transient production of a recombinant monoclonal antibody (Mab) and green fluorescent protein following transfection of a plasmid vector encoding both genes. The heritability of both cell-specific proliferation rate and Mab production was further assessed using a subset of 23 clones varying in functional capability that were subjected to cell culture regimes involving both cryopreservation and extended sub-culture. These data showed that whilst differences in transient Mab production capability were not heritable per se, clones exhibiting heritable variation in specific proliferation rate, endocytotic transfectability and N-glycan processing were identified. Finally, for clonal populations most "evolved" by extended sub-culture in vitro we investigated the relationship between cellular protein biomass content, specific proliferation rate and cell surface N-glycosylation. Rapid-specific proliferation rate was inversely correlated to CHO cell size and protein content, and positively correlated to cell surface glycan content, although substantial clone-specific variation in ability to accumulate cell biomass was evident. Taken together, our data reveal the dynamic nature of the CHO cell functional genome and the potential to evolve and isolate CHO cell variants with improved functional properties in vitro.

373 ||| 21540058 ||| (2, 0.44) ||| 2 ||| 2 ||| Recombinant glycoprotein E produced in mammalian cells in large-scale as an antigen for varicella-zoster-virus serology. A recombinant glycoprotein E (gE) from varicella-zoster virus (VZV) was generated and produced in Chinese Hamster Ovary (CHO) cells, in the development of a specific antigen for analysis of IgG antibodies to VZV. Several stable gE-secreting clones were established and one clone was adapted to growth in serum-free suspension culture. When the cells were cultured in a perfusion bioreactor, gE was secreted into the medium, from where it could be easily purified. The recombinant gE was then evaluated as a serological antigen in ELISA. When compared to a conventional whole virus antigen, the VZV gE showed similar results in ELISA-based seroprevalence studies of 854 samples derived from blood donors, students, ischemic stroke patients and their controls, including samples with border-line results in previous analyses. Eight samples (0.9%) were discordant, all being IgG-negative by the VZV gE ELISA and positive by the whole virus ELISA. The sensitivity and specificity of the VZV gE ELISA were 99.9% and 100%, respectively, compared to 100% and 88.9% for the VZV whole virus ELISA. The elderly subjects showed similar reactivities to both antigens, while VZV gE gave lower signals in the younger cohorts, suggesting that antibodies to gE may increase with age. It was concluded that the recombinant VZV gE from CHO cells was suitable as a serological antigen for the detection of IgG antibodies specific for VZV.

374 ||| 20564614 ||| (2, 0.44) ||| 2 ||| 2 ||| Highly efficient deletion of FUT8 in CHO cell lines using zinc-finger nucleases yields cells that produce completely nonfucosylated antibodies. IgG1 antibodies produced in Chinese hamster ovary (CHO) cells are heavily alpha1,6-fucosylated, a modification that reduces antibody-dependent cellular cytotoxicity (ADCC) and can inhibit therapeutic antibody function in vivo. Addition of fucose is catalyzed by Fut8, a alpha1,6-fucosyltransferase. FUT8(-/-) CHO cell lines produce completely nonfucosylated antibodies, but the difficulty of recapitulating the knockout in protein-production cell lines has prevented the widespread adoption of FUT8(-/-) cells as hosts for antibody production. We have created zinc-finger nucleases (ZFNs) that cleave the FUT8 gene in a region encoding the catalytic core of the enzyme, allowing the functional disruption of FUT8 in any CHO cell line. These reagents produce FUT8(-/-) CHO cells in 3 weeks at a frequency of 5% in the absence of any selection. Alternately, populations of ZFN-treated cells can be directly selected to give FUT8(-/-) cell pools in as few as 3 days. To demonstrate the utility of this method in bioprocess, FUT8 was disrupted in a CHO cell line used for stable protein production. ZFN-derived FUT8(-/-) cell lines were as transfectable as wild-type, had similar or better growth profiles, and produced equivalent amounts of antibody during transient transfection. Antibodies made in these lines completely lacked core fucosylation but had an otherwise normal glycosylation pattern. Cell lines stably expressing a model antibody were made from wild-type and ZFN-generated FUT8(-/-) cells. Clones from both lines had equivalent titer, specific productivity distributions, and integrated viable cell counts. Antibody titer in the best ZFN-generated FUT8(-/-) cell lines was fourfold higher than in the best-producing clones of FUT8(-/-) cells made by standard homologous recombination in a different CHO subtype. These data demonstrate the straightforward, ZFN-mediated transfer of the Fut8- phenotype to a production CHO cell line without adverse phenotypic effects. This process will speed the production of highly active, completely nonfucosylated therapeutic antibodies.

375 ||| 10099467 ||| (2, 0.43),(6, 0.34) ||| 2 ||| 2 ||| Chinese hamster ovary cells with constitutively expressed sialidase antisense RNA produce recombinant DNase in batch culture with increased sialic acid. Under some cell culture conditions, recombinant glycoprotein therapeutics expressed in Chinese hamster ovary (CHO) cells lose sialic acid during the course of the culture (Sliwkowski et al., 1992; Munzert et al., 1996). A soluble sialidase of CHO cell origin degrades the expressed recombinant protein and has been shown to be released into the culture fluid as the viability of the cells decreases. To reduce the levels of the sialidase and to prevent desialylation of recombinant protein, a CHO cell line has been developed that constitutively expresses sialidase antisense RNA. Several antisense expression vectors were prepared using different regions of the sialidase gene. Co-transfection of the antisense constructs with a vector conferring puromycin resistance gave rise to over 40 puromycin resistant clones that were screened for sialidase activity. A 5' 474 bp coding segment of the sialidase cDNA, in the inverted orientation in an SV 40-based expression vector, gave maximal reduction of the sialidase activity to about 40% wild-type values. To test if this level of sialidase would lead to increased sialic acid content of an expressed recombinant protein, the 474 antisense clone was employed as a host for expression of human DNase as a model glycoprotein. The sialic acid content of the DNase produced in the antisense cultures was compared with material made in the wild-type parental cell line. About 20-37% increase in sialic acid content, or 0.6-1.1 mole of additional sialic acid out of a total of 3.0 mole on the product, was found on the DNase made in the antisense cell lines.

376 ||| 17172664 ||| (2, 0.43) ||| 2 ||| 2 ||| Serum-free suspension large-scale transient transfection of CHO cells in WAVE bioreactors. Here, we report the development of a large-scale transient expression platform utilizing Chinese hamster ovary (CHO) cells. The majority of recombinant proteins and antibodies that are produced for preclinical models and clinical trials are expressed in stably transfected CHO cells. A protocol for transient transfection of CHO cells that is rapid, reproducible, and cost-effective would therefore streamline the process from research to development and help avoid any potential host species induced variation in the molecule of interest. CHO cells were adapted to grow in serum-free suspension conditions in spinner flask cultures in a proprietary in-house developed growth medium. In developing this transient transfection protocol, the parameters optimized included the transfection reagent of choice, the cell density at the time of transfection, the plasmid DNA concentration, and the transfection reagent concentration. Using this optimized protocol, we have expressed recombinant proteins, including antibodies, at an expression level of up to 9.4 mg/L. We also report transient transfections from 500 mL working volume (w.v.) up to 20 L w.v. in a WAVE bioreactor. Using this optimized protocol, it is possible to rapidly (within 10 d) produce up to 100 mg of recombinant protein for further study.

377 ||| 16752368 ||| (2, 0.43) ||| 2 ||| 2 ||| Enhancement of recombinant protein production in Chinese hamster ovary cells through anti-apoptosis engineering using 30Kc6 gene. It was previously reported that silkworm hemolymph (SH) inhibits apoptosis and increases the production of recombinant human erythropoietin (EPO) in Chinese hamster ovary (CHO) cells. The apoptosis-inhibiting component in SH is a member of 30K protein family. In this study, the CHO cell line producing EPO was manipulated genetically to express the 30Kc6 gene encoding a 30K protein in the hemolymph of the silkworm, Bombyx mori. The transient expression of 30Kc6 significantly suppressed the cell death induced by serum deprivation. A stable cell line expressing 30Kc6 with an anti-apoptotic property was established. The stable expression of 30Kc6 inhibited serum-deprivation-induced apoptosis and increased the cell density and EPO titer by 5- and 10-fold, respectively. The positive effects of the 30Kc6 expression on cell viability and productivity were due to the stable maintenance of the mitochondrial activity. The 30Kc6 expression efficiently suppressed the depolarization of the mitochondrial membrane and subsequently balanced the generation/consumption of ATP. The use of the 30Kc6 gene is expected to provide a new method of host cell engineering for improving the productivity of the recombinant protein.

378 ||| 19428735 ||| (2, 0.42),(1, 0.38) ||| 2 ||| 2 ||| Heterologous expression of the lipid transfer protein CERT increases therapeutic protein productivity of mammalian cells. Recent studies have demonstrated that the introduction of transgenes regulating protein transport or affecting post-translational modifications can further improve industrial processes for the production of therapeutic proteins in mammalian cells. Our study on improving therapeutic protein production in CHO cells by heterologous expression of the ceramide transfer protein (CERT) was initiated by the recent discovery that CERT is involved in protein kinase D (PKD)-dependent protein transport from the Golgi to the plasma membrane. We generated a set of CHO DG44 cell lines by stable integration of constructs expressing either CERT wild-type or CERT S132A, a mutant conferring increased lipid transfer activity, or a mock plasmid. CHO cells expressing heterologous CERT demonstrated significantly higher specific productivities of the therapeutic protein HSA when grown in inoculum suspension cultures. This effect translated into significantly increased overall HSA titers in a fed-batch format where cells are grown in chemically defined serum-free media. Furthermore, we could show that CERT also enhanced monoclonal antibody secretion in two IgG production cell lines with different basal productivities. The data demonstrate the potential of CERT engineering to improve mammalian cell culture production processes to yield high amounts of a therapeutic protein product of desired quality. To our knowledge, this is the first study showing a bottle neck in recombinant protein secretion at the Golgi complex in mammalian cells.

379 ||| 21312367 ||| (2, 0.41),(1, 0.38) ||| 2 ||| 2 ||| Fed-batch bioreactor performance and cell line stability evaluation of the artificial chromosome expression technology expressing an IgG1 in Chinese hamster ovary cells. The artificial chromosome expression (ACE) technology system uses an engineered artificial chromosome containing multiple site-specific recombination acceptor sites for the rapid and efficient construction of stable cell lines. The construction of Chinese hamster ovary(CHO) cell lines expressing an IgG1 monoclonal antibody (MAb) using the ACE system has been previously described (Kennard et al., Biotechnol Bioeng. 2009;104:540-553). To further demonstrate the manufacturing feasibility of the ACE system, four CHO cell lines expressing the human IgG1 MAb 4A1 were evaluated in batch and fed-batch shake flasks and in a 2-L fed-batch bioreactor. The batch shake flasks achieved titers between 0.7 and 1.1 g/L, whereas the fed-batch shake flask process improved titers to 2.5-3.0 g/L. The lead 4A1 ACE cell line achieved titers of 4.0 g/L with an average specific productivity of 40 pg/(cell day) when cultured in a non optimized 2-L fed-batch bioreactor using a completely chemically defined process. Generational stability characterization of the lead 4A1-expressing cell line demonstrated that the cell line was stable for up to 75 days in culture. Product quality attributes of the 4A1 MAb produced by the ACE system during the stability evaluation period were unchanged and also comparable to existing expression technologies such as the CHO-dhfr system. The results of this evaluation demonstrate that a clonal, stable MAb-expressing CHO cell line can be produced using ACE technology that performs competitively using a chemically defined fed-batch bioreactor process with comparable product quality attributes to cell lines generated by existing technologies.

380 ||| 25340707 ||| (2, 0.41),(1, 0.31) ||| 2 ||| 2 ||| Efficient production of sTNFRII-gAD fusion protein in large quantity by use of the modified CHO-S cell expression system. TNFalpha is one of the initial and important mediators to activate downstream signaling pathways by binding to trimerized TNFalpha receptors (TNFR), and thus is an ideal drug target for cancer therapy. Taking advantage of intrinsic homotimerization of the globular domain of adiponectin (gAD), we have developed a novel TNFalpha antagonist, the trimerized fusion protein named sTNFRII-gAD. However, our previously-used CHO expression system yielded less than 10 mg/L of sTNFRII-gAD. To produce large quantities of sTNFRII-gAD efficiently, we used a modified CHO-S cell expression system, which is based on a pMH3 vector with non-coding GC-rich DNA fragments for high-level gene expression. We obtained stable clones that produced 75 mg/L of sTNFRII-gAD in the 96-well plate, adapted the clones to 40 ml suspension serum-free batch culture, then optimized the culturing conditions to scale up the fed-batch culture in a 3 L shake-flask and finally in a 5 L AP30 bioreactor. We achieved a final yield of 52 mg/L of sTNFRII-gAD. The trimerized sTNFRII-gAD exhibited the higher affinity to TNFalpha with a dissociation constant (Kd) of 5.63 nM than the dimerized sTNFRII-Fc with a Kd of 13.4 nM, and further displayed the higher TNFalpha-neutralizing activity than sTNFRII-Fc (p<0.05) in a L929 cytotoxicity assay. Therefore, the strategy employed in this study may provide an efficient avenue for large-scale production of other recombinant proteins by use of the modified CHO-S cell expression system.

381 ||| 23794499 ||| (2, 0.41) ||| 2 ||| 2 ||| Use of an anti-apoptotic CHO cell line for transient gene expression. Transient gene expression in mammalian cells allows for rapid production of recombinant proteins for research and preclinical studies. Here, we describe the development of a polyethylenimine (PEI) transient transfection system using an anti-apoptotic host cell line. The host cell line, referred to as the Double Knockout (DKO), was generated by deleting two pro-apoptotic factors, Bax and Bak, in a CHO-K1 cell line using zinc finger nuclease mediated gene disruption. Optimized DNA and PEI volumes for DKO transfections were 50% and 30% lower than CHO-K1, respectively. During transfection DKO cells produced relatively high levels of lactate, but this was mitigated by a temperature shift to 31 C which further enhanced productivity. DKO cells expressed ~3- to 4-fold higher antibody titers than CHO-K1 cells. As evidence of their anti-apoptotic properties post-transfection, DKO cells maintained higher viability and had reduced levels of active caspase-3 compared to CHO-K1 cells. Nuclear plasmid DNA copy numbers and message levels were significantly elevated in DKO cells. Although DNA uptake levels, as early as 40 min post-transfection, were higher in DKO cells this was not due to differences in cell surface heparan sulfate (HS) or initial endocytosis mechanism as both cell types utilized caveolae- and clathrin-mediated endocytosis to internalize DNA:PEI complexes. These results suggest that the increased transfection efficiency and titers from DKO cells are attributed to their resistance to transfection-induced apoptosis and not differences in endocytosis mechanism.

382 ||| 10516580 ||| (2, 0.41) ||| 2 ||| 2 ||| Tetracycline-regulated overexpression of glycosyltransferases in Chinese hamster ovary cells. The glycosylation patterns of recombinant therapeutic glycoproteins can be engineered by overexpression of glycosyltransferases in the host cells used for glycoprotein production. Most prior glycosylation engineering experiments have involved constitutive expression of cloned glycosyltransferases. Here we use tetracycline-regulated expression of two glycosyltransferases, N-acetylglucosaminlytransferases III and V (GnTIII and GnTV) to manipulate glycoform biosynthesis in Chinese hamster ovary (CHO) cells and to study the effect of glycosyltransferase overexpression on this host. The amount of GnTIII and GnTV in these cells, and the glycosylation patterns of several cellular glycoproteins, could be controlled simply by manipulating the concentration of tetracycline in the culture medium. Using this system, it was found that overexpression of either GnTIII or GnTV to high levels led to growth inhibition and was toxic to the cells, indicating that this may be a general feature of glycosyltransferase overexpression. This phenomenon has not been reported previously, probably due to the widespread use of constitutive promoters, and should be taken into account when designing vectors for glycosylation engineering. The growth inhibition effect sets an upper limit to the level of glycosyltransferase overexpression, and may thereby also limit the maximum extent of in vivo modification of poorly accessible glycosylation sites. Also, such inhibition implies a bound on constitutive glycosyltransferase expression which can be cloned.

383 ||| 8521401 ||| (2, 0.40),(4, 0.36) ||| 2 ||| 2 ||| DNA topoisomerase II alpha expression is associated with alkylating agent resistance. Increased expression of DNA topoisomerase II alpha has been associated with resistance to certain DNA-damaging alkylating agents, but no causal relationship or mechanism has been established. To investigate this observation, we developed a model of topoisomerase II overexpression by transfecting a full-length Chinese hamster ovary topoisomerase II alpha into EMT6 mouse mammary carcinoma. Topoisomerase II alpha-transfected cell lines demonstrated continued topoisomerase II alpha mRNA and protein expression, which were undetectable in vector-only lines, in stationary phase (G0-G1). The topoisomerase II transfectants were approximately 5-10-fold resistant to the alkylating agents cisplatin and mechlorethamine. Upon release from G0-G1, the topoisomerase II transfectants demonstrated more rapid thymidine incorporation and shorter cell-doubling times than control cells. Purified topoisomerase II and nuclear extracts with topoisomerase II-decatenating activity bound to cisplatin-treated DNA with significantly greater affinity than to untreated DNA in a cisplatin concentration-dependent manner. These observations suggest that expression of topoisomerase II alpha may have a role in cellular resistance to antineoplastic alkylating agents. The mechanism for this may involve increased binding of topoisomerase II alpha to alkylating agent-damaged DNA.

384 ||| 19376231 ||| (2, 0.40),(1, 0.30) ||| 2 ||| 2 ||| Increased expression of the integral membrane protein ErbB2 in Chinese hamster ovary cells expressing the anti-apoptotic gene Bcl-xL. Receptor tyrosine kinases (RTKs) are the second largest family of membrane receptors and play a key role in the regulation of vital cellular processes, such as control of cell growth, differentiation, metabolism, and migration. The production of whole-length RTKs in large quantities for biophysical or structural characterization, however, is a challenge. In this study, a cell engineering strategy using the anti-apoptotic Bcl-2 family protein, Bcl-x(L), was tested as a potential method for increasing stable expression levels of a recombinant RTK membrane protein in Chinese hamster ovary (CHO) cells. Wild-type and CHO cells stably overexpressing heterologous Bcl-x(L) were transformed with the gene for a model RTK membrane protein, ErbB2, on a plasmid also containing the Zeocin resistance gene. While CHO cells exhibited a gradual decrease in expression with passaging, CHO-bcl-x(L) cells offered an increased and sustained level of ErbB2 expression following continuous passaging over more than 33 days in culture. The increased ErbB2 expression in CHO-bcl-x(L) cells was evident both in stable transfected pools and in clonal isolates, and demonstrated both in Western blot analysis and flow cytometry. Furthermore, the sustained high-level protein expression in CHO-bcl-x(L) cells does not alter the correct membrane localization of the ErbB2 protein. Our results demonstrate that cellular engineering, specifically anti-apoptosis engineering, can provide increased and stable ErbB2 membrane protein expression in mammalian cells. This approach may also be useful for other membrane proteins in which large quantities are needed for biophysical and structural studies.

385 ||| 9663332 ||| (2, 0.39),(6, 0.33) ||| 2 ||| 2 ||| Genetic engineering of glucose-stimulated insulin secretion in Chinese hamster ovary cells. To engineer an a non-islet cell capable of glucose-stimulated insulin secretion, a chinese hamster ovary cell line (CHO) was transfected with a mammalian expression vector carrying the human insulin cDNA (pCB/hINS). More proinsulin than insulin was released daily by the stably transformed cell line (CHO-INS). Examination of acid-ethanol extracts confirmed that both insulin and proinsulin were stored. Immunohistochemical analysis of the cells also showed that (pro)insulin was stored. Unlike beta cells, CHO-INS cells did not secrete insulin in response to glucose. To investigate this lack of effect, we examined whether transfection of GLUT2 cDNA, which is ordinarily not expressed in CHO-INS cells, would confer glucose-stimulated insulin secretion. Consequently, we have demonstrated that glucose regulated insulin release occurs in the CHO-INS-GLUT2 cell line and that glucose potentiates the insulin secretory response to non-glucose secretagogues.

386 ||| 23516471 ||| (2, 0.39) ||| 2 ||| 2 ||| Use of a molecular genetic platform technology to produce human Wnt proteins reveals distinct local and distal signaling abilities. Functional and mechanistic studies of Wnt signaling have been severely hindered by the inaccessibility of bioactive proteins. To overcome this long-standing barrier, we engineered and characterized a panel of Chinese hamster ovary (CHO) cell lines with inducible transgenes encoding tagged and un-tagged human WNT1, WNT3A, WNT5A, WNT7A, WNT11, WNT16 or the soluble Wnt antagonist Fzd8CRD, all integrated into an identical genomic locus. Using a quantitative real-time bioluminescence assay, we show that cells expressing WNT1, 3A and 7A stimulate Wnt/beta-catenin reporter activity, while the other WNT expressing cell lines interfere with this activation. Additionally, in contrast to WNT3A, WNT1 only exhibits activity when cell-associated, and thus only signals to neighboring cells. The reporter assay also revealed a rapid decline of Wnt activity at 37 C, indicating that Wnt activity is highly labile. These engineered cell lines will reduce the cost of making and purifying Wnt proteins and serve as a continuous, reliable and regulatable source of Wnts to research laboratories around the world.

387 ||| 23475711 ||| (2, 0.39) ||| 2 ||| 2 ||| Engineering a human-like glycosylation to produce therapeutic glycoproteins based on 6-linked sialylation in CHO cells. When recombinant glycoproteins for therapeutic use are to be produced on an industrial scale, there is a crucial need for technologies that can engineer fast-growing stable cells secreting the protein drug at a high rate and with a defined and safe glycosylation profile. Current cell lines approved for drug production are essentially from rodent origin. Their glycosylation machinery often adds undesired carbohydrate determinants which may alter protein folding, induce immunogenicity, and reduce circulatory life span of the drug. Notably, sialic acid as N-acetylneuraminic acid is not efficiently added in most mammalian cells and the 6-linkage is missing in rodent cells. Engineering cells with the various enzymatic activities required for sialic acid transfer has not yet succeeded in providing a human-like pattern of glycoforms to protein drugs. To date, there is a need for engineering animal cells and get highly sialylated products that resemble as closely as possible to human proteins. We have designed ST6Gal minigenes to optimize the ST6GalI sialyltransferase activity and used them to engineer ST6(+)CHO cells. When stably transfected in cells expressing a protein of interest or not, these constructs have proven to equip cell clones with efficient transfer activity of 6-linked sialic acid. In this chapter, we describe a methodology for generating healthy stable adherent clones with hypersialylation activity and high secretion rate.

388 ||| 22367610 ||| (2, 0.39) ||| 2 ||| 2 ||| Understanding the mechanistic roles of 30Kc6 gene in apoptosis and specific productivity in antibody-producing Chinese hamster ovary cells. Previously, we reported that the expression of Bombyx mori 30Kc6 gene in Chinese hamster ovary (CHO) cells increases recombinant protein production by both inhibiting apoptosis and enhancing specific productivity. In this study, in order to gain a thorough understanding of the roles of 30Kc6 gene in antibody production, the mechanisms modulating cell apoptosis and specific productivity were investigated. 30Kc6 gene was introduced into a CHO cell line producing a chimeric anti-human CD20 monoclonal antibody. The stable expression of 30Kc6 increased cell viability and productivity by 46.7% and 3.4-folds, respectively. It was observed that the Bax translocation from cytosol to mitochondria and the cytochrome c (cyt c) release from mitochondrial intermembrane space to cytosol were repressed, which resulted in a decrease in the activation of apoptosis executioner, caspase-3. On the other hand, 30Kc6 expression increased the specific productivity by 2.3-folds. However, at the transcription level, the relative levels of heavy and light chain mRNAs increased only by 8.3% and 8.7%, respectively, which was not accountable for the observed increment in the specific productivity. Instead, the mitochondrial membrane potential was maintained and the ATP generation was stimulated. A higher ATP level could activate the mammalian target of rapamycin (mTOR), which drives the translation initiation and elongation by phosphorylating eukaryotic initiation factor 4E binding protein 1 (4EBP1) and S6 kinase 1 (S6K1). In the 30Kc6-expressing cells, both the 4EBP1 and S6K1 were phosphorylated at higher levels, which indicated that the increased specific productivity primarily resulted from the boost of translation process. Furthermore, it was also found that the specific uptake rates of glucose and glutamine were not affected by 30Kc6 expression, demonstrating that the enhanced ATP generation and consequently maintained mTOR activity were due to 30Kc6 expression but not the different metabolic uptake rates. In conclusion, 30Kc6 expression inhibited apoptosis by repressing the Bax translocation, which down-regulated the downstream cascade responses including cyt c release and caspase-3 activation. Also, 30Kc6 expression increased the specific productivity by enhancing the translation process.

389 ||| 15005154 ||| (2, 0.39) ||| 2 ||| 2 ||| Enhanced productivity of G1 phase Chinese hamster ovary cells using the GADD153 promoter. Productivity of three different promoters at various cell cycle stages and under two distinct growth conditions was examined in Chinese hamster ovary cells. Under the Growth Arrest and DNA Damage inducible GADD153 promoter, productivity of the short half-live variant of the enhanced green fluorescent protein (d2EGFP) and the secreted alkaline phosphatase (SEAP) was highest at the G1 phase of the cell cycle and at serum starvation, while under the cytomegalovirus (CMV) or the simian virus SV40 promoter, productivity was highest at S-phase and in complete medium. These results indicate the utility of the GADD153 promoter for production purposes under protein-free conditions.

390 ||| 24504562 ||| (2, 0.38),(1, 0.38) ||| 2 ||| 2 ||| Dynamics of unfolded protein response in recombinant CHO cells. Genes in the protein secretion pathway have been targeted to increase productivity of monoclonal antibodies in Chinese hamster ovary cells. The results have been highly variable depending on the cell type and the relative amount of recombinant and target proteins. This paper presents a comprehensive study encompassing major components of the protein processing pathway in the endoplasmic reticulum (ER) to elucidate its role in recombinant cells. mRNA profiles of all major ER chaperones and unfolded protein response (UPR) pathway genes are measured at a series of time points in a high-producing cell line under the dynamic environment of a batch culture. An initial increase in IgG heavy chain mRNA levels correlates with an increase in productivity. We observe a parallel increase in the expression levels of majority of chaperones. The chaperone levels continue to increase until the end of the batch culture. In contrast, calreticulin and ERO1-L alpha, two of the lowest expressed genes exhibit transient time profiles, with peak induction on day 3. In response to increased ER stress, both the GCN2/PKR-like ER kinase and inositol-requiring enzyme-1alpha (Ire1alpha) signalling branch of the UPR are upregulated. Interestingly, spliced X-Box binding protein 1 (XBP1s) transcription factor from Ire1alpha pathway is detected from the beginning of the batch culture. Comparison with the expression levels in a low producer, show much lower induction at the end of the exponential growth phase. Thus, the unfolded protein response strongly correlates with the magnitude and timing of stress in the course of the batch culture.

391 ||| 21380526 ||| (2, 0.36) ||| 2 ||| 2 ||| Overexpression of survivin and cyclin D1 in CHO cells confers apoptosis resistance and enhances growth in serum-free suspension culture. To optimize Chinese hamster ovary (CHO) cell culture to recombinant protein therapeutic production, we stably overexpressed survivin and cyclin D1 in three CHO DG44-derived cell lines. The modifications conferred increases of 56-94% in S-phase fractions and decreases of 33-43% in early-stage apoptosis fractions. Clone 6.3, which expressed the highest levels of survivin and cyclin D1, reached significantly greater cell densities in suspension (2.7 x 10(6) cells/ml) following serum deprivation. Nude mice inoculated with the modified cells showed no tumorigenesis suggesting that the CHO DG44-derived cell lines are viable candidates for biopharmaceutical production.

392 ||| 9226719 ||| (2, 0.35),(3, 0.32) ||| 2 ||| 2 ||| Expression and purification of recombinant human apolipoprotein A-I in Chinese hamster ovary cells. The expression of apolipoprotein A-I (apoA-I) has been shown to be very difficult due to its amphiphilic character, autoaggregation, and degradation. We have expressed apoA-I using CHO cells, Baculovirus, and Escherichia coli [Schmidt et al., J. Biol. Chem. (1995) 270, 469-475]. Here we report about optimized conditions for the expression of proapoA-I in CHO cells, testing various serum-free media. We were able to yield apoA-I expression up to 80 micrograms/ml, by far the highest ever reported. However, immunoblot analysis revealed degraded apoA-I. The best apoA-I expression testing various conditions was about 20-30 micrograms/ml without any evidence of degradation. Interestingly, the apoA-I expression resulted in reproducible apoA-I fragments of 26 and 14 kDa. These fragments are consistent with already reported in vivo findings, in which carboxy-terminal proteolysis was suggested. The use of the protease inhibitors pepstatin and chymostatin, both carboxy-peptidase inhibitors, did result in contrast to other studied protease inhibitors in increased apoA-I yield. Therefore, limited carboxy-terminal proteolysis contributes to the degradation of CHO cell-secreted apoA-I. In addition, we evaluated various purification methods for the preparative isolation of recombinant apoA-I. In our hands we obtained the best recovery and no degradation with reversed-phase chromatography using a FPLC system.

393 ||| 8879169 ||| (2, 0.34) ||| 2 ||| 2 ||| Quantitative analysis of transient gene expression in mammalian cells using the green fluorescent protein. The green fluorescent protein (Gfp) has been used as a reporter, along with flow cytometric analysis, to follow the dynamics of gene expression in transiently transfected mammalian cells. Gene transfer conditions for lipofection were optimized. The highest fraction of transfectants were obtained when lipid-DNA complexes were formed with 6 microliters lipid and 1 microgram DNA for chinese hamster ovary (CHO) cells and with 9 microliters lipid and 2 micrograms DNA for NIH/3T3 cells. Chinese hamster ovary cells were monitored for Gfp expression and growth for 6 days following transfection. An initial decrease in viability for 36 h was observed after which cell growth followed exponential kinetics with increasing viability. Intracellular accumulation of recombinant protein peaked at 24 h post-transfection and then decreased with first order kinetics at a rate comparable to the specific growth rate. It appears that dilution by growth accounts for the decrease of Gfp in the biomass. Immunofluorescent staining of Gfp and subsequent flow cytometric analysis of transfected cells revealed a linear correlation between the green fluorescence and immunofluorescence. This indicates that green fluorescence is a quantitative measure of intracellular Gfp in single cells in spite of the dynamics of post-translational modifications involved in the conversion of expressed protein into its fluorescent form. A structured model has been formulated to describe the observed kinetics of gene expression and fluorophore formation. The model accurately predicts experimental trends and suggests that the fraction of non-fluorescent Gfp is significant only during the initial period of gene expression.

394 ||| 8830314 ||| (2, 0.33) ||| 2 ||| 2 ||| Expression of human metallothionein-III confers protection against serum-free exposure of stably transfected Chinese hamster ovary CHO-K1 cells. The cloned human metallothionein (MT)-III coding region was permanently transfected in Chinese hamster ovary (CHO-K1) cells in order to investigate the growth regulatory effects of this brain-specific protein. Reverse transcription-polymerase chain reaction (RT-PCR) experiments demonstrated stable expression of MT-III mRNA in CHO-K1/MT-III cells. Whereas in the presence of serum no differences were observed between the cell growth of both CHO-K1/MT-III and CHO-K1/pcDNA3-plasmid transfected cells, cell survival was attenuated differently in serum-free medium. CHO-K1/MT-III cells were more resistant (40 +/- 8%) to serum deprivation than pcDNA3-plasmid transfected cells. Recovery of cell growth for both cell lines was obtained by supplementing serum (0.25%), although with a different growth rate; half-maximal 3-(4,5-dimethylthiazol-2-yl)-2,5- diphenyl tetrazolium bromide (MTT)-conversion was observed between 5.1-5.7 and 4.2-4.5 days for CHO-K1/pcDNA3 and CHO-K1/MT-III cells, respectively. These results suggest a protective role for cloned human MT-III, besides its reported inhibitory and stimulatory effects on cell survival.

395 ||| 10486133 ||| (2, 0.33) ||| 2 ||| 2 ||| Filtroporation: A simple, reliable technique for transfection and macromolecular loading of cells in suspension. Cultured Chinese hamster ovary (CHO) cells suspended in their growth medium were forced by gas pressure through the uniformly sized micropores of filter membranes. This procedure caused transient damage to the plasma membrane, which increased the permeability of the cells to exogenous molecules. This "filtroporation" was indicated by uptake of fluorescent dextran molecules up to 500,000 MW in cells deemed viable by trypan blue dye exclusion. The macromolecular uptake was increased if the driving pressure was increased at constant micropore size, or if the micropore size was decreased at constant driving pressure. Larger membrane perturbations permitted uptake of a luciferase reporter plasmid, which resulted in transfection of the CHO cells with the surviving cells expressing luciferase activity after 2 days in culture. This simple and general new method of porating cells in suspension may be optimized to incorporate the desired macromolecules while retaining the maximum viability.

396 ||| 10356250 ||| (2, 0.33) ||| 2 ||| 2 ||| Stable production of a human growth hormone antagonist from CHO cells adapted to serum-free suspension culture. Human growth hormone (hGH) is a polypeptide with 191 amino acids and a molecular mass of 22 kDa. An hGH analogue was created with a single amino acid substitution (glycine[G] 120 to arginine[R]) in the third alpha-helix of the hGH molecule. This hGH analogue, named hGHG120R, was found to be an hGH antagonist. It is a parenteral drug candidate for treating conditions in which hGH levels are abnormally high, as found in type I diabetics. Previously, a genetically engineered anchorage-dependent mouse L cell line was created that produced and secreted hGHG120R in culture media (Dulbecco's modified Eagle's medium, DMEM) supplemented with 5% NuSerum IV. A multistep downstream process was developed to purify hGHG120R. The process consisted of cell clarification, salt precipitation, membrane ultrafiltration, size exclusion chromatography, reversed phase high-performance liquid chromatography, phase separation, and lyophilization. Here, we present the development of a superior eukaryotic system using a proper combination of genetic elements, cell line, and media formulation. This system is suitable for the large-scale production of the recombinant protein and is superior to the previously developed system in that it increases the specific production rate and at the same time eases the burden of the purification process, in both time and efficiency. Dihydrofolate reductase mutant (DHFR-) Chinese hamster ovary (CHO) cells were used that were stably transfected with an expression vector in which the hGHG120R gene is driven by the relatively strong human cytomegalovirus-early gene regulatory region. The hGHG120R tested to be biologically active. These cells were then adapted to grow in suspension in CHO-S-SFM (serum-free media). High cell densities, typically 2.0 x 10(6) cells/mL were obtained from spinner flask cultures. Partial purification of hGHG120R from CHO cell cultured media revealed that the level of impurities in SFM was significantly lower than the serum-supplemented DMEM. This suggests that the salt precipitation and the SEC step need not be employed in the purification of hGHG120R from SFM. This would result in a reduction of the operating time by 50 h and boost the recovery yield of hGHG120R to 75%.

397 ||| 9736393 ||| (2, 0.32),(3, 0.30) ||| 2 ||| 2 ||| Expression and bioactivity of recombinant canine erythropoietin. OBJECTIVE: To produce recombinant canine erythropoietin (rcEPO) and compare its biological activity with that of recombinant human EPO (rhEPO). ANIMALS: C57BL/6J mice. PROCEDURE: The gene encoding cEPO was isolated from a genomic library and subcloned into an eucaryotic expression vector. Production of rcEPO was achieved by stable transfection of the expression construct into Chinese hamster ovary cells. Biological activity was evaluated in vitro by analyzing the mitogenic activity of rcEPO on murine erythroid progenitor cells. In vivo bioactivity was assessed in mice by measuring the ability of rcEPO to increase blood reticulocyte counts. RESULTS: Size and glycosylation of rcEPO expressed in Chinese hamster ovary cells were similar to values for commercial rhEPO. Canine and human EPO stimulated proliferation of murine erythroid progenitor cells in vitro and murine reticulocytosis in vivo in a dose-dependent manner. CONCLUSIONS: Comparable biological activity was observed for rcEPO and rhEPO in the 2 murine-based assay systems studied. By avoiding interspecies variation in protein structure and the resulting potential for immunogenicity, rcEPO should represent a better option than rhEPO for treatment of dogs with erythropoietin-dependent anemia. CLINICAL RELEVANCE: Therapeutic use of rhEPO in companion animals is limited by its immunogenicity and the resulting potential to induce pure red cell aplasia. Development and availability of species-specific EPO preparations should avoid this problem.

398 ||| 24850296 ||| (2, 0.31) ||| 2 ||| 2 ||| A mechanistic dissection of polyethylenimine mediated transfection of CHO cells: to enhance the efficiency of recombinant DNA utilization. In this study, we examine the molecular and cellular interactions that underpin efficient internalization and utilization of polyethylenimine (PEI):DNA complexes (polyplexes) by Chinese Hamster Ovary (CHO) cells. Cell surface polyplex binding and internalization was a biphasic process, consisting of an initial rapid Phase (I), lasting approximately 15 min, followed by a slower second Phase (II), saturating at approximately 240 min post transfection. The second Phase accounted for the majority (60-70%) of polyplex internalization. While cell surface heparan sulphate proteoglycans (HSPGs) were rapidly cointernalized with polyplexes during Phase I, cell surface polyplex binding was not dependent on HSPGs. However, Phase II polyplex internalization and HSPG regeneration onto the surface of trypsinized cells occurred at similar rates, suggesting that the rate of recycling of HSPG-containing membrane to the plasma membrane limits Phase II internalization rate. Under optimal transfection conditions, polyplexes had a near neutral surface charge (zeta potential) and cell surface binding was dependent on hydrophobic interactions, being significantly inhibited by both chemical sequestration of cholesterol from the plasma membrane and addition of nonionic surfactant. Induced alterations in polyplex zeta potential, using ferric (III) citrate to decrease surface charge and varying PEI:DNA ratio to increase surface charge, served to inhibit polyplex binding or reduce secreted alkaline phosphatase reporter expression and cell viability, respectively. To increase polyplex hydrophobicity and internalization an alkylated derivative of PEI, propyl-PEI, was chemically synthesized. Using Design of Experiments-Response Surface Modeling to optimize the transfection process, the function of propyl-PEI was compared to that of unmodified PEI in both parental CHO-S cells and a subclone (Clone 4), which exhibited superior transgene expression via an increased resistance to polyplex cytotoxicity. The combination of propyl-PEI and Clone 4 doubled the efficiency of recombinant DNA utilization and reporter protein production. These data show that for maximal efficacy, strategies to increase polyplex internalization into cells must be used in concert with strategies to offset the inherent cytotoxicity of this process.

399 ||| 22326251 ||| (2, 0.31) ||| 2 ||| 2 ||| Metabolic engineering of Chinese hamster ovary cells: towards a bioengineered heparin. Heparin is the most widely used pharmaceutical to control blood coagulation in modern medicine. A health crisis that took place in 2008 led to a demand for production of heparin from non-animal sources. Chinese hamster ovary (CHO) cells, commonly used mammalian host cells for production of foreign pharmaceutical proteins in the biopharmaceutical industry, are capable of producing heparan sulfate (HS), a related polysaccharide naturally. Since heparin and HS share the same biosynthetic pathway, we hypothesized that heparin could be produced in CHO cells by metabolic engineering. Based on the expression of endogenous enzymes in the HS/heparin pathways of CHO-S cells, human N-deacetylase/N-sulfotransferase (NDST2) and mouse heparan sulfate 3-O-sulfotransferase 1 (Hs3st1) genes were transfected sequentially into CHO host cells growing in suspension culture. Transfectants were screened using quantitative RT-PCR and Western blotting. Out of 120 clones expressing NDST2 and Hs3st1, 2 clones, Dual-3 and Dual-29, were selected for further analysis. An antithrombin III (ATIII) binding assay using flow cytometry, designed to recognize a key sugar structure characteristic of heparin, indicated that Hs3st1 transfection was capable of increasing ATIII binding. An anti-factor Xa assay, which affords a measure of anticoagulant activity, showed a significant increase in activity in the dual-expressing cell lines. Disaccharide analysis of the engineered HS showed a substantial increase in N-sulfo groups, but did not show a pattern consistent with pharmacological heparin, suggesting that further balancing the expression of transgenes with the expression levels of endogenous enzymes involved in HS/heparin biosynthesis might be necessary.

400 ||| 21464137 ||| (2, 0.31) ||| 2 ||| 2 ||| In vivo crystallization of human IgG in the endoplasmic reticulum of engineered Chinese hamster ovary (CHO) cells. Protein synthesis and secretion are essential to cellular life. Although secretory activities may vary in different cell types, what determines the maximum secretory capacity is inherently difficult to study. Increasing protein synthesis until reaching the limit of secretory capacity is one strategy to address this key issue. Under highly optimized growth conditions, recombinant CHO cells engineered to produce a model human IgG clone started housing rod-shaped crystals in the endoplasmic reticulum (ER) lumen. The intra-ER crystal growth was accompanied by cell enlargement and multinucleation and continued until crystals outgrew cell size to breach membrane integrity. The intra-ER crystals were composed of correctly folded, endoglycosidase H-sensitive IgG. Crystallizing propensity was due to the intrinsic physicochemical properties of the model IgG, and the crystallization was reproduced in vitro by exposing a high concentration of IgG to a near neutral pH. The striking cellular phenotype implicated the efficiency of IgG protein synthesis and oxidative folding exceeded the capacity of ER export machinery. As a result, export-ready IgG accumulated progressively in the ER lumen until a threshold concentration was reached to nucleate crystals. Using an in vivo system that reports accumulation of correctly folded IgG, we showed that the ER-to-Golgi transport steps became rate-limiting in cells with high secretory activity.

401 ||| 18626955 ||| (2, 0.30) ||| 2 ||| 2 ||| Deregulated expression of cloned transcription factor E2F-1 in Chinese hamster ovary cells shifts protein patterns and activates growth in protein-free medium. Engineering of the cell cycle can be an effective means for bypassing growth factor requirements of animal cells. Cloned human E2F-1 from Nalm 6 cells was subcloned into pRc/CMV and transfected into Chinese hamster ovary (CHO) cells. Ten stable transfectant clones isolated from cells cultured under neomycin-resistance selection pressure all expressed significantly higher amounts of E2F-1 than control cells as determined by Western analysis. Confocal immunofluorescent microscopy and Southern analysis of several clones also provided evidence for the expression of cloned E2F-1 in these cells. CHO K1:E2F-1 cells are able to proliferate on well-defined serum- and protein-free basal medium and exhibit an S-phase extended by 65% compared to CHO K1 cells mitogenically stimulated by basic fibroblast growth factor (bFGF). Two-dimensional electrophoresis of the intracellular proteins of E2F-1 clones shows an increase in 236 gene products compared to CHO K1 control cells, further verifying a functional regulatory role of cloned E2F-1 in CHO cells. Among these upregulated species is the cell cycle regulatory protein, cyclin A, which has already been shown to be regulated by E2F-1 in human fibroblasts. Overexpression of cloned E2F-1 in CHO cells is a potentially useful new strategy for bypassing serum requirements in mammalian cell culture. Furthermore, such cell cycle control stimulus-protein pattern response data can contribute to a clearer understanding of complex multigene networks involved in mammalian cell cycle regulation.

402 ||| 11807766 ||| (2, 0.30) ||| 2 ||| 2 ||| Inhibiting apoptosis in mammalian cell culture using the caspase inhibitor XIAP and deletion mutants. Lower yields and poorer quality of biopharmaceutical products result from cell death in bioreactors. Such cell death may occur from necrosis but is more commonly associated with apoptosis. During the process of programmed cell death or apoptosis, caspases become activated and cause a cascade of events that eventually destroy the cell. XIAP is the most potent caspase inhibitor encoded in the mammalian genome. The effectiveness of XIAP and its deletion mutants was examined in two cell lines commonly utilized in commercial bioreactors: Chinese hamster ovary (CHO) and 293 human embryonic kidney (293 HEK) cells. CHO cells undergo apoptosis as a result of various insults, including Sindbis virus infection and serum deprivation. In this study, we demonstrate that 293 HEK cells undergo apoptosis during Sindbis virus infection and exposure to the toxins, etoposide and cisplatin. Two deletion mutants of XIAP were created; one containing three tandem baculovirus iap repeat (BIR) domains and the other containing only the C-terminal RING domain, lacking the BIRs. Viability studies were performed for cells expressing each mutant and the wild-type protein on transiently transfected cells, as stable pools, or as stable clonal cell populations after induction of apoptosis by serum deprivation, Sindbis virus infection, etoposide, and cisplatin treatment. Expression of the wild-type XIAP inhibited apoptosis significantly; however, the XIAP mutant containing the three BIRs provided equivalent or improved levels of apoptosis inhibition in all cases. Expression of the RING domain offered no protection and was pro-apoptotic in transient expression experiments. With the aid of an N-terminal YFP fusion to each protein, distribution within the cell was visualized, and the wild-type and mutants showed differing intracellular accumulation patterns. While the wild-type XIAP protein accumulated primarily in aggregates in the cytosol, the RING mutant was enriched in the nucleus. In contrast, the deletion mutant containing the three BIRs was distributed evenly throughout the cytosol. Thus, protein engineering of the XIAP protein can be used to alter the intracellular distribution pattern and improve the ability of this caspase inhibitor to protect against apoptosis for two mammalian cell lines.

# ----------------------------- TOPIC3 -----------------------------

403 ||| 9788319 ||| (3, 0.99) ||| 3 ||| 3 ||| Application of capillary electrophoresis, liquid chromatography, electrospray-mass spectrometry and matrix-assisted laser desorption/ionization - time of flight - mass spectrometry to the characterization of recombinant human erythropoietin. High performance capillary electrophoresis (HPCE), high performance liquid chromatography (HPLC), matrix-assisted laser desorption/ionization - time of flight - mass spectrometry (MALDI-TOF-MS), on-line CE-electrospray ionization-mass spectrometry (CE-ESI-MS) and on-line LC-ESI-MS have been employed to characterize a heterogeneous glycoprotein, recombinant human erythropoietin (rHuEPO) expressed from Chinese hamster ovary (CHO) cells. The analysis was demonstrated through two specific levels of detail: the intact protein and tryptic digests of the protein. Six glycoforms of rHuEPO were separated by HPCE; seventeen tryptic fragments in a total of 21 nonglycosylated and glycosylated peptides were characterized; the O-linked glycopeptides were analyzed directly by CE-ESI-MS and LC-ESI-MS. In particular, four glycans of O-acetylation of sialic acid were identified in the O-linked glycosylated fragments. The molecular weight of rHuEPO was accurately determined by MALDI-TOF-MS.

404 ||| 9525914 ||| (3, 0.99) ||| 3 ||| 3 ||| Purification and characterization of a GDP-fucose:polypeptide fucosyltransferase from Chinese hamster ovary cells. A GDP-fucose:polypeptide fucosyltransferase was purified 5000-fold to homogeneity from Chinese hamster ovary cell extracts in the absence of detergent. The purification procedure included two affinity chromatographic steps using the acceptor substrate, a recombinant factor VII EGF-1 domain, and the donor substrate analog, GDP-hexanolamine, as ligands. The purified enzyme migrates as a single band of 44,000 daltons on SDS-polyacrylamide gel electrophoresis and is itself a glycoprotein with more than one high mannose type oligosaccharide chain with a total molecular weight of 4000. The Km values for factor VII EGF-1 domain and GDP-fucose are 15 and 6 microM, respectively. The Vmax is 2.5 micromol.min-1.mg-1. The presence of 50 mM Mn2+ increased the enzyme activity 17-fold, but Mn2+ was not absolutely required, since the enzyme exhibited some activity even in the presence of EDTA. The acceptor substrate specificity was studied using site-directed mutagenesis of human factor IX EGF domain. Only one of several differently folded species could serve as acceptor substrate, although they all had the same molecular weight as determined by liquid chromatography on-line with mass spectrometry. This indicates that the enzyme requires proper folding of the epidermal growth factor domain for its activity.

405 ||| 9234902 ||| (3, 0.99) ||| 3 ||| 3 ||| Sequencing of N-linked oligosaccharides directly from protein gels: in-gel deglycosylation followed by matrix-assisted laser desorption/ionization mass spectrometry and normal-phase high-performance liquid chromatography. A generally applicable, rapid, and sensitive method for profiling and sequencing of glycoprotein-associated N-linked oligosaccharides from protein gels was developed. The method employed sodium dodecyl sulfate-polyacrylamide gel electrophoresis (SDS-PAGE) for protein separation and purification and in-gel deglycosylation using PNGase F for glycan release. Profiles of the neutral glycans from bovine ribonuclease B, chicken ovalbumin, and human immunoglobulin G (IgG), as well as sialic acid-containing sugars (following esterification of the acidic groups) of bovine fetuin and bovine alpha1-acid glycoprotein, were obtained by matrix-assisted laser desorption/ionization mass spectrometry (MALDI MS) and by normal-phase high-performance liquid chromatography following fluorescent labeling. Oligosaccharides were sequenced using specific exoglycosidases, and digestion products were analyzed by MALDI MS. Between 50 and 100 pmol (1.5 to 15 microg) of glycoprotein applied to the gel was sufficient to characterize its oligosaccharide contents. The identity of all glycoproteins investigated could be confirmed after deglycosylation by in-gel trypsin treatment followed by MALDI MS mass mapping and matching the measured molecular weights to a sequence database. The technique was used for the characterization of the glycan moieties of human immunodeficiency virus recombinant gp120 (Chinese hamster ovary cells) and to monitor changes in the glycosylation of this glycoprotein when produced in the presence of a glucosidase I inhibitor. Furthermore, since heavy and light chains of IgG became separated by SDS-PAGE, it could be established that most glycans were associated with the heavy chains.

406 ||| 23339644 ||| (3, 0.99) ||| 3 ||| 3 ||| Characterization of host-cell line specific glycosylation profiles of early transmitted/founder HIV-1 gp120 envelope proteins. Glycosylation plays an essential role in regulating protein function by modulating biological, structural, and therapeutic properties. However, due to its inherent heterogeneity and diversity, the comprehensive analysis of protein glycosylation remains a challenge. As part of our continuing effort in the analysis of glycosylation profiles of recombinant HIV-1 envelope-based immunogens, we evaluated and compared the host-cell specific glycosylation pattern of recombinant HIV-1 surface glycoprotein, gp120, derived from clade C transmitted/founder virus 1086.C expressed in Chinese hamster ovary (CHO) and human embryonic kidney containing T antigen (293T) cell lines. We used an integrated glycopeptide-based mass mapping workflow that includes a partial deglycosylation step described in our previous study with the inclusion of a fragmentation technique, electron transfer dissociation (ETD), to complement collision-induced dissociation. The inclusion of ETD facilitated the analysis by providing additional validation for glycopeptide identification and expanding the identified glycopeptides to include coverage of O-linked glycosylation. The site-specific glycosylation analysis shows that the transmitted/founder 1086.C gp120 expressed in CHO and 293T displayed distinct similarities and differences. For N-linked glycosylation, two sites (N386 and N392) in the V4 region were populated with high mannose glycans in the CHO cell-derived 1086.C gp120, while these sites had a mixture of high mannose and processed glycans in the 293T cell-derived 1086.C gp120. Compositional analysis of O-linked glycans revealed that 293T cell-derived 1086.C gp120 consisted of core 1, 2, and 4 type O-linked glycans, while CHO cell-derived 1086.C exclusively consisted of core 1 type O-linked glycans. Overall, glycosylation site occupancy of the CHO and 293T cell-derived 1086.C gp120 showed a high degree of similarity except for one site at N88 in the C1 region. This site was partially occupied in 293T-gp120 but fully occupied in CHO-gp120. Site-specific glycopeptide analysis of transmitted/founder 1086.C gp120 expressed in CHO cells revealed the presence of phosphorylated glycans, while 293T cell-produced 1086.C gp120 glycans were not phosphorylated. While the influence of phosphorylated glycans on immunogenicity is unclear, distinguishing host-cell specific variations in glycosylation profiles provide insights into the similarity (or difference) in recombinant vaccine products. While these differences had minimal effect on envelope antigenicity, they may be important in considering immunogenicity and functional capacities of recombinant envelope proteins produced in different expression systems.

407 ||| 21561106 ||| (3, 0.99) ||| 3 ||| 3 ||| Cell type-specific and site directed N-glycosylation pattern of FcgammaRIIIa. Human leukocyte receptor IIIa (hFcgammaRIIIa) plays a prominent role in the elimination of tumor cells by antibody-based cancer therapies. In previous studies, a major impact of the presence of carbohydrates at Asn-162 on the binding between the receptor and the Fc part of wild type fucosylated or glycoengineered nonfucosylated antibodies has been shown. In this study, we performed a site directed carbohydrate analysis at hFcgammaRIIIa derived from human embryonic kidney (HEK) and Chinese hamster ovary (CHO) cells, respectively. Using mass spectrometry (MS) and a multienzyme protein digest, we analyzed the proteolysis-generated glycopeptides in detail. We could show that hFcgammaRIIIa expressed by HEK cells was mostly bearing multifucosylated biantennary Asn162-glycans with a major fraction terminating with GalNAc residues replacing the more common Gal. We could demonstrate that the glycan antennae with terminal GalNAc could be sialylated as indicated by a novel reporter ion HexNAcHexNAcNeuAc(+) (m/z 698.28) using a source induced dissociation (SID) scan in the MS cycle. In contrast to the hFcgammaRIIIa Asn-162 glycosylation pattern from HEK cells, the CHO cells derived receptor contains bi- and triantennary galactosylated and highly sialylated carbohydrates. Our data suggest that the type of expression host system was a dominating factor for formation of distinct glycopatterns of hFcgammaRIIIa, while the protein sequence and the site of glycosylation remained unchanged for both types of cells. Using surface plasmon resonance (SPR) interaction analysis, we show that the cell type and site specific glycosylation pattern of hFcgammaRIIIa influences its binding behavior to immunoglobulin molecules.

408 ||| 15454184 ||| (3, 0.99) ||| 3 ||| 3 ||| Analysis of site-specific glycosylation in recombinant human follistatin expressed in Chinese hamster ovary cells. Follistatin (FS), a glycoprotein, plays an important role in cell growth and differentiation through the neutralization of the biological activities of activins. In this study, we analyzed the glycosylation of recombinant human FS (rhFS) produced in Chinese hamster ovary cells. The results of SDS-PAGE and MALDI-TOF MS revealed the presence of both non-glycosylated and glycosylated forms. FS contains two potential N-glycosylation sites, Asn95 and Asn259. Using mass spectrometric peptide/glycopeptide mapping and precursor-ion scanning, we found that both N-glycosylation sites were partially glycosylated. Monosaccharide composition analyses suggested the linkages of fucosylated bi- and triantennary complex-type oligosaccharides on rhFS. This finding was supported by mass spectrometric oligosaccharide profiling, in which the m/z values and elution times of some of the oligosaccharides from rhFS were in good agreement with those of standard oligosaccharides. Site-specific glycosylation was deduced on the basis of the mass spectra of the glycopeptides. It was suggested that biantennary oligosaccharides are major oligosaccharides located at both Asn95 and Asn259, whereas the triantennary structures are present mainly at Asn95.

409 ||| 9455918 ||| (3, 0.97) ||| 3 ||| 3 ||| Structural variations in the glycosaminoglycan-protein linkage region of recombinant decorin expressed in Chinese hamster ovary cells. Decorin is a small fibroblast proteoglycan consisting of a core protein and a single chondroitin/dermatan sulfate chain. The structure of the carbohydrate-protein linkage region of the recombinant decorin expressed in Chinese hamster ovary cells was investigated. The decorin was secreted in the culture medium and isolated by anion-exchange chromatography. The glycosaminoglycan chain was released from the decorin by beta-elimination using alkaline NaBH4, and then digested with chondroitinase ABC. These treatments resulted in a major and a few minor hexasaccharide alditols derived from the carbohydrate-protein linkage region. Their structures were analyzed by enzymatic digestion in conjunction with high-performance liquid chromatography. Two of these compounds have the conventional hexasaccharide core, deltaHexA alpha1-3GalNAc beta1-4GlcA beta1-3Gal beta1-3Gal beta1-4Xyl-ol. One is nonsulfated, and the other is monosulfated on C4 of the GalNAc residue. They represent 12% and 60% of the total linkage region, respectively. The other compound has the hexasaccharide alditol with an internal iduronic acid residue deltaHexA alpha1-3GalNAc(4-sulfate)beta1-4IdoA alpha1-3Gal beta1-3Gal beta1-4Xyl-ol, which was previously demonstrated in one of the five linkage hexasaccharide alditols isolated from dermatan sulfate proteoglycans of bovine aorta (Sugahara et al., J. Biol. Chem., 270, 7204-7212, 1995). The compound accounts for 11% of the total linkage region. These structural variations in the linkage hexasaccharide region of the decorin strikingly contrast to the uniformity demonstrated in the linkage hexasaccharide structure of human inter-alpha-trypsin inhibitor (Yamada et al., Glycobiology, 5, 335-341, 1995) and urinary trypsin inhibitor (Yamada et al., Eur. J. Biochem., 233, 687-693, 1995), both of which have a single chondroitin sulfate chain with a uniform linkage hexasaccharide structure, deltaHexA alpha1-3GalNAc(4-sulfate)beta1-4GlcA beta1-3Gal(4-sulfate)beta1-3Gal beta1-4Xyl, containing a 4-O-sulfated Gal residue.

410 ||| 10572956 ||| (3, 0.97) ||| 3 ||| 3 ||| Study of the sugar chains of recombinant human amyloid precursor protein produced by Chinese hamster ovary cells. The N- and O-glycans of recombinant amyloid precursor protein (APP), purified from Chinese hamster ovary cells transfected with the human 695-amino acid form of APP, were separately released by hydrazinolysis under different conditions. The reducing ends of the released N- and O-glycans were reduced with NaB3H4 and derivatized with 2-aminobenzamide (2AB), respectively. After acidic N-glycans were obtained by anion-exchange column chromatography, these were converted to neutral oligosaccharides by sialidase digestion, demonstrating that their acidic nature was entirely due to sialylation. The sialidase-treated N-glycans were then fractionated by lectin column chromatography and their structures were determined by linkage-specific sequential exoglycosidase digestion. These results demonstrated that recombinant APP has bi- and triantennary complex type N-glycans with fucosylated and nonfucosylated trimannosyl cores. In a similar fashion, the 2AB-labeled O-glycans derived from APP were determined to be mono- and disialylated core type 1 structures. Taken together, these results indicate that recombinant APP has sialylated bi- and triantennary N-glycans with fucosylated and nonfucosylated cores and sialylated O-glycans with core type 1 structures.

411 ||| 8639592 ||| (3, 0.96) ||| 3 ||| 3 ||| Amino acid sequence and carbohydrate structure of a recombinant human tissue factor pathway inhibitor expressed in Chinese hamster ovary cells: one N-and two O-linked carbohydrate chains are located between Kunitz domains 2 and 3 and one N-linked carbohydrate chain is in Kunitz domain 2. Human tissue factor pathway inhibitor is a protease inhibitor with three tandem Kunitz-type inhibitory domains. The recombinant protein (r-hTFPI) was produced using Chinese hamster ovary cells, and its polypeptide and carbohydrate chain structures were analyzed. The complete amino acid sequence, composed of 276 residues, was determined using a protein sequencer after protease digestion and it was identical to that predicted from the cDNA sequence. Among three potential N-glycosylation sites, both Asn117 and Asn167 were fully N-glycosylated but Asn228 was not. Thr175 was also fully O-glycosylated, but Ser174 was partially O-glycosylated. Carbohydrate composition and mass spectrometric analyses of the undecapeptide OG-11 (residues Leu 170approximately Leu180) showed that two O-linked carbohydrate chains consisted of a type-1 core structure (Gal-GalNAc-Ser/Thr) with 0-3 mol of N-acetylneuraminic acid(s). The N-linked carbohydrate chains were analyzed by two-dimensional carbohydrate mapping combined with sequential glycosidase digestion, after the reducing-ends of carbohydrate residues were tagged with 2-aminopyridine and non-reducing-end sialic acids were removed with sialidase. All the N-linked structures in r-hTFPI were complex-type carbohydrate chains with one fucose residue attached to the reducing-end GlcNAc and consisted of bi-, tri-, and tetraantennary carbohydrate chains in the ratio 1.9:1.3:1.0. Fucosylated tri- and tetraantennary carbohydrate chains with one or two N-acetyllactosaminyl repeats were also found (30% of carbohydrate chains determined). Thus, the region between Kunitz domains 2 and 3 encoded by exon 7 was highly glycosylated by two O-linked carbohydrate chains at Ser174 and Thr175 and one N-linked carbohydrate chain at Asn167. These results indicated that the region is occupied by a cluster of three bulky and acidic carbohydrate chains.

412 ||| 21805521 ||| (3, 0.96) ||| 3 ||| 3 ||| Human Dickkopf-1 (huDKK1) protein: characterization of glycosylation and determination of disulfide linkages in the two cysteine-rich domains. Human Dickkopf-1 (huDKK1), an inhibitor of the canonical Wnt-signaling pathway that has been implicated in bone metabolism and other diseases, was expressed in engineered Chinese hamster ovary cells and purified. HuDKK1 is biologically active in a TCF/lef-luciferase reporter gene assay and is able to bind LRP6 coreceptor. In SDS-PAGE, huDKK1 exhibits molecular weights of 27-28 K and 30 K at ~ 1:9 ratio. By MALDI-MS analysis, the observed molecular weights of 27.4K and 29.5K indicate that the low molecular weight form may contain O-linked glycans while the high molecular weight form contains both N- and O-linked glycans. LC-MS/MS peptide mapping indicates that ~ 92% of huDKK1 is glycosylated at Asn225 with three N-linked glycans composed of two biantennary forms with 1 and 2 sialic acid (23% and 60%, respectively), and one triantennary structure with 2 sialic acids (9%). HuDKK1 contains two O-linked glycans, GalNAc (sialic acid)-Gal-sialic acid (65%) and GalNAc-Gal[sialic acid] (30%), attached at Ser30 as confirmed by beta-elimination and targeted LC-MS/MS. The 10 intramolecular disulfide bonds at the N- and C-terminal cysteine-rich domains were elucidated by analyses including multiple proteolytic digestions, isolation and characterization of disulfide-containing peptides, and secondary digestion and characterization of selected disulfide-containing peptides. The five disulfide bonds within the huDKK1 N-terminal domain are unique to the DKK family proteins; there are no exact matches in disulfide positioning when compared to other known disulfide clusters. The five disulfide bonds assigned in the C-terminal domain show the expected homology with those found in colipase and other reported disulfide clusters.

413 ||| 19652963 ||| (3, 0.95) ||| 3 ||| 3 ||| Glycosylation pattern of humanized IgG-like bispecific antibody produced by recombinant CHO cells. The glycosylation pattern of a humanized anti-EGFRxanti-CD3 bispecific single-chain diabody with an Fc portion (hEx3-scDb-Fc) produced by recombinant Chinese hamster ovary cells was evaluated and compared with those of a recombinant humanized anti-IL-8 antibody (IgG1) and human serum IgG. N-Linked oligosaccharide structures were estimated by two-dimensional high-performance liquid chromatography and matrix-assisted laser desorption/ionization time-of-flight mass spectrometry. No sialylation was observed with purified hEx3-scDb-Fc and the anti-IL-8 antibody. From the analysis of neutral oligosaccharides, approximately more than 90% of the N-linked oligosaccharides of hEx3-scDb-Fc and the anti-IL-8 antibody were alpha-1,6-fucosylated. The galactosylated biantennary oligosaccharides comprise over 40% of the total N-linked oligosaccharides in both hEx3-scDb-Fc and the anti-IL-8 antibody. The fully galactosylated biantennary oligosaccharides from hEx3-scDb-Fc and the anti-IL-8 antibody accounted for only 10% of the N-linked; however, more than 20% of the N-linked oligosaccharides were fully galactosylated biantennary oligosaccharides in human serum IgG. The glycosylation pattern of hEx3-scDb-Fc was quite similar to that of the anti-IL-8 antibody.

414 ||| 9572875 ||| (3, 0.94) ||| 3 ||| 3 ||| Identification of O-glycosylation sites and partial characterization of carbohydrate structure and disulfide linkages of human insulin-like growth factor binding protein 6. The actions of insulin-like growth factors (IGFs) are modulated by a family of high-affinity binding proteins (IGFBPs), including IGFBP-6, which preferentially binds IGF-II and is O-glycosylated. Glycosylated and nonglycosylated recombinant human IGFBP-6, expressed in Chinese hamster ovary cells and Escherichia coli, respectively, were purified using IGF-II affinity chromatography and reverse-phase medium-pressure chromatography. Electrospray ionization mass spectrometry (ESMS) of glycosylated IGFBP-6 revealed considerable heterogeneity of carbohydrate composition. Major glycoforms contained 8-16 monosaccharides, including N-acetylhexosamine, hexose, and N-acetylneuraminic acid. Glycosylation sites of IGFBP-6 were identified as Thr126, Ser144, Thr145, Thr146, and Ser152 by using a combination of ESMS and Edman sequencing of tryptic fragments separated by reverse-phase high-pressure liquid chromatography. One oligosaccharide chain contained 5-6 monosaccharides, whereas the others contained 2-4 monosaccharides. Glycosylated IGFBP-6 exhibited greater resistance to proteolysis by chymotrypsin and trypsin than nonglycosylated IGFBP-6. Native disulfide bond positions in IGFBP-6 were localized by means of observed disulfide-linked tryptic fragments, revealing that there are two disulfide-linked subdomains within each of the N- and C-terminal regions and confirming a previous suggestion that the latter regions are not interconnected. A model of IGFBP-6 is developed in which these distinct domains are separated by a central region which is O-glycosylated.

415 ||| 10704524 ||| (3, 0.93) ||| 3 ||| 3 ||| Human alpha-N-acetylgalactosaminidase: site occupancy and structure of N-linked oligosaccharides. Human alpha-N-acetylgalactosaminidase (alpha-GalNAc; also known as alpha-galactosidase B) is the lysosomal exoglycohydrolase that cleaves alpha-N-acetylgalactosaminyl moieties in glycoconjugates. Mutagenesis studies indicated that the first five (N124, N177, N201, N359, and N385) of the six potential N-glycosylation sites were occupied. Site 3 occupancy was important for enzyme function and stability. Characterization of the N-linked oligosaccharide structures on the secreted enzyme overexpressed in Chinese hamster ovary cells revealed highly heterogeneous structures consisting of complex (approximately 53%), hybrid (approximately 12%), and high mannose-type (approximately 33%) oligosaccharides. The complex structures were mono-, bi-, 2,4-tri-, 2,6-tri-, and tetraantennary, among which the biantennary structures were most predominant (approximately 53%). Approximately 80% of the complex oligo-saccharides had a core-region fucose and 50% of the complex oligosaccharides were sialylated exclusively with alpha-2,3-linked sialic acid residues. The majority of hybrid type oligo-saccharides were GalGlcNAcMan(6)GlcNAc-Fuc(0-1)GlcNAc. Approximately 54% of the hybrid oligosaccharide were phosphorylated and one-third of these structures were further sialylated, the latter representing unique phosphorylated and sialylated structures. Of the high mannose oligosaccharides, Man(5-7)GlcNAc(2) were the predominant species (approximately 90%) and about 50% of the high mannose oligosaccharides were phosphorylated, exclusively as monoesters whose positions were determined. Comparison of the oligosaccharide structures of alpha-GalNAc and alpha-galactosidase A, an evolutionary-related and highly homologous exoglycosidase, indicated that alpha-GalNAc had more completed complex chains, presumably due to differences in enzyme structure/domains, rate of biosynthesis, and/or aggregation of the overexpressed recombinant enzymes.

416 ||| 7629081 ||| (3, 0.92) ||| 3 ||| 3 ||| Purification of recombinant porcine m2 muscarinic acetylcholine receptor from Chinese hamster ovary cells. Circular dichroism spectra and ligand binding properties. The recombinant porcine m2 muscarinic acetylcholine receptor (rPm2R) from Chinese hamster ovary cells has been purified to homogeneity. Two mg of purified rPm2R, with a specific activity of 12 nmol of R-(-)-quinuclidinyl benzilate/mg of protein, were obtained from 30 ml of packed Chinese hamster ovary cells. The apparent molecular mass (78.5 kDa) and specific activity for the rPm2R preparation were the same as that for the Pm2R purified from atrial tissue, but the yield was 100 times greater. Purified rPm2R bound agonist and antagonist with the same affinities and coupled to the inhibitory guanine nucleotide-binding protein with the same efficiency as the purified native atrial Pm2R. Ligand binding studies were consistent with a single class of antagonist binding sites but two subclasses of agonist binding sites. The fraction of rPm2R having high affinity for agonists was increased by mM Mg2+, low detergent concentration, and low temperature. Circular dichroism spectra obtained for the purified rPm2R with and without agonists were indistinguishable, but spectra for the antagonist-occupied receptor showed reproducibly deeper characteristic negative deflections at 208 and 220 nm. Secondary structure analysis of the CD spectra predicted 53% alpha-helix for the free receptor and 49% alpha-helix for the R-(-)-quinuclidinyl benzilate-receptor complex.

417 ||| 11263562 ||| (3, 0.92) ||| 3 ||| 3 ||| Selective glycopeptide mapping of erythropoietin by on-line high-performance liquid chromatography--electrospray ionization mass spectrometry. Selective glycopeptide mapping of recombinant human erythropoietin (rhEPO) used as a model glycoprotein was successfully carried out by on-line high-performance liquid chromatography-electrospray ionization mass spectrometry (LC-ESI-MS) using a Vydac C18 column eluted in acetonitrile-1 mM ammonium acetate, pH 6.8. rhEPO expressed in a Chinese hamster ovary clone was exhaustively digested into four glycopeptides and nine peptides with endoproteinase Glu-C. Both glycopeptides and peptides were eluted with trifluoroacetic acid as the eluent, whereas only glycopeptides were eluted selectively with ammonium acetate in the following order: N38, N24, 0126, and N83. Furthermore, many glycoforms included in each glycopeptide were found to be separated by differences in the numbers of sialic acid and N-acetyllactosaminyl repeats. Twenty, 16 and 22 different N-linked oligosaccharides were determined at Asn24, 38, and 83, respectively, and two different O-linked oligosaccharides were observed at Ser126. Our method is simple, rapid, and useful for determining the carbohydrate structures at each glycosylation site and for elucidating the site-specific carbohydrate heterogeneity.

418 ||| 10998266 ||| (3, 0.92) ||| 3 ||| 3 ||| Application of liquid chromatography/mass spectrometry and liquid chromatography with tandem mass spectrometry to the analysis of the site-specific carbohydrate heterogeneity in erythropoietin. High-performance liquid chromatography with electrospray ionization mass spectrometry (LC/MS) and liquid chromatography with tandem mass spectrometry (LC/MS/MS) were applied to the analysis of the site-specific carbohydrate heterogeneity in erythropoietin (EPO) used as a model of the sialylated glycoprotein. N-linked oligosaccharides were released from recombinant human EPO expressed in Chinese hamster ovary cells enzymatically and reduced with NaBH(4). Many different sialylated oligosaccharides of EPO were separated and characterized by LC/MS equipped with a graphitized carbon column (GCC). Glycosylation sites and the preliminary glycosylation pattern at each glycosylation site were determined by LC/MS of endoproteinase Glu-C-digested EPO. The detailed site-specific carbohydrate heterogeneity caused by the differences in the molecular weight, branch, linkage, and sequence was elucidated by GCC-LC/MS of the N-linked oligosaccharides released from the isolated glycopeptides. Structural details of the isomers were analyzed by LC/MS/MS, and it was indicated that di- and trisialylated tetraantennary oligosaccharides are attached to Asn24, 38, and 83, whereas their isomers, di- and trisialylated triantennary oligosaccharides containing N-acetyllactosamines, are combined with Asn24. Our method is useful for the determination of glycosylation sites, the site-specific carbohydrate heterogeneity of glycoproteins, and the carbohydrate structure.

419 ||| 10362837 ||| (3, 0.91) ||| 3 ||| 3 ||| The O-linked fucose glycosylation pathway: identification and characterization of a uridine diphosphoglucose: fucose-beta1,3-glucosyltransferase activity from Chinese hamster ovary cells. O-Linked fucose is an unusual carbohydrate modification in which fucose is linked directly to the hydroxyl groups of serines or threonines. It has been found on the epidermal growth factor-like modules of several secreted proteins involved in blood coagulation and fibrinolysis. We have recently reported the existence of an elongated form of O-linked fucose in Chinese hamster ovary cells consisting of a glucose linked to the 3'-hydroxyl of fucose (Glcbeta1,3Fuc- O-Ser/Thr). This structure is highly unusual for two reasons. First, in mammalian systems fucose is usually a terminal modification of N - and O-linked oligosaccharides. Here the fucose is internal. Secondly, terminal beta-linked glucose is extremely rare on mammalian glycoconjugates. Thus, the Glcbeta1,3Fuc structure is a very unique mammalian carbohydrate structure. Here we report the identification and initial characterization of a novel enzyme activity capable of forming this unique linkage: UDP-glucose: O-linked fucose beta1,3 glucosyltransferase. The enzyme utilizes UDP-glucose as the high energy donor and transfers glucose to alpha-linked fucose residues. The activity is linearly dependent on time, enzyme, and substrate concentrations and is enhanced in the presence of manganese ions. Activity is present in extracts of cultured cells from a variety of species (hamster, human, mouse, rat, chicken) and is enriched in brain and spleen of a normal adult rat. Thus, while this glycosyltransferase appears to be widespread in biology, it forms a very unique linkage, and it represents the first mammalian enzyme identified capable of elongating fucose.

420 ||| 11159927 ||| (3, 0.90) ||| 3 ||| 3 ||| In vivo trafficking and catabolism of IgG1 antibodies with Fc associated carbohydrates of differing structure. We have now produced mouse-human chimeric IgG1 in wild-type Chinese hamster ovary (CHO) cell lines Pro-5 as well as in the glycosylation mutants Lec 2, Lec 8, and Lec 1. Analysis of the attached carbohydrates shows those present on IgG1-Lec 1 were mannose terminated. Carbohydrate present on IgG1-Lec8 was uniformly biantennary terminating in N-acetylglucosamine. The glycosylation profiles of IgG1-Lec 2 and IgG1-Pro-5 were heterogeneous. Only IgG1-Pro-5 was sialylated with sialic acid present on only a small percentage of the carbohydrate structures. When the in vivo fate of antibodies labeled with (125)I-lactotyramine was determined, it was found that the majority of all of the antibodies, irrespective of the structure of their attached carbohydrate, is catabolized in the skin and muscle. However, the attached carbohydrate structure does influence the amount that is catabolized in the liver and the liver serves as a major site for the catabolism of proteins bearing carbohydrate with the Lec2 (with terminal galactose) or Lec1(with terminal mannose) structure.

421 ||| 9603907 ||| (3, 0.89) ||| 3 ||| 3 ||| Gain-of-function Chinese hamster ovary mutants LEC18 and LEC14 each express a novel N-acetylglucosaminyltransferase activity. LEC18 and LEC14 cells are gain-of-function glycosylation mutants isolated from Chinese hamster ovary cells for resistance to pea lectin. Structural studies have shown that LEC18 cells synthesize complex N-glycans with a GlcNAc residue linked at the O-6 position of the core GlcNAc (Raju, T. S., Ray, M. K., and Stanley, P. (1995) J. Biol. Chem. 270, 30294-30302), whereas LEC14 cells synthesize complex N-glycans with a GlcNAc residue linked at the O-2 position of the core beta-linked Man residue (Raju, T. S., and Stanley, P. (1996) J. Biol. Chem. 271, 7484-7493). Both modifications are novel and have not been reported in glycoproteins from any other source. We now show that, in both LEC18 and LEC14 cells, GlcNAc transfer is mediated by a distinct N-acetylglucosaminyltransferase (GlcNAc-T) activity. The LEC18 activity, termed GlcNAc-TVIII, transfers GlcNAc to GlcNAcbeta1-O-pNP and to a GlcNAc-terminating, biantennary, complex N-glycan, with or without a core fucose. By contrast, the LEC14 transferase, termed GlcNAc-TVII, does not have significant activity with simple acceptors, and transfers GlcNAc preferentially to a GlcNAc-terminating biantennary glycopeptide that contains a core fucose residue. The acceptor specificities and other biochemical properties of GlcNAc-TVII and GlcNAc-TVIII differ from previously characterized GlcNAc-transferases including GlcNAc-TIII, indicating that they represent new members of the mammalian GlcNAc-T group of transferases.

422 ||| 23424082 ||| (3, 0.89) ||| 3 ||| 3 ||| Mucin-type fusion proteins with blood group A or B determinants on defined O-glycan core chains produced in glycoengineered Chinese hamster ovary cells and their use as immunoaffinity matrices. Assays for quantification, and methods for removal, of anti-A and anti-B antibodies are the key for the success of ABO incompatible organ transplantation programs. In order to produce tools that can be used as substrates in tests for anti-A/anti-B quantification and specificity determination or as affinity matrices in extracorporeal immunoadsorption (IA) columns, we engineered Chinese hamster ovary (CHO) cells secreting mucin-type fusion proteins carrying blood group A or B determinants on defined O-glycan core saccharide chains. Besides the P-selectin glycoprotein ligand-1/mouse immunoglobulin G(2b) (PSGL-1/mIgG(2b)) cDNA, CHO cells were transfected with plasmids encoding core 2 (beta1,6GlcNAc-T1) or core 3 (beta1,3GlcNAc-T6 and beta1,3Gal-T5) enzymes together with alpha1,2Fuc-T1 or alpha1,2Fuc-T2 and the A or B gene-encoded alpha1,3GalNAcT or alpha1,3Gal-T, respectively. Selected clones with the correct glycophenotype were expanded and cultured in shaker flasks and Wave bioreactors. Western blotting was used to characterize purified fusion protein and liquid chromatography-mass spectrometry was used to characterize the released O-glycans. Clones producing PSGL-1/mIgG(2b) carrying O-glycans with A and B determinants on type 1 (Galbeta3GlcNAc), type 2 (Galbeta4GlcNAc) and type 3 (Galbeta3GalNAcalpha) outer core saccharide chains were established. The conversion of CHO cells from exclusive inner core 1 (Galbeta3GalNAc) to core 3 (GlcNAcbeta3GalNAc) O-glycan producers was almost complete, whereas conversion to inner core 2 (GlcNAcbeta6GalNAc) O-glycans was incomplete as was the alpha2-fucosylation of the core 1 chain. Sialylation may prevent these biosynthetic steps. The clinical utility of the blood group A and B substituted mucin-type fusion proteins as substrates in enzyme-linked immunosorbent assay or as affinity matrices in IA columns is explored.

423 ||| 8639667 ||| (3, 0.88) ||| 3 ||| 3 ||| Cell line and site specific comparative analysis of the N-linked oligosaccharides on human ICAM-1des454-532 by electrospray ionization mass spectrometry. Sialylated oligosaccharide structures were determined by the technique of electrospray ionization mass spectroscopy at seven of eight N-linked glycosylation sites of recombinant human ICAM-1des454-532 [tICAM(453)] purified from the tissue culture fluid of Chinese hamster ovary, human embryonic kidney, and mouse myeloma cell lines. The number of structures at each site depended on the cell line and ranged from 8 to 34. N-Glycolyneuraminic acid, a human oncofetal antigen, was found at all sites of all three cell line derived forms of tICAM(453). Tetraantennary complex structures containing one and/or two galactose-beta 1,4 N-acetylglucosamine repeats, characteristic of membrane bound proteins, were found on soluble tICAM(453) primarily at Asn-379. Asn-379, located between the D4 and D5 domains, is believed to be located close to the membrane surface in membrane bound ICAM-1. It has been proposed that the extent of N-linked glycosylation at Asn-240 and Asn-269 in the third domain of ICAM-1 may regulate the binding avidity of ICAM-1 to Mac-1 [Diamond, M. S., Staunton, D. E., Marlin, S. D., & Springer, T. A. (1991) Cell 65, 961-971]. In the present study the tICAM(453) Asn-269 site was found to contain predominantly one oligosaccharide structure that is conserved in all three cell lines. On the other hand, the Asn-240 site was found to contain cell line dependent oligosaccharide structural heterogeneity particularly in the degree of sialylation.

424 ||| 23183444 ||| (3, 0.88) ||| 3 ||| 3 ||| O-linked glucosylation of a therapeutic recombinant humanised monoclonal antibody produced in CHO cells. An unpredictable modification of a therapeutic recombinant humanised monoclonal antibody (rh-mAbX) produced using CHO cells was found. LC/MS analysis of rh-mAbX indicated the presence of heterogeneity in the light chain with a corresponding mass shift of 162 Da compared to the theoretical mass. To characterise the heterogeneity, that is, the attached moiety, several analyses were performed. Peptide mapping of rh-mAbX indicated that the attached moiety was located in the amino acid sequence from Leu20 to Lys45, which is a part of the variable region of the light chain. The peptide was efficiently purified in two-steps by RP-HPLC by utilising two different types of RP columns. N-terminal sequencing and LC/MS/MS analysis of the peptide suggested that Ser29 of the light chain was the modification site, and that the attached moiety was a single O-linked hexose. HPAEC-PAD analysis following beta-elimination indicated the presence of an O-linked glucose in the modified peptide. Monosaccharide composition analysis after acid hydrolysis supported this result. The content of antibodies containing this species was determined to be approximately 10% by Lys-C peptide mapping detected at 280 nm. Thus, this study demonstrated the formation of a unique O-linked glucosylation posttranslational modification in a recombinant humanised monoclonal antibody produced in CHO cells.

425 ||| 10226368 ||| (3, 0.88) ||| 3 ||| 3 ||| Isolation, primary structure characterization and identification of the glycosylation pattern of recombinant goldfish neurolin, a neuronal cell adhesion protein. Neurolin is a growth-associated cell surface glycoprotein from goldfish and zebra fish which has been shown to be involved in axonal path-finding in the goldfish retina and suggested to function as a receptor for axon guidance molecules. Being a member of the immunoglobulin superfamily of cell adhesion proteins, neurolin consists of five N-terminal extracellular immunoglobulin (Ig)-like domains, a transmembrane and a short cytoplasmatic domain. Repeated injections of polyclonal Fab fragments against neurolin and of monoclonal antibodies against either Ig domains cause path-finding errors and disturbance of axonal fasciculation. In order to obtain a complete structural characterization and a molecular basis for structure-function determination, recombinant neurolin with the complete extracellular part but lacking the transmembrane and cytoplasmatic domain was expressed in Chinese hamster ovary (CHO) cells (CHO-neurolin). The isolation of CHO-neurolin was carried out by Ni-affinity chromatography and subsequent high-performance liquid chromatography (HPLC). An exact molecular mass determination was obtained by matrix-assisted laser desorption/ionization mass spectrometry (MALDI/MS) and revealed 60.9 kDa, which suggested that approximately 10 kDa are due to glycosylation. The predicted molecular mass is 51.5 kDa, whereas sodium dodecyl sulphate polyacrylamide gel electrophoresis (SDS-PAGE) yielded an apparent molecular mass of 72 kDa. Gel shift assays using SDS-PAGE and Western blot analysis with anti-neurolin antibodies provided consistent molecular mass data. The complete primary structure and N-glycosylation patterns were identified using specific lectin assays, MALDI/MS peptide mapping analysis by proteolytic and in-gel digestion, electrospray ionization MS and MALDI/MS in combination with specific glycosidase degradation. HPLC isolation of glycosylated peptide fragments and MS after selective deglycosylation revealed heterogeneous glycosylations at all five N-glycosylation consensus sites. All attached N-glycans are of the complex type and show a mainly biantennary structure; they are fucosylated with alpha(2,3)-terminal neuraminic acid. These data serve as a first detailed model to characterize the molecular recognition structures exhibited by the extracellular domains.

426 ||| 9450795 ||| (3, 0.87) ||| 3 ||| 3 ||| Epoetin alfa and beta differ in their erythropoietin isoform compositions and biological properties. Epoetin alfa and beta are the two forms of recombinant DNA-derived erythropoietin (rEPO), both synthesized in Chinese hamster ovary cells, which are used for the treatment of erythropoietin (EPO)-responsive anaemias. Several batches of each of these rEPOs were compared for differences in their EPO isoform compositions by isoelectric focusing (IEF) and in a range of lectin-binding assays, and for differences in their EPO activities by in-vivo and in-vitro mouse bioassays and by immunoassay. Epoetin beta was found to differ from epoetin alfa in containing: (a) a greater proportion of more basic isoforms, (b) a greater proportion of EPO binding to Erythrina cristagalli agglutinin (which binds N-glycans with nonsialylated outer Gal beta1-4GlcNAc moieties), and (c) isoforms with higher in-vivo:in-vitro bioactivity ratios. Epoetin beta also contained slightly more than epoetin alfa of EPO binding to Lycopersicon esculentum agglutinin (which binds N-glycans containing repeating Gal beta1-4GlcNAc sequences), to the leucoagglutinin of Phaseolus vulgaris (which binds tetraantennary and 2,6-branched triantennary N-glycans) and to Agaricus bisporus agglutinin (which binds Gal beta1-3GalNAc containing O-glycans). No differences were found between the two rEPOs in their binding to a further five lectins. The differences between the isoform composition of epoetin alfa and beta, and the smaller inter-batch differences appear to be due to differences in glycosylation. The higher murine in-vivo:in-vitro bioactivity ratio of epoetin beta compared to epoetin alfa could not be explained in terms of differences in their degrees of sialylation, but was consistent with differences in their pharmacokinetics and pharmacodynamics observed in human subjects. There have been no reports that epoetin alfa differs from epoetin beta in its clinical efficacy, but the differences between epoetin alfa and beta in some analytical systems suggest that there might be a need for separate international standards for these two types of rEPO.

427 ||| 8631778 ||| (3, 0.87) ||| 3 ||| 3 ||| LEC14, a dominant Chinese hamster ovary glycosylation mutant expresses complex N-glycans with a new N-acetylglucosamine residue in the core region. The Chinese hamster ovary cell (CHO) glycosylation mutant, LEC14, was previously selected for resistance to pea lectin (Pisum sativum agglutinin) and shown to behave dominantly. The lectin resistance properties of LEC14 cells are related to, but distinct from, those of LEC18, a dominant Chinese hamster ovary mutant that synthesizes complex N-glycans with a novel O-6-linked GlcNAc residue in the core region (Raju, T.S., Ray, M., and Stanley, P. (1995) J. Biol. Chem. 270, 30294-30302). Detailed structural studies of a complex N-glycan fraction from LEC14 cells have revealed yet another novel modification of the core region. [3H]Glc-labeled LEC14 cellular glycopeptides were desialylated, and the fraction that did not bind to concanavalin A-Sepharose was found to have an increased proportion of species that bound to tomato-agarose, and to ricin-agarose. 1H NMR spectroscopy and methylation linkage analysis of the tomato and ricin-bound fractions purified from approximately 10(10) LEC14 cells showed they were complex N-glycans containing a 2,3,6-trisubstituted core Man residue. To examine the core region more closely, these N-glycans were digested with mixtures of beta-D-galactosidases and N-acetyl-beta-D-glucosaminidases to obtain core glycopeptides. The latter were largely unbound by concanavalin A-Sepharose or pea lectin-agarose. 1H NMR spectroscopy and electrospray ionization-mass spectrometry showed that the LEC14 core glycopeptides contain a new GlcNAc residue that substitutes the core beta(1-4)-Man residue at O-2 to give the following novel, N-linked core structure. [structure: see text]

428 ||| 11676606 ||| (3, 0.87) ||| 3 ||| 3 ||| Determination of carbohydrate structures N-linked to soluble CD154 and characterization of the interactions of CD40 with CD154 expressed in Pichia pastoris and Chinese hamster ovary cells. CD40-CD154 (CD40 ligand) interactions are essential for the development of protective immunity. Previous studies have described the CD40 binding site as a shallow groove formed between two monomers of CD154. However, these studies have not examined the structure or biological function of the carbohydrate on CD154. Human CD154 contains a single N-linked glycosylation site at asparagine 240. We have characterized the interactions between CD40 and soluble (s) CD154 in which sCD154 contains different types of carbohydrates. Detailed carbohydrate analysis revealed high-mannose structures on sCD154 purified from Pichia pastoris, whereas CD154 purified from Chinese hamster ovary E1A contained heterogeneous populations of complex carbohydrates. sCD154 purified from either system was trimeric, it bound to CD40 with similar affinities of 10-30 nM, and it functionally induced CD69 and CD95 expression on primary B cells. Together, these results indicate that the presence of varied types of N-linked glycans on asparagine 240 of CD154 does not play a significant role in the CD40-CD154 interactions.

429 ||| 10353820 ||| (3, 0.87) ||| 3 ||| 3 ||| The effect of O-fucosylation on the first EGF-like domain from human blood coagulation factor VII. The first epidermal growth factor-like domain (EGF-1) from blood coagulation factor VII (FVII) contains two unusual O-linked glycosylation sites at Ser-52 and Ser-60. We report here a detailed study of the effect of O-fucosylation at Ser-60 on the structure of FVII EGF-1, its Ca2+-binding affinity, and its interaction with tissue factor (TF). The in vitro fucosylation of the nonglycosylated FVII EGF-1 was achieved by using O-fucosyltransferase purified from Chinese hamster ovary cells. Distance and dihedral constraints derived from NMR data were used to determine the solution structures of both nonglycosylated and fucosylated FVII EGF-1 in the presence of CaCl2. The overall structure of fucosylated FVII EGF-1 is very similar to the nonfucosylated form even for the residues near the fucosylation site. The Ca2+ dissociation constants (Kd) for the nonfucosylated and fucosylated FVII EGF-1 were found to be 16.4 +/- 1.8 and 8.6 +/- 1.4 mM, respectively. The FVII EGF-1 domain binds to the extracellular part of TF with a low affinity (Kd approximately 0. 6 mM), and the addition of fucose appears to have no effect on this affinity. These results indicate that the FVII EGF-1 alone cannot form a tight complex with TF and suggest that the high binding affinity of FVIIa for TF requires cooperative interaction among the four domains in FVII with TF. Although the fucose has no significant effect on the interaction between TF and the individual FVII EGF-1 domain, it may affect the interaction of full-length FVIIa with TF by influencing its Ca2+-binding affinity.

430 ||| 23065139 ||| (3, 0.86) ||| 3 ||| 3 ||| N-glycan analysis of human alpha1-antitrypsin produced in Chinese hamster ovary cells. Human alpha-1-antitrypsin (alpha1AT) is a glycoprotein with protease inhibitor activity protecting tissues from degradation. Patients with inherited alpha1AT deficiency are treated with native alpha1AT (nAT) purified from human plasma. In the present study, recombinant alpha1AT (rAT) was produced in Chinese hamster ovary (CHO) cells and their glycosylation patterns, inhibitory activity and in vivo half-life were compared with those of nAT. A peptide mapping analysis employing a deglycosylation reaction confirmed full occupancy of all three glycosylation sites and the equivalency of rAT and nAT in terms of the protein level. N-glycan profiles revealed that rAT contained 10 glycan structures ranging from bi-antennary to tetra-antennary complex-type glycans while nAT displayed six peaks comprising majorly bi-antennary glycans and a small portion of tri-antennary glycans. In addition, most of the rAT glycans were shown to have only core alpha(1 - 6)-fucose without terminal fucosylation, whereas only minor portions of the nAT glycans contained core or Lewis X-type fucose. As expected, all sialylated glycans of rAT were found to have alpha(2 - 3)-linked sialic acids, which was in sharp contrast to those of nAT, which had mostly alpha(2 - 6)-linked sialic acids. However, the degree of sialylation of rAT was comparable to that of nAT, which was also supported by an isoelectric focusing gel analysis. Despite the differences in the glycosylation patterns, both alpha1ATs showed nearly equivalent inhibitory activity in enzyme assays and serum half-lives in a pharmacokinetic experiment. These results suggest that rAT produced in CHO cells would be a good alternative to nAT derived from human plasma.

431 ||| 9322435 ||| (3, 0.85) ||| 3 ||| 3 ||| Structural characterization of site-specific N-glycosylation of recombinant human factor VIII by reversed-phase high-performance liquid chromatography-electrospray ionization mass spectrometry. The present study addresses the site occupancy and the site-specific carbohydrate microheterogeneity of N-linked oligosaccharides in recombinant human factor VIII, expressed in Chinese hamster ovary cells. The four factor VIIIa polypeptides, formed upon incubation with human thrombin, were isolated and separately subjected to proteolysis with trypsin. These tryptic digests were analyzed by reversed-phase high-performance liquid chromatography/electrospray ionization mass spectrometry. Selected ion monitoring of diagnostic carbohydrate ions was utilized to identify glycopeptide-containing chromatographic peaks. Oligomannose and complex carbohydrates were detected at the glycosylation sites of the 50 and the 73 kDa polypeptides, while all the oligosaccharides identified on the B-domain were complex-type structures. Only the 43 kDa polypeptide was found nonglycosylated. These studies established a biantennary core-fucosylated carbohydrate as the major substituent, consistent with the conclusions of the analyses on the entire N-linked carbohydrate pool (Kumar, H. P. M.; Hague, C.; Haley, T.; Starr, C. M.; Besman, M. J.; Lundblad, R.; Baker, D. Biotechnol. Appl. Biochem. 1996, 24, 207-216.). In addition, this mass spectrometric investigation revealed the presence of a complex nonfucosylated oligosaccharide not reported previously for this glycoprotein.

432 ||| 8530451 ||| (3, 0.85) ||| 3 ||| 3 ||| LEC18, a dominant Chinese hamster ovary glycosylation mutant synthesizes N-linked carbohydrates with a novel core structure. The dominant Chinese hamster ovary cell glycosylation mutant, LEC18, was selected for resistance to pea lectin (Pisum sativum agglutinin (PSA)). Lectin binding studies show that LEC18 cells express altered cell surface carbohydrates with markedly reduced binding to 125I-PSA and increased binding to 125I-labeled Datura stramonium agglutinin (DSA) compared with parental cells. Desialylated [3H]Glc-labeled LEC18 cellular glycopeptides that did not bind to concanavalin A-Sepharose exhibited an increased proportion of species that were bound to DSA-agarose. Most of these glycopeptides bound to ricin-agarose and were unique to LEC18 cells. This fraction was purified from approximately 10(10) cells and shown by 1H NMR spectroscopy and methylation linkage analysis to contain novel N-linked structures. Digestion of these glycopeptides with mixtures of beta-D-galactosidases and N-acetyl-beta-D-glucosaminidases gave core glycopeptides that, in contrast to cores from parental cells, were mainly not bound to concanavalin A-Sepharose or to PSA-agarose. 1H NMR spectroscopy, matrix-assisted laser desorption ionization/time of flight mass spectrometry, electrospray mass spectrometry, and collision-activated dissociation mass spectrometry showed that the LEC18 core glycopeptides contained a new GlcNAc residue that substitutes the core GlcNAc residues. Methylation linkage analysis of the parent compound provided evidence that the GlcNAc is linked at O-6 to give the following novel, N-linked core structure. [formula: see text]

433 ||| 7737204 ||| (3, 0.85) ||| 3 ||| 3 ||| Structural analysis of the sialylated N- and O-linked carbohydrate chains of recombinant human erythropoietin expressed in Chinese hamster ovary cells. Sialylation patterns and branch location of dimeric N-acetyllactosamine units. The N-linked carbohydrate chains of recombinant human erythropoietin expressed in CHO cells were quantitatively released with peptide-N4-(N-acetyl-beta-glucosaminyl)asparagine amidase F, separated from the remaining O-glycoprotein by gel-permeation chromatography, and subsequently fractionated via FPLC on Mono Q, HPLC on Lichrosorb-NH2 and high-pH anion-exchange chromatography on CarboPac PA1. The purified sialylated oligosaccharides were analyzed by one-dimensional and two-dimensional 500-MHz 1H-NMR spectroscopy. When necessary, oligosaccharides were treated with endo-beta-galactosidase (and N-acetyl-beta-glucosaminidase) followed by 1H-NMR analysis of the incubation products, to obtain additional structural information. Di-, tri-, tri'- and tetraantennary N-acetyllactosamine-type oligosaccharides occur which can be completely (major) or partially (minor) sialylated. Three different types of alpha 2-3-linked sialic acids are present, namely, N-acetylneuraminic acid (95%), N-glycolylneuraminic acid (2%) and N-acetyl-9-O-acetylneuraminic acid (3%). In the case of partial sialylation, a non-random distribution of the sialic acids over the branches is observed. One or two extra N-acetyllactosamine units, being exclusively located in the branches attached to the alpha 1-6-linked Man residue, can be present in completely or partially sialylated di-, tri'-, and tetraantennary oligosaccharides. Tetraantennary oligosaccharides with N-acetyllactosamine repeats could be digested quantitatively with endo-beta-galactosidase from Bacteroides fragilis, whereas under the same conditions tri' antennary oligosaccharides hardly reacted (< 15%). Using endo-beta-galactosidase from Escherichia freundii, these tri'antennary oligosaccharides could be digested more extensively (> 75%). The O-linked carbohydrate chains were released from the O-glycoprotein by alkaline borohydride treatment, and purified via FPLC on Mono Q and HPLC on Lichrosorb-NH2. Two O-glycans were found, namely, Neu5Ac alpha 2-3Gal beta 1-3GalNAc-ol and Neu5Ac alpha 2-3Gal beta 1-3(Neu5Ac alpha 2-6)GalNAc-ol.

434 ||| 23125152 ||| (3, 0.84) ||| 3 ||| 3 ||| Affinity chromatographic purification of human immunoglobulin a from Chinese hamster ovary cell culture supernatant. Hexamer peptide ligand HWRGWV, initially screened from a solid phase combinatorial peptide library for immunoglobulins G (IgG) purification, is shown to also have potential for immunoglobulin A (IgA) purification. The determined dissociation constants for hIgA on HWRGWV resins at three different peptide densities from 0.11 to 0.55 meq/g fall in the range of 10(-6) -10(-7) M, which are somewhat lower than those for hIgG. Although relatively low dynamic binding capacity (DBC) in the range of 9.2-16.8 mg IgA/mL resin at linear flow rates from 173 to 35 cm/h were obtained for IgA compared to IgG, the DBC value of HWRGWV for IgA is much greater than current commercially available affinity ligands. Although relatively lower binding affinity to secretory IgA compared to monomeric IgA was observed, the peptide ligand resins exhibit great potential for large-scale purification of both human IgA and secretory IgA. Recoveries of 96.0% and 94.3%, and purities of 90.3% and 91.7% were achieved for human IgA and secretory IgA purification, respectively, from spiked Chinese hamster ovary cell culture supernatants without an extra afterwash step. Over 95% in purities were achieved for IgA and secretory IgA with an extra afterwash step; however, the recoveries would decrease at least 15% and 40% for IgA and secretory IgA, respectively.

435 ||| 9518460 ||| (3, 0.82) ||| 3 ||| 3 ||| Human keratinocyte growth factor recombinantly expressed in Chinese hamster ovary cells: isolation of isoforms and characterization of post-translational modifications. Keratinocyte growth factor (KGF) is a member of the fibroblast growth factor family that acts specifically on epithelial cells in a paracrine mode. We employed a mammalian expression system to synthesize recombinant human KGF and isolated two preparations, KGF-a and KGF-b, from medium conditioned by Chinese hamster ovary cells. On an SDS-PAGE gel, KGF-a migrates as two bands near 25-29 kDa and contains both N- and O-linked sugar moieties attached near the N-terminus. Detailed structural characterization confirms that KGF-a contains a single amino acid sequence predicted from cDNA sequence and the molecule has two intramolecular disulfide bridges, Cys1-Cys15 and Cys102-Cys106. An additional Cys at position 40 is free and resides in a solvent-inaccessible environment. Mass spectrometric analyses of KGF-a peptides verify the occurrence of several post-translational modifications in the molecule, including partial oxidation at Met28, partial sulfation at Tyr27, and glycosylation at Asn14 and Thr22. The Asn-linked carbohydrate structures are heterogeneous, which include biantennary, triantennary, and tetraantennary structures with none or up to four sialic acids attached to various structures, while the Thr-linked carbohydrates contain typical mucin-type structures. KGF-b is an N-terminally truncated form of KGF-a posttranslationally processed at Arg23 and is not glycosylated. Both KGF-a and KGF-b forms are capable of stimulating DNA synthesis in quiescent Balb/MK mouse epidermal keratinocytes.

436 ||| 11788797 ||| (3, 0.82) ||| 3 ||| 3 ||| Engineering neoglycoproteins with multiple O-glycans using repetitive pentapeptide glycosylation units. Controlled protein remodeling with O-linked glycans has been limited by our incomplete understanding of the process of glycosylation. Here we describe a secretable fibroblast growth factor (FGF) with multiple mucin-type O-glycans produced by introducing a minimum pentapeptide glycosylation unit in a decarepeat format at its N- or C-terminus. Expressed in Chinese hamster ovary cells, chemical and biochemical analyses of the resultant proteins (Nm10-FGF and Cm10-FGF, respectively) demonstrated that all O-glycosylation units were glycosylated and the dominant structure was sialylated Gal[beta1-3]GalNAc. This indicates that minimum O-glycosylation unit in multirepeat format serves as a remarkably efficient acceptor in CHO cells. The Nm10-FGF and Cm10-FGF proteins maintained the mitogenic activity to vascular endothelial cells. In addition, intact Cm10-FGF and its desialylated form interacted with several lectins in the same way as mucin-type glycoproteins. The intact Cm10-FGF with multiple sialylated O-glycans exhibited a longer lifetime in circulating blood, whereas the Cm10-FGF with desialylated O-glycans exhibited a shorter lifetime than the deglycosylated form of Cm10-FGF. Our approach would thus appear to be highly effective for engineering neoglycoproteins, the characteristics of which are determined by their multiple mucin-type O-glycans.

437 ||| 10561463 ||| (3, 0.82) ||| 3 ||| 3 ||| The glycan processing and site occupancy of recombinant Thy-1 is markedly affected by the presence of a glycosylphosphatidylinositol anchor. Thy-1 is a cell surface glycoprotein containing three N-linked glycosylation sites and a glycosylphosphatidylinositol (GPI) anchor. The effect of the anchor on its N-linked glyco-sylation was investigated by comparing the glycosylation of soluble recombinant Thy-1 (sThy-1) with that of recombinant GPI anchored Thy-1, both expressed in Chinese hamster ovary cells. The sThy-1 was produced in a variety of isoforms including some which lacked carbohydrate on all three sequons whereas the GPI anchored form appeared fully glycosylated like native Thy-1. This was surprising as the attachment of N-linked sugars occurs cotranslationally and it was not expected that the presence of a C-terminal GPI anchor signal sequence would affect sequon occupancy. Furthermore sThy-1 lacking glycosylation could be produced with the inhibitor tunicamycin but in contrast cell surface expression of unglycosylated GPI anchored Thy-1 could not be obtained. The GPI anchored form appeared less processed with almost 4-fold more oligo-mannose oligosaccharides than in sThy-1 and also with less sialylated and core fucosylated biantennary glycans. Possible mechanisms whereby the anchor or the anchor signal sequence, control site occupancy and maturation are discussed.

438 ||| 18239438 ||| (3, 0.81) ||| 3 ||| 3 ||| Overproduction of recombinant human VEGF (vascular endothelial growth factor) in Chinese hamster ovary cells. Vascular endothelial growth factors (VEGFs) are a family of proteins that mediate angiogenesis. VEGF165 is a VEGF-A isoform and has been extensively studied owing to its potential use in therapeutic angiogenesis. This study established Chinese hamster ovary (CHO) cells overexpressing recombinant human VEGF165 (rhVEGF165) protein. The production rate of the established CHO cells was over 80 mg/l of rhVEGF165 protein from a 7-day batch culture process using a 7.5-l bioreactor with a 5-l working volume and serum-free medium. The rhVEGF165 protein was purified to homogeneity from the culture supernatant using a two-step chromatographic procedure that resulted in a 48% recovery rate. The purified rhVEGF165 protein was a glycosylated homodimeric protein with a higher molecular weight (MW) than the protein expressed from insect cells, suggesting that the glycosylation of the rhVEGF165 protein in CHO cells differed from that in insect cells. The purified rhVEGF165 protein in this study was functionally active with a half-maximal effective concentration of 3.8 ng/ ml and specific activity of 2.5 x 10^5^ U/mg.

439 ||| 16737827 ||| (3, 0.81) ||| 3 ||| 3 ||| Purification of recombinant activin A using the second follistatin domain of follistatin-related gene (FLRG). Activins are multifunctional growth factors belonging to the transforming growth factor-beta superfamily. Isolation of activins from natural sources requires many steps and only produces limited quantities. Even though recombinant preparations have been used in recent studies, purification of recombinant activins still requires multiple steps. To purify recombinant activin A, we have developed a simple method using the second follistatin domain of an activin-binding protein follistatin-related gene (FLRG). An affinity column was prepared with a partial FLRG fusion protein. The partial FLRG protein contained the second follistatin domain and the C-terminus acidic domain, and was tagged with six histidine residues at its N-terminus. The fusion protein was expressed in Escherichia coli and purified with nickel affinity column. Thereafter, the purified fusion protein was coupled to NHS-activated column. Recombinant activin A was produced in Chinese hamster ovary (CHO) cells, which were stably transfected with rat inhibin/activin betaA-subunit cDNA. After 48-h suspension culture of the cells in a serum free medium, the culture media was recovered and passed through the FLRG-coupled column. After washing with phosphate-buffered saline, bound protein was eluted out with an acidic buffer. Any significant contaminations were not detected when the purified protein was analyzed by SDS-PAGE. Apparent sizes of the protein were 14 and 28 kDa under the reduced and non-reduced conditions, respectively. Western blot analysis confirmed that the purified protein was activin A. The purified recombinant activin stimulated p3TP-lux reporter activity in CHO cells and follicle-stimulating hormone secretion from rat pituitary cells.

440 ||| 11278492 ||| (3, 0.81) ||| 3 ||| 3 ||| Characterization of the glycosylation profiles of Alzheimer's beta -secretase protein Asp-2 expressed in a variety of cell lines. Amyloid 39-42 beta -peptides are the main components of amyloid plaques found in the brain of Alzheimer's disease patients. Amyloid 39-42 beta-peptide is formed from amyloid precursor protein by the sequential action of beta- and gamma-secretases. Asp-2 is a transmembrane aspartic protease expressed in the brain, shown to have beta-secretase activity. Mature Asp-2 has four N-glycosylation sites. In this report we have characterized the carbohydrate structures in this glycoprotein expressed in three different cell lines, namely Chinese hamster ovary, CV-1 origin of SV40, and baculovirus-infected SF9 cells. Biantennary and triantennary oligosaccharides of the "complex" type were released from glycoprotein expressed in the mammalian cells, whereas mannose-rich glycans were identified from glycoprotein synthesized in the baculovirus-infected cells. Site-directed mutagenesis of the asparagine residues at amino acid positions 153, 172, 223, and 354 demonstrate that the protease activity of Asp-2 is dependent on its glycosylation.

441 ||| 10745209 ||| (3, 0.81) ||| 3 ||| 3 ||| Modification of a recombinant GPI-anchored metalloproteinase for secretion alters the protein glycosylation. The N-linked glycans of recombinant leishmanolysin (GP63) expressed as a glycosylphosphatidylinositol (GPI)-anchored membrane protein or modified for secretion in Chinese hamster ovary (CHO) cells were analyzed by fast atom bombardment-mass spectrometry (FAB-MS). The glycans isolated from both membrane and secreted protein were predominantly complex biantennary structures. However other aspects of the glycan profiles showed striking differences. The degree of sialylation of the membrane form was greatly reduced and the core fucosylation of biantennary structures was increased compared to the secreted form. Glycans isolated from membrane expressed protein also contained a higher proportion of lactosamine repeats. Residence times in the secretory pathway were similar for both secreted and membrane protein. Glycosylation differences may therefore be due to differences in protein conformation and accessibility to glycosyltransferases or glycosidases. These differences in glycosylation represent an important factor when considering modifying membrane expressed proteins for secreted production.

442 ||| 7627805 ||| (3, 0.80) ||| 3 ||| 3 ||| Interferon-gamma receptor extracellular domain-IgG fusion protein produced in Chinese hamster ovary cells as mixture of glycoforms. Glycosylation of proteins fulfills important functions and because of its diversity contributes to apparent protein heterogeneity. We investigated the heterogeneity of a fusion protein comprising the extracellular domain of the human interferon-gamma (IFN-gamma) receptor and parts of the human IgG3 constant region, a potential IFN-gamma antagonist. The protein was produced in Chinese hamster ovary (CHO) cells and was secreted into the culture medium as an 175 kD glycoprotein. Glycosylation represented approximately one-third of the apparent molecular mass of the fusion protein, consisted of N- and O-linked carbohydrate moieties, and included sialic acid residues as part of both N- and O-linked oligosaccharides. Fusion protein forms with different apparent molecular masses and charges were separated by ion-exchange chromatography. Preparative electrofocusing revealed a wide spectrum of glycoforms. Glycosylation of the fusion protein and of soluble IFN-gamma receptors, comprising the extracellular domain of the native sequence, expressed in insect and CHO cells did not interfere with affinity of ligand binding.

443 ||| 23891370 ||| (3, 0.80) ||| 3 ||| 3 ||| Novel purification method of human immunoglobulin by using a thermo-responsive protein A. We attempted to evaluate a novel purification method of immunoglobulins (IgGs) by using a mutant type of protein A. Although this mutant protein A binds to IgGs at 5 C, the IgGs are released at 40 C; hence, it was designated as thermo-responsive protein A (TRPA). We aimed to purify IgG1 from the culture supernatant of CHO cells producing AE6F4 human monoclonal IgG1. AE6F4 IgG1 was purified using only a TRPA-filled column and by modifying the temperature, without any exposure to acidic conditions. Furthermore, the purified AE6F4 IgG1 maintained the inherent binding affinity to antigen, while this property was lost in AE6F4 IgG1 purified using a conventional protein A (CPA) column possibly because of product aggregation and fragmentation. These data suggest that IgG is sensitive to acid treatment; however, it can be highly purified with retention of high affinity by using a TRPA column. Further, this purification method can be used on an industrial scale for the purification of antibody drugs.

444 ||| 10833268 ||| (3, 0.80) ||| 3 ||| 3 ||| Comparative study of the asparagine-linked sugar chains of human lipocalin-type prostaglandin D synthase purified from urine and amniotic fluid, and recombinantly expressed in Chinese hamster ovary cells. Lipocalin-type prostaglandin D synthase (L-PGDS) is a highly glycosylated member of the lipocalin gene family and is secreted into various human body fluids. We comparatively analyzed the structures of asparagine-linked sugar chains of human L-PGDS produced by recombinant Chinese hamster ovary cells and naturally occurring human urine and amniotic fluid. After the sugar chains were liberated by hydrazinolysis followed by N-acetylation, they were derivatized with 2-aminobenzamide. All of the sugar chains of three L-PGDSs occur as biantennary complex-type sugar chains. Most of the sugar chains of three samples were fucosylated on the inner most N-acetylglucosamine residue. Although the sugar chains of the recombinant L-PGDS do not contain any bisecting N-acetylglucosamine residues, 58% and 34% of the fucosylated-sugar chains of amniotic fluid and urine L-PGDSs, respectively, contain bisecting N-acetylglucosamine residues. The sialic acid residues occur solely as Siaalpha2-->3Gal groups of the recombinant L-PGDS; the sialic acid residues of other L-PGDS occur as both Siaalpha2-->3Gal and Siaalpha2-->6Gal groups. Variations in L-PGDS glycosylation may prove useful as markers to further elucidate the role of L-PGDS glycoforms in different tissues.

445 ||| 9593742 ||| (3, 0.79) ||| 3 ||| 3 ||| Glycosylation profile of a recombinant urokinase-type plasminogen activator receptor expressed in Chinese hamster ovary cells. Association of urokinase-type plasminogen activator (uPA) to cells via binding to its specific cellular receptor (uPAR) augments the potential of these cells to support plasminogen activation, a process that has been implicated in the degradation of extracellular matrix proteins during cell migration and tissue remodeling. The uPA receptor is a glycolipid-anchored membrane protein belonging to the Ly-6/uPAR superfamily and is the only multidomain member identified so far. We have now purified the three individual domains of a recombinant soluble uPAR variant, expressed in Chinese hamster ovary cells, after limited proteolysis using chymotrypsin and pepsin. The glycosylation patterns of these domains have been determined by matrix assisted laser desorption ionization and electrospray ionization mass spectrometry. Of the five potential attachment sites for asparagine-linked carbohydrate in uPAR only four are utilized, as the tryptic peptide derived from domain III containing Asn233 was quantitatively recovered without carbohydrate. The remaining four attachment sites were shown to exhibit site-specific microheterogeneity of the asparagine-linked carbohydrate. The glycosylation on Asn52 (domain I) and Asn172 (domain II) is dominated by the smaller biantennary complex-type oligosaccharides, while Asn162 (domain II) and Asn200 (domain III) predominantly carry tri- and tetraantennary complex-type oligosaccharides. The carbohydrate moiety on Asn52 in uPAR domain I could be selectively removed by N-glycanase treatment under nondenaturing conditions. This susceptibility was abrogated when uPAR participitated in a bimolecular complex with pro-uPA or smaller receptor binding derivatives thereof, demonstrating the proximity of the ligand-binding site to this particular carbohydrate moiety. uPAR preparations devoid of carbohydrate on domain I exhibited altered binding kinetics toward uPA (a 4-6-fold increase in Kd) as assessed by real time biomolecular interaction analysis.

446 ||| 21573946 ||| (3, 0.79) ||| 3 ||| 3 ||| N-Glycosylation profiling of recombinant mouse extracellular superoxide dismutase produced in Chinese hamster ovary cells. Extracellular superoxide dismutase (EC-SOD), the major SOD isoenzyme in biological fluids, is known to be N-glycosylated and heterogeneous as was detected in most glycoproteins. However, only one N-glycan structure has been reported in recombinant human EC-SOD produced in Chinese hamster ovary (CHO) cells. Thus, a precise N-glycan profile of the recombinant EC-SOD is not available. In this study, we report profiling of the N-glycan in the recombinant mouse EC-SOD produced in CHO cells using high-resolution techniques, including the liberation of N-glycans by treatment with PNGase F, fluorescence labeling by pyridylamination, characterization by anion-exchange, normal and reversed phase-HPLC separation, and mass spectrometry. We succeeded in identifying 26 different types of N-glycans in the recombinant enzyme. The EC-SOD N-glycans were basically core-fucosylated (98.3% of the total N-glycan content), and were high mannose sugar chain, and mono-, bi-, tri-, and tetra-antennary complex sugar chains exhibiting varying degrees of sialylation. Four of the identified N-glycans were uniquely modified with a sulfate group, a Lewis(x) structure, or an alpha-Gal epitope. The findings will shed new light on the structure-function relationships of EC-SOD N-glycans.

447 ||| 11846800 ||| (3, 0.79) ||| 3 ||| 3 ||| Tyrosine sulfation and N-glycosylation of human heparin cofactor II from plasma and recombinant Chinese hamster ovary cells and their effects on heparin binding. The structure of post-translational modifications of human heparin cofactor II isolated from human serum and from recombinant Chinese hamster ovary cells and their effects on heparin binding have been characterized. Oligosaccharide chains were found attached to all three potential N-glycosylation sites in both protein preparations. The carbohydrate structures of heparin cofactor II circulating in blood are complex-type diantennary and triantennary chains in a ratio of 6 : 1 with the galactose being > 90% sialylated with alpha 2-->6 linked N-acetylneuraminic acid. About 50% of the triantennary structures contain one sLe(x) motif. Proximal alpha 1-->6 fucosylation of oligosacharides from Chinese hamster ovary cell-derived HCII was detected in > 90% of the diantennary and triantennary glycans, the latter being slightly less sialylated with exclusively alpha 2-->3-linked N-acetylneuraminic acid units. Applying the ESI-MS/ MS-MS technique, we demonstrate that the tryptic peptides comprising tyrosine residues in positions 60 and 73 were almost completely sulfated irrespective of the protein's origin. Treatment of transfected Chinese hamster ovary cells with chlorate or tunicamycin resulted in the production of heparin cofactor II molecules that eluted with higher ionic strength from heparin-Sepharose, indicating that tyrosine sulfation and N-linked glycans may affect the inhibitor's interaction with glycosaminoglycans.

448 ||| 10705443 ||| (3, 0.78) ||| 3 ||| 3 ||| Generation of recombinant human large latent transforming growth factor-beta 1 and monoclonal antibodies to it. Transforming growth factor beta 1 (TGF-beta 1) is a regulator of cell growth and differentiation. It is produced in various of cells and tissues as a biologically latent complex, whose significance is still unknown. We established a Chinese hamster ovary cells that produced recombinant human large latent TGF-beta 1. The growth factor was purified from serum-free conditioned medium of the cell line was purified to apparent homogeneity by four steps of column chromatography. The purified protein gave a single band with the apparent molecular weight of 210,000 on SDS-PAGE, and had four subunits, of 12.5, 40, 53, and 150-190 kDa. These components were identical to TGF-beta 1, the N-terminal remnant of pro-TGF-beta 1, pro-TGF-beta 1, and latent TGF-beta 1 binding protein, respectively. The purified growth factor had biological activity similar to that of the growth factor purified from human platelets. We prepared four monoclonal antibodies by immunization of mice with the recombinant protein. In western blotting, two of the antibodies bound to latent TGF-beta 1 binding protein. The two other antibodies reacted with the N-terminal remnant of pro-TGF-beta 1. Recombinant large latent TGF-beta 1 and its monoclonal antibodies could be used for detailed structural and functional studies of the large latent TGF-beta 1 complex.

449 ||| 8631761 ||| (3, 0.77) ||| 3 ||| 3 ||| O-linked L-fucose is present in Desmodus rotundus salivary plasminogen activator. DSPAalpha1 (Desmodus rotundus salivary plasminogen activator), a plasminogen activator from the saliva of the vampire bat Desmodus rotundus, is an effective thrombolytic agent. An unusual type of posttranslational modification, in which L-fucose is O-glycosidically linked to threonine 61 in the epidermal growth factor domain was found for natural DSPAalpha1 and its recombinant form isolated from Chinese hamster ovary cells. In the present study a combination of carbohydrate and amino acid composition analysis, amino acid sequencing, and mass spectrometry revealed that the L-fucose is bound to residues 56-68 of DSPAalpha1. The amino acid sequence of this glycosylation site agreed with the suggested consensus sequence Cys-Xaa-Xaa-Gly-Gly-Ser/Thr-Cys described for other proteins. Anew strategy for the identification of the modified amino acid was established. Direct evidence for the occurrence of fucosyl-threonine was obtained by mass spectrometry after digestion of the glycopeptide with a mixture of peptidases. On the basis of these results, DSPAalpha1 is a suitable model for studying the influence of O-fucosylation on clearance rates, particularly in comparative studies with the identically fucosylated and structurally related tissue plasminogen activator.

450 ||| 10207177 ||| (3, 0.77) ||| 3 ||| 3 ||| Oligosaccharide analysis and molecular modeling of soluble forms of glycoproteins belonging to the Ly-6, scavenger receptor, and immunoglobulin superfamilies expressed in Chinese hamster ovary cells. Most cell surface molecules are glycoproteins consisting of linear arrays of globular domains containing stretches of amino acid sequence with similarities to regions in other proteins. These conserved regions form the basis for the classification of proteins into superfamilies. Recombinant soluble forms of six leukocyte antigens belonging to the Ly-6 (CD59), scavenger receptor (CD5), and immunoglobulin (CD2, CD48, CD4, and Thy-1) superfamilies were expressed in the same Chinese hamster ovary cell line, thus providing an opportunity to examine the extent to which N-linked oligosaccharide processing might vary in a superfamily-, domain-, or protein-dependent manner in a given cell. While we found no evidence for superfamily-specific modifications of the glycans, marked differences were seen in the types of oligosaccharides attached to individual proteins within a given superfamily. The relative importance of local protein surface properties versus the overall tertiary structure of the molecules in directing this protein-specific variation was examined in the context of molecular models. These were constructed using the 3D structures of the proteins, glycan data from this study, and an oligosaccharide structural database. The results indicated that both the overall organization of the domains and the local protein structure can have a large bearing on site-specific glycan modification of cells in stasis. This level of control ensures that the surface of a single cell will display a diverse repertoire of glycans and precludes the presentation of multiple copies of a single oligosaccharide on the cell surface. The glycans invariably shield large regions of the protein surfaces although, for the glycoproteins examined here, these did not hinder the known active sites of the molecules. The models also indicated that sugars are likely to play a role in the packing of the native cell surface glycoproteins and to limit nonspecific protein-protein interactions. In addition, glycans located close to the cell membrane are likely to affect crucially the orientation of the glycoproteins to which they are attached.

451 ||| 9499380 ||| (3, 0.76) ||| 3 ||| 3 ||| Human alpha-galactosidase A: characterization of the N-linked oligosaccharides on the intracellular and secreted glycoforms overexpressed by Chinese hamster ovary cells. Human alpha-galactosidase A (alpha-Gal A) is the lysosomal glycohydrolase that cleaves the terminal alpha-galactosyl moieties of various glycoconjugates. Overexpression of the enzyme in Chinese hamster ovary (CHO) cells results in high intracellular enzyme accumulation and the selective secretion of active enzyme. Structural analysis of the N -linked oligosaccharides of the intracellular and secreted glycoforms revealed that the secreted enzyme's oligosaccharides were remarkably heterogeneous, having high mannose (63%), complex (30%), and hybrid (5%) structures. The major high mannose oligosaccharides were Man5-7GlcNAc2 species. Approximately 40% of the high mannose and 30% of the hybrid oligosaccharides had phosphate monoester groups. The complex oligosaccharides were mono-, bi-, 2,4-tri-, 2,6-tri- and tetraantennary with or without core-region fucose, many of which had incomplete outer chains. Approximately 30% of the complex oligosaccharides were mono- or disialylated. Sialic acids were mostly N -acetylneuraminic acid and occurred exclusively in alpha2, 3-linkage. In contrast, the intracellular enzyme had only small amounts of complex chains (7.7%) and had predominantly high mannose oligosaccharides (92%), mostly Man5GlcNAc2 and smaller species, of which only 3% were phosphorylated. The complex oligosaccharides were fucosylated and had the same antennary structures as the secreted enzyme. Although most had mature outer chains, none were sialylated. Thus, the overexpression of human alpha-Gal A in CHO cells resulted in different oligosaccharide structures on the secreted and intracellular glycoforms, the highly heterogeneous secreted forms presumably due to the high level expression and impaired glycosylation in the trans- Golgi network, and the predominately Man5-7GlcNAc2 cellular glycoforms resulting from carbohydrate trimming in the lysosome.

452 ||| 7547035 ||| (3, 0.76) ||| 3 ||| 3 ||| Stable expression in Chinese hamster ovary cells of a homogeneous recombinant active fragment of human platelet glycoprotein Ib alpha. A fragment (residues His1-Val289) of the alpha chain of human platelet glycoprotein Ib containing the von Willebrand factor and thrombin binding sites has been expressed in Chinese hamster ovary cells. The secreted soluble recombinant protein had an apparent molecular mass of 42 kD and reacted with a conformation-dependent monoclonal antibody that only binds to native GP Ib, thus demonstrating its proper folding. The rather broad band obtained after immobilization of the recombinant fragment on nitrocellulose could be resolved into a very sharp band of molecular weight of about 35 kD by growing the cells in the presence of tunicamycin, an inhibitor of N-linked glycosylation. The recombinant GP Ib alpha fragments (with or without glycosylation) were purified by immunoaffinity chromatography. Both truncated forms bound vWF in the presence of botrocetin with comparable affinity as a proteolytic 42 kD fragment of purified human platelet GP Ib-IX. They were also retained on thrombin-Sepharose. We then selected a cell clone (B1) that produced over at least three months about 1.5 micrograms of recombinant protein per million cells per day. Using this clone a large-scale production finally yielded milligram amounts of the functionally active recombinant human GP Ib alpha fragment.

453 ||| 24239937 ||| (3, 0.76) ||| 3 ||| 3 ||| Cation exchange versus multimodal cation exchange resins for antibody capture from CHO supernatants: identification of contaminating host cell proteins by mass spectrometry. We compared classical and multimodal cation exchange resins for the capture of recombinant antibodies from Chinese hamster ovary (CHO) cell culture supernatant. Both Capto S and Capto MMC resins present anionic groups while the multimodal Capto MMC also features a hydrophobic moiety. First we screened optimal binding and elution conditions in microplates with a pure antibody. We validated the results on the lab-scale with columns with a pure antibody and a CHO cell culture supernatant. Both resins achieved good yield and purity for the capture step of an antibody. However, the multimodal resin appeared more efficient and selective. Then we identified proteins in the antibody fraction by mass spectrometry in order to highlight the behavior of host cell proteins (HCPs).

454 ||| 9228088 ||| (3, 0.75) ||| 3 ||| 3 ||| The O-linked fucose glycosylation pathway. Evidence for protein-specific elongation of o-linked fucose in Chinese hamster ovary cells. O-Linked fucose is an unusual form of glycosylation recently shown to modify the hydroxyls of serine or threonine residues at a strict consensus site within epidermal growth factor-like domains of several serum proteins. Here we demonstrate that Chinese hamster ovary cells modify numerous proteins with O-linked fucose and that the fucose is elongated on specific proteins. We have identified at least two forms of O-linked fucose elongation in Chinese hamster ovary cells: a disaccharide (Glcbeta1,3Fuc) and a larger oligosaccharide of indeterminate structure. Interestingly, it appears that the level of monosaccharide accumulates in the cells over time whereas the disaccharide does not. Analysis of the O-linked fucose-containing saccharides on individual proteins revealed that some proteins are modified with the monosaccharide only, whereas others are modified with monosaccharide and disaccharide, or monosaccharide and oligosaccharide. These results suggest that elongation of the O-linked fucose monosaccharide is a protein-specific phenomena. The presence of elongated O-linked fucose moieties suggests that a novel glycosylation pathway exists in mammalian cells with O-linked fucose as the core.

455 ||| 20332087 ||| (3, 0.75) ||| 3 ||| 3 ||| Glycosylation pattern of mature dimeric leukocyte and recombinant monomeric myeloperoxidase: glycosylation is required for optimal enzymatic activity. The involvement of myeloperoxidase (MPO) in various inflammatory conditions has been the scope of many recent studies. Besides its well studied catalytic activity, the role of its overall structure and glycosylation pattern in biological function is barely known. Here, the N-glycan composition of native dimeric human MPO purified from neutrophils and of monomeric MPO recombinantly expressed in Chinese hamster ovary cells has been investigated. Analyses showed the presence of five N-glycans at positions 323, 355, 391, 483, 729 in both proteins. Site by site analysis demonstrated a well conserved micro- and macro-heterogeneity and more complex-type N-glycans for the recombinant form. Comparison of biological functionality of glycosylated and deglycosylated recombinant MPO suggests that glycosylation is required for optimal enzymatic activity. Data are discussed with regard to biosynthesis and the three-dimensional structure of MPO.

456 ||| 9634799 ||| (3, 0.74) ||| 3 ||| 3 ||| N-glycosylation of recombinant human interferon-gamma produced in different animal expression systems. Recombinant human interferon-gamma (IFN-gamma) was expressed in Chinese hamster ovary cells, baculovirus-infected Sf9 insect cells and the mammary gland of transgenic mice. The N-linked carbohydrate populations associated with both Asn25 and Asn97 glycosylation sites were characterized by matrix-assisted laser desorption/ionization-mass spectrometry (MALDI-MS) in combination with exoglycosidase array sequencing. A site-specific analysis of dual (2N) and single (1N) site-occupancy variants of IFN-gamma derived from Chinese hamster ovary cells showed that N-glycans were predominantly of the complex bi- and triantennary type. Although Asn25-linked glycans were substituted with a core fucose residue, Asn97 N-glycans were predominantly non-fucosylated, and truncated complex and high-mannose oligosaccharide chains were also evident. Transgenic mouse derived IFN-gamma exhibited considerable site-specific variation in N-glycan structures. Asn25-linked carbohydrates were of the complex, core fucosylated type, Asn97-linked carbohydrates were mainly of the oligomannose type, with smaller proportions of hybrid and complex N-glycans. Carbohydrates associated with both glycosylation sites of IFN-gamma from Sf9 insect cells were mainly tri-mannosyl core structures, with fucosylation confined to the Asn25 site. These data demonstrate the profound influence of host cell type and protein structure on the N-glycosylation of recombinant proteins.

457 ||| 7876170 ||| (3, 0.74) ||| 3 ||| 3 ||| Purification and characterization of heparan sulfate 6-sulfotransferase from the culture medium of Chinese hamster ovary cells. Heparan sulfate 6-sulfotransferase, which catalyzes the transfer of sulfate from 3'-phosphoadenylyl sulfate to position 6 of N-sulfoglucosamine in heparan sulfate, was purified 10,700-fold to apparent homogeneity with a 40% yield from the serum-free culture medium of Chinese hamster ovary cells. The isolation procedure included affinity chromatography of the first heparin-Sepharose CL-6B column (stepwise elution), 3',5'-ADP-agarose, and the second heparin-Sepharose CL-6B column (gradient elution). Sodium dodecyl sulfate-polyacrylamide gel electrophoresis of the purified enzyme showed two protein bands with molecular masses of 52 and 45 kDa. Both proteins appeared to be glycoproteins, because their molecular masses decreased after N-glycanase digestion. When completely desulfated and N-resulfated heparin was used as acceptor, the purified enzyme transferred sulfate to position 6 of N-sulfoglucosamine residue but did not transfer sulfate to the amino group of glucosamine residue or to position 2 of the iduronic acid residue. Heparan sulfate was also sulfated by the purified enzyme at position 6 of N-sulfoglucosamine residue. Chondroitin and chondroitin sulfate did not serve as acceptors. The optimal pH for enzyme activity was around 6.3. The enzyme activity was inhibited by dithiothreitol and was stimulated strongly by protamine. The Km value for adenosine 3'-phosphate 5'-phosphosulfate was 0.44 microM.

458 ||| 8725116 ||| (3, 0.73) ||| 3 ||| 3 ||| Purification, characterization and immunogenicity of recombinant varicella-zoster virus glycoprotein gE secreted by Chinese hamster ovary cells. The gene of the varicella-zoster virus (VZV) glycoprotein gE, engineered to code for a truncated molecule lacking the anchor and carboxy-terminal tail domains, was transfected into Chinese hamster ovary (CHO) cells via the pEE14 mammalian expression vector. One recombinant cell line, CHO-gE-2-9, secreted high levels of truncated gE into the culture medium. The product was purified to near homogeneity by a combination of anion exchange, hydrophobic and metal-chelate chromatographies. Purified recombinant gE showed the expected amino-terminal sequence and its glycosylation pattern proved similar to that of the natural product. When injected into mice, using either Freund's or alum as adjuvant, the native truncated gE induced complement-dependent neutralizing antibodies. In contrast, when the molecule was first denatured, it lost immunogenicity with alum. These data show that the recombinant gE, although truncated, could potentially be included in a subunit vaccine against VZV infection.

459 ||| 10622399 ||| (3, 0.73) ||| 3 ||| 3 ||| Influence of glycosylation on the clearance of recombinant human sex hormone-binding globulin from rabbit blood. Human sex hormone-binding globulin (hSHBG) is a plasma glycoprotein that binds sex steroids with high affinity. Variations in hSHBG glycosylation contribute to its electrophoretic microheterogeneity, but the functional significance of different SHBG glycoforms is unknown. Carbohydrates may influence the biological activities and half-lives of glycoproteins and we have examined how oligosaccharides at specific sites influence the plasma clearance of hSHBG in vivo. To accomplish this, fully-glycosylated hSHBG, or hSHBG mutants lacking specific oligosaccharides chains, were expressed in Chinese hamster ovary (CHO) cells and purified by immunoaffinity chromatography. The purified recombinant proteins were then biotinylated to study their plasma half-lives after intravenous injection into rabbits. When compared to hSHBG isolated from serum, recombinant hSHBG migrates with a slightly larger average molecular size during denaturing polyacrylamide gel electrophoresis. This is due to a greater proportion (33-39% vs. 3%) of more highly branched N-linked oligosaccharides on the recombinant proteins. When injected into rabbits, the disappearance of recombinant hSHBG showed two exponential components, as previously shown for natural hSHBG in the same animal model. The mean +/- S.E.M. plasma half-lives of recombinant hSHBG (t 1/2alpha 0.11+/-0.03 h and t 1/2beta 18.94+/-1.65 h) are shorter than previously measured for natural hSHBG (t 1/2alpha 3.43+/-0.72 h and t 1/2beta 38.18+/-7.22 h) and this is likely due to differences in the composition of their N-linked oligosaccharides. An O-linked chain at Thr7 does not influence the plasma clearance of hSHBG in the presence or absence of N-linked carbohydrates at Asn351 and Asn367. However, a 1.5-1.6 fold (p<0.03) increase in plasma half-life of variants lacking both N-glycosylation sites was observed and this is probably due to the fact these variants are not recognized by the asialoglycoprotein receptor-mediated clearance system. Removal of either N-glycosylation consensus site also increased (p<0.0001) the plasma half-life of hSHBG by 2.3 2.4 fold. Thus, the metabolic clearance of hSHBG appears to be determined by the number of N-linked oligosaccharides rather than their location.

460 ||| 9278435 ||| (3, 0.72) ||| 3 ||| 3 ||| Carbohydrate structures of recombinant human alpha-L-iduronidase secreted by Chinese hamster ovary cells. alpha-L-Iduronidase is a lysosomal hydrolase that is deficient in Hurler syndrome and clinically milder variants. Recombinant human alpha-L-iduronidase, isolated from secretions of an overexpressing Chinese hamster ovary cell line, is potentially useful for replacement therapy of these disorders. Because of the importance of carbohydrate residues for endocytosis and lysosomal targeting, we examined the oligosaccharides of recombinant alpha-L-iduronidase at each of its six N-glycosylation sites. Biosynthetic radiolabeling showed that three or four of the six oligosaccharides of the secreted enzyme were cleaved by endo-beta-N-acetylglucosaminidase H, with phosphate present on the sensitive oligosaccharides and L-fucose on the resistant ones. For structural analysis, tryptic and chymotryptic glycopeptides were isolated by lectin binding and reverse phase high pressure liquid chromatography; their molecular mass was determined by matrix-assisted laser desorption-time of flight mass spectrometry before and after treatment with endo- or exoglycosidases or with alkaline phosphatase. Identification of the peptides was assisted by amino- or carboxyl-terminal sequence analysis. The major oligosaccharide structures found at each site were as follows: Asn-110, complex; Asn-190, complex; Asn-336, bisphosphorylated (P2Man7GlcNAc2); Asn-372, high mannose (mainly Man9GlcNAc2, some of which was monoglucosylated); Asn-415, mixed high mannose and complex; Asn-451, bisphosphorylated (P2Man7GlcNAc2). The Asn-451 glycopeptide was unexpectedly resistant to digestion by N-glycanase unless first dephosphorylated, but it was sensitive to endo-beta-N-acetylglucosaminidase H and to glycopeptidase A. The heterogeneity of carbohydrate structures must represent the accessibility of the glycosylation sites as well as the processing capability of the overexpressing Chinese hamster ovary cells.

461 ||| 23201283 ||| (3, 0.72) ||| 3 ||| 3 ||| Overproduction of recombinant human mannose-binding lectin (MBL) in Chinese hamster ovary cells. Mannose-binding lectin (MBL) is an important serum protein that functions in the innate immune system and has been considered to have therapeutic potential in MBL replacement therapies for patients with deficient or low levels of MBL. In this study, we established a Chinese hamster ovary (CHO) cell line that overexpresses the recombinant human MBL (rhMBL) protein. In an 11-day batch culture process using a 30-L bioreactor (20-L working volume) and serum-free medium, these cells could produce over 226 mg/L of rhMBL protein. The recombinant protein was then purified to homogeneity from the culture supernatant using a three-step chromatographic procedure that resulted in a recovery rate of approximately 55%. This purified rhMBL protein adopted oligomeric bouquet-like structures that were similar to those of native MBL present in human blood, and these oligomeric structures were reported to be critical in MBL functions. We further demonstrated in carbohydrate binding and complementation activation assays that this rhMBL protein was functionally active with very similar dissociation constants and half maximal effective concentrations to those of native MBL.

462 ||| 11056384 ||| (3, 0.72) ||| 3 ||| 3 ||| Stabilization of human recombinant erythropoietin through interactions with the highly branched N-glycans. Human erythropoietin (EPO) produced in Chinese hamster ovary cells (CHO-EPO) is a hydrophobic protein stabilized by the highly branched complex-type N-glycans. To characterize the stabilizing effect of the N-glycans, the properties of enzymatically N-glycan-modified CHO-EPO species were compared spectrophotometrically. CD and fluorescence spectra following the protein unfolding induced by guanidine hydrochloride or pH revealed that the inner regions including the galactose residues of the N-glycans stabilize the protein conformation. The decrease in the conformational stability caused by enzymatic trimming of the N-glycans was associated with the exposure of the hydrophobic protein surface areas accessible to 1-anilino-8-naphthalenesulfonic acid (ANS) binding. Further, the ANS binding and heat denaturation of Escherichia coli-expressed EPO (nonglycosylated EPO) were depressed in dilute solutions (1 mM or so) of free N-glycans of the complex type. These results, together with the finding that the N-glycans of CHO-EPO make little contact with the aromatic amino acid residues exposed on the protein surface, indicate that the inner regions including the galactose residues of the intramolecular N-glycans stabilize the protein conformation by clinging to the hydrophobic protein surface areas mainly made up of nonaromatic hydrocarbon groups.

463 ||| 11003557 ||| (3, 0.72) ||| 3 ||| 3 ||| Aglycone structure influences alpha-fucosyltransferase III activity using N-acetyllactosamine glycoside acceptors. We showed previously that Chinese hamster ovary cells took up and utilized a variety of N-acetylglucosaminides as primers of oligosaccharide biosynthesis (Ding et al., 1999, J. Carbohydr. Chem., 18:471-475). In this study, a library of N-acetylglucosaminides was enzymatically galactosylated in vitro to yield type 2 chain N-acetyllactosaminides bearing a variety of aglycones. Those disaccharides are potential acceptors for fucosyltransferases. As an extension of the previous study, we tested the type 2 chain disaccharyl glycosides (Galbeta1,4-GlcNAcbeta-R) for their aglycone-dependent acceptor specificity for alpha-L-fucosyltransferase III (Fuc-TIII). The enzyme activity significantly depended on the aglycone structures, suggesting that, in addition to the polar groups on the sugar moiety, the hydrophobic aglycone can substantially contribute to recognition in this reaction.

464 ||| 10328551 ||| (3, 0.72) ||| 3 ||| 3 ||| Establishment of a method for mapping of N-linked oligosaccharides and its use to analyze industrially produced recombinant erythropoietin. A rapid and convenient method for N-linked oligosaccharide structure analysis was developed and applied to the quality examination of commercially manufactured recombinant human erythropoietin (rEPO). Oligosaccharides released from rEPO expressed in Chinese hamster ovary cells were labeled with 2-aminobenzamide, and analyzed by a combination of diethylaminoethyl (DEAE) column HPLC and matrix-assisted laser desorption ionization time-of-flight mass spectrometry (MALDI-TOF-MS). This newly developed method allowed us to analyze sialylated oligosaccharides directly. The results showed that these oligosaccharides are composed of neutral-mono-di-tri-tetrasialyl oligosaccharides in a ratio of <1:2:13:34:51%, and that the core structure was a mixture of complex type bi-, tri- and tetra-antennary forms with one to three polylactosaminyl chains. Further analysis showed clearly that the N-linked oligosaccharide structure of rEPO has not changed throughout the past ten years of manufacturing by our company. The analysis was carried out using a small quantity (1 nmol) of rEPO, demonstrating the efficacy of the newly developed method. Further, the N-linked oligosaccharides of rEPO manufactured by our company almost coincided with those of the erythropoietin European Pharmacopoeia Biological Reference Preparation (Eur.Ph.BRP), a standard product.

465 ||| 17662991 ||| (3, 0.71) ||| 3 ||| 3 ||| Practical reversed-phase high-performance liquid chromatography method for laboratory-scale purification of recombinant human thyrotropin. A small, semi-preparative C(4) RP-HPLC column was used to set up the conclusive laboratory-scale purification of Chinese hamster ovary-derived human thyrotropin (hTSH), after a preliminary concentration-purification of an extremely dilute and poorly ( approximately 0.6 microg hTSH/mL; mass fraction=0.35%) conditioned medium on a cation exchanger. Several fractions of this eluate were repeatedly injected on the semi-preparative column, obtaining, in a single run (<1h chromatographic time), a concentrated pool ( approximately 1.2 mg/mL) of highly purified hTSH that could be further concentrated to >3 mg/mL and then efficiently lyophilized. The overall recovery in the rapid RP-HPLC purification step, including concentration and lyophilization, was of the order of 80%. The final product, when tested via a precise, single-dose in vivo bioassay, confirmed that it did not suffer any loss of bioactivity. This same methodology can be easily adapted to the small-scale purification of other recombinant products, even when obtained from genetically modified organisms at extremely low concentrations and mass fractions.

466 ||| 11171073 ||| (3, 0.71) ||| 3 ||| 3 ||| Purification of phosphatidylglycerophosphate synthase from Chinese hamster ovary cells. Phosphatidylglycerophosphate (PGP) synthase catalyses the committed step in the biosynthesis of phosphatidylglycerol and cardiolipin in mammalian cells. Recently we isolated a Chinese hamster ovary (CHO) PGS1 cDNA encoding PGP synthase. In the present study we purified this PGP synthase to near-homogeneity from the mitochondrial fraction of CHO-K1 cells; the final enzyme preparation gave a single 60 kDa protein on SDS/PAGE. Polyclonal antibodies raised against a recombinant CHO PGS1 protein cross-reacted with the purified 60 kDa protein and with CHO membrane proteins of 60 kDa and 62 kDa that increased after transfection with the PGS1 cDNA. The 60 and 62 kDa protein levels in a PGP synthase-defective mutant of CHO-K1 cells were markedly lower than those in CHO-K1 cells. These results indicated that the purified 60 kDa protein was PGP synthase encoded by the PGS1 gene. In addition we found that the purified PGP synthase had no PGP phosphatase activity, indicating that phosphatidylglycerol was produced from CDP-diacylglycerol through two steps catalysed by distinct enzymes, PGP synthase and PGP phosphatase.

467 ||| 11003555 ||| (3, 0.71) ||| 3 ||| 3 ||| Asn-linked sugar chain structures of recombinant human thrombopoietin produced in Chinese hamster ovary cells. Human thrombopoietin (TPO) that regulates the numbers of megakaryocytes and platelets is a heavily N- and O-glycosylated glycoprotein hormone with partial homology to human erythropoietin (EPO). We prepared recombinant human TPO produced in Chinese hamster ovary (CHO) cells and analyzed the sugar chain structures quantitatively using 2-aminobenzamide labeling, sequential glycosidase digestion and matrix-assisted laser desorption ionization time-of-flight mass spectrometry (MALDI-TOF/MS). We found bi-, tri- and tetraantennary complex-type sugar chains with one or two N-acetyllactosamine repeats, which are common to recombinant human EPO produced in CHO cells. On the other hand, there were triantennary sugar chains with one or two N-acetyllactosamine repeats that were specific to the recombinant human TPO, and their distributions of branch structures were also different. These results suggested that proximal protein structure should determine the branch structure of Asn-linked sugar chains in addition to the glycosyltransferases subset.

468 ||| 9184837 ||| (3, 0.70) ||| 3 ||| 3 ||| Stereoselectivity of the Chinese hamster ovary cell sialidase: sialoside hydrolysis with overall retention of configuration. The stereochemical course of enzymatic hydrolysis by the soluble sialidase from Chinese hamster ovary cells, expressed as a recombinant protein in insect Sf9 cells, was determined using proton nuclear magnetic resonance spectroscopy. 4-Methyl umbelliferyl-N-acetyl neuraminic acid was employed as substrate, and the stereoselectivity of the enzyme catalysis was ascertained by monitoring the H3 axial and equatorial protons of the sialic acid product over the reaction course. At both high (3 U) and low concentrations (1 U) of the enzyme, the alpha anomer of the sialic acid was clearly observed as the initial reaction product. The corresponding beta anomer of sialic acid appeared much later in the reaction, arising from mutarotation of the alpha anomer. Similar studies were also carried out using the Salmonella typhimurium LT 2 sialidase, a protein of similar size and substrate specificity. Both enzymes apparently cleave the alpha linked sialoside substrate with retention of configuration. Based on the observations of a wide variety of other glycohydrolytic enzymes that have shown a strong correlation of the stereoselectivity of catalysis with active site topology (Gebler et al., J. Biol. Chem. 267, 12559-12561, 1992), the results obtained here suggest that the microbial and mammalian sialidases have a homologous active site architecture even though the molecules do not share significant primary sequence similarities.

469 ||| 19951948 ||| (3, 0.70) ||| 3 ||| 3 ||| Glycomics profiling of Chinese hamster ovary cell glycosylation mutants reveals N-glycans of a novel size and complexity. Identifying biological roles for mammalian glycans and the pathways by which they are synthesized has been greatly facilitated by investigations of glycosylation mutants of cultured cell lines and model organisms. Chinese hamster ovary (CHO) glycosylation mutants isolated on the basis of their lectin resistance have been particularly useful for glycosylation engineering of recombinant glycoproteins. To further enhance the application of these mutants, and to obtain insights into the effects of altering one specific glycosyltransferase or glycosylation activity on the overall expression of cellular glycans, an analysis of the N-glycans and major O-glycans of a panel of CHO mutants was performed using glycomic analyses anchored by matrix-assisted laser desorption ionization-time of flight/time of flight mass spectrometry. We report here the complement of the major N-glycans and O-glycans present in nine distinct CHO glycosylation mutants. Parent CHO cells grown in monolayer versus suspension culture had similar profiles of N- and O-GalNAc glycans, although the profiles of glycosylation mutants Lec1, Lec2, Lec3.2.8.1, Lec4, LEC10, LEC11, LEC12, Lec13, and LEC30 were consistent with available genetic and biochemical data. However, the complexity of the range of N-glycans observed was unexpected. Several of the complex N-glycan profiles contained structures of m/z approximately 13,000 representing complex N-glycans with a total of 26 N-acetyllactosamine (Gal beta1-4GlcNAc)(n) units. Importantly, the LEC11, LEC12, and LEC30 CHO mutants exhibited unique complements of fucosylated complex N-glycans terminating in Lewis(x) and sialyl-Lewis(x) determinants. This analysis reveals the larger-than-expected complexity of N-glycans in CHO cell mutants that may be used in a broad variety of functional glycomics studies and for making recombinant glycoproteins.

470 ||| 11156219 ||| (3, 0.70) ||| 3 ||| 3 ||| Phase I clinical trial of the chimeric monoclonal antibody (c30.6) in patients with metastatic colorectal cancer. The murine antibody 30.6 recognizes an antigen that is expressed on a high proportion of colorectal carcinomas and their metastases. We report the results of single-dose escalation studies of the chimeric 30.6 (c30.6) monoclonal antibody in metastatic colorectal cancer, to evaluate its safety, pharmacokinetics, and biodistribution. Recombinant c30.6 (IgG1kappa) antibody was secreted from Chinese hamster ovary cells and purified by a multistep chromatography process. Seventeen patients with metastatic colorectal cancer were enrolled in this dose escalation study. The first four patients were treated with 3 mg of 123I-labeled c30.6, whereas the next 13 received a single dose of unlabeled antibody (maximum dose, 50 mg/m2). The most frequent side effect was a novel syndrome of severe burning and erythema of the face, chest, neck, ears, palms, soles, and genitalia. The frequency of this syndrome was markedly reduced in those patients premedicated with high doses of histamine receptor 1 and histamine receptor 2 blockers. Other side effects were mild and predictable. Biodistribution studies showed a rapid and intensive hepatic uptake. At the 50 mg/m2 level the half-life and maximum serum concentration were 81 +/- 15 h and 7.9 microg/ml, respectively. One patient developed a low-level human anti-c30.6 response. Tumor response was assessed by computed tomography, positron emission tomography scanning, and serial carcinoembryonic antigen measurements. There were no partial responses, although positron emission tomography scanning demonstrated some reduction in tumor activity in three individuals. The chimerized c30.6 antibody is not immunogenic in humans and appears worthy of further study. It does, however, produce a unique profile of side effects that can be well controlled with premedication.

471 ||| 9989653 ||| (3, 0.69) ||| 3 ||| 3 ||| Structural characterization of recombinant human erythropoietins by fluorophore-assisted carbohydrate electrophoresis. Routine analysis of the carbohydrate moieties of a glycoprotein is critical for ensuring the consistent quality of biopharmaceutical products. Fluorophore-assisted carbohydrate electrophoresis (FACE) is a recently introduced method for the separation, by polyacrylamide gel electrophoresis, of oligosaccharides labeled with 8-amino-naphthalene-1,3,6-trisulphonic acid (ANTS). In this study, we have evaluated the applicability of the FACE method to analysis of the carbohydrate moieties of three different recombinant human erythropoietins (rHuEPOs), two Chinese hamster ovary (CHO) cell-derived rHuEPOs, and one baby hamster kidney (BHK) cell-derived rHuEPO. The N-linked oligosaccharides released from the rHuEPOs were labeled with ANTS. Enzymic sequence analysis of the N-linked oligosaccharides was performed by the FACE method. The results showed that the FACE method was useful for the analysis of sialo-, asialo-, and agalacto-oligosaccharides in the same gel. Its usefulness for rapidly and reliably revealing the oligosaccharide profiles of given glycoproteins, and its reproducibility were also confirmed in this study. In conclusion, the method can be used for evaluating the quality consistency of recombinant glycoprotein products in terms of the carbohydrate moiety.

472 ||| 8991512 ||| (3, 0.69) ||| 3 ||| 3 ||| Human melanoma and Chinese hamster ovary cells galactosylate n-alkyl-beta-glucosides using UDP gal:GlcNAc beta 1,4 galactosyltransferase. We previously showed that human melanoma, CHO and other cells can convert beta-xylosides into structural analogs of ganglioside GM3. We have investigated several potential acceptors including a series of n-alkyl-beta-D-glucosides (n = 6-9). All were labeled with 3H-galactose when incubated with human melanoma cells. Octyl-beta-D-glucoside (Glc beta Octyl) was the best acceptor, whereas neither octyl-alpha-D-glucoside nor N-octanoyl-methylglucamine (MEGA 8) were labeled. Analysis of the products by a combination of chromatographic methods and specific enzyme digestions showed that the acceptors first received a single Gal beta 1,4 residue followed by an alpha 2,3 linked sialic acid. Synthesis of these products did not affect cell viability, adherence, protein biosynthesis, or incorporation of radiolabeled precursors into glycoprotein, glycolipid or proteoglycans. To determine which beta 1,4 galactosyl transferase synthesized Gal beta 1,4Glc beta Octyl, we analyzed similar incubations using CHO cells and a mutant CHO line (CHO 761) which lacks GAG-core specific beta 1,4 galactosyltransferase. The mutant cells showed the same level of incorporation as the control, eliminating this enzyme as a candidate. Thermal inactivation kinetics using melanoma cell microsomes and rat liver Golgi to galactosylate Glc beta Octyl showed the same half-life as UDP-Gal:GlcNAc beta 1,4 galactosyltransferase, whereas LacCer synthase was inactivated at a much faster rate. We show that Glc beta Octyl is a substrate for purified bovine milk UDP-Gal:GlcNAc beta 1,4 galactosyltransferase. Furthermore, the galactosylation of Glc beta Octyl by CHO cell microsomes can be competitively inhibited by GlcNAc or GlcNAc beta MU. These results indicate that UDP-Gal:GlcNAc beta 1,4 galactosyltransferase is the enzyme used for the synthesis of the alkyl lactosides when cells or rat liver Golgi are incubated with alkyl beta glucosides.

473 ||| 18501915 ||| (3, 0.69) ||| 3 ||| 3 ||| Purification of human immunoglobulin G by thermoseparating aqueous two-phase systems. The purification of human immunoglobulin G (IgG) from a Chinese hamster ovary (CHO) cells supernatant was studied using an aqueous two-phase system (ATPS) composed of ethylene oxide/propylene oxide (UCON) and dextran. In UCON/dextran systems IgG partitions preferentially to the less hydrophobic dextran-rich phase (Kp<1). The addition of triethylene glycol-diglutaric acid (TEG-COOH) shifted the IgG partition into the upper phase showing significant improvements in both the recovery yields and purity. The purification of IgG from a CHO cell supernatant with UCON 2000/dextran/TEG-COOH system was optimised using a central composite design. Using an ATPS composed of 8% UCON, 6% dextran and 20% TEG-COOH, IgG was purified, in two steps, with a global yield of 85% and 88% purity. Statistical valid models were obtained to predict the effect of the experimental conditions on the IgG yield and purity, for both extraction and back-extraction steps. A system composed of 10% UCON, 5.5% dextran and 20% TEG-COOH was identified as the best compromise between final purity and yield.

474 ||| 10985765 ||| (3, 0.69) ||| 3 ||| 3 ||| Mass spectrometric characterization of the glycosylation pattern of HIV-gp120 expressed in CHO cells. An analytical approach is reported for the characterization of the specific glycans found on highly glycosylated proteins based on a combination of specific proteolysis and deglycosylation combined with two different mass spectrometric approaches, matrix-assisted laser desorption/ionization mass spectrometry, and nanoelectrospray mass spectrometry/tandem mass spectrometry using a hybrid quadrupole-time-of-flight tandem mass spectrometer. The high resolution and mass accuracy of the mass spectrometric data obtained on the hybrid instrument combined with the high parent mass capabilities are shown to be extremely useful in the site-specific assignment of heterogeneous glycans. Using this methodology, 25 of 26 consensus glycosylation sites on HIV-1(SF2) gp120, expressed in Chinese hamster ovary cells, could be assigned. Good correlations between the relative abundances of members of heterogeneous series in the matrix-assisted laser desorption/ionization mass spectra and the nanoelectrospray mass spectra were observed, indicating that the mass spectrometric data reflected the actual abundances of the members of the series. These data were incorporated with molecular modeling based on the solved structure of a mutant truncated, highly deglycosylated gp120 to propose a structural model for the completely glycosylated form.

475 ||| 18995863 ||| (3, 0.68) ||| 3 ||| 3 ||| Integrated process for the purification of antibodies combining aqueous two-phase extraction, hydrophobic interaction chromatography and size-exclusion chromatography. We have evaluated a process incorporating aqueous two-phase extraction, hydrophobic interaction chromatography (HIC) and size-exclusion chromatography (SEC) for the purification of human immunoglobulin G (IgG) from a Chinese hamster ovary (CHO) cell supernatant. These unit operations were chosen not only for allowing the removal of target impurities but also for facilitating the integration of different process units without the need for any conditioning step. Extraction in aqueous two-phase systems (ATPSs), composed of polyethylene glycol (PEG) and sodium citrate, allowed the concentration of the antibodies in the citrate-rich phase and the removal of the most hydrophobic compounds in the PEG-rich phase. An ATPS composed of 10% (w/w) PEG 3350 and 12% (w/w) citrate, at pH 6, allowed the recovery of IgG with a 97% yield, 41% HPLC purity and 72% protein purity. This bottom phase was then directly loaded on a phenyl-Sepharose HIC column. This intermediate purification step allowed the capture of the antibodies using a citrate mobile phase with 99% of the antibody recovered in the elution fractions, with 86% HPLC purity and 91% protein purity. Finally, SEC allowed the final polishing by removing IgG aggregates. HIC-eluted fractions were directly injected in a Superose 6 size-exclusion column affording a 100% pure IgG solution with 90% yield.

476 ||| 17010983 ||| (3, 0.68) ||| 3 ||| 3 ||| Comparison of ceramic hydroxy- and fluoroapatite versus Protein A/G-based resins in the isolation of a recombinant human antibody from cell culture supernatant. A recombinant human antibody (IgG(1)-subtype) was produced in Chinese Hamster Ovary (CHO) cells. Alternatives to the established isolation by Protein A affinity chromatography were investigated. Neither an alternative elution agent (Arginine) nor an alternative affinity ligand (Protein G) resulted in an improvement in yield and/or purity. Subsequently, apatite stationary phases including a novel ceramic fluoroapatite material were tested. By applying a double gradient (first 0 to 1M NaCl, then 0.01 to 0.4M phosphate) the culture supernatant was separated into three fractions: the flow through, which contained no active antibody, the NaCl-eluate, which contained the antibody and no other discernible protein contaminants, and a fraction that eluted in the phosphate gradient and contained several proteins, but no active antibody. In case of the hydroxyapatite, retention of the antibody decreased and yield increased when the pH was raised from 6.0 to 8.2 (isoelectric point (pI) of the antibody: 8.3), to reach a yield of 71% at pH of 8.2. In case of the fluoroapatite, retention was also found to increase with increasing mobile phase pH, but the yields went through a maximum (of ca. 90%) at a mobile phase pH of 7.0. No traces of contaminants were seen in the corresponding gel. This is the first time that yields of 90% and such high purities have been reported as the result of a single chromatographic step for the antibody in question with either (Protein A) affinity or apatite chromatography.

477 ||| 9450955 ||| (3, 0.67) ||| 3 ||| 3 ||| Protein C-mannosylation is enzyme-catalysed and uses dolichyl-phosphate-mannose as a precursor. C-mannosylation of Trp-7 in human ribonuclease 2 (RNase 2) is a novel kind of protein glycosylation that differs fundamentally from N- and O-glycosylation in the protein-sugar linkage. Previously, we established that the specificity determinant of the acceptor substrate (RNase 2) consists of the sequence -x-x-W, where the first Trp becomes C-mannosylated. Here we investigated the reaction with respect to the mannosyl donor and the involvement of a glycosyltransferase. C-mannosylation of Trp-7 was reduced 10-fold in CHO (Chinese hamster ovary) Lec15 cells, which are deficient in dolichyl-phosphate-mannose (Dol-P-Man) synthase activity, compared with wild-type cells. This was not a result of a decrease in C-mannosyltransferase activity. Rat liver microsomes were used to C-mannosylate the N-terminal dodecapeptide from RNase 2 in vitro, with Dol-P-Man as the donor. This microsomal transferase activity was destroyed by heat and protease treatment, and displayed the same acceptor substrate specificity as the in vivo reaction studied previously. The C-C linkage between the indole and the mannosyl moiety was demonstrated by tandem electrospray mass spectrometry analysis of the product. GDP-Man, in the presence of Dol-P, functioned as a precursor in vitro with membranes from wild-type but not CHO Lec15 cells. In contrast, with Dol-P-Man both membrane preparations were equally active. It is concluded that a microsomal transferase catalyses C-mannosylation of Trp-7, and that the minimal biosynthetic pathway can be defined as: Man -> -> GDP-Man -> Dol-P-Man -> (C2-Man-)Trp.

478 ||| 8809043 ||| (3, 0.67) ||| 3 ||| 3 ||| Comparison of the mRNA sequences for Pi class glutathione transferases in different hamster species and the corresponding enzyme activities with anti-benzo[a]pyrene-7,8-dihydrodiol 9,10-epoxide. Glutathione S-transferase (GST) of class Pi (GST Pi) is known to detoxify the mutagenic and carcinogenic (+)-anti-benzo[a]pyrene-7, 8-dihydrodiol 9,10-epoxide [(+)-anti-BPDE] by conjugation with glutathione. Previously, we have shown that Chinese hamster V79 cells contain GST Pi, but seem to lack the capacity to conjugate (+)-anti-BPDE, although these cells do conjugate other substrates with GSH [Romert, Dock, Jenssen and Jernstrom (1989) Carcinogenesis 10, 1701-1707; Swedmark, Romert, Morgenstern and Jenssen (1992) Carcinogenesis 13, 1719-1723; Swedmark and Jenssen (1994) Gene 139, 251-256]. In the present study we have compared four cell lines derived from different hamster species with respect to GST cDNA sequences and capacity to conjugate (+)-or(-)-anti-BPDE. The cell lines were V79 and Chinese hamster ovary cells (CHO), Armenian hamster lung (AHL) cells and baby hamster kidney (BHK) cells. The sequencing revealed a complete homology between the V79 and CHO cDNA for GST Pi, whereas the corresponding amino acid sequences predicted from the corresponding AHL and BHK cDNAs differed by six and nine amino acids, respectively, from the predicted V79 sequence. None of these changes alone was found to influence the xenobiotic substrate-binding site. The cytosolic fractions from BHK and AHL cells were found to catalyse conjugation of (+)-anti-BPDE with GSH, whereas the corresponding activity in CHO cells was non-detectable. As shown previously, V79 cells were devoid of activity towards (+)-anti-BPDE. All the cell lines studied demonstrated appreciable GST activity towards 1-chloro-2,4-dinitrobenzene, but no activity with (-)-anti-BPDE. The latter result suggests that GST Pi is the sole or predominant GST in these cell lines. This was confirmed by HPLC analysis of purified enzymes obtained by affinity chromatography. However, when the catalytic activities of the pure enzymes were determined, all four different GST Pi enzymes were found to be highly capable of conjugating (+)-anti-BPDE with GSH. This observation indicates the existence of an intracellular factor that selectively inhibits conjugation of (+)-anti-BPDE, but not of 1-chloro-2,4-dinitrobenzene in the V79 and CHO cell lines. This new phenomenon seems to be specific for Chinese hamster, since both these cell lines originate from this species.

479 ||| 7741215 ||| (3, 0.67) ||| 3 ||| 3 ||| Microheterogeneity of erythropoietin carbohydrate structure. The microheterogeneity of the carbohydrate structures on recombinant human erythropoietin (rHuEPO) expressed in Chinese hamster ovary cells has been evaluated by electrospray ionization (ESI) mass spectrometry (MS) of glycopeptide fragments. The microheterogeneity is largely associated with the presence or absence of terminal N-acetylneuraminic acid (Neu5Ac) residues, varying amounts of O-acetylation of the Neu5Ac residues, and the presence or absence of N-acetyllactosamine extensions. The N-linked carbohydrate structures were structurally diverse; 52 different N-linked oligosaccharide structures were identified. Consistent structural assignments could be made from data obtained using different proteolytic digests, ESI solvent systems (aqueous/methanol systems with acetic or formic acid), and on-line or off-line LC/MS analysis. All glycosylation sites exhibited some level of O-acetylation of Neu5Ac residues. Interestingly, glycosylation site asparagine-83 exhibits mono-O-acetyl and di-O-acetyl Neu5Ac residues, while the other sites, asparagine-24, asparagine-38, and serine-126, exhibit mainly mono-O-acetyl Neu5Ac derivatization. This difference in O-acetylation may be site specific or due to sample handling of labile structures. However, mild base treatment of rHuEPO with NaOH on ice removed the O-acetyl groups associated with a given carbohydrate structure, without adversely affecting the underlying oligosaccharide structure, resulting in a simplified mass spectra. Nuclear magnetic resonance spectroscopy of Neu5Ac residues released by neuraminidase treatment of total rHuEPO indicated that Neu5,9Ac2 residues were present. Additional resonances were also observed that were consistent with other Neu5Ac O-acetyl linkages; these O-acetyl resonances could be removed by mild base hydrolysis of rHuEPO.

480 ||| 25872915 ||| (3, 0.67) ||| 3 ||| 3 ||| Antibody glycosylation and its impact on the pharmacokinetics and pharmacodynamics of monoclonal antibodies and Fc-fusion proteins. Understanding the impact of glycosylation and keeping a close control on glycosylation of product candidates are required for both novel and biosimilar monoclonal antibodies (mAbs) and Fc-fusion protein development to ensure proper safety and efficacy profiles. Most therapeutic mAbs are of IgG class and contain a glycosylation site in the Fc region at amino acid position 297 and, in some cases, in the Fab region. For Fc-fusion proteins, glycosylation also frequently occurs in the fusion partners. Depending on the expression host, glycosylation patterns in mAb or Fc-fusions can be significantly different, thus significantly impacting the pharmacokinetics (PK) and pharmacodynamics (PD) of mAbs. Glycans that have a major impact on PK and PD of mAb or Fc-fusion proteins include mannose, sialic acids, fucose (Fuc), and galactose (Gal). Mannosylated glycans can impact the PK of the molecule, leading to reduced exposure and potentially lower efficacy. The level of sialic acid, N-acetylneuraminic acid (NANA), can also have a significant impact on the PK of Fc-fusion molecules. Core Fuc in the glycan structure reduces IgG antibody binding to IgG Fc receptor IIIa relative to IgG lacking Fuc, resulting in decreased antibody-dependent cell-mediated cytotoxicity (ADCC) activities. Glycoengineered Chinese hamster ovary (CHO) expression systems can produce afucosylated mAbs that have increased ADCC activities. Terminal Gal in a mAb is important in the complement-dependent cytotoxicity (CDC) in that lower levels of Gal reduce CDC activity. Glycans can also have impacts on the safety of mAb. mAbs produced in murine myeloma cells such as NS0 and SP2/0 contain glycans such as Galalpha1-3Galbeta1-4N-acetylglucosamine-R and N-glycolylneuraminic acid (NGNA) that are not naturally present in humans and can be immunogenic when used as therapeutics.

481 ||| 25722186 ||| (3, 0.67) ||| 3 ||| 3 ||| O-glycan repertoires on a mucin-type reporter protein expressed in CHO cell pools transiently transfected with O-glycan core enzyme cDNAs. Glyco-engineering of host cells is used to increase efficacy, decrease immunogenicity and increase circulatory half-lives of protein biopharmaceuticals. The effect of transiently expressed O-glycan core chain glycosyltransferases on O-glycan biosynthesis pathways in CHO cells is reported. Liquid chromatography-mass spectrometry and Western blotting were used to map the O-glycome of a mucin-type fusion protein transiently co-transfected with beta1,3-N-acetylglucosaminyltransferase 3 (extended C1 beta3GnT3), core 2 beta1,6-N-acetylglucosaminyltransferase I (C2 beta3GnT1) or core 3 beta1,3-N-acetylglucosaminyltransferase 6 (C3 beta3GnT6) in CHO cells. Extended core 1 (GlcNAcbeta1,3Galbeta1,3GalNAc) and core 3 (GlcNAcbeta1,3GalNAc), and increased expression of core 2 [Galbeta1,3(GlcNAcbeta1,6)GalNAc], O-glycans were generated on P-selectin glycoprotein ligand-1/mouse IgG2b (PSGL1/mIgG2b). Endogenous poly-N-acetyllactosamine (poly-LacNAc) synthase elongated extended core 1 and core 3 generating O-glycans with up to five LacNAc repeats. Low amounts of core 3 O-glycans appeared upon extended C1 beta3GnT3 expression. The alpha2,6-sialylated type 2 chain was detected upon co-transfection with the beta-galactoside alpha2,6-sialyltransferase I. N-acetylglucosamine-6-O-sulfotransferase 2 transferred sulfate to carbon 6 of GlcNAc in poly-LacNAc sequences. CHO cells with its known O-glycan repertoire can be used to express recombinant mucin-type proteins together with selected glycosyltransferases in order to recreate carbohydrate determinants on defined O-glycan chains. They will become important tools for assessing the core chain-dependent binding activity of carbohydrate-binding proteins.

482 ||| 8728318 ||| (3, 0.66) ||| 3 ||| 3 ||| Glycosylation structure and enzyme activity of lecithin:cholesterol acyltransferase from human plasma, HepG2 cells, and baculoviral and Chinese hamster ovary cell expression systems. The glycosylation state of lecithin:cholesterol acyltransferase (LCAT) may be important in determining its enzymatic activity. We compared glycosylation structure, enzyme kinetics, and phosphatidylcholine (PC) acyl specificity of human LCAT from four sources: human plasma (pLCAT), media from HepG2 cells (HepG2 LCAT), media from SF21 cells infected with a recombinant baculovirus (bLCAT) and media from stably transfected Chinese hamster ovary (CHO) cells (CHO LCAT). bLCAT was underglycosylated (molecular weight approximately 50 kDa) and resistant to digestion by N-glycanase F, endoglycosidase F, and neuraminidase. CHO and HepG2 LCAT were overglycosylated (approximately 68 kDa and approximately 70-75 kDa) compared to pLCAT (approximately 65 kDa). CHO LCAT, like pLCAT, was sensitive to N-glycanase F and neuraminidase but not to endoglycosidase F. HepG2 LCAT demonstrated resistance to N-glycanase F and endoglycosidase F. Apparent Km values for all four enzymes were similar (1.4-9.2 microM cholesterol) for recombinant high density lipoproteins (rHDL) containing sn-1 16:0, sn-2 18:1 PC (POPC). Apparent Vmax values (nmol cholesteryl ester formed/h per micrograms) were 52.6 for pLCAT, 48.6 for CHO LCAT, 15.3 for bLCAT, and 8.3 for HepG2 LCAT. Changes in PC acyl specificity in the presence and absence of cholesterol were characterized by comparing the ratio of LCAT activity on rHDL containing sn-1 16:0, sn-2 20:4 PC (PAPC) or POPC (PAPC/POPC activity ratio). The ratios for pLCAT, bLCAT, CHO LCAT, and HepG2 LCAT activity were 0.63, 0.49, 0.56, and 0.51 with cholesterol and 0.34, 0.29, 0.36, and 0.99 without cholesterol, respectively. We conclude that LCAT source influences glycosylation structure, which affects the apparent Vmax for cholesteryl ester formation with only minor changes in apparent Km or acyl substrate specificity.

483 ||| 8563134 ||| (3, 0.66) ||| 3 ||| 3 ||| Catabolism of glycan moieties of lipid intermediates leads to a single Man5GlcNAc oligosaccharide isomer: a study with permeabilized CHO cells. This paper presents kinetic and structural analyses of oligosaccharide material released during glycosylation in permeabilized Chinese hamster ovary cells incubated with sugar nucleotides. Permeabilized cells released 30 times more oligosaccharide material than metabolically labelled cells, normalized to the amount of labelled glycoprotein acceptor, making this an amenable system for study. Fifteen to forty per cent of the oligosaccharide material released by permeabilized cells was oligosaccharide-phosphate, depending on the nature and amount of the oligosaccharide-lipids synthesized. The oligosaccharide-phosphates released were recovered in the cytosol, and were exclusively Man2Glc-NAc2P and Man5GlcNAc2P, released from oligosaccharide-lipids thought to be facing the cytosol. In contrast, the structures found as neutral oligosaccharide material were similar to those attached to newly synthesized glycoproteins, indicating that the oligosaccharides were subjected to the same processing enzymes whether or not they were protein bound. Importantly, the kinetics of the transfer to protein and the release of free neutral oligosaccharide were parallel, suggesting that the same enzyme was responsible for both processes. Structural analyses demonstrated that the same Man5GlcNAc2 structure was transferred to protein and released as free oligosaccharide. Neutral oligosaccharides were found in both the cytosol and the pellet; however, oligosaccharides with one GlcNAc residue at the reducing end (OS-Gn1) were found exclusively in the supernate. The major neutral oligosaccharide produced after 2 h of metabolic labelling was Man5GlcNAc and it was found in the cytosol.

484 ||| 11886842 ||| (3, 0.66) ||| 3 ||| 3 ||| Glycosylation of the hepatitis C virus envelope protein E1 occurs posttranslationally in a mannosylphosphoryldolichol-deficient CHO mutant cell line. The addition of N-linked glycans to a protein is catalyzed by oligosaccharyltransferase, an enzyme closely associated with the translocon. N-glycans are believed to be transferred as the protein is being synthesized and cotranslationally translocated in the lumen of the endoplasmic reticulum. We used a mannosylphosphoryldolichol-deficient Chinese hamster ovary mutant cell line (B3F7 cells) to study the temporal regulation of N-linked core glycosylation of hepatitis C virus envelope protein E1. In this cell line, truncated Glc(3)Man(5)GlcNAc(2) oligosaccharides are transferred onto nascent proteins. Pulse-chase analyses of E1 expressed in B3F7 cells show that the N-glycosylation sites of E1 are slowly occupied until up to 1 h after protein translation is completed. This posttranslational glycosylation of E1 indicates that the oligosaccharyltransferase has access to this protein in the lumen of the endoplasmic reticulum for at least 1 h after translation is completed. Comparisons with the N-glycosylation of other proteins expressed in B3F7 cells indicate that the posttranslational glycosylation of E1 is likely due to specific folding features of this acceptor protein.

485 ||| 11092786 ||| (3, 0.66) ||| 3 ||| 3 ||| Bone induction by Escherichia coli -derived recombinant human bone morphogenetic protein-2 compared with Chinese hamster ovary cell-derived recombinant human bone morphogenetic protein-2. Most recombinant human bone morphogenetic protein (rhBMP) is currently obtained from Chinese hamster ovary (CHO) cells. If rhBMP with more activity could be derived from Escherichia coli (E. coli), a large quantity of rhBMP could be produced at low cost. The bone-inducing ability of an E. coli -derived rhBMP-2 (ErhBMP-2) variant with an N-terminal sequence was examined and compared with CHO cell-derived rhBMP-2 (CrhBMP-2). Two, 10, or 50 microgram of ErhBMP-2 or CrhBMP-2 was mixed with 3mg of atelopeptide type I collagen as the carrier, and specimens were implanted into the calf muscle pouches of Wistar rats (n= 5 in each group). Three weeks later, new bone had formed in all the ErhBMP-2-implanted and CrhBMP-2-implanted muscles. Radiographic and histological examinations showed that the bone induced by ErhBMP-2 had a large hollow bone matrix with more fatty marrow than the bone induced by CrhBMP-2. Quantitative analysis indicated that the activity of ErhBMP-2 was similar to that of CrhBMP-2, so ErhBMP-2 may be useful for inducing bone formation.

486 ||| 10510781 ||| (3, 0.66) ||| 3 ||| 3 ||| Aptamer affinity chromatography: combinatorial chemistry applied to protein purification. The systematic evolution of ligands by exponential enrichment process is a combinatorial chemistry method that allows the identification of specific oligonucleotide sequences, known as aptamers, that bind to a desired target molecule with high affinity and specificity. Here, a DNA-aptamer specific for human L-selectin was immobilized to a chromatography support to create an affinity column. This column was effectively applied as either the first or second step in the purification of a recombinant human L-selectin-Ig fusion protein from Chinese hamster ovary cell-conditioned medium. The fusion protein was efficiently bound to the column and efficiently eluted by gentle elution schemes. Application of the aptamer column as the initial purification step resulted in a 1500-fold purification with an 83% single step recovery. These results demonstrate that oligonucleotide aptamers can be effective affinity purification reagents.

487 ||| 7543523 ||| (3, 0.65) ||| 3 ||| 3 ||| Expression, purification, and characterization of the recombinant proform of eosinophil granule major basic protein. The cDNA for the highly toxic eosinophil granule major basic protein (MBP) encodes a 25-kDa acidic precursor (proMBP) that is processed to form the 14-kDa mature MBP. To characterize the biochemical and biological properties of proMBP, and compare these to the known properties of MBP, we expressed recombinant proMBP in Chinese hamster ovary cells and purified the secreted form from supernatants. We developed a mAb specific for proMBP, J163-15E10, and by using a proMBP-specific RIA we found that recombinant proMBP was expressed quite efficiently at levels between 10 and 100 mg/l. By SDS-PAGE and immunoblotting analyses of bulk Chinese hamster ovary supernatants, recombinant proMBP was electrophoretically heterogeneous with an apparent molecular mass ranging from 3 x 10(4) to 1 x 10(5) daltons. Despite difficulties encountered because of the extreme molecular heterogeneity of the proform, two methods for purification of a predominant 33-kDa form of recombinant proMBP are presented. Glycosylation analysis of purified 33-kDa proMBP indicated that approximately 5 kDa is likely accounted for by the addition of one glycosaminoglycan group, three O-linked, and one N-linked complex type carbohydrate groups. Functional studies of purified recombinant proMBP were also conducted. Using amounts of proMBP determined to be optimal for MBP activity, it was shown that proMBP not only lacked the ability to inhibit protein synthesis in K562 cells, but it also lacked the ability to stimulate basophil histamine release or generate neutrophil superoxide anion release. Furthermore, proMBP inhibited in a dose-responsive manner the basophil histamine release and superoxide anion generation stimulated by MBP. The development of a mAb and RIA specific for proMBP will now make it possible to analyze biologic fluids for the presence of this protein, especially in pregnancy, when proMBP is increased.

488 ||| 8535164 ||| (3, 0.64) ||| 3 ||| 3 ||| Chinese hamster rhodanese cDNA: activity of the expressed protein is not blocked by a C-terminal extension. We describe a cDNA from Chinese hamster ovary cells which encodes a protein 91 and 96% identical to bovine and rat mitochondrial rhodaneses, respectively. Recombinant protein was expressed from the cDNA in Escherichia coli, purified to homogeneity, and found to have kinetic properties nearly indistinguishable from those of the bovine enzyme, the only cloned rhodanese previously verified by characterization of the recombinant protein. The carboxyl-terminus of the enzyme is characterized by a duplicated tripeptide, which can be proteolytically processed in vivo. We constructed a mutant in which the last 5 amino acids were replaced by 28 residues of unrelated sequence. This protein was expressed, purified, and found to have kinetic constants similar to those of the wild-type enzyme. The functionally verified Chinese hamster rhodanese cDNA encodes a protein of 297 residues and differs from the rat enzyme at 13 positions. None of these substitutions occurs at residues suggested to play essential roles in catalysis or structural stabilization.

489 ||| 26011979 ||| (3, 0.64) ||| 3 ||| 3 ||| Expression of GPI anchored human recombinant erythropoietin in CHO cells is devoid of glycosylation heterogeneity. Erythropoietin is a glycohormone involved in the regulation of the blood cell levels. It is a 166 amino acid protein having 3 N-glycosylation and one O-linked glycosylation sites, and is used to treat anaemia related illness. Though human recombinant erythropoietin (rEPO) is produced in CHO cells, the loss in quality control is 80% due to incomplete glycosylation of the rEPO with low levels of fully glycosylated active rEPO. Here, we describe the expression from CHO cells of fully glycosylated human rEPO when expressed as a GPI anchored molecule (rEPO-g). The results demonstrated the production of a homogenous completely glycosylated human rEPO-g as a 42 kD band without any low molecular weight glycoform variants as shown by affinity chromatography followed by SDS-PAGE and anti-human EPO specific western blot. The western blot using specific monoclonal antibody is the available biochemical technique to prove the presence of homogeneity in the expressed recombinant protein. The GPI anchor can be removed during the purification process to yield a therapeutically relevant recombinant erythropoietin molecule cells with a higher in vivo biological activity due to its high molecular weight of 40 kD. This is possibly the first report on the production of a homogenous and completely glycosylated human rEPO from CHO cells for efficient therapy.

490 ||| 17559960 ||| (3, 0.64) ||| 3 ||| 3 ||| Optimisation of aqueous two-phase extraction of human antibodies. The purification of human antibodies in an aqueous two-phase system (ATPS) composed of polyethylene glycol (PEG) 6000 and phosphate was optimised by surface response methodology. A central composite design was used to evaluate the influence of phosphate, PEG and NaCl concentration and of the pH on the purity and extraction yield of IgG from a simulated serum medium. The conditions that maximise the partition of IgG into the upper phase were determined to be high concentrations of NaCl and PEG, low concentrations of phosphate and low pH values. An ATPS composed of 12% PEG, 10% phosphate, 15% NaCl at pH 6 was further used to purify human monoclonal antibodies from a Chinese Hamster Ovary (CHO) concentrated cell culture supernatant with a recovery yield of 88% in the upper PEG-rich phase and a purification factor of 4.3. This ATPS was also successfully used to purify antibodies from a hybridoma cell culture supernatant with a recovery yield of 90% and a purification factor of 4.1.

491 ||| 21599028 ||| (3, 0.63) ||| 3 ||| 3 ||| Identification of N-glycans displaying mannose-6-phosphate and their site of attachment on therapeutic enzymes for lysosomal storage disorder treatment. Characterization of mono- and bis-mannose-6-phosphate (M6P) bearing oligosaccharides present on acid hydrolase enzymes poses a considerable analytical challenge. In the current paper, we investigated the use of UPLC profiling on a 1.7 mum HILIC phase and capillary electrophoresis with laser induced fluorescence detection (CE-LIF) combined with exoglycosidase digestion and weak anion exchange fractionation for the characterization of M6P bearing glycans on recombinant beta-glucuronidase expressed in Chinese Hamster Ovary (CHO) cells. Using this multidimensional approach a number of peaks were observed to resist digestion, suggesting the presence and blocking activity of the M6P tag. To investigate further, mixed oxide affinity purification on a combined TiO(2)/ZrO(2) resin facilitated the selective enrichment of oligosaccharides bearing mono- or diphospho-esters that corresponded to those peaks previously identified to resist exoglycosidase digestion. Alkaline phosphatase digestion identified Man(6)GlcNAc(2) and Man(7)GlcNAc(2) glycans as the primary carriers of the M6P tag. Site-specific glycoproteomic analysis revealed that Man(7)GlcNAc(2)-M6P oligosaccharides were present at asparagine 272 and 420, while asparagine 631 displayed Man(6)GlcNAc(2)-M6P. The analytical strategy applied herein represents a novel yet simple approach for the qualitative and semiquantitative structural characterization of M6P containing oligosaccharides on therapeutic enzymes.

492 ||| 10903509 ||| (3, 0.63) ||| 3 ||| 3 ||| Recombinant glycodelin carrying the same type of glycan structures as contraceptive glycodelin-A can be produced in human kidney 293 cells but not in chinese hamster ovary cells. We have produced human recombinant glycodelin in human kidney 293 cells and in Chinese hamster ovary (CHO) cells. Structural analyses by lectin immunoassays and fast atom bombardment mass spectrometry showed that recombinant human glycodelin produced in CHO cells contains only typical CHO-type glycans and is devoid of any of the N, N'-diacetyllactosediamine (lacdiNAc)-based chains previously identified in glycodelin-A (GdA). By contrast, human kidney 293 cells produced recombinant glycodelin with the same type of carbohydrate structures as GdA. The presence of a beta1-->4-N-acetylgalactosaminyltransferase functioning in the synthesis of lacdiNAc-based glycans in human kidney 293 cells is concluded to be the cause of the occurrence of lacdiNAc-based glycans on glycodelin produced in these cells. Furthermore, human kidney 293 cells were found to be particularly suited for the production of recombinant glycodelin when they were cultured in high glucose media. Lowering the glucose concentration and the addition of glucosamine resulted in higher relative amounts of oligomannosidic-type glycans and complex glycans with truncated antennae. Human glycodelin is an attractive candidate for the development of a contraceptive agent, and this study gives valuable information for selecting the proper expression system and cell culture conditions for the production of a correctly glycosylated recombinant form.

493 ||| 10464766 ||| (3, 0.63) ||| 3 ||| 3 ||| Purification and characterization of two recombinant human granulocyte colony-stimulating factor glycoforms. Pharmacokinetic and activity studies of single-dose administration in mice. Two recombinant human granulocyte colony-stimulating factor (rhG-CSF) isoforms were isolated from the medium conditioned by an engineered Chinese hamster ovary (CHO) cell line. The two rhG-CSFs were characterized and were found to differ in the carbohydrate structure attached to Thr-133. The glycoform, referred to as Peak 1, contains the O-linked glycan Neu5Ac(alpha 2-3)Gal(beta 1-3)GalNAc; the Peak 2 glycoform contains the O-linked glycan Neu5Ac(alpha 2-3)Gal(beta 1-3)[Neu5Ac(alpha 2-6)]GalNAc. The two glycoforms displayed a similar biological activity in cultures of a mouse 32D C13 cell line and human bone-marrow myelo-monocytic progenitor cells (CFU-GM). In the latter test both glycoforms displayed a higher activity than nonglycosylated rMet-hG-CSF from Escherichia coli. The pharmacokinetic profile and activity of the two rhG-CSF glycoforms and of a mixture of them (Pool) were investigated in mice treated with a single injection of rhG-CSF at the doses of 125 micrograms and 250 micrograms/kg, given via the intravenous (i.v.) and the subcutaneous (s.c.) route, respectively. The plasma concentration profiles obtained were similar for all three substances and did not show any relevant differences in absorption or elimination. The pharmacokinetic parameters indicate that the three substances have similar area under the curve (AUCs), volumes of distribution, and terminal half-life. Furthermore, our data indicate a high bioavailability of the two different glycoforms of rhG-CSF when given to mice via the s.c. route either singularly or as a mixture. Detectable levels of rhG-CSF persisted for more than 8 h in the i.v. and more than 24 h in the s.c. route of administration. All three substances induced early neutrophilia in mice. All rhG-CSF-treated mice developed a two-four-fold rise in neutrophil counts as early as 4 h after the intravenous and 2 h after the subcutaneous injection. Relatively high levels of neutrophils were maintained for at least 8 and 24 h after i.v. and s.c. administration, respectively.

494 ||| 25499076 ||| (3, 0.62) ||| 3 ||| 3 ||| N-glycoprofiling analysis in a simple glycoprotein model: a comparison between recombinant and pituitary glycosylated human prolactin. Human prolactin (hPRL) is a polypeptide hormone occurring in the non-glycosylated (NG-hPRL) and glycosylated (G-hPRL) forms, with MM of approximately 23 and 25kDa, respectively. It has a single, partially occupied N-glycosylation site located at Asn-31, which makes it a particularly simple and interesting model for glycosylation studies. The bioactivity of G-hPRL is lower than that of NG-hPRL (by ca. 4-fold) and its physiological function is not clear. However, carbohydrate moieties generally play important roles in the biosynthesis, secretion, biological activity, and plasma survival of glycohormones and can vary depending on the host cell. The main objective of this study was to determine the N-glycan structures present in native, pituitary G-hPRL and compare them with those present in the recombinant hormone. To obtain recombinant G-hPRL, genetically modified Chinese hamster ovary cells (CHO), adapted to growth in suspension, were treated with cycloheximide, thus increasing the glycosylation site occupancy from 5.5% to 38.3%, thereby facilitating G-hPRL purification. CHO cell-derived G-hPRL (CHO-G-hPRL) was compared to pituitary G-hPRL (pit-G-hPRL) especially with regard to N-glycoprofiling. Among the main differences found in the pituitary sample were an extremely low presence of sialylated (1.7%) and a high percentage of sulfated (74.0%) and of fucosylated (90.5%) glycans. A ~6-fold lower in vitro bioactivity and a higher clearance rate in mice were also found for pit-G-hPRL versus CHO-G-hPRL. N-Glycan profiling proved to be a useful and accurate methodology also for MM and carbohydrate content determination for the two G-hPRL preparations, in good agreement with the values obtained directly via MALDI-TOF-MS.

495 ||| 24662970 ||| (3, 0.62) ||| 3 ||| 3 ||| Influence of glycosylation pattern on the molecular properties of monoclonal antibodies. Glycosylation is an important post-translational modification during protein production in eukaryotic cells, and it is essential for protein structure, stability, half-life, and biological functions. In this study, we produced various monoclonal antibody (mAb) glycoforms from Chinese hamster ovary (CHO) cells, including the natively glycosylated antibody, the enriched G0 form, the deglycosylated form, the afucosylated form, and the high mannose form, and we compared their intrinsic properties, side-by-side, through biophysical and biochemical approaches. Spectroscopic analysis indicates no measureable secondary or tertiary structural changes after in vitro or in vivo modification of the glycosylation pattern. Thermal unfolding experiments show that the high mannose and deglycosylated forms have reduced thermal stability of the CH2 domain compared with the other tested glycoforms. We also observed that the individual domain's thermal stability could be pH dependent. Proteolysis analysis indicates that glycosylation plays an important role in stabilizing mAbs against proteases. The stability of antibody glycoforms at the storage condition (2-8 C) and at accelerated conditions (30 and 40 C) was evaluated, and the results indicate that glycosylation patterns do not substantially affect the storage stability of the antibody we studied.

496 ||| 19056325 ||| (3, 0.62) ||| 3 ||| 3 ||| Downstream processing of human antibodies integrating an extraction capture step and cation exchange chromatography. In this paper we explore an alternative process for the purification of human antibodies from a Chinese hamster ovary (CHO) cell supernatant comprising a ligand-enhanced extraction capture step and cation exchange chromatography (CEX). The extraction of human antibodies was performed in an aqueous two-phase system (ATPS) composed of dextran and polyethylene glycol (PEG), in which the terminal hydroxyl groups of the PEG molecule were modified with an amino acid mimetic ligand in order to enhance the partition of the antibodies to the PEG-rich phase. This capture step was optimized using a design of experiments and a central composite design allowed the determination of the conditions that favor the partition of the antibodies to the phase containing the PEG diglutaric acid (PEG-GA) polymer, in terms of system composition. Accordingly, higher recovery yields were obtained for higher concentrations of PEG-GA and lower concentrations of dextran. The highest yield experimentally obtained was observed for an ATPS composed of 5.17% (w/w) dextran and 8% (w/w) PEG-GA. Higher purities were however predicted for higher concentrations of both polymers. A compromise between yield and purity was achieved using 5% dextran and 10% PEG-GA, which allowed the recovery of 82% of the antibodies with a protein purity of 96% and a total purity of 63%, determined by size-exclusion chromatography. ATPS top phases were further purified by cation exchange chromatography and it was observed that the most adequate cation exchange ligand was carboxymethyl, as the sulfopropyl ligand induced the formation of multi-aggregates or denatured forms. This column allowed the elution of 89% of the antibodies present in the top phase, with a protein purity of 100% and a total purity of 91%. The overall process containing a ligand-enhanced extraction step and a cation exchange chromatography step had an overall yield of 73%.

497 ||| 11270813 ||| (3, 0.62) ||| 3 ||| 3 ||| Characterization of cytosolic sialidase from Chinese hamster ovary cells: part II. Substrate specificity for gangliosides. Cytosolic Chinese hamster ovary (CHO) cell sialidase has been cloned as a soluble glutathione S-transferase (GST)-sialidase fusion protein with an apparent molecular weight of 69 kD in Escherichia coli. The enzyme has then been produced in mg quantities at 25-L bioreactor scale and purified by one-step affinity chromatography on glutathione sepharose (Burg, M.; Muthing, J. Carbohydr. Res. 2001, 330, 335-346). The cloned sialidase was probed for desialylation of a wide spectrum of different types of gangliosides using a thin-layer chromatography (TLC) overlay kinetic assay. Different gangliosides were separated on silica gel precoated TLC plates, incubated with increasing concentrations of sialidase (50 degreesU/mL up to 1.6 mU/mL) without detergents, and desialylated gangliosides were detected with specific anti-asialoganglioside antibodies. The enzyme exhibited almost identical hydrolysis activity in degradation of GM3(Neu5Ac) and GM3(Neu5Gc). A slightly enhanced activity, compared with reference Vibrio cholerae sialidase, was detected towards terminally alpha(2-3)-sialylated neolacto-series gangliosides IV3-alpha-Neu5Ac-nLc4Cer and VI3-alpha-Neu5Ac-nLc6Cer. The ganglio-series gangliosides G(D1a), G(D1b), and G(T1b), the preferential substrates of V. cholerae sialidase for generating cleavage-resistant G(M1), were less suitable targets for the CHO cell sialidase. The increasing evidence on colocalization of gangliosides and sialidase in the cytosol strongly suggests the involvement of the cytosolic sialidase in ganglioside metabolism on intracellular level by yet unknown mechanisms.

498 ||| 22570865 ||| (3, 0.61) ||| 3 ||| 3 ||| Recombinant IgA production: single step affinity purification using camelid ligands and product characterization. Immunoglobulins of isotype A (IgA) mediate a key role in mucosal immunity and are promising new immunotherapeutic candidates, but difficulties in obtaining enough material often hamper their in vivo exploration. We established recombinant Chinese hamster ovary (CHO) cells which stably expressed two IgA1 antibodies under serum-free conditions. The two cell lines achieved significantly different specific productivities of 16 pg per cell and day and 100 times less, a common phenomenon in recombinant antibody expression which challenges the production and purification process. Polymeric IgA in crude culture supernatants was assembled with J chain and showed expected specificity. We employed an immobilized camelid anti-human alpha-chain VHH ligand and isolated both recombinant IgAs at high purity and yield in a single chromatographic step. The described method was irrespective of the light chain and specificity and may be used as a generic capture step for the isolation of any IgA. Results were compared with a multistep purification process consisting of an affinity step based on immobilized jacalin followed by anion exchange and hydrophobic interaction chromatography.

499 ||| 20860349 ||| (3, 0.61) ||| 3 ||| 3 ||| Re-engineering erythropoietin as an IgG fusion protein that penetrates the blood-brain barrier in the mouse. Erythropoietin (EPO) is a potent neuroprotective agent that could be developed for the treatment of multiple brain disorders. However, EPO does not cross the blood-brain barrier (BBB). A brain-penetrating form of EPO, specific for the mouse, was engineered by fusion of the 166 amino acid EPO to the carboxyl terminus of the heavy chain of a chimeric monoclonal antibody (mAb) against the mouse transferrin receptor (TfR), and this new fusion protein is designated cTfRMAb-EPO. The fusion protein was expressed in stably transfected Chinese hamster ovary cells and purified by protein G chromatography. The fusion protein was homogeneous on SDS-PAGE and Western blotting, and bound the mouse EPO receptor (EPOR) with high affinity, ED50 = 0.33 +- 0.04 nM. The cTfRMAb-EPO fusion protein was radiolabeled by tritiation and injected intravenously (iv) into adult mice for measurements of the plasma pharmacokinetics and brain uptake. The (3)H-fusion protein was rapidly cleared from the blood with a clearance rate of 5.9 +- 0.3 mL/min/kg, which is 14-fold faster than the clearance of EPO in the mouse. The cTfRMAb-EPO fusion protein penetrated the BBB in vivo, as shown by the capillary depletion method. The brain uptake of the cTfRMAb-EPO fusion protein was 2.0 +- 0.1% injected dose/g of brain following iv administration. The high level of brain uptake of the fusion protein enables pharmacologic increases in exogenous EPO in the mouse brain following the systemic injection of the cTfRMAb-EPO fusion protein. In conclusion, EPO has been re-engineered as an IgG fusion protein that binds dual receptors: the mouse TfR, to enable penetration of the BBB, and the mouse EPOR, to produce neuroprotection in brain behind the intact BBB.

500 ||| 19366632 ||| (3, 0.61) ||| 3 ||| 3 ||| Stable expression of a human-like sialylated recombinant thyrotropin in a Chinese hamster ovary cell line expressing alpha2,6-sialyltransferase. A CHO cell line, previously genetically modified by the introduction of rat alpha2,6-sialyltransferase cDNA, generated for the first time a human-like sialylated recombinant hTSH (hlsr-hTSH) more similar to the native hormone, with 61% of alpha2,3- and 39% of alpha2,6-linked sialic acid residues. The best clone, when submitted to gene amplification with up to 8 microM methotrexate, presented a secretion level of approximately 2 microg hTSH/10(6)cells/day, useful for product purification and characterization. The relative molecular masses (M(r)) of the heterodimer and of the alpha- and beta-subunits of purified hlsr-hTSH, determined by MALDI-TOF mass spectrometry, and the relative hydrophobicities, determined by RP-HPLC, were not remarkably different from those presented by two r-hTSH preparations secreted by normal CHO cells. Some differences were observed, though, in N-glycan composition, with more tri- and much more tetra-sialylated structures in hlsr-hTSH. When analyzed via an in vivo bioassay based on hTSH-induced T(4) release in mice, hlsr-hTSH was shown to be equipotent (p>0.05) with the commercial preparation of r-hTSH (Thyrogen), and 1.6-fold more potent than native hTSH (p<0.001).

501 ||| 19135424 ||| (3, 0.61) ||| 3 ||| 3 ||| Mass spectrometric quantification of neutral and sialylated N-glycans from a recombinant therapeutic glycoprotein produced in the two Chinese hamster ovary cell lines. Quality control and assurance of glycan profiles of a recombinant glycoprotein from lot to lot is a critical issue in the pharmaceutical industry. To develop an easy and simple quantitative and qualitative glycan profile method based on matrix-assisted laser desorption/ionization-time-of-flight mass spectrometry (MALDI-TOF MS), the modification with Girard's reagent T (GT) was exploited. Because GT-derivatized quantification of oligosaccharides using MALDI-TOF MS is possible only with neutral glycans, sialylated glycans are not subjected to quantitative analysis with MALDI-TOF MS. To solve this problem, mild methyl esterification and subsequent GT derivatization were employed, enabling us to perform rapid qualitative and quantitative analysis of sialylated and neutral N-linked oligosaccharides using MALDI-TOF MS. This modified method was used in the comparative quantification of N-glycans from the recombinant therapeutic glycoprotein expressed in two different Chinese hamster ovary (CHO) cell lines. The percentages of sialylated N-glycans to total were 22.5 and 5.2% in CHO-I and CHO-II cells, respectively, resulting in a significant difference in the biological activity of the recombinant glycoprotein.

502 ||| 9784244 ||| (3, 0.60) ||| 3 ||| 3 ||| Transfer of two oligosaccharides to protein in a Chinese hamster ovary cell B211 which utilizes polyprenol for its N-linked glycosylation intermediates. B211, a glycosylation mutant isolated from Chinese hamster ovary cells, synthesizes 10- to 15-fold less Glc3Man9GlcNAc2-P-P-lipid, the substrate used by the oligosaccharide transferase in the synthesis of asparagine-linked glycoproteins. B211 cells are also 10- to 15-fold deficient in the glucosylation of oligosaccharide-lipid. Despite these properties, protein glycosylation in B211 cells proceeds at a level similar to (50% of) parental cells. We asked whether the near wild-type level of glycosylation was due to the transfer of alternative oligosaccharide structures to protein in B211 cells. The aberrant size of [35S]methionine-labeled VSV G protein and the increased percentage of endoglycosidase H-resistant tryptic peptides as compared to parental cells supported this hypothesis. B211 cells were labeled with [2-3H]mannose either for 1 min or for 1 h in the presence of glycoprotein-processing inhibitors so that the oligosaccharides initially transferred to protein could be analyzed. In addition to Glc3Man9GlcNAc2, a second, endoglycosidase H-resistant oligosaccharide was transferred whose structure was determined by alpha-mannosidase digestion, gel filtration chromatography, and HPLC to be Glc0,1Man5GlcNAc2. Finally, since the synthesis of reduced amounts of Glc3Man9GlcNAc2-P-P-lipid was also a phenotype seen in another glycosylation mutant, Lec9, we analyzed the long-chain prenol in B211 cells. B211 cells synthesized and utilized polyprenol rather than dolichol for all N-linked glycosylation intermediates as determined by HPLC analysis of [3H]mevalonate-labeled lipids. Cell fusions analyzed by similar techniques indicated that B211, originally isolated as a concanavalin A-resistant cell line, is in the Lec9 complementation group.

503 ||| 10722674 ||| (3, 0.60) ||| 3 ||| 3 ||| Purification of the serine palmitoyltransferase complex responsible for sphingoid base synthesis by using affinity peptide chromatography techniques. Serine palmitoyltransferase (SPT), a membrane-bound enzyme of the endoplasmic reticulum, catalyzes the condensation of palmitoyl coenzyme A (CoA) and L-serine to produce 3-ketodihydrosphingosine. This enzyme contains at least two different subunits, named the LCB1 and LCB2 proteins. In the present study, we expressed a FLAG- and His(6) peptide-tagged version of the hamster LCB1 protein in a Chinese hamster ovary cell mutant strain lacking the endogenous LCB1 subunit and purified SPT from the cells near to homogeneity by affinity peptide chromatography. The endogenous LCB2 protein was co-purified with the tagged LCB1 protein in purification of SPT. In various aspects, including optimum pH, acyl-CoA specificity, and sphingofungin sensitivity, the activity of purified SPT was consistent with the activity detected in lysates of wild-type Chinese hamster ovary cells. The optimum concentration of palmitoyl-CoA for 3-ketodihydrosphingosine formation by purified SPT was approximately 25 microM, and the apparent K(m) of L-serine was 0.28 mM. Competition analysis of the SPT reaction with various serine analogs showed that all of the amino, carboxyl, and hydroxyl groups of L-serine were responsible for the substrate recognition of the enzyme. SDS-polyacrylamide gel electrophoretic analysis of purified SPT, together with immunoprecipitation analysis of metabolically labeled LCB proteins, strongly suggested that the SPT enzyme consisted of the LCB1 and LCB2 proteins with a stoichiometry of 1:1.

504 ||| 9806900 ||| (3, 0.59) ||| 3 ||| 3 ||| Partial purification of heparanase activities in Chinese hamster ovary cells: evidence for multiple intracellular heparanases. Heparanases are mammalian endoglycosidases that cleave heparan sulphate glycosaminoglycans from proteoglycan core proteins and degrade them into shorter chains. The enzymes have been proposed to act in a variety of cellular processes, including proteoglycan catabolism, remodelling of basement membranes and release of heparan sulphate-binding ligands from their extracellular storage sites. Additional functions for heparanases may be to generate short heparan sulphate chains that stabilize or activate other proteins. While heparanase activities have been described in a number of tissues and cell lines, it is not known how many different enzymes are responsible for these activities. Our recent studies characterizing the short glycosaminoglycans produced in Chinese hamster ovary (CHO) cells suggested that multiple heparanases are necessary for the formation of the short heparan sulphate chains [Bame and Robson (1997) J. Biol. Chem. 272, 2245-2251]. We examined whether this is the case by purifying heparanase activity from CHO cell homogenates. Based on their ability to bind ion-exchange resins and their elution from gel-filtration columns, four separate heparanase activities were partially purified. All four activities cleave free glycosaminoglycans over a broad pH range of 3.5-6.0 or 6. 5, suggesting that they act in the endosomal/lysosomal pathway. The sizes of the short heparan sulphate chains generated by the partially purified heparanases ranged from 6 to 9 kDa, and for two of the activities the product size is pH-dependent. Three of the four activities degrade proteoglycans as well as the free glycosaminoglycan chain. Interestingly, all four enzymes generate short glycosaminoglycans with a sulphate-rich, modified domain at the non-reducing end of the newly formed chain. Since our previous studies showed that in CHO cells there is also a population of short heparan sulphates with a modified domain at the reducing end of the chain, this suggests that there may be another heparanase in CHO cells that was not purified. Alternatively, our findings suggest that the formation of short heparan sulphate glycosaminoglycans inside CHO cells may be a result of the concerted action of multiple heparanases, and may depend on the proportions of the different enzymes and the environment in which the chains are degraded.

505 ||| 9698230 ||| (3, 0.59) ||| 3 ||| 3 ||| Quantitative analysis and process monitoring of site-specific glycosylation microheterogeneity in recombinant human interferon-gamma from Chinese hamster ovary cell culture by hydrophilic interaction chromatography. A chromatographic method was developed for quantitative analysis of site-specific microheterogeneity of the two N-linked glycosylation sites in recombinant human interferon-gamma produced from Chinese hamster ovary (CHO) cell culture. After the interferon-gamma was harvested by affinity chromatography, the tryptic digestion was carried out. The two glycopeptide pools, isolated from reversed-phase chromatography of tryptic digestion of interferon-gamma, were subjected to further separation by hydrophilic interaction chromatography. Each peak in the chromatograms was identified by matrix-assisted laser desorption ionization and time-of-flight mass spectrometry (MALDI-TOF-MS). The overall elution order of the glycopeptides was the following: neutral glycopeptides, monosialylated glycopeptides, bisialylated glycopeptides, trisialylated glycopeptide and tetrasialylated glycopeptides. Based on the integrated peak area for each compound in the chromatograms, the percentage for each glycan was utilized to quantify the glycosylation pattern of the interferon-gamma. Finally, sialylation and antennarity structure percentages at the two glycosylation sites were chosen as the quality indicators in process monitoring of interferon-gamma production from a serum-free suspension-batch CHO culture.

506 ||| 24391029 ||| (3, 0.59) ||| 3 ||| 3 ||| Expression and fast preparation of biologically active recombinant human coagulation factor VII in CHO-K1 cells. Human coagulation factor VII (FVII) plays an important role in the blood coagulation process and exists in micro amounts in human plasma; therefore, any attempt at the large-scale production of FVII in significant quantities is challenging. The purpose of this study was to express and obtain biologically active recombinant FVII (rFVII) from Chinese hamster ovary K1 (CHO-K1) cells. The full-length FVII cDNA was isolated from a HepG2 cell line and then subcloned in pcDNA3.1 to construct an expression vector, pcDNA-FVII. CHO-K1 cells were transfected with 1 microg pcDNA-FVII. The cell line that stably expressed secretory FVII was screened using 900 microg/mL G418. The FVII copy number in CHO-K1 cells was detected by quantitative polymerase chain reaction (qPCR). The rFVII was purified in ligand affinity chromatography medium. The purified protein was detected by sodium dodecyl sulfate-polyacrylamide gel electrophoresis (SDS-PAGE) and Western blot analysis. The biological activity of the purified FVII protein was determined by a prothrombin time assay. Three cell lines that permanently expressed rFVII were screened. The qPCR results demonstrated that each CHO-K1 cell harbored two FVII DNA copies. The SDS-PAGE and Western blot analysis showed that the purified protein was about 50 kDa. The purity of the target protein was 95%. The prothrombin time assay indicated that the FVII-specific activity of rFVII was 2573 +- 75 IU/mg. This method enabled the fast preparation of high-purity rFVII from CHO-K1 cells, and the purified protein had good biological activity.

507 ||| 24326352 ||| (3, 0.59) ||| 3 ||| 3 ||| Glycosylation analysis of an aggregated antibody produced by Chinese hamster ovary cells in bioreactor culture. N-Glycosylation of therapeutic antibodies contributes not only to their biological function, but also to their stability and tendency to aggregate. Here, we investigated the impact of the glycosylation status of an aggregated antibody that accumulated during the bioreactor culture of Chinese hamster ovary cells. High-performance liquid chromatography analysis showed that there was no apparent difference in the glycosylation patterns of monomeric, dimeric, and large aggregated forms of the antibody. In contrast, lectin binding assays, which enable the total amounts of specific sugar residues to be detected, showed that both galactose and fucose residues in dimers and large aggregates were reduced to 70-80% of the amount in monomers. These results strongly suggest that the lack of N-linked oligosaccharides, a result of deglycosylation or aglycosylation, occurred in a proportion of the dimeric and large aggregated components. The present study demonstrates that glycosylation heterogeneities are a potential cause of antibody aggregation in cell culture of Chinese hamster ovary cells, and that the lack of N-glycosylation promotes the formation of dimers and finally results in large aggregates.

508 ||| 21041724 ||| (3, 0.59) ||| 3 ||| 3 ||| Fc-glycosylation influences Fcgamma receptor binding and cell-mediated anti-HIV activity of monoclonal antibody 2G12. Interactions between the Fc segment of IgG and FcgammaRs on a variety of cells are likely to play an important role in the anti-HIV activity of Abs. Because the nature of the glycan structure on the Fc domain is a critical determinant of Fc-FcgammaR binding, proper Fc glycosylation may contribute to Ab-mediated protection. We have generated five different glycoforms of the broadly HIV-1-neutralizing mAb 2G12 in wild-type and glycoengineered plants and Chinese hamster ovary cells. Plant-derived 2G12 exhibited highly homogeneous glycosylation profiles with a single dominant N-glycan species. Using flow cytometry with FcgammaR-expressing cell lines, all 2G12 glycoforms demonstrated similar binding to FcgammaRI, FcgammaRIIa, and FcgammaRIIb. In contrast, two glycoforms derived from glycoengineered plants that lack plant-specific xylose and core alpha1,3-fucose, and instead carry human-like glycosylation with great uniformity, showed significantly enhanced binding to FcgammaRIIIa compared with Chinese hamster ovary or wild-type plant-derived 2G12. Using surface plasmon resonance, we show that binding of 2G12 to FcgammaRIIIa is markedly affected by core fucose, irrespective of its plant-specific alpha1,3 or mammalian-type alpha1,6 linkage. Consistent with this finding, 2G12 glycoforms lacking core fucose (and xylose) mediated higher antiviral activity against HIV-1 or simian immunodeficiency virus as measured by Ab-dependent cell-mediated virus inhibition. This is, to our knowledge, the first demonstration that specific alterations of Fc glycosylation can improve antiviral activity. Such alterations may result in better immunotherapeutic reagents. Moreover, biasing vaccine-induced immune responses toward optimal Fc glycosylation patterns could result in improved vaccine efficacy.

509 ||| 8621667 ||| (3, 0.58) ||| 3 ||| 3 ||| The natural mutation Y248C of human angiotensinogen leads to abnormal glycosylation and altered immunological recognition of the protein. Common molecular variants of the angiotensinogen gene have been associated with human hypertension. The rare Tyr to Cys change at residue 248 of mature angiotensinogen was identified in one pedigree. Heterozygous individuals (Y248C) had a 40% decrease in plasma angiotensinogen concentration and a 35% reduction of the angiotensin I production rate. Recombinant wild-type (Tyr-248) and mutant (Cys-248) proteins were stably expressed in Chinese hamster ovary cells. Angiotensinogen monoclonal antibodies revealed marked differences in the epitope recognition of the mutant protein and allowed the demonstration of its presence in plasma of Y248C individuals. Similar kinetic constants of angiotensin I production with human renin were observed for both proteins. Western blot analysis showed similar heterogeneities; however, a 3-kDa increase in molecular mass for the Cys-248 protein was observed after immunopurification. Metabolic labeling of the intracellular Cys-248 protein showed a 61-kDa band in addition to the 55.5- and 58-kDa bands observed for the Tyr-248 protein, with all bands being sensitive to endoglycosidase H. In addition, pulse-chase studies revealed a slower intracellular processing for the Cys-248 protein. In conclusion, the Cys-248 mutation alters the structure, glycosylation, and secretion of angiotensinogen in Chinese hamster ovary cells and is accompanied by a decrease in plasma angiotensinogen concentration in Y248C individuals.

510 ||| 11504739 ||| (3, 0.58) ||| 3 ||| 3 ||| A remodeling system of the 3'-sulfo-Lewis a and 3'-sulfo-Lewis x epitopes. It has been reported that the chemically synthesized 3'-sulfo-Le(a) and 3'-sulfo-Le(x) epitopes have a high potential as a ligand for selectins. To elucidate the physiological functions of 3'-sulfated Lewis epitopes, a remodeling system was developed using a combination of a betaGal-3-O-sulfotransferase GP3ST, hitherto known alpha1,3/1,4-fucosyltransferases (FucT-III, IV, V, VI, VII, and IX) and arylsulfatase A. The pyridylaminated (PA) lacto-N-tetraose (Galbeta1-3GlcNAcbeta1-3Galbeta1-4Glc) was first converted to 3'-sulfolacto-N-fucopentaose II (sulfo-3Galbeta1-3(Fucalpha1-4)GlcNAcbeta1-3Galbeta1-4Glc)-PA by sequential reactions with GP3ST and FucT-III. The 3'-sulfolacto-N-fucopentaose III (sulfo-3Galbeta1-4(Fucalpha1-3)GlcNAcbeta1-3Galbeta1-4Glc)-PA was then synthesized from lacto-N-neotetraose (Galbeta1-4GlcNAcbeta1-3Galbeta1-4Glc)-PA by GP3ST and FucT-III, -IV, -V, -VI, -VII, or -IX in a similar manner. The substrate specificity for the 3'-sulfated acceptor of the alpha1,3-fucosyltransferases was considerably different from that for the non-substituted and 3'-sialylated varieties. When the GP3ST gene was introduced into A549 and Chinese hamster ovary cells expressing FucT-III, they began to express 3'-sulfo-Le(a) and 3'-sulfo-Le(x) epitopes, respectively, suggesting that GP3ST is responsible for their biosynthesis in vivo. The expression of the 3'-sialyl-Le(x) epitope on Chinese hamster ovary cells was attenuated by the introduction of GP3ST gene, indicating that GP3ST and alpha2,3-sialyltransferase compete for the common Galbeta1-4GlcNAc-R oligosaccharides. Last, arylsulfatase A, which is a lysosomal hydrolase that catalyzes the desulfation of 3-O-sulfogalactosyl residues in glycolipids, was found to hydrolyze the sulfate ester bond on the 3'-sulfo-Le(x) (type 2 chain) but not that on the 3'-sulfo-Le(a) (type 1 chain). The present remodeling system might be of potential use as a tool for the study of the physiological roles of 3'-sulfated Lewis epitopes, including interaction with selectins.

511 ||| 23182963 ||| (3, 0.57) ||| 3 ||| 3 ||| A novel approach for the simultaneous quantification of a therapeutic monoclonal antibody in serum produced from two distinct host cell lines. Therapeutic monoclonal antibodies (mAbs) possess a high degree of heterogeneity associated with the cell expression system employed in manufacturing, most notably glycosylation. Traditional immunoassay formats used to quantify therapeutic mAbs are unable to discriminate between different glycosylation patterns that may exist on the same protein amino acid sequence. Mass spectrometry provides a technique to distinguish specific glycosylation patterns of the therapeutic antibody within the same sample, thereby allowing for simultaneous quantification of the same mAb with different glycosylation patterns. Here we demonstrate a two-step approach to successfully differentiate and quantify serum mixtures of a recombinant therapeutic mAb produced in two different host cell lines (CHO vs. Sp2/0) with distinct glycosylation profiles. Glycosylation analysis of the therapeutic mAb, CNTO 328 (siltuximab), was accomplished through sample pretreatment consisting of immunoaffinity purification (IAP) and enrichment, followed by liquid chromatography (LC) and mass spectrometry (MS). LC-MS analysis was used to determine the percentage of CNTO 328 in the sample derived from either cell line based on the N-linked G1F oligosaccharide on the mAb. The relative amount of G1F derived from each cell line was compared with ratios of CNTO 328 reference standards prepared in buffer. Glycoform ratios were converted to concentrations using an immunoassay measuring total CNTO 328 that does not distinguish between the different glycoforms. Validation of the IAP/LC-MS method included intra-run and inter-run variability, method sensitivity and freeze-thaw stability. The method was accurate (%bias range = -7.30-13.68%) and reproducible (%CV range = 1.49-10.81%) with a LOQ of 2.5 mug/mL.

512 ||| 10438455 ||| (3, 0.57) ||| 3 ||| 3 ||| Human homolog of Caenorhabditis elegans sqv-3 gene is galactosyltransferase I involved in the biosynthesis of the glycosaminoglycan-protein linkage region of proteoglycans. A cDNA encoding a novel galactosyltransferase was identified based on BLAST analysis of expressed sequence tags, and the cDNA clones were isolated from a human melanoma line library. The new cDNA sequence encoded a type II membrane protein with 327 amino acid sequence and showed 38% homology to the Caenorhabditis elegans sqv-3 gene involved in the vulval invagination and oocyte development. Extracts from L cells transfected with the galactosyltransferase cDNA in an expression vector and a fusion protein with protein A exhibited marked galactosyltransferase activity specific for p-nitrophenyl-beta-D-xylopyranoside. Moreover, transfection with the cloned cDNA restored glycosaminoglycan synthesis of galactosyltransferase I-deficient Chinese hamster ovary mutant pgsB-761 cells. Analysis of the enzyme product by beta-galactosidase digestion, mass spectroscopy, and NMR spectroscopy revealed that the reaction product was formed via beta-1,4 linkage, indicating that the enzyme is galactosyltransferase I (UDP-galactose:O-beta-D-xylosylprotein 4-beta-D-galactosyltransferase, EC 2.4.1.133) involved in the synthesis of the glycosaminoglycan-protein linkage region of proteoglycans.

513 ||| 7753821 ||| (3, 0.56) ||| 3 ||| 3 ||| Glycosylation of the c-Myc transactivation domain. O-linked N-acetylglucosamine (O-GlcNAc) is an abundant and dynamic posttranslational modification composed of a single monosaccharide, GlcNAc, glycosidically composed of a single monosaccharide, GlcNAc, glycosidically linked to the side-chain hydroxyl of serine or threonine residues. Although O-GlcNAc occurs on a myriad of nuclear and cytoplasmic proteins, only a few have thus far been identified. These O-GlcNAc-bearing proteins are also modified by phosphorylation and form reversible multimeric complexes. Here we present evidence for O-GlcNAc glycosylation of the oncoprotein c-Myc, a helix-loop-helix/leucine zipper phosphoprotein that heterodimerizes with Max and participates in the regulation of gene transcription in normal and neoplastic cells. O-GlcNAc modification of c-Myc is shown by three different methods: (i) demonstration of lectin binding to in vitro translated protein using a protein-protein interaction mobility-shift assay; (ii) glycosidase or glycosyltransferase treatment of in vitro translated protein analyzed by lectin affinity chromatography; and (iii) direct characterization of the sugar moieties on purified recombinant protein overexpressed in either insect cells or Chinese hamster ovary cells. Analyses of serial deletion mutants of c-Myc further suggest that the O-GlcNAc site(s) are located within or near the N-terminal transcription activation/malignant transformation domain, a region where mutations of c-Myc that are frequently found in Burkitt and AIDS-related lymphomas cluster.

514 ||| 11357880 ||| (3, 0.56) ||| 3 ||| 3 ||| Recombinant expression of human mannan-binding lectin. Mannan-binding lectin (MBL) constitutes an important part of the innate immune defence by effecting the deposition of complement on microbial surfaces. MBL deficiency is among the most common primary immunodeficiencies and is associated with recurrent infections and symptoms of poor immune complex clearance. Plasma-derived MBL has been used in reconstitution therapy but concerns over viral contamination and production capacity point to recombinant MBL (rMBL) as a future source of this protein for clinical use. Natural human MBL is an oligomer of up to 18 identical polypeptide chains. The synthesis of rMBL has been accomplished in several mammalian cell lines, however, the recombinant protein differed structurally from natural MBL. In this, study we compare rMBL produced in myeloma cells, Chinese hamster ovary (CHO) cells, human hepatocytes, and human embryonic kidney (HEK) cells. We report that rMBL structurally and functionally similar to natural MBL can be obtained through synthesis in the human embryonic kidney cells followed by selective carbohydrate affinity chromatography.

515 ||| 8815742 ||| (3, 0.55) ||| 3 ||| 3 ||| Rapid monitoring of site-specific glycosylation microheterogeneity of recombinant human interferon-gamma. An analytical system is presented for rapid assessment of site-specific microheterogeneity of the two potential N-linked glycosylation sites of recombinant human interferon-gamma (IFN-gamma) derived from Chinese hamster ovary cell culture. The target protein is first purified from culture supernatant by immunoaffinity chromatography, and the acidic eluent is neutralized via an in-line mixing tee. On-line proteolysis is rapidly performed by an immobilized trypsin cartridge, and reversed-phase chromatography isolates the two pools of glycopeptides representing the potential glycosylation sites. Following off-line analysis by matrix-assisted laser-desorption ionization/time-of-flight (MALDI/TOF) mass spectrometry, observed mass shifts of glycopeptides relative to the known masses of their amino acid portions are correlated to site-specific oligosaccharide structures. Desialylation of glycopeptides by sialidase treatment on the MALDI sample plate allows for quantitative estimations of asialoglycan structures by MALDI/TOF. This methodology permits glycoprotein microheterogeneity to be evaluated in a time frame of approximately 2 h, utilizing as little as 0.5 microgram (25 pmol) of product. Results of monitoring a batch culture are presented as well as analysis of a culture containing deoxymannojirimycin, an inhibitor of glycoprotein processing.

516 ||| 11294837 ||| (3, 0.55) ||| 3 ||| 3 ||| Monoglucosylated oligomannosides are released during the degradation process of newly synthesized glycoproteins. The Chinese hamster ovary mutant MI8-5 is known to synthesize Man(9)GlcNAc(2)-P-P-dolichol rather than the fully glucosylated lipid intermediate Glc(3)Man(9)GlcNAc(2)-P-P-dolichol. This nonglucosylated oligosaccharide lipid precursor is used as donor for N-glycosylation. In this paper we demonstrate that a significant part of the glycans bound to the newly synthesized glycoproteins in MI8-5 cells are monoglucosylated. The presence of monoglucosylated glycans on glycoproteins determines their binding to calnexin as part of the quality control machinery. Furthermore, we point out the presence of Glc(1)Man(5)GlcNAc(1) in the cytosol of MI8-5 cells. This indicates that part of the monoglucosylated glycoproteins can be directed toward a deglycosylation process that occurs in the cytosol. Besides studies on glycoprotein degradation based on the disappearance of protein moieties, MI8-5 cells can be used as a tool to elucidate the various step leading to glycoprotein degradation by studying the fate of the glycan moieties.

517 ||| 11270812 ||| (3, 0.55) ||| 3 ||| 3 ||| Characterization of cytosolic sialidase from Chinese hamster ovary cells: part I: cloning and expression of soluble sialidase in Escherichia coli. The cDNA of Chinese hamster ovary (CHO) cell cytosolic sialidase was amplified by RT-PCR and cloned into the pGEX-2T plasmid vector encoding for glutathione S-transferase (GST). Screening revealed transformed Escherichia coli clones with the constructed plasmid encoding the CHO cell sialidase sequence. After isopropyl-beta-D-thiogalactopyranoside (IPTG) induction, SDS-PAGE of the total protein extracts revealed a new protein of about 70 kDa, correlating with the molecular weight of a fusion protein composed of the GST (26 kDa) and the cloned cytosolic CHO cell sialidase (43 kDa). A soluble fusion protein was purified from sonified E. coli homogenates by one-step affinity chromatography on Glutathione Sepharose 4B, which showed sialidase activity towards 4-methyl-umbelliferyl-alpha-D-N-acetylneuraminic acid (MUF-Neu5Ac) substrate. Induction of cells with 0.1, 0.5, and 1.0 mM IPTG revealed highest total protein amounts after induction with 1.0 mM IPTG, but highest specific activity for affinity chromatography purified eluates from cultures induced with 0.1 mM IPTG. Therefore, large scale production was performed by inducing cells during exponential growth in a 25 L bioreactor for 3 h with 0.1 mM IPTG after chilling the cell suspension to 25 degrees C. The amount of 26.46 mg of 40-fold purified GST-sialidase with a specific activity of 0.999 U/mg protein was obtained from crude protein extracts by one-step affinity chromatography. 2-Deoxy-2,3-dehydro-N-acetylneuraminic acid (Neu5Ac2en) and Neu5Ac were competitive inhibitors for the sialidase, the former being the more effective one using MUF-Neu5Ac as the substrate. The cytosolic sialidase is capable of desialylating a wide spectrum of different types of gangliosides using a thin-layer chromatography overlay kinetic assay without detergents. This is the subject of the accompanying paper (Muthing, J.; Burg, M. Carbohydr. Res. 2001, 330, 347-356).

518 ||| 10833408 ||| (3, 0.55) ||| 3 ||| 3 ||| Purification and characterization of recombinant human alpha-N-acetylglucosaminidase secreted by Chinese hamster ovary cells. alpha-N-Acetylglucosaminidase (EC 3.2.1.50) is a lysosomal enzyme that is deficient in the genetic disorder Sanfilippo syndrome type B. To study the human enzyme, we expressed its cDNA in Lec1 mutant Chinese hamster ovary (CHO) cells, which do not synthesize complex oligosaccharides. The enzyme was purified to apparent homogeneity from culture medium by chromatography on concanavalin A-Sepharose, Poros 20-heparin, and aminooctyl-agarose. The purified enzyme migrated as a single band of 83 kDa on SDS-PAGE and as two peaks corresponding to monomeric and dimeric forms on Sephacryl-300. It had an apparent K(m) of 0.22 mM toward 4-methylumbelliferyl-alpha-N-acetylglucosaminide and was competitively inhibited by two potential transition analogs, 2-acetamido-1,2-dideoxynojirimycin (K(i) = 0.45 microM) and 6-acetamido-6-deoxycastanospermine (K(i) = 0.087 microM). Activity was also inhibited by mercurials but not by N-ethylmaleimide or iodoacetamide, suggesting the presence of essential sulfhydryl residues that are buried. The purified enzyme preparation corrected the abnormal [(35)S]glycosaminoglycan catabolism of Sanfilippo B fibroblasts in a mannose 6-phosphate-inhibitable manner, but its effectiveness was surprisingly low. Metabolic labeling experiments showed that the recombinant alpha-N-acetylglucosaminidase secreted by CHO cells had only a trace of mannose 6-phosphate, probably derived from contaminating endogenous CHO enzyme. This contrasts with the presence of mannose 6-phosphate on naturally occurring alpha-N-acetylglucosaminidase secreted by diploid human fibroblasts and on recombinant human alpha-l-iduronidase secreted by the same CHO cells. Thus contrary to current belief, overexpressing CHO cells do not necessarily secrete recombinant lysosomal enzyme with the mannose 6-phosphate-targeting signal; this finding has implications for the preparation of such enzymes for therapeutic purposes.

519 ||| 21329680 ||| (3, 0.54) ||| 3 ||| 3 ||| Human and rodent aldo-keto reductases from the AKR1B subfamily and their specificity with retinaldehyde. NADP(H)-dependent cytosolic aldo-keto reductases (AKR) are mostly monomeric enzymes which fold into a typical (alpha/beta)(8)-barrel structure. Substrate specificity and inhibitor selectivity are determined by interaction with residues located in three highly variable loops (A, B, and C). Based on sequence identity, AKR have been grouped into families, namely AKR1-AKR15, containing multiple subfamilies. Two human enzymes from the AKR1B subfamily (AKR1B1 and AKR1B10) are of special interest. AKR1B1 (aldose reductase) is related to secondary diabetic complications, while AKR1B10 is induced in cancer cells and is highly active with all-trans-retinaldehyde. Residues interacting with all-trans-retinaldehyde and differing between AKR1B1 and AKR1B10 are Leu125Lys and Val131Ala (loop A), Leu301Val, Ser303Gln, and Cys304Ser (loop C). Recently, we demonstrated the importance of Lys125 as a determinant of AKR1B10 specificity for retinoids. Residues 301 and 304 are also involved in interactions with substrates or inhibitors, and thus we checked their contribution to retinoid specificity. We also extended our study with retinoids to rodent members of the AKR1B subfamily: AKR1B3 (aldose reductase), AKR1B7 (mouse vas deferens protein), AKR1B8 (fibroblast-growth factor 1-regulated protein), and AKR1B9 (Chinese hamster ovary reductase), which were tested against all-trans isomers of retinaldehyde and retinol. All enzymes were active with retinaldehyde, but with k(cat) values (0.02-0.52 min(-1)) much lower than that of AKR1B10 (27 min(-1)). None of the enzymes showed oxidizing activity with retinol. Since these enzymes (except AKR1B3) have Lys125, other residues should account for retinaldehyde specificity. Here, by using site-directed mutagenesis and molecular modeling, we further delineate the contribution of residues 301 and 304. We demonstrate that besides Lys125, Ser304 is a major structural determinant for all-trans-retinaldehyde specificity of AKR1B10.

520 ||| 11027166 ||| (3, 0.54) ||| 3 ||| 3 ||| Effect of ammonia on the glycosylation of human recombinant erythropoietin in culture. Recombinant human erythropoietin (EPO) was produced by a stable transfected CHO-K1 cell clone (EPO-81) grown in serum-free medium. Our previous work showed that there was a significant increase in the heterogeneity of the glycoforms of EPO and a reduction of the sialylation at 20 mM NH(4)Cl. In the work presented here, the effects of ammonia on EPO N-linked oligosaccharides were analyzed. EPO was purified from culture supernatants by immunoaffinity chromatography. The N-linked oligosaccharides were released enzymatically and analyzed by fluorophore-assisted carbohydrate electrophoresis (FACE) and HPLC. The FACE N-linked oligosaccharide profile showed that the sialylated glycans contain one prominent band at a position corresponding to eight glucose units. The density of the major band was greatly diminished and the width was significantly increased in cultures containing added ammonia. The proportion of tetraantennary structures was reduced by 60%, while the tri- and biantennary structures were increased proportionally in the presence of ammonia. Glycan analysis by HPLC using a weak anion exchange column showed that the most significant characteristic effect of ammonia was a reduction of the proportion of glycans with four sialic acids from 46% in control cultures to 29% in ammonia-treated cultures. Analysis of the desialylated glycans by normal phase chromatography indicated a distribution of tetra-, tri-, and biantennary structures similar to that shown by FACE. The N-linked glycan sequence was determined by sequential exoglycosidase digestion followed by FACE. The results indicated a typical N-linked complex oligosaccharide structure. Glycans from ammonia-containing cultures showed the same sequence pattern. In conclusion, we showed that ammonia in the culture medium affected EPO glycosylation, which was observed as a reduction of the tetraantennary and tetrasialylated oligosaccharide structures. However, the presence of ammonia in the cultures did not change the oligosaccharide sequence.

521 ||| 23475710 ||| (3, 0.53) ||| 3 ||| 3 ||| Engineering of therapeutic and diagnostic O-glycans on recombinant mucin-type immunoglobulin fusion proteins expressed in CHO cells. Metabolic engineering of mammalian cells for optimized glycosylation is usually done to improve activity and the pharmacokinetic features of glycoprotein therapeutics. The field is mainly focused around engineering of N-glycans. We have created a platform in which recombinant mucin-type immunoglobulin fusion proteins are used as scaffolds for multivalent expression of O-glycans with diagnostic or therapeutic potential. The methods used to make stable CHO cell lines secreting a mucin-type fusion protein with blood group A or B determinants following expression of up to five different cDNAs are described.

522 ||| 20639190 ||| (3, 0.53) ||| 3 ||| 3 ||| Production of non-fucosylated antibodies by co-expression of heterologous GDP-6-deoxy-D-lyxo-4-hexulose reductase. All IgG-type antibodies are N-glycosylated in their Fc part at Asn-297. Typically, a fucose residue is attached to the first N-acetylglucosamine of these complex-type N-glycans. Antibodies lacking core fucosylation show a significantly enhanced antibody-dependent cell-mediated cytotoxicity (ADCC) and an increased efficacy of anti-tumor activity. In cases where the clinical efficacy of an antibody is to some extent mediated by its ADCC effector function, afucosylated N-glycans could help to reduce dose requirement and save manufacturing costs. Using Chinese hamster ovary (CHO) cells as a model, we demonstrate here that heterologous expression of the prokaryotic enzyme GDP-6-deoxy-d-lyxo-4-hexulose reductase within the cytosol can efficiently deflect the fucose de novo pathway. Antibody-producing CHO cells that were modified in this way secrete antibodies lacking core fucose as demonstrated by MALDI-TOF mass spectrometry and HPAEC-PAD monosaccharide analysis. Engineering of the fucose de novo pathway has led to the construction of IgGs with a strongly enhanced ADCC effector function. The method described here should have broad practical applicability for the development of next-generation therapeutic antibodies.

523 ||| 19297726 ||| (3, 0.53) ||| 3 ||| 3 ||| Application of aqueous two-phase systems to antibody purification: a multi-stage approach. The single-stage equilibrium aqueous two-phase extraction (ATPE) of human antibodies from a Chinese hamster ovary (CHO) cells supernatant was investigated. The performance of polyethylene glycol 3350 (PEG 3350)/phosphate aqueous two-phase systems (ATPS) was evaluated by studying several experimental conditions, such as pH, ionic strength, volume ratio and initial antibody concentration in the feed stock. The conditions that favoured the extraction of human immunoglobulin G (IgG) were low pH values, high NaCl concentration and high volume ratios. The percentage of contaminants removal did not depend on the pH value and NaCl concentration, increasing, however, as the volume ratio decreased. About 66% of total contaminants were removed when a volume ratio of 0.4 was used. The multi-stage equilibrium ATPE was also investigated by simulating a four stages cross-current operation in test tubes. According to the IgG equilibrium curves and respective McCabe Thiele diagrams, a predicted optimised scheme of a counter-current multi-stage ATPE was described. An IgG recovery yield of 89% and a protein purity of 75% can be achieved using a PEG/phosphate ATPS containing 10% (w/w) NaCl, five stages and a volume ratio of 0.4. This represents significant improvements in the recovery yield and purity when compared to a single-stage trial performed at the same experimental conditions, where a 61% recovery yield and 55% protein purity were attained. Based on the CHO cells supernatant components equilibrium curves, it was observed that IgG can be completely purified from the higher molecular weight (MW) components and partially purified from the lower MW components.

524 ||| 11707585 ||| (3, 0.53) ||| 3 ||| 3 ||| Fringe modulation of Jagged1-induced Notch signaling requires the action of beta 4galactosyltransferase-1. Fringe modulates Notch signaling resulting in the establishment of compartmental boundaries in developing organisms. Fringe is a beta 3N-acetylglucosaminyltransferase (beta 3GlcNAcT) that transfers GlcNAc to O-fucose in epidermal growth factor-like repeats of Notch. Here we use five different Chinese hamster ovary cell glycosylation mutants to identify a key aspect of the mechanism of fringe action. Although the beta 3GlcNAcT activity of manic or lunatic fringe is shown to be necessary for inhibition of Jagged1-induced Notch signaling in a coculture assay, it is not sufficient. Fringe fails to inhibit Notch signaling if the disaccharide generated by fringe action, GlcNAc beta 3Fuc, is not elongated. The trisaccharide, Gal beta 4GlcNAc beta 3Fuc, is the minimal O-fucose glycan to support fringe modulation of Notch signaling. Of six beta 4galactosyltransferases (beta 4GalT) in Chinese hamster ovary cells, only beta 4GalT-1 is required to add Gal to GlcNAc beta 3Fuc, identifying beta 4GalT-1 as a new modulator of Notch signaling.

525 ||| 10052355 ||| (3, 0.53) ||| 3 ||| 3 ||| Engineered glycoforms of an antineuroblastoma IgG1 with optimized antibody-dependent cellular cytotoxic activity. The glycosylation pattern of chCE7, an antineuroblastoma chimeric IgG1, was engineered in Chinese hamster ovary cells with tetracycline-regulated expression of beta(1,4)-N-acetylglucosaminyltransferase III (GnTIII), a glycosyltransferase catalyzing formation of bisected oligosaccharides that have been implicated in antibody-dependent cellular cytotoxicity (ADCC). Measurement of the ADCC activity of chCE7 produced at different tetracycline levels showed an optimal range of GnTIII expression for maximal chCE7 in vitro ADCC activity, and this activity correlated with the level of constant region-associated, bisected complex oligosaccharides determined by matrix-assisted laser desorption/ionization time-of-flight mass spectrometry. The new optimized variants of chCE7 exhibit substantial ADCC activity and, hence, may be useful for treatment of neuroblastoma. The strategy presented here should be applicable to optimize the ADCC activity of other therapeutic IgGs.

526 ||| 22531450 ||| (3, 0.52) ||| 3 ||| 3 ||| Galactosylation variations in marketed therapeutic antibodies. There are currently ~25 recombinant full-length IgGs (rIgGs) in the market that have been approved by regulatory agencies as biotherapeutics to treat various human diseases. Most of these are based on IgG1k framework and are either chimeric, humanized or human antibodies manufactured using either Chinese hamster ovary (CHO) cells or mouse myeloma cells as the expression system. Because CHO and mouse myeloma cells are mammalian cells, rIgGs produced in these cell lines are typically N-glycosylated at the conserved asparagine (Asn) residues in the CH2 domain of the Fc, which is also the case with serum IgGs. The Fc glycans present in these rIgGs are for the most part complex biantennary oligosaccharides with heterogeneity associated with the presence or the absence of several different terminal sugars. The major Fc glycans of rIgGs contain 0 or 1 or 2 (G0, G1 and G2, respectively) terminal galactose residues as non-reducing termini and their relative proportions may vary depending on the cell culture conditions in which they were expressed. Since glycosylation is strongly associated with antibody effector functions and terminal galactosylation may affect some of those functions, a panel of commercially available therapeutic rIgGs expressed in CHO cells and mouse myeloma cells were examined for their galactosylation patterns. The results suggest that the rIgGs expressed in CHO cells are generally less galactosylated compared to the rIgGs expressed in mouse myeloma cells. Accordingly, rIgGs produced in CHO cells tend to contain higher levels of G0 glycans compared with rIgGs produced in mouse myeloma cell lines. Despite the apparent wide variability in galactose content, adverse events or safety issues have not been associated with specific galactosylation patterns of therapeutic antibodies. Nevertheless, galactosylation may have an effect on the mechanisms of action of some therapeutic antibodies (e.g., effector pathways) and hence further studies to assess effects on product efficacy may be warranted for such antibodies. For antibodies that do not require effector functions for biological activity, however, setting a narrow specification range for galactose content may be unnecessary.

527 ||| 10688334 ||| (3, 0.52) ||| 3 ||| 3 ||| Recombinant human factor X: high yield expression and the role of furin in proteolytic maturation in vivo and in vitro. Factor X/Xa plays a pivotal role in the coagulation cascade and exhibits a therapeutic potential for the treatment of factor X-deficient as well as FVIII and FIX inhibitor patients. This report describes the establishment of Chinese hamster ovary cell clones expressing recombinant human factor X up to 120 microg/mL x day and 78 microg/10(6) cells x day, that is to 100-fold higher levels than reported previously. Although propeptide removal and single chain precursor to light and heavy chain processing as well as vitamin K-dependent gamma-carboxylation became impaired at these expression levels, up to 25% of the recombinant human factor X produced was active. This represents the highest functional activity ever reported for a vitamin K-dependent protein at such an expression level. Expression of recombinant human factor X in Chinese hamster ovary cells lacking the endoprotease Furin revealed that propeptide removal still occurred, whereas single chain precursor to light/heavy chain processing was abolished. This suggests that a protease different from Furin mediates propeptide removal, a unique finding compared with the other vitamin K-dependent coagulation factors. In contrast, exposure of incompletely processed rFX molecules to soluble recombinant Furin in vitro mediated both of these cleavage reactions despite the absence of a typical argP4-xP3-lys/argP2-argP1 Furin cleavage site in the propeptide, indicating relaxed specificity in vitro. Concomitantly with the degree of processing, the functional activity of recombinant human factor X increased. Interestingly, Furin was shown to even perform correct N-terminal proteolytic trimming of FX molecules truncated amino-terminal to the P3 residue in vitro. Depending on the absence or presence of warfarin in the culture media, as well as on the processing state, four distinct recombinant human factor X light chain isoforms were observed and their structure characterized. One of these light chain forms correlated with the functional activity. Finally, the distribution of the individual light chain isoforms suggests that gamma-carboxylation may be a prerequisite for propeptide removal.

528 ||| 10619704 ||| (3, 0.52) ||| 3 ||| 3 ||| The AATPAP sequence is a very efficient signal for O-glycosylation in CHO cells. The peptide signal sequence for protein O-glycosylation is not fully characterized, although a recent in vitro study proposed that the sequence motif, XTPXP, serves as a signal for mucin-type O-glycosylation. Here, we show that the AATPAP sequence acts as an efficient O-glycosylation signal, in vivo. A secreted fibroblast growth factor (secFGF) was used as a model to analyze glycosylation and its effects on the biological activity of FGF. Two constructs encoding [AATPAP]secFGF in which AATPAP was introduced at the N- or C-terminus of secFGF were constructed in an eukaryotic expression vector. [AATPAP]secFGF proteins were then expressed in Chinese hamster ovary (CHO) cells and secreted into the surrounding medium, primarily as modified forms sensitive to sialidase but not to peptide N-glycosidase F. The modifying groups were not seen when the AATPAP sequence was converted to AAAPAP or when [AATPAP]secFGF was expressed in mutant cells incapable of UDP-GalNAc biosynthesis. The results indicate that the modifying groups were mucin-type O-glycans and that the AATPAP served as an efficient O-glycosylation signal sequence. The O-glycosylated forms of [AATPAP]secFGF were as mitogenic toward human vascular endothelial cells as unmodified secFGF, suggesting that introduction of the signal into biologically active polypeptides is a promising approach with which O-glycosylation may be achieved without affecting original activity.

529 ||| 9804772 ||| (3, 0.51) ||| 3 ||| 3 ||| GDP-L-fucose pyrophosphorylase. Purification, cDNA cloning, and properties of the enzyme. The enzyme that catalyzes the formation of GDP-L-fucose from GTP and beta-L-fucose-1-phosphate (i.e. GDP-beta-L-fucose pyrophosphorylase, GFPP) was purified about 560-fold from the cytosolic fraction of pig kidney. At this stage, there were still a number of protein bands on SDS gels, but only the 61-kDa band became specifically labeled with the photoaffinity substrate, azido-GDP-L-[32P]fucose. Several peptides from this 61-kDa band were sequenced and these sequences were used for cloning the gene. The cDNA clone yielded high levels of GFPP activity when expressed in myeloma cells and in a baculovirus system, demonstrating that the 61-kDa band is the authentic GFPP. The porcine tissue with highest specific activity for GFPP was kidney, with lung, liver, and pancreas being somewhat lower. GFPP was also found in Chinese hamster ovary, but not Madin-Darby canine kidney cells. Northern analysis showed the mRNA in human spleen, prostate, testis, ovary, small intestine, and colon. GFPP was stable at 4 (o)C in buffer containing 50 mM sucrose, with little loss of activity over a 9-day period. GTP was the best nucleoside triphosphate substrate but significant activity was also observed with ITP and to a lesser extent with ATP. The enzyme was reasonably specific for beta-L-fucose-1-P, but could also utilize alpha-D-arabinose-1-P to produce GDP-alpha-D-arabinose. The product of the reaction with GTP and alpha-L-fucose-1-P was characterized as GDP-beta-L-fucose by a variety of chemical and chromatographic methods.

530 ||| 9153262 ||| (3, 0.51) ||| 3 ||| 3 ||| Molecular cloning and expression of Chinese hamster ovary cell heparan-sulfate 2-sulfotransferase. Heparan-sulfate 2-sulfotransferase (HS2ST), which catalyzes the transfer of sulfate from 3'-phosphoadenosine 5'-phosphosulfate to L-iduronic acid at position 2 in heparan sulfate, was purified from cultured Chinese hamster ovary (CHO) cells to apparent homogeneity (Kobayashi, M., Habuchi, H., Habuchi, O., Saito, M., and Kimata, K. (1996) J. Biol. Chem. 271, 7645-7653). The internal amino acid sequences were obtained from the peptides after digestion of the purified protein with a combination of endoproteinases. Mixed oligonucleotides based on the peptide sequences were used as primers to obtain a probe fragment by reverse transcriptase-polymerase chain reaction using CHO cell poly(A)+ RNA as template. The clone obtained from a CHO cDNA library by screening with the probe is 2.2 kilobases in size and contains an open reading frame of 1068 bases encoding a new protein composed of 356 amino acid residues. The protein predicts a type II transmembrane topology similar to other Golgi membrane proteins. Messages of 5.0 and 3.0 kilobases were observed in Northern analysis. Evidence that the cDNA clone corresponds to the purified HS2ST protein is as follows. (a) The predicted amino acid sequence contains all five peptides obtained after endoproteinase digestion of the purified protein; (b) the characteristics of the predicted protein fit those of the purified protein in terms of molecular mass, membrane localization, and N-glycosylation; and (c) when the cDNA containing the entire coding sequence of the enzyme in a eukaryotic expression vector was transfected into COS-7 cells, the HS2ST activity increased 2.6-fold over controls, and the FLAG-HS2ST fusion protein purified by affinity chromatography showed the HS2ST activity alone.

531 ||| 25092905 ||| (3, 0.51) ||| 3 ||| 3 ||| The GalNAc-type O-Glycoproteome of CHO cells characterized by the SimpleCell strategy. The Chinese hamster ovary cell (CHO) is the major host cell factory for recombinant production of biological therapeutics primarily because of its "human-like" glycosylation features. CHO is used for production of several O-glycoprotein therapeutics including erythropoietin, coagulation factors, and chimeric receptor IgG1-Fc-fusion proteins, however, some O-glycoproteins are not produced efficiently in CHO. We have previously shown that the capacity for O-glycosylation of proteins can be one limiting parameter for production of active proteins in CHO. Although the capacity of CHO for biosynthesis of glycan structures (glycostructures) on glycoproteins are well established, our knowledge of the capacity of CHO cells for attaching GalNAc-type O-glycans to proteins (glycosites) is minimal. This type of O-glycosylation is one of the most abundant forms of glycosylation, and it is differentially regulated in cells by expression of a subset of homologous polypeptide GalNAc-transferases. Here, we have genetically engineered CHO cells to produce homogeneous truncated O-glycans, so-called SimpleCells, which enabled lectin enrichment of O-glycoproteins and characterization of the O-glycoproteome. We identified 738 O-glycoproteins (1548 O-glycosites) in cell lysates and secretomes providing the first comprehensive insight into the O-glycosylation capacity of CHO (http://glycomics.ku.dk/o-glycoproteome\_db/).

532 ||| 19947664 ||| (3, 0.51) ||| 3 ||| 3 ||| Characterization of oligosaccharides in recombinant tissue plasminogen activator produced in Chinese hamster ovary cells: two decades of analytical technology development. Recombinant tissue plasminogen activator (rt-PA) is a well-characterized glycoprotein with a great deal of published information on its structure, post-translational modifications, and O- and N-glycosylation. Most of the characterization was accomplished in the late 1980s. During the past 2 decades, however, mass spectrometry has made a quantum leap forward offering new capabilities in soft electrospray ionization, speed, resolution, and accuracy of mass measurements. From this point of view, it is worthwhile to revisit the characterization of familiar proteins, such as rt-PA, using the new capabilities of modern analytical technology. In this work, we applied LC-MS with state-of-the-art instrumentation to the characterization of glycoforms of rt-PA. This method takes advantage of accurate mass measurements along with a fast "in-source" voltage switching for the detection of characteristic oxonium ions of saccharides. This method confirmed previously identified glycan structures based on existing knowledge of rt-PA glycans. In addition, we identified two novel glycan structures in rt-PA. A low level of Asn142 N-glycosylation was detected at an atypical Asn-Xaa-Cys consensus motif. It was found to be modified predominantly by biantennary hybrid structures. This N-glycosylation site was confirmed using a recently developed electron-transfer dissociation (ETD) technique. Also using this method, we detected low levels of elongation of fucose-O-Thr61 to di-, tri-, and tetrasaccharides, not previously observed in rt-PA. The results demonstrate that use of state-of-the-art analytical methods can reveal low-level, previously undetected modifications of well-characterized biopharmaceuticals.

533 ||| 19003383 ||| (3, 0.51) ||| 3 ||| 3 ||| Enhanced erythropoietin heterogeneity in a CHO culture is caused by proteolytic degradation and can be eliminated by a high glutamine level. The molecular heterogeneity of recombinant humanerythropoietin (EPO) increased during the course of abatch culture of transfected Chinese hamster ovary(CHO) cells grown in serum-free medium. This wasshown by both an increased molecular weight and pIrange of the isolated EPO at the end of the culture. However, analysis of the N-glycan structures of themolecule by fluorophore-assisted carbohydrateelectrophoresis (FACE) and HPLC anion exchangechromatography indicated a consistent pattern ofglycosylation. Seven glycoforms were identified, thepredominant structure being a fully sialylatedtetra-antennary glycan. The degree of sialylationwas maintained throughout the culture. Analysis ofthe secreted EPO indicated a time-dependent increasein the molecular weight band width of the peptideconsistent with proteolytic degradation. A highglutamine concentration (16-20 mM) in the culturedecreased the apparent degradation of the EPO.

534 ||| 10910973 ||| (3, 0.51) ||| 3 ||| 3 ||| Exploring the outcome of genetic modifications of glycosylation in cultured cell lines by concurrent isolation of the major classes of vertebrate glycans. In the preceding article (Manzi,A.E., Norgard-Sumnicht,K., Argade,S., Marth,J.D., van Halbeek,H. and Varki.A. [2000] GLYCOBIOLOGY:, 10, 669-688), we reported a comprehensive approach for the extraction, fractionation, and isolation of all of the major classes of sugar chains (glycans) from vertebrate tissues. Here we apply this "Glycan Isolation Protocol" to a variety of cultured mammalian cell lines, including two wild-type Chinese hamster ovary (CHO) cell lines and some of their genetically modified variants that were predicted or known to have defined abnormalities in the biosynthesis of one or more classes of glycans. We also use this approach to characterize clone 489, a new derivative of the GAG-deficient CHO clone pgsA-745, in which sulfation has been restored by transfection of a wild-type CHO cDNA library. By metabolically labeling the cell lines with [6-(3)H]glucosamine we were able to monitor the recovery of all major classes of glycans. The results allow us to reach several conclusions: first, the protocol described in the preceding paper is further validated by finding good recovery of total radioactivity and appropriate distribution of label in the correct glycan classes in the fractions from a variety of cell lines; second, the amount of radioactivity recovered in free glycosylphosphatidylinositol (GPI) lipids is remarkably high when compared to that found in GPI anchors, with the former being the dominant form in some cells; third, cells with known genetic mutations in specific glycosylation pathways are shown to have the expected changes in the distribution of recovered radioactivity in the appropriate fractions; fourth, the N- and O- glycans recovered via the protocol are of adequate quality to demonstrate marked differences in their structural profiles and/or content; fifth, the protocol can pick up unexpected differences of glycan classes not predicted to be affected by the primary defect; finally, the reappearance of sulfation in the novel clone 489 is not due to restoration of GAG sulfation, but rather due to the new expression of sulfation in the fraction enriched in N- and O-linked glycopeptides. These results demonstrate the power of this comprehensive approach for the concurrent exploration and profiling of the different major classes of glycans in cells.

535 ||| 9315609 ||| (3, 0.50) ||| 3 ||| 3 ||| The functional purification of P-glycoprotein is dependent on maintenance of a lipid-protein interface. P-Glycoprotein (P-gp) is a 180-kDa membrane-bound transporter which can confer the multi-drug resistance phenotype on tumor cells. We have examined the factors required to preserve activity of P-gp during its purification. The starting material for purification was plasma membranes from Chinese hamster ovary (CHrB30) cells, overexpressing P-glycoprotein. These membranes displayed drug stimulated ATPase activity (Vm = 897 +/- 55 nmol min(-1) mg(-1); Km = 1.8 +/- 0.4 mM) and high affinity binding of [3H]vinblastine (Kd = 36 +/- 5 nM; Bm = 161 +/- 11 pmol/mg). Several non-ionic detergents which readily solubilized P-glycoprotein significantly inhibited ATPase activity and drug binding at concentrations well below their respective CMC values. This inactivation was prevented by excess crude lipid mixtures, with the greatest protection afforded against dodecyl-maltoside. Furthermore, the significantly reduced binding affinity and capacity of solubilized P-gp was partly reversed by the addition of lipids. A combination of anion-exchange and hydroxyapatite chromatography were used to purify P-gp with high yield to greater than 90%. The purified, reconstituted P-gp displayed high ATPase activity (Vm = 2137 +/- 309; Km = 2.9 +/- 0.9 mM) which was stimulated by verapamil (EC50 = 3.8 +/- 0.6 microM) and inhibited by orthovanadate (3.1 +/- 0.8 microM). Pure P-gp also displayed high affinity vinblastine binding (Kd = 64 +/- 9 nM) with a capacity of 2320 +/- 192 pmol/mg. This purification scheme yields the highest P-gp activity reported to date, and indicates a dependence of function on maintaining a lipid-protein interface.

536 ||| 9083034 ||| (3, 0.50) ||| 3 ||| 3 ||| The interaction between basic fibroblast growth factor and heparan sulfate can prevent the in vitro degradation of the glycosaminoglycan by Chinese hamster ovary cell heparanases. Heparan sulfate proteoglycans on Chinese hamster ovary (CHO) cell surfaces can bind and internalize basic fibroblast growth factor (bFGF). We have investigated whether this interaction affects heparan sulfate catabolism in vitro by measuring the ability of partially purified CHO heparanase activities to degrade 35S-labeled heparan sulfate glycosaminoglycans in the absence or presence of bFGF. Our studies show that the presence of the growth factor prevents partially purified heparanases from degrading the nascent 81-kDa chains to short 6-kDa products, whether the glycosaminoglycan is free in solution or covalently bound to core proteins. A 30-60 molar excess of the growth factor is required to inhibit completely chain degradation by heparanases, implying that multiple bFGF molecules must be bound to the glycosaminoglycan to prevent heparanase-catalyzed catabolism. This hypothesis is supported by protection studies indicating that nascent CHO heparan sulfate glycosaminoglycans have at least four to eight bFGF binding sites/chain. It does not appear, however, that the growth factor inhibits heparanase-catalyzed degradation of the glycosaminoglycan by binding to the sequence cleaved by the enzyme. Both the nascent and short chains bind bFGF with similar affinity (Kd values of 27.0 +/- 3.5 and 38.9 +/- 5.1 nM, respectively), indicating that heparanase activities do not destroy the bFGF binding sites. Rather, our results suggest that the growth factor interferes sterically with heparanase action by binding the heparan sulfate chain at a sequence next to the cleavage site or at a secondary site recognized by the enzyme.

537 ||| 24070904 ||| (3, 0.50) ||| 3 ||| 3 ||| Novel recombinant human lactoferrin: differential activation of oxidative stress related gene expression. Lactoferrin, an iron-binding protein found in high concentrations in mammalian exocrine secretions, is an important component of the host defense system. It is also a major protein of the secondary granules of neutrophils from which is released upon activation. Due to its potential clinical utility, recombinant human lactoferrin (rhLF) has been produced in various eukaryotic expression systems; however, none of these are fully compatible with humans. Most of the biopharmaceuticals approved by the FDA for use in humans are produced in mammalian expression systems. The Chinese hamster ovary cells (CHO) have become the system of choice for proteins that require post-translational modifications, such as glycoproteins. The aim of this study was to scale-up expression and purification of rhLF in a CHO expression system, verify its glycan primary structure, and assess its biological properties in cell culture models. A stable CHO cell line producing >200mg/L of rhLF was developed and established. rhLF was purified by a single-step cation-exchange chromatography procedure. The highly homogenous rhLF has a molecular weight of approximately 80 kDa. MALDI-TOF mass spectrometric analysis revealed N-linked, partially sialylated glycans at two glycosylation sites, typical for human milk LF. This novel rhLF showed a protective effect against oxidative stress in a similar manner to its natural counterpart. In addition, rhLF revealed a modulatory effect on cellular redox via upregulation of key antioxidant enzymes. These data imply that the CHO-derived rhLF is fully compatible with the native molecule, thus it has promise for human therapeutic applications.

538 ||| 23527859 ||| (3, 0.50) ||| 3 ||| 3 ||| Enhanced sialylation and in vivo efficacy of recombinant human alpha-galactosidase through in vitro glycosylation. Human alpha-galactosidase A (GLA) has been used in enzyme replacement therapy for patients with Fabry disease. We expressed recombinant GLA from Chinese hamster ovary cells with very high productivity. When compared to an approved GLA (agalsidase beta), its size and charge were found to be smaller and more neutral. These differences resulted from the lack of terminal sialic acids playing essential roles in the serum half-life and proper tissue targeting. Because a simple sialylation reaction was not enough to increase the sialic acid content, a combined reaction using galactosyltransferase, sialyltransferase, and their sugar substrates at the same time was developed and optimized to reduce the incubation time. The product generated by this reaction had nearly the same size, isoelectric points, and sialic acid content as agalsidase beta. Furthermore, it had better in vivo efficacy to degrade the accumulated globotriaosylceramide in target organs of Fabry mice compared to an unmodified version.

539 ||| 16931047 ||| (3, 0.50) ||| 3 ||| 3 ||| High-level expression and efficient purification of recombinant human long pentraxin PTX3 in Chinese hamster ovary cells. PTX3 is a secreted multimeric glycoprotein which plays a key role in innate immunity by activating the classical complement pathway through specific recognition of the C1q subunit. A method is described for the high level expression of the recombinant human PTX3 in Chinese hamster ovary cells (CHO), adapted to a suspension growth in spinner flasks containing a serum-free chemically defined medium and producing about 50 mg of PTX3/L of culture. A purification procedure to produce a homogeneous protein preparation from the supernatant, by means of anion exchange, hydroxyapatite and size exclusion chromatography, is also reported. This three-step protocol allows us to obtain PTX3 with a recovery yield close to 70%, a purity degree exceeding 95%, and a final host cell protein (HCP) content lower than 150 ppm. The recombinant purified PTX3 retains its biological activity, as demonstrated by C1q binding ELISA assay, and displays a complex quaternary structure characterized by a high secondary structure content quite different from human short pentraxin C-reactive protein (CRP) and serum amyloid P component (SAP), as determined by circular dichroism, fluorescence analysis, and native and SDS-PAGE experiments.

540 ||| 10545921 ||| (3, 0.50) ||| 3 ||| 3 ||| Engineering Chinese hamster ovary cells to maximize sialic acid content of recombinant glycoproteins. We have engineered two Chinese hamster ovary cell lines secreting different recombinant glycoproteins to express high levels of human beta1,4-galactosyltransferase (GT, E.C. 2.4.1.38) and/or alpha2, 3-sialyltransferase (ST, E.C. 2.4.99.6). N-linked oligosaccharide structures synthesized by cells overexpressing the glycosyltransferases showed greater homogeneity compared with control cell lines. When GT was overexpressed, oligosaccharides terminating with GlcNAc were significantly reduced compared with controls, whereas overexpression of ST resulted in sialylation of >/=90% of available branches. As expected, GT overexpression resulted in reduction of oligosaccharides terminating with GlcNAc, whereas overexpression of ST resulted in sialylation of >/=90% of available branches. The more highly sialylated glycoproteins had a significantly longer mean residence time in a rabbit model of pharmacokinetics. These experiments demonstrate the feasibility of genetically engineering cell lines to produce therapeutics with desired glycosylation patterns.

541 ||| 10444843 ||| (3, 0.50) ||| 3 ||| 3 ||| Real-time control of antibody loading during protein A affinity chromatography using an on-line assay. We show that an on-line chromatographic assay can reliably control antibody loading in real-time during protein A affinity chromatography purification of a recombinant antibody from clarified Chinese hamster ovary cell culture fluid. The on-line assay directly sampled preparative column effluent and provided real-time measurement of antibody breakthrough during loading. The on-line assay used protein A immobilized on perfusion chromatography media, equilibrated with phosphate-buffered saline at pH 7.2 and eluted with phosphate-buffered saline at pH 2.2. The assay reliably ended loading at 1% breakthrough with minimal yield loss. Reproducible yield and purity were obtained over 23 consecutive cycles. Yield remained constant while breakthrough capacity and the antibody concentration in the load changed.

542 ||| 24168114 ||| (3, 0.49) ||| 3 ||| 3 ||| Arginine modifications by methylglyoxal: discovery in a recombinant monoclonal antibody and contribution to acidic species. Heterogeneity is common among protein therapeutics. For example, the so-called acidic species (charge variants) are typically observed when recombinant monoclonal antibodies (mAbs) are analyzed by weak-cation exchange chromatography (WCX). Several protein post-translational modifications have been established as contributors but still cannot completely account for all heterogeneity. As reported herein, an unexpected modification by methylglyoxal (MGO) was identified, for the first time, in a recombinant monoclonal antibody expressed in Chinese hamster ovary (CHO) cells. Modifications of arginine residues by methylglyoxal lead to two adducts (dihydroxyimidazolidine and hydroimidazolone) with increases of molecular weights of 72 and 54 Da, respectively. In addition, the modification by methylglyoxal causes the antibody to elute earlier in the weak cation exchange chromatogram. Consequently, the extent to which an antibody was modified at multiple sites corresponds to the degree of shift in elution time. Furthermore, cell culture parameters also affected the extent of modifications by methylglyoxal, a highly reactive metabolite that can be generated from glucose or lipids or other metabolic pathways. Our findings again highlight the impact that cell culture conditions can have on the product quality of recombinant protein pharmaceuticals.

543 ||| 12376727 ||| (3, 0.48),(4, 0.38) ||| 3 ||| 3 ||| Intact Golgi synthesize complex branched O-linked chains on glycoside primers: evidence for the functional continuity of seven glycosyltransferases and three sugar nucleotide transporters. We examined the functional co-localization and continuity of glycosyltransferases and sugar nucleotide transporters in the Golgi of two Chinese hamster ovary (CHO) cell lines that synthesize different types of O-linked oligosaccharides. CHO cells normally synthesize primarily Sia2,3Galbeta1,3GalNAc- on glycoproteins. CHO cells transfected with core-2 GlcNAc transferase (Core 2) can synthesize glycoproteins containing branched O-linked oligosaccharides with poly-N-acetyllactosamines. CHO lines incubated with [(3)H]galactose and GalNAc-alpha-phenyl (GAP) as a primer, synthesize labeled glycoside products that faithfully resemble those found on the endogenous acceptors: CHO cells make Sia2,3[(3)H]Gal(beta)1,3GAP, while CHO Core2 cells synthesize GAPs with complex branched chains including poly-N-acetyllactosamines. To determine if isolated Golgi preparations make similar products, we prepared Golgi by established homogenization methods, documented their intactness, and added tracer UDP-[(3)H]Gal, unlabeled sugar nucleotides, and GAP. CHO Golgi preparations synthesized only Sia2,3[(3)H]Gal(beta)1,3GAP. CHO Core2, also made this product and a small amount of Core-2 GlcNAc transferase-dependent products. No endogenous glycoproteins were labeled. However, when either cell line was gently permeabilized with streptolysin-O or given hypo-osmotic shock, both GAP and endogenous acceptors were efficiently glycosylated within an intact functional Golgi lumen and remained there. Significantly, Golgi from CHO Core2 cells made mostly branched GAP products including some with poly-N-acetyllactosamines as complex as those made and secreted by living cells incubated with GAP. These results suggest that the lumen of the Golgi apparatus is functionally continuous or interconnected. Once glycosides diffuse into the Golgi lumen, they have access to all the sugar nucleotide transporters and glycosyltransferases used for complex GAP-based products without requiring metabolic energy or inter-vesicular transport. Glycosylation of artificial acceptors could be used to track the functional continuity or co-localization of multiple glycosyltransferases and transporters under conditions where Golgi morphology disintegrates and/or reappears.

544 ||| 23078371 ||| (3, 0.48) ||| 3 ||| 3 ||| Effect of pH, temperature, and salt on the stability of Escherichia coli- and Chinese hamster ovary cell-derived IgG1 Fc. The circulation half-life of a potential therapeutic can be increased by fusing the molecule of interest (an active peptide, the extracellular domain of a receptor, an enzyme, etc.) to the Fc fragment of a monoclonal antibody. For the fusion protein to be a successful therapeutic, it must be stable to process and long-term storage conditions, as well as to physiological conditions. The stability of the Fc used is critical for obtaining a successful therapeutic protein. The effects of pH, temperature, and salt on the stabilities of Escherichia coli- and Chinese hamster ovary cell (CHO)-derived IgG1 Fc high-order structure were probed using a variety of biophysical techniques. Fc molecules derived from both E. coli and CHO were compared. The IgG1 Fc molecules from both sources (glycosylated and aglycosylated) are folded at neutral pH and behave similarly upon heat- and low pH-induced unfolding. The unfolding of both IgG1 Fc molecules occurs via a multistep unfolding process, with the tertiary structure and C(H)2 domain unfolding first, followed by changes in the secondary structure and C(H)3 domain. The acid-induced unfolding of IgG1 Fc molecules is only partially reversible, with the formation of high-molecular weight species. The CHO-derived Fc protein (glycosylated) is more compact (smaller hydrodynamic radius) than the E. coli-derived protein (aglycosylated) at neutral pH. Unfolding is dependent on pH and salt concentration. The glycosylated C(H)2 domain melts at a temperature 4-5 C higher than that of the aglycosylated domain, and the low-pH-induced unfolding of the glycosylated Fc molecule occurs at a pH ~0.5 pH unit lower than that of the aglycosylated protein. The difference observed between E. coli- and CHO-derived Fc molecules primarily involves the C(H)2 domain, where the glycosylation of the Fc resides.

545 ||| 16159680 ||| (3, 0.48) ||| 3 ||| 3 ||| Fast and efficient protein purification using membrane adsorber systems. The purification of proteins from complex cell culture samples is an essential step in proteomic research. Traditional chromatographic methods often require several steps resulting in time consuming and costly procedures. In contrast, protein purification via membrane adsorbers offers the advantage of fast and gentle but still effective isolation. In this work, we present a new method for purification of proteins from crude cell extracts via membrane adsorber based devices. This isolation procedure utilises the membranes favourable pore structure allowing high flow rates without causing high back pressure. Therefore, shear stress to fragile structures is avoided. In addition, mass transfer takes place through convection rather than diffusion, thus allowing very rapid separation processes. Based on this membrane adsorber technology the separation of two model proteins, human serum albumin (HSA) and immungluboline G (IgG) is shown. The isolation of human growth hormone (hGH) from chinese hamster ovary (CHO) cell culture supernatant was performed using a cation exchange membrane. The isolation of the enzyme penicillin acylase from the crude Escherichia coli supernatant was achieved using an anion exchange spin column within one step at a considerable purity. In summary, the membrane adsorber devices have proven to be suitable tools for the purification of proteins from different complex cell culture samples.

546 ||| 24318171 ||| (3, 0.47),(8, 0.33) ||| 3 ||| 3 ||| Evaluation of a PEG/hydroxypropyl starch aqueous two-phase system for the separation of monoclonal antibodies from cell culture supernatant. In this study, an aqueous two-phase system (ATPS) with PEG and hydroxypropyl starch (HPS) was used to separate monoclonal antibody (mAb) from Chinese hamster ovary cell culture supernatant. The phase diagram of the PEG/HPS ATPS was determined, and the effects of NaCl addition were investigated. The results showed that NaCl addition could lead to a shift of the binodal curve and that phase separation would occur at higher PEG and HPS concentrations. The effects of NaCl addition, pH, and the load of cell supernatant on the partitioning of mAb in a PEG/HPS ATPS were investigated. It was found that with 6% cell supernatant and 15% NaCl addition at pH 6.0, the yield of mAb in the upper phase was 96.7% with a purity of 96.0%. The back-extraction of mAb with a PEG/phosphate ATPS were also studied, and the results showed that after the two-step extraction with ATPSs the purity of mAb could reach 97.6 +- 0.5% with a yield of 86.8 +- 1.0%, which was comparable to the purification with Protein A chromatography. These results indicate that the two-step extraction with PEG/HPS and PEG/phosphate ATPSs might be a promising alternative for the separation of mAb from cell culture supernatant.

547 ||| 24450425 ||| (3, 0.46),(8, 0.38) ||| 3 ||| 3 ||| Filter-aided N-glycan separation (FANGS): a convenient sample preparation method for mass spectrometric N-glycan profiling. We have developed a simple method for the release and isolation of glycoprotein N-glycans from whole-cell lysates using less than a million cells, for subsequent implementation with mass spectrometric analysis. Cellular protein extracts prepared using SDS solubilization were sequentially treated in a membrane filter device to ultimately release glycans enzymatically using PNGase F in the volatile buffer ammonium bicarbonate. The released glycans are recovered in the filtrate following centrifugation and typically permethylated prior to mass spectrometric analysis. We call our method "filter-aided N-glycan separation" and have successfully applied it to investigate N-glycan profiles of wild-type and mutant Chinese hamster ovary cells. This method is readily multiplexed and, because of the small numbers of cells needed, is compatible with the analysis of replicate samples to assess the true nature of glycan variability in tissue culture samples.

548 ||| 9006797 ||| (3, 0.46),(8, 0.35) ||| 3 ||| 3 ||| Monitoring the glycosylation pattern of recombinant interferon-omega with high-pH anion-exchange chromatography and capillary electrophoresis. The enzymatic glycosylation of certain asparagine residues in the protein structure has a profound influence on their physico-chemical properties. Many recombinant DNA derived glycoprotein pharmaceuticals for therapeutic use are glycosylated. Their oligosaccharides are important with regard to stability, solubility, in vivo activity and antigenicity. Sophisticated analytical methods that allow a high resolution of synthesized carbohydrate structures are therefore necessary to ensure a constant product quality. To elucidate the robustness of interferon-omega glycosylation regarding process modifications. Chinese Hamster Ovary (CHO)-cells expressing human interferon-omega were cultivated under different fermentation conditions. The most significant glycosylation alterations resulted from the varied parameters such as initial ammonia concentration in the production medium, cultivation mode (adherent versus suspended) or process time. These are detectable with HPAEC (high-pH anion-exchange chromatography) oligosaccharide mapping as well as with capillary electrophoresis.

549 ||| 22173005 ||| (3, 0.46),(8, 0.31) ||| 3 ||| 3 ||| Continuous aqueous two-phase extraction of human antibodies using a packed column. The performance of a pilot scale packed differential contactor was evaluated for the continuous counter-current aqueous two-phase extraction (ATPE) of human immunoglobulin G (IgG) from a Chinese hamster ovary (CHO) cells supernatant (CS) enriched with pure protein. Preliminary studies have been firstly performed in order to select the dispersed phase (phosphate-rich or polyethylene glycol 3350 Da (PEG)-rich phase) and the column packing material. The PEG-rich phase has been selected as the dispersed phase and the stainless steel as the preferred material for the column packing bed since it was not wetted preferentially by the selected dispersed phase. Hydrodynamic studies have been also performed, and the experimental results were successfully adjusted to the Richardson-Zaki and Misek equations, typically used for the conventional organic-aqueous two-phase systems. An experimental set-up combining the packed column with a pump mixer-settler stage showed to have the best performance and to be advantageous when compared to the IgG batch extraction. An IgG recovery yield of 85% could be obtained with about 50% of total contaminants and more than 85% of contaminant proteins removal. Mass transfer studies have revealed that the mass transfer was controlled by the PEG-rich phase. A higher efficiency could be obtained when using an extra pump mixer-settler stage and higher flow rates.

550 ||| 10022380 ||| (3, 0.46),(2, 0.40) ||| 3 ||| 3 ||| High-level and effective production of human mannan-binding lectin (MBL) in Chinese hamster ovary (CHO) cells. We have developed a high-expression system of recombinant human mannan-binding lectin (MBL) with CHO cells. Geneticin-resistant transformants harboring human MBL cDNA in the expression vector pNOW/CMV-A were screened by immunoblot analysis for secretion of recombinant MBL. Cloning and selection by both geneticin and methotrexate resulted in the production of recombinant MBL to a final concentration of 128.8 microg/ml in media after four days of culture. SDS-PAGE and gel-filtration analyses showed that recombinant MBL is characterized by two lower-order oligomeric structures (apparent molecular weights: 1150 kDa and 300 kDa) compared to native MBL (apparent molecular weight: 1300 kDa). The recombinant human MBL has both sugar-binding and complement activation activity and, like native MBL, can inhibit hemagglutination of influenza A virus. Lectin blots with recombinant MBL indicate that it can bind such microorganisms as HIV and influenza virus suggesting that it might inhibit their infection of hosts. This high-level expression of human MBL with the full range of biological activity will be useful for studies on the immunological role of MBL in humans.

551 ||| 23532914 ||| (3, 0.45),(8, 0.36) ||| 3 ||| 3 ||| Fast and scalable purification of a therapeutic full-length antibody based on process crystallization. The potential of process crystallization for purification of a therapeutic monoclonal IgG1 antibody was studied. The purified antibody was crystallized in non-agitated micro-batch experiments for the first time. A direct crystallization from clarified CHO cell culture harvest was inhibited by high salt concentrations. The salt concentration of the harvest was reduced by a simple pretreatment step. The crystallization process from pretreated harvest was successfully transferred to stirred tanks and scaled-up from the mL-scale to the 1 L-scale for the first time. The crystallization yield after 24 h was 88-90%. A high purity of 98.5% was reached after a single recrystallization step. A 17-fold host cell protein reduction was achieved and DNA content was reduced below the detection limit. High biological activity of the therapeutic antibody was maintained during the crystallization, dissolving, and recrystallization steps. Crystallization was also performed with impure solutions from intermediate steps of a standard monoclonal antibody purification process. It was shown that process crystallization has a strong potential to replace Protein A chromatography. Fast dissolution of the crystals was possible. Furthermore, it was shown that crystallization can be used as a concentrating step and can replace several ultra-/diafiltration steps. Molecular modeling suggested that a negative electrostatic region with interspersed exposed hydrophobic residues on the Fv domain of this antibody is responsible for the high crystallization propensity. As a result, process crystallization, following the identification of highly crystallizable antibodies using molecular modeling tools, can be recognized as an efficient, scalable, fast, and inexpensive alternative to key steps of a standard purification process for therapeutic antibodies.

552 ||| 11410853 ||| (3, 0.45),(2, 0.35) ||| 3 ||| 3 ||| Expression of GnTIII in a recombinant anti-CD20 CHO production cell line: Expression of antibodies with altered glycoforms leads to an increase in ADCC through higher affinity for FC gamma RIII. The gene encoding the rat glycosylation enzyme beta1-4-N-acetylglucosaminyltransferase III (GnTIII) was cloned and coexpressed in a recombinant production Chinese hamster ovary (CHO) cell line expressing a chimeric mouse/human anti-CD20 IgG1 antibody. The new cell lines expressed high levels of antibody and have growth kinetics similar to that of the parent. Relative QPCR showed the cell lines to express varying levels of mRNA. High-performance liquid chromatography (HPLC) analysis showed the enzyme to have added bisecting N-acetylglucosamine (GlcNAc) residues in most (48% to 71%) of the N-linked oligosaccharides isolated from antibody preparations purified from the cell lines. In an ADCC assay the new antibody preparations promoted killing of CD20-positive target cells at approximately 10- to 20-fold lower concentrations than the parent. This activity was blocked using an anti-Fc gamma RIII antibody, supporting the role of Fc gamma RIII binding in this increase. In addition, cell binding assays showed the modified antibody bound better to Fc gamma RIII-expressing cells. The increase in ADCC activity is therefore likely due to an increased affinity of the modified antibody for the Fc gamma RIII receptor.

553 ||| 9535912 ||| (3, 0.45),(2, 0.31) ||| 3 ||| 3 ||| Molecular characterization and expression of heparan-sulfate 6-sulfotransferase. Complete cDNA cloning in human and partial cloning in Chinese hamster ovary cells. Heparan-sulfate 6-sulfotransferase (HS6ST) catalyzes the transfer of sulfate from 3'-phosphoadenosine 5'-phosphosulfate to position 6 of the N-sulfoglucosamine residue of heparan sulfate. The enzyme was purified to apparent homogeneity from the serum-free culture medium of Chinese hamster ovary (CHO) cells (Habuchi, H., Habuchi, O., and Kimata, K. (1995) J. Biol Chem. 270, 4172-4179). From the amino acid sequence data of the purified enzyme, degenerate oligonucleotides were designed and used as primers for the reverse transcriptase-polymerase chain reaction using poly(A)+ RNA from CHO cells as a template. The amplified cDNA fragment was then used as a probe to screen a cDNA library of CHO cells. The cDNA clone thus obtained encoded a partial peptide sequence composed of 236 amino acid residues that included the sequences of six peptides obtained after endoproteinase digestion of the purified enzyme. This cDNA clone was applied to the screening of a human fetal brain cDNA library by cross-hybridization. The isolated cDNA clones contained a whole open reading frame that predicts a type II transmembrane protein composed of 401 amino acid residues. No significant amino acid sequence identity to any other proteins, including heparan-sulfate 2-sulfotransferases, was observed. When the cDNA for the entire coding sequence of the protein was inserted into a eukaryotic expression vector and transfected into COS-7 cells, the HS6ST activity increased 7-fold over the control. The FLAG fusion protein purified by anti-FLAG affinity chromatography showed the HS6ST activity alone. Northern blot analysis revealed the occurrence of a single transcript of 3.9 kilobases in both human fetal brain and CHO cells. The results, together with the ones from our recent cDNA analysis of heparan-sulfate 2-sulfotransferase (Kobayashi, M., Habuchi, H., Yoneda, M., Habuchi, O., and Kimata, K. (1997) J. Biol. Chem. 272, 13980-13985), suggest that at least two different gene products are responsible for 6- and 2-O-sulfations of heparan sulfate.

554 ||| 10686152 ||| (3, 0.45),(2, 0.31) ||| 3 ||| 3 ||| Expression, purification, and characterization of human recombinant thrombopoietin in Chinese hamster ovary cells. Thrombopoietin (TPO) is a primary regulator of megakaryocytopoiesis, a process through which megakaryocytes proliferate and mature into platelets. Recombinant human TPO (rhTPO) was expressed in Chinese hamster ovary (CHO) cells and purified from the culture medium. The cDNA encoding full-length TPO, including the native signal peptide sequence, was amplified by PCR from a human fetal liver cDNA library. The product was cloned into a mammalian expression vector under the control of the SV40 early promoter and enhancer. Secreted rhTPO was purified in three conventional chromatography steps. It migrates on SDS-PAGE as a broad band, characteristic of a heavily glycosylated protein, with an average molecular mass of 85 kDa. rhTPO expressed in CHO cells is biologically active in vitro as demonstrated by its ability to stimulate the proliferation of a megakaryocytic cell line and to trigger the JAK/STAT signal transduction pathway. rhTPO also shows activity in vivo as judged by the elevation of platelet count in treated mice.

555 ||| 23229953 ||| (3, 0.45) ||| 3 ||| 3 ||| Continuous purification of antibodies from cell culture supernatant with aqueous two-phase systems: from concept to process. An aqueous two-phase extraction (ATPE) process based on a PEG/phosphate system was developed for the capture of human immunoglobulin G and successfully applied to a Chinese hamster ovary and a PER.C6 cell supernatant. A continuous ATPE process incorporating three different steps (extraction, back-extraction, and washing) was set up and validated in a pump mixer-settler battery. Most of the higher molecular weight cell supernatant impurities were removed during the extraction step, while most of the lower molecular weight impurities were removed during the subsequent steps. A global recovery yield of 80% and a final protein purity of more than 99% were obtained for the IgG purification from a CHO cell supernatant, representing a 155-fold reduction in the protein/IgG ratio. For the purification of IgG from a PER.C6 cell supernatant, a global recovery yield of 100%, and a host cell protein purity were attained, representing a 22-fold reduction in the host cell protein/IgG ratio. These results, thus, open promising perspectives for the application of the developed ATPE process as a platform for the capture of antibodies. In fact, this new process has shown the ability to successfully recover and purify different antibodies from distinct cell culture supernatants. This technology can also overcome some of the limitations encountered using the typical chromatographic processes, besides inherent advantages of scalability, process integration, capability of continuous operation, and economic feasibility.

556 ||| 16523522 ||| (3, 0.44),(6, 0.32) ||| 3 ||| 3 ||| The effect of dissolved oxygen on the production and the glycosylation profile of recombinant human erythropoietin produced from CHO cells. Human recombinant erythropoietin (rHuEPO) was produced from Chinese hamster ovary (CHO) cells transfected with the human EPO gene. The cells were grown in batch cultures in controlled bioreactors in which the set-points for dissolved oxygen varied between 3% and 200%. The cell-specific growth rate and final cell yield was significantly lower under hyperoxic conditions (200% DO). However, there was no significant difference in growth rates at other oxygen levels compared to control cultures run under a normoxic condition (50% DO). The specific productivity of EPO was significantly lower at a DO set-point of 3% and 200% but maintained a consistently high value between 10% to 100% DO. The EPO produced under all conditions as analyzed by two-dimensional electrophoresis showed a molecular weight range of 33 to 37 kDa and a low isoelectric point range of 3.5 to 5.0. This corresponds to a highly glycosylated and sialylated protein with a profile showing at least seven distinct isoforms. The glycan pattern of isolated samples of EPO was analyzed by weak anion exchange (WAX) HPLC and by normal-phase HPLC incorporating sequential digestion with exoglycosidase arrays. Assigned structures were confirmed by mass spectrometry (MALDI-MS). The most prominent glycan structures were core fucosylated tetranntenary with variable sialylation. However, significant biantennary, triantennary, and non-fucosylated glycans were also identified. Detailed analysis of these glycan structures produced under variable dissolved oxygen levels did not show consistently significant variations except for the ratio of fucosylated to non-fucosylated isoforms. Maximum core fucosylation (80%) was observed at 50% and 100% DO, whereas higher or lower DO levels resulted in reduced fucosylation. This observation of lower fucosylation at high or low DO levels is consistent with previous data reported for glycoprotein production in insect cells.

557 ||| 9276682 ||| (3, 0.44),(6, 0.30) ||| 3 ||| 3 ||| Structures of the sugar chains of recombinant macrophage colony-stimulating factor produced in Chinese hamster ovary cells. The structures of the N- and O-linked sugar chains of recombinant human macrophage colony-stimulating factor (rhM-CSF) from Chinese hamster ovary (CHO) cells were studied. rhM-CSF is a homodimeric glycoprotein. Sugar composition analysis revealed that rhM-CSF contained 4.1 mol N-acetylgalactosamine, 10.3 mol N-acetylglucosamine, 5.0 mol mannose, 10.0 mol galactose, 1.4 mol fucose, and 11.8 mol sialic acid per mol of the monomer. The N- and O-linked sugar chains liberated by hydrazinolysis were N-acetylated, and the reducing-end sugar residues were tagged with 2-aminopyridine. The pyridylamino (PA-) sugar chains thus obtained were purified by HPLC. The structures of the PA-sugar chains were analyzed by a combination of reversed-phase and size-fractionation HPLC, and exoglycosidase digestions, from which the structures of the rhM-CSF sugar chains were estimated to be as follows: monosialo biantennary sugar chain (9 mol%), monosialo fucosylbiantennary sugar chain (10 mol%), disialo biantennary sugar chain (30 mol%), disialo fucosylbiantennary sugar chain (28 mol%), disialo triantennary sugar chain (7 mol%), trisialo triantennary sugar chain (11 mol%), and trisialo fucosyltriantennary sugar chain (5 mol%) for the N-linked sugar chains, and asialo (27 mol%), monosialo (51 mol%), and disialo (22 mol%) Galbeta1-3GalNAc for the O-linked sugar chains. Sialic acid residues were linked to the N-linked sugar chains through an alpha2-3 linkage.

558 ||| 22692203 ||| (3, 0.44),(4, 0.43) ||| 3 ||| 3 ||| Metabolism of vertebrate amino sugars with N-glycolyl groups: incorporation of N-glycolylhexosamines into mammalian glycans by feeding N-glycolylgalactosamine. The outermost positions of mammalian cell-surface glycans are predominantly occupied by the sialic acids N-acetylneuraminic acid (Neu5Ac) and N-glycolylneuraminic acid (Neu5Gc). To date, hydroxylation of CMP-Neu5Ac resulting in the conversion into CMP-Neu5Gc is the only known enzymatic reaction in mammals to synthesize a monosaccharide carrying an N-glycolyl group. In our accompanying paper (Bergfeld, A. K., Pearce, O. M., Diaz, S. L., Pham, T., and Varki, A. (2012) J. Biol. Chem. 287, jbc.M112.363549), we report a metabolic pathway for degradation of Neu5Gc, demonstrating that N-acetylhexosamine pathways are tolerant toward the N-glycolyl substituent of Neu5Gc breakdown products. In this study, we show that exogenously added N-glycolylgalactosamine (GalNGc) serves as a precursor for Neu5Gc de novo biosynthesis, potentially involving seven distinct mammalian enzymes. Following the GalNAc salvage pathway, UDP-GalNGc is epimerized to UDP-GlcNGc, which might compete with the endogenous UDP-GlcNAc for the sialic acid biosynthetic pathway. Using UDP-N-acetylglucosamine 2-epimerase/N-acetylmannosamine kinase-deficient cells, we confirm that conversion of GalNGc into Neu5Gc depends on this key enzyme of sialic acid biosynthesis. Furthermore, we demonstrate by mass spectrometry that the metabolic intermediates UDP-GalNGc and UDP-GlcNGc serve as substrates for assembly of most major classes of cellular glycans. We show for the first time incorporation of GalNGc and GlcNGc into chondroitin/dermatan sulfates and heparan sulfates, respectively. As demonstrated by structural analysis, N-glycolylated hexosamines were found in cellular gangliosides and incorporated into Chinese hamster ovary cell O-glycans. Remarkably, GalNAc derivatives altered the overall O-glycosylation pattern as indicated by the occurrence of novel O-glycan structures. This study demonstrates that mammalian N-acetylhexosamine pathways and glycan assembly are surprisingly tolerant toward the N-glycolyl substituent.

559 ||| 10397812 ||| (3, 0.44) ||| 3 ||| 3 ||| Constraints on the transport and glycosylation of recombinant IFN-gamma in Chinese hamster ovary and insect cells. In this study we compare intracellular transport and processing of a recombinant glycoprotein in mammalian and insect cells. Detailed analysis of the N-glycosylation of recombinant human IFN-gamma by matrix-assisted laser-desorption mass spectrometry showed that the protein secreted by Chinese hamster ovary and baculovirus-infected insect Sf9 cells was associated with complex sialylated or truncated tri-mannosyl core glycans, respectively. However, the intracellular proteins were predominantly associated with high-mannose type oligosaccharides (Man-6 to Man-9) in both cases, indicating that endoplasmic reticulum to cis-Golgi transport is a predominant rate-limiting step in both expression systems. In CHO cells, although there was a minor intracellular subpopulation of sialylated IFN-gamma glycoforms identical to the secreted product (therefore associated with late-Golgi compartments or secretory vesicles), no other intermediates were evident. Therefore, anterograde transport processes in the Golgi stack do not limit secretion. In Sf9 insect cells, there was no direct evidence of post-ER glycan-processing events other than core fucosylation and de-mannosylation, both of which were glycosylation site-specific. To investigate the influence of nucleotide-sugar availability on cell-specific glycosylation, the cellular content of nucleotide-sugar substrates in both mammalian and insect cells was quantitatively determined by anion-exchange HPLC. In both host cell types, UDP-hexose and UDP-N-acetylhexosamine were in greater abundance relative to other substrates. However, unlike CHO cells, sialyltransferase activity and CMP-NeuAc substrate were not present in uninfected or baculovirus-infected Sf9 cells. Similar data were obtained for other insect cell hosts, Sf21 and Ea4. We conclude that although the limitations on intracellular transport and secretion of recombinant proteins in mammalian and insect cells are similar, N-glycan processing in Sf insect cells is limited, and that genetic modification of N-glycan processing in these insect cell lines will be constrained by substrate availability to terminal galactosylation.

560 ||| 23890547 ||| (3, 0.43),(8, 0.41) ||| 3 ||| 3 ||| Isolation of monoclonal antibody from a Chinese hamster ovary supernatant. I: assessment of different separation concepts. The performance of different non-affinity purification techniques commonly used for isolating CHO derived monoclonal antibodies has been investigated. Ion exchange chromatography (IEC), hydrophobic interaction chromatography (HIC), aqueous-two-phase extraction (ATPE) and their integration has been evaluated in terms of yield and purity of the product obtained. The integration of chromatographic techniques comprised two steps, in which the CHO supernatant was directly injected into the IEC column to capture monoclonal IgG1 and then the isolated fraction was processed using the HIC column. To reduce the influence of the feed media on retention of the target protein, the feed mixture was on-column diluted by use of the multi-injection technique. In the coupled process of extraction and chromatography the ATPE operation was used for the pre-purification of the supernatant as well as for buffer exchange. The bottom ATPE phase containing the target protein was further purified on the HIC column without feed dilution. The influence of operating conditions on the effectiveness of different purification processes has been evaluated. The best performance with respect to the product purity was achieved for the coupled process of IEC and HIC. The experimental data acquired were exploited in subsequent investigations for determining underlying kinetic parameters and for the process prediction and optimization.

561 ||| 18465215 ||| (3, 0.43),(2, 0.38) ||| 3 ||| 3 ||| High-level expression and purification of a designed angiopoietin-1 chimeric protein, COMP-Ang1, produced in Chinese hamster ovary cells. A designed angiopoietin-1 (Ang1) chimeric protein with nonleaky angiogenic activity, COMP-Ang1, is an effective alternative to native Ang1 for therapeutic angiogenesis in vivo. Recombinant Chinese hamster ovary (rCHO) cell lines expressing a high level (>20 microg/mL) of COMP-Ang1 and an amino-terminal FLAG-tag were constructed by transfecting the expression vector into dihydrofolate reductase-deficient CHO cells and the subsequent gene amplification in medium containing stepwise increments in methotrexate level such as 0.02, 0.08, 0.32, and 1 microM. The COMP-Ang1 secreted from rCHO cells was purified at a purification yield of 40.3% from the culture medium using an anti-FLAG M2 agarose affinity gel. SDS-PAGE and Western blot analyses showed that rCHO cells secrete COMP-Ang1 in homopentameric and homotetrameric glycoprotein forms. Furthermore, COMP-Ang1 binds to the Tie2 receptor and phosphorylates Tie2, indicating its potential for therapeutic angiogenesis.

562 ||| 9548545 ||| (3, 0.42),(4, 0.37) ||| 3 ||| 3 ||| Micropatterned immobilization of epidermal growth factor to regulate cell function. Photoreactive epidermal growth factor (EGF) was synthesized by conjugating mouse EGF with photoreactive polyallylamine, which was synthesized by the coupling reaction of polyallylamine with N-[4-(azidobenzoyl)oxy]succinimide. The EGF derivative was pattern-immobilized onto a polystyrene plate by UV irradiation in the presence of a photomask in a prescribed micropattern. The patterned immobilization of EGF on the polystyrene plate was confirmed by immunostaining with anti-EGF antibody. Chinese hamster ovary (CHO) cells overexpressing EGF receptors were cultured on the micropatterned plate. The phosphorylated tyrosine residues of signal proteins, including EGF receptors, were detected only in the cells adhered in the EGF-immobilized area, and cell growth was observed only in the EGF-immobilized area. The cells growing in the EGF-immobilized area were partially stained by anti-phosphotyrosine antibody, when the area of EGF immobilization was smaller than the cell. The partial staining of activated proteins indicates that immobilization of EGF inhibited the free lateral diffusion and internalization of the activated EGF-EGF receptor complex. The enhanced cell growth is due to juxtacrine stimulation realized by immobilized EGF.

563 ||| 18623533 ||| (3, 0.41),(6, 0.32) ||| 3 ||| 3 ||| N-glycans of recombinant human interferon-gamma change during batch culture of chinese hamster ovary cells. A recombinant Chinese hamster ovary (CHO) cell line making human interfron-gamma (IFN-gamma) was grown in 12-L stirred tank fermentors in three batch fermentations under conditions of constant temperature, pH, and dissolved oxygen tension. In addition to cell growth, metabolite, and productivity data, a detailed analysis of the carbohydrate structures attached to each glycosylation site of IFN-gamma was achieved using matrix-assisted laser desorption mass spectrometry (MALDI-MS) in combination with exoglycosidase array sequencing. Complex biantennary oligosaccharides (particularly Gal(2)GlcNAc(4)Man(3) which was core alephl-6 fucosylated at Asn(25) but not at Asng(97)) were most prevalent at both glycosylation sites. However, considerable microheterogeneity arising from the presence of triantennary and truncated glycan structures was also observed. The proportion of the dominant core glycan structure (Gal(2)GlcNAc(4)Man(3) +/- Fuc(1)) decreased by 15-26% during batch culture, with increases in the proportion of oligomannose and truncated glycans over the same time period. Prolonged culture resulting from an extended lag phase led to further accumulation of oligomannose and truncated structures, reaching up to 52% of total glycans attached to Asng(97) by 240 h of culture. The implications of these glycosylation changes for optimizing the time for harvesting cell cultures, and for the clearance of recombinant therapeutic products in vivo are discussed. (c) 1995 John Wiley & Sons, Inc.

564 ||| 10224052 ||| (3, 0.41),(4, 0.41) ||| 3 ||| 3 ||| Chinese hamster ovary cell mutants defective in glycosaminoglycan assembly and glucuronosyltransferase I. The proteoglycans of animal cells typically contain one or more heparan sulfate or chondroitin sulfate chains. These glycosaminoglycans assemble on a tetrasaccharide primer, -GlcAbeta1, 3Galbeta1,3Galbeta1,4Xylbeta-O-, attached to specific serine residues in the core protein. Studies of Chinese hamster ovary cell mutants defective in the first or second enzymes of the pathway (xylosyltransferase and galactosyltransferase I) show that the assembly of the primer occurs by sequential transfer of single monosaccharide residues from the corresponding high energy nucleotide sugar donor to the non-reducing end of the growing chain. In order to study the other reactions involved in linkage tetrasaccharide assembly, we have devised a powerful selection method based on induced resistance to a mitotoxin composed of basic fibroblast growth factor-saporin. One class of mutants does not incorporate 35SO4 and [6-3H]GlcN into glycosaminoglycan chains. Incubation of these cells with naphthol-beta-D-xyloside (Xylbeta-O-Np) resulted in accumulation of linkage region intermediates containing 1 or 2 mol of galactose (Galbeta1, 4Xylbeta-O-Np and Galbeta1, 3Galbeta1, 4Xylbeta-O-Np) and sialic acid (Siaalpha2,3Galbeta1, 3Galbeta1, 4Xylbeta-O-Np) but not any GlcA-containing oligosaccharides. Extracts of the mutants completely lacked UDP-glucuronic acid:Galbeta1,3Gal-R glucuronosyltransferase (GlcAT-I) activity, as measured by the transfer of GlcA from UDP-GlcA to Galbeta1,3Galbeta-O-naphthalenemethanol (<0.2 versus 3.6 pmol/min/mg). The mutation most likely lies in the structural gene encoding GlcAT-I since transfection of the mutant with a cDNA for GlcAT-I completely restored enzyme activity and glycosaminoglycan synthesis. These findings suggest that a single GlcAT effects the biosynthesis of common linkage region of both heparan sulfate and chondroitin sulfate in Chinese hamster ovary cells.

565 ||| 11278604 ||| (3, 0.41),(4, 0.35) ||| 3 ||| 3 ||| Chinese hamster ovary (CHO) cells may express six beta 4-galactosyltransferases (beta 4GalTs). Consequences of the loss of functional beta 4GalT-1, beta 4GalT-6, or both in CHO glycosylation mutants. Six beta4-galactosyltransferase (beta4GalT) genes have been cloned from mammalian sources. We show that all six genes are expressed in the Gat(-)2 line of Chinese hamster ovary cells (Gat(-)2 CHO). Two independent mutants termed Pro(-)5Lec20 and Gat(-)2Lec20, previously selected for lectin resistance, were found to have a galactosylation defect. Radiolabeled biantennary N-glycans synthesized by Pro(-)5Lec20 were proportionately less ricin-bound than similar species from parental CHO cells, and Lec20 cell extracts had a markedly reduced ability to transfer Gal to GlcNAc-terminating acceptors. Northern blot analysis revealed a severe reduction in beta4GalT-1 transcripts in Pro(-)5Lec20 cells. The Gat(-)2Lec20 mutant expressed beta4GalT-1 transcripts of reduced size due to a 311-base pair deletion in the beta4GalT-1 gene coding region. Northern analysis with probes from the remaining five beta4GalT genes revealed that Gat(-)2 CHO and Gat(-)2Lec20 cells express all six beta4GalT genes. Unexpectedly, the beta4GalT-6 gene is not expressed in either Pro(-)5 or Pro(-)5Lec20 cells. Thus, in addition to a deficiency in beta4GalT-1, Pro(-)5Lec20 cells lack beta4GalT-6. Nevertheless, matrix-assisted laser desorption/ionization time-of-flight mass spectrometry data of N-glycans released from cellular glycoproteins showed that both the beta4GalT-1(-) (Gat(-)2Lec20) and beta4GalT-1(-)/beta4GalT-6(-) (Pro(-)5Lec20) mutants have a similar Gal deficiency, affecting neutral and sialylated bi-, tri-, and tetraantennary N-glycans. By contrast, glycolipid synthesis was normal in both mutants. Therefore, beta4GalT-1 is a key enzyme in the galactosylation of N-glycans, but is not involved in glycolipid synthesis in CHO cells. beta4GalT-6 contributes only slightly to the galactosylation of N-glycans and is also not involved in CHO cell glycolipid synthesis. These CHO glycosylation mutants provide insight into the variety of in vivo substrates of different beta4GalTs. They may be used in glycosylation engineering and in investigating roles for beta4GalT-1 and beta4GalT-6 in generating specific glycan ligands.

566 ||| 11082196 ||| (3, 0.41),(4, 0.35) ||| 3 ||| 3 ||| Cloning and bacterial expression of monomeric short-chain dehydrogenase/reductase (carbonyl reductase) from CHO-K1 cells. Mammalian carbonyl reductase (EC 1.1.1.184) is an enzyme that can catalyze the reduction of many carbonyl compounds, using NAD(P)H. We isolated a cDNA of carbonyl reductase (CHO-CR) from CHO-K1 cells which was 1208 bp long, including a poly(A) tail, and contained an 831-bp ORF. The deduced amino-acid sequence of 277 residues contained a typical motif for NADP+-binding (TGxxxGxG) and an SDR active site motif (S-Y-K). CHO-CR closely resembles mammalian carbonyl reductases with 71-73% identity. CHO-CR cDNA had the highest similarity to human CBR3 with 86% identity. Using the pET-28a expression vector, recombinant CHO-CR (rCHO-CR) was expressed in Escherichia coli BL21 (DE3) cells and purified with a Ni2+-affinity resin to homogeneity with a 35% yield. rCHO-CR had broad substrate specificity towards xenobiotic carbonyl compounds. RT-PCR of Chinese hamster tissues suggest that CHO-CR is highly expressed in kidney, testis, brain, heart, liver, uterus and ovary. Southern blotting analysis indicated the complexity of the Chinese hamster carbonyl reductase gene.

567 ||| 17619940 ||| (3, 0.40),(2, 0.39) ||| 3 ||| 3 ||| Expression and purification of recombinant human angiopoietin-1 produced in Chinese hamster ovary cells. Angiopoietin-1 (Ang1) is an essential molecule for blood vessel formation. In an effort to produce large quantities of Ang1, recombinant Chinese hamster ovary (rCHO) cells expressing a high level of recombinant human Ang1 protein (rhAng1) with an amino terminal FLAG-tag were constructed by transfecting the expression vector into dihydrofolate reductase-deficient CHO cells and subsequent gene amplification in a medium containing step-wise increments of methotrexate, such as 0.02, 0.08, and 0.32 microM. The rhAng1 secreted from rCHO cells was purified at a purification yield of 18.4% from the cultured medium using an anti-FLAG M2 agarose affinity gel. SDS-PAGE and Western blot analyses showed that rCHO cells secret rhAng1 as heterogeneous multimers. Moreover, rhAng1 expressed in rCHO cells is biologically active in vitro as demonstrated by its ability to bind to the Tie2 receptor and to phosphorylate Tie2. Therefore, the rhAng1 produced from CHO cells could be useful for clarifying the biological effects of exogenous rhAng1 in the future.

568 ||| 8953280 ||| (3, 0.40),(2, 0.36) ||| 3 ||| 3 ||| CHO cells provide access to novel N-glycans and developmentally regulated glycosyltransferases. Chinese hamster ovary (CHO) cells express only a subset of the glycosyltransferases activities known to exist. They do not express several fucosyltransferases, galactosyltransferases, sialyltransferases or N-acetylglucosaminyltransferases. However, following mutagenesis or transfection with large amounts of DNA, rare mutants that express a transferase activity de novo have been obtained. The first CHO mutant of this type was LEC10, which expresses the N-acetylglucosaminyltransferase, GlcNAc-TIII, that adds the bisecting GlcNAc to complex N-glycans. Several analogous gain-of-function mutants have now been characterized and, all express a new glycosyltransferase activity. In several cases, expression is known to reflect gene activation at the transcriptional level. Thus, CHO cells contain quiescent glycosyltransferase genes that may be activated by mutational events. Several of these transferases have properties distinct from previously described enzymes. In fact, the most recently characterized dominant CHO mutants, LEC14 and LEC18, each express a GlcNAc-T activity that creates novel N-glycans never before observed in glycoproteins from any other source. In these and possibly other cases, it appears the CHO genome has provided access to new GlcNAc-Ts that may be difficult to identify by conventional methods.

569 ||| 7575473 ||| (3, 0.40),(2, 0.33) ||| 3 ||| 3 ||| Expression, purification and characterization of recombinant human N-acetylgalactosamine-6-sulphatase. Full-length cDNA sequences encoding human N-acetylgalactosamine-6-sulphatase were stably expressed in Chinese hamster ovary cells under the transcriptional control of the human polypeptide chain elongation factor 1 alpha gene promoter. A clonal cell line overexpressing recombinant N-acetylgalactosamine-6-sulphatase to a level of approx. 3 mg/l of culture medium was isolated. The secreted precursor enzyme was purified to homogeneity by a two-column procedure with an overall yield of 53% of the activity. The physical and catalytic parameters of the recombinant enzyme were similar to those of the mature form isolated from liver. On SDS/PAGE and gel filtration, recombinant N-acetylgalactosamine-6-sulphatase had a native molecular mass of 58-60 kDa. Recombinant N-acetylgalactosamine-6-sulphatase was endocytosed by mucopolysaccharidosis IVA fibroblasts via the mannose-6-phosphate receptor-mediated pathway and was efficiently localized to lysosomes.

570 ||| 9548768 ||| (3, 0.39),(2, 0.33) ||| 3 ||| 3 ||| Synthesis of bisected glycoforms of recombinant IFN-beta by overexpression of beta-1,4-N-acetylglucosaminyltransferase III in Chinese hamster ovary cells. Genetic engineering of oligosaccharide biosynthesis pathways in mammalian cells makes possible generation of new recombinant glycoproteins of potential importance in the biopharmaceutical industry. Most prior investigations of glycosylation engineering of secreted heterologous glycoproteins involve terminal steps of oligosaccharide biosynthesis. In particular, increasing the frequency of bisected structures within the glycoform distribution has not before been considered. A Chinese hamster ovary (CHO) cell line capable of producing bisected oligosaccharides on glycoproteins was created by overexpression of a recombinant N-acetylglucosaminyltransferase III (GnT-III). Interferon beta (IFN-beta) was chosen as a model and potential therapeutic secreted heterologous protein to demonstrate the effect of recombinant GnT-III-expression on product glycosylation. IFN-beta with bisected oligosaccharides was produced by the GnT-III-engineered CHO cells but not by the unmodified parental cell line.

571 ||| 23996121 ||| (3, 0.39) ||| 3 ||| 3 ||| Enhancement of human thyrotropin synthesis by sodium butyrate addition to serum-free CHO cell culture. The influence of sodium butyrate (NaBu) on the synthesis of recombinant human thyrotropin (r-hTSH) by CHO cells was investigated for the first time. A volumetric productivity of ~10 mug hTSH/mL was repeatedly obtained, with a 3.3-fold increase over a control culture carried out in the absence of NaBu. Since NaBu can induce CHO cell apoptosis and cell growth arrest, the increase in specific productivity was even higher, i.e., ca. 5-fold. Analysis of the N-glycan composition of r-hTSH obtained with the addition of NaBu to the culture medium showed an approximately 12 % increase in the amount of sialic acid, as well as in total carbohydrate, partly due to the increase in the site occupancy from 2.77 to 2.93 glycans per mole of hTSH. The two hormone preparations were characterized by N-glycan structural analysis, which showed that NaBu increased the bi-antennary structures by ca. 13 % while decreasing the tri-antennary structures by approximately the same amount. The in vivo biological activity and pharmacokinetic behavior (clearance) were found to be similar for the two hormone preparations.

572 ||| 11447136 ||| (3, 0.38),(4, 0.38) ||| 3 ||| 3 ||| N-glycan structure of a short-lived variant of ribophorin I expressed in the MadIA214 glycosylation-defective cell line reveals the role of a mannosidase that is not ER mannosidase I in the process of glycoprotein degradation. A soluble form of ribophorin I (RI(332)) is rapidly degraded in Hela and Chinese hamster ovary (CHO) cells by a cytosolic proteasomal pathway, and the N-linked glycan present on the protein may play an important role in this process. Specifically, it has been suggested that endoplasmic reticulum (ER) mannosidase I could trigger the targeting of improperly folded glycoproteins to degradation. We used a CHO-derived glycosylation-defective cell line, MadIA214, for investigating the role of mannosidase(s) as a signal for glycoprotein degradation. Glycoproteins in MadIA214 cells carry truncated Glc(1)Man(5)GlcNAc(2) N-glycans. This oligomannoside structure interferes with protein maturation and folding, leading to an alteration of the ER morphology and the detection of high levels of soluble oligomannoside species caused by glycoprotein degradation. An HA-epitope-tagged soluble variant of ribophorin I (RI(332)-3HA) expressed in MadIA214 cells was rapidly degraded, comparable to control cells with the complete Glc(3)Man(9)GlcNAc(2) N-glycan. ER-associated degradation (ERAD) of RI(332)-3HA was also proteasome-mediated in MadIA214 cells, as demonstrated by inhibition of RI(332)-3HA degradation with agents specifically blocking proteasomal activities. Two inhibitors of alpha1,2-mannosidase activity also stabilized RI(332)-3HA in the glycosylation-defective cell line. This is striking, because the major mannosidase activity in the ER is the one of mannosidase I, specific for a mannose alpha1,2-linkage that is absent from the truncated Man(5) structure. Interestingly, though the Man(5) derivative was present in large amounts in the total protein pool, the two major species linked to RI(332)-3HA shortly after synthesis consisted of Glc(1)Man(5 )and Man(4), being replaced by Man(4 )and Man(3) when proteasomal degradation was inhibited. In contrast, the untrimmed intermediate of RI(332)-3HA was detected in mutant cells treated with mannosidase inhibitors. Our results unambiguously demonstrate that an alpha1,2-mannosidase that is not ER mannosidase I is involved in ERAD of RI(332-)3HA in the glycosylation-defective cell line, MadIA214.

573 ||| 9949190 ||| (3, 0.38) ||| 3 ||| 3 ||| Incorporation of 15N from ammonium into the N-linked oligosaccharides of an immunoadhesin glycoprotein expressed in Chinese hamster ovary cells. Elevated ammonium concentrations in the medium of cultivated cells have been shown to increase the intracellular levels of uridine-5'-diphospho-N-acetylglucosamine (UDP-GlcNAc) and uridine-5'-diphospho-N-acetylgalactosamine (UDP-GalNAc; Ryll et al., 1994). These sugar nucleotides are substrates for glycosyltransferases in the glycosylation pathway. In our experiments, recombinant Chinese hamster ovary cells producing an immunoadhesin glycoprotein (GP1-IgG) have been cultivated under controlled cell culture conditions in the presence of different ammonium concentrations.15N-Labeled ammonium chloride (15NH4Cl) was added exogenously to the cell culture media to determine if ammonium was incorporated into UDP-GlcNAc and cytidine-5'-monophospho-N-acetylneuraminic acid (CMP-NANA) pools, and subsequently incorporated into GP1-IgG as N-linked glycans. The intracellular pools of UDP-activated hexosamines (UDP-GNAc) were followed during the time course of the experiment. To assess the extent of15NH4+incorporation into the glycans of GP1-IgG, the glycoprotein was first purified to homogeneity by protein A chromatography. Enzymatically released N-glycans were then analyzed by matrix-assisted laser desorption/ionization time-of-flight mass spectrometry. N-Glycans synthesized in the presence of15NH4Cl revealed an N-glycan-dependent increase in mass-to-charge of 2.5-4.8 Da. These results indicate that 60-70% of the total nitrogen containing monosaccharides had incorporated15N. Presumably,15NH4+was incorporated into GlcNAc and N-acetylneuraminic acid as proposed earlier (Ryll et al., 1994). This might be a universal and previously not described reaction in mammalian cells when exposed to nonphysiological but in cell culture commonly found concentrations of ammonium. The data presented here are of significance for glycoprotein production in mammalian cell culture, since it has been shown previously that elevated levels of UDP-activated hexosamines affect N-glycan characteristics such as branching and degree of amino sugar incorporation. In addition, our results demonstrate that isotope labeling in combination with MALDI-TOF-MS can be used as an alternate tool to radioactive labeling of sugar substrates in metabolic studies.

574 ||| 11231279 ||| (3, 0.37) ||| 3 ||| 3 ||| Overexpression of the Golgi-localized enzyme alpha-mannosidase IIx in Chinese hamster ovary cells results in the conversion of hexamannosyl-N-acetylchitobiose to tetramannosyl-N-acetylchitobiose in the N-glycan-processing pathway. Golgi alpha-mannosidase II is an enzyme that processes the intermediate oligosaccharide Gn(1)M(5)Gn(2) to Gn(1)M(3)Gn(2) during biosynthesis of N-glycans. Previously, we isolated a cDNA encoding a protein homologous to alpha-mannosidase II and designated it alpha-mannosidase IIx. Here, we show by immunocytochemistry that alpha-mannosidase IIx resides in the Golgi in HeLa cells. When coexpressed with alpha-mannosidase II, alpha-mannosidase IIx colocalizes with alpha-mannosidase II in COS cells. A protein A fusion of the catalytic domain of alpha-mannosidase IIx hydrolyzes a synthetic substrate, 4-umbelliferyl-alpha-D-mannoside, and this activity is inhibited by swainsonine. [(3)H]glucosamine-labeled Chinese hamster ovary cells overexpressing alpha-mannosidase IIx show a reduction of M(6)Gn(2) and an accumulation of M(4)Gn(2). Structural analysis identified M(4)Gn(2) to be Man alpha 1-->6(Man alpha 1-->2Man alpha 1-->3)Man beta 1-->4GlcNAc beta 1-->4GlcNAc. The results suggest that alpha-mannosidase IIx hydrolyzes two peripheral Man alpha 1-->6 and Man alpha 1-->3 residues from [(Man alpha 1-->6)(Man alpha 1-->3)Man alpha 1-->6](Man alpha 1-->2Man alpha 1-->3)Man beta 1-->4GlcNAc beta 1-->4GlcNAc, during N-glycan processing.

575 ||| 16901717 ||| (3, 0.36),(2, 0.31) ||| 3 ||| 3 ||| An improved recombinant mammalian cell expression system for human transforming growth factor-beta2 and -beta3 preparations. Transforming growth factor-beta2 and -beta3 (TGF-beta2 and -beta3) are important members of TGF-beta family which play important roles in the growth, maintenance, and repair processes of developing embryos, neonates, and adults. Preparation of large quantities of these two cytokines, which is necessary for structural studies and other applications, has proven to be extremely difficult. We have developed a novel Chinese hamster ovary cell-based expression system for high-level expression and high recovery of recombinant human TGF-beta2 and -beta3. In this system, we used a mammalian expression vector which contains a glutamine synthetase coding region for amplification, together with a modified TGF-beta2 or -beta3 open reading frame for expression. The leader peptide of TGF-beta2 or -beta3 was replaced by that from the V-J2-C region of a mouse immunoglobulin kappa-chain, and a poly-histidine tag was inserted immediately after the leader sequence to facilitate protein purification without changing the mature TGF-beta2 or -beta3 amino acid sequence. In addition, the extreme N-terminal cysteine residue of TGF-beta2 or -beta3 was replaced by a serine residue. The resulting expression constructs produced two stable cell clones expressing 10 mg of TGF-beta2 and 8 mg of TGF-beta3 per liter of spent medium. The purification scheme involved the use of two simple chromatographic steps with a typical yield of 5 mg of TGF-beta2 and 4 mg of TGF-beta3. This method represents a significant improvement over previously published methods and may be applicable to other TGF-beta superfamily members. We further confirmed that latent TGF-beta2 and -beta3 can be activated by proteolysis and glycolysis, which have not been reported before.

576 ||| 22703837 ||| (3, 0.36),(1, 0.36) ||| 3 ||| 3 ||| Discrimination between glycosylation patterns of therapeutic antibodies using a microfluidic platform, MALDI-MS and multivariate statistics. Optimal glycosylation with respect to the efficacy, serum half-life time, and immunogenic properties is essential in the generation of therapeutic antibodies. The glycosylation pattern can be affected by several different parameters during the manufacture of antibodies and may change significantly over cultivation time. Fast and robust methods for determination of the glycosylation patterns of therapeutic antibodies are therefore needed. We have recently presented an efficient method for the determination of glycans on therapeutic antibodies using a microfluidic CD platform for sample preparation prior to matrix-assisted laser-desorption mass spectrometry analysis. In the present work, this method is applied to analyse the glycosylation patterns of three commercially available therapeutic antibodies and one intended for therapeutic use. Two of the antibodies produced in mouse myeloma cell line (SP2/0) and one produced in Chinese hamster ovary (CHO) cells exhibited similar glycosylation patterns but could still be readily differentiated from each other using multivariate statistical methods. The two antibodies with most similar glycosylation patterns were also studied in an assessment of the method's applicability for quality control of therapeutic antibodies. The method presented in this paper is highly automated and rapid. It can therefore efficiently generate data that helps to keep a production process within the desired design space or assess that an identical product is being produced after changes to the process.

577 ||| 8746631 ||| (3, 0.35) ||| 3 ||| 3 ||| Purification and properties of insulin receptor ectodomain from large-scale mammalian cell culture. Ectodomain of the exon 11+ form of the human insulin receptor (hIR) was expressed in the mammalian cell secretion vector pEE6.HCMV-GS, containing the glutamine synthetase gene. Following transfection of the hIR ectodomain gene into Chinese hamster ovary (CHO-K1) cells, clones were isolated by selecting for glutamine synthetase expression with methionine sulphoximine. The expression levels of ectodomain were subsequently increased by gene amplification. Production was scaled up using a 40-liter airlift fermenter in which the transfected CHO-K1 cells were cultured on microcarrier beads, initially in medium containing 10% fetal calf serum (FCS). By continuous perfusion of serum-free medium into the bioreactor, cell viability was maintained during reduction of FCS, which enabled soluble hIR ectodomain to be harvested for at least 22 days. Harvests were concentrated 20-fold by anion-exchange chromatography. Optimal recovery of ectodomain from early harvests containing large quantities of serum proteins was achieved by insulin-affinity chromatography, whereas in later harvests purification was achieved by multistep chromatography. Analysis of the purified hIR ectodomain showed that it had a molecular weight by sedimentation equilibrium analysis of 269,500. Amino-terminal amino acid sequence analysis showed that the ectodomain was correctly processed to alpha and beta chains and that glycosylation characteristics were similar to those of native hIR. The integrity of the ectodomain was demonstrated by the recognition of conformation-dependent anti-hIR antibodies and by its binding of insulin (Kd approximately 2 x 10(-9) M). These results demonstrate the successful production and purification of hIR ectodomain by processes amenable to scale-up and in a form appropriate for structure/function studies of the ligand-binding domain of the receptor.

578 ||| 25087738 ||| (3, 0.35) ||| 3 ||| 3 ||| Ethanol precipitation for purification of recombinant antibodies. Currently, the golden standard for the purification of recombinant humanized antibodies (rhAbs) from CHO cell culture is protein A chromatography. However, due to increasing rhAbs titers alternative methods have come into focus. A new strategy for purification of recombinant human antibodies from CHO cell culture supernatant based on cold ethanol precipitation (CEP) and CaCl2 precipitation has been developed. This method is based on the cold ethanol precipitation, the process used for purification of antibodies and other components from blood plasma. We proof the applicability of the developed process for four different antibodies resulting in similar yield and purity as a protein A chromatography based process. This process can be further improved using an anion-exchange chromatography in flowthrough mode e.g. a monolith as last step so that residual host cell protein is reduced to a minimum. Beside the ethanol based process, our data also suggest that ethanol could be replaced with methanol or isopropanol. The process is suited for continuous operation.

579 ||| 11469797 ||| (3, 0.35) ||| 3 ||| 3 ||| The widespread effect of beta 1,4-galactosyltransferase on N-glycan processing. We investigated beta 1,4-GalT (UDP-galactose: beta-d-N-acetylglucosaminide beta 1,4-galactosyltransferase) in terms of intracellular competition with GnT-IV (UDP-N-acetylglucosamine: alpha1,3-d-mannoside beta1,4-N-acetylglucosaminyltransferase) and GnT-V (UDP-N-acetylglucosamine: alpha1,6-d-mannoside beta 1,6-N-acetylglucosaminyltransferase). The beta 1,4-GalT-I gene was introduced into Chinese hamster ovary (CHO) cells producing human interferon (hIFN)-gamma (IM4/V/IV cells) and five clones expressing various levels of beta 1,4-GalT were isolated. As we previously reported, parental IM4/V/IV cells express high levels of GnT-IVa and -V and produce hIFN-gamma having primarily tetraantennary sugar chains. The branching of sugar chains on hIFN-gamma was suppressed in the beta 1,4-GalT-enhanced clones to a level corresponding to the intracellular activity of beta 1,4-GalT relative to GnTs. Moreover, the contents of hybrid-type and high-mannose-type sugar chains increased in these clones. The results showed that beta 1,4-GalT widely affects N-glycan processing by competing with GnT-IV, GnT-V, and alpha-mannosidase II in cells and also by some other mechanisms that suppress the conversion of high-mannose-type sugar chains to the hybrid type.

580 ||| 21443274 ||| (3, 0.33) ||| 3 ||| 3 ||| Evaluation of process efficiency and bioequivalence of biosimilar recombinant human chorionic gonadotropin (rhCG). BACKGROUND: Human chorionic gonadotropin (hCG) is a therapeutic protein used for ovulation induction in women with infertility. Dong-A Pharm. Co. has developed recombinant hCG (rhCG) [product code DA-3803] produced in Chinese hamster ovary cells and evaluated its biologic properties, such as biologic potency, efficacy, and pharmacokinetic profile, compared with a reference product, Ovidrel . OBJECTIVE: The purpose of this study was to evaluate the efficiency of the purification process of Dong-A rhCG (DA-3803) and its bioequivalence from a biosimilar perspective. METHODS: The efficiency of the purification process was estimated through scale-down clearance studies for viruses, endotoxins, host cell DNAs (HCDs) and host cell proteins (HCPs). To confirm bioequivalence, the in vivo/in vitro biologic potency, ovulation induction rate, and pharmacokinetic profile of DA-3803 were compared with those of Ovidrel . RESULTS: In the clearance studies, the lowest log reduction value (LRV) for model viruses was 8.43. LRVs for endotoxins, HCDs, and HCPs were greater than 5.27, 16.36, and 3.37, respectively. DA-3803 showed equivalent potency with Ovidrel , and similarity between DA-3803 and Ovidrel was observed in an efficacy evaluation that measured ovulation induction. The bioequivalence was also confirmed in a rat pharmacokinetic study, which compared pharmacokinetic parameters such as maximum serum concentration, area under the concentration-time curve, time to reach maximum serum concentration, and half-life. In a comparison of different isoform groups of DA-3803, it was shown that the potency and pharmacokinetic profile depend on the sialic acid content. CONCLUSION: The purification process of DA-3803 was effective in removing the major process impurities, and DA-3803 showed similar biologic properties to the reference drug, Ovidrel .

581 ||| 11551899 ||| (3, 0.32) ||| 3 ||| 3 ||| 6-O-sulfotransferase-1 represents a critical enzyme in the anticoagulant heparan sulfate biosynthetic pathway. Using recombinant retroviral transduction, we have introduced the heparin/heparan sulfate (HS) 3-O-sulfotransferase 1 (3-OST-1) gene into Chinese hamster ovary (CHO) cells. Expression of 3-OST-1 confers upon CHO cells the ability to produce anticoagulantly active HS (HS(act)). To understand how 6-OST and other proteins regulate HS(act) biosynthesis, a CHO cell clone with three copies of 3-OST-1 was chemically mutagenized. Resulting mutants that make HS but are defective in generating HS(act) were single-cell-cloned. One cell mutant makes fewer 6-O-sulfated residues. Modification of HS chains from the mutant with pure 6-OST-1 and 3'-phosphoadenosine 5'-phosphosulfate increased HS(act) from 7% to 51%. Transfection of this mutant with 6-OST-1 created a CHO cell line that makes HS, 50% of which is HS(act). We discovered in this study that (i) 6-OST-1 is a limiting enzyme in the HS(act) biosynthetic pathway in vivo when the limiting nature of 3-OST-1 is removed; (ii) HS chains from the mutant cells serve as an excellent substrate for demonstrating that 6-OST-1 is the limiting factor for HS(act) generation in vitro; (iii) in contradiction to the literature, 6-OST-1 can add 6-O-sulfate to GlcNAc residues, especially the critical 6-O-sulfate in the antithrombin binding motif; (iv) both 3-O- and 6-O-sulfation can be the final step in HS(act) biosynthesis in contrast to prior publications that concluded 3-O-sulfation is the final step in HS(act) biosynthesis; (v), in the presence of HS interacting protein peptide, 3-O-sulfate-containing sugars can be degraded into disaccharides by heparitinase digestion as demonstrated by capillary high performance liquid chromatography coupled with mass spectrometry.

582 ||| 9341186 ||| (3, 0.31) ||| 3 ||| 3 ||| O-Glycosylation of C-terminal tandem-repeated sequences regulates the secretion of rat pancreatic bile salt-dependent lipase. Amino acid sequences rich in Pro, Glu, Ser, and Thr (PEST) are common to rapidly degraded proteins (Rogers, S., Wells, R. & Rechsteiner, M. (1986) Science 234, 364-368). On pancreatic bile salt-dependent lipase (BSDL), PEST sequences are present in the C-terminal region of the enzyme to which is associated the O-glycosylation. We have postulated that the O-glycosylation of BSDL may contribute to mask PEST sequences and to trigger the secretion of this enzyme instead of its delivery into a degradative pathway (Bruneau, N., and Lombardo, D. (1995) J. Biol. Chem. 270, 13524-13523). To further examine the role of the O-linked glycosylation on BSDL metabolism, rat pancreatic BSDL cDNA was stably transfected into two Chinese hamster ovary (CHO) cell lines, the CHO K1 wild-type line and the O-glycosylation defective CHO ldlD line. In these latter cells, O-glycosylation can be reversibly modulated by culture conditions. Results indicate that the rate of BSDL synthesis by transfected CHO K1 or CHO ldlD cells reflects, independently of culture conditions, the amount of mRNA specific for BSDL present in these transfected cells. Nevertheless, the rate of secretion of the enzyme depends upon cell culture conditions and increases with the cell capability to O-glycosylate C-terminal tandem-repeated sequences. Immunoprecipitation experiments performed on cell lysates suggested that a rapid degradation of BSDL occurred particularly when transfected CHO ldlD cells were cultured under non-permissive conditions. We further showed that BSDL secreted by CHO ldlD cells grown under non-permissive conditions that normally prevent O-glycosylation incorporated galactose and was reactive with peanut agglutinin, which recognizes the core structure of O-linked glycans. We concluded that the BSDL expressed by CHO ldlD cells grown under non-permissive conditions was rapidly degraded but a fraction of the enzyme was allowed to O-glycosylate and consequently was secreted.

583 ||| 11511814 ||| (3, 0.31) ||| 3 ||| 3 ||| Genetic engineering of CHO cells producing human interferon-gamma by transfection of sialyltransferases. Natural human interferon-gamma (hIFN-gamma) contains mainly biantennary complex-type sugar chains. We previously remodeled the branch structures of N-glycans on hIFN-gamma in Chinese hamster ovary (CHO) cells by overexpressing UDP-N-acetylglucosamine: alpha1,6-D-mannoside beta1,6-N-acetylglucosaminyltransferase (GnT-V). Normal CHO cells primarily produced hIFN-gamma having biantennary sugar chains, whereas a CHO clone, designated IM4/Vh, transfected with GnT-V, primarily produced hIFN-gamma having GlcNAcbeta1-6 branched triantennary sugar chains when sialylation was incomplete and an increase in poly-N-acetyllactosamine (Galbeta1-4GlcNAcbeta1-3)n was observed. In the present study, we introduced mouse Galbeta1-3/4GlcNAc-R alpha2,3-sialyltransferase (ST3Gal IV) and/or rat Galbeta1-4GlcNAc-R alpha2,6-sialyltransferase (ST6Gal I) cDNAs into the IM4/Vh cells to increase the extent of sialylation and to examine the effect of sialyltransferase (ST) type on the linkage of sialic acid. Furthermore, we speculated that sialylation extent might affect the level of poly-N-acetyllactosamine. We isolated four clones expressing different levels of alpha2,3-ST and/or alpha2,6-ST. The extent of sialylation of hIFN-gamma from the IM4/Vh clone was 61.2%, which increased to about 80% in every ST transfectant. The increase occurred regardless of the type of overexpressed ST, and the proportion of alpha2,3- and alpha2,6-sialic acid corresponded to the activity ratio of alpha2,3-ST to alpha2,6-ST. Furthermore, the proportion of N-glycans containing poly-N-acetyllactosamine was significantly reduced (less than 10%) in the ST transfectants compared with the parental IM4/Vh clone (22.9%). These results indicated that genetic engineering of STs is highly effective for regulating the terminal structures of sugar chains on recombinant proteins in CHO cells.

584 ||| 10930398 ||| (3, 0.31) ||| 3 ||| 3 ||| Active aminoacyl-tRNA synthetases are present in nuclei as a high molecular weight multienzyme complex. Recent studies suggest that aminoacylation of tRNA may play an important role in the transport of these molecules from the nucleus to the cytoplasm. However, there is almost no information regarding the status of active aminoacyl-tRNA synthetases within the nuclei of eukaryotic cells. Here we show that at least 13 active aminoacyl-tRNA synthetases are present in purified nuclei of both Chinese hamster ovary and rabbit kidney cells, although their steady-state levels represent only a small percentage of those found in the cytoplasm. Most interestingly, all the nuclear aminoacyl-tRNA synthetases examined can be isolated as part of a multienzyme complex that is more stable, and consequently larger, than the comparable complex isolated from the cytoplasm. These data directly demonstrate the presence of active aminoacyl-tRNA synthetases in mammalian cell nuclei. Moreover, their unexpected structural organization raises important questions about the functional significance of these multienzyme complexes and whether they might play a more direct role in nuclear to cytoplasmic transport of tRNAs.

585 ||| 7772861 ||| (3, 0.30) ||| 3 ||| 3 ||| Genomic organization and expression of hamster UDP-N-acetylglucosamine:dolichyl phosphate N-acetylglucosaminyl phosphoryl transferase. The hamster gene for uridine diphosphate N-acetyl-D-glucosamine:dolichyl phosphate N-acetylglucosaminyl phosphoryl transferase (L-G1PT) was found to extend over 6.5 kb and to contain nine exons. The exons ranged in size from 63 to 214 bp, encoding the 408 amino acid protein. The introns ranged from 85 bp to 1.4 kb. Upstream 5' sequences included two possible TATA boxes, one possible CCAAT box and at least two potential GC boxes. Heterologous expression was successful in Schizosaccharomyces pombe, and resulted in cells that were tunicamycin resistant and had 12-fold more L-G1PT activity than wild-type cells. Antiserum prepared to a hydrophilic peptide (residues 300-320) of the L-G1PT protein reacted with a 35-36 kDa protein in membrane samples from Chinese hamster ovary (CHO) cells and S. pombe cells that had increased levels of L-G1PT activity. In both cases, antigenic peptide competed with the 35-36 kDa protein detected by the antiserum.

586 ||| 24315529 ||| (3, 0.30) ||| 3 ||| 3 ||| Trehalose suppresses antibody aggregation during the culture of Chinese hamster ovary cells. The aggregation of therapeutic antibodies during the manufacturing process is problematic because of the potential risks posed by the aggregates, such as an unexpected immune response. One of the hallmark effects of trehalose, a disaccharide consisting of two alpha-glucose units, is as a chemical chaperone with anti-aggregation activity. In this study, Chinese hamster ovary (CHO) cell line producing a diabody-type bispecific antibody were cultured in medium containing trehalose and the aggregation of the secreted proteins during the culture process was analyzed. An analysis of the various forms of the antibody (monomeric, dimeric, and large aggregates) showed that trehalose decreased the relative content of large aggregates by two thirds. The aggregation kinetics indicated that trehalose directly inhibited the polymerization and aggregation steps in a nucleation-dependent aggregation mechanism. Moreover, both specific and volumetric antibody production were increased in CHO cells cultured in trehalose-containing medium. Thus, the addition of trehalose to recombinant CHO cell cultures would offer a practical strategy for quality improvement in the production of therapeutic antibodies.

# ----------------------------- TOPIC4 -----------------------------

587 ||| 9456320 ||| (4, 0.99) ||| 4 ||| 5 ||| ARF6 targets recycling vesicles to the plasma membrane: insights from an ultrastructural investigation. We have shown previously that the ADP-ribosylation factor (ARF)-6 GTPase localizes to the plasma membrane and intracellular endosomal compartments. Expression of ARF6 mutants perturbs endosomal trafficking and the morphology of the peripheral membrane system. However, another study on the distribution of ARF6 in subcellular fractions of Chinese hamster ovary (CHO) cells suggested that ARF6 did not localize to endosomes labeled after 10 min of horseradish peroxidase (HRP) uptake, but instead was uniquely localized to the plasma membrane, and that its reported endosomal localization may have been a result of overexpression. Here we demonstrate that at the lowest detectable levels of protein expression by cryoimmunogold electron microscopy, ARF6 localized predominantly to an intracellular compartment at the pericentriolar region of the cell. The ARF6-labeled vesicles were partially accessible to HRP only on prolonged exposure to the endocytic tracer but did not localize to early endocytic structures that labeled with HRP shortly after uptake. Furthermore, we have shown that the ARF6-containing intracellular compartment partially colocalized with transferrin receptors and cellubrevin and morphologically resembled the recycling endocytic compartment previously described in CHO cells. HRP labeling in cells expressing ARF6(Q67L), a GTP-bound mutant of ARF6, was restricted to small peripheral vesicles, whereas the mutant protein was enriched on plasma membrane invaginations. On the other hand, expression of ARF6(T27N), a mutant of ARF6 defective in GDP binding, resulted in an accumulation of perinuclear ARF6-positive vesicles that partially colocalized with HRP on prolonged exposure to the tracer. Taken together, our findings suggest that ARF activation is required for the targeted delivery of ARF6-positive, recycling endosomal vesicles to the plasma membrane.

588 ||| 8687482 ||| (4, 0.99) ||| 4 ||| 5 ||| Bioactivation of mitomycin antibiotics by aerobic and hypoxic Chinese hamster ovary cells overexpressing DT-diaphorase. DT-Diaphorase catalyzes a two-electron reduction of mitomycin C (MC) and porfiromycin (POR) to reactive species. Many cell lines that overexpress DT-diaphorase and are sensitive to the mitomycins are protected from the aerobic cytotoxicity of these drugs by the DT-diaphorase inhibitor dicumarol. The cytoprotective properties of this relatively non-specific inhibitor, however, vanish under hypoxic conditions. To ascertain the role of DT-diaphorase in mitomycin bioactivation and cytotoxicity in living cells, a rat liver DT-diaphorase cDNA was transfected into Chinese hamster ovary cells. MC was equitoxic to the parental cells under oxygenated and hypoxic conditions. In contrast, POR was less toxic than MC to these cells under aerobic conditions, but significantly more toxic than MC under hypoxia. Two DT-diaphorase-transfected clones displayed increases in DT-diaphorase activity of 126- and 133-fold over parental cells. The activities of other oxidoreductases implicated in mitomycin bioreduction were unchanged. MC was more toxic to both DT-diaphorase-transfected lines than to parental cells; the toxicity of MC to the transfected lines was similar in air and hypoxia. POR was also more toxic to the DT-diaphorase-elevated clones than to parental cells under oxygenated conditions. Under hypoxia, however, the toxicity of POR to the transfected clones was unchanged from that of parental cells. The findings implicate DT-diaphorase in mitomycin bioactivation in living cells, but suggest that this enzyme does not contribute to the differential toxicity of MC or POR in air and hypoxia.

589 ||| 8682648 ||| (4, 0.99) ||| 4 ||| 5 ||| Synthesis and secretion of lipoprotein lipase in heparan sulfate-deficient Chinese hamster ovary cells. Synthesis and secretion of lipoprotein lipase was studied in two mutants of Chinese hamster ovary (CHO) cells which, due to a lack of xylosyl transferase (pgsA-745) or galactosyl transferase (pgsB-761), respectively, were deficient in heparan sulfate and chondroitin sulfate. One of the mutants (pgsB-761) was two- to threefold more active in synthesis and secretion of catalytically active lipoprotein lipase than the other mutant, which was about as active as the wild-type (K1) cells. A similar relation was found when lipoprotein lipase was metabolically labelled with 35S-methionine and then immunoprecipitated. Heparin stimulated secretion from all three cell types to a similar extent (about twofold). Heparin-releasable binding of 125I-labelled lipoprotein lipase was lower to either of the mutant cells than to the wild-type cells. Binding to the wild-type cells was reduced by heparitinase, while the low binding to the mutants was not affected. By immunogold labelling of cryosections, lipoprotein lipase was detected on the plasma membranes and on the inside of secretory vesicles of both wild-type and mutant cells, suggesting that some carrier could be involved. Inhibition of vesicular transport by monensin caused accumulation of lipoprotein lipase in the cells. In wild-type cells the lipase was mainly on the inside of vesicular structures, while in the mutants the main part was associated with membranous bodies that formed within the vesicles during a chase period. These results suggest that if lipoprotein lipase needs a carrier during intracellular assembly and transport, this function can be fulfilled by some structure other than heparan sulfate.

590 ||| 8631914 ||| (4, 0.99) ||| 4 ||| 5 ||| Wortmannin-sensitive trafficking pathways in Chinese hamster ovary cells. Differential effects on endocytosis and lysosomal sorting. Phosphatidylinositol (PI) 3'-kinases are a family of lipid kinases implicated in the regulation of cell growth by oncogene products and tyrosine kinase growth factor receptors. The catalytic subunit of the p85/p110 PI 3'-kinase is homologous to VPS-34, a phosphatidylinositol-specific lipid kinase involved in the sorting of newly synthesized hydrolases to the yeast vacuole. This suggests that PI 3'-kinases may play analogous roles in mammalian cells. We have measured a number of secretory and endocytic trafficking events in Chinese hamster ovary cells in the presence of wortmannin, a potent inhibitor of PI 3'-kinase. Wortmannin caused a 40-50% down-regulation of surface transferrin receptors, with a dose dependence identical to that required for maximal inhibition of the p85/p110 PI 3'-kinase in intact cells. The redistribution of transferrin receptors reflected a 60% increase in the internalization rate and a 35% decrease in the recycling rate. Experiments with fluorescent transferrin showed that entry of transferrin receptors into the recycling compartment and efflux of receptors out of the compartment were slowed by wortmannin. Wortmannin altered the morphology of the recycling compartment, which was more vesiculated than in untreated cells. Using Semliki Forest virus as a probe, we also found that delivery of the endocytosed virus to its lysosomal site of degradation was slowed by wortmannin, whereas endosomal acidification was unaffected. In contrast to these effects on endocytosis and recycling, wortmannin did not affect intracellular processing of newly synthesized viral spike proteins. Wortmannin did induce missorting of the lysosomal enzyme cathepsin D to the secretory pathway, but only at a dose 20-fold greater than that required to inhibit p85/p110 PI 3'-kinase activity or to redistribute transferrin receptors. Our data demonstrate the presence of wortmannin-sensitive enzymes at three distinct steps of the endocytic cycle in Chinese hamster ovary cells: internalization, transit from early endosomes to the recycling and degradative compartments, and transit from the recycling compartment back to the cell surface. The wortmannin-sensitive enzymes critical for endocytosis and recycling are distinct from those involved in sorting newly synthesized lysosomal enzymes.

591 ||| 23269685 ||| (4, 0.99) ||| 4 ||| 5 ||| Role for phospholipid flippase complex of ATP8A1 and CDC50A proteins in cell migration. Type IV P-type ATPases (P4-ATPases) and CDC50 family proteins form a putative phospholipid flippase complex that mediates the translocation of aminophospholipids such as phosphatidylserine (PS) and phosphatidylethanolamine (PE) from the outer to inner leaflets of the plasma membrane. In Chinese hamster ovary (CHO) cells, at least eight members of P4-ATPases were identified, but only a single CDC50 family protein, CDC50A, was expressed. We demonstrated that CDC50A associated with and recruited P4-ATPase ATP8A1 to the plasma membrane. Overexpression of CDC50A induced extensive cell spreading and greatly enhanced cell migration. Depletion of either CDC50A or ATP8A1 caused a severe defect in the formation of membrane ruffles, thereby inhibiting cell migration. Analyses of phospholipid translocation at the plasma membrane revealed that the depletion of CDC50A inhibited the inward translocation of both PS and PE, whereas the depletion of ATP8A1 inhibited the translocation of PE but not that of PS, suggesting that the inward translocation of cell-surface PE is involved in cell migration. This hypothesis was further examined by using a PE-binding peptide and a mutant cell line with defective PE synthesis; either cell-surface immobilization of PE by the PE-binding peptide or reduction in the cell-surface content of PE inhibited the formation of membrane ruffles, causing a severe defect in cell migration. These results indicate that the phospholipid flippase complex of ATP8A1 and CDC50A plays a major role in cell migration and suggest that the flippase-mediated translocation of PE at the plasma membrane is involved in the formation of membrane ruffles to promote cell migration.

592 ||| 17021051 ||| (4, 0.99) ||| 4 ||| 5 ||| An intracellular multi-effector complex mediates somatostatin receptor 1 activation of phospho-tyrosine phosphatase eta. The receptor-like phosphotyrosine phosphatase eta (PTPeta) is an important intracellular effector of the cytostatic action of SST. Here we characterize, in Chinese hamster ovary-k1 cells, the intracellular pathway that from somatostatin receptor 1 (SSTR1), leads to the activation of PTPeta and that involves, in a multimeric complex and sequential activation, the tyrosine kinases Janus kinase (JAK) 2 and Src, and the cytosolic phosphotyrosine phosphatase SHP-2. We show that inhibitors of JAK2 and Src and dominant-negative mutants of SHP-2 and Src abolished the SSTR1-mediated PTPeta activation, suggesting that all these effectors participate in the activation of PTPeta. In basal conditions, JAK2 forms a multimeric complex with SHP-2, Src and PTPeta. In response to SST, JAK2 is activated in a G protein-dependent manner, dissociates from and phosphorylates SHP-2, increasing its activity. Subsequently, SHP-2 dissociates from Src, dephosphorylates the Src inhibitory tyrosine-529, and causes an autocatalytical increase of the phosphorylation of Src tyrosine 418, located inside its kinase activation loop. Active Src, in turn, controls the activity of PTPeta, via a direct interaction and phosphorylation of the phosphatase. These data for the first time depict an intracellular pathway involving a precise sequence of interactions and cross-activation among tyrosine phosphatases and kinases acting upstream of PTPeta. In particular the sequential activation of JAK2, SHP-2, and Src conveys the molecular signaling from SSTR1 to the activation of this phosphatase that is responsible for the final biological effects of SST.

593 ||| 10085248 ||| (4, 0.99) ||| 4 ||| 5 ||| An arrested late endosome-lysosome intermediate aggregate observed in a Chinese hamster ovary cell mutant isolated by novel three-step screening. Chinese hamster ovary cell mutants defective in the post-uptake degradation of low-density lipoprotein (LDL) in lysosomes were selected from mutagenized cells by novel three-step screening. First, in the presence of LDL, clones sensitive to an inhibitor of the rate-limiting enzyme of the cholesterol biosynthetic pathway, 3-hydroxy-3-methylglutaryl-CoA reductase, were isolated. Second, from the selected clones, those lacking in the degradation of a constituent of a fluorescent LDL were qualitatively screened by microscopy. Third, the clones were further screened by previously established quantitative analytical flow cytometry that detects the early-phase disintegration of LDL by lysosomal acid hydrolases. One of the isolated mutant clones, LEX1 (Lysosome-Endosome X 1), was a recessive mutant, and exhibited a specific disorder in the late endocytic pathway. LEX1 cells showed an unusual perinuclear aggregate of vesicles, heterogeneously positive for lysosomal glycoprotein-B/cathepsin D and rab7, yet negative for the cation-independent mannose 6-phosphate receptor. The aggregate was formed around the microtubule organizing center, and was disrupted by nocodazole treatment. Internalized octadecyl rhodamine B-labeled LDL (R18-LDL) was accumulated in the perinuclear rab7-positive vesicles. In a Percoll density gradient, neither internalized R18-LDL nor internalized horseradish peroxidase was efficiently chased into heavy lysosomal fractions positive for beta-hexosaminidase. LEX1 cells showed differences in the activity and subcellular distribution of lysosomal enzymes. These characteristics of LEX1 cells are consistent with the ideas that the perinuclear vesicle aggregate is an arrested intermediate of direct fusion or divergence between lysosomes and rab7-positive, cation-independent mannose 6-phosphate receptor-negative late endosomes, and that equilibrium between the lysosomes and the late endosomes is shifted towards the late endosomes in LEX1 cells. Such fusion or divergence between the late endosomes and the lysosomes would determine an appropriate equilibrium between them, and might thereby play an important role for proper lysosomal digestive functions. LEX1 mutant cells would be helpful for the dissection of the as yet unrevealed details of the late endocytic membrane dynamics and for the identification of factors involved in the process arrested by the mutation.

594 ||| 10318801 ||| (4, 0.95) ||| 4 ||| 5 ||| Studies of the role of group VI phospholipase A2 in fatty acid incorporation, phospholipid remodeling, lysophosphatidylcholine generation, and secretagogue-induced arachidonic acid release in pancreatic islets and insulinoma cells. An 84-kDa group VI phospholipase A2 (iPLA2) that does not require Ca2+ for catalysis has been cloned from Chinese hamster ovary cells, murine P388D1 cells, and pancreatic islet beta-cells. A housekeeping role for iPLA2 in generating lysophosphatidylcholine (LPC) acceptors for arachidonic acid incorporation into phosphatidylcholine (PC) has been proposed because iPLA2 inhibition reduces LPC levels and suppresses arachidonate incorporation and phospholipid remodeling in P388D1 cells. Because islet beta-cell phospholipids are enriched in arachidonate, we have examined the role of iPLA2 in arachidonate incorporation into islets and INS-1 insulinoma cells. Inhibition of iPLA2 with a bromoenol lactone (BEL) suicide substrate did not suppress and generally enhanced [3H]arachidonate incorporation into these cells in the presence or absence of extracellular calcium at varied time points and BEL concentrations. Arachidonate incorporation into islet phospholipids involved deacylation-reacylation and not de novo synthesis, as indicated by experiments with varied extracellular glucose concentrations and by examining [14C]glucose incorporation into phospholipids. BEL also inhibited islet cytosolic phosphatidate phosphohydrolase (PAPH), but the PAPH inhibitor propranolol did not affect arachidonate incorporation into islet or INS-1 cell phospholipids. Inhibition of islet iPLA2 did not alter the phospholipid head-group classes into which [3H]arachidonate was initially incorporated or its subsequent transfer from PC to other lipids. Electrospray ionization mass spectrometric measurements indicated that inhibition of INS-1 cell iPLA2 accelerated arachidonate incorporation into PC and that inhibition of islet iPLA2 reduced LPC levels by 25%, suggesting that LPC mass does not limit arachidonate incorporation into islet PC. Gas chromatography/mass spectrometry measurements indicated that BEL but not propranolol suppressed insulin secretagogue-induced hydrolysis of arachidonate from islet phospholipids. In islets and INS-1 cells, iPLA2 is thus not required for arachidonate incorporation or phospholipid remodeling and may play other roles in these cells.

595 ||| 9295290 ||| (4, 0.94) ||| 4 ||| 5 ||| An animal cell mutant with a deficiency in acyl/alkyl-dihydroxyacetone-phosphate reductase activity. Effects on the biosynthesis of ether-linked and diacyl glycerolipids. In the accompanying paper (James, P. F., and Zoeller, R. A. (1997) J. Biol. Chem. 272, 23532-23539), we reported the isolation of a series of mutants from the fibroblast-like cell line, CHO-K1, that are deficient in the incorporation of the long chain fatty alcohol, hexadecanol, into complex lipids. All but one of these mutants, FAA. K1B, were deficient in long-chain-fatty alcohol oxidase (FAO) activity. We have further characterized this FAO+ isolate. FAA.K1B cells displayed a 40% decrease in [9,10-3H]hexadecanol uptake when compared with the parent strain. Although incorporation of hexadecanol into the phospholipid fraction was decreased by 52%, the cells accumulated label in alkylglycerol (20-fold over wild type). The increase in 1-alkylglycerol labeling corresponded to a 4-fold increase in alkylglycerol mass. Short term labeling with 32Pi showed a 45-50% decrease in overall phospholipid biosynthesis in FAA.K1B. Both diacyl- and ether-linked species were affected, suggesting a general defect in phospholipid biosynthesis. Mutant cells were able to partially compensate for the decreased biosynthesis by decreasing the turnover of the phospholipid pools. The primary lesion in FAA. K1B was identified as a 95% reduction in acyl/alkyl-dihydroxyacetone-phosphate reductase activity. Whole cell homogenates from FAA.K1B were unable to reduce either acyl-dihydroxyacetone phosphate (DHAP) or alkyl-DHAP, supporting the notion that the reduction of these two compounds is catalyzed by a single enzyme. These data suggest that the biosynthesis of diacyl phospholipids, in Chinese hamster ovary cells, begins with the acylation of dihydroxyacetone phosphate as well as glycero-3-phosphate and that the "DHAP pathway" contributes significantly to diacyl glycerolipid biosynthesis. Also, the severe reduction in acyl/alkyl-DHAP reductase activity in FAA.K1B resulted in only a moderate decrease in ether lipid biosynthesis. These latter data together with the observed increase in alkylglycerol levels support the existence of a shunt pathway that is able to partially bypass the enzymatic lesion.

596 ||| 16571669 ||| (4, 0.93) ||| 4 ||| 5 ||| Oxysterol-binding protein and vesicle-associated membrane protein-associated protein are required for sterol-dependent activation of the ceramide transport protein. Sphingomyelin (SM) and cholesterol are coregulated metabolically and associate physically in membrane microdomains involved in cargo sorting and signaling. One mechanism for regulation of this metabolic interface involves oxysterol binding protein (OSBP) via high-affinity binding to oxysterol regulators of cholesterol homeostasis and activation of SM synthesis at the Golgi apparatus. Here, we show that OSBP regulation of SM synthesis involves the endoplasmic reticulum (ER)-to-Golgi ceramide transport protein (CERT). RNA interference (RNAi) experiments in Chinese hamster ovary (CHO)-K1 cells revealed that OSBP and vesicle-associated membrane protein-associated protein (VAP) were required for stimulation of CERT-dependent ceramide transport and SM synthesis by 25-hydroxycholesterol and cholesterol depletion in response to cyclodextrin. Additional RNAi experiments in human embryonic kidney 293 cells supported OSBP involvement in oxysterol-activated SM synthesis and also revealed a role for OSBP in basal SM synthesis. Activation of ER-to-Golgi ceramide transport in CHO-K1 cells required interaction of OSBP with the ER and Golgi apparatus, OSBP-dependent Golgi translocation of CERT, and enhanced CERT-VAP interaction. Regulation of CERT by OSBP, sterols, and VAP reveals a novel mechanism for integrating sterol regulatory signals with ceramide transport and SM synthesis in the Golgi apparatus.

597 ||| 9281305 ||| (4, 0.92) ||| 4 ||| 5 ||| Farnesol as a regulator of HMG-CoA reductase degradation: characterization and role of farnesyl pyrophosphatase. We have recently reported that the isoprenoid compound farnesol accelerates degradation of the cholesterologenic enzyme 3-hydroxy-3-methylglutaryl coenzyme A (HMG-CoA) reductase, when added to cultured cells. We have thus proposed that farnesol is a required nonsterol regulator of this degradation event (T. E. Meigs, D. S. Roseman, and R. D. Simoni, 1996, J. Biol. Chem. 271, 7916-7922). In this report, we have studied the enzyme farnesyl pyrophosphatase (FPPase) in Chinese hamster ovary cells. We demonstrate that FPPase activity increases under conditions of increased metabolic flow through the isoprenoid pathway. Also, we show that a nonhydrolyzable analog of farnesyl pyrophosphate, an isoprenoid (phosphinylmethyl)phosphonate, inhibits FPPase in vitro, and when added to cells this inhibitor blocks the mevalonate-dependent, sterol-induced degradation of HMG-CoA reductase. Furthermore, exogenous farnesol overcomes the effect of this inhibitor. These results suggest an isoprenoid-mediated regulatory mechanism governing intracellular farnesol production and support the hypothesis that farnesol is a nonsterol regulator of reductase degradation.

598 ||| 20501808 ||| (4, 0.91) ||| 4 ||| 5 ||| Distinct roles of cytochrome P450 reductase in mitomycin C redox cycling and cytotoxicity. Mitomycin c (MMC), a quinone-containing anticancer drug, is known to redox cycle and generate reactive oxygen species. A key enzyme mediating MMC redox cycling is cytochrome P450 reductase, a microsomal NADPH-dependent flavoenzyme. In the present studies, Chinese hamster ovary (CHO) cells overexpressing this enzyme (CHO-OR cells) and corresponding control cells (CHO-WT cells) were used to investigate the role of cytochrome P450 reductase in the actions of MMC. In lysates from both cell types, MMC was found to redox cycle and generate H(2)O(2); this activity was greater in CHO-OR cells (V(max) = 1.2 +/- 0.1 nmol H(2)O(2)/min/mg protein in CHO-WT cells versus 32.4 +/- 3.9 nmol H(2)O(2)/min/mg protein in CHO-OR cells). MMC was also more effective in generating superoxide anion and hydroxyl radicals in CHO-OR cells, relative to CHO-WT cells. Despite these differences in MMC redox cycling, MMC-induced cytotoxicity, as measured by growth inhibition, was similar in the two cell types (IC(50) = 72 +/- 20 nmol/L for CHO-WT and 75 +/- 23 nmol/L for CHO-OR cells), as was its ability to induce G(2)-M and S phase arrest. Additionally, in nine different tumor cell lines, although a strong correlation was observed between MMC-induced H(2)O(2) generation and cytochrome P450 reductase activity, there was no relationship between redox cycling and cytotoxicity. Hypoxia, which stabilizes MMC radicals generated by redox cycling, also had no effect on the sensitivity of tumor cells to MMC-induced cytotoxicity. These data indicate that NADPH cytochrome P450 reductase-mediated MMC redox cycling is not involved in the cytotoxicity of this chemotherapeutic agent.

599 ||| 9461625 ||| (4, 0.90) ||| 4 ||| 5 ||| Evidence for a cholesterol transport pathway from lysosomes to endoplasmic reticulum that is independent of the plasma membrane. We have studied the movement of low density lipoprotein (LDL)-derived cholesterol in cultured Chinese hamster ovary cells. Our hypothesis is that when LDL cholesterol is effluxed from lysosomes, the bulk of LDL cholesterol is mobilized to the plasma membrane, while another pathway delivers LDL cholesterol from lysosomes to acyl-CoA/cholesterol acyltransferase (ACAT) in the endoplasmic reticulum. Three lines of evidence support this model. First, LDL cholesterol transport to ACAT can be blocked without inhibiting the movement of cholesterol from lysosomes to plasma membrane or from plasma membrane to endoplasmic reticulum. Second, LDL cholesterol transport to ACAT is normal in a Chinese hamster ovary mutant with defective plasma membrane-to-ACAT movement. Third, LDL cholesterol is not diluted by the plasma membrane cholesterol pool before reaching ACAT. Our evidence supports a vesicular model of cholesterol transport from lysosomes to the endoplasmic reticulum that is independent of the plasma membrane.

600 ||| 9360982 ||| (4, 0.89) ||| 4 ||| 5 ||| Non-capacitative calcium entry in Chinese hamster ovary cells expressing the platelet-derived growth factor receptor. Platelet-derived growth factor (PDGF) is believed to produce intracellular calcium (Ca2+i) transients by inositol trisphosphate (InsP3)-mediated release of intracellular Ca2+ stores followed by "capacitative" Ca2+ entry due to emptying of these stores. We examined the roles for the phospholipase Cgamma-InsP3 pathway and the emptying of InsP3-dependent intracellular Ca2+ stores in PDGF-mediated Ca2+ entry. Intracellular Ca2+ release and Ca2+ entry were measured with fluorometric methods in Chinese hamster ovary cells expressing wild type or mutant PDGF receptors. Activation of the wild type PDGF receptor caused both intracellular "Ca2+ release, " measured in nominally 0 Ca2+ extracellular medium, and "Ca2+ entry, " measured upon addition of 2 mM Ca2+ medium. Both phases were absent in Chinese hamster ovary cells expressing a PDGF receptor mutant (Y977F,Y989F) that fails to bind phospholipase Cgamma. Blockade of the InsP3 receptor, by microinjection of single cells with low molecular weight heparin (5-50 mg/ml), blocked only Ca2+i release (following PDGF or flash photolysis of caged InsP3) and had no effect on PDGF-induced Ca2+ entry. In whole cell patch-clamp experiments, intracellular heparin also failed to block PDGF-evoked ion currents. Release of InsP3-dependent intracellular Ca2+ stores, by flash photolysis of caged InsP3, was apparently not sufficient to maximally activate Ca2+ entry. Intracellular InsP3 caused significantly less Ca2+ entry than PDGF alone. These data suggest that InsP3 alone is not sufficient to maximally activate Ca2+ entry by the capacitative pathway and that products of phosphatidylinositol 4,5-bisphosphate breakdown other than InsP3 probably play a role in PDGF-mediated Ca2+ entry.

601 ||| 9405408 ||| (4, 0.88) ||| 4 ||| 5 ||| A mammalian homolog of the yeast LCB1 encodes a component of serine palmitoyltransferase, the enzyme catalyzing the first step in sphingolipid synthesis. Serine palmitoyltransferase (SPT; EC 2.3.1.50) catalyzes the initial step dedicated to sphingolipid biosynthesis and is thought to be a key enzyme for regulating cellular sphingolipid content. For SPT activity, the yeast Saccharomyces cerevisiae requires two genes, LCB1 and LCB2. We isolated mammalian LCB1 cDNA homologs from mouse and Chinese hamster ovary (CHO) cells and an LCB2 cDNA homolog from CHO cells. The mammalian LCB1 proteins are predicted to have about 35% amino acid identity to the yeast Lcb1 protein, whereas the CHO LCB2 protein is predicted to have about 40% amino acid identity to the yeast Lcb2 protein. Northern blot analysis of mRNA isolated from various mouse tissues revealed that the tissue distribution of both LCB1 and LCB2 messengers followed a similar pattern. Transfection of an SPT-defective CHO mutant strain with a CHO LCB1-expressing plasmid restored both SPT activity and de novo sphingolipid synthesis to the wild type levels, whereas transfection of the mutant strain with a CHO LCB2-expressing plasmid did not exhibit any recovery effects, indicating that the SPT defect in the mutant cells is specifically complemented by the CHO LCB1 homolog. Furthermore, when the SPT-defective mutant cells were transfected with a plasmid encoding a His6-tagged CHO LCB1 protein, SPT activity bound to a Ni2+-immobilized resin. These results indicate that the CHO LCB1 homolog encodes a component of SPT.

602 ||| 9741690 ||| (4, 0.87) ||| 4 ||| 5 ||| Analysis of isoprenoid biosynthesis in peroxisomal-deficient Pex2 CHO cell lines. ZR-78 and ZR-82 cells are two peroxisomal-deficient Chinese hamster ovary (CHO) cell mutants. These cells lack normal peroxisomes and show reduced levels of plasmalogen synthesis and other peroxisomal functions attributed to the deficiency of peroxisomal matrix enzymes. As we have recently identified two HMG-CoA reductase proteins in CHO cells, a 97 kDa reductase localized in the ER and a 90 kDa reductase protein localized in peroxisomes, this enabled us to study the two reductase proteins for the first time in peroxisomal-deficient CHO cells. In this study we report the results of a detailed analysis of the isoprenoid biosynthetic pathway in the peroxisomal-deficient CHO cell lines ZR-78 and ZR-82. We demonstrate that total HMG-CoA reductase activity is significantly reduced in the peroxisomal-deficient cells as compared to the wild type cells. Analysis of the two reductase proteins in permeabilized cells indicated that in the ZR-78 and ZR-82 cells the 90 kDa peroxisomal reductase protein was mainly localized to the cytosol. We further show that the rates of both sterol (cholesterol) and non-sterol (dolichols) biosynthesis were significantly lower in the peroxisomal-deficient cells, when either [3H] acetate or [3H] mevalonate was used as substrate. In contrast, the rate of dolichol biosynthesis in the peroxisomal-deficient cells was similar to that of the wild type cells when incubated with [3H] farnesol. In addition, we demonstrate that the peroxisomal-deficient cells exhibited increased rates of lanosterol biosynthesis as compared to wild type cells. The results of this study provide further evidence for the essential requirement of peroxisomes for cholesterol biosynthesis as well as for dolichol production.

603 ||| 10535934 ||| (4, 0.86) ||| 4 ||| 5 ||| Isolation of a Chinese hamster ovary cell mutant defective in intramitochondrial transport of phosphatidylserine. A CHO-K1 cell mutant with a specific decrease in cellular phosphatidylethanolamine (PE) level was isolated as a variant resistant to Ro09-0198, a PE-directed antibiotic peptide. The mutant was defective in the phosphatidylserine (PS) decarboxylation pathway for PE formation, in which PS produced in the endoplasmic reticulum is transported to mitochondria and then decarboxylated by an inner mitochondrial membrane enzyme, PS decarboxylase. Neither PS formation nor PS decarboxylase activity was reduced in the mutant, implying that the mutant is defective in some step of PS transport. The transport processes of phospholipids between the outer and inner mitochondrial membrane were analyzed by use of isolated mitochondria and two fluorescence-labeled phospholipid analogs, 1-palmitoyl-2-[N-[6(7-nitrobenz-2-oxa-1, 3-diazol-4-yl)amino]caproyl]-PS (C6-NBD-PS) and C6-NBD-phosphatidylcholine (C6-NBD-PC). On incubation with the CHO-K1 mitochondria, C6-NBD-PS was readily decarboxylated to C6-NBD-PE, suggesting that the PS analog was partitioned into the outer leaflet of mitochondria and then translocated to the inner mitochondrial membrane. The rate of decarboxylation of C6-NBD-PS in the mutant mitochondria was reduced to approximately 40% of that in the CHO-K1 mitochondria. The quantity of phospholipid analogs translocated from the outer leaflet of mitochondria into inner mitochondrial membranes was further examined by selective extraction of the analogs from the outer leaflet of mitochondria. In the mutant mitochondria, the translocation of C6-NBD-PS was significantly reduced, whereas the translocation of C6-NBD-PC was not affected. These results indicate that the mutant is defective in PS transport between the outer and inner mitochondrial membrane and provide genetic evidence for the existence of a specific mechanism for intramitochondrial transport of PS.

604 ||| 18632668 ||| (4, 0.85) ||| 4 ||| 5 ||| Group IVA phospholipase A2 is necessary for the biogenesis of lipid droplets. Lipid droplets (LD) are organelles present in all cell types, consisting of a hydrophobic core of triacylglycerols and cholesteryl esters, surrounded by a monolayer of phospholipids and cholesterol. This work shows that LD biogenesis induced by serum, by long-chain fatty acids, or the combination of both in CHO-K1 cells was prevented by phospholipase A(2) inhibitors with a pharmacological profile consistent with the implication of group IVA cytosolic phospholipase A(2) (cPLA(2)alpha). Knocking down cPLA(2)alpha expression with short interfering RNA was similar to pharmacological inhibition in terms of enzyme activity and LD biogenesis. A Chinese hamster ovary cell clone stably expressing an enhanced green fluorescent protein-cPLA(2)alpha fusion protein (EGFP-cPLA(2)) displayed higher LD occurrence under basal conditions and upon LD induction. Induction of LD took place with concurrent phosphorylation of cPLA(2)alpha at Ser(505). Transfection of a S505A mutant cPLA(2)alpha showed that phosphorylation at Ser(505) is key for enzyme activity and LD formation. cPLA(2)alpha contribution to LD biogenesis was not because of the generation of arachidonic acid, nor was it related to neutral lipid synthesis. cPLA(2)alpha inhibition in cells induced to form LD resulted in the appearance of tubulo-vesicular profiles of the smooth endoplasmic reticulum, compatible with a role of cPLA(2)alpha in the formation of nascent LD from the endoplasmic reticulum.

605 ||| 11546763 ||| (4, 0.85) ||| 4 ||| 5 ||| Mitochondrial glycerol phosphate acyltransferase directs the incorporation of exogenous fatty acids into triacylglycerol. The mitochondrial isoform of glycerol-3-phosphate acyltransferase (GPAT), the first step in glycerolipid synthesis, is up-regulated by insulin and by high carbohydrate feeding via SREBP-1c, suggesting that it plays a role in triacylglycerol synthesis. To test this hypothesis, we overexpressed mitochondrial GPAT in Chinese hamster ovary (CHO) cells. When GPAT was overexpressed 3.8-fold, triacylglycerol mass was 2.7-fold higher than in control cells. After incubation with trace [(14)C]oleate ( approximately 3 microm), control cells incorporated 4.7-fold more label into phospholipid than triacylglycerol, but GPAT-overexpressing cells incorporated equal amounts of label into phospholipid and triacylglycerol. In GPAT-overexpressing cells, the incorporation of label into phospholipid, particularly phosphatidylcholine, decreased 30%, despite normal growth rate and phospholipid content, suggesting that exogenous oleate was directed primarily toward triacylglycerol synthesis. Transiently transfected HEK293 cells that expressed a 4.4-fold increase in GPAT activity incorporated 9.7-fold more [(14)C]oleate into triacylglycerol compared with control cells, showing that the effect of GPAT overexpression was similar in two different cell types that had been transfected by different methods. When the stable, GPAT-overexpressing CHO cells were incubated with 100 microm oleate to stimulate triacylglycerol synthesis, they incorporated 1.9-fold more fatty acid into triacylglycerol than did the control cells. Confocal microscopy of CHO and HEK293 cells transfected with the GPAT-FLAG construct showed that GPAT was located correctly in mitochondria and was not present elsewhere in the cell. These studies indicate that overexpressed mitochondrial GPAT directs incorporation of exogenous fatty acid into triacylglycerol rather than phospholipid and imply that (a) mitochondrial GPAT and lysophosphatidic acid acyltransferase produce a separate pool of lysophosphatidic acid and phosphatidic acid that must be transported to the endoplasmic reticulum where the terminal enzymes of triacylglycerol synthesis are located, and (b) this pool remains relatively separate from the pool of lysophosphatidic acid and phosphatidic acid that contributes to the synthesis of the major phospholipid species.

606 ||| 17981807 ||| (4, 0.84) ||| 4 ||| 5 ||| Endogenous 24(S),25-epoxycholesterol fine-tunes acute control of cellular cholesterol homeostasis. Certain oxysterols, when added to cultured cells, are potent regulators of cholesterol homeostasis, decreasing cholesterol synthesis and uptake and increasing cholesterol efflux. However, very little is known about whether or not endogenous oxysterol(s) plays a significant role in cholesterol homeostasis. 24(S),25-Epoxycholesterol (24,25EC) is unique among oxysterols in that it is produced in a shunt of the mevalonate pathway which also produces cholesterol. We investigated the role of endogenously produced 24,25EC using a novel strategy of overexpressing the enzyme 2,3-oxidosqualene cyclase in Chinese hamster ovary cells to selectively inhibit the synthesis of this oxysterol. First, loss of 24,25EC decreased expression of the LXR target gene, ABCA1, substantiating its role as an endogenous ligand for LXR. Second, loss of 24,25EC increased acute cholesterol synthesis, which was rationalized by a concomitant increase in HMG-CoA reductase gene expression at the level of SREBP-2 processing. Therefore, in the absence of 24,25EC, fine-tuning of the acute regulation of cholesterol homeostasis is lost, supporting the hypothesis that 24,25EC functions to protect the cell against the accumulation of newly synthesized cholesterol.

607 ||| 10037789 ||| (4, 0.84) ||| 4 ||| 5 ||| Genetic evidence for ATP-dependent endoplasmic reticulum-to-Golgi apparatus trafficking of ceramide for sphingomyelin synthesis in Chinese hamster ovary cells. LY-A strain is a Chinese hamster ovary cell mutant resistant to sphingomyelin (SM)-directed cytolysin and has a defect in de novo SM synthesis. Metabolic labeling experiments with radioactive serine, sphingosine, and choline showed that LY-A cells were defective in synthesis of SM from these precursors, but not syntheses of ceramide (Cer), glycosphingolipids, or phosphatidylcholine, indicating a specific defect in the conversion of Cer to SM in LY-A cells. In vitro experiments showed that the specific defect of SM formation in LY-A cells was not due to alterations in enzymatic activities responsible for SM synthesis or degradation. When cells were treated with brefeldin A, which causes fusion of the Golgi apparatus with the endoplasmic reticulum (ER), de novo SM synthesis in LY-A cells was restored to the wild-type level. Pulse-chase experiments with a fluorescent Cer analogue, N-(4,4-difluoro-5,7-dimethyl-4-bora-3a, 4a-diaza-s-indacene-3-pentanoyl)-D-erythro-sphingosine (C5-DMB-Cer), revealed that in wild-type cells C5-DMB-Cer was redistributed from intracellular membranes to the Golgi apparatus in an intracellular ATP-dependent manner, and that LY-A cells were defective in the energy-dependent redistribution of C5-DMB-Cer. Under ATP-depleted conditions, conversion of C5-DMB-Cer to C5-DMB-SM and of [3H]sphingosine to [3H]SM in wild-type cells decreased to the levels in LY-A cells, which were not affected by ATP depletion. ER-to-Golgi apparatus trafficking of glycosylphosphatidylinositol-anchored or membrane-spanning proteins in LY-A cells appeared to be normal. These results indicate that the predominant pathway of ER-to-Golgi apparatus trafficking of Cer for de novo SM synthesis is ATP dependent and that this pathway is almost completely impaired in LY-A cells. In addition, the specific defect of SM synthesis in LY-A cells suggests different pathways of Cer transport for glycosphingolipids versus SM synthesis.

608 ||| 9642289 ||| (4, 0.83) ||| 4 ||| 5 ||| Genetic evidence that phosphatidylserine synthase II catalyzes the conversion of phosphatidylethanolamine to phosphatidylserine in Chinese hamster ovary cells. Phosphatidylserine (PS) in mammalian cells is synthesized through the exchange of free L-serine with the base moiety of phosphatidylcholine or phosphatidylethanolamine (PE). The serine base exchange in Chinese hamster ovary (CHO) cells is catalyzed by at least two enzymes, PS synthase (PSS) I and II. A PSS I-lacking mutant of CHO-K1 cells, PSA-3, which exhibits approximately 2-fold lower serine base exchange activity than CHO-K1, is defective in the conversion of phosphatidylcholine to PS but has the ability to convert PE to PS. The PSA-3 mutant requires exogenous PS or PE for cell growth. In the present study, from PSA-3 mutant cells, we isolated a mutant, named PSB-2, with a further decrease in the serine base exchange activity. The activity in the homogenate of PSB-2 mutant cells was approximately 10% that of PSA-3 mutant cells and approximately 5% that of CHO-K1 cells. The PSB-2 mutant exhibited an approximately 80% reduction in the PSS II mRNA level relative to that in PSA-3 mutant and CHO-K1 cells. These results showed that the PSB-2 mutant is defective in PSS II. Like the PSA-3 mutant, the PSB-2 mutant grew well in medium supplemented with PS. However, in the medium supplemented with PE, the PSB-2 mutant was incapable of growth, in contrast to the PSA-3 mutant. In the medium with exogenous PE, the PSB-2 mutant was defective in PS biosynthesis, whereas the PSA-3 mutant synthesized a normal amount of PS. A metabolic labeling experiment with exogenous [32P]PE revealed that the PSB-2 mutant was defective in the conversion of exogenous PE to PS. This defect and the growth and PS biosynthetic defects of the PSB-2 mutant cultivated with exogenous PE were complemented by the PSS II cDNA. In addition, the cDNA of the other PS synthase, PSS I, was shown not to complement the defect in the conversion of exogenous PE to PS of the PSB-2 mutant, implying that PSS I negligibly contributes to the conversion of PE to PS in CHO-K1 cells. These results indicated that PSS II is critical for the growth and PS biosynthesis of PSA-3 mutant cells cultivated with exogenous PE and suggested that most of the PS formation from PE in CHO-K1 cells is catalyzed by PSS II.

609 ||| 9388473 ||| (4, 0.82) ||| 4 ||| 5 ||| Nociceptin/orphanin FQ activates protein kinase C, and this effect is mediated through phospholipase C/Ca2+ pathway. The effect of nociceptin/orphanin FQ (N/OFQ) on protein kinase C (PKC) was investigated in Chinese hamster ovary cells stably expressing opioid receptor-like (ORL1) receptor (CHO-ORL1 cells). N/OFQ significantly activated PKC in CHO-ORL1 cells with EC50 of 0.2 nM. This response was blocked by PKC inhibitors chelerythrine and Go 6976, and by pretreatment of cells with pertussis toxin (PTX). The inhibition of PKC activation by N/OFQ was also achieved by use of Ca(2+)-chelators and phospholipase C (PLC) inhibitor U-73122. These results indicate that N/OFQ can effectively activate PKC via ORL1 receptor, and suggest the activation involve the PLC/Ca2+ system.

610 ||| 17558022 ||| (4, 0.82) ||| 4 ||| 5 ||| De novo biosynthesis of the late endosome lipid, bis(monoacylglycero)phosphate. Bis(monoacylglycero)phosphate (BMP) is a unique lipid enriched in the late endosomes participating in the trafficking of lipids and proteins through this organelle. The de novo biosynthesis of BMP has not been clearly demonstrated. We investigated whether phosphatidylglycerol (PG) and cardiolipin (CL) could serve as precursors of de novo BMP synthesis using two different cellular models: CHO cells deficient in phosphatidylglycerophosphate (PGP) synthase, the enzyme responsible for the first step of PG synthesis; and human lymphoblasts from patients with Barth syndrome (BTHS), characterized by mutations in tafazzin, an enzyme implicated in the deacylation-reacylation cycle of CL. The biosynthesis of both PG and BMP was reduced significantly in the PGP synthase-deficient CHO mutants. Furthermore, overexpression of PGP synthase in the deficient mutants induced an increase of BMP biosynthesis. In contrast to CHO mutants, BMP biosynthesis and its fatty acid composition were not altered in BTHS lymphoblasts. Our results thus suggest that in mammalian cells, PG, but not CL, is a precursor of the de novo biosynthesis of BMP. Despite the decrease of de novo synthesis, the cellular content of BMP remained unchanged in CHO mutants, suggesting that other pathway(s) than de novo biosynthesis are also used for BMP synthesis.

611 ||| 12767923 ||| (4, 0.81) ||| 4 ||| 5 ||| An apparent decrease in cholesterol biosynthesis in peroxisomal-defective Chinese hamster ovary cells is related to impaired mitochondrial oxidation. Recent data suggest that impaired mitochondrial activities in Zellweger fibroblasts are related to defective peroxisome biogenesis and vice versa. To investigate the contribution of functional mitochondria to cholesterol biosynthesis, radioactive precursor molecules that form acetyl-CoA via beta-oxidation-independent (pyruvate) or -dependent (palmitate and octanoate) pathways were used. Production of both 14C-labeled cholesterol and 14C-labeled CO(2) from these radioactive tracers was significantly impaired in peroxisomal-defective ZR-82 Chinese hamster ovary cells in comparison to controls. In contrast, cholesterol synthesis from acetate--a tracer directly converted to acetyl-CoA without the involvement of mitochondrial activities--was threefold higher in ZR-82 cells than in controls. Pathways further contributing to cellular cholesterol homeostasis, i.e., receptor-mediated binding of exogenous lipoprotein-associated cholesterol as well as intracellular mobilization of cholesteryl ester deposits were similar in ZR-82 and controls. From these findings, we propose that peroxisomal dysfunction in ZR-82 cells is tightly coupled to impaired mitochondrial activities, e.g., defective mitochondrial beta-oxidation and formation of acetyl-CoA from short chain fatty acids resulting in a decreased rate of CO(2) production, and an apparent decrease in cholesterol biosynthesis. Actually, cholesterol biosynthesis from acetate is increased in the peroxisomal-defective cells. This explains previous conflicting conclusions.

612 ||| 10395967 ||| (4, 0.81) ||| 4 ||| 5 ||| Regulation of phosphatidylcholine homeostasis by calcium-independent phospholipase A2. Phosphatidylcholine (PtdCho) is the most abundant phospholipid in mammalian cell membranes and is essential for cell viability. The levels of this lipid must be tightly controlled to maintain homeostasis. Therefore, changes in the rate of PtdCho synthesis are generally balanced by changes in PtdCho catabolism and vice versa. It is commonly accepted that the rate of PtdCho synthesis is regulated by CTP:phosphocholine cytidylyltransferase (CT). However, it is not certain if PtdCho mass is regulated by specific catabolic enzyme(s). Our goal is to determine if PtdCho homeostasis is regulated by a phospholipase A2 (PLA2). To this end, we have prepared Chinese hamster ovary (CHO) cell lines that overexpress CT. CT activity is 7-10-fold higher in the transfected cells than in parental CHO cells. This increase in CT activity is associated with increases in both PtdCho synthesis and PtdCho catabolism. Glycerophosphocholine is the PtdCho catabolite that accumulates in the transfected cells, which suggests that PtdCho turnover is mediated by a phospholipase A2 (PLA2). Indeed, higher levels of calcium-independent PLA2 activity are measured in the cytosols of the CHO cells that overexpress CT, compared to parental CHO cells. The elevated calcium-independent PLA2 activity is associated with increases in the expression of the 80-kDa calcium-independent PLA2 (iPLA2). Together, these data suggest that the 80-kDa iPLA2 may be modulated in response to changes in PtdCho levels and therefore is involved in the regulation of PtdCho homeostasis in CHO cells.

613 ||| 10882735 ||| (4, 0.80) ||| 4 ||| 5 ||| Reconstitution of ATP- and cytosol-dependent transport of de novo synthesized ceramide to the site of sphingomyelin synthesis in semi-intact cells. Transport of ceramide synthesized at the endoplasmic reticulum to the Golgi compartment, where sphingomyelin (SM) synthase exists, was reconstituted within semi-intact Chinese hamster ovary cells. When [(3)H]ceramide that had been produced from [(3)H]sphingosine at 15 degrees C in perforated cells was chased at 37 degrees C, [(3)H]ceramide-to-[(3)H]SM conversion occurred in a cytosol-dependent manner. In various aspects (i.e. kinetics, ATP dependence, and temperature dependence), [(3)H]ceramide-to-[(3)H]SM conversion in perforated cells was consistent with that in intact cells. The cytosol from LY-A strain, a Chinese hamster ovary cell mutant defective in endoplasmic reticulum-to-Golgi transport of ceramide, did not support [(3)H]ceramide-to-[(3)H]SM conversion in perforated wild-type cells, whereas the wild-type cytosol rescued the conversion in perforated LY-A cells. Brefeldin A-treated cells, in which the endoplasmic reticulum and the Golgi apparatus were merged, no longer required cytosol for conversion of [(3)H]ceramide to [(3)H]SM. These results indicated that the assay of [(3)H]ceramide-to-[(3)H]SM conversion in semi-intact cells is a faithful in vitro assay for the activity of cytosol-dependent transport of ceramide and that LY-A cells are defective in a cytosolic factor involved in ceramide transport. In addition, conversion of [(3)H]ceramide to [(3)H]glucosylceramide in semi-intact cells was little dependent on cytosol, suggesting that ceramide reached the site of glucosylceramide synthesis by a cytosol-independent (or less dependent) pathway.

614 ||| 10471370 ||| (4, 0.80) ||| 4 ||| 5 ||| Diethanolamine inhibits choline uptake and phosphatidylcholine synthesis in Chinese hamster ovary cells. Diethanolamine (DEA), an alkanolamine used widely in industry, is hepatocarcinogenic in mice. The goal of this work was to determine whether DEA altered choline homeostasis in cultured cells, so as to ascertain whether the liver tumor response may be related to choline deficiency. CHO cells were cultured in Ham's F-12 medium containing DEA (0-1000 microgram/ml) and [(33)P]-phosphorus was used to label phospholipid pools. After 48 hours incubation, lipids were extracted and [(33)P]-labeled phospholipids were quantified by autoradiography after thin layer chromatographic separation. In control cells, phosphatidylcholine (PC) accounted for 51 +/- 0.7% of the total lipid (33)P incorporation. DEA had no effect on cell number or total phospholipid biosynthesis, but it significantly decreased the incorporation of (33)P into PC at concentrations >/=50 microgram/ml. DEA (>/=20 microgram/ml) also inhibited the uptake of [(3)H]-choline into CHO cells, with 95% inhibition observed at 250 microgram/ml. To determine whether supplemental choline prevented PC synthesis inhibition by DEA, CHO cells were cultured with or without excess choline (30 mM) and DEA (500 microgram/ml). DEA reduced PC synthesis to 27 +/- 3% of total phospholipids, but had no effect on PC synthesis in choline-supplemented cells. When [(14)C]-DEA was incubated with CHO cells, it was also incorporated into the phospholipid fraction. Collectively, these results indicate that DEA reversibly inhibits PC synthesis by blocking choline uptake and competing for utilization in the CDP-choline pathway in CHO cells.

615 ||| 20888376 ||| (4, 0.78) ||| 4 ||| 5 ||| SNARE-mediated membrane traffic is required for focal adhesion kinase signaling and Src-regulated focal adhesion turnover. Integrin signaling is central to cell growth and differentiation, and critical for the processes of apoptosis, cell migration and wound repair. Previous research has demonstrated a requirement for SNARE-dependent membrane traffic in integrin trafficking, as well as cell adhesion and migration. The goal of the present research was to ascertain whether SNARE-dependent membrane trafficking is required specifically for integrin-mediated signaling. Membrane traffic was inhibited in Chinese hamster ovary cells by expression of dominant-negative (E329Q) N-ethylmaleimide-sensitive fusion protein (NSF) or a truncated form of the SNARE SNAP23. Integrin signaling was monitored as cells were plated on fibronectin under serum-free conditions. E329Q-NSF expression inhibited phosphorylation of focal adhesion kinase (FAK) on Tyr397 at early time points of adhesion. Phosphorylation of FAK on Tyr576, Tyr861 and Tyr925 was also impaired by expression of E329Q-NSF or truncated SNAP23, as was trafficking, localization and activation of Src and its interaction with FAK. Decreased FAK-Src interaction coincided with reduced Rac activation, decreased focal adhesion turnover, reduced Akt phosphorylation and lower phosphatidylinositol 3,4,5-trisphosphate levels in the cell periphery. Over-expression of plasma membrane-targeted Src or phosphatidylinositol 3-kinase (PI3K) rescued cell spreading and focal adhesion turnover. The results suggest that SNARE-dependent trafficking is required for integrin signaling through a FAK/Src/PI3K-dependent pathway.

616 ||| 11160369 ||| (4, 0.78) ||| 4 ||| 5 ||| Diacylglycerol generated in CHO cell plasma membrane by phospholipase C is used for triacylglycerol synthesis. The diacylglycerol (DAG) signal generated from membrane phospholipids by hormone-activated phospholipases is attenuated by mechanisms that include lipolysis or phospholipid resynthesis. To determine whether the DAG signal might also be terminated by incorporation of DAG into triacylglycerol (TAG), we studied the direct formation of TAG from endogenous DAG generated by bacterial phospholipase C (PLC). When Chinese hamster ovary (CHO) cells prelabeled with [(14)C]oleate were treated with PLC from Clostridium perfringens for 6 h, [(14)C]phospholipid decreased 15% and labeled TAG increased 60%. This transfer of (14)C label was even greater when the cells were simultaneously exposed to PLC and 100 microM oleic acid. PLC as well as oleate treatment concomitantly increased the TAG mass within the cell. Moreover, when phospholipids were prelabeled with [(3)H]glycerol, a subsequent increase in [(3)H]TAG indicated that an intact DAG moiety was channeled into the TAG structure. Incubating CHO cells with the diacylglycerol kinase inhibitor R59022 enhanced the formation of TAG from phospholipids hydrolyzed by PLC or by PLC in the presence of 100 microM oleate, but not by incubation with oleate alone, indicating that the DAG released from plasma membrane phospholipids does not require the formation of a phosphatidic acid precursor for TAG synthesis. Similarly, the diacylglycerol lipase inhibitor RHC 80267 did not alter TAG synthesis from plasma membrane DAG, further supporting direct incorporation of DAG into TAG. These studies indicate that DAG derived from plasma membrane phospholipid is largely used for TAG formation, and support the view that this mechanism can terminate DAG signals. The studies also suggest that a transport mechanism exists to move plasma membrane-derived DAG to the endoplasmic reticulum.-Igal, R. A., J. M. Caviglia, I. N. T. de Gomez Dumm, and R. A. Coleman. Diacylglycerol generated in CHO cell plasma membrane by phospholipase C is used for triacylglycerol synthesis. J. Lipid Res. 2001. 42: 88;-95.

617 ||| 10497220 ||| (4, 0.78) ||| 4 ||| 5 ||| Failure to cleave sterol regulatory element-binding proteins (SREBPs) causes cholesterol auxotrophy in Chinese hamster ovary cells with genetic absence of SREBP cleavage-activating protein. We describe a line of mutant Chinese hamster ovary cells, designated SRD-13A, that cannot cleave sterol regulatory element-binding proteins (SREBPs) at site 1, due to mutations in the gene encoding SREBP cleavage-activating protein (SCAP). The SRD-13A cells were obtained by two rounds of gamma-irradiation followed first by selection for a deficiency of low density lipoprotein receptors and second for cholesterol auxotrophy. In the SRD-13A cells, the only detectable SCAP allele encodes a truncated nonfunctional protein. In the absence of SCAP, the site 1 protease fails to cleave SREBPs, and their transcriptionally active NH(2)-terminal fragments cannot enter the nucleus. As a result, the cells manifest a marked reduction in the synthesis of cholesterol and its uptake from low density lipoproteins. The SRD-13A cells grow only when cholesterol is added to the culture medium. SREBP cleavage is restored and the cholesterol requirement is abolished when SRD-13A cells are transfected with expression vectors encoding SCAP. These results provide formal proof that SCAP is essential for the cleavage of SREBPs at site 1.

618 ||| 9727033 ||| (4, 0.77) ||| 4 ||| 5 ||| Newly identified Chinese hamster ovary cell mutants are defective in biogenesis of peroxisomal membrane vesicles (Peroxisomal ghosts), representing a novel complementation group in mammals. We isolated peroxisome biogenesis-defective mutants from Chinese hamster ovary cells by the 9-(1'-pyrene)nonanol/ultraviolet (P9OH/UV) method. Seven cell mutants, ZP116, ZP119, ZP160, ZP161, ZP162, ZP164, and ZP165, of 11 P9OH/UV-resistant cell clones showed cytosolic localization of catalase, a peroxisomal matrix enzyme, apparently indicating a defect of peroxisome biogenesis. By transfection of PEX cDNAs and cell fusion analysis, mutants ZP119 and ZP165 were found to belong to a novel complementation group (CG), distinct from earlier mutants. CG analysis by cell fusion with fibroblasts from patients with peroxisome biogenesis disorders such as Zellweger syndrome indicated that ZP119 and ZP165 were in the same CG as the most recently identified human CG-J. The peroxisomal matrix proteins examined, including PTS1 proteins as well as a PTS2 protein, 3-ketoacyl-CoA thiolase, were also found in the cytosol in ZP119 and ZP165. Furthermore, these mutants showed typical peroxisome assembly-defective phenotype such as severe loss of resistance to 12-(1'-pyrene)dodecanoic acid/UV treatment. Most strikingly, peroxisomal reminiscent vesicular structures, so-called peroxisomal ghosts noted in all CGs of earlier Chinese hamster ovary cell mutants as well as in eight CGs of patients' fibroblasts, were not discernible in ZP119 and ZP165, despite normal synthesis of peroxisomal membrane proteins. Accordingly, ZP119 and ZP165 are the first cell mutants defective in import of both soluble and membrane proteins, representing the 14th peroxisome-deficient CG in mammals, including humans.

619 ||| 9731498 ||| (4, 0.76) ||| 4 ||| 5 ||| Lysosomal sequestration of polyamine analogues in Chinese hamster ovary cells resistant to the S-adenosylmethionine decarboxylase inhibitor, CGP-48664. CGP-48664, an inhibitor of the polyamine biosynthetic enzyme S-adenosylmethionine decarboxylase (AdoMetDC), is presently undergoing Phase 1 clinical trials as an experimental anticancer agent. We have shown previously (D. L. Kramer et al., J. Biol. Chem., 270: 2124-2132, 1995) that Chinese hamster ovary (CHO) cells that are made resistant to the growth inhibitory effects of the drug overexpress AdoMetDC because of a stable gene amplification. Unexpectedly, these same cells (CHO/644) were found to be insensitive to the growth inhibitory effects of N1,N11-diethylnorspermine (DENSPM)-a polyamine analogue also undergoing Phase 1 clinical trials-despite accumulating approximately 5 times more analogue than parental cells. We now report that treatment of CHO/664 cells with DENSPM results in the formation of numerous large cytoplasmic vacuoles, which on the basis of electron microscopy and cytochemical staining seem to be lysosomal in origin. A series of newly established CHO cell lines made differentially resistant to 1, 3, 10, 30, and 100 microM CGP-48664 by chronic exposure were used to demonstrate that vacuole formation correlated with the accumulation of extremely high levels of DENSPM without increasing growth inhibition. These same cells were used to show that AdoMetDC gene overexpression as indicated by mRNA levels was unrelated to vacuole formation; cells resistant to 100 microM CGP-48664 displayed a 170-fold increase in AdoMetDC mRNA levels and formed vacuoles in response to DENSPM, whereas those resistant to 10 microM CGP-48664 displayed a 120-fold increase in AdoMetDC mRNA levels and failed to form vacuoles. Despite accumulating to high intracellular levels, DENSPM was much less effective than spermine at down-regulating ornithine decarboxylase and polyamine transport activities in highly resistant cells. Similarly, DENSPM was less able to induce spermidine/spermine N1-acetyltransferase activity in cells that formed vacuoles than in those that did not. Overall, natural polyamines failed to induce vacuoles and various analogues of DENSPM were used to probe the structural specificity of the effect. The data are consistent with the probability that DENSPM is sequestered to high concentrations in lysosomal vacuoles of CGP-48664-resistant cells and is, therefore, not available to interact with polyamine regulatory sites or to cytotoxically affect cell growth. In addition to implicating the lysosome as a potential new site of CGP-48664 drug action that could be involved in antitumor activity and/or host toxicities, the findings also suggest a potential mechanism of cell resistance to analogues such as DENSPM.

620 ||| 7742354 ||| (4, 0.75) ||| 4 ||| 5 ||| Inhibition of acyl-CoA:cholesterol acyltransferase in Chinese hamster ovary (CHO) cells by short-chain ceramide and dihydroceramide. The biological activity of ceramide, an intermediate in the synthesis and catabolism of sphingolipids, has been shown to be mimicked by short-chain N-acyl analogues. A potential role for ceramide in modulating cholesterol esterification was investigated using a series of short-chain ceramides and dihydroceramides. Acyl-CoA:cholesterol acyltransferase (ACAT) in CHO cells was inhibited rapidly (< 30 min) and in a dose-dependent fashion by two N-acyl analogues of naturally occurring D-erythro-ceramide, N-acetyl-sphingosine (D-erythro-C2-ceramide) and N-hexanoyl-sphingosine (D-erythro-C6-ceramide). At 10 microM D-erythro-C2-ceramide, esterification of cholesterol was inhibited by 95% in CHO cells grown in delipidated serum, and 80-85% in cells grown in 25-hydroxycholesterol or human low-density lipoprotein (LDL). D-erythro-C2-Ceramide did not inhibit [14C]oleate-labelling of triacylglycerol and phospholipid. Inhibition of cholesterol esterification in cells and isolated membranes required the D-erythro (2S,3R) configuration (the L-threo isomer of C2-ceramide was not inhibitory) and an N-acyl group (sphingosine and sphinganine did not inhibit). DL-erythro-C2-Dihydroceramide was also a potent ACAT inhibitor in isolated membranes (IC50 0.2 microM) and cells indicating lack of requirement for a 4-trans double bond. Consistent with results for C2-ceramides, DL-threo-C2-dihydroceramide was not inhibitory in cells or in vitro. Long-chain ceramide and N-palmitoyl-dihydroceramide did not inhibit ACAT in isolated membranes. Compared to D-erythro-C2-ceramide, D-erythro-C6- and C4-ceramide were slightly weaker inhibitors of ACAT in isolated membranes. Thus, N-acyl chain length could influence inhibition, either by altering the effective concentration of ceramide in membranes or affinity for the ACAT enzyme. Short-chain ceramides and dihydroceramides are the first ACAT inhibitors described with structural similarity to a naturally occurring compound.

621 ||| 18946045 ||| (4, 0.75) ||| 4 ||| 5 ||| Transport of LDL-derived cholesterol from the NPC1 compartment to the ER involves the trans-Golgi network and the SNARE protein complex. Mammalian cells acquire cholesterol mainly from LDL. LDL enter the endosomes, allowing cholesteryl esters to be hydrolyzed by acid lipase. The hydrolyzed cholesterol (LDL-CHOL) enters the Niemann-Pick type C1 (NPC1)-containing endosomal compartment en route to various destinations. Whether the Golgi is involved in LDL-CHOL transport downstream of the NPC1 compartment has not been demonstrated. Using subcellular fractionation and immunoadsorption to enrich for specific membrane fractions, here we show that, when parental Chinese hamster ovary (CHO) cells are briefly exposed to (3)H-cholesteryl linoleate (CL) labeled-LDL, newly liberated (3)H-LDL-CHOL appears in membranes rich in trans-Golgi network (TGN) long before it becomes available for re-esterification at the endoplasmic reticulum (ER) or for efflux at the plasma membrane. In mutant cells lacking NPC1, the appearance of newly liberated (3)H-LDL-CHOL in the TGN-rich fractions is much reduced. We next report a reconstituted transport system that recapitulates the transport of LDL-CHOL to the TGN and to the ER. The transport system requires ATP and cytosolic factors and depends on functionality of NPC1. We demonstrate that knockdown by RNAi of 3 TGN-specific SNAREs (VAMP4, syntaxin 6, and syntaxin 16) reduces >/=50% of the LDL-CHOL transport in intact cells and in vitro. These results show that vesicular trafficking is involved in transporting a significant portion of LDL-CHOL from the NPC1-containing endosomal compartment to the TGN before its arrival at the ER.

622 ||| 9869656 ||| (4, 0.73) ||| 4 ||| 5 ||| Chinese hamster ovary cells overexpressing the oxysterol binding protein (OSBP) display enhanced synthesis of sphingomyelin in response to 25-hydroxycholesterol. 25-Hydroxycholesterol negatively regulates cholesterol synthesis and activates cholesterol esterification in a variety of cultured cells. Concurrent with these effects, 25-hydroxycholesterol also stimulates the synthesis of sphingomyelin in Chinese hamster ovary (CHO)-K1 cells. The role of oxysterol binding protein (OSBP), a high affinity receptor for 25-hydroxycholesterol, in activation of SM synthesis was assessed by overexpression in CHO-K1 cells. When compared to mock transfected controls, three CHO-K1 clones overexpressing OSBP by 10- to 15-fold displayed a 2- to 3-fold enhancement of [3H]serine incorporation into sphingomyelin when treated with 25-hydroxycholesterol. Closer examination of one of these clones (CHO-OSBP cells) revealed a >8.5-fold stimulation of sphingomyelin synthesis after a 6-h treatment with 25-hydroxycholesterol compared to 3.5-fold in controls, slightly higher basal levels of sphingomyelin synthesis, and a more rapid response to 25-hydroxycholesterol. [3H]serine incorporation into phosphatidylserine, phosphatidylethanolamine, ceramide, or glucosylceramide was affected by <15%. Synthesis of sphingomyelin from exogenous [3H]sphinganine-labeled ceramide was enhanced in overexpressing cells treated with 25-hydroxycholesterol. However, in vitro activities of sphinganine N-acyltransferase, sphingomyelin synthase, and serine palmitoyltransferase were not affected by OSBP overexpression or 25-hydroxycholesterol. Overexpression of OSBP or 25-hydroxycholesterol did not significantly affect the ceramide content of Golgi-enriched fractions from control or overexpressing cells. However, diglyceride mass was reduced in Golgi-enriched fractions from overexpressing cells and by treatment with 25-hydroxycholesterol. Results from overexpressing cells show that OSBP potentiates the stimulatory effects of 25-hydroxycholesterol on sphingomyelin synthesis. 25-Hydroxycholesterol promotes translocation of OSBP to the Golgi apparatus where it appears to stimulate conversion of ceramide to sphingomyelin.

623 ||| 8898195 ||| (4, 0.73) ||| 4 ||| 5 ||| Sterol resistance in CHO cells traced to point mutation in SREBP cleavage-activating protein. Through expression cloning we have isolated a cDNA-encoding SREBP cleavage-activating protein (SCAP), which regulates cholesterol metabolism by stimulating cleavage of transcription factors SREBP-1 and -2, thereby releasing them from membranes. The cDNA was isolated from Chinese hamster ovary cells with a dominant mutation that renders them resistant to sterol-mediated suppression of cholesterol synthesis and uptake. Sterol resistance was traced to a G-->A transition at codon 443 of SCAP, changing aspartic acid to asparagine. The D443N mutation enhances the cleavage-stimulating ability of SCAP and renders it resistant to inhibition by sterols. SCAP has multiple membrane-spanning regions, five of which resemble the sterol-sensing domain of HMG CoA reductase, an endoplasmic reticulum enzyme whose degradation is accelerated by sterols. SCAP appears to be a central regulator of cholesterol metabolism in animal cells.

624 ||| 7666011 ||| (4, 0.73) ||| 4 ||| 5 ||| 25-Hydroxycholesterol stimulates sphingomyelin synthesis in Chinese hamster ovary cells. In several experimental and pathological conditions the cellular concentrations of cholesterol and sphingomyelin (SM) change coordinately. In an effort to identify factors mediating co-regulation, a class of suppressors of cholesterol synthesis, generically termed oxysterols, were tested for effects on sphingolipid synthesis in Chinese hamster ovary (CHO) cells. 25-Hydroxycholesterol was found to stimulate [3H]serine, [1-3H]palmitate, and [methyl-3H]choline incorporation into sphingomyelin 2- to 3-fold and increase sphingomyelin mass significantly, but did not influence synthesis of other major phospholipids. Maximal labeling of sphingomyelin by [3H]serine was observed 4-6 h after oxysterol addition, and coincided with inhibition of transcription of sterol-regulated genes and activation of cholesteryl ester synthesis. 25-Hydroxycholesterol dose-response curves for activation of sphingomyelin synthesis, suppression of sterol-regulated transcription, and activation of cholesteryl ester synthesis were also similar. Stimulation of SM and glucosylceramide synthesis was observed only with 25-hydroxycholesterol; other oxysterols and cholesterol were ineffective or inhibitory. The effects of 25-hydroxycholesterol on sphingolipid synthesis could not be reproduced by low density lipoprotein (LDL), whole serum, or non-lipoprotein cholesterol in the medium, and stimulation by 25-hydroxycholesterol was evident irrespective of a cholesterol source in the medium. 25-Hydroxycholesterol-treated CHO cells displayed enhanced conversion of [3H]sphinganine-labeled ceramide into sphingomyelin. Sphingomyelin synthesis from N-hexanoyl [3-3H]ceramide and N-hexanoyl [3-3H]dihydroceramide was also increased significantly. Consistent with enhanced ceramide conversion to sphingomyelin, ceramide mass was reduced by 20-40% in 25-hydroxycholesterol-treatment. However, in vitro activity of sphingomyelin synthase (assayed with short-chain ceramide) was not increased in membranes from oxysterol-treated cells. Stimulation of sphingolipid synthesis by 25-hydroxycholesterol is temporally related to effects of this oxysterol on cholesterol metabolism, and is the result of enhanced conversion of ceramide to SM.

625 ||| 11546801 ||| (4, 0.73) ||| 4 ||| 5 ||| A novel inhibitor of ceramide trafficking from the endoplasmic reticulum to the site of sphingomyelin synthesis. Ceramide produced at the endoplasmic reticulum (ER) is transported to the lumen of the Golgi apparatus for conversion to sphingomyelin (SM). N-(3-Hydroxy-1-hydroxymethyl-3-phenylpropyl)dodecanamide (HPA-12) is a novel analog of ceramide. Metabolic labeling experiments showed that HPA-12 inhibits conversion of ceramide to SM, but not to glucosylceramide, in Chinese hamster ovary cells. Cultivation of cells with HPA-12 significantly reduced the content of SM. HPA-12 did not inhibit the activity of SM synthase. The inhibition of SM formation by HPA-12 was abrogated when the Golgi apparatus was made to merge with the ER by brefeldin A. Moreover, HPA-12 inhibited redistribution of a fluorescent analog of ceramide, N-(4,4-difluoro-5,7-dimethyl-4-bora-3a,4a-diaza-s-indacene-3-pentanoyl)-d-erythro-sphingosine (C(5)-DMB-Cer), from intracellular membranes to the Golgi region. Among four stereoisomers of the drug, (1R,3S)-HPA-12, [corrected] which resembles natural ceramide stereochemically, was found to be the most active, although (1R,3S)-HPA-12 [corrected] did not affect ER-to-Golgi trafficking of protein. Interestingly, (1R,3S)-HPA-12 [corrected] inhibited conversion of ceramide to SM little in mutant cells defective in an ATP- and cytosol-dependent pathway of ceramide transport. These results indicated that (1R,3S)-HPA-12 [corrected] inhibits ceramide trafficking from the ER to the site of SM synthesis, possibly due to an antagonistic interaction with a ceramide-recognizing factor(s) involved in the ATP- and cytosol-dependent pathway.

626 ||| 10484604 ||| (4, 0.73) ||| 4 ||| 5 ||| Differential binding of proteins to peroxisomes in rat hepatoma cells: unique association of enzymes involved in isoprenoid metabolism. Farnesyl diphosphate synthase (FPPS: EC2.5.1.10), a key enzyme in isoprenoid metabolic pathways, catalyzes the synthesis of farnesyl diphosphate (FPP) an intermediate in the biosynthesis of both sterol and non-sterol isoprenoid end products. The localization of FPPS to peroxisomes has been reported (Krisans, S. K., J. Ericsson, P. A. Edwards, and G. A. Keller. 1994. J. Biol. Chem. 269: 14165;-14169). Using indirect immunofluorescence and immunoelectron microscopic techniques we show here that FPPS is localized predominantly in the peroxisomes of rat hepatoma H35 cells. However, the partial release of 60;-70% of cellular FPPS activity is observed by selective permeabilization of these cells with digitonin. Under these conditions, lactate dehydrogenase, a cytosolic enzyme, is completely released whereas catalase, a known peroxisomal enzyme, is fully retained. Digitonin treatment of H35 cells differentially affects the release of other peroxisomal enzymes involved in isoprenoid metabolism. For instance, mevalonate kinase and phosphomevalonate kinase are almost totally released (95% and 91%, respectively), whereas 3-hydroxy-3-methylglutaryl-CoA reductase is fully retained. Indirect immunoflourescence studies indicate that FPPS is localized in peroxisomes of Chinese hamster ovary (CHO)-K1 cells but is dispersed in the cytosol of ZR-82 cells, a mutant that lacks peroxisomes. Unlike in H35 cells, FPPS is completely released upon digitonin permeabilization of CHO-K1 and ZR-82 cells. In contrast, under the same permeabilization conditions, catalase is fully retained in CHO-K1 cells but completely released from ZR-82 cells. These studies indicate that FPPS and other enzymes in the isoprenoid biosynthetic pathways, involved in the formation of FPP, are differentially associated with peroxisomes and may easily diffuse to the cytosol. Based on these observations, the significance and a possible regulatory model in the formation of isoprenoid end-products are discussed.

627 ||| 7742357 ||| (4, 0.72) ||| 4 ||| 5 ||| Metabolism of short-chain ceramide and dihydroceramide analogues in Chinese hamster ovary (CHO) cells. A series of radiolabelled ceramides (D-erythro and L-threo) and dihydroceramides (DL-erythro and DL-threo) with 2, 4 or 6 carbon N-acyl groups were synthesized. These analogues were incubated with cultured CHO cells and radioactive products isolated and analyzed. In addition to synthesis of short-chain sphingomyelin and glucosylceramide, radiolabelled sphingosine and sphinganine were released from short-chain ceramides and dihydroceramides and subsequently utilized for synthesis of long-chain ceramide and sphingolipids. Substrate preference for short-chain sphingomyelin synthesis in cells was D-erythro-ceramides > L-threo-ceramides > DL-erythro-dihydroceramides > DL-threo-dihydroceramides, and C4- and C6-analogues were preferred over the C2-analogue. Kinetic constants for conversion of short-chain (dihydro)ceramides to short-chain sphingomyelin were determined using CHO cell membranes and found to correlate with substrate preference in cultured cells. D-erythro-C6-Ceramide was the preferred substrate for short-chain glucosylceramide synthesis. D-erythro-C2-ceramide inhibited incorporation of [3H]serine into sphingomyelin, glucosylceramide and ceramide rapidly (2 h) and in a dose-dependent manner. Over a similar time period, [3H]choline-labelling of sphingomyelin was not affected. Inhibition of [3H]serine-labelling of sphingolipids appeared to correlate with release of [3H]long-chain bases from short-chain ceramides and dihydroceramides and synthesis of long-chain sphingolipids. However, some discrepancies between DL-erythro-C4- and C6-dihydroceramides, and D-erythro-C2-ceramide suggested that short-chain dihydroceramides were less efficient in suppressing de novo synthesis from [3H]serine, while contributing substantially to endogenous sphingolipid synthesis. Inhibition of de novo sphingolipid synthesis by short-chain ceramides and dihydroceramides could not be related to inhibition of serine palmitoyltransferase activity in vitro.

628 ||| 11027164 ||| (4, 0.72) ||| 4 ||| 5 ||| Effect of PDI overexpression on recombinant protein secretion in CHO cells. In eukaryotic cells, protein disulfide isomerase (PDI) found in the endoplasmic reticulum (ER) catalyzes disulfide bond exchange and assists in protein folding of newly synthesized proteins. PDI also functions as a molecular chaperone and has been found associated with proteins in the ER. In addition, PDI functions as a subunit of two more complex enzyme systems: the prolyl-4-hydroxylase and the triacylglycerol transfer proteins. Increasing PDI activity in bacterial, yeast, and insect cell expression systems can lead to increased secretion of heterologous proteins containing disulfide bridges. Since Chinese hamster ovary (CHO) cells are widely used for the expression of recombinant proteins, we expressed recombinant human PDI (rhu PDI) in CHO cells to increase cellular PDI levels and examined its effect on the secretion of two different recombinant proteins: interleukin 15 (IL-15) and a tumor necrosis factor receptor:Fc fusion protein (TNFR:Fc). Secretion of TNFR:Fc (a disulfide-rich protein) is decreased in cells overexpressing PDI; the TNFR:Fc protein is retained inside these cells and colocalizes with the overexpressed rhu PDI protein in the endoplasmic reticulum. PDI overexpression did not result in intracellular retention of IL15. The nature of the interaction between PDI and TNFR:Fc was further investigated by expressing a disulfide isomerase mutant PDI in CHO cells to determine if the functional activity of PDI is involved in the cellular retention of TNFR:Fc protein.

629 ||| 9354375 ||| (4, 0.70) ||| 4 ||| 5 ||| Characterization of tyrosine-phosphorylated delta isoform of protein kinase C isolated from Chinese hamster ovary cells. Phorbol ester treatment of Chinese hamster ovary cells stably overexpressing the delta isoform of protein kinase C induced the association of the isoform with the particulate fraction and the tyrosine phosphorylation of a small portion of the delta isoform. The delta isoform without tyrosine phosphorylation was recovered as an enzyme dependent on phospholipid and diacylglycerol, whereas the tyrosine-phosphorylated delta isoform was recovered in two fractions, one dependent on, and the other independent of, phospholipid and diacylglycerol. The tyrosine-phosphorylated delta isoform independent of lipid activators might be associated with phorbol ester and phospholipids. Immunoblot analysis revealed that the delta isoform is a doublet protein of 76 and 78 kDa, and that the delta isoform fraction without tyrosine phosphorylation contained 76- and 78-kDa proteins, whereas the tyrosine-phosphorylated delta isoform contained the 78-kDa protein but not the 76-kDa protein. In vitro analysis showed that the 78-kDa protein of the delta isoform without tyrosine phosphorylation is an efficient substrate of tyrosine kinase only when phosphatidylserine and either diacylglycerol or phorbol ester are present; however, the 76-kDa protein can not be tyrosine-phosphorylated even in the presence of these lipid activators. The phospholipid and diacylglycerol-dependent form of the tyrosine-phosphorylated enzyme isolated from the cell line required lower concentrations of phosphatidylserine and phorbol ester for its activity in vitro as compared with the enzyme without tyrosine phosphorylation. These results suggest that the tyrosine-phosphorylated enzyme generated upon stimulation of the cells may associate with membranes and exert its full activity even with the lower concentrations of the lipid activators.

630 ||| 9114014 ||| (4, 0.70) ||| 4 ||| 5 ||| A fibroblast cell line defective in alkyl-dihydroxyacetone phosphate synthase: a novel defect in plasmalogen biosynthesis. Using fluorescence-activated cytotoxicity selection, followed by colony autoradiographic screening of the surviving population, we have isolated a unique plasmalogen-deficient Chinese hamster ovary (CHO) cell line. The mutant, NZel-1, showed a dramatic (90%) reduction in the rate of biosynthesis and levels of plasmalogens, as determined using short- and long-term labeling with 32Pi. Enzymatic assays and lipid supplementation studies showed that NZel-1 was defective in a single step in the biosynthetic pathway for plasmalogens. This step, catalyzed by the peroxisomal enzyme, alkyl-dihydroxyacetone phosphate (DHAP) synthase, is responsible for the introduction of the ether bond found in plasmalogens. The activity of alkyl-DHAP synthase was reduced in whole-cell homogenates from NZel-1 to 18% of wild-type values. Unlike previously described plasmalogen-deficient mutants, NZel-1 contained peroxisomes, as confirmed by immunofluorescence microscopy and catalase release by digitonin. Peroxisomal functions, including the breakdown of very long-chain (>20 carbons) fatty acids, phytanic acid oxidation, and the acylation of DHAP, were normal. Cell fusion studies revealed that the mutation is recessive and belongs to a new complementation group. To our knowledge this is the first report describing the isolation and characterization of a mutant CHO cell line defective in plasmalogen biosynthesis which contains intact, functional peroxisomes. These cells will allow us to examine the role of ether lipids in cellular functions without complications associated with peroxisome deficiency.

631 ||| 8910328 ||| (4, 0.70) ||| 4 ||| 5 ||| A point mutation causes mistargeting of Golgi GlcNAc-TV in the Lec4A Chinese hamster ovary glycosylation mutant. The Lec4A and Lec4 Chinese hamster ovary glycosylation mutants lack N-linked glycans with GlcNAcbeta(1,6)Manalpha(1,6) branches that are initiated by the transferase termed GlcNAc-TV. Detergent extracts of Lec4 cells have no detectable GlcNAc-TV activity, but Lec4A extracts have activity equivalent to that of parental Chinese hamster ovary cells. This discrepancy occurs because Lec4A GlcNAc-TV activity co-localizes with membranes of the endoplasmic reticulum (ER) instead of with Golgi membranes (Chaney, W., Sundaram, S., Friedman, N., and Stanley, P. (1989) J. Cell. Biol. 109, 2089-2096). cDNAs from the coding region of the GlcNAc-TV gene have now been isolated from each mutant line. Lec4 GlcNAc-TV cDNA was found to possess two insertions, the first of which shifts the open reading frame and codes for a truncated transferase missing 585 amino acids from the catalytic domain. By contrast, Lec4A GlcNAc-TV cDNA possesses a single point mutation from T to G, which results in a change from Leu to Arg at position 188. When transfected into Lec4 cells, both cDNAs gave the appropriate phenotype; Lec4 cDNA was unable to restore GlcNAc-TV activity, whereas Lec4A cDNA converted Lec4 cells to the Lec4A phenotype, with an active GlcNAc-TV mislocalized to ER membranes. Moreover, Lec4A cDNA cured of its mutation restored a functional, Golgi-localized GlcNAc-TV to Lec4 cells. The results demonstrate that a single change in the 740 amino acids of GlcNAc-TV serves to functionally inactivate the transferase in an intact cell by causing it to localize to the ER instead of the Golgi compartment. The mislocalized transferase retains full enzyme activity, showing that it is well folded and stable and suggesting that the L188R mutation either prevents association with exit complexes from the ER or causes retrograde transport from a Golgi compartment.

632 ||| 7640304 ||| (4, 0.70) ||| 4 ||| 5 ||| An aphidicolin-resistant mutant of Chinese hamster ovary cell with altered DNA polymerase and 3' exonuclease activities. A comparison was made of partially purified DNA polymerases alpha, delta, epsilon and from normal Chinese hamster ovary cells and mutant cells (JK3-1-2A) resistant to aphidicolin, araA, and araC. In vitro the pol alpha from the mutant cells (1) was resistant to aphidicolin and araCTP but was sensitive to araATP, (2) showed a 1.6 to 2.6-fold reduced specific activity, and (3) was more sensitive to carbonyldiphosphonate, DMSO and SJK 287-38 anti-pol alpha antibody inhibition, but was less sensitive to alkylphenyl nucleotide analogs BuPdGTP and BuAdATP. On the other hand, pol delta and pol epsilon of the mutant cells did not show increased aphidicolin-resistance but differed from the wild type enzymes with regard to their 3' exonuclease activity. The 3' exonuclease/DNA polymerase activity ratio was increased 6-fold for pol delta and 3.3-fold for pol epsilon for enzymes from the mutant cells in comparison to wild type values. It is suggested that these altered properties of the DNA polymerases of the alpha-family are responsible for in vivo aphidicolin resistance of the mutant cells. The higher 3' exonuclease activity may explain the observed antimutator phenotype of this cell line. In view of the proficient 3' exonuclease activities of the DNA pol delta and epsilon, the present aphr mutant is unique among all mammalian DNA polymerase mutants.

633 ||| 9148949 ||| (4, 0.69) ||| 4 ||| 5 ||| Cloning, sequencing, and enzymatic activity of an inducible aldo-keto reductase from Chinese hamster ovary cells. Treatment of Chinese hamster ovary (CHO) cells by the aldehyde containing calpain inhibitor I resulted in the induction of a 35-kDa protein that was partially sequenced and shown to be a member of the aldo-keto reductase superfamily (Inoue, S., Sharma, R. C., Schimke, R. T., and Simoni, R. D. (1993) J. Biol. Chem. 268, 5894-5898). Using rapid amplification of cDNA ends polymerase chain reaction, we have sequenced the cDNA for this protein (CHO reductase). This enzyme is a new member of the aldo-keto reductase superfamily and shows greatest amino acid sequence identity to mouse fibroblast growth factor-regulated protein and mouse vas deferens protein (92 and 80% sequence identity, respectively). The enzyme exhibits about 70% sequence identity with the aldose reductases (ALR2; EC 1.1.1.21) and about 47% with the aldehyde reductases (ALR1; EC 1.1.1.2). Northern analysis showed that it is induced in preference to either ALR1 or ALR2 and RNase protection assays showed gene expression in bladder, testis, jejunum, and ovary in descending order of expression. The cDNA for this inducible reductase was cloned into the pET16b vector and expressed in BL21(DE3) cells. Expressed CHO reductase showed kinetic properties distinct from either ALR1 or ALR2 including the ability to metabolize ketones. This protein joins a growing number of inducible aldo-keto reductases that may play a role in cellular regulation and protection.

634 ||| 7479912 ||| (4, 0.68) ||| 4 ||| 5 ||| Muscarinic acetylcholine receptor down-regulation limits the extent of inhibition of cell cycle progression in Chinese hamster ovary cells. Cellular desensitization is believed to be important for growth control but direct evidence is lacking. In the current study we compared effects of wild-type and down-regulation-resistant mutant m3 muscarinic receptors on Chinese hamster ovary (CHO-K1) cell desensitization, proliferation, and transformation. We found that down-regulation of m3 muscarinic acetylcholine receptors was the principal mechanism of desensitization of receptor-activated inositol phosphate phospholipid hydrolysis in these cells. Activation of wild-type and mutant receptors inhibited anchorage-independent growth as assayed by colony formation in agar. However, the potency for inhibition of anchorage-independent growth was greater for cells expressing the mutant receptor. Activation of either receptor also initially inhibited anchorage-dependent cell proliferation in randomly growing populations. Rates of DNA synthesis and cell division were profoundly reduced by carbachol in cells expressing either receptor at early time points. Analysis of cell cycle parameters indicated that cell cycle progression was inhibited at transitions from G1 to S and G2/M to G1 phases. However, mutant receptor effects on anchorage-dependent growth were sustained, whereas wild-type receptor effects were transient. Thus, receptor down-regulation restored cell cycle progression. In contrast, activation of either receptor blocked entry into the cell cycle from quiescence, and this response was not reduced by receptor down-regulation. Therefore, activation of m3 muscarinic acetylcholine receptors inhibited CHO cell anchorage-dependent and -independent growth. In anchored cells carbachol inhibited the cell cycle at three distinct points. Inhibitions at two of these points were eliminated by wild-type receptor down-regulation while the other was not. These results directly demonstrate that desensitization mechanisms can act as principal determinants of cellular growth responses.

635 ||| 22863936 ||| (4, 0.68) ||| 4 ||| 5 ||| The essential structures of ISP-I that influence serine palmitoyltransferase inhibition in Chinese hamster ovary cells. We investigated the structure-activity relationship between various ISP-I (myriocin, thermozymocidin) analogous which has sphingosine-like structure and serine palmitoyltransferase (SPT) in Chinese hamster ovary (CHO) cells utilizing sphingolipid production as a marker. Our data suggest that the double bond and/or ketone group within the alkyl chain as well as the alkyl chain are necessary for ISP-I to inhibit SPT. In addition, a serine structure is necessary for SPT inhibitory activity, which confirms previous findings.

636 ||| 10446148 ||| (4, 0.68) ||| 4 ||| 5 ||| Control of phosphatidylserine synthase II activity in Chinese hamster ovary cells. Phosphatidylserine (PtdSer) in Chinese hamster ovary (CHO) cells is synthesized through the action of PtdSer synthase (PSS) I and II, which catalyzes the exchange of L-serine with the base moiety of phosphatidylcholine and phosphatidylethanolamine, respectively. The PtdSer synthesis in a CHO cell mutant, PSA-3, which lacks PSS I but has normal PSS II activity, was almost completely inhibited by the addition of PtdSer to the culture medium, like that in the wild-type CHO-K1 cells. In contrast, the PtdSer synthesis in a PSS II-overproducing stable transformant of CHO-K1, K1/wt-pssB, was reduced by only 35% upon addition of PtdSer. The serine exchange activity in a membrane fraction of K1/wt-pssB cells was not inhibited by PtdSer at all, whereas those of PSA-3 and CHO-K1 cells were inhibited by >95%. These results indicated that PSS II activity in PSA-3 and CHO-K1 cells is inhibited by exogenous PtdSer and that overproduction of PSS II leads to the loss of normal control of PSS II activity by exogenous PtdSer. Although overproduced PSS II in K1/wt-pssB cells was not normally controlled by exogenous PtdSer, K1/wt-pssB cells cultivated without exogenous PtdSer exhibited a normal PtdSer biosynthetic rate similar to that in CHO-K1 cells. In contrast to K1/wt-pssB cells, another stable transformant of CHO-K1, K1/R97K-pssB, which overproduces R97K mutant PSS II, exhibited a approximately 4-fold higher PtdSer biosynthetic rate compared with that in CHO-K1 cells. These results suggested that for maintenance of a normal PtdSer biosynthetic rate, the activity of overproduced wild-type PSS II in K1/wt-pssB cells is depressed by an as yet unknown post-translational mechanisms other than those for the exogenous PtdSer-mediated inhibition and that Arg-97 of PSS II is critical for this depression of overproduced PSS II activity. When the cDNA-directed wild-type and R97K mutant PSS II activities were expressed at nonoverproduction levels in a PSS I- and PSS II-defective mutant of CHO-K1 cells, expression of the mutant PSS II activity but not that of the wild-type PSS II activity induced the PtdSer-resistant PtdSer biosynthesis. This suggested that Arg-97 of PSS II is critical also for the exogenous PtdSer-mediated inhibition of PSS II.

637 ||| 9724629 ||| (4, 0.67) ||| 4 ||| 5 ||| DPM2 regulates biosynthesis of dolichol phosphate-mannose in mammalian cells: correct subcellular localization and stabilization of DPM1, and binding of dolichol phosphate. Biosynthesis of glycosylphosphatidylinositol and N-glycan precursor is dependent upon a mannosyl donor, dolichol phosphate-mannose (DPM). The Thy-1negative class E mutant of mouse lymphoma and Lec15 mutant Chinese hamster ovary (CHO) cells are incapable of DPM synthesis. The class E mutant is defective in the DPM1 gene which encodes a mammalian homologue of Saccharomyces cerevisiae Dpm1p that is a DPM synthase, whereas Lec15 is a different mutant, indicating that mammalian DPM1 is not sufficient for DPM synthesis. Here we report expression cloning of a new gene, DPM2, which is defective in Lec15 cells. DPM2, an 84 amino acid membrane protein expressed in the endoplasmic reticulum (ER), makes a complex with DPM1 that is essential for the ER localization and stable expression of DPM1. Moreover, DPM2 enhances binding of dolichol phosphate, a substrate of DPM synthase. Mammalian DPM1 is catalytic because a fusion protein of DPM1 that was stably expressed in the ER synthesized DPM without DPM2. Therefore, biosynthesis of DPM in mammalian cells is regulated by DPM2.

638 ||| 8917671 ||| (4, 0.67) ||| 4 ||| 5 ||| Arsenite efflux is inhibited by verapamil, cyclosporin A, and GSH-depleting agents in arsenite-resistant Chinese hamster ovary cells. We have previously demonstrated that arsenite-resistant SA7 cells can extrude arsenite more effectively and completely than their parental Chinese hamster ovary cells. Our present results show that arsenite efflux from SA7 cells is inhibited by chemosensitizing agents to multidrug resistant-associated protein: verapamil and cyclosporin A and by glutathione-depleting agents: dinitrofluorobenzene and diethyl maleate. These results suggest that arsenite extrusion in SA7 cells may be mediated by a GSH-dependent and verapamil- and cyclosporin A-sensitive membrane transport system. Since arsenite extrusion was found dose-dependently inhibited by energy poison [potassium cyanide (KCN)] and an ATPase inhibitor (sodium vanadate), ATP is apparently required for arsenite extrusion in SA7 cells.

639 ||| 18571506 ||| (4, 0.67) ||| 4 ||| 5 ||| Isolation and characterization of mutant animal cell line defective in alkyl-dihydroxyacetonephosphate synthase: localization and transport of plasmalogens to post-Golgi compartments. We herein isolated plasmalogen-deficient Chinese hamster ovary (CHO) mutant, ZPEG251, with a phenotype of normal import of peroxisomal matrix and membrane proteins. In ZPEG251, plasmenylethanolamine (PlsEtn) was severely reduced. Complementation analysis by expression of genes responsible for the plasmalogen biogenesis suggested that alkyl-dihydroxyacetonephosphate synthase (ADAPS), catalyzing the second step of plasmalogen biogenesis, was deficient in ZPEG251. ADAPS mRNA was barely detectable as verified by Northern blot and reverse transcription-PCR analyses. Defect of ADAPS expression was also assessed by immunoblot. As a step toward delineating functional roles of PlsEtn, we investigated its subcellular localization. PlsEtn was localized to post-Golgi compartments and enriched in detergent-resistant membranes. Transport of PlsEtn to post-Golgi compartments was apparently affected by lowering cellular ATP, but not by inhibitors of microtubule assembly and vesicular transport. Partitioning of cholesterol and sphingomyelin, a typical feature of lipid rafts, was not impaired in plasmalogen-deficient cells, including peroxisome assembly-defective mutants, hence suggesting that PlsEtn was not essential for lipid-raft architecture in CHO cells.

640 ||| 16316991 ||| (4, 0.67) ||| 4 ||| 5 ||| Mechanism of proteasomal degradation of inositol trisphosphate receptors in CHO-K1 cells. myo-Inositol 1,4,5-trisphosphate receptor (IP3R) degradation occurs in response to carbachol (Cch) stimulation of CHO-K1 cells. The response was mediated by endogenous muscarinic receptors and was blocked by atropine or proteasomal inhibitors. We have used these cells to identify the sites of ubiquitination on IP3Rs and study the role of Ca2+ and substrate recognition properties of the degradation system using exogenously expressed IP3R constructs. Employing caspase-3 for IP3R cleavage, we show that Cch promotes polyubiquitination in the N-terminal domain and monoubiquitination in the C-terminal domain. The addition of extracellular Ca2+ to Ca2+-depleted Chinese hamster ovary (CHO) cells initiates IP3R degradation provided Cch is present. This effect is inhibited by thapsigargin. The data suggest that both a sustained elevation of IP3 and a minimal content of Ca2+ in the endoplasmic reticulum lumen is required to initiate IP3R degradation. Transient transfection of IP3R constructs into CHO cells indicated the selective degradation of only the SI+ splice variant of the type I IP3R. This was also the splice form present endogenously in these cells. A pore-defective, nonfunctional SI+ IP3R mutant (D2550A) was also degraded in Cch-stimulated cells. The Cch-mediated response in CHO cells provides a convenient model system to further analyze the Ca2+ dependence and structural requirements of the IP3R proteasomal degradation pathway.

641 ||| 10864928 ||| (4, 0.67) ||| 4 ||| 5 ||| Location of the glucuronosyltransferase domain in the heparan sulfate copolymerase EXT1 by analysis of Chinese hamster ovary cell mutants. Heparan sulfate formation occurs by the copolymerization of glucuronic acid (GlcA) and N-acetylglucosamine (GlcNAc) residues. Recent studies have shown that these reactions are catalyzed by a copolymerase encoded by EXT1 and EXT2, members of the exostosin family of putative tumor suppressors linked to hereditary multiple exostoses. Previously, we identified a collection of Chinese hamster ovary cell mutants (pgsD) that failed to make heparan sulfate (Lidholt, K., Weinke, J. L., Kiser, C. S., Lugemwa, F. N., Bame, K. J., Cheifetz, S., Massague, J., Lindahl, U., and Esko, J. D. (1992) Proc. Natl. Acad. Sci. U. S. A. 89, 2267-2271). Here, we show that pgsD mutants contain mutations that either alter GlcA transferase activity selectively or that affect both GlcNAc and GlcA transferase activities. Expression of EXT1 corrects the deficiencies in the mutants, whereas EXT2 and the related EXT-like cDNAs do not. Analysis of the EXT1 mutant alleles revealed clustered missense mutations in a domain that included a (D/E)X(D/E) motif thought to bind the nucleotide sugar from studies of other transferases. These findings provide insight into the location of the GlcA transferase subdomain of the enzyme and indicate that loss of the GlcA transferase domain may be sufficient to cause hereditary multiple exostoses.

642 ||| 9448939 ||| (4, 0.66) ||| 4 ||| 5 ||| Simulations of the effects of inositol 1,4,5-trisphosphate 3-kinase and 5-phosphatase activities on Ca2+ oscillations. Inositol 1,4,5-trisphosphate (Ins-1,4,5-P3) is responsible for Ca2+ mobilization in response to external stimulation in many cell types. The latter phenomenon often occurs as repetitive Ca2+ spikes. In this study, the effect of the two Ins-1,4,5-P3 metabolizing enzymes (Ins-1,4,5-P3 3-kinase and 5-phosphatase) on the temporal pattern of Ca2+ oscillations has been investigated. On the basis of the well-documented Ins-1,4,5-P3 3-kinase stimulation by the Ca2+/calmodulin complex and of the experimentally-determined kinetic characteristics of these enzymes, we predict that 5-phosphatase primarily controls the levels of Ins-1,4,5-P3 and, thereby, the occurrence and frequency of Ca2+ oscillations. Consequently, the model reproduces the experimental observation performed in Chinese hamster ovary cells that 5-phosphatase overexpression has a much more pronounced effect on the pattern of Ca2+ oscillations than 3-kinase overexpression. We also investigated, in more detail, under which conditions a similar effect could be observed in other cell types expressing various Ins-1,4,5-P3 3-kinase activities.

643 ||| 9405438 ||| (4, 0.66) ||| 4 ||| 5 ||| Protection against oxidative stress-induced cell death by intracellular platelet-activating factor-acetylhydrolase II. Platelet-activating factor-acetylhydrolase (PAF-AH), which removes the acetyl group at the sn-2 position of PAF, is distributed widely in tissues and plasma. Tissue cytosol contains at least two types of PAF-AH, isoforms Ib and II. Isoform Ib is a tertiary G-protein complex-like heterotrimeric enzyme that is involved in brain development such as formation of the brain cortex. Isoform II (PAF-AH(II)), however, is a 40-kDa monomer and has an amino acid sequence that exhibits a 41% identity with that of plasma PAF-AH. Although PAF-AH(II) preferentialy hydrolyzes oxidized phospholipids as well as PAF in vitro, the function of this enzyme has not, as yet, been elucidated. Here, we report that PAF-AH(II) functions as an anti-oxidant phospholipase. PAF-AH(II) was found to be an N-myristoylated enzyme that has never been reported among lipases and phospholipases. In MDBK cells treated with oxidants, PAF-AH(II) translocated from cytosol to membranes within 20 min, whereas in cells treated with anti-oxidants, it translocated, conversely, from membranes to cytosol. Overexpression of PAF-AH(II) in Chinese hamster ovary-K1 cells suppressed oxidative stress-induced cell death, which occurs by apoptosis. These findings suggest that intracellular PAF-AH(II) translocates between cytosol and membranes in response to a redox state of the cell and protects the cell against oxidative stress most probably by hydrolyzing oxidized phospholipids.

644 ||| 9079687 ||| (4, 0.65) ||| 4 ||| 5 ||| A novel cytosolic calcium-independent phospholipase A2 contains eight ankyrin motifs. We report the purification, molecular cloning, and expression of a novel cytosolic calcium-independent phospholipase A2 (iPLA2) from Chinese hamster ovary cells, which lacks extended homology to other phospholipases. iPLA2 is an 85-kDa protein that exists as a multimeric complex of 270-350 kDa with a specific activity of 1 micromol/min/mg. The full-length cDNA clone encodes a 752-amino acid cytoplasmic protein with one lipase motif (GXS465XG) and eight ankyrin repeats. Expression of the cDNA in mammalian cells generates an active 85-kDa protein. Mutagenesis studies show that Ser465 and the ankyrin repeats are required for activity. We demonstrate that iPLA2 selectively hydrolyzes the sn-2 over sn-1 fatty acid by 5-fold for 1,2-dipalmitoyl phosphatidylcholine in a mixed micelle. Moreover, we found the fatty acid preference at the sn-2 position to be highly dependent upon substrate presentation. However, iPLA2 does have a marked preference for 1,2-dipalmitoyl phosphatidic acid presented in a vesicle, generating the lipid second messenger lysophosphatidic acid. Finally the enzyme is able to hydrolyze the acetyl moiety at the sn-2 position of platelet-activating factor.

645 ||| 8982265 ||| (4, 0.65) ||| 4 ||| 5 ||| Effect of fumonisin B1 on phosphatidylethanolamine biosynthesis in Chinese hamster ovary cells. Fumonisin B1 has been shown to inhibit dihydroceramide synthesis and elevate cellular sphinganine levels in several cultured cell lines. In Chinese hamster ovary (CHO)-K1 cells, 20 microM fumonisin B1 inhibited sphingomyelin synthesis by 75% after 5 h, but stimulated [3H]serine incorporation into PtdEtn by 5- to 7-fold. Fumonisin caused a 10-20% increase in [3H]serine labelling of PtdSer. While fumonisin (20 microM) caused sustained inhibition of sphingomyelin synthesis, PtdEtn labelling peaked at 7-fold above controls at 12 h and declined to 4-fold by 24 h. Fumonisin treatment for 12 h increased the in vitro activity of PtdSer synthase by 62% and inhibited PtdSer decarboxylase by 35%, suggesting that increased PtdEtn labelling by [3H]serine is not by this pathway. An ethanolamine 'trap' experiment was performed to assess the contribution of phosphoethanolamine from sphinganine degradation for PtdEtn labelling. Stimulation of [3H]serine incorporation into PtdEtn by fumonisin could be reduced by 60% with the inclusion of 50 microM unlabelled ethanolamine in the culture medium. The ethanolamine-mediated reduction in [3H]serine incorporation into PtdEtn was accompanied by 4-fold increase in cellular [3H]phosphoethanolamine. In control cells labelled with [3H]serine, 50 microM ethanolamine did not cause [3H]phosphoethanolamine to accumulate. Consistent with elevated phosphoethanolamine production in fumonisin-treated cells, [3H]ethanolamine incorporation into PtdEtn was inhibited by 75% after 12 h. The degradation of endogenous long-chain bases to phosphoethanolamine and entry into the CDP-ethanolamine pathway appears to be a major pathway for PtdEtn synthesis in fumonisin-treated CHO-K1 cells.

646 ||| 8576232 ||| (4, 0.65) ||| 4 ||| 5 ||| Progesterone inhibits cholesterol biosynthesis in cultured cells. Accumulation of cholesterol precursors. Cells acquire cholesterol through endogenous synthesis and through receptor-mediated uptake of cholesterol-rich low density lipoprotein (LDL). Esterification of LDL-derived cholesterol is catalyzed by acyl-CoA:cholesterol acyltransferase (ACAT) in the endoplasmic reticulum (ER). Progesterone inhibits esterification, and, although the mechanism of inhibition is not completely understood, this inhibition results from progesterone's ability to inhibit the activity of multiple drug resistance (MDR) P-glycoproteins (P. DeBry and J. E. Metherall, submitted for publication). In the current manuscript, we demonstrate that progesterone inhibits cholesterol biosynthesis resulting in the accumulation of a number of sterol precursors. In Chinese hamster ovary (CHO) cells, high concentrations (100 microM) of progesterone completely blocked cholesterol production, resulting in the accumulation of lanosterol and a lanosterol precursor. Lower concentrations (40 microM) of progesterone cause plasma membrane accumulation of several sterol products. The majority of these sterols are precursors of cholesterol since they were efficiently converted to cholesterol upon removal of progesterone from the culture medium. Although very high concentrations (> 200 microM) of progesterone killed CHO cells, their growth was restored by the addition of cholesterol to the growth medium, indicating that progesterone toxicity resulted from cholesterol auxotrophy. The effect of progesterone was not unique to CHO cells; progesterone also inhibited cholesterol biosynthesis in all human cell lines tested. These observations suggest that a common progesterone-sensitive pathway is involved in both cholesterol biosynthesis and the processing of LDL-derived cholesterol.

647 ||| 7575679 ||| (4, 0.65) ||| 4 ||| 5 ||| A QSAR study comparing the cytotoxicity and DNA topoisomerase II inhibitory effects of bisdioxopiperazine analogs of ICRF-187 (dexrazoxane). A series of twelve structurally related bisdioxopiperazines that included ICRF-187 (dexrazoxane), ICRF-159 (razoxane), ICRF-193, and ICRF-154 were examined both for their ability to inhibit the growth of Chinese hamster ovary (CHO) cells and their ability to inhibit the catalytic activity of mammalian DNA topoisomerase II. The bisdioxopiperazines exhibited a wide range in both growth inhibitory effects (30,000-fold), and in their ability to inhibit the catalytic activity of topoisomerase II (150-fold). The cytotoxicity of the bisdioxopiperazines toward CHO cells was highly correlated (correlation coefficient r = 0.86, P = 0.0003) with their inhibition of the catalytic activity of DNA topoisomerase II. This result strongly suggests that DNA topoisomerase II is the functional target of the bisdioxopiperazines. The stereoisomers (+)-ICRF-187 and (-)-ICRF-186 were observed to be equally cytotoxic and equally inhibitory toward DNA topoisomerase II. This result indicates that the bisdioxopiperazine binding site on DNA topoisomerase II is large enough or flexible enough to accommodate either form of the drug. The strongly metal-ion binding fully rings-opened hydrolysis product of ICRF-187, ADR-925, demonstrated no measurable inhibitory activity toward DNA topoisomerase II or cytotoxicity toward CHO cells.

648 ||| 7491919 ||| (4, 0.64) ||| 4 ||| 5 ||| Volume-associated osmolyte fluxes in cell lines with or without the anion exchanger. To investigate the involvement of a red cell-type anion exchanger in the volume-sensitive amino acid release, the hyposmolarity-evoked release of D-[3H]aspartate and [3H]taurine was examined in three cell lines: 1) wild-type Chinese hamster ovary (CHO-K1) cells, expressing an anion exchanger activity (Cl-/SO4(2-)) functionally similar to the erythroid band 3; 2) a mutant CHO cell type (CHO 605) lacking this anion exchanger activity; and 3) 293 cells in which the Cl-/HCO3(-) anion exchanger is absent. All cell types accumulated D-[3H]aspartate and [3H]taurine under isosmotic conditions, and, similarly, in the three cell lines, cell swelling evoked by hyposmolarity induced a rapid and transient increase in the amino acid efflux. Blockers of the anion exchanger and/or Cl- channels [niflumic acid, dipyridamole, diphenylamine-2-carboxylate,5-nitro-2-(3-phenylpropylamino)-benzoi c acid, and 4,4'-diisothiocyanostilbene-2,2'-disulfonic acid] were potent inhibitors of amino acid efflux in the three cell lines. 125I- efflux, used as a marker for Cl- fluxes, was also markedly increased in response to cell swelling in all cell lines, and this efflux was inhibited by the anion exchanger/Cl- channel blockers. These results do not support a role for an anion exchanger in the hyposmolarity-induced amino acid efflux and suggest that amino acids and Cl- may be transported by the same or a similar mechanism, presumably an anion channel-like structure.

649 ||| 21876188 ||| (4, 0.64) ||| 4 ||| 5 ||| Identification of a de novo thymidylate biosynthesis pathway in mammalian mitochondria. The de novo and salvage dTTP pathways are essential for maintaining cellular dTTP pools to ensure the faithful replication of both mitochondrial and nuclear DNA. Disregulation of dTTP pools results in mitochondrial dysfunction and nuclear genome instability due to an increase in uracil misincorporation. In this study, we identified a de novo dTMP synthesis pathway in mammalian mitochondria. Mitochondria purified from wild-type Chinese hamster ovary (CHO) cells and HepG2 cells converted dUMP to dTMP in the presence of NADPH and serine, through the activities of mitochondrial serine hydroxymethyltransferase (SHMT2), thymidylate synthase (TYMS), and a novel human mitochondrial dihydrofolate reductase (DHFR) previously thought to be a pseudogene known as dihydrofolate reductase-like protein 1 (DHFRL1). Human DHFRL1, SHMT2, and TYMS were localized to mitochondrial matrix and inner membrane, confirming the presence of this pathway in mitochondria. Knockdown of DHFRL1 using siRNA eliminated DHFR activity in mitochondria. DHFRL1 expression in CHO glyC, a previously uncharacterized mutant glycine auxotrophic cell line, rescued the glycine auxotrophy. De novo thymidylate synthesis activity was diminished in mitochondria isolated from glyA CHO cells that lack SHMT2 activity, as well as mitochondria isolated from wild-type CHO cells treated with methotrexate, a DHFR inhibitor. De novo thymidylate synthesis in mitochondria prevents uracil accumulation in mitochondrial DNA (mtDNA), as uracil levels in mtDNA isolated from glyA CHO cells was 40% higher than observed in mtDNA isolated from wild-type CHO cells. These data indicate that unlike other nucleotides, de novo dTMP synthesis occurs within mitochondria and is essential for mtDNA integrity.

650 ||| 9306012 ||| (4, 0.63) ||| 4 ||| 5 ||| The role of exocytosis in the activation of the chloride conductance in Chinese hamster ovary cells (CHO) stably expressing CFTR. The aim of this study was to examine the question of whether activation of wt-CFTR (wild-type cystic fibrosis transmembrane conductance regulator) by cAMP and the opening of a Cl- conductance is paralleled by exocytosis and corresponding increases in membrane capacitance. To this end three types of Chinese hamster ovary (CHO) cells were examined: a control group of CHO cells; a group of CHO cells stably expressing wt-CFTR at high levels (also called BQ2-CHO); and a group of CHO cells stably expressing the frequent mutation DeltaF508-CFTR. Whole-cell patch-clamp studies were performed to measure the membrane voltage (Vm), the membrane conductance (Gm) and the membrane capacitance (Cm). Cm was assessed by a two-frequency lock-in amplifier method. Forskolin (Fsk, 0.1 micromol/l) and isobutylmethylxanthine (IBMX, 0.1 mmol/l) were used to increase cytosolic cAMP. It is shown that Fsk and IBMX had no effect on Vm and Gm in control CHO and DeltaF508-CFTR-CHO cells. Fsk and IBMX depolarized wt-CFTR-expressing CHO cells significantly (from -40 +/- 1.5 to -32 +/- 1.6 mV, n = 41) and enhanced Gm strongly from 5.0 +/- 0.9 to 36 +/- 3.9 nS (n = 65). The conductance increase was mostly for Cl-, because under stimulated conditions a reduction in bath Cl- concentration depolarized these cells further and significantly from -26 +/- 1.8 to -10 +/- 1.2 mV (n = 16). This conductance had the characteristic wt-CFTR selectivity of Br- > Cl- > I- (n = 16). Despite this large increase in the Fsk- and IBMX-induced conductance Cm was not altered significantly (15.5 versus 15.7 pF, n = 50). These data indicate that stable overexpression of wt-CFTR but not of DeltaF508-CFTR in CHO cells induces a cAMP-activated Cl- conductance. The activation of this large conductance obviously proceeds with little if any exocytosis.

651 ||| 9235902 ||| (4, 0.63) ||| 4 ||| 5 ||| Cloning of a Chinese hamster ovary (CHO) cDNA encoding phosphatidylserine synthase (PSS) II, overexpression of which suppresses the phosphatidylserine biosynthetic defect of a PSS I-lacking mutant of CHO-K1 cells. Phosphatidylserine (PtdSer) in mammalian cells is synthesized through the exchange of free L-serine for the polar head group (base) of preexisting phospholipid. We previously showed the presence of two different enzymes catalyzing the serine base exchange in Chinese hamster ovary (CHO) cells and isolated the cDNA of one of the enzymes, PtdSer synthase (PSS) I, which also catalyzes the exchange of the base moiety of phospholipid(s) for ethanolamine and choline. In this study, we cloned a CHO cDNA, designated as pssB, which encodes a protein exhibiting 32% amino acid sequence identity with CHO PSS I. Introduction of the pssB cDNA into CHO-K1 cells resulted in striking increases in both the serine and ethanolamine base exchange activities. In contrast to the PSS I cDNA, the pssB cDNA was incapable of increasing the choline base exchange activity. The expression of the pssB gene in Sf9 insect cells also results in striking increases in both serine and ethanolamine base exchange activities. The pssB cDNA was found to transform a PtdSer-auxotrophic PSS I-lacking mutant of CHO-K1 cells to PtdSer prototrophy. The PtdSer content of the resultant transformant grown without exogenous PtdSer for 2 days was 4-fold that of the mutant and similar to that of CHO-K1 cells, indicating that the pssB cDNA complemented the PtdSer biosynthetic defect of the PSS I-lacking mutant. These results suggested that the pssB cDNA encoded the second PtdSer synthase PSS II, which catalyzed the serine and ethanolamine base exchange, but not the choline base exchange.

652 ||| 7592658 ||| (4, 0.63) ||| 4 ||| 5 ||| Insulin activates nuclear factor kappa B in mammalian cells through a Raf-1-mediated pathway. We examined the effect of insulin on nuclear factor kappa B (NF-kappa B) activity in Chinese ovary (CHO) cells overexpressing wild-type (CHO-R cells) or -defective insulin receptors mutated at Tyr1162 and Tyr1163 autophosphorylation sites (CHO-Y2 cells). In CHO-R cells, insulin caused a specific, time-, and concentration-dependent activation of NF-kappa B. The insulin-induced DNA-binding complex was identified as the p50/p65 heterodimer. Insulin activation of NF-kappa B: 1) was related to insulin receptor number and tyrosine kinase activity since it was markedly reduced in parental CHO cells which proved to respond to insulin growth factor-1 and phorbol 12-myristate 13-acetate (PMA) activation, and was dramatically decreased in CHO-Y2 cells; 2) persisted in the presence of cycloheximide and was blocked by pyrrolidine dithiocarbamate, aspirin and sodium salicylate, three compounds interfering with I kappa B degradation and/or NF-kappa B.I kappa B complex dissociation; 3) was independent of both PMA-sensitive and atypical (zeta) protein kinases C; and 4) was dependent on Raf-1 kinase activity since insulin-stimulated NF-kappa B DNA binding activity was inhibited by 8-bromo-cAMP, a Raf-1 kinase inhibitor. Moreover, insulin activation of NF-kappa B-driven luciferase reporter gene expression was blocked in CHO-R cells expressing a Raf-1 dominant negative mutant. This is the first evidence that insulin activates NF-kappa B in mammalian cells through a post-translational mechanism requiring both insulin receptor tyrosine kinase and Raf-1 kinase activities.

653 ||| 12139966 ||| (4, 0.63) ||| 4 ||| 5 ||| Molecular cloning of Chinese hamster ceramide glucosyltransferase and its enhanced expression in peroxisome-defective mutant Z65 cells. To clarify the metabolic bases of characteristic increases in the concentrations of glucosylceramide (CMH) and GM3 in peroxisome-defective mutant Chinese hamster ovary (CHO) cells (Z65), we measured the ceramide glucosyltransferase (CGT) and beta-glucosidase activities in Z65 and CHO-K1 cells, and found that the former enzyme was responsible for the accumulation of CMH in Z65 cells. Inhibition of CGT by D,L-threo-1-phenyl-2-decanoylamino-3-morpholino-1-propanol (PDMP) caused a marked reduction in a incorporation of [3-14C]serine to CMH in both CHO-K1 and Z65 cells, but resulted in the accumulation of ceramide in Z65 cells in a concentration higher than that in CHO-K1 cells. Then, we cloned the cDNA encoding CGT from CHO-K1 cells, which exhibited sequence homology with the human gene product (98.7%). Northern blot analysis of CGT revealed increased expression of it in Z65 cells compared with that in CHO-K1 cells, which probably caused the simultaneous increase in GM3. With an immunohistochemical procedure, GM3 was found to be more strongly expressed in the cell membrane of Z65 cells than in CHO-K1 cells.

654 ||| 10216280 ||| (4, 0.63) ||| 4 ||| 5 ||| Accumulation of glycolipids in mutant Chinese hamster ovary cells (Z65) with defective peroxisomal assembly and comparison of the metabolic rate of glycosphingolipids between Z65 cells and wild-type CHO-K1 cells. The influence of peroxisomal dysfunction on glycosphingolipid metabolism was investigated using mutant Chinese hamster ovary (CHO) cells (Z65) with defective assembly of the peroxisomal membranes. In accordance with previous observations, the concentration of very long chain fatty acid (C24:0) was shown to be higher in Z65 cells than in control cells. We then compared the composition of glycolipids in Z65 cells with that in CHO-K1 cells, which are wild-type Chinese hamster ovary cells with intact peroxisomes, and found significantly increased concentrations of ceramide monohexoside (CMH) and ganglioside GM3 in Z65 cells. However, there were no differences in the concentrations of glycerophospholipids, triglycerides, free fatty acids and cholesterol between Z65 and CHO-K1 cells. Further, to investigate the metabolic rate of the major lipids, Z65 and CHO-K1 cells were pulse-labeled with [3-14C]serine. [3-14C]Serine was incorporated into phosphatidylserine, phosphatidylethanolamine and sphingomyelin more quickly in CHO-K1 than in Z65 cells. However, after 48 h, the radioactivity incorporated into those lipids, including CMH, was greater in Z65 cells than in CHO-K1 cells. Thus, the altered metabolism of glycosphingolipids, probably due to peroxisomal dysfunction, was thought to be responsible for the change in glycosphingolipid composition in Z65 cells.

655 ||| 7646554 ||| (4, 0.62) ||| 4 ||| 5 ||| Degradation of 2-(3-aminopropylamino)-ethanethiol (WR-1065) by Cu-dependent amine oxidases and influence on glutathione status of Chinese hamster ovary cells. The radioprotective drug 2-(3-aminopropylamino)ethanethiol (WR-1065) can be degraded when incubated in cell culture medium in vitro. The degradation reaction consumes oxygen and results from the action of Cu-dependent amine oxidases present in the serum content of the medium. Analysis of the degradation products of WR-1065 demonstrates the formation of cysteamine, acrolein and H2O2. WR-2721, the inactive prodrug of WR-1065, is not a substrate for these enzymes. Extracellular degradation of WR-1065 by Cu-dependent amine oxidases leads to an intracellular depletion of glutathione (GSH) in Chinese hamster ovary (CHO) cells and to reduction of clonogenic cell survival. Addition of aminoguanidine, an inhibitor of Cu-dependent amine oxidases, protects CHO cells from the toxic effects of WR-1065 and under these conditions an increase of intracellular GSH levels occurs. These data demonstrate that WR-1065 can be degraded to toxic compounds by the presence of Cu-dependent amine oxidases which might have further implications for the clinical use of WR-2721.

656 ||| 7544576 ||| (4, 0.62) ||| 4 ||| 5 ||| Structural and functional analysis of the canine histamine H2 receptor by site-directed mutagenesis: N-glycosylation is not vital for its action. G-protein-coupled receptors generally share a similar structure containing seven membrane-spanning domains and extracellular site(s) for N-glycosylation. The histamine H2 receptor is a member of the family of G-protein-coupled receptors, and has three extracellular potential sites for N-glycosylation (Asn-4, Asn-162 and Asn-168). To date, however, no information has been presented regarding N-glycosylation of the H2 receptor. To investigate the presence, location and functional roles of N-glycosylation of the H2 receptor, site-directed mutagenesis was performed to eliminate the potential site(s) for N-glycosylation singly and collectively. The wild-type and mutated H2 receptors were expressed stably in Chinese hamster ovary (CHO) cells or transiently in COS7 cells. Immunoblotting of the wild-type and mutated H2 receptors with an antiserum directed against the C-terminus of the H2 receptor showed that mutation at Asn-162, but not at Asn-168, resulted in a substantial decrease in the molecular mass. A mutation at Asn-4 led to a further decrease in the molecular mass. Tunicamycin treatment of the transfected cells yielded a sharp band with a molecular mass identical to that of the mutant devoid of all three potential sites for N-glycosylation. These findings indicate that the H2 receptor is N-glycosylated, and that N-glycosylation takes place mainly at two sites, Asn-4 and Asn-162. Neither the affinity for tiotidine nor that for histamine was affected by the mutagenesis. Immunocytochemistry and tiotidine binding showed that the mutated receptors were exclusively distributed on the cell surface in a fashion similar to that of the wild-type. In addition, the glycosylation-defective receptor was capable of activating adenylate cyclase and elevating the intracellular Ca2+ concentration in response to histamine in stable CHO cell lines. Thus N-glycosylation of the H2 receptor is not required for cell surface localization, ligand binding or functional coupling to G-protein(s).

657 ||| 10400226 ||| (4, 0.62) ||| 4 ||| 5 ||| Supersensitization of the adenylyl cyclase system in Chinese hamster ovary cells co-expressing cloned opioid receptors and Gz, a PTX-insensitive G protein. Chronic and/or sustained stimulation of opioid receptors has been shown to lead to an increase in activity of the adenylyl cyclase (AC) system via pertussis toxin (PTX)-sensitive Gi/o, family G protein. In the present study, we examined whether supersensitization of the AC system is induced via a PTX-insensitive G protein, Gz, activation of which leads to the inhibition of AC activity. In Chinese hamster ovary (CHO) cells expressing either mu- or kappa-opioid receptors, acute treatment with morphine or U69,593, but not naloxone or norbinaltorphimine, respectively, suppressed forskolin-induced cyclic AMP (cAMP) accumulation, while sustained (4 h) treatment with the opioid agonists induced cAMP overshoot above the naive level (supersensitization of the AC system). Both effects were completely blocked by pretreatment with PTX. In CHO cells co-expressing mu- or kappa-opioid receptors and alpha(z), inhibitory effects of cAMP accumulation by acute treatment with the opioid agonists and supersensitization of the AC system by sustained treatment with them were induced despite pretreatment with PTX. These data suggest that supersensitization of the AC system is induced by sustained opioid agonist treatment not only via Gi/o but also via Gz.

658 ||| 9539713 ||| (4, 0.61) ||| 4 ||| 5 ||| Control of phosphatidylserine biosynthesis through phosphatidylserine-mediated inhibition of phosphatidylserine synthase I in Chinese hamster ovary cells. Phosphatidylserine (PtdSer) synthesis in Chinese hamster ovary (CHO) cells occurs through the exchange of L-serine with the base moiety of phosphatidylcholine or phosphatidylethanolamine. The synthesis is depressed on the addition of PtdSer to the culture medium. A CHO cell mutant named mutant 29, whose PtdSer biosynthesis is highly resistant to this depression by exogenous PtdSer, has been isolated from CHO-K1 cells. In the present study, the PtdSer-resistant PtdSer biosynthesis in the mutant was traced to a point mutation in the PtdSer synthase I gene, pssA, resulting in the replacement of Arg-95 of the synthase by lysine. Introduction of the mutant pssA cDNA, but not the wild-type pssA cDNA, into CHO-K1 cells induced the PtdSer-resistant PtdSer biosynthesis. In a cell-free system, the serine base-exchange activity of the wild-type pssA-transfected cells was inhibited by PtdSer, but that of the mutant pssA-transfected cells was resistant to the inhibition. Like the mutant 29 cells, the mutant pssA-transfected cells grown without exogenous PtdSer exhibited an approximately 2-fold increase in the cellular PtdSer level compared with that in CHO-K1 cells, although the wild-type pssA-transfected cells did not exhibit such a significant increase. These results indicated that the inhibition of PtdSer synthase I by PtdSer is essential for the maintenance of a normal PtdSer level in CHO-K1 cells and that Arg-95 of the synthase is a crucial residue for the inhibition.

659 ||| 17586788 ||| (4, 0.61) ||| 4 ||| 5 ||| Amplification of the gene for SCAP, coupled with Insig-1 deficiency, confers sterol resistance in mutant Chinese hamster ovary cells. The endoplasmic reticulum membrane proteins Insig-1 and Insig-2 limit cholesterol synthesis, in part through their sterol-dependent binding to sterol-regulatory element binding protein (SREBP) cleavage-activating protein (SCAP). This binding prevents proteolytic processing of SREBPs, membrane-bound transcription factors that enhance cholesterol synthesis. We report here the characterization of mutant Chinese hamster ovary (CHO) cells, designated SRD-19, that are resistant to 25-hydroxycholesterol, a potent inhibitor of SREBP processing. SRD-19 cells were produced by mutagenesis of Insig-1-deficient SRD-14 cells, followed by selection in high levels of 25-hydroxycholesterol. 25-Hydroxycholesterol fails to suppress SREBP processing in SRD-19, even though they express normal levels of Insig-2. The number of copies of the gene encoding SCAP was found to be increased by 4-fold in SRD-19 cells compared with wild-type CHO cells, leading to the overproduction of SCAP mRNA and protein. Our data indicate that overproduced SCAP saturates the remaining Insig-2 in SRD-19 cells, thus explaining their resistance to 25-hydroxycholesterol. Consistent with this conclusion, regulated SREBP processing is restored in SRD-19 cells upon transfection of plasmids encoding either Insig-1 or Insig-2. These results highlight the importance of SCAP/Insig ratios in normal sterol-regulated processing of SREBPs in cultured cells.

660 ||| 8626666 ||| (4, 0.60) ||| 4 ||| 5 ||| A single point mutation in epsilon-COP results in temperature-sensitive, lethal defects in membrane transport in a Chinese hamster ovary cell mutant. At the nonpermissive temperature of 39.5 degrees C, the Chinese hamster ovary cell conditionally lethal, temperature-sensitive (ts) mutant ldlF exhibits the following defects: rapid degradation of low density lipoprotein receptors, disruption of ER-through Golgi transport, and disintegration of the Golgi apparatus. All of these are corrected by transfection with an expression vector for wild-type epsilon-COP, a subunit of coatomers (Guo, Q., Vasile, E., and Krieger, M. (1994) J. Cell Biol. 125, 1213-1224). We now report the identification in ldlF cells of a point mutation in the epsilon-COP gene, Glu251 to Lys251, which prevents the corresponding cDNA from correcting the defects in transfected ldlF cells and the immunochemical analysis of the synthesis, structure, and stability of epsilon-COP. At the permissive temperature (34 degrees C), the steady state level of ts-epsilon-COP in ldlF cells was about half that of epsilon-COP in wild-type Chinese hamster ovary cells and the isoelectric point of ts-epsilon-COP was 0.14 pH units higher than that of the wild-type protein. The stability but not the biosynthesis of ts-epsilon-COP was temperature-sensitive (t1/2 > 6 h at 34 degrees C and approximately 1-2 h at 39.5 degrees C), and this accounts for the virtual absence of detectable ts-epsilon-COP protein in ldlF cells after incubation at 39.5 degrees C for > 6h. The steady state levels in ldlF cells of another coatomer subunit, beta-COP, and the peripheral Golgi protein ldlCp were not temperature-sensitive. Thus, a mutation in epsilon-COP that causes instability at 39.5 degrees C is responsible for all of the temperature-sensitive defects in ldlF cells, and the stability of beta-COP is not linked directly to that of epsilon-COP. ldlF cells should be useful for the future analysis of the structure and function of epsilon-COP, the assembly of COPs into coatomers, and the participation of coatomers in intracellular membrane transport.

661 ||| 17673461 ||| (4, 0.60) ||| 4 ||| 5 ||| The CDP-ethanolamine pathway and phosphatidylserine decarboxylation generate different phosphatidylethanolamine molecular species. In mammalian cells, phosphatidylethanolamine (PtdEtn) is mainly synthesized via the CDP-ethanolamine (Kennedy) pathway and by decarboxylation of phosphatidylserine (PtdSer). However, the extent to which these two pathways contribute to overall PtdEtn synthesis both quantitatively and qualitatively is still not clear. To assess their contributions, PtdEtn species synthesized by the two routes were labeled with pathway-specific stable isotope precursors, d(3)-serine and d(4)-ethanolamine, and analyzed by high performance liquid chromatography-mass spectrometry. The major conclusions from this study are that (i) in both McA-RH7777 and Chinese hamster ovary K1 cells, the CDP-ethanolamine pathway was favored over PtdSer decarboxylation, and (ii) both pathways for PtdEtn synthesis are able to produce all diacyl-PtdEtn species, but most of these species were preferentially made by one pathway. For example, the CDP-ethanolamine pathway preferentially synthesized phospholipids with mono- or di-unsaturated fatty acids on the sn-2 position (e.g. (16:0-18:2)PtdEtn and (18:1-18:2)PtdEtn), whereas PtdSer decarboxylation generated species with mainly polyunsaturated fatty acids on the sn-2 position (e.g. (18:0-20:4)PtdEtn and (18:0-20:5)PtdEtn in McArdle and (18: 0-20:4)PtdEtn and (18:0-22:6)PtdEtn in Chinese hamster ovary K1 cells). (iii) The main PtdEtn species newly synthesized from the Kennedy pathway in the microsomal fraction appeared to equilibrate rapidly between the endoplasmic reticulum and mitochondria. (iv) Newly synthesized PtdEtn species preferably formed in the mitochondria, which is at least in part due to the substrate specificity of the phosphatidylserine decarboxylase, seemed to be retained in this organelle. Our data suggest a potentially essential role of the PtdSer decarboxylation pathway in mitochondrial functioning.

662 ||| 10092635 ||| (4, 0.60) ||| 4 ||| 5 ||| Chinese hamster ovary cells require the coexpression of microsomal triglyceride transfer protein and cholesterol 7alpha-hydroxylase for the assembly and secretion of apolipoprotein B-containing lipoproteins. Due to the absence of microsomal triglyceride transfer protein (MTP), Chinese hamster ovary (CHO) cells lack the ability to translocate apoB into the lumen of the endoplasmic reticulum, causing apoB to be rapidly degraded by an N-acetyl-leucyl-leucyl-norleucinal-inhibitable process. The goal of this study was to examine if expression of MTP, whose genetic deletion is responsible for the human recessive disorder abetalipoproteinemia, would recapitulate the lipoprotein assembly pathway in CHO cells. Unexpectedly, expression of MTP mRNA and protein in CHO cells did not allow apoB-containing lipoproteins to be assembled and secreted by CHO cells expressing apoB53. Although expression of MTP in cells allowed apoB to completely enter the endoplasmic reticulum, it was degraded by a proteolytic process that was inhibited by dithiothreitol (1 mM) and chloroquine (100 microM), but resistant to N-acetyl-leucyl-leucyl-norleucinal. In marked contrast, coexpression of the liver-specific gene product cholesterol 7alpha-hydroxylase with MTP resulted in levels of MTP lipid transfer activity that were similar to those in mouse liver and allowed intact apoB53 to be secreted as a lipoprotein particle. These data suggest that, although MTP-facilitated lipid transport is not required for apoB translocation, it is required for the secretion of apoB-containing lipoproteins. We propose that, in CHO cells, MTP plays two roles in the assembly and secretion of apoB-containing lipoproteins: 1) it acts as a chaperone that facilitates apoB53 translocation, and 2) its lipid transfer activity allows apoB-containing lipoproteins to be assembled and secreted. Our results suggest that the phenotype of the cell (e.g. expression of cholesterol 7alpha-hydroxylase by the liver) may profoundly influence the metabolic relationships determining how apoB is processed into lipoproteins and/or degraded.

663 ||| 9799804 ||| (4, 0.59) ||| 4 ||| 5 ||| Splice donor site mutations in the 3-hydroxy-3-methylglutaryl coenzyme A reductase gene cause a deficiency of the endoplasmic reticulum 3-hydroxy-3-methylglutaryl coenzyme A reductase protein in UT2 cells. UT2 cells are a mutant clone of Chinese hamster ovary (CHO) cells that are deficient in the 97 kDa endoplasmic reticulum (ER) 3-hydroxy-3-methylglutaryl coenzyme A (HMG-CoA) reductase protein. The analysis of UT2 cell cDNA and genomic DNA has led to the identification of two novel point mutations in intronic sequences of the ER HMG-CoA reductase gene. One mutation identified at the +1 position (G --> A) of the 5' splice site of exon 11-12 junction was shown to cause exon 11 skipping which resulted in the insertion of premature stop codons. We also identified a second mutation at the +5 position (G --> A) of the 5' splice site in the intron spanning exons 13 and 14. Furthermore, the data indicate that the two mutations in the reductase gene are present on the same allele. As demonstrated by reverse transcription-polymerase chain reaction (RT-PCR) of UT2 cell mRNA, the mutations produce aberrant spliced messages. If the aberrant messages were translated, truncated proteins of 44 kDa or 66 kDa would be predicted. More importantly, these truncated proteins would be expected not to have catalytic activity. In addition, we have also recently demonstrated that the UT2 cells express a 90 kDa HMG-CoA reductase protein that is localized exclusively in peroxisomes, and is up-regulated when the cells are grown in the absence of added mevalonate. Thus, the mutations identified in the ER reductase gene in UT2 cells indicate that neither a 97 kDa nor a 90 kDa reductase protein can be produced from this gene.

664 ||| 7592670 ||| (4, 0.59) ||| 4 ||| 5 ||| Heparan sulfate proteoglycans are primarily responsible for the maintenance of enzyme activity, binding, and degradation of lipoprotein lipase in Chinese hamster ovary cells. Various aspects of lipoprotein lipase (LPL) metabolism, including cell surface binding, degradation, and enzymatic activity, were compared between Chinese hamster ovary (CHO) cells and two distinct proteoglycan-deficient CHO cell lines. The contribution of low density lipoprotein receptor-related protein in binding LPL was also analyzed by the use of a 39-kDa receptor-associated protein expressed as a glutathione S-transferase fusion protein (GST-RAP). Equilibrium binding data with 125I-LPL revealed the presence of a class of high affinity binding sites with a KD of 7.8 nM in CHO cells, whereas no high affinity binding was observed for proteoglycan-deficient cells. The high affinity binding of LPL in CHO cells appeared to be concentrated in cell surface projections and was not effectively inhibited by GST-RAP. Moreover, degradation of endogenous and exogenous LPL was significantly greater in control CHO cells than in proteoglycan-deficient cells. Degradation of LPL in CHO cells was not affected by GST-RAP, suggesting that proteoglycans and not low density lipoprotein receptor-related protein are responsible for the majority of binding and degradation of LPL in these cells. Our data also show that proteoglycan binding is not essential for the assembly of active LPL homodimers, although proteoglycan binding controls the distribution of LPL activity. Furthermore, LPL produced by CHO cells was more stable than LPL produced by proteoglycan-deficient cells.

665 ||| 18848543 ||| (4, 0.59) ||| 4 ||| 5 ||| The peroxin Pex14p is involved in LC3-dependent degradation of mammalian peroxisomes. As a step toward understanding the homeostasis of peroxisomes in mammalian cells, we investigated a degradation system of peroxisomes in Chinese hamster ovary (CHO)-K1 cells in response to the nutrient-starvation. Peroxisomal proteins were degraded apparently in a preferential manner as compared to cytosolic proteins, when CHO-K1 cells were starved in Hank's solution and then re-cultured in a normal medium. We verified whether microtubule-associated protein I light chain 3 (LC3), an essential factor for autophagy, was involved in the degradation of peroxisomal proteins. In the LC3-knocked-down CHO-K1 cells, the specific degradation of peroxisomal proteins was no longer observed and proteins including peroxisomal and cytosolic proteins were rather non-selectively degraded under the starvation condition. The starvation-dependent non-selective protein degradation was inhibited with proteasome inhibitors, MG132 and Epoxomicin. The integral membrane peroxin, Pex14p interacted with membrane-bound LC3-II, the modified form of LC3, via microtubules under the starvation condition. Taken together, these results suggest that peroxisomal proteins are degraded by two degradation systems involving autophagy and proteasomes depending on various cell-culture conditions, and that Pex14p plays a pivotal role as a prerequisite factor for the degradation of peroxisomal proteins by autophagy with the aid of microtubules.

666 ||| 17408498 ||| (4, 0.59) ||| 4 ||| 5 ||| Synthesis of the oxysterol, 24(S), 25-epoxycholesterol, parallels cholesterol production and may protect against cellular accumulation of newly-synthesized cholesterol. AIM: The effects of 24(S),25-epoxycholesterol (24,25EC) on aspects of cholesterol homeostasis is well-documented. When added to cells, 24,25EC decreases cholesterol synthesis and up-regulates cholesterol efflux genes, including ABCA1. Synthesis of 24,25EC occurs in a shunt of the mevalonate pathway which also produces cholesterol. Therefore, 24,25EC synthesis should be subject to the same negative feedback regulation as cholesterol synthesis. To date, no role has been ascribed to 24,25EC in light of the fact that increased accumulation of cholesterol should decrease formation of this oxysterol through feedback inhibition. This leads to the intriguing paradox: why inhibit production of an apparently important regulator of cholesterol homeostasis when it is needed most? METHODS: We used a combination of pharmacological and genetic approaches in Chinese Hamster Ovary cell-lines to investigate this paradox. Endogenous synthesis of 24,25EC was manipulated using partial inhibition of the enzyme, Oxidosqualene Cyclase. Changes in cholesterol and 24,25EC synthesis were determined using metabolic labelling with [1-14C]-acetate, thin-layer chromatography and phosphorimaging. Transcriptional effects mediated via SREBP and LXR were analysed by luciferase reporter assays. RESULTS: We showed that cholesterol addition to cells lead to a rapid and preferential inhibition of 24,25EC synthesis. Addition of 24,25EC resulted in parallel inhibition of 24,25EC and cholesterol synthesis. Furthermore, we used a variety of approaches to examine the relationship between cholesterol and 24,25EC synthesis, including cell-lines with different rates of cholesterol synthesis, varying cholesterol synthetic rates by pre-treatment with a statin, or lipoprotein cholesterol loading of macrophages. In all cases, we showed that 24,25EC synthesis faithfully tracked cholesterol synthesis. Moreover, changes in 24,25EC synthesis exerted downstream effects, reducing SREBP transcriptional activity whilst increasing ABCA1 and LXR transcriptional activity. CONCLUSION: Our results show that 24,25EC synthesis parallels cholesterol synthesis, consistent with this oxysterol functioning as a safety valve to protect against the accumulation of newly-synthesised cholesterol (as opposed to exogenously-derived cholesterol). Considering that 24,25EC is capable of being produced in all cholesterogenic cells, we propose that production of 24,25EC may represent a ubiquitous defence mechanism.

667 ||| 16901265 ||| (4, 0.59) ||| 4 ||| 5 ||| SREBP-2 positively regulates transcription of the cholesterol efflux gene, ABCA1, by generating oxysterol ligands for LXR. Cholesterol accumulation and removal are regulated by two different transcription factors. SREBP-2 (sterol-regulatory-element-binding protein-2) is best known to up-regulate genes involved in cholesterol biosynthesis and uptake, whereas LXR (liver X receptor) is best known for up-regulating cholesterol efflux genes. An important cholesterol efflux gene that is regulated by LXR is the ATP-binding cassette transporter, ABCA1 (ATP-binding cassette transporter-A1). We have previously shown that statin treatment down-regulated ABCA1 expression in human macrophages, probably by inhibiting synthesis of the LXR ligand 24(S),25-epoxycholesterol. However, it was subsequently reported that ABCA1 expression is down-regulated by SREBP-2 through binding of SREBP-2 to an E-box element in ABCA1's proximal promoter. As statin treatment induces SREBP-2 activation, this may provide an alternative explanation for the statin-mediated down-regulation of ABCA1. In the present study, we employed a set of CHO (Chinese-hamster ovary) mutant cell lines to investigate the role of SREBP-2 in the regulation of ABCA1. We observed increased ABCA1 mRNA levels in SREBP-2-overexpressing cells and decreased levels in cells lacking a functional SREBP-2 pathway, which were restored when the SREBP-2 pathway was reinstated. Moreover, ABCA1 gene expression was positively associated with synthesis of 24(S),25-epoxycholesterol in these cell lines. In studies using a human ABCA1 promoter reporter assay, mutation of the E-box motif had a similar response as the wild-type construct to either statin treatment or addition of 24(S),25-epoxycholesterol. By contrast, these responses were completely ablated when the DR4 element to which LXR binds was mutated. These results support the idea that 24(S),25-epoxycholesterol and statin treatment influence ABCA1 transcription via supply of an LXR ligand and not through an SREBP-2/E-box-related mechanism. In addition, our results indicate a critical role of SREBP-2 as a positive regulator of ABCA1 gene expression by enabling the generation of oxysterol ligands for LXR.

668 ||| 10921918 ||| (4, 0.59) ||| 4 ||| 5 ||| Molecular defects that cause loss of polysialic acid in the complementation group 2A10. Polysialic acid (PSA) is a dynamically regulated posttranslational modification of the neural cell adhesion molecule (NCAM), which modulates NCAM binding functions. PSA biosynthesis is catalyzed by two polysialyltransferases, ST8SiaII and ST8SiaIV. The catalytic mechanisms of these enzymes are unknown. In Chinese hamster ovary cells, ST8SiaIV is responsible for PSA expression. In the complementation group 2A10, the ST8SiaIV gene is disrupted. Investigating the molecular defects in this complementation group, seven clones with missense mutations in ST8SiaIV were found. Mutations cause replacement of amino acids that are highly conserved in alpha2,8-sialyltransferases. To verify the physiological relevance of identified mutations, identical amino acid substitutions were introduced into epitope-tagged variants of hamster ST8SiaIV and murine ST8SiaII and recombinant proteins were tested in vivo and in vitro. None of these constructs reconstituted PSA synthesis in 2A10 cells, although the proteins were expressed and with the exception of the cysteine variants ST8SiaIV-C356F and ST8SiaII-C371F correctly targeted to the Golgi apparatus. Interestingly, two mutations (ST8SiaIV-R277G and -M333V and the corresponding mutants ST8SiaII-R292G and -M348V) could be partially rescued if tested in vitro. Although these mutants were negative for autopolysialylation, partial reconstitution of both auto- and NCAM polysialylation was achieved in the presence of NCAM. The data presented in this study suggest a functional link between auto- and NCAM polysialylation.

669 ||| 9919308 ||| (4, 0.58) ||| 4 ||| 5 ||| Structural and functional analysis of the protein products derived from mutant fur alleles in an endoprotease-deficient Chinese hamster ovary cell strain. The fur gene encodes the endoprotease, furin. We recently demonstrated mutations in both fur alleles in the mutant Chinese hamster ovary (CHO)-K1 strain, RPE.40, and hypothesized that these mutations were responsible for the endoprotease-deficient phenotype of these cells. We now present the structural and functional properties of three protein products derived from the mutant fur alleles. None of these protein products were able to process the precursor to von Willebrand factor, which is processed by wild-type furin. Pro-protein processing activity initially attributed to one of the mutant proteins was due to wild-type furin produced inadvertently from one of the expression constructs used in these experiments. None of the mutant proteins exhibited evidence of autocatalysis, consistent with the lack of activity versus the test substrate, and glycosylation patterns suggested at least two of them remained in the endoplasmic reticulum. These results confirm that RPE.40 cells are furin null mutants, as earlier evidence had suggested.

670 ||| 9452450 ||| (4, 0.58) ||| 4 ||| 5 ||| Preferential decarboxylation of hydrophilic phosphatidylserine species in cultured cells. Implications on the mechanism of transport to mitochondria and cellular aminophospholipid species compositions. In baby hamster kidney and other cultured cells the majority of phosphatidylethanolamine (PE) is synthesized from phosphatidylserine (PS) in a process which involves transport of PS from the endoplasmic reticulum to mitochondria and decarboxylation therein by PS decarboxylase. To study the mechanism of this transport process, we first determined the molecular species composition of PE and PS from baby hamster kidney and Chinese hamster ovary cells. Interestingly, the hydrophilic diacyl molecular species were found to be much more abundant in PE than in PS, suggesting that hydrophilic PS species may be more readily transported to mitochondria than the hydrophobic ones. To study this, we compared the rates of decarboxylation of different PS molecular species in these cells. The cells were pulse labeled with [3H]serine whereafter the distribution of the labels among PS and PE molecular species was determined by reverse phase high performance liquid chromatography and liquid scintillation counting. The hydrophilic PE species contained relatively much more 3H label than those of PS, which indicates that they are more readily decarboxylated than the hydrophobic ones. Control experiments showed that differences in [3H]PS and -PE molecular species profiles are not due to (i) incorporation of 3H label to some PE species via alternative pathways, (ii) differences in degradation or remodeling among species, or (iii) selective decarboxylation of PS molecular species by the enzyme. Therefore, hydrophilic PS species are indeed decarboxylated faster than the hydrophobic ones. The rate of decarboxylation decreased systematically with hydrophobicity, strongly suggesting that formation of so called activated monomers, i.e. lipid molecules perpendicularly displaced from the membrane (Jones, J. D., and Thompson, T. E. (1990) Biochemistry 29, 1593-1600), is the rate-limiting step in the transport of PS from the endoplasmic reticulum to mitochondria. The formation of activated monomers and thus the rate of transfer is probably greatly enhanced by frequent collisions between the two membranes which tend to be closely associated. The present data also provides a feasible explanation why hydrophilic molecular species in these cells are much more abundant in PE as compared with PS, its immediate precursor.

671 ||| 8977298 ||| (4, 0.57) ||| 4 ||| 5 ||| Defective antigen processing correlates with a low level of intracellular glutathione. Previously, we reported that Chinese hamster ovary (CHO) cells transfected with murine mouse major histocompatibility complex class II genes, exhibit a unique antigen (Ag) processing defect whereby these cells are impaired in processing only Ag with disulfide bonds. Here, we examined various aspects of the intracellular reducing environment in the CHO cells to understand the underlying mechanism causing the defect. A cell hybrid generated by the fusion of CHO cells and L cell fibroblasts was used for comparison due to their competency in processing Ag. The transport pathway of cysteine within the CHO cells appeared normal. However, these cells had a significantly lower level of glutathione, a major physiological reducing thiol, compared to the cell hybrid. Treatment of the CHO cells with N-acetyl-L-cysteine did not augment their glutathione content nor their ability to process Ag. When the cell hybrid was treated with L-buthionine-(S,R)-sulfoximine (BSO), which significantly decreased their glutathione level, the hybrid poorly processed hen egg lysozyme (HEL) and ovalbumin, which have disulfide bonds. In contrast, BSO treatment did not affect the capacity of the hybrid to process pigeon cytochrome c and carboxymethylated HEL, which lack disulfide bonds. Therefore, low intracellular glutathione levels in antigen-presenting cells correlated with defective processing of Ag with disulfide bonds, indicating that this thiol may be a critical factor in regulating productive Ag processing.

672 ||| 8825742 ||| (4, 0.57) ||| 4 ||| 5 ||| Cytotoxicity studies of quercetin, shikimate, cyclohexanecarboxylate and ptaquiloside. A study was carried out to determine the cytotoxic effects of quercetin, shikimate, cyclohexane-carboxylate and ptaquiloside on Chinese hamster ovary (CHO), mouse fibroblast (3T3) and normal rat kidney (NRK) cells by cell growth inhibition assay of monolayers grown in 96-well plates. Molar concentrations inhibiting cell growth by 50% (IC50) after 48 h of incubation for quercetin, shikimate, cyclohexanecarboxylate and ptaquiloside were 0.8 x 10(-4)M, 0.8 x 10(-3)M, 0.4 x 10(-2) M and 0.6 x 10(-1)M respectively for CHO cells; 1.2 x 10(-4)M, 0.7 x 10(-3)M, 0.2 x 10(-7)M and 0.6 x 10(-1)M respectively for 3T3 cells; and 0.7 x 10(-4)M, 1.0 x 10(-3)M, 0.2 x 10(-2)M and 0.6 x 10(-1)M respectively for NRK cells. These inhibiting concentrations were extremely high when compared with mitomycin C whose cytotoxic effect occurred at 1 x 10(-6)M. Cyclohexanecarboxylate is a metabolite of shikimate; any possible cytotoxic effect of shikimate in vivo may be mediated by the major metabolite cyclohexanecarboxylate per se or after futher metabolism of this metabolite following absorption in animals. Quercetin, ptaquiloside and shikimate are constituents of bracken fern. The low order of cytotoxic activity of these constituents suggest that they are probably not directly the etiological agents of acute cattle bracken fern poisoning.

673 ||| 9884408 ||| (4, 0.56) ||| 4 ||| 5 ||| Nonglucosylated oligosaccharides are transferred to protein in MI8-5 Chinese hamster ovary cells. A CHO mutant MI8-5 was found to synthesize Man9-GlcNAc2-P-P-dolichol rather than Glc3Man9GlcNAc2-P-P-dolichol as the oligosaccharide-lipid intermediate in N-glycosylation of proteins. MI8-5 cells were incubated with labeled mevalonate, and the prenol was found to be dolichol. The mannose-labeled oligosaccharide released from oligosaccharide-lipid of MI8-5 cells was analyzed by HPLC and alpha-mannosidase treatment, and the data were consistent with a structure of Man9GlcNAc2. In addition, MI8-5 cells did not incorporate radioactivity into oligosaccharide-lipid during an incubation with tritiated galactose, again consistent with MI8-5 cells synthesizing an unglucosylated oligosaccharide-lipid. MI8-5 cells had parental levels of glucosylphosphoryldolichol synthase activity. However, in two different assays, MI8-5 cells lacked dolichol-P-Glc:Man9GlcNAc2-P-P-dolichol glucosyltransferase activity. MI8-5 cells were found to synthesize glucosylated oligosaccharide after they were transfected with Saccharomyces cerevisiae ALG 6, the gene for dolichol-P-Glc:Man9GlcNAc2-P-P-dolichol glucosyltransferase. MI8-5 cells were found to incorporate mannose into protein 2-fold slower than parental cells and to approximately a 2-fold lesser extent.

674 ||| 9843380 ||| (4, 0.56) ||| 4 ||| 5 ||| Antiapoptotic signaling by the insulin receptor in Chinese hamster ovary cells. We have sought to determine whether insulin can promote cell survival and protect Chinese hamster ovary (CHO) cells from apoptosis induced by serum starvation. Low concentrations of insulin were antiapoptotic for cells overexpressing wild-type insulin receptors but not in cells transfected with kinase-defective insulin receptor mutants that lacked a functional ATP binding site. However, treatment with orthovanadate (50 microM), a widely used tyrosine phosphatase inhibitor, led a dramatic reduction in internucleosomal DNA fragmentation in both cell lines. Cells transfected with truncated receptor mutants in either the juxtamembrane or C-terminal domain were as responsive as cells overexpressing wild-type receptors in mediating insulin antiapoptotic protection. The mechanisms underlying insulin antiapoptotic protection were investigated using a variety of pharmacological tools known to inhibit distinct signaling pathways. The phosphatidylinositol-3' kinase inhibitors wortmannin and LY294002 had only a modest influence whereas blocking protein farnesylation with manumycin severely disrupted the antiapoptotic capacity of the insulin receptor. Of interest, cells gained antiapoptotic potential following inhibition of extracellular signal-regulated kinase activation with the pharmacological agent PD98059. Insulin induced MKK3/MKK6 phosphorylation and activation of p38 MAP kinase whose activity was inhibited with SB203580. However, the inhibition of p38 MAP kinase had no effect on the protection offered by insulin. We conclude that the antiapoptotic function of the insulin receptor requires intact receptor kinase activity and implicates a farnesylation-dependent pathway. Increase in cellular phosphotyrosine content, however, triggers antiapoptotic signal that may converge downstream of the insulin receptor.

675 ||| 10801878 ||| (4, 0.56) ||| 4 ||| 5 ||| Why expression of phosphatidylethanolamine N-methyltransferase does not rescue Chinese hamster ovary cells that have an impaired CDP-choline pathway. The mutant Chinese hamster ovary cell line (CHO), MT58, has a temperature-sensitive mutation in CTP:phosphocholine cytidylyltransferase (CT), preventing phosphatidylcholine (PC) synthesis at 40 degrees C which results in apoptosis. Previous studies (Houweling, M., Cui, Z., and Vance, D. E. (1995) J. Biol. Chem. 270, 16277-16282) showed that expression of wild-type CT-alpha rescued the cells at 40 degrees C, whereas expression of phosphatidylethanolamine N-methyltransferase-2 (PEMT2) did not, even though PC levels appeared to be maintained at wild-type levels after 24 h at the restrictive temperature. We report that the failure of PEMT2 to rescue the MT58 cell line is due to inadequate long term PC synthesis. We found that changing the medium every 24 h rescued the PEMT2-expressing MT58 cells grown at 40 degrees C. This was due to the uptake and utilization of lipids in the serum. At 40 degrees C, PC levels in the wild-type CHO cells and CT-expressing MT58 cells increased over time whereas PC levels did not change in both the MT58 and PEMT2-expressing MT58 cell lines. Further investigation found that both the PEMT2-expressing MT58 and MT58 cell lines accumulated triacylglycerol at 40 degrees C. Pulse-chase experiments indicated that lyso-PC accumulated to a higher degree at 40 degrees C in the PEMT2-expressing MT58 cells compared with CT-expressing MT58 cells. Transfection of the PEMT-expressing MT58 cells with additional PEMT2 cDNA partially rescued the growth of these cells at 40 degrees C. Inhibition of PC degradation, by inhibitors of phospholipases, also stimulated PEMT-expressing MT58 cell growth at 40 degrees C. Best results were observed using a calcium-independent phospholipase A(2) inhibitor, methyl arachidonyl fluorophosphonate. This inhibitor also increased PC mass in the PEMT2-expressing MT58 cells. When the cells are shifted to 40 degrees C, PC degradation by enzymes such as phospholipases is greater than PC synthesis in the mutant PEMT2-expressing MT58 cells. Taken together, these results indicate that PEMT2 expression fails to rescue the mutant cell line at 40 degrees C because it does not maintain PC levels required for cellular replication.

676 ||| 9761739 ||| (4, 0.55) ||| 4 ||| 5 ||| Cytosolic deglycosylation process of newly synthesized glycoproteins generates oligomannosides possessing one GlcNAc residue at the reducing end. Recent studies on the mechanism of degradation of newly synthesized glycoproteins suggest the involvement of a retrotranslocation of the glycoprotein from the lumen of the rough endoplasmic reticulum into the cytosol, where a deglycosylation process takes place. In the studies reported here, we used a glycosylation mutant of Chinese hamster ovary cells that does not synthesize mannosylphosphoryldolichol and has an increased level of soluble oligomannosides originating from glycoprotein degradation. In the presence of anisomycin, an inhibitor of protein synthesis, we observed an accumulation of glucosylated oligosaccharide-lipid donors (Glc3Man5GlcNAc2-PP-Dol), which are the precursors of the soluble neutral oligosaccharide material. Inhibition of rough endoplasmic reticulum glucosidase(s) by castanospermine led to the formation of Glc3Man5GlcNAc2(OSGn2) (in which OSGn2 is an oligomannoside possessing two GlcNAc residues at its reducing end), which was then retained in the lumen of intracellular vesicles. Thus they were protected during an 8 h chase period from the action of cytosolic chitobiase, which is responsible for the conversion of OSGn2 to oligomannosides possessing one GlcNAc residue at the reducing end (OSGn1). In contrast, when protein synthesis was maintained in the presence of castanospermine, glucosylated oligomannosides (Glc1-3Man5GlcNAc1) were recovered in cytosol. Except for monoglucosylated Man5 species, which are potential substrates for luminal calnexin and calreticulin, the pattern of oligomannosides was similar to that observed on glycoproteins. The occurrence in the cytosol of glucosylated species with one GlcNAc residue at the reducing end implies that the deglycosylation process that generates glucosylated OSGn1 from glycoproteins occurs in the cytosol.

677 ||| 9003379 ||| (4, 0.55) ||| 4 ||| 5 ||| Overexpression of phosphatidylethanolamine N-methyltransferase 2 in CHO-K1 cells does not attenuate the activity of the CDP-choline pathway for phosphatidylcholine biosynthesis. Chinese hamster ovary (CHO) cells express only a trace amount of phosphatidylethanolamine N-methyltransferase (PEMT) activity. CHO cells make their phosphatidylcholine (PC) via the CDP-choline pathway. We investigated whether or not overexpression of PEMT2, an isoform of PEMT, in these cells would down-regulate the activity of the CDP-choline pathway. Transfection of CHO cells with PEMT2 cDNA behind the cytomegalovirus promoter resulted in a series of cell lines that overexpressed PEMT2. Phospholipid metabolism was characterized in cell lines that expressed a medium (281 pmol/min per mg of protein) and a high (1300 pmol/min per mg of protein) level of PEMT activity. The expression of the regulated enzyme (CTP:phosphocholine cytidylyltransferase) in the CDP-choline pathway was increased, not decreased, in these cell lines as judged by immunoblot analysis and enzymic activity. Conversion of phosphatidylethanolamine to PC was enhanced in CHO cells that expressed PEMT2 activity. PC mass was not increased in the transfected compared with the control cells. The rate of PC catabolism made by either the CDP-choline or methylation pathways was unaffected by PEMT2 expression. We conclude that expression of PEMT2 in CHO cells does not down-regulate, but rather enhances, the expression of CTP:phosphocholine cytidylyltransferase.

678 ||| 7641798 ||| (4, 0.55) ||| 4 ||| 5 ||| Farnesyl acetate, a derivative of an isoprenoid of the mevalonate pathway, inhibits DNA replication in hamster and human cells. Cells treated with compactin, an inhibitor of 3-hydroxy-3-methylglutaryl coenzyme A (HMG-CoA) reductase, the enzyme which catalyzes the rate-limiting step of the mevalonate pathway, are arrested prior to the DNA synthesis (S) phase of the cell cycle. Identification of a specific pathway product or products with a role in DNA replication, however, has remained elusive. In this report we demonstrate that farnesyl acetate, a derivative of the key isoprenoid pathway intermediate farnesyl pyrophosphate, inhibits DNA replication in both Chinese hamster ovary cells and human (HeLa) cells. This effect is revealed by measurement of DNA content using fluorescence-activated cell sorter analysis and by measurement of [3H]thymidine incorporation. We show that cells treated with farnesyl acetate retain protein synthesis capacity as DNA replication is inhibited and remain intact as viewed with the vital stain propidium iodide. The inhibition of DNA replication by farnesyl acetate occurs in cells treated with high levels of compactin and in cells lacking HMG-CoA reductase. These results indicate that farnesyl acetate action is not dependent on metabolism through the isoprenoid pathway and is not the result of the loss of a metabolite required for replication nor the accumulation of a metabolite which is inhibitory. In addition, cells treated with farnesyl acetate for over 6 h are irreversibly blocked from progressing through S phase, a phenomenon which differs sharply from the results with compactin, removal of which results in synchronous progression through S phase. Farnesyl acetate also blocks protein prenylation in cells, to a degree comparable to a known farnesylation inhibitor, BZA-5B. We propose that farnesyl acetate is acting in a manner quite different from the metabolic block caused by compactin, causing a rapid and irreversible block of DNA replication.

679 ||| 11606046 ||| (4, 0.55) ||| 4 ||| 5 ||| Temperature-sensitive phenotype of Chinese hamster ovary cells defective in PEX5 gene. SK32 mutant cells, which were isolated as peroxisome-deficient Chinese hamster ovary (CHO) cells by an advantage of a visible peroxisome form of green fluorescent protein (GFP), were found to suffer from a functional loss of PEX5 gene encoding for PTS1R. The sequence analysis of cDNA indicated that PEX5 gene encoded for the two isoforms composed of 603 amino acids (PTS1RS) and 640 amino acids (PTS1RL). The mutation changed glycine to arginine at amino acid position 343 of PTS1RL (corresponding to the position 306 of PTS1RS) in SK32 cells. The mutant cells exhibited a temperature-sensitive (TS) phenotype on the peroxisomal localizations of the recombinant GFP and urate oxidase appending a genuine peroxisome targeting signal 1 (PTS1), a tripeptide of Ser-Lys-Leu (SKL) at the C-terminus, but did not on that of catalase harboring a divergent PTS1, Lys-Ala-Asn-Leu (KANL) sequence. 3-ketoacyl-CoA thiolase (hereafter referred to as thiolase), which harbors an extension sequence (PTS2) at the N-terminus, never appeared to be affected on the peroxisomal localization in the mutant cells. When thiolase was examined on the molecular size in the mutant cells, the enzyme existed as the larger precursor form in the peroxisomes at 37 degrees C and a considerable part (almost half) was converted to the mature size at 30 degrees C. These results indicate that the amino acid substitution, Gly306Arg in PTS1RS and/or Gly343Arg in PTSRL, gives rise to TS phenotype on the peroxisomal translocation of PTS1 proteins and the maturation of PTS2 protein.

680 ||| 9880566 ||| (4, 0.54) ||| 4 ||| 5 ||| Isolation of a chinese hamster ovary (CHO) cDNA encoding phosphatidylglycerophosphate (PGP) synthase, expression of which corrects the mitochondrial abnormalities of a PGP synthase-defective mutant of CHO-K1 cells. Phosphatidylglycerophosphate (PGP) synthase catalyzes the first step in the cardiolipin (CL) branch of phospholipid biosynthesis in mammalian cells. In this study, we isolated a Chinese hamster ovary (CHO) cDNA encoding a putative protein similar in sequence to the yeast PGS1 gene product, PGP synthase. The gene for the isolated CHO cDNA was named PGS1. Expression of the CHO PGS1 cDNA in CHO-K1 cells and production of a recombinant CHO PGS1 protein with a N-terminal extension in Escherichia coli resulted in 15-fold and 90-fold increases of PGP synthase specific activity, respectively, establishing that CHO PGS1 encodes PGP synthase. A PGP synthase-defective CHO mutant, PGS-S, isolated previously (Ohtsuka, T., Nishijima, M., and Akamatsu, Y. (1993) J. Biol. Chem. 268, 22908-22913) exhibits striking reductions in biosynthetic rate and cellular content of phosphatidylglycerol (PG) and CL and shows mitochondrial morphological and functional abnormalities. The CHO PGS-S mutant transfected with the CHO PGS1 cDNA exhibited 620-fold and 7-fold higher PGP synthase activity than mutant PGS-S and wild type CHO-K1 cells, respectively, and had a normal cellular content and rate of biosynthesis of PG and CL. In contrast to mutant PGS-S, the transfectant had morphologically normal mitochondria. When the transfectant and mutant PGS-S cells were cultivated in a glucose-depleted medium, in which cellular energy production mainly depends on mitochondrial function, the transformant but not mutant PGS-S was capable of growth. These results demonstrated that the morphological and functional defects displayed by the PGS-S mutant are due directly to the reduced ability to make normal levels of PG and/or CL.

681 ||| 8611395 ||| (4, 0.54) ||| 4 ||| 5 ||| Dipyridamole increases VP16 growth inhibition, accumulation and retention in parental and multidrug-resistant CHO cells. Dipyridamole (DP) has been shown to reverse multidrug resistance (MDR) via interactions with P-glycoprotein (P-gp). The effect of DP on VP16 growth inhibition was investigated in parental (CHO-K1) and MDR (CHO-Adr(r)) Chinese hamster ovary cells. CHO-Adr(r) cells were 18-fold resistant to VP16 and intracellular accumulation was 28% less than in CHO-K1 cells. DP reduced the resistance of CHO-Adr(r) to VP16 by a factor of 2-3 and caused a similar potentiation of VP16 growth inhibition in the parental cells. A time-dependent increase in intracellular VP16 accumulation, which was similar in both cell lines, was caused by DP. The intracellular retention of VP16 was increased 2- to 3-fold by DP in both cell lines. The magnitude of the effect of DP on all three parameters measured was similar (2- to 4-fold), suggesting that the increased growth inhibition was related to increased intracellular exposure to VP16 owing to the inhibition of the efflux of VP16 by DP. However, since the effect of DP was similar in both parental and P-gp-overexpressing cells it is unlikely that the potentiation of VP16 by DP is mediated via an interaction with P-gp.

682 ||| 7608211 ||| (4, 0.54) ||| 4 ||| 5 ||| Phorbol ester abrogates up-regulation of DNA polymerase beta by DNA-alkylating agents in Chinese hamster ovary cells. Mammalian DNA polymerase beta (beta-pol), a DNA repair polymerase, is known to be constitutively expressed in cultured cells, but treatment of cells with the DNA-alkylating agents MNNG or methyl methanesulfonate has been shown to up-regulate beta-pol mRNA level. To further characterize this response, we prepared a panel of monoclonal antibodies and used one of them to quantify beta-pol in whole cell extracts by immunoblotting. We found that treatment of Chinese hamster ovary cells with either DNA-alkylating agent up-regulated the beta-pol protein level 5-10-fold. This induction appeared to be secondary to DNA alkylation, as induction was not observed with a genetically altered cell line overexpressing the DNA repair enzyme O6-methylguanine-methyltransferase. We also found that 12-O-tetradecanoylphorbol-13-acetate (TPA) treatment of wild type Chinese hamster ovary cells increased expression of beta-pol protein (approximately 10-fold). Any interrelationship between this TPA response and the DNA-alkylation response was studied by treatment with combinations of MNNG and TPA. The beta-pol up-regulation observed with MNNG treatment was abrogated by TPA, and conversely the up-regulation observed with TPA treatment was abrogated by MNNG.

683 ||| 21616557 ||| (4, 0.54) ||| 4 ||| 5 ||| Characterisation of a novel glycosylphosphatidylinositol-anchored mono-ADP-ribosyltransferase isoform in ovary cells. The mammalian mono-ADP-ribosyltransferases are a family of enzymes related to bacterial toxins that can catalyse both intracellular and extracellular mono-ADP-ribosylation of target proteins involved in different cellular processes, such as cell migration, signalling and inflammation. Here, we report the molecular cloning and functional characterisation of a novel glycosylphosphatidylinositol (GPI)-anchored mono-ADP-ribosyltransferase isoform from Chinese hamster ovary (CHO) cells (cARTC2.1) that has both NAD-glycohydrolase and arginine-specific ADP-ribosyltransferase activities. cARTC2.1 has the R-S-EXE active-site motif that is typical of arginine-specific ADP-ribosyltransferases, with Glu209 as the predicted catalytic amino acid. When over-expressed in CHO cells, the E209G single point mutant of cARTC2.1 cannot hydrolyse NAD(+), although it retains low arginine-specific ADP-ribosyltransferase activity. This ADP-ribosyltransferase activity was abolished only with an additional mutation in the R-S-EXE active-site motif, with both of the glutamate residues of the EKE sequence of cARTC2.1 mutated to glycine (E207/209G). These glutamate-mutated proteins localise to the plasma membrane, as does wild-type cARTC2.1. Thus, the partial or total loss of enzymatic activity of cARTC2.1 that arises from these mutations does not affect its cellular localisation. Importantly, an endogenous ADP-ribosyltransferase is indeed expressed and active in a subset of CHO cells, while a similar activity cannot be detected in ovarian cancer cells. With respect to this endogenous ecto-ART activity, we have identified two cell populations: ART-positive and ART-negative CHO cells. The subset of ART-positive cells, which represented 5% of the total cells, is tightly maintained in the CHO cell population.

684 ||| 10487535 ||| (4, 0.54) ||| 4 ||| 5 ||| Inhibitory effects of prostaglandin A1 on membrane transport of folates mediated by both the reduced folate carrier and ATP-driven exporters. Studies are reported that describe the multifaceted inhibitory effects of prostaglandin A1 (PGA1) on processes that govern the transport of folates across the plasma membrane of Chinese hamster ovary (CHO) cells: the reduced folate carrier, RFC1, and ATP-dependent exporters. PGA1 was a noncompetitive inhibitor of MTX influx mediated by RFC1 with a Ki of approximately 21 microM. The onset of inhibition was virtually instantaneous, not reversible, and appeared to require the incorporation of PGA1 into the lipid membrane; surface adsorption alone was insufficient for inhibition of RFC1 transport activity. In contrast, the effect of PGA1 on folic acid transport was small (approximately 20% inhibition of total influx), consistent with the observation that the major portion of folic acid transport in CHO cells is mediated by a low pH mechanism distinct from RFC1. PGA1 was also a potent inhibitor of the ATP-driven efflux of both MTX and folic acid. At a concentration of 7 microM PGA1, the efflux rate constants for these folates were depressed by approximately 70 and approximately 50%, respectively. The net effects of PGA1 on the bidirectional folate fluxes translated into marked alterations in net transport. The addition of 7 microM PGA1 to cells at steady state with 1 microM MTX produced a rapid onset of net uptake and the achievement of an approximately 3-fold increase in the steady-state free MTX level as compared with untreated CHO cells. The addition of 7 microM PGA1 to cells at steady state with 1 microM folic acid produced an approximately 5-fold increase in the free folate level. These studies establish PGA1 as a potent inhibitor of both the reduced folate carrier and ATP-driven folate exporter(s). The noncompetitive nature of the inhibition of RFC1 is unique among anionic compounds, which are usually competitive inhibitors of the carrier.

685 ||| 9359869 ||| (4, 0.53) ||| 4 ||| 5 ||| Rapid induction of apoptosis by deregulated uptake of polyamine analogues. Treatment of Chinese hamster ovary cells with alpha-difluoromethylornithine for 3 days, followed by exposure to cycloheximide, led to an unregulated, rapid and massive accumulation of polyamine analogues. This accumulation led to cell death by apoptosis within a few hours. Clear evidence of DNA fragmentation was seen in response to both N-terminally ethylated polyamines and to polyamines containing methyl groups on the terminal carbon atoms. Programmed cell death was induced within 2-4 h of exposure to 1 microM or higher concentrations of N1,N11-bis(ethyl)norspermine. The presence of cycloheximide increased the uptake of the polyamine analogues and therefore led to cell death at lower analogue concentrations, but it was not essential for the induction of apoptosis, since similar effects were seen when the protein synthesis inhibitor was omitted and the concentration of N1, N11-bis(ethyl)norspermine was increased to 5 microM or more. The induction of apoptosis was blocked both by the addition of the caspase inhibitor N-benzyloxycarbonyl-Val-Ala-Asp-fluoromethylketone, or by the addition of the polyamine oxidase inhibitor N1-methyl-N2-(2,3-butadienyl)butane-1,4-diamine (MDL 72,527). These experiments provide evidence to support the concepts that: (1) polyamines or their oxidation products may be initiators of programmed cell death; (2) regulation of polyamine biosynthesis and uptake prevents the accumulation of toxic levels of polyamines; and (3) the anti-neoplastic effects of bis(ethyl) polyamine analogues may be due to the induction of apoptosis in sensitive tumour cells.

686 ||| 21552977 ||| (4, 0.53) ||| 4 ||| 5 ||| Characterization of an altered DNA topoisomerase ii-alpha from a mitoxantrone resistant Mammalian-cell line hypersensitive to DNA cross-linking agents. To further define the molecular basis for drug resistance to mitoxantrone, a Chinese hamster ovary cell line (MXN(4)) was selected in the presence of 25 nM mitoxantrone and fully characterized. This cell line is 20-fold resistant to mitoxantrone, cross-resistant to several other topoisomerase II poisons, and 2- to 3-fold collaterally sensitive to cisplatin, carboplatin and BCNU. Neither an alteration in cellular uptake of topoisomerase II inhibitor nor overexpression of P-glycoprotein contribute to the drug resistance of MXN(4) cells. Immunoblotting demonstrates equivalent amounts of topoisomerase II alpha and beta in the wild-type and drug resistant cell lines, suggesting that a quantitative alteration in topoisomerase II is not the mechanism of resistance of MXN(4) cells. Mitoxantrone-induced DNA double strand breaks measured in situ were attenuated 28-fold in the drug resistant cell line. Nuclear extracts of MXN(4) cells, as well as topoisomerase II alpha purified to homogeneity from these cells, were found to be markedly resistant to drug-induced covalent DNA: topoisomerase II complex formation. The catalytic activity of purified MXN(4) topoisomerase II was the same as wild-type activity. Thus, the resistance of MXN(4) cells to mitoxantrone involves the expression of a topoisomerase II alpha with altered DNA cleavage activity. The hypersensitivity of this cell line to platinum analogs is due to an apparent increased uptake of these drugs which results in augmented DNA interstrand crosslinking.

687 ||| 10651037 ||| (4, 0.53) ||| 4 ||| 5 ||| Crystal structure of CHO reductase, a member of the aldo-keto reductase superfamily. Chinese hamster ovary (CHO) reductase is an enzyme belonging to the aldo-keto reductase (AKR) superfamily that is induced by the aldehyde-containing protease inhibitor ALLN (Inoue, Sharma, Schimke, et al., J Biol Chem 1993;268: 5894). It shows 70% sequence identity to human aldose reductase (Hyndman, Takenoshita, Vera, et al., J Biol Chem 1997;272:13286), which is a target for drug design because of its implication in diabetic complications. We have determined the crystal structure of CHO reductase complexed with nicotinamide adenine dinucleotide phosphate (NADP)+ to 2.4 A resolution. Similar to aldose reductase and other AKRs, CHO reductase is an alpha/beta TIM barrel enzyme with cofactor bound in an extended conformation. All key residues involved in cofactor binding are conserved with respect to other AKR members. CHO reductase shows a high degree of sequence identity (91%) with another AKR member, FR-1 (mouse fibroblast growth factor-regulated protein), especially around the variable C-terminal end of the protein and has a similar substrate binding pocket that is larger than that of aldose reductase. However, there are distinct differences that can account for differences in substrate specificity. Trp111, which lies horizontal to the substrate pocket in all other AKR members is perpendicular in CHO reductase and is accompanied by movement of Leu300. This coupled with movement of loops A, B, and C away from the active site region accounts for the ability of CHO reductase to bind larger substrates. The position of Trp219 is significantly altered with respect to aldose reductase and appears to release Cys298 from steric constraints. These studies show that AKRs such as CHO reductase are excellent models for examining the effects of subtle changes in amino acid sequence and alignment on binding and catalysis.

688 ||| 9497322 ||| (4, 0.52) ||| 4 ||| 5 ||| Functional expression of the chicken low density lipoprotein receptor-related protein in a mutant chinese hamster ovary cell line restores toxicity of Pseudomonas exotoxin A and degradation of alpha2-macroglobulin. The low density lipoprotein receptor-related protein (LRP) is responsible for the clearance of several physiological ligands including a complex of proteinase and alpha2-macroglobulin (alpha2M) and for the entrance of Pseudomonas exotoxin A (PEA) into cells. We have prepared expression plasmids for the full-length chicken LRP (designated LRP100) and two intermediates encoding 25 and 67% of the receptor (designated LRP25 and LRP67, respectively) using overlapping cDNA fragments. LRP25 and LRP67 encode the N-terminal 22 and 64%, respectively, of LRP100 plus the transmembrane and intracellular domains. Transient transfection of these plasmids into COS-7 cells yielded recombinant proteins of expected molecular mass and immunoreactivity. However, LRP100 was incompletely processed into alpha- (515-kDa) and beta- (85-kDa) chains and was poorly transported from the endoplasmic reticulum to the Golgi compartment. Stable transformants of LRP100, LRP67, and LRP25 were generated in a mutant Chinese hamster ovary cell line that lacked expression of endogenous LRP and was resistant to PEA. All forms of recombinant LRP proteins were transported from the endoplasmic reticulum to the Golgi apparatus in Chinese hamster ovary cells as shown by their sensitivity to endoglycosidase H and resistance to neuraminidase. Cell surface iodination and subcellular fractionation studies indicated that all three LRP variants were expressed on the plasma membrane. Furthermore, expression of the three LRP variants restored, to various degrees, sensitivity to PEA and the ability to degrade methylamine-activated alpha2M (alpha2M\*). These data suggest that deletion of large internal portions of LRP, including the processing site, does not prevent transport of LRP to the plasma membrane, nor does it abolish the interaction of LRP with alpha2M\* or PEA. This LRP expression system may allow for the characterization of domains within LRP responsible for its multifunctionality.

689 ||| 8663247 ||| (4, 0.52) ||| 4 ||| 5 ||| A genetic defect in phosphatidylcholine biosynthesis triggers apoptosis in Chinese hamster ovary cells. We have investigated the cell death of a Chinese hamster ovary mutant (MT-58) with a thermo-sensitive CTP:phosphocholine cytidylyltransferase, the rate-limiting enzyme of the CDP-choline pathway for phosphatidylcholine biosynthesis (Esko, J. D., Wermuth, M. M., and Raetz, C. R. H. (1981) J. Biol. Chem. 256, 7388-7393). After MT-58 cells were shifted to the restrictive temperature of 40 degrees C, the cytidylyltransferase was inactivated immediately leading to a decrease in phosphatidylcholine biosynthesis and cell death. DNA content and number of cells in the S phase decreased significantly in the dying MT-58 cells according to flow cytometrical analyses. The fragmentation of genomic DNA was detected by DNA ladders in agarose gel and release of the prelabeled genomic DNA into cytosolic fractions 14 h after the temperature shift. The dying cells underwent a dramatic reduction of cellular volume while maintaining the membrane containment of cellular contents. These events indicated that the inactivation of cytidylyltransferase triggered apoptosis in Chinese hamster ovary cells. This is the first report that apoptosis was induced in cultured cells, not by an added agent, but by a mutation in phospholipid biosynthesis.

690 ||| 16237731 ||| (4, 0.52) ||| 4 ||| 5 ||| Deregulation of cyclin-dependent kinase 2 activity correlates with UVC-induced apoptosis in Chinese hamster ovary cells. Progression through the cell cycle relies on the activities of cyclin-dependent kinases (Cdk), which in turn are modulated by inhibitory proteins such as p21(waf1/cip1) that are induced when genomic damage occurs. In this study, we show that exposure of normal mammalian cells, such as NIH3T3 fibroblasts, to UVC (25 J/m2, at 254 nm) induces the expression of p21 without causing significant apoptosis, whereas similar treatment of Chinese hamster ovary (CHO-K1) cells with UVC causes apoptosis without inducing p21. The absence of p21 in UV-irradiated CHO-K1 cells is accompanied by the deregulation of Cdk2 activity. The elevation of Cdk2 activity correlates with the increase of UV-induced apoptosis, which can be suppressed by small-molecule Cdk2 inhibitors such as roscovitine and pyrrolidine dithiocarbamate. The results of this study suggest that the deregulation of Cdk2 activity may be critical to UV-induced apoptosis in CHO-K1 cells.

691 ||| 11900549 ||| (4, 0.52) ||| 4 ||| 5 ||| Pentavalent ions dependency is a conserved property of adenosine kinase from diverse sources: identification of a novel motif implicated in phosphate and magnesium ion binding and substrate inhibition. The catalytic activity of adenosine kinase (AK) from mammalian sources has previously been shown to exhibit a marked dependency upon the presence of pentavalent ions (PVI), such as phosphate (PO4), arsenate, or vanadate. We now show that the activity of AK from diverse sources, including plant, yeast, and protist species, is also markedly enhanced in the presence of PVI. In all cases, PO4 or other PVI exerted their effects primarily by decreasing the Km for adenosine and alleviating the inhibition caused by high concentrations of substrates. These results provide evidence that PVI dependency is a conserved property of AK and perhaps of the PfkB family of carbohydrate kinases which includes AK. On the basis of sequence alignments, we have identified a conserved motif NXXE within the PfkB family. The N and E of this motif make close contacts with Mg2+ and PO4 ions in the crystal structures of AK and bacterial ribokinase (another PfkB member which shows PVI dependency), implicating these residues in their binding. Site-directed mutagenesis of these residues in Chinese hamster AK have resulted in active proteins with greatly altered phosphate stimulation and substrate inhibition characteristics. The N239Q mutation leads to the formation of an active protein whose activity was not stimulated by PO4 or inhibited by high concentrations of adenosine or ATP. The activity of the E242D mutant protein was also not significantly altered in the presence of phosphate. Although PO4 had no effect on the KmAdenosine for this mutant, the KmATP, K(i)Adenosine, and K(i)ATP were significantly decreased. In contrast to these mutations, N239L or E242L mutant proteins showed greatly decreased activity with an altered Mg2+ requirement. These observations support the view that N239 and E242 play an important role in the binding of PO4 and Mg2+ ions required for the catalytic activity of adenosine kinase.

692 ||| 11522300 ||| (4, 0.52) ||| 4 ||| 5 ||| Phosphatidylserine induces apoptosis in CHO cells without mitochondrial dysfunction in a manner dependent on caspases other than caspases-1, -3, -8 and -9. Treatment of Chinese hamster ovary K1 cells with phosphatidylserine (PS) caused typical apoptosis with distinct morphological and biochemical features in a dose- and time-dependent manner. However, unlike camptothecin-induced apoptosis, changes in mitochondrial transmembrane potential were not observed. In addition, cytochrome c release did not occur in PS-induced apoptosis. A pan caspase inhibitor, Z-VAD, significantly inhibited the apoptosis, but inhibitors of caspase-1, -3, -8 and -9 did not. Activities of caspase-1, -3, -8 and -9 were increased by treatment of the cells with camptothecin, but not with PS. These results suggest that PS-induced apoptosis occurs without the collapse of mitochondrial transmembrane potential and without the release of cytochrome c, in a manner independent of caspase-1, -3, -8 and -9.

693 ||| 9407052 ||| (4, 0.51) ||| 4 ||| 5 ||| Heat shock increases the association of binding protein-1 with initiation factor 4E. The effects of heat shock on the regulation of the cap-binding initiation factor 4E (eIF4E) and its inhibitory binding protein, 4E-BP1, have been examined in Chinese hamster ovary cells and in cardiac myocytes. Heat shock increased the association between eIF4E and 4E-BP1, and this was associated with a dephosphorylation of 4E-BP1. These effects did not appear to be due wholly to decreased activity of the p70 S6 kinase pathway, which is implicated in the control of 4E-BP1, and they were not mediated by the stress-activated p38 microtubule-associated protein kinase pathway. Increased binding of 4E-BP1 to eIF4E correlated with a decrease in the amount of eIF4G which co-purified with the latter. This could account for the previously observed impairment of eIF4F function during heat shock, and, since heat shock protein mRNAs are believed to be relatively cap-independent, could provide a mechanism for the selective up-regulation of the synthesis of heat shock proteins and other stress proteins during heat shock.

694 ||| 9015161 ||| (4, 0.51) ||| 4 ||| 5 ||| Molecular spectrum of mutations induced by 5-hydroxymethyl-2'-deoxyuridine in (CHO)-PL61 cells. We have utilized (CHO)-PL61 cells to characterize the mutations produced in mammalian cells by exogenous treatment with the nucleoside 5-hydroxymethyl-2'-deoxyuridine (hmdUrd). HmdUrd is incorporated into DNA as a thymidine analogue and is removed by the repair enzyme hmUra-DNA glycosylase. PL61 cells are hprt(-) and contain adjacent single copies of the Escherichia coli gpt and neo genes (gpt+, neo+) separated by 2 kb, rendering the cells thioguanine sensitive (TGs) and geneticin resistant (G418r). Cells were exposed to hmdUrd and the colonies resistant to thioguanine or thioguanine and G418 were selected. Selection in thioguanine alone (TGr/gpt(-)) allows the growth of all gpt(-) mutants (small, intermediate and large deletions/insertions and point mutations) while selection in thioguanine and G418 (TGr/gpt(-), G418r/neo+) prevents survival of colonies containing vary large deletions of the gpt gene that include the neo gene. To confirm the types of mutation at the molecular level, the gpt gene was amplified from mutants' genomic DNA by PCR, and the amplified DNA was sequenced directly by the dideoxy method. Our study showed that 4 microM hmdUrd induced mutations to TGr/gpt(-) at a rate 3-4 times that of control, but showed no marked increase in mutation to TGr/gpt(-), G418r/neo+. The predominant type of hmdUrd induced mutation in the thioguanine resistant cells at the gpt locus was complete loss of the gpt gene resulting from a large deletion. Background mutations were generally point mutations or small insertion/deletion mutations. We propose that hmdUrd induces large/intermediate deletions as a major type of mutations in mammalian cells as a consequence of DNA repair, and not as a result of misincorporation or mispairing, suggesting that base excision repair by itself can lead to large deletion mutagenesis.

695 ||| 8791009 ||| (4, 0.50) ||| 4 ||| 5 ||| Stable expression of the recombinant human VIP1 receptor in clonal Chinese hamster ovary cells: pharmacological, functional and molecular properties. We stably transfected Chinese hamster ovary (CHO) cells with the recombinant human vasoactive intestinal peptide (VIP)1 receptor. A clone referred to as Clone 15 was isolated and studied for receptor properties. The following data were obtained: (1) one class of binding site was identified by Scatchard analysis of [125I]VIP binding to cell membranes with a Kd of 0.41 nM and a Bmax of 1.62 pmol/mg protein; (2) the constant Ki for the inhibition of [125I]VIP binding by VIP and related peptides was: VIP (0.9 nM) = pituitary adenylate cyclase-activating peptide (PACAP)-27 (1.3 nM) < PACAP-38 (6.8 nM) < helodermin (46.0 nM) < human growth hormone-releasing factor (GRF) (0.6 microM) < peptide histidine methionineamide (2.0 microM) < secretin (> 10 microM); (3) cross-linking experiments using [125I]VIP identified a single M(r) 67000 recombinant receptor; (4) VIP stimulated cAMP production in Clone 15 cells with an EC50 of 0.20 nM; (5) some previously described VIP receptor antagonists including [4-Cl-D-Phe6, Leu17]VIP, [Ac-Tyr1,D-Phe2]GRF-(1-29) amide and VIP-(10-28) inhibited [125I]VIP binding with a Ki of 0.7, 1.6 and 2.5 microM, respectively. They failed to stimulate cAMP production in Clone 15 cells and inhibited, at least partially, the VIP (0.3 nM)-evoked cAMP production; (6) exposure of Clone 15 cells to 10 nM VIP for 24 h resulted in a sharp decrease in Bmax in Clone 15 cells (0.43 vs. 1.62 pmol/mg protein in control cells) and in the potency and efficacy of VIP in stimulating cAMP. Moreover, immunofluorescence studies using confocal microscopy indicated that the receptor was internalized and sequestered in vesicular structures within the cells. It is concluded that Clone 15 cells provide a valuable tool to further characterize various functional and pharmacological aspects of the human VIP1 receptor.

696 ||| 8611185 ||| (4, 0.50) ||| 4 ||| 5 ||| Evidence for intracellular partitioning of serine and glycine metabolism in Chinese hamster ovary cells. Serine hydroxymethyltransferase (SHMT) is the primary enzyme in the interconversion of serine and glycine. The roles of mitochondrial and cytosolic SHMT in the interconversion of serine and glycine were determined in two Chinese hamster ovary (CHO) cell lines that both contain cytosolic SHMT but either have (CHOm+) or lacK (CHOm-) mitochondrial SHMT. Mitochondrial SHMT activity was significantly reduced in CHOm- (0.24 +/- 0.11 nmol/min per mg of mitochondrial protein) compared with CHOm+ (3.21 +/- 0.66 nmol/min per mg of mitochondrial protein; P = 0.02) cells, whereas cytosolic SHMT activity was similar in CHOm- and CHOm+ cells (1.09 +/- 0.31 and 1.53 +/- 0.12 nmol/min per mg of cytosolic protein respectively; P = 0.57). In CHOm+ and CHOm- cells, the relative flux of glycine to serine measured with either [1-13C]- or [2-13C]-glycine was similar (CHOm-: 538 +/- 82 nmol/24 per mg of DNA; CHOm+: 616 +/- 88 nmol/24 h per mg of DNA; P = 0.42). In contrast, the relative flux of serine to glycine measured with [1-13C]serine was low in CHOm- cells (80 +/- 28 nmol/24 h per mg of DNA) compared with CHOm+ cells (3080 +/- 320 nmol/24 h per mg of DNA; P = 0.0001). The rate of glycine production determined by [1-(13)C]glycine dilution was lower in CHOm- (1200 +/- 200 nmol/24 h per mg of DNA) than CHOm+ (10200 +/- 1800 nmol/24 h per mg of DNA; P = 0.03) cells, whereas glycine utilization was similar in the two cell lines. Serine production was similar in the two cell lines but serine utilization was lower in CHOm- (3800 +/- 1200 mu mol/24 h per mg of DNA) than CHOm+ (6600 +/- 1000 nmol/24 h per mg of DNA; P = 0.0002) cells. Increasing the serine concentration in the medium resulted in an increase in glycine production in CHOm+ but not in CHOm- cells. Intracellular studies with [1-13C]serine confirm the findings of decreased glycine production from serine. In CHO cells there is partitioning of intracellular serine and glycine metabolism. Our data support the hypothesis that mitochondrial SHMT is the primary pathway for serine into glycine interconversion.

697 ||| 7677755 ||| (4, 0.50) ||| 4 ||| 5 ||| Participation of a proton-cotransporter, MCT1, in the intestinal transport of monocarboxylic acids. A molecular mechanism for the intestinal monocarboxylic acid transport was characterized by using a proton/monocarboxylate transporter, MCT1, in Chinese hamster ovary (CHO) cells, first found by Garcia et al. (Cell, 76, 865-873, 1994). Northern blotting analysis showed that MCT1-isomers exist in the rat and rabbit intestinal enterocytes and Caco-2 cells. The expression of [14C]lactic acid uptake by Xenopus laevis oocytes injected with rabbit intestinal mRNA was reduced by hybridizing the mRNA with a MCT1 cDNA of CHO cells before microinjection used as the antisense DNA. [14C]Lactic acid uptake by CHO cells was pH dependent, saturable, stereospecific, and reduced in the presence of acetic acid, benzoic acid, S- and R-ibuprofen, S- and R-mandelic acid, nicotinic acid, pravastatin, propionic acid and valproic acid. In addition, several monocarboxylic acids were transported in pH-dependent and saturable manners. These results suggest that the intestinal MCT1-related protein contributes to a carrier-mediated absorption for organic weak acid compounds.

698 ||| 11513727 ||| (4, 0.50) ||| 4 ||| 5 ||| Biosynthesis and intracellular post-translational processing of normal and mutant platelet glycoprotein GPIb-IX. The multisubunit leucine-rich glycoprotein (GP) Ib-IX-V complex mediates von Willebrand factor-dependent platelet adhesion at sites of blood-vessel injury. Molecular defects of this receptor are reported to cause the Bernard-Soulier haemorrhagic disorder. To gain insight into the mechanisms controlling expression of normal and defective receptors, we performed pulse-chase metabolic studies and detailed analysis of intracellular processing in GPIb-IX-transfected Chinese-hamster ovary cells. In the native complex, after early subunit association, sugars N-linked to the three subunits are trimmed and sialylated in the Golgi compartment and GPIbalpha undergoes extensive O-glycosylation. Surface biotinylation during chase demonstrated that only fully processed complexes reach the cell surface. Tunicamycin treatment revealed that early N-glycosylation is not required for O-glycosylation of GPIbalpha and surface expression of the complex. Biosynthetic studies were then performed on a Bernard-Soulier variant based on previous description of abnormal GPIbalpha size and decreased surface expression. The mutant complex associated normally, but displayed defective processing of its N-linked sugars and abnormal O-glycosylation of GPIbalpha. Confocal immunofluorescence microscopy revealed that the mutant complexes could reach the cell surface but also accumulated intracellularly, while use of compartment specific markers showed strong co-localization in the endoplasmic reticulum (ER) and ER-to-Golgi intermediate compartments ('ERGIC') and only slight labelling of the cis-Golgi. Blockade before the Golgi was confirmed by brefeldin A treatment, which restored O-glycosylation and processing of N-linked sugars. The present study has shown that transfer from the ER to the Golgi represents an important step for controlling post-translational processing and surface expression of normal GPIb-IX-V complex.

699 ||| 23468612 ||| (4, 0.49),(8, 0.33) ||| 4 ||| 5 ||| Dynamic modeling of cell migration and spreading behaviors on fibronectin coated planar substrates and micropatterned geometries. An integrative cell migration model incorporating focal adhesion (FA) dynamics, cytoskeleton and nucleus remodeling, actin motor activity, and lamellipodia protrusion is developed for predicting cell spreading and migration behaviors. This work is motivated by two experimental works: (1) cell migration on 2-D substrates under various fibronectin concentrations and (2) cell spreading on 2-D micropatterned geometries. These works suggest (1) cell migration speed takes a maximum at a particular ligand density (~1140 molecules/microm(2)) and (2) that strong traction forces at the corners of the patterns may exist due to combined effects exerted by actin stress fibers (SFs). The integrative model of this paper successfully reproduced these experimental results and indicates the mechanism of cell migration and spreading. In this paper, the mechanical structure of the cell is modeled as having two elastic membranes: an outer cell membrane and an inner nuclear membrane. The two elastic membranes are connected by SFs, which are extended from focal adhesions on the cortical surface to the nuclear membrane. In addition, the model also includes ventral SFs bridging two focal adhesions on the cell surface. The cell deforms and gains traction as transmembrane integrins distributed over the outer cell membrane bond to ligands on the ECM surface, activate SFs, and form focal adhesions. The relationship between the cell migration speed and fibronectin concentration agrees with existing experimental data for Chinese hamster ovary (CHO) cell migrations on fibronectin coated surfaces. In addition, the integrated model is validated by showing persistent high stress concentrations at sharp geometrically patterned edges. This model will be used as a predictive model to assist in design and data processing of upcoming microfluidic cell migration assays.

700 ||| 9043639 ||| (4, 0.49),(1, 0.37) ||| 4 ||| 5 ||| The effect of alpha-tocopherol and beta-tocopherol on proliferation, protein kinase C activity and gene expression in different cell lines. alpha-Tocopherol, but not beta-tocopherol, negatively regulates proliferation of A7r5 vascular smooth muscle cells at physiological concentration. The HeLa cell line was not affected whereas the Chinese hamster ovary cell line (CHO) was slightly inhibited by both alpha-tocopherol and beta-tocopherol. In A7r5 cells alpha-tocopherol inhibited protein kinase C activity, and this correlated with inhibition of proliferation. beta-Tocopherol did not inhibit either protein kinase C or proliferation. In HeLa cells no inhibition by alpha-tocopherol or beta-tocopherol of protein kinase C activity and cell proliferation was observed. In Chinese hamster ovary cells both tocopherols inhibited protein kinase C activity but not proliferation. Thus in the latter cells proliferation was not protein kinase C-dependent. In A7r5 cells alpha-tocopherol but not beta-tocopherol activated AP-1-mediated gene expression. In HeLa cells no change in gene expression was observed in agreement with the finding that also protein kinase C was not affected. In CHO cells gene-expression was activated by both alpha-tocopherol and beta-tocopherol. In this case also a positive correlation was found with similar inhibition of protein kinase C activity. In these cells, however, the changes at the level of protein kinase C activity and gene expression did not result in proliferation changes. The effect of alpha-tocopherol and beta-tocopherol on protein kinase C activity and gene expression suggest a cause-to-effect relationship. Inhibition of proliferation, however, correlates in the case of A7r5 and HeLa cells but not in the case of CHO suggesting a different proliferation pathway for these cells.

701 ||| 11870603 ||| (4, 0.48),(2, 0.33) ||| 4 ||| 5 ||| Induction of protein aggregation in an early secretory compartment by elevation of expression level. A variety of valuable therapeutic proteins are expressed in mammalian cells. Currently, rate-limiting for secretion of recombinant glycoproteins are activities in the secretory pathway of eukaryotic cells, i.e., folding and glycosylation of the naked polypeptide chain. In this paper we provide evidence that elevation of expression level alone is sufficient to cause intracellular aggregation of a structurally relatively simple glycoprotein, antithrombin III (ATIII). Elevation of expression level by selection for increased drug resistance in Chinese hamster ovary cells stably expressing ATIII resulted in formation of disulfide-bonded aggregates of ATIII. Aggregated ATIII displayed incomplete sialylation and Endo H-sensitivity and located to the endoplasmic reticulum and the cis-Golgi compartment in subcellular fractionations. To explore possible causes for aggregation of ATIII at elevated expression levels we investigated the influence of the two major energy sources of cultured mammalian cells, D-glucose and L-glutamine, on the ATIII-yield. We found that utilization of D-glucose was not limiting for synthesis of ATIII at elevated expression levels. However, the amount of ATIII-synthesized per L-glutamine consumed did not seem to increase steadily with expression level for ATIII, indicating that secretion of ATIII may be limited by the capacity of the cell to utilize L-glutamine.

702 ||| 9637787 ||| (4, 0.48) ||| 4 ||| 5 ||| Nucleotide excision repair is not required for the antiapoptotic function of insulin-like growth factor 1. The expression of ERCC1, a member of the nucleotide excision repair (NER) family, is enhanced in cells transfected with insulin-like growth factor 1 (IGF-1) receptors. Of interest, an excellent concordance between ERCC1 expression and NER-mediated cell survival has been demonstrated. The two aims of the present study were to determine the signaling pathways used by IGF-1 to confer protection against apoptotic cell death in Chinese hamster ovary (CHO) cells and to assess the role of NER in this IGF-1 action. Experiments with pharmacological inhibitors indicated that phosphatidylinositol 3-kinase (PI 3-kinase) but not mitogen-activated protein kinase (ERK1/ERK2) mediates IGF-1 antiapoptotic activity. Using two series of CHO cells that have altered expression of ERCC1 or XPB/ERCC3, we examined IGF-1's ability to delay apoptotic death and reduction of mitochondrial oxidative function mediated by growth factor withdrawal. IGF-1 effectively blocked apoptosis, concomitant with increased MTT activity, in a pair of CHO cell lines expressing inactive ERCC1 (43-3B cells) and the transfected line of the mutant carrying the expressed human ERCC1 gene (83-G5 cells). Similarly, repair-deficient UV24 cells, which lack XPB/ERCC3, and their parental line AA8 were also responsive to the IGF-1's antiapoptotic capacity. In the presence of IGF-1, these cell lines became resistant to the cleavage of poly(ADP-ribose) polymerase, a key player in DNA damage recognition and DNA repair. These results suggest that PI 3-kinase activation plays a determinant role in the antiapoptotic function of IGF-1, but that functional NER does not play a critical part in mediating this IGF-1 response.

703 ||| 11788599 ||| (4, 0.48) ||| 4 ||| 5 ||| Mutation in the glucose-6-phosphate dehydrogenase gene leads to inactivation of Ku DNA end binding during oxidative stress. Glucose-6-phosphate dehydrogenase (G6PD), the rate-limiting enzyme of the oxidative pentose phosphate cycle, regulates the NADPH/NADP(+) ratio in eukaryotic cells. G6PD deficiency is one of the most common mutations in humans and is known to cause health problems for hundreds of millions worldwide. Although it is known that decreased G6PD functionality can result in increased susceptibility to oxidative stress, the molecular targets of this stress are not known. Using a Chinese hamster ovary G6PD-null mutant, we previously demonstrated that exposure to a thiol-specific oxidant, hydroxyethyldisulfide, caused enhanced radiation sensitivity and an inability to repair DNA double strand breaks. We now demonstrate a molecular mechanism for these observations: the direct inhibition of DNA end binding activity of the Ku heterodimer, a DNA repair protein, by oxidation of its cysteine residues. Inhibition of Ku DNA end binding was found to be reversible by treatment of the nuclear extract with dithiothreitol, suggesting that the homeostatic regulation of reduced cysteine residues in Ku is a critical function of G6PD and the oxidative pentose cycle. In summary, we have discovered a new layer of DNA damage repair, that of the functional maintenance of repair proteins themselves. In view of the rapidly escalating number of roles ascribed to Ku, these results may have widespread ramifications.

704 ||| 15501434 ||| (4, 0.47),(5, 0.43) ||| 4 ||| 5 ||| Effect of cadmium on cell cycle progression in Chinese hamster ovary cells. Chinese hamster ovary K1 (CHO K1) cells are very sensitive to cadmium (Cd) toxicity. They were used to investigate the effect of Cd on cell cycle progression. Cells were cultured with 0.1, 0.4, 1 or 4 microM Cd for various time intervals. There was no difference in growth rate when less than 0.4 microM Cd was given within 24 h. A dose-dependent reduction of cell proliferation was observed when more than 0.4 microM of Cd was given. The cells were pulse-labeled with 5-bromodeoxyuridine (BrdU), and the labeled cells were cultured in the presence of increasing concentrations of Cd. Cell cycle progression was retarded as a function of Cd concentration. G2/M arrest was observed when the BrdU-labeled cells were treated with 1 microM Cd for 8h, whereas cells receiving 4 microM Cd stopped at the S phase within 4 h. Cell cycle analysis of cells treated with Cd for 24 h showed that G2/M arrest occurred only when cells received 0.8 to 2 microM Cd. Despite the occurrence of G2/M arrest in the Cd treatment, only a limited proportion of the cells were blocked in the M phase. However, the increase in M phase cells coincided with an elevation in the cyclin-dependent kinase 1 activity. To examine whether Cd acts on cells at a specific cell stage, they were synchronized at the G1 or G2/M phase then treated with 1 microM Cd for 12 h. The cells were blocked at the G2/M and G1/S phase, respectively. This finding indicates that Cd toxicity is global and not cell phase specific. We also investigated the involvement of Cd-induced reactive oxygen species (ROS) with the occurrence of G2/M block and found a lack of correlation between cell cycle arrest and ROS production. We measured the Cd content that caused G2/M arrest from a series of Cd treatments and determined the ranges of cumulative Cd concentrations that could result in cell cycle arrest.

705 ||| 18162584 ||| (4, 0.47),(2, 0.33) ||| 4 ||| 5 ||| StARD13(Dlc-2) RhoGap mediates ceramide activation of phosphatidylglycerolphosphate synthase and drug response in Chinese hamster ovary cells. To identify genes involved in etoposide drug response, we used promoter trap mutagenesis to isolate an etoposide-resistant Chinese hamster ovary (CHO) cell line. This resistant CHO-K1 line, named E91, showed cross-resistance to C(2)-ceramide (N-acetylsphingosine). The promoter trap retrovirus was found integrated into intron 1-2 of the Dlc-2 (Stard13) RhoGap gene. The E91 cells showed elevated guanosine triphosphate (GTP)-bound RhoA levels compared with the parental line, suggesting that retrovirus integration had inactivated one of the Dlc-2 RhoGap alleles. To test whether E91 cells were impaired in an intracellular ceramide-regulated process not directly related to cell killing, we measured mitochondrial phosphatidylglycerolphosphate (PGP) synthase and phospholipase A2 enzyme activities in cells after C(2)-ceramide addition. Parental cells showed elevated enzyme activities after treatment with C(2)-ceramide or tumor necrosis factor alpha, but not the E91 cells. These results suggested that intracellular ceramide signaling was defective in E91 cells due to increased levels of active GTP-bound RhoA. RNA knockdown experiments of the Dlc2 RhoGap resulted in increased GTP-bound RhoA and reduced induction of PGP synthase after C(2)-ceramide addition compared with controls. Expression of a dominant-negative RhoA in the E91 cell line allowed induction of PGP synthase by ceramide. The RNA interference knockdown cell line also showed increased etoposide resistance. This study is the first report for the regulation of a phospholipid biosynthetic enzyme through RhoGap expression.

706 ||| 9653452 ||| (4, 0.47) ||| 4 ||| 5 ||| Proficient deoxyribonucleic acid repair of methylation damage in hamster ERCC-gene mutants. Three major pathways, nucleotide excision repair (NER), base excision repair (BER) and O6-methylguanine-DNA methyltransferase (MGMT), are responsible for the removal of most adducts to DNA and thus for the survival of cells influenced by deoxyribonucleic acid (DNA) adduct-forming chemicals. We have evaluated host cell reactivation and cell survival of wild type Chinese hamster ovary cells and of mutants in the NER-genes ERCC1, ERCC2, and ERCC4 after treatment with the methylating compounds dimethylsulfate and methylnitrosourea. No effect of the three genes could be demonstrated, i.e., survival and host cell reactivation after methylation damage in the mutants and the wild type cells were similar. Gene-specific repair experiments confirmed the proficient removal of methyl lesions. We conclude that the three nucleotide excision repair genes are immaterial to the repair of methylation damage. This suggests that NER does not play a role in the removal of methylation in mammalian cells and that BER and MGMT are responsible for the survival of such cells, when they are challenged with methylation of DNA.

707 ||| 7782327 ||| (4, 0.47) ||| 4 ||| 5 ||| Isolation and characterization of a stable Chinese hamster ovary cell line overexpressing the plasma membrane Ca(2+)-ATPase. Stable Chinese hamster ovary (CHO) cell lines overexpressing the human plasma membrane Ca(2+)-ATPase (PMCA) were generated, and three independent cell clones were characterized in details. They overexpressed high amounts of active PMCA pump (15-20 times over the amount of endogenous PMCA) as indicated by experiments in which the formation of the phosphoenzyme intermediate and the uptake of Ca2+ by microsomes were measured. Immunocytochemistry experiments coupled to the biotinylation of the pump in the intact cells indicated the correct deliver of the expressed pump to the plasma membrane. The expressed pump was purified by affinity chromatography on calmodulin sepharose. The PMCA of transfected CHO cells promoted an increase of Ca2+ into the medium, after induction of Ca2+ release from the internal stores by activation of a purinergic receptor. An evident decrease of the activity of the endogenous sarcoplasmic reticulum Ca(2+)-ATPase pump was observed, probably related to the down-regulation of its expression. The cells overexpressing the PMCA pump had delayed recovery after trypsinization and plating. Their doubling time was, however, the same as CHO cells.

708 ||| 8529199 ||| (4, 0.46),(2, 0.32) ||| 4 ||| 5 ||| Gain and loss of hypersensitivity to resistance modifiers in multidrug resistant Chinese hamster ovary cells. Some, but not all, multidrug resistant cell lines exhibit collateral hypersensitivity to resistance modifiers. We have examined the relationship between levels of P-glycoprotein expression and resistance modifier hypersensitivity in Chinese hamster ovary cell lines. We have defined a model system for the gain and loss of such sensitivity which indicates that its presence is not simply proportional to P-glycoprotein levels, but is acquired only above a certain level of P-glycoprotein expression. We also show that previously reported differences in such sensitivity between lines is not attributable to differences in genetic background of the cell lines used for selection of drug resistance.

709 ||| 9211923 ||| (4, 0.46) ||| 4 ||| 5 ||| Amidophosphoribosyltransferase limits the rate of cell growth-linked de novo purine biosynthesis in the presence of constant capacity of salvage purine biosynthesis. Factors controlling relative flux rates of the de novo and salvage pathways of purine nucleotide biosynthesis during animal cell growth are not fully understood. To examine the relative role of each pathway for cell growth, three cell lines including CHO K1 (a wild-type Chinese hamster ovary fibroblast cell line), CHO ade -A (an auxotrophic cell line deficient of amidophosphoribosyltransferase (ATase), a presumed rate-limiting enzyme of the de novo pathway), and CHO ade -A transfected with human ATase cDNA (-A+hATase) resulting in 30-350% of the ATase activity of CHO K1, were cultured in purine-rich or purine-free media. Based on the enzyme activities of ATase and hypoxanthine phosphoribosyltransferase, the metabolic rate of the de novo and salvage pathways, the rate of cell growth (growth rate) in three cell lines under various culture conditions, and the effect of hypoxanthine infusion on the metabolic rate of the de novo pathway in rat liver, we concluded the following. 1) In -A+hATase transfectants, ATase activity limits the rate of the de novo pathway, which is closely linked with the growth rate. 2) Purine nucleotides are synthesized preferentially by the salvage pathway as long as hypoxanthine, the most essential source of purine salvage, can be utilized, which was confirmed in rat liver in vivo by hypoxanthine infusion. The preferential usage of the salvage pathway results in sparing the energy expenditure required for de novo synthesis. 3) The regulatory capacity of the de novo pathway (about 200%) was larger than that of the salvage pathway (about 20%) with constant hypoxanthine phosphoribosyltransferase activity.

710 ||| 20506311 ||| (4, 0.46) ||| 4 ||| 5 ||| Effect of inducible co-overexpression of protein disulfide isomerase and endoplasmic reticulum oxidoreductase on the specific antibody productivity of recombinant Chinese hamster ovary cells. To enhance specific antibody (Ab) productivity (q(Ab)) of recombinant Chinese hamster ovary (rCHO) cells, post-translational limitations in the endoplasmic reticulum during antibody production should be relieved. Previously, we reported that overexpression of protein disulfide isomerase (PDI), which catalyzes disulfide bond exchanges and assists in protein folding of newly synthesized proteins, enhanced q(Ab) of rCHO cells by about 27% (Mohan et al., 2007, Biotechnol Bioeng 98:611-615) . Since the rate limiting step in disulfide bond formation is found to be the regeneration of oxidized PDI, the oxidation state of PDI, as well as the amount of PDI, might be important. Endoplasmic reticulum oxidoreductase (ERO1L) maintains PDI in an oxidized state so that disulfide bond formation occurs. Here, PDI and its helper protein, ERO1L were overexpressed in rCHO cells producing an Ab in an attempt to ease the bottleneck in disulfide bond formation, and hence, Ab folding and secretion. Transient expression of ERO1L alone and with PDI resulted in enhanced q(Ab) by 37% and 55%, respectively. In contrast, under stable inducible co-overexpression of PDI and ERO1L, the q(Ab) was unaffected or negatively affected by varying degrees, depending on the individual expression levels of these genes. In stable clones with altered oxidation state of PDI due to co-overexpression of PDI and ERO1L, secretion of Ab was hindered and PDI-associated retention of Ab was seen in the cells. Under transient gene expression, secretion of Ab was not compromised. The data presented here suggests a possible mechanism of PDI/ERO1L interaction with the target Ab and shows how the expression levels of these proteins could affect the q(Ab) of this Ab-producing rCHO cell line.

711 ||| 11485549 ||| (4, 0.46) ||| 4 ||| 5 ||| Steady-state localization of a medial-Golgi glycosyltransferase involves transit through the trans-Golgi network. The steady-state localization of medial-Golgi enzymes is likely to involve retrograde transport pathways; however, the trafficking of these resident enzymes through the Golgi stack is unclear. To investigate if the medial-Golgi enzyme beta-1,2-N-acetylglucosaminyltransferase I (GlcNAc-TI) is transported to the late Golgi, a modified GlcNAc-TI bearing an N-glycan site on the C-terminus was constructed. The modified GlcNAc-TI was demonstrated to be functionally active in vivo, and was localized to the Golgi stack of transfected cells. In stable Chinese-hamster ovary (CHO) cell clones, the N-glycosylated GlcNAc-TI carried sialylated complex N-glycan chains. Pulse-chase studies showed that the majority of GlcNAc-TI was sialylated within 60 min of synthesis. Treatment of transfected CHO cells with Brefeldin A resulted in the glycosylated GlcNAc-TI bearing endo-beta-N-acetylglucosaminidase H resistant chains; however, the sialylation of glycosylated GlcNAc-TI was dramatically reduced. These data imply that, in CHO cells, newly synthesized GlcNAc-TI is transported rapidly through the Golgi stack to the trans-Golgi network, suggesting that GlcNAc-TI continuously recycles from the late Golgi. Furthermore, this data suggests that retrograde transport pathways play an important role in establishing the asymmetric distribution of GlcNAc-TI within the Golgi stack.

712 ||| 10984471 ||| (4, 0.46) ||| 4 ||| 5 ||| Endoplasmic reticulum (ER)-associated degradation of misfolded N-linked glycoproteins is suppressed upon inhibition of ER mannosidase I. To examine the role of early carbohydrate recognition/trimming reactions in targeting endoplasmic reticulum (ER)-retained, misfolded glycoproteins for ER-associated degradation (ERAD), we have stably expressed the cog thyroglobulin (Tg) mutant cDNA in Chinese hamster ovary cells. We found that inhibitors of ER mannosidase I (but not other glycosidases) acutely suppressed Cog Tg degradation and also perturbed the ERAD process for Tg reduced with dithiothreitol as well as for gamma-carboxylation-deficient protein C expressed in warfarin-treated baby hamster kidney cells. Kifunensine inhibition of ER mannosidase I also suppressed ERAD in castanospermine-treated cells; thus, suppression of ERAD does not require lectin-like binding of ER chaperones calnexin and calreticulin to monoglucosylated oligosaccharides. Notably, the undegraded protein fraction remained completely microsome-associated. In pulse-chase studies, kifunensine-sensitive degradation was still inhibitable even 1 h after Tg synthesis. Intriguingly, chronic treatment with kifunensine caused a 3-fold accumulation of Cog Tg in Chinese hamster ovary cells and did not lead to significant induction of the ER unfolded protein response. We hypothesize that, in a manner not requiring lectin-like activity of calnexin/calreticulin, the recognition or processing of a specific branched N-linked mannose structure enhances the efficiency of glycoprotein retrotranslocation from the ER lumen.

713 ||| 7654210 ||| (4, 0.45),(2, 0.39) ||| 4 ||| 5 ||| Evidence for an ethanolamine cycle: differential recycling of the ethanolamine moiety of phosphatidylethanolamine derived from phosphatidylserine and ethanolamine. Evidence is presented for the operation of an ethanolamine-phosphatidylethanolamine (PtdEtn) cycle in Chinese hamster ovary cells. PtdEtn was labelled with [3H]ethanolamine and radioactivity was chased by incubation with 1 mM unlabelled ethanolamine. Radioactivity in [3H]PtdEtn gradually declined over a 23 h time period. In contrast, when the cells were incubated in medium lacking unlabelled ethanolamine, radioactivity in PtdEtn remained constant for at least 23 h. These observations suggest that the ethanolamine moiety is continuously released from PtdEtn and recycled back into PtdEtn. In cells incubated without unlabelled ethanolamine, labelled ethanolamine released from PtdEtn is re-incorporated into PtdEtn without significant dilution. In contrast, in cells incubated with unlabelled ethanolamine the specific radioactivity of the intracellular ethanolamine pool decreases as a result of dilution by the exogenous ethanolamine, hence radioactivity in PtdEtn gradually declines. Similar results were obtained for confluent and non-confluent cells. Our data also demonstrate that when PtdEtn is derived from phosphatidylserine decarboxylation, the ethanolamine cycle operates only in actively dividing, and not in confluent, cells, implying that PtdEtn derived from different biosynthetic origins [i.e. from decarboxylation of phosphatidylserine or from ethanolamine (most likely via the CDP-ethanolamine pathway)] is metabolized differently.

714 ||| 8589689 ||| (4, 0.45) ||| 4 ||| 5 ||| Gene amplification of the Menkes (MNK; ATP7A) P-type ATPase gene of CHO cells is associated with copper resistance and enhanced copper efflux. Three copper-resistant variants of cultured Chinese hamster ovary (CHO) cells were isolated and each was shown to accumulate less intracellular copper than the parental cells when grown in copper-supplemented media. The reduced copper accumulation was related to enhanced copper efflux. As cultured cells from patients with Menkes disease (mutations in MNK; ATP7A gene) accumulate copper, probably due to defective copper efflux, we investigated the possible role of the MNK gene in the molecular basis of copper resistance. We found increased MNK mRNA and MNK protein in all three resistant variants. The MNK protein, which has not been previously demonstrated experimentally in mammalian cells, was observed to have an apparent molecular weight of 178 kDa on SDS gels. The degree of increase in MNK mRNA and protein correlated well with the level of copper resistance and extent of copper efflux. By Southern blot and FISH analysis we determined that the molecular basis for overexpression of MNK was genomic amplification of the MNK gene. These data, combined with the clinical and cellular phenotype in Menkes disease, provide strong evidence that the MNK protein is involved in transmembrane copper efflux, and demonstrate a new system of gene amplification in mammalian cells.

715 ||| 20884265 ||| (4, 0.45) ||| 4 ||| 5 ||| Molecular characterization of the AdeI mutant of Chinese hamster ovary cells: a cellular model of adenylosuccinate lyase deficiency. Adenylosuccinate lyase (ADSL, E. C. 4.3.2.2) carries out two non-sequential steps in de novo AMP synthesis, the conversion of succinylaminoimidazole carboxamide ribotide (SAICAR) to aminoimidazolecarboxamide ribotide (AICAR) and the conversion of succinyl AMP (AMPS) to AMP. In humans, mutations in ADSL lead to an inborn error of metabolism originally characterized by developmental delay, often with autistic features. There is no effective treatment for ADSL deficiency. Hypotheses regarding the pathogenesis include toxicity of high levels of SAICAR, AMPS, or their metabolites, deficiency of the de novo purine biosynthetic pathway, or lack of a completely functional purine cycle in muscle and brain. One important approach to understand ADSL deficiency is to develop cell culture models that allow investigation of the properties of ADSL mutants and the consequences of ADSL deficiency at the cellular level. We previously reported the isolation and initial characterization of mutants of Chinese hamster ovary (CHO-K1) cells (AdeI) that lack detectable ADSL activity, accumulate SAICAR and AMPS, and require adenine for growth. Here we report the cDNA sequences of ADSL from CHO-K1 and AdeI cells and describe a mutation resulting in an alanine to valine amino acid substitution at position 291 (A291V) in AdeI ADSL. This substitution lies in the "signature sequence" of ADSL, inactivates the enzyme, and validates AdeI as a cellular model of ADSL deficiency.

716 ||| 18708345 ||| (4, 0.44),(3, 0.43) ||| 4 ||| 5 ||| Inhibition of heparan sulfate and chondroitin sulfate proteoglycan biosynthesis. Proteoglycans (PGs) are composed of a protein moiety and a complex glycosaminoglycan (GAG) polysaccharide moiety. GAG chains are responsible for various biological activities. GAG chains are covalently attached to serine residues of the core protein. The first step in PG biosynthesis is xylosylation of certain serine residues of the core protein. A specific linker tetrasaccharide is then assembled and serves as an acceptor for elongation of GAG chains. If the production of endogenous GAG chains is selectively inhibited, one could determine the role of these endogenous molecules in physiological and developmental functions in a spatiotemporal manner. Biosynthesis of PGs is often blocked with the aid of nonspecific agents such as chlorate, a bleaching agent, and brefeldin A, a fungal metabolite, to elucidate the biological roles of GAG chains. Unfortunately, these agents are highly lethal to model organisms. Xylosides are known to prime GAG chains. Therefore, we hypothesized that modified xylose analogs may able to inhibit the biosynthesis of PGs. To test this, we synthesized a library of novel 4-deoxy-4-fluoroxylosides with various aglycones using click chemistry and examined each for its ability to inhibit heparan sulfate and chondroitin sulfate using Chinese hamster ovary cells as a model cellular system.

717 ||| 8910379 ||| (4, 0.44) ||| 4 ||| 5 ||| Glycosylation defect in Lec1 Chinese hamster ovary mutant is due to a point mutation in N-acetylglucosaminyltransferase I gene. The Lec1 Chinese hamster ovary (CHO) mutant is a leuco-phytohemagglutinin resistant cell line unable to synthesize complex and hybrid N-glycans due to the lack of N-acetylglucosaminyltransferase I (GnTI) activity. Here we have identified the lec1 mutation. Using specific antibodies to GnTI we demonstrate that Lec1 cells synthesize an inactive GnTI protein identical in size to the wild-type CHO enzyme. We have cloned and sequenced the gene coding GnTI from parental CHO and Lec1 mutant cells. Comparison of GnTI sequences detected three mutations within the luminal domain of Lec1 GnTI, each resulting in an amino acid substitution. The effect of each mutation on enzyme activity was analyzed by site-directed mutagenesis of wild-type rabbit GnTI and transient expression in COS cells. One of the three mutations (Cys123 --> Arg123) resulted in complete loss of activity, whereas the other two mutations had no apparent effect on enzyme activity. This conclusion was confirmed by expression of GnTI mutants in the GnTI null background of Saccharomyces cerevisiae. Both Lec1 GnTI and the GnTI mutant (Cys123 --> Arg123) are correctly localized to the Golgi apparatus, indicating that the inactive GnTI molecules are sufficiently well folded for efficient transport from the endoplasmic reticulum. These results demonstrate that the lec1 mutation is a point mutation and that Cys123 is a critical residue for GnTI activity.

718 ||| 17189266 ||| (4, 0.44) ||| 4 ||| 5 ||| Biosynthesis of chondroitin and heparan sulfate in chinese hamster ovary cells depends on xylosyltransferase II. Xylosyltransferase (XylT) catalyzes the initial enzymatic reaction in the glycosaminoglycan assembly pathway for proteoglycan biosynthesis. Its activity is thought to be rate-limiting. Two xylosyltransferases have been found using genomic analyses, and one of these, XylT1, has been shown to have xylosyltransferase activity. On the other hand, the less studied XylT2 in recombinant form lacks xylosyltransferase activity and has no known function. Wild-type Chinese hamster ovary cells express abundant Xylt2 mRNA levels and lack detectable Xylt1 mRNA levels. Analysis of a previously described Chinese hamster ovary cell xylosyltransferase mutant (psgA-745) shows that it harbors an Xylt2 nonsense mutation and fails to assemble glycosaminoglycans onto recombinant biglycan. Transfection of this cell line with a murine Xylt2 minigene results in the production of recombinant chondroitin sulfate-modified biglycan core protein and restoration of fibroblast growth factor binding to cell surface-associated heparan sulfate. Expression analyses on 10 different human transformed cell lines detect exclusive XYLT2 expression in two and co-expression of XYLT1 and XYLT2 in the others but at disparate ratios where XYLT2 expression is greater than XYLT1 in most cell lines. These results indicate that XylT2 has a significant role in proteoglycan biosynthesis and that cell type may control which family member is utilized.

719 ||| 8530468 ||| (4, 0.43),(3, 0.32) ||| 4 ||| 5 ||| Lec32 is a new mutation in Chinese hamster ovary cells that essentially abrogates CMP-N-acetylneuraminic acid synthetase activity. LEC29.Lec32 is a glycosylation mutant that was isolated from a selection of mutagenized Chinese hamster ovary (CHO) cells for lectin resistance. Compared with LEC29 CHO cells, the double mutant exhibited an unusually high sensitivity to the toxic lectin, ricin, indicating increased exposure of galactose residues on cell surface carbohydrates. Structural analysis of LEC29.Lec32 cellular glycoproteins showed a nearly complete lack of sialic acid residues. Genetic analysis demonstrated that the lec32 mutation is recessive and novel. Biochemical analysis showed that the mutant cells contained less than 5% of the cytidine 5'-monophosphate N-acetylneuraminic acid (CMP-NeuAc) present in parental CHO cells (1.6 nmol/mg of cell protein). A sensitive radiochemical assay used to measure CMP-NeuAc synthetase activity showed that the properties of this enzyme in parental CHO cells were essentially identical to those of CMP-NeuAc synthetase in various mammalian tissues. However, no CMP-NeuAc synthetase activity was detected in LEC29.Lec32 extracts. Mixing experiments provided no evidence for an inhibitor in the mutant CHO cells, and two revertants, which expressed only the LEC29 phenotype, had normal CMP-NeuAc synthetase levels. The combined evidence indicates that the lec32 mutation resides in either the structural gene encoding CMP-NeuAc synthetase or in a gene that regulates the production of active enzyme.

720 ||| 10025667 ||| (4, 0.42),(5, 0.40) ||| 4 ||| 5 ||| Chinese hamster ovary cells with reduced hexokinase activity maintain normal GDP-mannose levels. Parental Chinese hamster ovary (CHO) cells were mutagenized and subjected first to a mannose suicide selection technique and second to a screen of individual colonies grown on polyester discs for reduced mannose incorporation into protein. The incorporation of radioactivity for the selection and the screen was conducted at 41.5 degrees C instead of the normal growth temperature of 34 degrees C in order to allow for the isolation of temperature-sensitive lesions. This selection/screening procedure resulted in the isolation of M15-4 cells, which had three- to five-fold lower incorporation of [2-3H]mannose into mannose 6-phosphate, mannose 1-phosphate, GDP-mannose, oligosaccharide-lipid, and glycoprotein at 41.5 degrees C. We detected no difference in the qualitative pattern of mannose-labeled lipid-linked oligosaccharides compared to parental cells. M15-4 cells synthesized dolichol. The defect of M15-4 cells was determined to be in hexokinase activity; crude cytosolic extracts were eight- to nine-fold lower in hexokinase activity in M15-4 cells compared to parental cells. As a result of this defect, incorporation of labeled mannose from the medium was significantly decreased. However, the level of GDP-mannose in M15-4 cells was 70% of normal. The phenotype of M15-4 was a lower specific activity of labeled GDP-mannose, not a substantial reduction in the level of GDP-mannose. Consistent with these results, no alterations in the glycosylation of a model glycoprotein, G protein of vesicular stomatitis virus, were observed. These cells grew slower than parental cells, especially in low-glucose medium.

721 ||| 8792530 ||| (4, 0.41),(7, 0.35) ||| 4 ||| 5 ||| Cytotoxicity and mutagenicity of 6-aminonicotinamide in Chinese hamster ovary cells. A strong teratogen-6-aminonicotinamide (6-AN)-was tested for its ability to induce cytotoxicity and mutagenicity in Chinese hamster ovary (CHO) cells. Tests were performed in the presence and absence of a metabolic activation system (S-9 mix). Cytotoxicity was evaluated in CHO cells by the total protein content. The two single-gene mutation systems in CHO cells have been investigated. Both involve evaluating the response of the hypoxanthine-guanine phosphoribosyl transferase (HGPRT) locus and specific inhibitors of the cellular (Na2+K+)-ATPase using 6-thioguanine and ouabain as selective agents, respectively. From our results, 6-AN showed a higher cytotoxic effect at concentrations over 1 x 10(-1) mg/ml. Cytotoxicity was significantly different with and without S-9 mix. 6-AN was cytotoxic per se, however, when 6-AN was biotransformed, in the presence of S-9 mix no biological activity (cytotoxic) was detected. Non-significant mutagenic activity was detected with 6-AN in the presence and in the absence of the metabolic activation system.

722 ||| 10477274 ||| (4, 0.41),(5, 0.34) ||| 4 ||| 5 ||| GA2/GM2/GD2 synthase localizes to the trans-golgi network of CHO-K1 cells. UDP-GalNAc:lactosylceramide/GM3/GD3 beta-1,4-N-acetylgalactosaminyltransferase (GalNAc-T) transforms its acceptors into the gangliosides GA2, GM2 and GD2. It is well established that it is a Golgi-located glycosyltransferase, but its sub-Golgi localization is still unclear. We addressed this question in Chinese hamster ovary K1 cell clones stably transfected with a c-myc-tagged version of GalNAc-T which express the enzyme at different levels of activity. In these cell clones we examined the effect of brefeldin A (BFA) on the synthesis of glycolipids (in metabolic-labelling experiments) and on the sub-Golgi localization of the GalNAc-T (by immunocytochemistry). We found that in cell clones expressing moderate levels of activity, GalNAc-T immunoreactivity behaved as the trans-Golgi network (TGN) marker mannose-6-P receptor (M6PR) both in BFA-treated and untreated cells, and that BFA completely blocked the synthesis of GM2, GM1 and GD1a. On the other hand, in cell clones expressing high levels of activity and treated with BFA, most GalNAc-T immunoreactivity redistributed to the endoplasmic reticulum, as did the medial-Golgi marker mannosidase II, and the synthesis of GM2, GM1 and GD1a was not completely blocked. These results indicate that GalNAc-T is a TGN-located enzyme and that the mechanism that localizes it to this compartment involves steps that, when saturated, lead to its mislocalization to the cis-, medial- or trans-Golgi. Changes of Golgi membrane properties by modification of local glycolipid composition due to the activity of the expressed enzyme were not the main cause of mislocalization, since it persists when glycolipid synthesis is inhibited with d, l-threo-1-phenyl-2-hexadecanoylamino-3-pyrrolidino-1-propanol-HCl.

723 ||| 8782489 ||| (4, 0.41) ||| 4 ||| 5 ||| New mutations and phenotypes associated with glutamate and aspartate transport in Chinese hamster ovary (CHO-K1) cells. Two new Chinese hamster ovary cell (CHO-K1) mutants lacking amino acid transport System X-AG activity were isolated by [3H]aspartate suicide selection. These null mutants, Dd-B6 and Dd-B7, were analyzed by somatic cell hybridization, along with previously described partial-function mutants, Ed-A1 and Ed-B8. With respect to System X-AG activity, all four mutations fell into a single complementation group. By quantitative assay, the mutations in Ed-A1 and Ed-B8 behaved as simple recessives in fusions with wild type cells, while those in Dd-B6 and Dd-B7 were codominant. We have discovered that Ed-A1 and Ed-B8 are highly permeable to small neutral molecules. This high permeability phenotype was dominant to wild-type. Northern, Southern, and Western analyses indicated that System X-AG in CHO is not closely related to any of the three well characterized glutamate transporters represented by GLT-1, EAACI or GLAST.

724 ||| 24801859 ||| (4, 0.40),(8, 0.30) ||| 4 ||| 5 ||| An in-silico study of the regulation of CHO cells glycolysis. In this work, a kinetic-metabolic model previously developed for CHO cells is used to study glycolysis regulation. The model is assessed for its biological relevance by analyzing its ability to simulate metabolic events induced following a hypoxic perturbation. Feedback and feedforward regulatory mechanisms known to occur to either inhibit or activate fluxes of glycolysis, are implemented in various combined scenarios and their effects on the metabolic response were analyzed. This study aims at characterizing the role of intermediates of glycolysis and of the cell energetic state, described as the AMP-to-ATP ratio, as inhibitors and activators of glycolysis pathway. In addition to the glycolysis pathway, we here describe the transient metabolic response of pathways that are connected to glycolysis, such as the pentose phosphate pathway, TCA cycle, cell bioenergetics system, glutamine and amino acids metabolisms. Taken individually, each regulatory mechanism leads to an oscillatory behavior in response to a hypoxic perturbation, while their combination clearly damps oscillations. However, only the addition of the cell energetic state to the regulatory mechanisms results in a non-oscillating response leading to metabolic flux rate rearrangement corresponding to the anaerobic metabolism expected to prevail under hypoxic conditions. We thus demonstrate in this work, from model simulations, that the robustness of a cell energetic metabolism can be described from a combination of feedback and feedforward inhibition and activation regulatory mechanisms of glycolysis fluxes, involving intermediates of glycolysis and the cell energetic state itself.

725 ||| 10101035 ||| (4, 0.40),(5, 0.36) ||| 4 ||| 5 ||| Multiple mechanisms of resistance to polyglutamatable and lipophilic antifolates in mammalian cells: role of increased folylpolyglutamylation, expanded folate pools, and intralysosomal drug sequestration. Chinese hamster ovary PyrR100 cells display more than 1000-fold resistance to pyrimethamine (Pyr), a lipophilic antifolate inhibitor of dihydrofolate reductase. PyrR100 cells had wild-type DHFR activity, lost folate exporter activity, and had a 4-fold increased activity of a low pH folic acid transporter. Here we report on the marked alterations identified in PyrR100 cells compared with parental cells: 1) approximately 100-fold decreased folic acid growth requirement; 2) a 25-fold higher glucose growth requirement in Pyr-containing medium; 3) a 2.5- to 4.1-fold increase in folylpolyglutamate synthetase activity; 4) a 3-fold increase in the accumulation of [3H]folic acid and a 3-fold expansion of the intracellular folate pools; 5) a 4-fold increase in the activity of the lysosomal marker beta-hexoseaminidase, suggesting an increased lysosome number/PyrR100 cell; and 6) a small reduction in the steady-state accumulation of [3H]Pyr and no evidence of catabolism or modification of cellular [3H]Pyr. Consequently, PyrR100 cells were markedly resistant to the lipophilic antifolates trimetrexate (40-fold) and AG377 (30-fold) and to the polyglutamatable antifolates 5,10-Dideaza-5,6,7,8-tetrahydrofolic acid (DDATHF) (26-fold) and AG2034 (14-fold). Resistance to these drugs was reversed in PyrR100 cells transferred into folate-depleted medium. In conclusion, these multiple resistance factors collectively result in a prominent increase in folate accumulation, an expansion of the intracellular folylpolyglutamate pool, and abolishment of the cytotoxic activity of polyglutamatable and lipophilic antifolates. The role of increased lysosome number per cell in sequestration of hydrophobic weak base drugs such as Pyr is also discussed as a novel mechanism of drug resistance.

726 ||| 7836441 ||| (4, 0.40) ||| 4 ||| 5 ||| Stable amplification of the S-adenosylmethionine decarboxylase gene in Chinese hamster ovary cells. A Chinese hamster ovary cell subline (CHO/664) > 1000-fold resistant to the S-adenosylmethionine decarboxylase (AdoMetDC) inhibitor, CGP-48664 (4-(aminoiminomethyl)-2,3-dihydro-1H-inden-1-one diaminomethylenehydrazone), has been developed and characterized. The cells were also cross-resistant to the highly specific nucleoside analog inhibitor of AdoMetDC, MDL-73811. These unique cells stably overexpress AdoMetDC due to a 10-16-fold amplification of the AdoMetDC gene, which resulted in a similar increase in AdoMetDC transcript levels. In the presence of 100 microM CGP-48664, the CHO/664 cells displayed AdoMetDC activities similar to the parental line. Following removal of the inhibitor, AdoMetDC activity increased steadily over 20 days to 10-12 times that found in parental CHO cells. Decarboxylated (dc) AdoMet pools accumulated rapidly from < 5 pmol/10(6) cells to approximately 1000-1500 pmol/10(6) cells at 3 days due to diffusion away of intracellular inhibitor and to the depletion of putrescine and spermidine as aminopropyl acceptors in dcAdoMet-mediated synthase reactions. Polyamine pools shifted as putrescine, and spermidine pools were processed forward to spermine. During the period from 3 days to 20 days, dcAdoMet pools fell steadily and eventually stabilized at 100-200 pmol/10(6) cells. Providing excess putrescine at this time as an aminopropyl acceptor rapidly lowered dcAdoMet pools and led to a near normalization of polyamine pools, indicating that both dcAdoMet and putrescine are essential in maintaining steady-state polyamine pool profiles. As with cell line variants that overproduce ornithine decarboxylase, polyamine transport was found to be increased in CHO/664 cells due to an apparent inability of the system to down-regulate polyamine transport in response to polyamine excess. Given the unique metabolic disturbances seen in these cells, we anticipate that in addition to providing a useful system for evaluating the specificity of newly developed AdoMetDC inhibitors, they will undoubtedly prove valuable for investigating the various regulatory interrelationships involved in polyamine homeostasis and possibly other aspects of purine metabolism.

727 ||| 7559470 ||| (4, 0.40) ||| 4 ||| 5 ||| Mutations in the elongation factor 2 gene which confer resistance to diphtheria toxin and Pseudomonas exotoxin A. Genetic and biochemical analyses. Both diphtheria toxin and Pseudomonas exotoxin A inhibit eukaryotic protein synthesis by ADP-ribosylating diphthamide, a posttranslationally modified histidine residue present in the elongation factor 2 (EF-2) protein. Elongation factor 2 cannot be ADP-ribosylated by the toxins unless this histidine is modified. In this report we identify three new point mutations in toxin-resistant alleles of the Chinese hamster ovary cell elongation factor 2 gene. The mutations resulted in amino acid substitutions at positions 584 (serine to glycine), 714 (isoleucine to asparagine), and 719 (glycine to aspartic acid). All three amino acid substitutions prevented the biosynthesis of diphthamide. The amount by which the toxins reduced protein synthesis in each of these mutant cell strains suggested that all three mutations also either impaired the function of EF-2 or reduced its steady state level in the cytoplasm. Western blot analysis showed that equal amounts of EF-2 were present in each of the cell strains, indicating that the mutations impaired the catalytic function of EF-2.

728 ||| 10037775 ||| (4, 0.39),(2, 0.37) ||| 4 ||| 5 ||| Molecular enzymology of mammalian Delta1-pyrroline-5-carboxylate synthase. Alternative splice donor utilization generates isoforms with different sensitivity to ornithine inhibition. Delta1-Pyrroline-5-carboxylate synthase (P5CS; EC not assigned), a mitochondrial inner membrane, ATP- and NADPH-dependent, bifunctional enzyme, catalyzes the reduction of glutamate to Delta1-pyrroline-5-carboxylate, a critical step in the de novo biosynthesis of proline and ornithine. We utilized published plant P5CS sequence to search the expressed sequence tag data base and cloned two full-length human P5CS cDNAs differing in length by 6 base pairs (bp) in the open reading frame. The short cDNA has a 2379-bp open reading frame encoding a protein of 793 residues; the long cDNA, generated by "exon sliding," a form of alternative splicing, contains an additional 6-bp insert following bp +711 of the short form resulting in inclusion of two additional amino acids in the region predicted to be the gamma-glutamyl kinase active site of P5CS. The long form predominates in all tissues examined except gut. We also isolated the corresponding long and short murine P5CS transcripts. To confirm the identity of the putative P5CS cDNAs, we expressed both human forms in gamma-glutamyl kinase- and gamma-glutamyl phosphate reductase-deficient strains of Saccharomyces cerevisiae and showed that they conferred the proline prototrophy. Additionally, we found expression of the murine putative P5CS cDNAs conferred proline prototrophy to P5CS-deficient Chinese hamster ovary cells (CHO-K1). We utilized stable CHO-K1 cell transformants to compare the biochemical characteristics of the long and short murine P5CS isoforms. We found that both confer P5CS activity and that the short isoform is inhibited by L-ornithine with a Ki of approximately 0.25 mM. Surprisingly, the long isoform is insensitive to ornithine inhibition. Thus, the two amino acid insert in the long isoform abolishes feedback inhibition of P5CS activity by L-ornithine.

729 ||| 9442042 ||| (4, 0.39) ||| 4 ||| 5 ||| Mechanism of acidification of the trans-Golgi network (TGN). In situ measurements of pH using retrieval of TGN38 and furin from the cell surface. Sorting of secretory cargo and retrieval of components of the biosynthetic pathway occur at the trans-Golgi network (TGN). The pH within the TGN is thought to be an important determinant of these functions. However, studies of the magnitude and regulation of the pH of the TGN have been hampered by the lack of appropriate detection methods. This report describes a noninvasive strategy to measure the luminal pH of the TGN in intact cells. We took advantage of endogenous cellular mechanisms for the specific retrieval of TGN resident proteins, such as TGN38 and furin, that transit briefly to the plasma membrane. Cells were transfected with chimeric constructs that contained the internalization and retrieval signals of TGN resident proteins, and a luminal (extracellular) epitope (CD25). Like TGN38 and furin, the chimeras were shown by fluorescence microscopy to accumulate within the TGN. During their transient exposure at the cell surface, the chimeras were labeled with extracellular anti-CD25 antibodies conjugated with a pH-sensitive fluorophore. Subsequent endocytosis and retrograde transport resulted in preferential labeling of the TGN with the pH-sensitive probe. Continuous, quantitative measurements of the pH of the TGN were obtained by ratio fluorescence imaging. The resting pH, calibrated using either ionophores or the "null point" technique, averaged 5.95 in Chinese hamster ovary cells and 5.91 in HeLa cells. The acidification was dissipated upon addition of concanamycin, a selective blocker of vacuolar-type ATPases. The counterion conductance was found to be much greater than the rate of H+ pumping at the steady state, suggesting that the acidification is not limited by an electrogenic potential. Both Cl- and K+ were found to contribute to the overall counterion permeability of the TGN. No evidence was found for the presence of active Na+/H+ or Ca2+/H+ exchangers on the TGN membrane. In conclusion, selective retrieval of recombinant proteins can be exploited to target ion-sensitive fluorescent probes to specific organelles. The technique provides real-time, noninvasive, and quantitative determinations of the pH, allowing the study of pH regulation within the TGN in intact cells. The acidic pH of the TGN reflects active H+ pumping into an organelle with a low intrinsic H+ permeability and a high conductance to monovalent ions.

730 ||| 21055620 ||| (4, 0.39) ||| 4 ||| 5 ||| Radioiodinated 4-iodo-L-meta-tyrosine, a system L selective artificial amino acid: molecular design and transport characterization in Chinese hamster ovary cells (CHO-K1 cells). INTRODUCTION: High expression of the system L amino acid transporter has been observed in clinically important tissues including tumors and the blood-brain barrier. We examined amino acid transport system L selectivity of (14)C(U)-L-tyrosine ((14)C-Tyr), (125)I-4-iodo-L-meta-tyrosine (4-(125)I-mTyr), (125)I-6-iodo-L-meta-tyrosine (6-(125)I-mTyr), (125)I-3-iodo-alpha-methyl-L-tyrosine ((125)I-IMT) and (125)I-3-iodo-L-tyrosine (3-(125)I-Tyr) using Chinese hamster ovary cells (CHO-K1). METHODS: Cells in the exponential growth phase were incubated with 18.5 kBq of labeled amino acid in 2 mL of phosphate-buffered saline-based uptake solution and an uptake solution with/without Na(+) at 37 C or 4 C. We examined the effects of the following compounds (1.0 mM) on transport: 2-(methylamino)isobutyric acid (a specific inhibitor of system A, in Na(+)-containing uptake solution); 2-amino-bicyclo[2,2,1]heptane-2-carboxylic acid (a specific inhibitor of system L, in Na(+)-free uptake solution); sodium azide and 2,4-dinitrophenol (NaN(3) and DNP, inhibitors of the generation of adenosine triphosphate); p-aminohippurate and tetraethylammonium (PAH and TEA, inhibitors of organic anion and cation transporters); and L- and D-isomers of natural amino acids. RESULTS: (14)C-Tyr exhibited affinity for systems L, A and ASC. 4-(125)I-mTyr and 3-(125)I-Tyr exhibited high specificity for system L, whereas 6-(125)I-mTyr and (125)I-IMT exhibited affinity for both systems L and ASC. Uptake of 4-(125)I-mTyr was markedly reduced by incubation at 4 C, and was not significantly inhibited by NaN(3), DNP, PAH or TEA. The inhibition profiles of the L- and D-isomers of natural amino acids indicated that system L mediates the transport of 4-(125)I-mTyr. CONCLUSIONS: 4-(125)I-mTyr exhibited the greatest system L specificity (93.46 +- 0.13%) of all of the tested amino acids.

731 ||| 11319223 ||| (4, 0.38),(2, 0.34) ||| 4 ||| 5 ||| Point mutations identified in Lec8 Chinese hamster ovary glycosylation mutants that inactivate both the UDP-galactose and CMP-sialic acid transporters. Nucleotide-sugar transporters (NSTs) are critical components of glycosylation pathways in eukaryotes. The identification of structural elements that are involved in NST functions provides an important task. Chinese hamster ovary glycosylation mutants defective in nucleotide-sugar transport provide access to inactive transporters that can define such structure/function relationships. In this study, we have cloned the hamster UDP-galactose transporter (UGT) and identified defects in UGT gene transcripts from nine independent Chinese hamster ovary mutants that belong to the Lec8 complementation group. Reverse transcription polymerase chain reaction with primers that span the UGT open reading frame showed that three Lec8 mutants express a full-length open reading frame, while six Lec8 mutants predominantly express truncated UGT gene transcripts. Sequencing identified different single or triplet nucleotide changes in full-length UGT transcripts from three of the mutants. These mutations translate into three different amino acid changes at positions that are highly conserved in all the known mammalian NSTs. Transfection of a cDNA encoding either of the mutations Delta serine 213 or G281D failed to correct the UDP-galactose transport defect in Lec8 transfectants. Most importantly, introducing these same mutations into the homologous region of the murine CMP-sialic acid transporter caused inactivation of this transporter. Thus, identifying point mutations that inactivate UGT in Lec8 mutants resulted in the discovery of amino acids that are critical to the activity of both UGT and CST, the two most divergent mammalian NSTs.

732 ||| 9568280 ||| (4, 0.38),(2, 0.33) ||| 4 ||| 5 ||| Branched-chain and unsaturated 1,7-diaminoheptane derivatives as deoxyhypusine synthase inhibitors. Deoxyhypusine synthase catalyzes the first step in the posttranslational biosynthesis of the unusual amino acid hypusine [N epsilon-(4-amino-2-hydroxybutyl)lysine] in eukaryotic translation initiation factor 5A (eIF-5A). eIF-5A and its single hypusine residue are essential for cell proliferation. Two series of 1,7-diaminoheptane derivatives were prepared and tested as inhibitors of human deoxyhypusine synthase. These include branched-chain saturated derivatives and both branched- and straight-chain unsaturated derivatives providing size and positional variation in branching and different torsional constraints. Of the branched-chain compounds, 7-amino-1-guanidinooctane (39) proved to be the most potent inhibitor in vitro (IC50, 34 nM), while 1,7-diamino-trans-hept-3-ene (20a) displayed the greatest inhibition (IC50, 0.7 microM) among the unsaturated compounds. Compound 39 also provided effective inhibition of hypusine production in Chinese hamster ovary cells in culture. Considerations of the in vitro inhibition data reported here, along with earlier findings, allowed some speculation concerning the conformation of the substrate spermidine during its productive interaction at the active site of deoxyhypusine synthase.

733 ||| 8908193 ||| (4, 0.38) ||| 4 ||| 5 ||| Arsenite induces apoptosis in Chinese hamster ovary cells by generation of reactive oxygen species. Arsenic, a human carcinogen, possesses a serious environmental threat but the mechanism of its toxicity remains unclear. Knowledge of how arsenic induces cell death and how cells escape the death path may help to understand arsenic carcinogenesis. We have investigated the nature of sodium arsenite-induced cell death in Chinese hamster ovary K1 cells. Following phosphate-citric acid buffer extraction, apoptotic cells with lower DNA content than the G1 cells were detected by flow cytometry. Immediately after 4 h of 40 microM arsenite treatment, no appreciable fraction of cells with sub-G1 DNA content was detected; however, the sub-G1 cell fraction increased with postarsenite incubation time, and detectable increase started at 8 h of incubation, whereas the intracellular peroxide level as measured by the fluorescent intensity of 2',7'-dichlorofluorescein increased immediately following a 4-h arsenite treatment. Simultaneous treatment with arsenite plus antioxidant (N-acetyl-cysteine, Trolox, and Tempo); copper ion chelator (neocuproine); protein kinase inhibitor (H-7) or protein synthesis inhibitor (cycloheximide) reduced the fraction of sub-G1 cell and internucleosomal DNA degradation. Trolox, neocuproine, or cycloheximide given after arsenite treatment also effectively reduced apoptosis. These results lead to a working hypothesis that arsenite-induced apoptosis in CHO-K1 cells is triggered by the generation of hydrogen peroxide, followed by a copper-mediated Fenton reaction that catalyzes the production of hydroxyl radicals, which selectively activates protein kinase through de novo synthesis of macromolecules.

734 ||| 24468831 ||| (4, 0.37) ||| 4 ||| 5 ||| Modulation of heparan sulfate biosynthesis by sodium butyrate in recombinant CHO cells. Sodium butyrate, a histone deacetylase inhibitor, has been used to improve transgene expression in Chinese hamster ovary (CHO) cells. The current study explores the impact of butyrate treatment on heparan sulfate (HS) biosynthesis and structural composition in a recombinant CHO-S cell line expressing enzymes in the heparin (HP)/(HS) biosynthetic pathway (Dual-10 stably expressing NDST2 and HS3st1). Flow cytometric analysis showed that antithrombin binding was increased in Dual-10 cells and basic fibroblast growth factor binding was decreased in response to sodium butyrate treatment. The results were in agreement with the AMAC-LCMS (2-aminoacridine-tagged HS/HP analysis by liquid chromatography mass spectrometry) data that showed that there was an increase in heparan sulfate tri-sulfated disaccharides and a decrease in N-sulfated disaccharides in the butyrate-treated cells. However, we could not detect any changes in the chondroitin sulfate pathway in Dual-10 cells treated with butyrate. The current study is the first to report the effect of butyrate on glycosaminoglycan profiles.

735 ||| 21585340 ||| (4, 0.37) ||| 4 ||| 5 ||| Demonstration that endoplasmic reticulum-associated degradation of glycoproteins can occur downstream of processing by endomannosidase. During quality control in the ER (endoplasmic reticulum), nascent glycoproteins are deglucosylated by ER glucosidases I and II. In the post-ER compartments, glycoprotein endo-alpha-mannosidase provides an alternative route for deglucosylation. Previous evidence suggests that endomannosidase non-selectively deglucosylates glycoproteins that escape quality control in the ER, facilitating secretion of aberrantly folded as well as normal glycoproteins. In the present study, we employed FOS (free oligosaccharides) released from degrading glycoproteins as biomarkers of ERAD (ER-associated degradation), allowing us to gain a global rather than single protein-centred view of ERAD. Glucosidase inhibition was used to discriminate between glucosidase- and endomannosidase-mediated ERAD pathways. Endomannosidase expression was manipulated in CHO (Chinese-hamster ovary)-K1 cells, naturally lacking a functional version of the enzyme, and HEK (human embryonic kidney)-293T cells. Endomannosidase was shown to decrease the levels of total FOS, suggesting decreased rates of ERAD. However, following pharmacological inhibition of ER glucosidases I and II, endomannosidase expression resulted in a partial switch between glucosylated FOS, released from ER-confined glycoproteins, to deglucosylated FOS, released from endomannosidase-processed glycoproteins transported from the Golgi/ERGIC (ER/Golgi intermediate compartment) to the ER. Using this approach, we have identified a previously unknown pathway of glycoprotein flow, undetectable by the commonly employed methods, in which secretory cargo is targeted back to the ER after being processed by endomannosidase.

736 ||| 9753618 ||| (4, 0.35),(3, 0.31) ||| 4 ||| 5 ||| A functional link between N-linked glycosylation and apoptosis in Chinese hamster ovary cells. Seven different Chinese hamster ovary (CHO) cell mutants, isolated in different ways and having biochemical defects that were expressed at 34 degrees C, were found to be temperature sensitive for growth at 40.5 degrees C. Six of the mutants had five different lesions in N-linked glycosylation; two mutants were in the same complementation group. The temperature-sensitive phenotype in three mutants appeared by cell fusion studies to be linked to the glycosylation phenotype. In some of the glycosylation mutants [B4-2-1 (Lec15.1), Lec9, Lec1, and Lec24], but not in all of them (MI5-4 and MI8-5), incubation at 40.5 degrees C induced apoptosis, as determined by appearance of DNA fragmentation. Tunicamycin (TM) also induced apoptosis in both parental and Lec9 cells. There was a direct correlation between inhibition of glycosylation by TM treatment and induction of apoptosis. Induction of apoptosis by TM was inhibited by cycloheximide. These studies suggest that specific alterations in N-linked glycosylation in CHO cells are endogenous inducers of apoptosis.

737 ||| 9396632 ||| (4, 0.35),(3, 0.31) ||| 4 ||| 5 ||| Chinese hamster ovary cells lacking GM1 and GD1a synthesize gangliosides upon transfection with human GM2 synthase. GM3-positive Chinese hamster ovary cells (CHO-K1 cells) lack the ability to synthesize GM2 and the complex gangliosides GM1 and GD1a from [3H]Gal added to the culture medium. However, they acquire the ability to synthesize GM2 and to synthesize and immunoexpress complex gangliosides upon transient transfection with a cDNA encoding the human GM3:N-acetylgalactosaminyl transferase (GM2 synthase). The activities of endogenous GM1- and GD1a-synthases in the parental cell line and in cells transfected with the plasmid with or without the GM2 synthase cDNA were essentially identical and comparable in terms of specific activity with the endogenous GM3 synthase. Results indicate that glycosyltransferases acting on GM2 to produce GM1 and GD1a are constitutively present in CHO-K1 cells, and that the expression of their activities depend on the supply of the acceptor GM2. In addition, these results lend support to the notion that GM2 synthase is a key regulatory enzyme influencing the balance between simple and complex gangliosides.

738 ||| 9562246 ||| (4, 0.34),(2, 0.33) ||| 4 ||| 5 ||| Characterization of multiple transcripts of the hamster dolichol-P-dependent N-acetylglucosamine-1-P transferase suggests functionally complex expression. The evolutionarily conserved dolichol-P-dependent N-acetylglucosamine-1-P transferase gene, ALG7, functions by initiating the dolichol pathway of protein N-glycosylation. In yeast, ALG7 has a complex expression pattern and plays a critical role in diverse cellular functions, including proliferation and morphological response. In Chinese hamster ovary cells (CHO), ALG7 gives rise to three mRNAs of 1.5, 1.9 and 2.2 kb. We report results of RNA blotting assays, ribonuclease protection, PCR-amplification and sequencing of the CHO ALG7 transcripts 5' and 3' ends which suggest that the 1.5 and 1.9 kb transcripts are produced as a consequence of initiation at 2 distinct start sites, 350-379 bp apart. The transcriptional start site for the 1.5 kb mRNA is positioned between the first two in frame ATGs, while that of the 1.9 kb species is located upstream of these two in-frame ATGs. In order to test the translational competence of the 1.5 and 1.9 kb mRNAs, we constructed DNA templates specifying these transcripts and used them for in vitro transcription/translation. Our data show that the 1.9 kb mRNA served in the synthesis of 36 and 24 kDa species, as well as a low-abundance 32 kDa protein. The 1.5 kb transcript gave rise to a translation product of 32 kDa. The latter is synthesized in CHO cells and hamster submandibular glands. These results suggest the possibility that the 1.5 and 1.9 kb transcripts give rise to related protein isoforms with different lengths of their NH2-terminal regions.

739 ||| 9570071 ||| (4, 0.32) ||| 4 ||| 5 ||| Behavior of CHO cells on phosphated cellulose membranes. Phosphate groups (negatively charged chemical groups) were grafted onto the surface of cellulose membranes by a reaction between hydroxyl groups of cellulose and phosphorus pentoxide to observe the effect of phosphate groups on cellular behavior. X-ray photoelectron spectroscopy (XPS) was used to determine phosphorylation. Captive bubble contact angle measurement was used to determine surface wettability. XPS was also used to analyze serum protein adsorption. Chinese hamster ovary (CHO) cells were maintained in Ham's F-12 nutrient mixture with and without fetal calf serum. Total cell area and shape factor were analyzed using image-analyzing software. Serum proteins showed higher adsorption on phosphated cellulose. Cell spreading on phosphated membranes was greater than on the cellulose membrane that served as control. The cell growth rate was faster compared to the control. Large cell aggregates were not found on the phosphated membranes, in contrast to the control membrane. The cells on the control were aggregated regardless of the existence of divalent cations in the medium.

# ----------------------------- TOPIC5 -----------------------------

740 ||| 7813636 ||| (5, 0.99) ||| 5 ||| 0 ||| Ornithine decarboxylase and S-adenosylmethionine decarboxylase expression during the cell cycle of Chinese hamster ovary cells. Cells in mitosis were harvested from exponentially growing Chinese hamster ovary cells by the mitotic detachment technique. Immediately after harvesting, the mitotic cells were seeded in tissue culture flasks and incubated at 37 degrees C in a CO2 incubator. Care was taken not to perturb the progression of cells through the cell cycle. At every hour after seeding for 14 h, cells were collected for analysis of cell cycle distribution, cellular polyamine content, ornithine decarboxylase (ODC) and S-adenosylmethionine decarboxylase (AdoMetDC) activities, and relative mRNA contents. The progression through the cell cycle was monitored by DNA flow cytometry. The putrescine, spermidine, and spermine levels were approximately doubled during the cell cycle: putrescine mainly during late S and G2, spermidine continuously during the entire cell cycle, and spermine mainly during G1 and S. The ODC activity was low in seeded mitotic cells and the enzyme was activated in late G1 and reached a plateau in S phase. A second burst in activity was observed during late S phase and maximal ODC activity was found at the S/G2 transition. The relative ODC mRNA level approximately doubled during the cell cycle and the increase in the relative level mainly took part during mid and late S phase. AdoMetDC activity increased in late G1 and a first maximum was observed during the G1/S transition. A second burst in activity was found in mid S phase. Maximal AdoMetDC activity was found in G2. The relative AdoMetDC mRNA approximately doubled during the cell cycle and the increase in the relative level mainly took place during late G1 and early S phase. Our results indicate that polyamine synthesis was regulated at transcriptional and translational/post-translational levels during the cell cycle of Chinese hamster ovary cells.

741 ||| 10486245 ||| (5, 0.98) ||| 5 ||| 0 ||| Half-lives of ornithine decarboxylase and S-adenosylmethionine decarboxylase activities during the cell cycle of Chinese hamster ovary cells. Cells in mitosis were seeded immediately after being harvested by the mitotic shake off technique from a culture of exponentially growing Chinese hamster ovary cells. At 2, 5, 7, 10, and 12 h after seeding, cycloheximide was added. Cells were sampled at various times after cycloheximide addition and the ornithine decarboxylase (ODC) and S-adenosylmethionine decarboxylase (AdoMetDC) activities were determined. Flow cytometric analysis showed that cells sampled at 2, 5, 7, 10, and 12 h after seeding were found in mid G(1), at the G(1)/S transition, in mid S phase, at the S/G(2) transition, and in late G(2), respectively. The half-lives of ODC and AdoMetDC activities varied during the cell cycle. The half-life of ODC activity showed a biphasic pattern with increases in connection to the G(1)/S and S/G(2) transitions while the half-life of AdoMetDC activity increased only at the G(1)/S transition.

742 ||| 17318909 ||| (5, 0.87) ||| 5 ||| 0 ||| Dependence on glucose limitation of the pCO2 influences on CHO cell growth, metabolism and IgG production. The culture levels of glucose and CO(2) have been reported to independently have important influences on mammalian cell processes. In this work the combined effects of glucose limitation and CO(2) partial pressure (pCO(2)) on monoclonal antibody (IgG) producing Chinese Hamster Ovary cells were investigated in a perfusion reactor operated with controlled cell specific medium feed rate, pH and osmolality. Under high glucose conditions (14.3 +/- 0.8 mM), the apparent growth rate decreased (from 0.021 to 0.009 h(-1)) as the pCO(2) increased to approximately 220 mmHg, while the cell specific IgG productivity was almost unchanged. The lactate yield from glucose was not affected by pCO(2) up to approximately 220 mmHg and glucose was mainly converted to lactate. A feed medium modification from high (33 mM) to low (6 mM) glucose resulted in <0.1 mM glucose in the culture. As a result of apparently shifting metabolism towards the conversion of pyruvate to CO(2), both the ratio of lactate to glucose and the alanine production rate were lowered (1.51-1.14 and 17.7-0.56 nmol/10(6) cells h, respectively). Interestingly, when the pCO(2) was increased to approximately 140 mmHg, limiting glucose resulted in 1.7-fold higher growth rates, compared to high glucose conditions. However, at approximately 220 mmHg pCO(2) this beneficial effect of glucose limitation on these CHO cells was lost as the growth rate dropped dramatically to 0.008 h(-1) and the IgG productivity was lowered by 15% (P < 0.01) relative to the high glucose condition. The IgG galactosylation increased under glucose- limited compared to high-glucose conditions.

743 ||| 10458739 ||| (5, 0.87) ||| 5 ||| 0 ||| Bicarbonate concentration and osmolality are key determinants in the inhibition of CHO cell polysialylation under elevated pCO(2) or pH. Accumulation of CO(2) in animal cell cultures can be a significant problem during scale-up and production of recombinant glycoprotein biopharmaceuticals. By examining the cell-surface polysialic acid (PSA) content, we show that elevated CO(2) partial pressure (pCO(2)) can alter protein glycosylation. PSA is a high-molecular-weight polymer attached to several complex N-linked oligosaccharides on the neural cell adhesion molecule (NCAM), so that small changes in either core glycosylation or in polysialylation are amplified and easily measured. Flow-cytometric analysis revealed that PSA levels on Chinese hamster ovary (CHO) cells decrease with increasing pCO(2) in a dose-dependent manner, independent of any change in NCAM content. The results are highly pH-dependent, with a greater decrease in PSA at higher pH. By manipulating medium pH and pCO(2), we showed that decreases in PSA correlate well with bicarbonate concentration ([HCO(3)(-)]). In fact, it was possible to offset a 60% decrease in PSA content at 120 mm Hg pCO(2) by decreasing the pH from 7.3 to 6.9, such that [HCO(3)(-)] was lowered to that of control (38 mm Hg pCO(2)). When the increase in osmolality associated with elevated [HCO(3)(-)] was offset by decreasing the basal medium [NaCl], elevated [HCO(3)(-)] still caused a decrease in PSA, although less extensive than without osmolality control. By increasing [NaCl], we show that hyperosmolality alone decreases PSA content, but to a lesser extent than for the same osmolality increase due to elevated [NaHCO(3)]. In conclusion, we demonstrate the importance of pH and pCO(2) interactions, and show that [HCO(3)(-)] and osmolality can account for the observed changes in PSA content over a wide range of pH and pCO(2) values.

744 ||| 8592004 ||| (5, 0.86) ||| 5 ||| 0 ||| Thermotolerance and intracellular pH in two Chinese hamster cell lines adapted to growth at low pH. As an in vitro model for the low extracellular pH (pHe) which has frequently been observed in tumors, cell lines have been grown in a low-pH medium in order to allow cell adaptation to that milieu. Two Chinese hamster cell lines [Chinese hamster ovary (CHO) and Chinese hamster ovarian carcinoma (OvCa)] were compared, both of which acquired thermotolerance during 42 degrees C heating in pHe = 7.3 buffer, but not in pHe = 6.7 medium unless grown at that pH long enough to become adapted. CHO cells, even when acutely acidified, showed higher intracellular pH (pHi) values in a suspension assay than OvCa cells, which confirmed the danger of comparing absolute values of pHi between cell lines. Despite this fundamental difference, relative changes in pHi were similar in that both lines showed a higher pHi in adapted than in unadapted cells, over the range of pHe values tested. The upregulation of pHi was statistically significant, but the two lines differed in the time frame over which adaptation occurred. OvCa cells acquired an enhanced ability to develop tolerance to 42 degrees heat at pHe = 6.7 in 4 days, but the CHO cells acquired this ability more progressively, achieving a maximum ability at approximately 100 days. In contrast, both lines were able to upregulate their pHi within 4 hours of being exposed to pH 6.7 medium. A further indication of different biochemical mechanisms at work was the opposite effects seen on pHi in the two cell lines upon the removal of extracellular CO2/HCO3-. The differential between adapted and unadapted OvCa cells was enhanced by removal of bicarbonate, whereas CHO cells seemed less stable and the data with greater scatter failed to show any difference between adapted and unadapted cells.

745 ||| 9428798 ||| (5, 0.82) ||| 5 ||| 0 ||| Multiple subphases of DNA replication in Chinese hamster ovary (CHO-K1) cells. Replicative DNA synthesis has been measured throughout the S phase in synchronized populations of Chinese hamster ovary cells. When exponentially growing, cells in suspension cultures were subjected to counterflow centrifugal elutriation and the resolution power was increased the biphasic replication profile has been resolved and multiple subphases were distinguished. These replication peaks, termed replication checkpoints, are distributed evenly throughout the S phase. The replication checkpoints have been characterized by their average C values corresponding to 2.05, 2.12, 2.2, 2.45, 2.6, 2.8, 2.95, 3.15, 3.3, 3.45, and 3.85.

746 ||| 10904546 ||| (5, 0.81) ||| 5 ||| 0 ||| Apoptosis is regulated by polyamines in the cell cycle of Chinese hamster ovary cells. This paper deals with the relationship between the polyamine metabolism and apoptosis in the different phases of the cell cycle in a Chinese hamster ovary (CHO) cell line. Synchronously growing cells were obtained by the addition of 1.2 mM hydroxyurea and the progression through the cell cycle was monitored by determining the incorporation of 3H-thymidine in the DNA. Ornithine decarboxylase (ODC) activity showed a peak in S phase, while intracellular putrescine and spermine contents increased constantly, reaching to a maximum level at G2 phase; spermidine content doubled during G2 and increased four times during M, compared to G1. The increment in the endogenous polyamine content was associated to a diminished uptake from the medium. The apoptotic index was higher in G2 phase, coinciding with the maximum level observed in putrescine content. The results support the idea that intracellular putrescine level is closely related to apoptosis.

747 ||| 19547928 ||| (5, 0.78) ||| 5 ||| 0 ||| Relationship between tissue plasminogen activator production and specific growth rate in Chinese hamster ovary cells cultured in mannose at low temperature. Chinese hamster ovary (CHO) cells, producing human recombinant tissue plasminogen activator (tPA), were grown with mannose (5, 20 and 40 mM) instead of glucose at 31, 33 and 37 degrees C. The highest tPA concentration (1.5 mg l(-1) at 144 h of cultivation) and tPA specific production rate (47 ng 10(-6) cell h(-1)) were obtained at 31 degrees C and 40 mM mannose. Regardless of the temperature or mannose concentration used, an inverse relationship between the specific growth rate and tPA specific production rate was observed, suggesting that tPA production rate would be directly controlled by the growth rate.

748 ||| 18629861 ||| (5, 0.76) ||| 5 ||| 0 ||| Effects of elevated pCO(2) and/or osmolality on the growth and recombinant tPA production of CHO cells. Carbon dioxide is a by-product of mammalian cell metabolism that will build up in culture if it is not removed from the medium. Increased carbon dioxide levels are generally not a problem in bench-top bioreactors, but inhibitory levels can easily be reached in large-scale vessels, especially if the aeration gas is enriched in oxygen. Due to the equilibrium attained between dissolved CO(2) and bicarbonate, increased pCO(2) is associated with increased osmolality in bioreactors with pH control. While a few preliminary reports indicate that elevated pCO(2) levels can inhibit cell growth and/or recombinant protein production, no comprehensive analysis of the interrelated effects of elevated pCO(2) and osmolality has been published. We have examined the effects of 140, 195, and 250 mm Hg (187, 260, and 333 mbar, respectively) pCO(2) (with and without osmolality control) on the growth of and recombinant tPA production by MT2-1-8 Chinese hamster ovary (CHO) cells. The effects of elevated osmolality were also investigated at the control pCO(2) of 36 mm Hg. Elevated pCO(2) at 310 mOsm/kg osmolality inhibited cell growth in a dose-dependent fashion, with a maximum decrease of 30% in the specific growth rate (mu) at 250 mm Hg. Osmolality alone had no effect on mu, but the combination of elevated pCO(2) and osmolality increased the maximal reduction in mu to 45%. Elevated pCO(2) at 310 mOsm/kg osmolality decreased the specific tPA production rate (q(tPA)) by up to 40% at 250 mm Hg. Interestingly, while increased osmolality decreased q(tPA) significantly at 140 mm Hg pCO(2), it had no effect or even increased q(tPA) at 195 and 250 mm Hg. (c) 1996 John Wiley & Sons, Inc.

749 ||| 24773761 ||| (5, 0.75) ||| 5 ||| 0 ||| Non-stationary 13C metabolic flux analysis of Chinese hamster ovary cells in batch culture using extracellular labeling highlights metabolic reversibility and compartmentation. BACKGROUND: Mapping the intracellular fluxes for established mammalian cell lines becomes increasingly important for scientific and economic reasons. However, this is being hampered by the high complexity of metabolic networks, particularly concerning compartmentation. RESULTS: Intracellular fluxes of the CHO-K1 cell line central carbon metabolism were successfully determined for a complex network using non-stationary 13C metabolic flux analysis. Mass isotopomers of extracellular metabolites were determined using [U-13C6] glucose as labeled substrate. Metabolic compartmentation and extracellular transport reversibility proved essential to successfully reproduce the dynamics of the labeling patterns. Alanine and pyruvate reversibility changed dynamically even if their net production fluxes remained constant. Cataplerotic fluxes of cytosolic phosphoenolpyruvate carboxykinase and mitochondrial malic enzyme and pyruvate carboxylase were successfully determined. Glycolytic pyruvate channeling to lactate was modeled by including a separate pyruvate pool. In the exponential growth phase, alanine, glycine and glutamate were excreted, and glutamine, aspartate, asparagine and serine were taken up; however, all these amino acids except asparagine were exchanged reversibly with the media. High fluxes were determined in the pentose phosphate pathway and the TCA cycle. The latter was fueled mainly by glucose but also by amino acid catabolism. CONCLUSIONS: The CHO-K1 central metabolism in controlled batch culture proves to be robust. It has the main purpose to ensure fast growth on a mixture of substrates and also to mitigate oxidative stress. It achieves this by using compartmentation to control NADPH and NADH availability and by simultaneous synthesis and catabolism of amino acids.

750 ||| 11787009 ||| (5, 0.73) ||| 5 ||| 0 ||| Effects of osmoprotectant compounds on NCAM polysialylation under hyperosmotic stress and elevated pCO(2). Elevated osmolality and pCO(2) have been shown to alter sialylation in a protein-specific manner. In Chinese hamster ovary (CHO)MT2-l-8 cells, tPA sialylation changed only slightly from 40 to 250 mm Hg pCO(2), whereas neural cell adhesion molecule polysialic acid (NCAM PSA) content decreased by up to 70% at 250 mm Hg pCO(2), pH 7.2. NCAM PSA content also decreased with increasing NaCl or NH(4)Cl concentration. This suggests that PSA content is a sensitive indicator of conditions that may alter glycosylation. Amino acids and their derivatives have been used to protect hybridoma and CHO cell growth under hyperosmotic stress. We examined the impact of osmoprotectants on NCAM PSA content in CHO MT2-1-8 cells under hyperosmolality (up to 545 mOsm/kg) and at 195 and 250 mm Hg pCO(2). NCAM PSA content at 545 mOsm/kg was at least two-fold greater in the presence of glycine betaine or L-proline compared to that without osmoprotectant. Surprisingly, in the presence of 20 mM glycine betaine, PSA levels were 50-60% of the control level for osmolalities ranging from 320 to 545 mOsm/kg. Thus, glycine betaine inhibits NCAM polysialylation at osmolalities below 435 mOsm/kg and is beneficial at higher osmolalities. In contrast to glycine betaine, L-proline increased PSA content by 25-120% relative to the unprotected culture at < or =545 mOsm/kg. The decrease in NCAM PSA levels of CHO MT2-1-8 cells cultured at 195 mm Hg pCO(2)-435 mOsm/kg was not mitigated by the presence of 25 mM glycine betaine, glycine, or L-threonine, even though all of these compounds enhanced cell growth. At 250 mm Hg pCO(2), all osmoprotectants tested (20 mM L-threonine, L-proline, glycine, or glycine betaine) increased NCAM polysialylation, with 20 mM glycine betaine restoring NCAM PSA to near control levels. Thus, osmoprotectants may (partially) offset changes in glycosylation, as well as the inhibition of growth, in cells under environmental stress. Supernatant beta-galactosidase levels, which increase upon alkalization of acidic organelles, did not differ significantly under elevated pCO(2) and hyperosmolality from that at control conditions.

751 ||| 23949330 ||| (5, 0.71) ||| 5 ||| 0 ||| An investigation into the relationship between metabolic responses and energy regulation in antibody-producing cell. Energy-efficient metabolic responses were often noted in high-productive cultures. To better understand these metabolic responses, an investigation into the relationship between metabolic responses and energy regulation was conducted via a comparative analysis among cultures with different energy source supplies. Both glycolysis and glutaminolysis were studied through the kinetic analyses of major extracellular metabolites concerning the fast and slow cell growth stages, respectively, as well as the time-course profiles of intracellular metabolites. In three cultures showing distinct antibody productivities, the amino acid metabolism and energy state were further examined. Both the transition of lactate from production to consumption and steady intracellular pools of pyruvate and lactate were observed to be correlated with efficient energy regulation. In addition, an efficient utilization of amino acids as the replenishment for the TCA cycle was also found in the cultures with upregulated energy metabolism. It was further revealed that the inefficient energy regulation would cause low cell productivity based on the comparative analysis of cell growth and productivity in cultures having distinct energy regulation.

752 ||| 22105551 ||| (5, 0.71) ||| 5 ||| 0 ||| Correlation of antibody production rate with glucose and lactate metabolism in Chinese hamster ovary cells. A linear relationship was found between the antibody production rate (q(mAb)) and the glucose and lactate consumption rate (q(GL)) in Chinese hamster ovary cells. Under a series of q(mAb)-perturbing conditions, q(GL) was determined and a linear relationship between q(mAb) and q(GL) was further established (R(2) = 0.914). Mitochondrial dehydrogenase activity was monitored in all the q(mAb)-perturbing conditions and showed a linear correlation with q(GL) (R(2) = 0.874) as well as with q(mAb). Taken collectively, our results establish that the metabolic parameter, q(GL), is linearly correlated with q (mAb); this finding strengthens our current understanding of process optimization for antibody production.

753 ||| 22991240 ||| (5, 0.70) ||| 5 ||| 0 ||| Flux balance analysis of CHO cells before and after a metabolic switch from lactate production to consumption. Mammalian cell cultures typically exhibit an energy inefficient phenotype characterized by the consumption of large quantities of glucose and the concomitant production of large quantities of lactate. Under certain conditions, mammalian cells can switch to a more energy efficient state during which lactate is consumed. Using a metabolic model derived from a mouse genome scale model we performed flux balance analysis of Chinese hamster ovary cells before and after a metabolic switch from lactate production (in the presence of glucose) to lactate consumption (after glucose depletion). Despite a residual degree of freedom after accounting for measurements, the calculated flux ranges and associated errors were narrow enough to enable investigation of metabolic changes across the metabolic switch. Surprisingly, the fluxes through the lower part of the TCA cycle from oxoglutarate to malate were very similar (around 60 micromol/gDW/h) for both phases. A detailed analysis of the energy metabolism showed that cells consuming lactate have an energy efficiency (total ATP produced per total C-mol substrate consumed) six times greater than lactate producing cells.

754 ||| 22683938 ||| (5, 0.69) ||| 5 ||| 0 ||| Lactate metabolism shift in CHO cell culture: the role of mitochondrial oxidative activity. Lactate production is monitored in industrial processes as a crucial metabolite for cultured mammalian cells. Typically lactate is strongly produced during the exponential growth phase, while its net consumption is frequently observed when cells enter into the stationary phase. Such a metabolic shift is desirable because it seems to favor optimal process performance. However, this shift is neither generic nor can it be easily controlled, as the mechanisms modulating lactate production/consumption in cell culture are still under investigation. In this study different lactate profiles were observed in a chemically defined medium for the parental CHO-S cells and a non-recombinant subclone. The initial lactate production phase, which is typical for fast growing cells, was similar for both cell lines. After glutamine depletion the situation changed: the parental cell line promptly switched to net lactate consumption, whereas the subclone continued to produce lactate until glucose was depleted as well. We speculated that the extra lactate production would be ascribed to a different mitochondrial oxidative capacity in the subclone. Therefore, the mitochondrial membrane potential and oxygen consumption were measured for both cell lines. Indeed, a correlation between high lactate production and a reduced oxidative metabolism was found. Interestingly, this particular metabolic phenotype was also strongly influenced by the medium composition: both cell lines underwent a switch to lactate consumption when cultivated in a second medium, while a third one promoted continuous lactate production even for the parental CHO cells. Again, the correlation between lactate profile and oxidative metabolism was confirmed, pointing to a central role of mitochondria on lactate metabolism.

755 ||| 21821143 ||| (5, 0.69) ||| 5 ||| 0 ||| Metabolic flux analysis of CHO cells at growth and non-growth phases using isotopic tracers and mass spectrometry. Chinese hamster ovary (CHO) cells are the main platform for production of biotherapeutics in the biopharmaceutical industry. However, relatively little is known about the metabolism of CHO cells in cell culture. In this work, metabolism of CHO cells was studied at the growth phase and early stationary phase using isotopic tracers and mass spectrometry. CHO cells were grown in fed-batch culture over a period of six days. On days 2 and 4, [1,2-(13)C] glucose was introduced and the labeling of intracellular metabolites was measured by gas chromatography-mass spectrometry (GC-MS) at 6, 12 and 24h following the introduction of tracer. Intracellular metabolic fluxes were quantified from measured extracellular rates and (13)C-labeling dynamics of intracellular metabolites using non-stationary (13)C-metabolic flux analysis ((13)C-MFA). The flux results revealed significant rewiring of intracellular metabolic fluxes in the transition from growth to non-growth, including changes in energy metabolism, redox metabolism, oxidative pentose phosphate pathway and anaplerosis. At the exponential phase, CHO cell metabolism was characterized by a high flux of glycolysis from glucose to lactate, anaplerosis from pyruvate to oxaloacetate and from glutamate to alpha-ketoglutarate, and cataplerosis though malic enzyme. At the stationary phase, the flux map was characterized by a reduced flux of glycolysis, net lactate uptake, oxidative pentose phosphate pathway flux, and reduced rate of anaplerosis. The fluxes of pyruvate dehydrogenase and TCA cycle were similar at the exponential and stationary phase. The results presented here provide a solid foundation for future studies of CHO cell metabolism for applications such as cell line development and medium optimization for high-titer production of recombinant proteins.

756 ||| 16233031 ||| (5, 0.68) ||| 5 ||| 0 ||| Effects of shifts up and down in osmotic pressure on production of tissue plasminogen activator by Chinese hamster ovary cells in suspension. The effects of changes in osmotic pressure on the production of tissue plasminogen activator (tPA) by Chinese hamster ovary (CHO) cells in suspension were investigated. The osmotic pressure was shifted up from 300 mOsm/kg and down from 400 or 500 mOsm/kg to 400, 350, or 300 mOsm/kg during the cell growth and tPA production phases. In both phases, the specific cell growth rate was higher at a lower osmotic pressure after the shift, whatever it was up or down. After the osmotic pressure was shifted up during the production phase, the specific tPA production rate was high at a high osmotic pressure; however, shifting the osmotic pressure down also resulted in a high specific rate of tPA production. Additionally, specific production rate increased for 2 d after upward or downward shifts during the growth phase. Shifting the osmotic pressure cyclically between 300 and 500 mOsm/kg during the production phase improved the tPA production 1.13-fold compared with the amount produced at a constant osmotic pressure of 300 mOsm/kg.

757 ||| 9369953 ||| (5, 0.67) ||| 5 ||| 0 ||| Altered proton extrusion in cells adapted to growth at low extracellular pH. Intracellular pH (pHi) homeostasis is crucial to cell survival. Cells that are chronically exposed to a low pH environment must adapt their hydrogen ion extrusion mechanisms to maintain their pHi in the physiologic range. An important component of the adaptation to growth at low pH is the upregulation of pHi relative to the extracellular pH (pHe). To test the ability of low pHe adapted cells to respond to a pHi lowering challenge, a fluorescence assay was used that directly monitors proton removal as the rate of change of pHi during recovery from cytosolic acidification. Two cell lines of Chinese hamster origin (ovarian carcinoma and ovary fibroblastoid cells) were compared, both of which showed altered proton extrusion after adaptation to growth at low pHe = 6.70. In the ovarian carcinoma (OvCa) cell line, the pattern was consistent with an upregulation by means of an increase in the number of functional proton transporters in the plasma membrane. In the ovary fibroblastoid (CHO-10B) cell line, pHi was consistently elevated in adapted cells as compared with cells grown at normal pHe = 7.30 without an increase in maximum extrusion rate. This upregulation was consistent with a shift in the activating pHi of proton transporters without an increase in the number of transporters, i.e., a change in substrate affinity of the transporter. In OvCa cells, recovery from acidification could be blocked by amiloride, an inhibitor of Na+/ H+ exchange. In contrast, a more modest effect of amiloride on CHO cells was observed but a complete inhibition was seen with the Cl-/HCO(-3)exchange inhibitor 4,4'-diisothiocyanatostilbene-2,2'-disulfonic acid (DIDS). These data indicate that the two cell lines rely to different degrees on the two major pathways for pH regulation during recovery from cytosolic acidification.

758 ||| 23381838 ||| (5, 0.67) ||| 5 ||| 0 ||| Peak antibody production is associated with increased oxidative metabolism in an industrially relevant fed-batch CHO cell culture. Cell metabolism can vary considerably over the course of a typical fed-batch antibody production process. However, the intracellular pathway alterations associated with various phases of growth and antibody production have yet to be fully elucidated using industrially relevant production hosts. Therefore, we performed (13)C labeling experiments and metabolic flux analysis (MFA) to characterize CHO cell metabolism during four separate phases of a fed-batch culture designed to closely represent industrial process conditions. First, we found that peak specific growth rate was associated with high lactate production and minimal TCA cycling. Conversely, we found that lactate metabolism switched from net production to net consumption as the culture transitioned from peak growth to peak antibody production. During the peak antibody production phase, energy was primarily generated through oxidative phosphorylation, which was also associated with elevated oxidative pentose phosphate pathway (oxPPP) activity. Interestingly, as TCA cycling and antibody production reached their peaks, specific growth rate continued to diminish as the culture entered stationary phase. However, TCA cycling and oxPPP activity remained high even as viable cell density began to decline. Overall, we found that a highly oxidative state of metabolism corresponded with peak antibody production, whereas peak cell growth was characterized by a highly glycolytic metabolic state.

759 ||| 9336989 ||| (5, 0.66) ||| 5 ||| 0 ||| Effects of ammonium and lactate on growth and metabolism of a recombinant Chinese hamster ovary cell culture. A Chinese hamster ovary (CHO) cell line producing a recombinant glycoprotein was cultured in batch mode with different initial concentrations of ammonium chloride (0-10 mM), sodium lactate (0-60 mM), or sodium chloride (0-60 mM). High ammonium concentrations did not inhibit cell growth and productivity or glucose and glutamine consumption. In contrast, specific ammonia and alanine production decreased by 55% and 40%, respectively. There were also significant increases in specific aspartate and glutamate consumption in high ammonium concentrations. These observations indicated a shift in glutamine catabolic pathways in response to the effects of ammonium. The influence of lactate on growth and metabolism were the combined effects of lactate concentration and osmolarity. After "correcting" for osmolarity effects, lactate was found to inhibit growth by 25% but to increase specific productivity slightly (10%). Lactate had profound effects not only on glycolysis but also on glutaminolysis. While specific glucose and glutamine consumptions decreased by 15-20%, the effects of lactate on their metabolic products were far more significant. Lactate production was halted, and specific ammonia and alanine productions decreased by 64% and 70% at high lactate concentration. Theories on how ammonium and lactate affected the metabolic pathways of glucose and glutamine are presented.

760 ||| 25449760 ||| (5, 0.66) ||| 5 ||| 0 ||| Titer of trastuzumab produced by a Chinese hamster ovary cell line is associated with tricarboxylic acid cycle activity rather than lactate metabolism. Achieving high productivity and quality is the final goal of therapeutic antibody development, but the productivity and quality of antibodies are known to be substantially dependent on the nature of the cell lines expressing the antibodies. We characterized two contrasting cell lines that produce trastuzumab, namely cell line A with a high titer and a low aggregate content and cell line B with a low titer and a high aggregate content to identify the causes of the differences. We observed the following differences: cell growth (A > B), proportion of defucosylated oligosaccharides on antibodies (A < B), and proportion of covalent antibody aggregates (A > B). Our results suggest that the high monoclonal antibody (mAb) titers in cell line A is associated with the high proliferation and is not caused by the lactate metabolism shift (switching from lactate production to net lactate consumption). Rather, these differences can be accounted for by the following: levels of tricarboxylic acid cycle intermediates (A > B), ammonium ion levels (A <= B), and oxidative stress (A > B).

761 ||| 11835145 ||| (5, 0.66) ||| 5 ||| 0 ||| Monitoring of temperature effects on animal cell metabolism in a packed bed process. Animal cell (Chinese Hamster Ovary) concentration was determined on-line in a packed bed process using dielectric spectroscopy. This enabled the evaluation of the effect of temperature on specific metabolic rates during 3 months of continuous culture. The effect of low cultivation temperature on cell growth and metabolism was monitored, and the data were used for process development. At 37 degrees C cells grew exponentially with a specific growth rate of 0.038 d-1 and specific glucose uptake and lactate production rates increased continually. Reduction of the temperature to 33.5 degrees C resulted in a lowering of these metabolic rates while having no effect on cell proliferation. Subsequent reduction of the temperature to 32 degrees C resulted in stabilization of the cell concentration at a high density (3.6 x 10(7) cell per mL of packed bed). In addition, the specific production rate of the protein of interest increased by a factor of 6 compared to the value at 37 degrees C. During the stationary phase at 32 degrees C, all other specific metabolic rates could be controlled to low and constant levels.

762 ||| 10875440 ||| (5, 0.63) ||| 5 ||| 0 ||| Differential effects of zinc on amyloid precursor protein (APP) processing in copper-resistant variants of cultured Chinese hamster ovary cells. Previous studies have demonstrated that adding copper to Chinese-hamster ovary (CHO) cells greatly reduced the levels of beta-amyloid (Abeta) peptide in parental CHO-K1 and in copper resistant CHO-CUR3 cells which have lower intracellular copper levels. In the current study, zinc, the zinc chelator 1,10-phenanthroline or copper chelators bathocuproine and D-penicillamine were added to the culture media of stably transfected CHO cells. The data show that zinc up to concentrations of 50 microM or the presence of 1,10-phenanthroline specifically increased the level of secreted APP in CHO-K1 cells. By contrast, the level of secreted APP in CHO-CUR3 cells remained unaffected. APP holoprotein increased dramatically in CHO-CUR3 cells compared with CHO-K1 cells. The large decrease of Abeta release seen in both cell lines at elevated extracellular zinc levels was due to specific inhibition of secretion. These results indicate that a disturbed zinc-homeostasis may be an important factor influencing APP production, transport and processing.

763 ||| 23111062 ||| (5, 0.59) ||| 5 ||| 0 ||| Parallel labeling experiments with [1,2-(13)C]glucose and [U-(13)C]glutamine provide new insights into CHO cell metabolism. We applied a parallel labeling strategy using two isotopic tracers, [1,2-(13)C]glucose and [U-(13)C]glutamine, to determine metabolic fluxes in Chinese hamster ovary (CHO) cells. CHO cells were grown in parallel cultures over a period of six days with glucose and glutamine feeding. On days 2 and 5, isotopic tracers were introduced and (13)C-labeling of intracellular metabolites was measured by gas chromatography-mass spectrometry (GC-MS). Metabolites in glycolysis pathway reached isotopic steady state for [1,2-(13)C]glucose within 1.5h, and metabolites in the TCA cycle reached isotopic steady state for [U-(13)C]glutamine within 3h. Combined analysis of multiple data sets produced detailed flux maps at two key metabolic phases, exponential growth phase (day 2) and early stationary phase (day 5). Flux results revealed significant rewiring of intracellular metabolism in the transition from growth to non-growth, including changes in oxidative pentose phosphate pathway, anaplerosis, amino acid metabolism, and fatty acid biosynthesis. At the growth phase, de novo fatty acid biosynthesis correlated well with the lipid requirements for cell growth. However, surprisingly, at the non-growth phase the fatty acid biosynthesis flux remained high even though no new lipids were needed for cell growth. Additionally, we identified a discrepancy in the estimated TCA cycle flux obtained using traditional stoichiometric flux balancing and (13)C-metabolic flux analysis. Our results suggested that CHO cells produced additional metabolites from glucose that were not captured in previous metabolic models. Follow-up experiments with [U-(13)C]glucose confirmed that additional metabolites were accumulating in the medium that became M+3 and M+6 labeled.

764 ||| 20672285 ||| (5, 0.58) ||| 5 ||| 0 ||| Metabolic flux analysis of CHO cell metabolism in the late non-growth phase. Chinese hamster ovary (CHO) cell cultures are commonly used for production of recombinant human therapeutic proteins. Often the goal of such a process is to separate the growth phase of the cells, from the non-growth phase where ideally the cells are diverting resources to produce the protein of interest. Characterizing the way that the cells use nutrients in terms of metabolic fluxes as a function of culture conditions can provide a deeper understanding of the cell biology offering guidance for process improvements. To evaluate the fluxes, metabolic flux analysis of the CHO cell culture in the non-growth phase was performed by a combination of steady-state isotopomer balancing and stoichiometric modeling. Analysis of the glycolytic pathway and pentose phosphate pathway (PPP) indicated that almost all of the consumed glucose is diverted towards PPP with a high NADPH production; with even recycle from PPP to G6P in some cases. Almost all of the pyruvate produced from glycolysis entered the TCA cycle with little or no lactate production. Comparison of the non-growth phase against previously reported fluxes from growth phase cultures indicated marked differences in the fluxes, in terms of the split between glycolysis and PPP, and also around the pyruvate node. Possible reasons for the high NADPH production are also discussed. Evaluation of the fluxes indicated that the medium strength, carbon dioxide level, and temperature with dissolved oxygen have statistically significant impacts on different nodes of the flux network.

765 ||| 19003317 ||| (5, 0.58) ||| 5 ||| 0 ||| Increased productivity of recombinant tissular plasminogen activator (t-PA) by butyrate and shift of temperature: a cell cycle phases analysis. Directed control of cell metabolism by a modification of the physicochemical conditions (presence of Na-butyrate and modification of the temperature) was used to modulate the productivity of human recombinant tissular plasminogen activator (t-PA) expressed under control of SV40 promoter in Chinese Hamster Ovary (CHO) cell lines. We showed that both by adding Na-butyrate or lowering temperature from 37 degrees C to 32 degrees C there is an increase in the amount of t-PA excreted, while cell growth is significantly reduced. The treatments also increased the intracellular amount of t-PA. We measured the distribution of cell cycle phases by cytometry and used a modification of the equations of Kromenaker and Srienc (1991, 1994 a, b) to analyse the intracellular t-PA production rate in the different cell cycle phases. Intracellular t-PA was shown to accumulate in G1 phase in all conditions (at 37 degrees C, at 32 degrees C and in presence of butyrate). Moreover, we have shown that the distribution of the time cells treated by butyrate are maintained in the G1cell cycle phase is significantly increased. t-PA produced in the different cell culture conditions tested was analysed by zymogram and western blotting: neither butyrate, neither the shift of temperature changed significantly the overall quality of the protein. The N-glycan patterns of recombinant human t-PA was also analysed with carbohydrate-specific lectins. Butyrate caused a transitory increase in N-linked complex high-mannose oligosaccharides without any effect on the sialic acid content of t-PA.

766 ||| 17086415 ||| (5, 0.58) ||| 5 ||| 0 ||| Down-regulation of lactate dehydrogenase-A by siRNAs for reduced lactic acid formation of Chinese hamster ovary cells producing thrombopoietin. Lactate, one of the major waste products in mammalian cell culture, can inhibit cell growth and affect cellular metabolism at high concentrations. To reduce lactate formation, lactate dehydrogenase-A (LDH-A), an enzyme catalyzing the conversion of glucose-derived pyruvate to lactate, was down-regulated by an expression vector of small interfering RNAs (siRNA) in recombinant Chinese hamster ovary (rCHO) cells producing human thrombopoietin (hTPO). Three clones expressing low levels of LDH-A, determined by reverse transcription-PCR and an enzyme activity test, were established in addition to a negative control cell line. LDH-A activities in the three clones were decreased by 75-89%, compared with that of the control CHO cell line, demonstrating that the effect of siRNA is more significant than that of other traditional methods such as homologous recombination (30%) and antisense mRNA (29%). The specific glucose consumption rates of the three clones were reduced to 54-87% when compared to the control cell line. Similarly, the specific lactate production rates were reduced to 45-79% of the control cell line level. In addition, reduction of LDH-A did not impair either cell proliferation or hTPO productivity. Taken together, these results show that the lactate formation rate in rCHO cell culture can be efficiently reduced through the down-regulation of LDH via siRNA.

767 ||| 21964570 ||| (5, 0.57) ||| 5 ||| 0 ||| Comparative metabolite analysis to understand lactate metabolism shift in Chinese hamster ovary cell culture process. A metabolic shift from lactate production (LP) to net lactate consumption (LC) phenotype was observed in certain Chinese hamster ovary (CHO) cell lines during the implementation of a new chemically defined medium (CDM) formulation for antibody production. In addition, this metabolic shift typically leads to process performance improvements in cell growth, productivity, process robustness, and scalability. In our previous studies, a correlation between a key media component, copper, and this lactate metabolism shift was observed. To further investigate this phenomenon, two complementary studies were conducted. In the first study, a single cell line was cultivated in two media that only differed in their copper concentrations, yet were known to generate an LP or LC phenotype with that cell line. In the second study, two different cell lines, which were known to possess inherently different lactate metabolic characteristics, were cultivated in the same medium with a high level of copper; one cell line produced lactate throughout the duration of the culture, and the other consumed lactate after an initial period of LP. Cell pellet and supernatant samples from both studies were collected at regular time intervals, and their metabolite profiles were investigated. The primary finding from the metabolic analysis was that the cells in LP conditions exhibited a less efficient energy metabolism, with glucose primarily being converted into pyruvate, sorbitol, lactate, and other glycolytic intermediates. This decrease in energy efficiency may be due to an inability of pyruvate and acetyl-CoA to progress into the TCA cycle. The lack of progression into the TCA cycle or overflow metabolism in the LP phenotype resulted in the inadequate supply of ATP for the cells. As a consequence, the glycolysis pathway remained the major source of ATP, which in turn, resulted in continuous LP throughout the culture. In addition, the accumulation of free fatty acids was observed; this was thought to be a result of phospholipid catabolism that was being used to supplement the energy produced through glycolysis in order to meet the needs of LP cells. A thorough review of the metabolic profiles indicated that the lactate metabolic shift could be related to the oxidative metabolic capacity of cells.

768 ||| 19002978 ||| (5, 0.56) ||| 5 ||| 0 ||| The effect of osmolarity on metabolism and morphology in adhesion and suspension chinese hamster ovary cells producing tissue plasminogen activator. The effects of constant osmolarity, between 300 and500 mOsm/kg, on the metabolism of Chinese HamsterOvary (CHO) cells producing tissue plasminogenactivator (tPA) were compared between adhesion andsuspension cultures. In both suspension and adhesionculture, the specific rates of glucose consumption(nu(G)), lactate production (q(L)), and tPAproduction (q(tPA)) increased as osmolarityincreased, while these rates decreased when osmolaritywas higher than the respective critical levels. However, specific growth rate (mu) decreased withincrease in osmolarity and this slope grew steeper inthe osmolarity range higher than the critical level. The decrease in mu in the adhesion culture was morerapid than that in the suspension culture. Thecritical osmolarity for adhesion culture (400 mOsm/kg)was lower than that for suspension culture (450 mOsm/kg). These results indicated that the adhesionculture was more sensitive to increase of osmolaritythan the suspension culture, while the specific ratesobtained from the adhesion cultures were in general1.5- to 3-fold higher than those obtained from thesuspension cultures. Cell volume increased asosmolarity increased in both the suspension andadhesion cultures, as reported previously forsuspension culture of hybridoma cells, but there wasno morphological change in the suspension culture. Incontrast, cell height decreased and cell adhesion areamarkedly increased as osmolarity increased in theadhesion culture. This morphological change inadhesion cultures may be one reason for the highersensitivity of adherent cells to the increase ofosmolarity than suspended cells.

769 ||| 19002908 ||| (5, 0.56) ||| 5 ||| 0 ||| Enhancement of recombinant erythropoietin production in CHO cells in an incubator without CO(2) addition. The effect of low levels of carbon dioxide (CO(2)) in the gas phase on the production of recombinant human erythropoietin (EPO)in CHO cells was explored. A T-flask culture in an incubator without CO(2) addition showed a slow cell growth initially followed by the cessation of growth, while other cultures incubated under 0.5-5% CO(2) concentrations grew normally at the same rate during the entire period of cultivation. Interestingly, the production of EPO in the culture incubated under no CO(2) supply was highest among the tested cultures. The cell specific secretion rate of EPO (q(EPO)) of the culture under no CO(2) supply was about 3 times higher than that of the culture under 5% CO(2) supply. Western blot analysis and in vivo bioassay of EPO showed no apparent changes in EPO quality between the two cases of different CO(2) environments (air vs. 5% CO(2)), suggesting robust glycosylation of EPO by CHO cells even under very reduced CO(2) environment. Various combinations of the two extreme cases, with 5% CO(2) supply (suitable for cell growth) and no CO(2) addition (better for EPO production), were made in order to maximize the volumetric productivity of EPO secretion (P(V)) in CHO cells. The P(V) of the cultures programmed with initial incubation under 5% CO(2) followed by no CO(2) supply was about 2 times superior to that of the culture incubated only under no CO(2) supply. The P(V) of the culture under no CO(2) supply was slightly lower than that of culture grown under 5% CO(2). However, the q(EPO) of the no CO(2) supply case was more than 5 times higher than that of the culture under 5% CO(2) supply. In conclusion, we have demonstrated that a simple programming of CO(2) supply to an incubator can enhance the production of EPO in CHO cells remarkably, without any apparent change of the EPO quality.

770 ||| 15504103 ||| (5, 0.56) ||| 5 ||| 0 ||| A detailed understanding of the enhanced hypothermic productivity of interferon-gamma by Chinese-hamster ovary cells. Culturing CHO (Chinese-hamster ovary) cells at low temperature leads to growth arrest in the G0/G1 phase of the cell cycle and, in many cases, causes an increase in the specific productivity of recombinant protein. Controlled proliferation is often used to increase CHO specific productivity, and thus there is speculation that the enhanced productivity at low temperature is due to G0/G1-phase growth arrest. However, we show that the positive effect of low temperature on recombinant protein production is due to elevated mRNA levels and not due to growth arrest and that a cell line can still exhibit growth-associated productivity at low temperatures. Using a CHO cell producing recombinant human IFN-gamma (interferon-gamma), we show that productivity increases as the percentage of cells in the S phase of the cell cycle increases, at both 32 and 37 degrees C. The increased productivity is due to higher recombinant IFN-gamma mRNA levels. We also show that, for a given cell-cycle distribution, specific productivity increases as the temperature is lowered from 37 to 32 degrees C. Thus specific productivity is maximized when cells are actively growing (high percentage of S-phase cells) and also exposed to low temperature. These findings have important implications for cell-culture optimization and cell-line engineering, providing evidence that a CHO cell line capable of actively growing at low temperature would provide improved total protein production relative to the current growth strategies, namely 37 degrees C active growth or low-temperature growth arrest.

771 ||| 25504679 ||| (5, 0.55) ||| 5 ||| 0 ||| Manganese increases high mannose glycoform on monoclonal antibody expressed in CHO when glucose is absent or limiting: Implications for use of alternate sugars. Alternate sugars such as galactose and fructose are metabolized at a slower rate than glucose and result in lower accumulation of lactate. While low lactate accumulation is desirable, we report that complete substitution of glucose with these sugars results in an increase in M5 high mannose glycans. Surprisingly, this increase is much higher when the culture is supplemented with manganese: for example, when cells are cultured with galactose, M5 high mannose glycan content increased from 5% at 1 nM Mn(2+) in the basal medium to 32% with 16 microM Mn(2+) supplementation. When galactose is supplemented with glucose maintained at low concentrations, a small reduction in high mannose glycans is seen. In control cultures with glucose, the high mannose content was however <2% in this range of Mn(2+) concentration. By varying Mn(2+) and glucose supplementation levels, with or without galactose, we systematically demonstrate that Mn(2+) concentration and glucose availability, together, significantly affect the high mannose glycan content. To our knowledge, this is the first report that shows that the effect of Mn(2+) on high mannose glycan content depends on glucose availability. At each Mn(2+) supplementation level evaluated, galactosylation percentages were highest for cultures where galactose was supplemented with glucose at non-limiting concentration.

772 ||| 19002878 ||| (5, 0.55) ||| 5 ||| 0 ||| pH Condition in temperature shift cultivation enhances cell longevity and specific hMab productivity in CHO culture. Controlling cell proliferation during cell culturing is an effective way to improve the production yield in mammalian cell culture. We examined the effect of temperature shifts (TS) under pH control conditions in Chinese hamster ovary cells. When we shifted the culture temperature from 37 degrees C to 31 degrees C before a stationary phase at pH 6.8 (TS/pH 6.8), cell viability remained high, and the final human monoclonal antibody (hMab) concentration increased to 2.3 times that in the culture remaining at 37 degrees C. However, there were no significant effects on the cell viability or production yield with the same TS at pH 7.0 (TS/pH 7.0). The average specific hMab productivity and mRNA level of TS/pH 7.0 were the same as that of TS/pH 6.8. The control of cell growth by the TS or the addition of rapamycin was effective in the maintenance of cell viability, but there was no significant increase of the average specific hMab productivity in the culture where cell proliferation was controlled with rapamycin. The hMab mRNA concentration decreased to 55%-65% at a 37 degrees C culture with the addition of actinomycin D. In contrast, actinomycin D did not affect the mRNA level in the TS culture. This result suggested that the increase in the mRNA level in the TS condition was caused by an increase in mRNA stability. In this study, we show that TS can produce two unrelated effects: a prolongation of cell longevity and an improvement in mRNA stability.

773 ||| 21392546 ||| (5, 0.54) ||| 5 ||| 0 ||| Decreasing lactate level and increasing antibody production in Chinese Hamster Ovary cells (CHO) by reducing the expression of lactate dehydrogenase and pyruvate dehydrogenase kinases. Large-scale fed-batch cell culture processes of CHO cells are the standard platform for the clinical and commercial production of monoclonal antibodies. Lactate is one of the major by-products of CHO fed-batch culture. In pH-controlled bioreactors, accumulation of high levels of lactate is accompanied by high osmolality due to the addition of base to control pH of the cell culture medium, potentially leading to lower cell growth and lower therapeutic protein production during manufacturing. Lactate dehydrogenase (LDH) is an enzyme that catalyzes the conversion of the substrate, pyruvate, into lactate and many factors including pyruvate concentration modulate LDH activity. Alternately, pyruvate can be converted to acetyl-CoA by pyruvate dehydrogenases (PDHs), to be metabolized in the TCA cycle. PDH activity is inhibited when phosphorylated by pyruvate dehydrogenase kinases (PDHKs). In this study, we knocked down the gene expression of lactate dehydrogenase A (LDHa) and PDHKs to investigate the effect on lactate metabolism and protein production. We found that LDHa and PDHKs can be successfully downregulated simultaneously using a single targeting vector carrying small inhibitory RNAs (siRNA) for LDHa and PDHKs. Moreover, our fed-batch shake flask evaluation data using siRNA-mediated LDHa/PDHKs knockdown clones showed that downregulating LDHa and PDHKs in CHO cells expressing a therapeutic monoclonal antibody reduced lactate production, increased specific productivity and volumetric antibody production by approximately 90%, 75% and 68%, respectively, without appreciable impact on cell growth. Similar trends of lower lactate level and higher antibody productivity on average in siRNA clones were also observed from evaluations performed in bioreactors.

774 ||| 9299777 ||| (5, 0.53) ||| 5 ||| 0 ||| The effect of oxidative stress on the production of the recombinant protein, interferon gamma, produced by Chinese hamster ovary cells in stirred-batch culture. The CHO320 cell line, engineered to produce human interferon gamma was investigated with regard to its susceptibility to oxidative stress. Batch cultures of the cells grown in a bench-top bioreactor exhibited no marked response to changes in oxygen concentration between 6% and 14% whereas cell growth and recombinant protein production were inhibited by increasing the oxygen to 20%. High concentrations of hydrogen peroxide (in excess of 200 microM) were required to inhibit growth of the CHO320 cells whereas concentrations of 50 microns and 100 microM had no effect on recombinant protein production. Buthionine sulphoximine (50 microM and 100 microM) completely depleted the cells of glutathione within 24 h; however, no quantitative effect on recombinant protein production was seen. It is concluded that the CHO320 cells are, possibly as a consequence of the long selection process they have undergone, very resistant to oxidative stress.

775 ||| 24525334 ||| (5, 0.52) ||| 5 ||| 0 ||| Metabolic control at the cytosol-mitochondria interface in different growth phases of CHO cells. Metabolism at the cytosol-mitochondria interface and its regulation is of major importance particularly for efficient production of biopharmaceuticals in Chinese hamster ovary (CHO) cells but also in many diseases. We used a novel systems-oriented approach combining dynamic metabolic flux analysis and determination of compartmental enzyme activities to obtain systems level information with functional, spatial and temporal resolution. Integrating these multiple levels of information, we were able to investigate the interaction of glycolysis and TCA cycle and its metabolic control. We characterized metabolic phases in CHO batch cultivation and assessed metabolic efficiency extending the concept of metabolic ratios. Comparing in situ enzyme activities including their compartmental localization with in vivo metabolic fluxes, we were able to identify limiting steps in glycolysis and TCA cycle. Our data point to a significant contribution of substrate channeling to glycolytic regulation. We show how glycolytic channeling heavily affects the availability of pyruvate for the mitochondria. Finally, we show that the activities of transaminases and anaplerotic enzymes are tailored to permit a balanced supply of pyruvate and oxaloacetate to the TCA cycle in the respective metabolic states. We demonstrate that knowledge about metabolic control can be gained by correlating in vivo metabolic flux dynamics with time and space resolved in situ enzyme activities.

776 ||| 24130854 ||| (5, 0.52) ||| 5 ||| 0 ||| 1H NMR spectroscopy profiling of metabolic reprogramming of Chinese hamster ovary cells upon a temperature shift during culture. We report an NMR based approach to determine the metabolic reprogramming of Chinese hamster ovary cells upon a temperature shift during culture by investigating the extracellular cell culture media and intracellular metabolome of CHOK1 and CHO-S cells during culture and in response to cold-shock and subsequent recovery from hypothermic culturing. A total of 24 components were identified for CHOK1 and 29 components identified for CHO-S cell systems including the observation that CHO-S media contains 5.6 times the level of glucose of CHOK1 media at time zero. We confirm that an NMR metabolic approach provides quantitative analysis of components such as glucose and alanine with both cell lines responding in a similar manner and comparable to previously reported data. However, analysis of lactate confirms a differentiation between CHOK1 and CHO-S and that reprogramming of metabolism in response to temperature was cell line specific. The significance of our results is presented using principal component analysis (PCA) that confirms changes in metabolite profile in response to temperature and recovery. Ultimately, our approach demonstrates the capability of NMR providing real-time analysis to detect reprogramming of metabolism upon cellular perception of cold-shock/sub-physiological temperatures. This has the potential to allow manipulation of metabolites in culture supernatant to improve growth or productivity.

777 ||| 19619865 ||| (5, 0.52) ||| 5 ||| 0 ||| Na-butyrate sustains energetic states of metabolism and t-PA productivity of CHO cells. The effects of Na-butyrate on the physiological behaviour and on the specific productivity of recombinant tissue plasminogen activator (t-PA) Chinese Hamster Ovary (CHO) cells were characterized. Batch cultures were performed in a 3.5-L bioreactor. Na-butyrate was added either at the mid-exponential growth phase (48 h) or at the end of the exponential growth phase (74 h). The cultures with Na-butyrate showed higher net specific productivity of t-PA and lower final cell density and viability. Maximum specific productivity of t-PA for all cultures coincided with the early plateau phase (84 h). The cell's specific oxygen uptake rate (qO2) increased after the Na-butyrate addition and remained higher than that of the controlled culture. Triphosphate nucleotides, ADP, AMP and UDP-sugars all increased after 84 h in the cultures with Na-butyrate, showing different behaviours when Na-butyrate was added at 48 h or 74 h. Na-butyrate did not affect the cell's adenylate energy charge until the cell's viability started to decrease (156-168 h). The controlled culture and the culture with Na-butyrate addition, showed at 74 h, similar time trends as for purine and nucleotide ratios ((ATP+GTP)/(UTP+CTP) and UTP/ATP) with clear shifts in behaviour at 84 h and 168 h. However, the addition of Na-butyrate at 48 h resulted in damped variations of purine and nucleotide ratios in comparison to both the control culture and the culture with Na-butyrate addition at 74 h.

778 ||| 10861399 ||| (5, 0.52) ||| 5 ||| 0 ||| Limitations to the amplification and stability of human tissue-type plasminogen activator expression by Chinese hamster ovary cells. Chinese hamster ovary cell production of recombinant tissue-type plasminogen activator (t-PA) was increased by amplification of cotransfected dihydrofolate reductase cDNA using stepwise adaptation to increasing methotrexate (MTX) concentrations. The highest producing clones were isolated at 5 microM MTX and yielded 26,000 U/10(6) cells/day t-PA (43 microgram/10(6) cells/day). Above 25 microM MTX, cell specific t-PA production rates became increasingly variable and the cDNA copynumbers decreased. No apparent correlation between the cell specific t-PA production rate and the growth rate was observed upon subcloning of the amplified cells. When MTX selection was removed, the t-PA production rate decreased up to tenfold within 40 days; this was accompanied by an up to 60% drop in cDNA copynumber. Subclones isolated after 108 days of culture in the absence of MTX were, on average, sixfold more stable than their parental cells. In culture without MTX, the maximum stable t-PA production rate obtained (over 250 days) was 7000 +/- 750 U/10(6) cells/day (approximately 12 microgram/10(6) cells/day), approximately threefold lower than the maximum unstable levels of production reached under selective pressure. Taken together, these results define a wide range of the highest t-PA expression rates obtained under MTX selection, for which stable expression without selection has not been reported.

779 ||| 24635945 ||| (5, 0.51) ||| 5 ||| 0 ||| Efficient folding/assembly in Chinese hamster ovary cells is critical for high quality (low aggregate content) of secreted trastuzumab as well as for high production: stepwise multivariate regression analyses. When developing cell culture processes for therapeutic antibodies, the low content of aggregated proteins is the most critical because administering aggregated antibody molecules might result in adverse effects such as immunogenicity. To characterize cells with high productivity and quality, we determined factors that are closely related to antibody titer, which is a productivity indicator, and the area percentage of high molecular weight species in cultivated media, which is equivalent to aggregate content and is used as a quality indicator. We examined the factors influencing antibody titer and aggregate content using various data from 28 cell lines throughout their culture periods from growth to death phases. Our study using correlation analysis revealed that statistically significant correlations between factors and indicators changes with sampling points, hence we thought that various factors would influence each indicator simultaneously. To understand the relationship between these factors and titer/aggregates contents, we performed stepwise multiple linear regression analyses and deduced a multiple linear model for each indicator. The titer was found to positively associate with specific growth rate and specific production rate and negatively with intracellular heavy chain content. The aggregate content was found to positively associate with protein disulfide isomerase mRNA level and negatively with light chain secreted into culture media, specific production rate, intracellular light chain content, and specific growth rate. Our observations suggest that correct and efficient assembling and/or folding of an antibody molecule in an endoplasmic reticulum are important for high titer and low aggregates contents.

780 ||| 24403277 ||| (5, 0.50) ||| 5 ||| 0 ||| Effects of copper on CHO cells: insights from gene expression analyses. Copper concentration can impact lactate metabolism in Chinese Hamster ovary (CHO) cells. In our previous study, a 20-fold increase in initial copper concentration enabled CHO cultures to shift from net lactate production to net lactate consumption, and achieve higher cell growth and productivity. In this follow-up study, we used transcriptomics to investigate the mechanism of action (MOA) of copper that mediates this beneficial metabolism shift. From microarray profiling (days 0-7), the number of differentially expressed genes increased considerably after the lactate shift (>day 3). To uncouple the effects of copper at early time points (days 0-3) from that of lactate per se (>day 3), and to validate microarray hits, we analyzed samples before the lactate shift by RNA-Seq. Out of 6,398 overlapping genes analyzed by both transcriptomic methods, only the early growth response 1 gene-coding for a transcription factor that activates signaling pathways in response to environmental stimuli-satisfied the differential expression criteria (fold change >= 1.5; P < 0.05). Gene expression correlation and biological pathway analyses further confirmed that copper differences exerted minimal transcriptional impact on the CHO cultures before the lactate shift. By contrast, genes associated with hypoxia network and oxidative stress response were upregulated after the lactate shift. These upregulations should boost cell proliferation and survival, but do not account for the preceding shift in lactate metabolism. The findings here indicate that the primary MOA of copper that enabled the shift in lactate metabolism is not at the transcriptional level.

781 ||| 10989172 ||| (5, 0.50) ||| 5 ||| 0 ||| Expression of carbamoyl phosphate synthetase I and ornithine transcarbamoylase genes in Chinese hamster ovary dhfr-cells decreases accumulation of ammonium ion in culture media. Ammonium ion accumulation in mammalian cell culture media causes toxicity which inhibits cell growth and productivity. To reduce the level of the accumulated ammonium ion, carbamoyl phosphate synthetase I (CPS I) and ornithine transcarbamoylase (OTC) were used, which catalyze the first and second steps of the urea cycle in the liver. To examine the effects of overexpressed CPS I and OTC genes on the concentration of the ammonium ion in culture media, the two genes were introduced into Chinese hamster ovary (CHO) dhfr-cells. The CPS I expressing cell lines (CPS I-CHO) and both CPS I and OTC expressing cell lines (CPS I/OTC-CHO) were confirmed at the mRNA level and analyzed in terms of the cell growth and the accumulation of ammonium ion in culture media. The accumulation of ammonium ion was approximately 25-33% less in CPS I/OTC-CHO than in either CPS I-CHO or the vector-control cell lines. Interestingly however, the cell growth was approximately 15-30% faster in both CPS I-CHO and CPS I/OTC-CHO than in the control cell lines. Forced expression of urea cycle enzymes in the CHO cells revealed that both the expression of CPS I and OTC can reduce the accumulation of ammonium ion in the culture media.

782 ||| 24699760 ||| (5, 0.49) ||| 5 ||| 0 ||| Differential effect of culture temperature and specific growth rate on CHO cell behavior in chemostat culture. Mild hypothermia condition in mammalian cell culture technology has been one of the main focuses of research for the development of breeding strategies to maximize productivity of these production systems. Despite the large number of studies that show positive effects of mild hypothermia on specific productivity of r-proteins, no experimental approach has addressed the indirect effect of lower temperatures on specific cell growth rate, nor how this condition possibly affects less specific productivity of r-proteins. To separately analyze the effects of mild hypothermia and specific growth rate on CHO cell metabolism and recombinant human tissue plasminogen activator productivity as a model system, high dilution rate (0.017 h(-1)) and low dilution rate (0.012 h(-1)) at two cultivation temperatures (37 and 33 C) were evaluated using chemostat culture. The results showed a positive effect on the specific productivity of r-protein with decreasing specific growth rate at 33 C. Differential effect was achieved by mild hypothermia on the specific productivity of r-protein, contrary to the evidence reported in batch culture. Interestingly, reduction of metabolism could not be associated with a decrease in culture temperature, but rather with a decrease in specific growth rate.

783 ||| 24771076 ||| (5, 0.47),(1, 0.30) ||| 5 ||| 0 ||| Metabolic responses of CHO cells to limitation of key amino acids. Chinese hamster ovary (CHO) cells are the predominant host for production of therapeutic glycoproteins. In particular, the glutamine-synthetase (GS) expression system has been widely used in the biopharmaceutical industry for efficient selection of high-yielding clones. However, much remains unclear on how metabolic wiring affects culture performance. For instance, asparagine and serine have been observed to be the largest nitrogen sources taken up by GS-CHO cells, but their roles in biosynthesis and energy generation are poorly understood. In this work, a comprehensive profiling of extracellular metabolites coupled with an analysis of intracellular label distributions after 1-(13) C-pyruvate supplementation were used to trace metabolic rearrangements in different scenarios of asparagine and serine availability. The absence of asparagine in the medium caused growth arrest, and was associated with a dramatic increase in pyruvate uptake, a higher ratio of pyruvate carboxylation to dehydrogenation and an inability for de novo asparagine synthesis. The release of ammonia and amino acids such as aspartate, glutamate, and alanine were deeply impacted. This confirms asparagine to be essential for these GS-CHO cells as the main source of intracellular nitrogen as well as having an important anaplerotic role in TCA cycle activity. In turn, serine unavailability also negatively affected culture growth while triggering its de novo synthesis, confirmed by label incorporation coming from pyruvate, and reduced glycine and formate secretion congruent with its role as a precursor in the metabolism of one-carbon units. Overall, these results unfold important insights into GS-CHO cells metabolism that lay a clearer basis for fine-tuning bioprocess optimization.

784 ||| 18051290 ||| (5, 0.47) ||| 5 ||| 0 ||| Differences in optimal pH and temperature for cell growth and antibody production between two Chinese hamster ovary clones derived from the same parental clone. To investigate clonal variations of recombinant Chinese hamster ovary (rCHO) clones in response to culture pH and temperature, serum-free suspension cultures of two antibody-producing CHO clones (clones A and B), which were isolated from the same parental clone by the limiting dilution method, were performed in a bioreactor at pH values in the range of 6.8-7.6, and two different temperatures, 33 degrees C and 37 degrees C. In regard to cell growth, clone A and clone B displayed similar responses to temperature, although their degree of response differed. In contrast, clones A and B displayed different responses to temperature in regard to antibody production. In the case of clone A, no significant increase in maximum antibody concentration was achieved by lowering the culture temperature. The maximum antibody concentration obtained at 33 degrees C (pH 7.4) and 37 degrees C (pH 7.0) were 82.0 +/- 2.6 and 73.2 +/- 4.1 microg/ml, respectively. On the other hand, in the case of clone B, an approximately 2.5-fold increase in maximum antibody concentration was achieved by lowering the culture temperature. The enhanced maximum antibody concentration of clone B at 33 degrees C (132.6 +/- 14.9 microg/ml at pH 7.2) was due to not only enhanced specific antibody productivity but also to prolonged culture longevity. At 33 degrees C, the culture longevity of clone A also improved, but not as much as that of clone B. Taken together, CHO clones derived from the same parental clone displayed quite different responses to culture temperature and pH with regards antibody production, suggesting that environmental parameters such as temperature and pH should be optimized for each CHO clone.

785 ||| 11461779 ||| (5, 0.47) ||| 5 ||| 0 ||| Hypothermia enhances bcl-2 expression and protects against oxidative stress-induced cell death in Chinese hamster ovary cells. Oxidative stress is one of the major causes of cellular injury. Various reactive oxygen (ROS) and nitrogen (RNS) species such as superoxide, hydroxyl radical, peroxynitrite, and nitric oxide are involved in the manifestations of different types of organ toxicity and the resultant syndromes, symptoms, or diseases. Hypothermic conditions have been reported to reduce the oxidative stress in various in vitro and in vivo studies. In the present study, we sought to determine the effect of lowered temperatures on oxidative stress-induced cell death in Chinese hamster ovary (CHO) cells. We also investigated the oxidative stress-induced alterations in the expression of anti-apoptotic protein, bcl-2, in CHO cells at lowered temperatures. CHO cells were incubated at four different temperatures of 30, 32, 35, and 37 degrees C (control temperature) from 1 to 4 d. In another set, the cells were incubated with 100 microM hydrogen peroxide (H(2)O(2)) for 30 min before harvesting at different time points. The cells were harvested at 1, 2, 3, and 4 d. Cell survival was significantly higher at 30 degrees C as compared to 37 degrees C over 4 d of incubation. In cells incubated with H(2)O(2), significantly higher cell viability was observed at lower temperatures as compared to the cells incubated at 37 degrees C. The activity of glutathione peroxidase (GSH-Px) also increased significantly at lower temperatures. Lowered temperature also provided a significant increase in the expression of anti-apoptotic protein, bcl-2 after 4 d of incubation. These data suggest that hypothermic conditions lowers the risk of oxidative stress-induced cellular damage and programmed cell death by increasing the activity of GSH-Px and by the induction in the expression of the anti-apoptotic protein, bcl-2.

786 ||| 8554348 ||| (5, 0.46) ||| 5 ||| 0 ||| Correlation between glutathione and stimulation of the pentose phosphate cycle in situ in Chinese hamster ovary cells exposed to hydrogen peroxide. The effect of glutathione on stimulation of pentose phosphate cycle activity during oxidative challenge was evaluated in intact Chinese hamster ovary cells in situ. Glutathione was depleted to varying levels with L-buthionine-[S,R] sulfoximine. The level of stimulation of pentose phosphate cycle activity by exogenous H2O2 (4 mumol/10(7) cells) was dependent on the time of pretreatment with L-buthionine-[S,R] sulfoximine and was proportional to the total glutathione concentration. This was not related to the amount of GSSG, since its level was exceedingly low under conditions where H2O2 stimulated pentose phosphate cycle activity. The amount of GSSG in cells increased after exposure to 10-fold higher concentrations of H2O2 under conditions where there was no stimulation of pentose phosphate cycle activity above the basal level. Paraquat caused stimulation of pentose phosphate cycle activity which was independent of L-buthionine-[S,R] sulfoximine pretreatment and of the glutathione content of cells. The stimulatory effects of both oxidants on pentose phosphate cycle activity appeared to be independent of glutathione reductase activity since they were unaffected in cells treated with 1,3-bis(2-chloroethyl)-1-nitrosourea. The inhibitory effect of L-buthionine-[S,R] sulfoximine on stimulation of pentose phosphate cycle activity by H2O2 did not appear to be due to the inhibitor itself, but rather to the overall level of glutathione. Glutathione could have a role in maintaining activity of the pentose phosphate cycle at a level which is appropriate for the severity of the oxidative challenge as well as for the capacity of the cellular antioxidant defenses.

787 ||| 8672083 ||| (5, 0.45),(9, 0.38) ||| 5 ||| 0 ||| Effects of hyperthermia on intracellular chloride. Hyperthermia induces transient changes in [Na+]i and [K+]i in mammalian cells. Since Cl- flux is coupled with Na+ and K+ in several processes, including cell volume control, we have measured the effects of heat on [Cl-]i using the chloride indicator, MQAE, with flow cytometry. The mean basal level of [Cl-]i in Chinese hamster ovary cells was 12 mM. Cells heated at 42.0 degrees or 45.0 degrees C for 30 min had about a 2.5-fold increase in [Cl-]i above unheated control values when measured immediately after heating. There was about a 3-fold decrease in [Na+]i under the same conditions, as measured by Sodium Green. The magnitude of the increase in [Cl-]i depended upon time and temperature. The [Cl-]i recovered in a time-dependent fashion to control values by 30 min after heating. When cells were heated at 45.0 degrees C for 30 min in the presence of 1.5 mM furosemide, the heat-induced [Cl-]i increase was completely blocked. Since furosemide inhibits the Na+/K+/2Cl- cotransporter, Cl- channels, and even Cl-HCO3 exchange, these ion transporters may be involved in the heat-induced increase in [Cl-]i.

788 ||| 24039207 ||| (5, 0.45),(7, 0.45) ||| 5 ||| 0 ||| Effect of Bcl-x(L) overexpression on lactate metabolism in chinese hamster ovary cells producing antibody. Previously, overexpression of anti-apoptotic proteins, such as E1B-19K and Aven, was reported to alter lactate metabolism of CHO cells in culture. To investigate the effect of Bcl-xL , a well-known anti-apoptotic protein, on lactate metabolism of recombinant CHO (rCHO) cells, two antibody-producing rCHO cell lines with regulated Bcl-xL overexpression (CS13\*-0.02-off-Bcl-xL and CS13\*-1.00-off-Bcl-xL ) were established using the Tet-off system. When cells were cultivated without Bcl-xL overexpression, the specific lactate production rate (qLac ) of CS13\*-0.02-off-Bcl-xL and CS13\*-1.00-off-Bcl-xL were 7.32 +- 0.37 and 6.78 +- 0.56 pmol/cell/day, respectively. Bcl-xL overexpression, in the absence of doxycycline, did not affect the qLac of either cell line, though it enhanced the viability during cultures. Furthermore, activities of the enzymes related to glucose and lactate metabolism, such as hexokinase, glucose-6-phosphate dehydrogenase, lactate dehydrogenases, and alanine aminotransferase, were not affected by Bcl-xL overexpression either. Taken together, Bcl-xL overexpression showed no significant effect on the lactate metabolism of rCHO cells.

789 ||| 25797592 ||| (5, 0.45),(1, 0.44) ||| 5 ||| 0 ||| Metabolic markers associated with high mannose glycan levels of therapeutic recombinant monoclonal antibodies. High mannose (HM) glycan levels on secreted monoclonal antibodies can be influenced by external factors, including osmolality and copper deficiency, and by intrinsic factors determined by different cell lines. In order to identify the metabolic markers associated with HM glycan levels, metabolomics analysis was performed to assess the changes in the extracellular metabolites of recombinant cell lines at different time points during fed-batch production process. Ornithine was identified as the common metabolic marker influenced by both external and intrinsic factors when eight different medium conditions and eight different cell lines exhibiting different levels of HM were compared. A strong correlation was also observed between HM and mRNA expression levels of arginase 1, an enzyme that catalyzes the conversion of arginine to ornithine. The results from functional validation study showed that the supplementation of ornithine to the culture medium leads to an increased level of HM, while reduced concentration of spermine, a downstream product of ornithine metabolism, leads to a decreased level of HM. Additional metabolic markers correlating with HM glycan levels were identified from eight-cell line comparison analysis. A common feature shared by these identified markers is their previously described roles as contributors of cellular redox regulation.

790 ||| 25768021 ||| (5, 0.45),(1, 0.36) ||| 5 ||| 0 ||| Comparative metabolic analysis of CHO cell clones obtained through cell engineering, for IgG productivity, growth and cell longevity. Cell engineering has been used to improve animal cells' central carbon metabolism. Due to the central carbon metabolism's inefficiency and limiting input of carbons into the TCA cycle, key reactions belonging to these pathways have been targeted to improve cultures' performance. Previous works have shown the positive effects of overexpressing PYC2, MDH II and fructose transporter. Since each of these modifications was performed in different cell lines and culture conditions, no comparisons between these modifications can be made. In this work we aim at contrasting the effect of each of the modifications by comparing pools of transfected IgG producing CHO cells cultivated in batch cultures. Results of the culture performance of engineered clones indicate that even though all studied clones had a more efficient metabolism, not all of them showed the expected improvement on cell proliferation and/or specific productivity. CHO cells overexpressing PYC2 were able to improve their exponential growth rate but IgG synthesis was decreased, MDH II overexpression lead to a reduction in cell growth and protein production, and cells transfected with the fructose transporter gene were able to increase cell density and reach the same volumetric protein production as parental CHO cells in glucose. We propose that a redox unbalance caused by the new metabolic flux distribution could affect IgG assembly and protein secretion. In addition to reaction dynamics, thermodynamic aspects of metabolism are also discussed to further understand the effect of these modifications over central carbon metabolism.

791 ||| 24362913 ||| (5, 0.45),(1, 0.35) ||| 5 ||| 0 ||| Dynamics of growth and metabolism controlled by glutamine availability in Chinese hamster ovary cells. The physiology of animal cells is characterized by constantly changing environmental conditions and adapting cellular responses. Applied dynamic metabolic flux analysis captures metabolic dynamics and can be applied to industrially relevant cultivation conditions. We investigated the impact of glutamine availability or limitation on the physiology of CHO K1 cells in eight different batch and fed-batch cultivations. Varying glutamine availability resulted in global metabolic changes. We observed dose-dependent effects of glutamine in batch cultivation. Identifying metabolic links from the glutamine metabolism to specific metabolic pathways, we show that glutamine feeding results in its coupling to tricarboxylic acid cycle fluxes and in its decoupling from metabolic waste production. We provide a mechanistic explanation of the cellular responses upon mild or severe glutamine limitation and ammonia stress. The growth rate of CHO K1 decreased with increasing ammonia levels in the supernatant. On the other hand, growth, especially culture longevity, was stimulated at mild glutamine-limiting conditions. Flux rearrangements in the pyruvate and amino acid metabolism compensate glutamine limitation by consumption of alternative carbon sources and facilitating glutamine synthesis and mitigate ammonia stress as result of glutamine abundance.

792 ||| 9766522 ||| (5, 0.45) ||| 5 ||| 0 ||| Ammonia inhibits neural cell adhesion molecule polysialylation in Chinese hamster ovary and small cell lung cancer cells. Ammonia is a major concern in biotechnology because it often limits recombinant protein production by animal cells. Conditions, such as ammonia accumulation, in large-scale production systems can parallel those that develop within fast-growing solid tumors such as small cell lung cancer (SCLC). Ammonia's specific inhibition of the sialylation of secreted glycoproteins is well documented, but it is not known how ammonia affects membrane-bound proteins, nor what role it may have on important glycosylation determinants in cancer. We therefore examined the effects of NH4Cl on polysialic acid (PolySia) in the neural cell adhesion molecule (NCAM). By using flow cytometry combined with two NCAM antibodies, one specific for the peptide backbone and another that recognizes PolySia chains, we show that ammonia causes rapid, dose-dependent, and reversible inhibition of NCAM polysialylation in Chinese hamster ovary (CHO) and SCLC NCI-N417 cells. The decrease in PolySia was accompanied by a small increase in NCAM, suggesting that the changes were specific to the oligosaccharide. Inhibition by ammonia was greater for CHO cells, with PolySia cell surface content decreasing to 10% of control after a 4-day culture with 10 mM NH4Cl, while N417 cell PolySia was reduced by only 35%. Ammonia caused a 60% decrease in the CHO cell yield from glucose, while N417 cells were barely affected, suggesting that increased resistance to ammonia by N41 7 cells is a global rather than glycosylation-specific phenomenon. The data presented show that the tumor microenvironment may be an important factor in the regulation of PolySia expression.

793 ||| 24286971 ||| (5, 0.44) ||| 5 ||| 0 ||| The availability of glucose to CHO cells affects the intracellular lipid-linked oligosaccharide distribution, site occupancy and the N-glycosylation profile of a monoclonal antibody. The glycosylation pattern of a chimeric heavy chain antibody (EG2) produced from CHO cells was affected by the glucose concentration (0-25mM) of cultures established at high density (>10(6)ml(-1)) over 24h. The resulting proportion of non-glycosylated Mab was directly correlated to the exposure time of cells to media depleted of glucose. Deprivation of glucose for the full 24h resulted in a 45% non-glycosylated Mab fraction. Analysis of steady state levels of intracellular lipid-linked oligosaccharides (LLOs) showed that under glucose limitation there was a reduction in the amount of full length LLO (Glc3Man9GlcNac2), with a concomitant increase in the smaller mannosyl-glycans (Man2-5GlcNAc2). Glycan microheterogeneity was quantified by galactosylation and sialylation indices (GI and SI) which showed a direct correlation to the cell specific glucose uptake. The GI increased to 0.83 following media supplementation with a cocktail of uridine, manganese and galactose. This is significantly higher than for a fully humanized antibody (DP12) produced under the similar conditions or for similar antibodies reported in the literature. The high GI of the chimeric antibody (EG2) may be due to its low molecular weight and unusual structure. These findings are important in relation to the low substrate that may occur in fed-batch cultures for Mab production.

794 ||| 21626724 ||| (5, 0.42),(8, 0.37) ||| 5 ||| 0 ||| Estimating cell specific oxygen uptake and carbon dioxide production rates for mammalian cells in perfusion culture. We present robust methods for online estimation of cell specific oxygen uptake and carbon dioxide production rates (q(O2) and q(CO2), respectively) during perfusion cultivation of mammalian cells. Perfusion system gas and liquid phase mass balance expressions for oxygen and carbon dioxide were used to estimate q(O2), q(CO2) and the respiratory quotient (RQ) for Chinese hamster ovary (CHO) cells in perfusion culture over 12 steady states with varying dissolved oxygen (DO), pH, and temperature set points. Under standard conditions (DO = 50%, pH = 6.8, T = 36.5 C), q(O2) and q(CO2) ranges were 5.14-5.77 and 5.31-6.36 pmol/cell day, respectively, resulting in RQ values of 0.98-1.14. Changes to DO had a slight reducing effect on respiration rates with q(O2) and q(CO2) values of 4.64 and 5.47, respectively, at DO = 20% and 4.57 and 5.12 at DO = 100%. Respiration rates were lower at low pH with q(O2) and q(CO2) values of 4.07 and 4.15 pmol/cell day at pH = 6.6 and 4.98 and 5.36 pmol/cell day at pH = 7. Temperature also impacted respiration rates with respective q(O2) and q(CO2) values of 3.97 and 4.02 pmol/cell day at 30.5 C and 5.53 and 6.25 pmol/cell day at 37.5 C. Despite these changes in q(O2) and q(CO2) values, the RQ values in this study ranged from 0.98 to 1.23 suggesting that RQ was close to unity. Real-time q(O2) and q(CO2) estimates obtained using the approach presented in this study provide additional quantitative information on cell physiology both during bioprocess development and commercial biotherapeutic manufacturing.

795 ||| 10940860 ||| (5, 0.42),(6, 0.35) ||| 5 ||| 0 ||| Multiple cell culture factors can affect the glycosylation of Asn-184 in CHO-produced tissue-type plasminogen activator. Human tissue-type plasminogen activator (t-PA) contains a variably occupied glycosylation site at Asn-184 in naturally produced t-PA and in t-PA produced in recombinant Chinese hamster ovary (CHO) cells. The presence of an oligosaccharide at this site has previously been shown to reduce specific activity and fibrin binding. In this report, the site occupancy of t-PA is shown to increase gradually over the course of batch and fed-batch CHO cultures. Additional cell culture factors, including butyrate and temperature, are also shown to influence the degree of glycosylation. In each of these cases, conditions with decreased growth rate correlate with increased site occupancy. Investigations using quinidine and thymidine to manipulate the cell cycle distribution of cultures further support this correlation between site occupancy and growth state. Comparison of the cell cycle distribution across the range of cell culture factors investigated shows a consistent relationship between site occupancy and the fraction of cells in the G(0)/G(1) phase of the cell cycle. These results support a correlation between growth state and site occupancy, which fundamentally differs from site occupancy trends previously observed and illustrates the importance of the growth profile of CHO cultures in producing consistently glycosylated recombinant glycoproteins.

796 ||| 14705000 ||| (5, 0.42),(6, 0.30) ||| 5 ||| 0 ||| Maximizing interferon-gamma production by Chinese hamster ovary cells through temperature shift optimization: experimental and modeling. The Chinese hamster ovary (CHO) cell line producing interferon-gamma (IFN-gamma) exhibits a 2-fold increase in specific productivity when grown at 32 degrees C compared to 37 degrees C. Low temperature also causes growth arrest, meaning that the cell density is significantly lower at 32 degrees C, nutrients are consumed at a slower rate and the batch culture can be run for a longer period of time prior to the onset of cell death. At the end of the batch, product concentration is doubled at the low temperature. However, the batch time is nearly doubled as well, and this causes volumetric productivity to only marginally improve by using low temperature. One approach to alleviate the problem of slow growth at low temperature is to utilize a biphasic process, wherein cells are cultured at 37 degrees C for a period of time in order to obtain reasonably high cell density and then the temperature is shifted to 32 degrees C to achieve high specific productivity. Using this approach, it is hypothesized that IFN-gamma volumetric productivity would be maximized. We developed and validated a model for predicting the optimal point in time at which to shift the culture temperature from 37 degrees C to 32 degrees C. It was found that by shifting the temperature after 3 days of growth, the IFN-gamma volumetric productivity is increased by 40% compared to growth and production at 32 degrees C and by 90% compared to 37 degrees C, without any decrease in total production relative to culturing at 32 degrees C alone. The modeling framework presented here is applicable for optimizing controlled proliferation processes in general.

797 ||| 24692242 ||| (5, 0.41),(1, 0.41) ||| 5 ||| 0 ||| Control of galactosylated glycoforms distribution in cell culture system. Cell culture process conditions including media components and bioreactor operation conditions have a profound impact on recombinant protein quality attributes. Considerable changes in the distribution of galactosylated glycoforms (G0F, G1F, and G2F) were observed across multiple CHO derived recombinant proteins in development at Eli Lilly and Company when switching to a new chemically defined (CD) media platform condition. In the new CD platform, significantly lower G0F percentages and higher G1F and G2F were observed. These changes were of interest as glycosylation heterogeneity can impact the effectiveness of a protein. A systematic investigation was done to understand the root cause of the change and control strategy for galactosylated glycoforms distribution. It was found that changes in asparagine concentration could result in a corresponding change in G0F, G1F, and G2F distribution. A follow-up study examined a wider range of asparagine concentration and it was found that G0F, G1F, and G2F percentage could be titrated by adjusting asparagine concentration. The observed changes in heterogeneity from changing asparagine concentration are due to resulting changes in ammonium metabolism. Further study ascertained that different integrated ammonium level during the cell culture process could control G0F, G1F, and G2F percentage distribution. A mechanism hypothesis is proposed that integrated ammonium level impacts intracellular pH, which further regulates beta-1, 4 galactosyltransferase activity.

798 ||| 25014175 ||| (5, 0.41),(1, 0.37) ||| 5 ||| 0 ||| The impact of anti-apoptotic gene Bcl-2 expression on CHO central metabolism. Anti-apoptosis engineering is an established technique to prolong the viability of mammalian cell cultures used for industrial production of recombinant proteins. However, the effect of overexpressing anti-apoptotic proteins on central carbon metabolism has not been systematically studied. We transfected CHO-S cells to express Bcl-2 , an engineered anti-apoptotic gene, and selected clones that differed in their Bcl-2 expression and caspase activity. (13)C metabolic flux analysis (MFA) was then applied to elucidate the metabolic alterations induced by Bcl-2 . Expression of Bcl-2Delta reduced lactate accumulation by redirecting the fate of intracellular pyruvate toward mitochondrial oxidation during the lactate-producing phase, and it significantly increased lactate re-uptake during the lactate-consuming phase. This flux redistribution was associated with significant increases in biomass yield, peak viable cell density (VCD), and integrated VCD. Additionally, Bcl-2 expression was associated with significant increases in isocitrate dehydrogenase and NADH oxidase activities, both rate-controlling mitochondrial enzymes. This is the first comprehensive (13)C MFA study to demonstrate that expression of anti-apoptotic genes has a significant impact on intracellular metabolic fluxes, especially in controlling the fate of pyruvate carbon, which has important biotechnology applications for reducing lactate accumulation and enhancing productivity in mammalian cell cultures.

799 ||| 17239640 ||| (5, 0.41) ||| 5 ||| 0 ||| Down-regulation of cold-inducible RNA-binding protein does not improve hypothermic growth of Chinese hamster ovary cells producing erythropoietin. Discovery of the cold-inducible RNA-binding protein (CIRP) in mouse fibroblasts suggests that growth suppression at hypothermic conditions is due to an active response by the cell rather than due to passive thermal effects. To determine the effect of down-regulated CIRP expression on cell growth and erythropoietin (EPO) production in recombinant Chinese hamster ovary (rCHO) cells at low culture temperature, stable CHO cell clones with reduced CIRP expression level were established by transfecting (rCHO) cells with the CIRP siRNA vector with a target sequence of TCGTCCTTCCATGGCTGTA. For comparison of the degree of specific growth rate (micro) reduction at low culture temperature, three CIRP-reduced clones with different mu and three control clones transfected with null vector were cultivated at two different temperatures, 32 degrees C and 37 degrees C. Unlike mouse fibroblasts, alleviation of hypothermic growth arrest of rCHO cells by CIRP down-regulation was insignificant, as shown by statistical analysis using the t-test (P<0.18, n=3). The ratios of mu at 32 degrees C to micro at 37 degrees C of CIRP-reduced clones and control clones were 0.29+/-0.03 and 0.25+/-0.03 on an average, respectively. Furthermore, it was also found that overexpression of CIRP did not inhibit rCHO cell growth significantly at 37 degrees C. Taken together, the data obtained show that down-regulation of only CIRP in rCHO cells, unlike mouse fibroblasts, is not sufficient to recover growth arrest at low-temperature culture (32 degrees C).

800 ||| 10099545 ||| (5, 0.40),(6, 0.37) ||| 5 ||| 0 ||| Metabolic effects on recombinant interferon-gamma glycosylation in continuous culture of Chinese hamster ovary cells. Asparagine linked (N-linked) glycosylation is an important modification of recombinant proteins, because the attached oligosaccharide chains can significantly alter protein properties. Potential glycosylation sites are not always occupied with oligosaccharide, and site occupancy can change with the culture environment. To investigate the relationship between metabolism and glycosylation site occupancy, we studied the glycosylation of recombinant human interferon-gamma (IFN-gamma) produced in continuous culture of Chinese hamster ovary cells. Intracellular nucleotide sugar levels and IFN-gamma glycosylation were measured at different steady states which were characterized by central carbon metabolic fluxes estimated by material balances and extracellular metabolite rate measurements. Although site occupancy varied over a rather narrow range, we found that differences correlated with the intracellular pool of UDP-N-acetylglucosamine + UDP-N-acetylgalactosamine (UDP-GNAc). Measured nucleotide levels and estimates of central carbon metabolic fluxes point to UTP depletion as the cause of decreased UDP-GNAc during glucose limitation. Glucose limited cells preferentially utilized available carbon for energy production, causing reduced nucleotide biosynthesis. Lower nucleoside triphosphate pools in turn led to lower nucleotide sugar pools and reduced glycosylation site occupancy. Subsequent experiments in batch and fed-batch culture have confirmed that UDP-sugar concentrations are correlated with UTP levels in the absence of glutamine limitation. Glutamine limitation appears to influence glycosylation by reducing amino sugar formation and hence UDP-GNAc concentration. The influence of nucleotide sugars on site occupancy may only be important during periods of extreme starvation, since relatively large changes in nucleotide sugar pools led to only minor changes in glycosylation.

801 ||| 10876158 ||| (5, 0.40),(4, 0.37) ||| 5 ||| 0 ||| The rate of cell growth is regulated by purine biosynthesis via ATP production and G(1) to S phase transition. We recently showed that an increased supply of purine nucleotides increased the growth rate of cultured fibroblasts. To understand the mechanism of the growth rate regulation, CHO K1 (a wild type of Chinese hamster ovary fibroblast cell line) and CHO ade (-)A (a cell line deficient in amidophosphoribosyltransferase, a rate-limiting enzyme of the de novo pathway) were cultured under various conditions. Moreover, a defective de novo pathway in CHO ade (-)A cells was exogenously restored by 5-amino-4-imidazole-carboxamide riboside, a precursor of the de novo pathway. The following parameters were determined: the growth rate of CHO fibroblasts, the metabolic rate of the de novo pathway, the enzyme activities of amidophosphoribosyltransferase and hypoxanthine phosphoribosyltransferase, the content of intracellular nucleotides, and the duration of each cell-cycle phase. We concluded the following: (i) Purine de novo synthesis, rather than purine salvage synthesis or pyrimidine synthesis, limits the growth rate. (ii) Purine nucleotides are synthesized preferentially by the salvage pathway as long as hypoxanthine is available for energy conservation. (iii) The GTP content depends on the intracellular ATP content. (iv) Biosynthesis of purine nucleotides increases the growth rate mainly through ATP production and promotion of the G(1)/S transition.

802 ||| 15575699 ||| (5, 0.40),(2, 0.37) ||| 5 ||| 0 ||| Enhancing effect of low culture temperature on specific antibody productivity of recombinant Chinese hamster ovary cells: clonal variation. To understand the different responses of recombinant Chinese hamster ovary (rCHO) cells to low culture temperature regarding specific productivity (q), 12 parental clones and their corresponding amplified clones producing a humanized antibody were cultivated at 32 and 37 degrees C. The specific growth rate of all clones, including both parental and amplified clones, decreased by 30-63% at 32 degrees C, compared to rates at 37 degrees C. In contrast, their specific antibody productivity (qAb) was significantly enhanced at 32 degrees C. Furthermore, the degree of qAb enhancement at 32 degrees C varied a lot from 4- to 25-fold among the parental clones. At 32 degrees C, most of the amplified clones, regardless of methotrexate (MTX) levels, also showed enhanced qAb but to a lesser extent than their parental clones. However, clone 14 amplified at 0.32 microM MTX (clone 14-0.32) and clone 20 amplified at 1 microM MTX (clone 20-1.00), unlike their parental clones, did not show enhanced qAb at 32 degrees C. Thus, it was found that the enhancing effect of low culture temperature on q of rCHO cells depends on clones. Taken together, the results obtained here emphasize the importance of clonal selection for the successful application of low culture temperature to the enhanced foreign protein production in rCHO cells.

803 ||| 19194899 ||| (5, 0.39),(6, 0.35) ||| 5 ||| 0 ||| CHO cells adapted to hypothermic growth produce high yields of recombinant beta-interferon. Mild hypothermic conditions (30-33 degrees C) have previously been shown to increase cell-specific productivity (Q(p)) of recombinant proteins from mammalian cells. However, this is often associated with a lower growth rate which off-sets any potential advantage of higher product titers. We report the isolation of a population of Chinese Hamster Ovary (CHO) cells that have been adapted to low-temperature growth by continuous subculture at low temperature for up to 300 days. This adapted cell population achieved a growth rate twofold greater than nonadapted cells under low-temperature conditions (32 degrees C) while maintaining an elevated level of cell-specific expression of recombinant beta-interferon. The volumetric titer of beta-interferon was enhanced by 70% in stationary cultures and by more than twofold by application of a temperature-shift strategy involving a growth to production phase. However, the low-temperature-adapted cells were fragile and demonstrated an increased sensitivity to hydrodynamic stress in agitated cultures. This problem was resolved by the use of macroporous microcarriers which protected the cells and allowed growth of high-density cultures under hypothermic conditions. This eventually resulted in a threefold enhancement of volumetric titer of monomeric beta-interferon compared to the original control culture at 37 degrees C.

804 ||| 7727525 ||| (5, 0.39),(4, 0.34) ||| 5 ||| 0 ||| Direct measurement of the accumulation and mitochondrial conversion of nitric oxide within Chinese hamster ovary cells using an intracellular electron paramagnetic resonance technique. We have developed an electron paramagnetic resonance (EPR) method for the nondestructive detection and quantification of intracellular NO in real time. Based upon this technique, we have obtained evidence for the metabolism of this bioregulatory molecule by mitochondria. Line-broadening of the EPR signal of a coal derivative, fusinite, was calibrated as a function of NO concentration in aqueous solution. The methodology was validated using two compounds which release NO in a controlled and predictable manner with first-order rate constants k1 = 5.0 x 0.10(-3) s-1 and k'1 = 3.4 x 10(-4) s-1 (35 degrees C). Fusinite was internalized in Chinese hamster ovary cells (CHO) by phagocytosis, after which the cells were allowed to consume the available O2, producing an hypoxic environment. The NO released from one of the NO donors, added to the culture fluid at an initial concentration of 50 microM, was directly measured in the intracellular environment as line-broadening of the fusinite EPR signal. The linewidth diminished with time, indicating that NO was being converted to a non-paramagnetic species by the cells with an apparent zero-order rate constant of 5 x 10(8) NO molecules cell-1 min-1 (20 degrees C). Addition of cyanide to the culture medium (5 mM final concentration) inhibited this disappearance of NO. NO also was converted in the presence of isolated mitochondria in the absence of oxygen. These observations suggest that under hypoxic conditions, there exists in CHO cells a metabolic pathway for the conversion of NO to diamagnetic species, which involves interactions with mitochondria.

805 ||| 24632968 ||| (5, 0.39) ||| 5 ||| 0 ||| Analyzing clonal variation of monoclonal antibody-producing CHO cell lines using an in silico metabolomic platform. Monoclonal antibody producing Chinese hamster ovary (CHO) cells have been shown to undergo metabolic changes when engineered to produce high titers of recombinant proteins. In this work, we have studied the distinct metabolism of CHO cell clones harboring an efficient inducible expression system, based on the cumate gene switch, and displaying different expression levels, high and low productivities, compared to that of the parental cells from which they were derived. A kinetic model for CHO cell metabolism was further developed to include metabolic regulation. Model calibration was performed using intracellular and extracellular metabolite profiles obtained from shake flask batch cultures. Model simulations of intracellular fluxes and ratios known as biomarkers revealed significant changes correlated with clonal variation but not to the recombinant protein expression level. Metabolic flux distribution mostly differs in the reactions involving pyruvate metabolism, with an increased net flux of pyruvate into the tricarboxylic acid (TCA) cycle in the high-producer clone, either being induced or non-induced with cumate. More specifically, CHO cell metabolism in this clone was characterized by an efficient utilization of glucose and a high pyruvate dehydrogenase flux. Moreover, the high-producer clone shows a high rate of anaplerosis from pyruvate to oxaloacetate, through pyruvate carboxylase and from glutamate to alpha-ketoglutarate, through glutamate dehydrogenase, and a reduced rate of cataplerosis from malate to pyruvate, through malic enzyme. Indeed, the increase of flux through pyruvate carboxylase was not driven by an increased anabolic demand. It is in fact linked to an increase of the TCA cycle global flux, which allows better regulation of higher redox and more efficient metabolic states. To the best of our knowledge, this is the first time a dynamic in silico platform is proposed to analyze and compare the metabolomic behavior of different CHO clones.

806 ||| 11257601 ||| (5, 0.39) ||| 5 ||| 0 ||| Metabolic control of recombinant protein N-glycan processing in NS0 and CHO cells. Chinese hamster ovary and murine myeloma NS0 cells are currently favored host cell types for the production of therapeutic recombinant proteins. In this study, we compared N-glycan processing in GS-NS0 and GS-CHO cells producing the same model recombinant glycoprotein, tissue inhibitor of metalloproteinases 1. By manipulation of intracellular nucleotide-sugar content, we examined the feasibility of implementing metabolic control strategies aimed at reducing the occurrence of murine-specific glycan motifs on NS0-derived recombinant proteins, such as Galalpha1,3Galbeta1,4GlcNAc. Although both CHO and NS0-derived oligosaccharides were predominantly of the standard complex type with variable sialylation, 30% of N-glycan antennae associated with NS0-derived TIMP-1 terminated in alpha1,3-linked galactose residues. Furthermore, NS0 cells conferred a greater proportion of terminal N-glycolylneuraminic (sialic) acid residues as compared with the N-acetylneuraminic acid variant. Inclusion of the nucleotide-sugar precursors, glucosamine (10 mM, plus 2 mM uridine) and N-acetylmannosamine (20 mM), in culture media were shown to significantly increase the intracellular pools of UDP-N-acetylhexosamine and CMP-sialic acid, respectively, in both NS0 and CHO cells. The elevated UDP-N-acetylhexosamine content induced by the glucosamine/uridine treatment was associated with an increase in the antennarity of N-glycans associated with TIMP-1 produced in CHO cells but not N-glycans associated with TIMP-1 from NS0 cells. In addition, elevated UDP-N-acetylhexosamine content was associated with a slight decrease in sialylation in both cell lines. The elevated CMP-sialic acid content induced by N-acetylmannosamine had no effect on the overall level of sialylation of TIMP-1 produced by both CHO and NS0 cells, although the ratio of N-glycolylneuraminic acid:N-acetylneuraminic acid associated with NS0-derived TIMP-1 changed from 1:1 to 1:2. These data suggest that manipulation of nucleotide-sugar metabolism can promote changes in N-glycan processing that are either conserved between NS0 and CHO cells or specific to either NS0 cells or CHO cells.

807 ||| 20363268 ||| (5, 0.38),(1, 0.35) ||| 5 ||| 0 ||| Metabolomics-driven approach for the improvement of Chinese hamster ovary cell growth: overexpression of malate dehydrogenase II. We have established a liquid chromatography-mass spectrometry based metabolomics platform to identify extracellular metabolites in the medium of recombinant Chinese hamster ovary (CHO) fed-batch reactor cultures. Amongst the extracellular metabolites identified, malate accumulation was the most significant. The contributing factors to malate efflux were found to be the supply of aspartate from the medium, and an enzymatic bottleneck at malate dehydrogenase II (MDH II) in the tricarboxylic acid cycle. Subsequent metabolic engineering to overexpress MDH II in CHO resulted in increases in intracellular ATP and NADH, and up to 1.9-fold improvement in integral viable cell number.

808 ||| 9328145 ||| (5, 0.35) ||| 5 ||| 0 ||| Menadione-resistant Chinese hamster ovary cells have an increased capacity for glutathione synthesis. A cell line (MRc40) resistant to the model quinone compound, menadione, has been isolated from a parental Chinese hamster ovary cell line (CHO-K1). The known relationship between menadione toxicity and glutathione (GSH) depletion led us to investigate whether the mechanism of resistance of MRc40 was related to alteration in GSH homeostasis. Intracellular concentrations of GSH and cysteine (CySH) were twofold and 3.2-fold greater in MRc40 than in CHO-K1. Following exposure to menadione, GSH and CySH were depleted, but subsequent recovery of thiols was more rapid and of greater magnitude in MRc40 than in CHO-K1. Twelve hours after exposure to menadione, the concentrations of GSH and CySH were 9.7- and 4.2-fold greater in MRc40 than in CHO-K1. Using nuclear magnetic resonance (NMR) spectroscopy, we observed the in situ removal of menadione from cell suspensions of CHO-K1 and MRc40. However, only in CHO-K1 did we observe concomitant depletion of NMR-visible GSH. We conclude that the perturbation of GSH metabolism contributes to the resistant phenotype and is an important characteristic of menadione-resistant CHO cells.

809 ||| 11745163 ||| (5, 0.34),(1, 0.32) ||| 5 ||| 0 ||| Decoupling cell growth and product formation in Chinese hamster ovary cells through metabolic control. The development of a strategy for the culture of Chinese hamster ovary (CHO) cells producing tissue plasminogen activator (t-PA) is investigated. This strategy is based on the replacement of the main carbon source, glucose, by another compound that is slowly metabolizable, particularly galactose. The introduction of this change allows for acute change in cell behavior at various levels. Cell growth is stopped after this nutrient shift, and the cells can be kept in long-duration culture at a low growth rate and high viability as compared with a culture strategy based solely on glucose utilization. Moreover, the capability of cells to produce recombinant proteins (t-PA in this work) can be maintained over the entire period of galactose feeding. From the metabolic point of view, use of a slowly metabolizable carbon source (galactose) introduces important changes in the production of lactate, ammonia, and some amino acids. The use of this metabolic shift enables the generation of biphasic processes, with a first phase with cell growth on glucose and a second stationary phase on galactose, which is particularly suited to perfusion systems.

810 ||| 8597537 ||| (5, 0.34) ||| 5 ||| 0 ||| Influence of targeted asparagine starvation on extra- and intracellular amino acid pools of cultivated Chinese hamster ovary cells. For the development of an expression system with an amino-acid-inducible promoter, the influence of extracellular stress, by starvation of the non-essential amino acid asparagine, on the extra- and intracellular amino acid pool was investigated. Therefore a widely used nontransformed CHO cell line was cultivated in a serum-free and optimized DMEM/F12 medium in repeated batch mode. During the last repeat the medium contained no asparagine. The cells could compensate totally for this lack by an increased conversion of aspartate, glutamate, asparagine, serine, glutamine and arginine, while almost the whole intracellular pool of amino acids decreased. By this enhanced metabolic activity the maximum growth rate rose from 0.8 day-1 in complete medium to 1.1 day-1 in asparagine-free medium. The exceptional increase in asparagine biosynthesis points to a strong activation of asparagine synthetase, the key enzyme within the asparagine biosynthesis pathway. The regulation mechanism for the asparagine synthetase at the transcription level had to be analysed further in detail and will lead to an asparagine-sensitive promotor. To investigate reaction cascades that influence the protein synthesis or the overall gene expression, one had to look carefully at intracellular amino acid levels, because of their importance for polypeptide synthesis and energy supply, but also because of their obvious sensitivity to extracellular stresses.

811 ||| 25220616 ||| (5, 0.33),(1, 0.33) ||| 5 ||| 0 ||| Amino acid and glucose metabolism in fed-batch CHO cell culture affects antibody production and glycosylation. Fed-batch Chinese hamster ovary (CHO) cell culture is the most commonly used process for IgG production in the biopharmaceutical industry. Amino acid and glucose consumption, cell growth, metabolism, antibody titer, and N-glycosylation patterns are always the major concerns during upstream process optimization, especially media optimization. Gaining knowledge on their interrelations could provide insight for obtaining higher immunoglobulin G (IgG) titer and better controlling glycosylation-related product quality. In this work, different fed-batch processes with two chemically defined proprietary media and feeds were studied using two IgG-producing cell lines. Our results indicate that the balance of glucose and amino acid concentration in the culture is important for cell growth, IgG titer and N-glycosylation. Accordingly, the ideal fate of glucose and amino acids in the culture could be mainly towards energy and recombinant product, respectively. Accumulation of by-products such as NH4(+) and lactate as a consequence of unbalanced nutrient supply to cell activities inhibits cell growth. The levels of Leu and Arg in the culture, which relate to cell growth and IgG productivity, need to be well controlled. Amino acids with the highest consumption rates correlate with the most abundant amino acids present in the produced IgG, and thus require sufficient availability during culture. Case-by-case analysis is necessary for understanding the effect of media and process optimization on glycosylation. We found that in certain cases the presence of Man5 glycan can be linked to limitation of UDP-GlcNAc biosynthesis as a result of insufficient extracellular Gln. However, under different culture conditions, high Man5 levels can also result from low alpha-1,3-mannosyl-glycoprotein 2-beta-N-acetylglucosaminyltransferase (GnTI) and UDP-GlcNAc transporter activities, which may be attributed to high level of NH4+ in the cell culture. Furthermore, galactosylation of the mAb Fc glycans was found to be limited by UDP-Gal biosynthesis, which was observed to be both cell line and cultivation condition-dependent. Extracellular glucose and glutamine concentrations and uptake rates were positively correlated with intracellular UDP-Gal availability. All these findings are important for optimization of fed-batch culture for improving IgG production and directing glycosylation quality.

812 ||| 24841241 ||| (5, 0.33),(1, 0.33) ||| 5 ||| 0 ||| Complete knockout of the lactate dehydrogenase A gene is lethal in pyruvate dehydrogenase kinase 1, 2, 3 down-regulated CHO cells. Accumulation of high level of lactate can negatively impact cell growth during fed-batch culture process. In this study, we attempted to knockout the lactate dehydrogenase A (LDHA) gene in CHO cells in order to attenuate the lactate level. To prevent the potential deleterious effect of pyruvate accumulation, consequent to LDHA knockout, on cell culture, we chose a pyruvate dehydrogenase kinase 1, 2, and 3 (PDHK1, 2, and 3) knockdown cell line in which to knock out LDHA alleles. Around 3,000 clones were screened to obtain 152 mutants. Only heterozygous mutants were identified. An attempt to knockout the remaining wild-type allele from one such heterozygote yielded only two mutants after screening 567 clones. One had an extra valine. Another evidenced a duplication event, possessing at lease one wild-type and two different frameshifted alleles. Both mutants still retained LDH activity. Together, our data strongly suggest that a complete knockout of LDHA is lethal in CHO cells, despite simultaneous down-regulation of PDHK1, 2, and 3.

813 ||| 21792592 ||| (5, 0.33) ||| 5 ||| 0 ||| Combinatorial engineering of ldh-a and bcl-2 for reducing lactate production and improving cell growth in dihydrofolate reductase-deficient Chinese hamster ovary cells. In Chinese hamster ovary (CHO) cells, rapid glucose metabolism normally leads to inefficient use of glucose, most of which is converted to lactate during cell cultures. Since lactate accumulation during the culture often exerts a negative effect on cell growth and valuable product formation, several genetic engineering approaches have been developed to suppress lactate dehydrogenase-A (LDH-A), the enzyme converting pyruvate into lactate. However, despite the reduced lactate accumulation, such cell cultures are eventually terminated in the late period of the culture, mainly due to apoptosis. Therefore, we developed an apoptosis-resistant, less lactate-producing dhfr(-) CHO cell line (CHO-Bcl2-LDHAsi) by overexpressing Bcl-2, one of the most well-known anti-apoptotic proteins, and by downregulating LDH-A in a dhfr(-) CHO cell line. When the dhfr(-) CHO-Bcl2-LDHAsi cell line was used as a host cell line for the development of recombinant CHO (rCHO) cells producing an Fc-fusion protein, the culture longevity of the rCHO cells was extended without any detrimental effect of genetic engineering on specific protein productivity. Simultaneously, the specific lactate production rate and apparent yield of lactate from glucose were reduced to 21-65% and 37-78% of the control cells, respectively. Taken together, these results show that the use of an apoptosis-resistant, less lactate-producing dhfr(-) CHO cell line as a host cell line saves the time and the effort of establishing an apoptosis-resistant, less lactate-producing rCHO cells for producing therapeutic proteins.

814 ||| 9756617 ||| (5, 0.31),(3, 0.31) ||| 5 ||| 0 ||| Alpha 1,6-linked fucose affects the expression and stability of polysialic acid-carrying glycoproteins in Chinese hamster ovary cells. To determine the effect of alpha1,6-linked fucose modification of N-glycans on the expression of polysialic acids (PSAs), the expression of PSAs in a fucose-lacking mutant of Chinese hamster ovary (CHO) cells, Lec13, was compared with that in CHO K1 cells. PSA synthase activity in these cells and the antennary structures of N-glycans associated with the neural adhesion molecule (NCAM), which is a major PSA-carrying glycoprotein, did not differ between the two types of cells. Metabolic labeling of cells with [3H]glucosamine for 48 h followed by immunoprecipitation with anti-PSA monoclonal antibodies revealed that the amount of labeled PSA-carrying glycoproteins obtained from Lec13 cells was 10-times less than that from K1 cells, although the incorporation of [3H]glucosamine into total extracts and NCAM was almost the same. In contrast, when cells were pulse labeled with [35S]methionine followed by a 1 h chase, there was not such a great difference in PSA-carrying protein synthesis between K1 and Lec13 cells. However, during a prolonged chase period, PSA-carrying proteins rapidly decreased in Lec13 cells, whereas those in K1 cells did not change. The degradation of PSA-carrying glycoproteins in Lec13 cells was partly prevented when the cells were grown in fucose-containing medium. Therefore, fucose modification of core N-glycans may affect the efficient expression of PSAs through the intracellular stability of PSA-carrying glycoproteins.

815 ||| 22359204 ||| (5, 0.30) ||| 5 ||| 0 ||| Apoptosis in CHO cell batch cultures: examination by flow cytometry. Chinese hamster ovary cells grown under conditions which are optimal for the production of a genetically engineered protein in batch culture, lose significant viability shortly after entering the stationary phase. This cell death was investigated morphologically and was found to be almost exclusively via apoptosi. Furthermore, cells were analyzed by flow cytometry using a fluorescent DNA end-labeling assay to label apoptotic cells, in conjunction with cell cycle analysis using propidium iodide. Apoptotic cells could be detected by this method, and by the radioactive end-labeling of extracted DNA, on all days of culture from day 1 to day 7; however, the degree of apoptotic cell death increased dramatically when the cells entered the stationary phase, rising to 50-60% of the total cell number at the termination of the culture. Flow cytometric analysis showed that the majority of cells underwent apoptosis whilst in G(1)/G(0) and formed an apoptotic population with high DNA FITC end-labeling and hypodiploid propidium iodide binding. Additionally, the ability or inability to secrete specific protein products did not appear to interfere with the development of the apoptotic population with time.

# ----------------------------- TOPIC6 -----------------------------

816 ||| 21509567 ||| (6, 0.99) ||| 6 ||| 7 ||| Effects of culture temperature and pH on flag-tagged COMP angiopoietin-1 (FCA1) production from recombinant CHO cells: FCA1 aggregation. To maximize the production of flag-tagged cartilage oligomeric matrix protein angiopoietin-1 (FCA1) from Chinese hamster ovary (CHO) cells, the effects of culture pH and temperature on cell growth and FCA1 production were investigated. Cells were cultivated in a bioreactor at different culture pH (6.7, 6.9, 7.2, and 7.5) and temperatures (33 and 37 C). Lowering the culture temperature suppressed cell growth while allowing maintenance of high cell viability for a longer culture period. The specific FCA1 productivity (q (FCA1)) was increased at low culture temperature. Accordingly, the highest FCA1 concentration was obtained at pH 7.2 and 33 C, and was approximately 4.0-fold higher than that at pH 7.2 and 37 C. However, aggregates and a monomeric form of FCA1, which are undesirable due to reduced biological activity or immunogenicity, were significant at pH 7.2 and 33 C. It was also found that the expression pattern of FCA1 was affected more significantly by culture pH than by the culture temperature. FCA1 aggregation dramatically decreased at culture pH 7.5 regardless of the culture temperature. Furthermore, the monomeric form of FCA1 was not observed. Taken together, optimization of culture temperature and culture pH (33 C and pH 7.5) significantly improves the production of biologically active FCA1 with tetrameric or pentameric forms from CHO cells.

817 ||| 20521303 ||| (6, 0.99) ||| 6 ||| 7 ||| Sialylation enhancement of CTLA4-Ig fusion protein in Chinese hamster ovary cells by dexamethasone. The importance of glycoprotein sialic acid levels is well known, as increased levels have been shown to increase in vivo serum half-life profiles. Here we demonstrate for the first time that dexamethasone (DEX) was capable of improving the sialylation of a CTLA4-Ig fusion protein produced by Chinese hamster ovary (CHO) cells. DEX was shown to enhance the intracellular addition of sialic acid by sialyltransferases as well as reduce extracellular removal of sialic acid by sialidase cleavage. We illustrated that DEX addition resulted in increased expression of the glycosyltransferases alpha2,3-sialyltransferase (alpha2,3-ST) and beta1,4-galactosyltransferase (beta1,4-GT) in CHO cells. Based upon our previous results showing DEX addition increased culture cell viability, we confirmed here that cultures treated with DEX also resulted in decreased sialidase activity. Addition of the glucocorticoid receptor (GR) antagonist mifepristone (RU-486) was capable of blocking the increase in sialylation by DEX which further supports that DEX affected sialylation as well as provides evidence that the sialylation enhancement effects of DEX on recombinant CHO cells occurred through the GR. Finally, the effects of DEX on increasing sialylation were then confirmed in 5-L controlled bioreactors. Addition of 1 microM DEX to the bioreactors on day 2 resulted in harvests with average increases of 16.2% for total sialic acid content and 15.8% in the protein fraction with N-linked sialylation. DEX was found to be a simple and effective method for increasing sialylation of this CTLA4-Ig fusion protein expressed in CHO cells.

818 ||| 18980186 ||| (6, 0.99) ||| 6 ||| 7 ||| Effect of culture temperature on erythropoietin production and glycosylation in a perfusion culture of recombinant CHO cells. To investigate the effect of culture temperature on erythropoietin (EPO) production and glycosylation in recombinant Chinese hamster ovary (CHO) cells, we cultivated CHO cells using a perfusion bioreactor. Cells were cultivated at 37 degrees C until viable cell concentration reached 1 x 10(7) cells/mL, and then culture temperature was shifted to 25 degrees C, 28 degrees C, 30 degrees C, 32 degrees C, 37 degrees C (control), respectively. Lowering culture temperature suppressed cell growth but was beneficial to maintain high cell viability for a longer period. In a control culture at 37 degrees C, cell viability gradually decreased and fell below 80% on day 18 while it remained over 90% throughout the culture at low culture temperature. The cumulative EPO production and specific EPO productivity, q(EPO), increased at low culture temperature and were the highest at 32 degrees C and 30 degrees C, respectively. Interestingly, the cumulative EPO production at culture temperature below 32 degrees C was not as high as the cumulative EPO production at 32 degrees C although the q(EPO) at culture temperature below 32 degrees C was comparable or even higher than the q(EPO) at 32 degrees C. This implies that the beneficial effect of lowering culture temperature below 32 degrees C on q(EPO) is outweighed by its detrimental effect on the integral of viable cells. The glycosylation of EPO was evaluated by isoelectric focusing, normal phase HPLC and anion exchange chromatography analyses. The quality of EPO at 32 degrees C in regard to acidic isoforms, antennary structures and sialylated N-linked glycans was comparable to that at 37 degrees C. However, at culture temperatures below 32 degrees C, the proportions of acidic isoforms, tetra-antennary structures and tetra-sialylated N-linked glycans were further reduced, suggesting that lowering culture temperature below 32 degrees C negatively affect the quality of EPO. Thus, taken together, cell culture at 32 degrees C turned out to be the most satisfactory since it showed the highest cumulative EPO production, and moreover, EPO quality at 32 degrees C was not deteriorated as obtained at 37 degrees C.

819 ||| 18623371 ||| (6, 0.99) ||| 6 ||| 7 ||| The effect of ammonia on the O-linked glycosylation of granulocyte colony-stimulating factor produced by chinese hamster ovary cells. Ammonium ion concentrations ranging from 0 to 10 mM are shown to significantly reduce the sialylation of granuiocyte colony-stimulating factor (G-CSF) produced by recombinant Chinese hamster ovary cells. Specifically, the degree of completion of the final reaction in the O-linked glycosylation pathway, the addition of sialic acid in an alpha(2,6) linkage to N-acetylgalactosamine, is reduced by NH(4) (+) concentrations of as low as 2 mM. The effect of ammonia on sialylation is rapid, sustained, and does not affect the secretion rate of G-CSF. Additionally, the effect can be mimicked using the weak base chloroquine, suggesting that the effect is related to the weak base characteristics of ammonia. In support of this hypothesis, experiments using brefeldin A suggest that the addition of sialic acid in an alpha(2,6) linkage to N-acetylgalactosamine occurs in the trans-Golgi compartment prior to the trans-Golgi network, which would be expected under normal conditions to have a slightly acidic pH in the range from 6.5 to 6.75. Ammonium ion concentrations of 10 mM would be expected to reduce significantly the differences in pH between acidic intracellular compartments and the cytoplasm. The pH-activity profile for the CHO O-linked alpha(2,6) sialytransferase using monosialylated G-CSF as a substrate reveals a twofold decrease in enzymatic activity across the pH range from 6.75 to 7.0.Mathematical modeling of this sialylation reaction supports the hypothesis that this twofold decrease in sialyltransferase activity resulting from an ammoniainduced increase in trans-Golgi pH could produce the observed decrease in G-CSF sialylation. (c) 1995 John Wiley & Sons, Inc.

820 ||| 15818560 ||| (6, 0.99) ||| 6 ||| 7 ||| Gene-expression profiles for five key glycosylation genes for galactose-fed CHO cells expressing recombinant IL-4/13 cytokine trap. Recombinant protein glycosylation profiles have been shown to affect the in-vivo half-life, and therefore the efficacy and economics, for many therapeutics. While much research has been conducted correlating the effects of various stimuli on recombinant protein glycosylation characteristics, relatively little work has examined glycosylation-related gene-expression profiles. In this study, the effects of galactose feeding on the gene-expression profiles for five key glycosylation-related genes were determined for Chinese hamster ovary cells producing a recombinant IL-4/13 cytokine trap fusion. The genes investigated were sialidase, a putative alpha2,3-sialyltransferase, CMP-sialic acid transporter, beta1,4-galactosyltransferase, and UDP-galactosyltransferase. Additionally, the sialic acid content (sialylation) of the recombinant protein was examined. The peak sialic acid content of the IL-4/13 cytokine trap fusion protein was observed to be similar for the control and galactose-fed cultures. The gene-expression profiles for four of the glycosylation genes were observed to be sensitive to the glucose concentration and not significantly different for the control and galactose-fed cultures prior to glucose depletion. However, the sialidase gene-expression profiles were different for the control and galactose-fed cultures. The sialidase gene-expression profile increased significantly for the galactose-fed cultures prior to glucose depletion, whereas for the control cultures, the sialidase gene-expression profiles did not increase until the late stationary phase. The intracellular sialidase enzyme activity decreased exponentially with time for the control cultures; however, for the galactose-fed cultures, the intracellular sialidase enzyme activity decreased initially and then remained relatively high compared to the control cultures. These results indicate that the galactose feeding may increase the potential for desialylation, which offsets any improvements in the sialylation rate due to increased substrate levels. Thus, galactose feeding is an unnecessary expense for the production of the IL-4/13 cytokine trap fusion protein in a batch process.

821 ||| 11753926 ||| (6, 0.87) ||| 6 ||| 7 ||| Erythropoietin production from CHO cells grown by continuous culture in a fluidized-bed bioreactor. A Chinese hamster ovary (CHO) cell line that expresses human erythropoietin (huEPO) was in a 2-L Cytopilot fluidized-bed bioreactor with 400 mL macroporous Cytoline-1 microcarriers and a variable perfusion rate of serum-free and protein-free medium for 48 days. The cell density increased to a maximum of 23 x 10(6) cells/mL, beads on day 27. The EPO concentration increased to 600 U/mL during the early part of the culture period (on day 24) and increased further to 980 U/mL following the addition of a higher concentration of glucose and the addition of sodium butyrate. The EPO concentration was significantly higher (at least 2x than that in a controlled stirred-tank bioreactor, in a spinner flask, or in a stationary T-flask culture. The EPO accumulated to a total production of 28,000 kUnits over the whole culture period. The molecular characteristics of EPO with respect to size and pattern of glycosylation did not change with scale up. The pattern of utilization and production of 18 amino acids was similar in the Cytopilot culture to that in a stationary batch culture in a T-flask. The concentration of ammonia was maintained at a low level (< 2 mM) over the entire culture period. The specific rate of consumption of glucose, as well as the specific rates of production of lactate and ammonia, were constant throughout the culture period indicating a consistent metabolic behavior of the cells in the bioreactor. These results indicate the potential of the Cytopilot bioreactor culture system for the continuous production of a recombinant protein over several weeks.

822 ||| 10099302 ||| (6, 0.85) ||| 6 ||| 7 ||| Improvement of interferon-gamma sialylation in Chinese hamster ovary cell culture by feeding of N-acetylmannosamine. Because the presence of sialic acid can extend circulatory lifetime, a high degree of sialylation is often a desirable feature of therapeutic glycoproteins. In this study, the incomplete intracellular sialylation of interferon-gamma (IFN-gamma), produced by Chinese hamster ovary cell culture, was minimized by supplementing the culture medium with N-acetylmannosamine (ManNAc), a direct intracellular precursor for sialic acid synthesis. By introducing 20 mM ManNAc into the culture medium, incompletely sialylated biantennary glycan structures were reduced from 35% to 20% at the Asn97 glycosylation site. This effect was achieved without affecting cell growth or product yield. The intracellular pool of CMP-sialic acid, the nucleotide sugar substrate for sialyltransferase, was also extracted and quantified by HPLC. Feeding of 20 mM ManNAc increased this intracellular pool of CMP-sialic acid by nearly thirtyfold compared with unsupplemented medium. When radiolabeled ManNAc was used to trace the incorporation of the precursor, it was found that supplemental ManNAc was exclusively incorporated into IFN-gamma as sialic acid and that, at 20 mM ManNAc feeding, nearly 100% of product sialylation originated from the supplemental precursor.

823 ||| 17476501 ||| (6, 0.81) ||| 6 ||| 7 ||| Effect of culture temperature on follicle-stimulating hormone production by Chinese hamster ovary cells in a perfusion bioreactor. Follicle-stimulating hormone (FSH) was produced in Chinese hamster ovary (CHO) cells using a perfusion bioreactor. Perfusion culture at 37 degrees C yielded a high cell density but a low FSH production. To investigate the effect of culture temperature in the range of 26-37 degrees C on cell growth and FSH production, batch cultures were performed. Lowering culture temperature below 32 degrees C resulted in growth suppression. However, specific productivity of FSH, q (FSH), increased as culture temperature decreased, and the maximum q (FSH) of 43.4 ng/10(6) cells/h was obtained at 28 degrees C, which is 13-fold higher than that at 37 degrees C. Based on the results obtained from batch cultures, we performed perfusion cultures with two consecutive temperatures. CHO cells were grown up to 3.2 x 10(7) cells/ml at 37 degrees C and culture temperature shifted down to 28 degrees C to obtain a high FSH titer. Soon after the maximum FSH titer of 21 mug/ml was achieved, a rapid loss of not only viable cell concentration but also cell viability was observed, probably due to the low activities of enzymes related to cell growth. Thus, the extension of production period at 28 degrees C is critical for the enhancement of FSH production, and the use of antiapoptotic genes seems to be promising.

824 ||| 8622981 ||| (6, 0.79) ||| 6 ||| 7 ||| Protein-free cell culture on an artificial substrate with covalently immobilized insulin. Insulin was immobilized on a surface-hydrolyzed poly(methyl methacrylate) film. Chinese hamster ovary cells overexpressing human insulin receptors were cultured on the film in the absence of serum or soluble proteins. Small amounts of immobilized insulin (1-10% of the required amount of free insulin) were sufficient to stimulate cell proliferation. In addition, the maximal mitogenic effect of immobilized insulin was greater than that of free insulin. Immobilized insulin activated the insulin receptor and downstream signaling proteins, and this activation persisted for longer periods than that obtained with free insulin, probably explaining the greater mitogenic effect of the immobilized insulin. Finally the immobilized-insulin film was usable repeatedly without marked loss of activity.

825 ||| 23648861 ||| (6, 0.78) ||| 6 ||| 7 ||| Effect of mild-thiol reducing agents and alpha2,3-sialyltransferase expression on secretion and sialylation of recombinant EPO in CHO cells. We have previously reported that N-acetylcysteine (NAC) not only delayed apoptosis but also enhanced the production of recombinant erythropoietin (EPO) in Chinese hamster ovary (CHO) cell culture. To investigate the production enhancement mechanism, the effects of similar thiolreducing agents were studied. Intriguingly, all mild reducing agents examined including mercaptoethanesulfonic acid (MESNA), thiolactic acid (TLA), and thioglycolate (TG) were shown to block apoptosis and increase EPO production. A pulse-chase study of EPO secretion revealed that all four thiol-reducing agents increased the EPO secretion rate; among them TLA showed the highest rate. In terms of product quality, the sialic acid content of the glycoprotein is one of the most important factors. It was reported that a number of glycoproteins produced by CHO cells often have incomplete sialylation, particularly under high-producing conditions. Human alpha2,3-sialyltransferase (alpha2,3-ST) was introduced into EPO-producing CHO cells in order to compensate for the reduced sialylation during supplementation with NAC. When alpha2,3-ST was expressed in the presence of NAC, reduced sialylation was restored and an even more sialylated EPO was produced. Thus, our study is significant in that it offers increased EPO production while still allowing the prevention of decreased sialylation of EPO.

826 ||| 20506284 ||| (6, 0.78) ||| 6 ||| 7 ||| An investigation of intracellular glycosylation activities in CHO cells: effects of nucleotide sugar precursor feeding. Controlling glycosylation of recombinant proteins produced by CHO cells is highly desired as it can be directed towards maintaining or increasing product quality. To further our understanding of the different factors influencing glycosylation, a glycosylation sub-array of 79 genes and a capillary electrophoresis method which simultaneously analyzes 12 nucleotides and 7 nucleotide sugars; were used to generate intracellular N-glycosylation profiles. Specifically, the effects of nucleotide sugar precursor feeding on intracellular glycosylation activities were analyzed in CHO cells producing recombinant human interferon-gamma (IFN-gamma). Galactose (+/-uridine), glucosamine (+/-uridine), and N-acetylmannosamine (ManNAc) (+/-cytidine) feeding resulted in 12%, 28%, and 32% increase in IFN-gamma sialylation as compared to the untreated control cultures. This could be directly attributed to increases in nucleotide sugar substrates, UDP-Hex ( approximately 20-fold), UDP-HexNAc (6- to 15-fold) and CMP-sialic acid (30- to 120-fold), respectively. Up-regulation of B4gal and St3gal could also have enhanced glycan addition onto the proteins, leading to more complete glycosylation (sialylation). Combined feeding of glucosamine + uridine and ManNAc + cytidine increased UDP-HexNAc and CMP-sialic acid by another two- to fourfold as compared to feeding sugar precursors alone. However, it did not lead to a synergistic increase in IFN-gamma sialylation. Other factors such as glycosyltransferase or glycan substrate levels could have become limiting. In addition, uridine feeding increased the levels of uridine- and cytidine-activated nucleotide sugars simultaneously, which could imply that uridine is one of the limiting substrates for nucleotide sugar synthesis in the study. Hence, the characterization of intracellular glycosylation activities has increased our understanding of how nucleotide sugar precursor feeding influence glycosylation of recombinant proteins produced in CHO cells. It has also led to the optimization of more effective strategies for manipulating glycan quality.

827 ||| 22358597 ||| (6, 0.77) ||| 6 ||| 7 ||| Chinese hamster ovary cells produce sufficient recombinant insulin-like growth factor I to support growth in serum-free medium. Serum-free growth of IGF-I-producing CHO cells. Insulin-like growth factor I has similar mitogenic effects to insulin, a growth factor required by most cells in culture, and it can replace insulin in serum-free formulations for some cells. Chinese Hamster Ovary cells grow well in serum-free medium with insulin and transferrin as the only exogenous growth factors. An alternative approach to addition of exogenous growth factors to serum-free medium is transfection of host cells with growth factor-encoding genes, permitting autocrine growth. Taking this approach, we constructed an IGF-I heterologous gene driven by the cytomegalovirus promoter, introduced it into Chinese Hamster Ovary cells and examined the growth characteristics of Insulin-like growth factor I-expressing clonal cells in the absence of the exogenous factor. The transfected cells secreted up to 500 ng/10(6) cells/day of mature Insulin-like growth factor I into the conditioned medium and as a result they grew autonomously in serum-free medium containing transferrin as the only added growth factor. This growth-stimulating effect, observed under both small and large scale culture conditions, was maximal since no further improvement was observed in the presence of exogenous insulin.

828 ||| 15575729 ||| (6, 0.76) ||| 6 ||| 7 ||| Temperature effects on product-quality-related enzymes in batch CHO cell cultures producing recombinant tPA. Culture conditions that affect product quality are important to the successful operation and optimization of bioreactors. Previous studies have demonstrated that enzymes, such as proteases and sialidases, accumulate in batch bioreactors. These enzymes are known to be detrimental to the quality of recombinant glycoproteins. Bioreactor temperature has been used to control cell growth and recombinant protein production rates. However, the effect of culture temperature on the production of proteases and sialidases has not been investigated. In this study, Chinese hamster ovary cells were cultured with a temperature profile that decreased from 37 to 34 degrees C over 8 days and with a constant temperature of 37 degrees C. Analysis of extracellular protease activity indicated that two major proteases were present (50 and 69 kDa). The 50 kDa protease activity decreased similarly with time for both culture conditions. The 69 kDa protease activity increased with time for both culture conditions. The constant-temperature cultures had significantly lower 69 kDa protease levels compared to the ramped-temperature cultures in the early stationary phase. Intracellular sialidase activity was present in both cultures. The intracellular sialidase activity increased dramatically for both culture conditions immediately after the cells were inoculated into fresh medium. The initial peak in intracellular sialidase activity was followed by a first-order decay. The intracellular sialidase activities for the two culture conditions were not significantly different. The production of recombinant tissue type plasminogen activator was not significantly different for the two culture conditions. Thus, the previously hypothesized advantages that lower culture temperatures have reduced protease activity and improved productivity do not appear to be universal.

829 ||| 12599255 ||| (6, 0.74) ||| 6 ||| 7 ||| Effect of low culture temperature on specific productivity, transcription level, and heterogeneity of erythropoietin in Chinese hamster ovary cells. To determine the effect of low culture temperature on erythropoietin (EPO) production in recombinant Chinese hamster ovary (rCHO) cells, rCHO cells producing EPO (LGE10-9-27) were cultivated at 30, 33, and 37 degrees C. At a culture temperature lower than 37 degrees C cell growth was suppressed, but cell viability remained high for a longer culture period. When the culture temperature was lowered from 37 degrees C to 33 degrees C, more than a 2.5-fold increase in the maximum EPO concentration was achieved. This enhanced EPO production at 33 degrees C was not just because of the extended culture longevity with the decreased release of proteolytic enzymes from dead cells, but mainly because of enhanced q(EPO). The q(EPO) at 33 degrees C was 0.35 +/- 0.08 microg/10(6) cells/h, which was approximately 4-fold higher than that at 37 degrees C. Although the highest q(EPO) of 0.49 +/- 0.14 micro/10(6) cells/h was obtained at 30 degrees C, the maximum EPO concentration was lowest because the detrimental effect of lowering culture temperature on cell growth outweighed its beneficial effect on q(EPO). Like q(EPO), the relative EPO mRNA content increased by lowering culture temperature, indicating that the increased transcription level of EPO was responsible in part for the enhanced q(EPO) at low culture temperature. The quality of EPO produced at 33 degrees C in regard to isoform pattern, sialic acid content, and in vivo biological activity was comparable to or even better than that produced at 37 degrees C. Taken together, the results obtained demonstrate the potential of the application of low culture temperature to the commercial EPO production in rCHO cells.

830 ||| 18642278 ||| (6, 0.72) ||| 6 ||| 7 ||| Gamma-interferon production and quality in stoichiometric fed-batch cultures of Chinese hamster ovary (CHO) cells under serum-free conditions. The application of a stoichiometric medium design approach was studied in fed-batch cultivation of Chinese hamster ovary (CHO) cells. A serum-free medium containing a very low protein concentration (2 mg/L insulin) was developed. A supplemental medium was formulated according to the stoichiometric equation governing cell growth using cell composition obtained from hybridoma cells. Fed-batch culture was conducted in spinner flasks using the supplemental medium for feeding. Significant improvement in cell growth, by-product reduction, and Gamma-Interferon (IFN-gamma) production was achieved as compared to a typical batch culture. Results indicate that the stoichiometric approach, originally developed for hybridoma cultures, is a fast and effective method for cell culture process design and improvement. The glycosylation of IFN-gamma was monitored off-line during the culture process. The accumulative IFN-gamma glycosylation efficiency was slightly improved as compared to that of the batch culture, due to the nutritional control through the stoichiometric feeding. Periodic glucose starvation was observed during the fed-batch culture as a result of the manual feeding. Pulse-chase radiolabeling assay shows that glucose starvation leads to a deteriorated IFN-gamma glycosylation efficiency. (c) 1997 John Wiley & Sons, Inc. Biotechnol Bioeng 56: 577-582, 1997.

831 ||| 15296466 ||| (6, 0.71) ||| 6 ||| 7 ||| Effect of simultaneous application of stressful culture conditions on specific productivity and heterogeneity of erythropoietin in Chinese hamster ovary cells. A single stressful culture condition induced by hypoosmotic stress (210 mOsm kg(-1)), low culture temperature (32 degrees C), or NaBu addition (1 mM) resulted in a 1.8- to 2.2-fold enhancement of specific erythropoietin (EPO) productivity (qEPO) of recombinant Chinese hamster ovary (rCHO) cells compared to normal culture condition (37 degrees C and 310 mOsm kg(-1)). Simultaneous application of these stressful conditions further enhanced qEPO up to approximately 5-fold. However, the quality of EPO was affected by stressful culture conditions. The proportion of acidic isoforms of EPO under a single stressful condition was 2.8-13.8% lower than that under normal culture condition. Simultaneous application of the stressful conditions further decreased the portion of acidic isoforms but not significantly. Despite 5-fold enhancement of q(EPO), the portion of acidic isoforms under the simultaneous application of stressful culture conditions was 12.9-21.6% lower than that under normal culture condition. Taken together, these results suggest the potential of simultaneous application of different stressful culture conditions to the production phase of two-stage culture, where cell growth and production phases are separated, for improved EPO production.

832 ||| 18636497 ||| (6, 0.69) ||| 6 ||| 7 ||| Site- and branch-specific sialylation of recombinant human interferon-gamma in Chinese hamster ovary cell culture. Since sialic acid content is known to be a critical determinant of the biological properties of glycoproteins, it is essential to characterize and monitor sialylation patterns of recombinant glycoproteins intended for therapeutic use. This study reports site- and branch-specific differences in sialylation of human interferon-gamma (IFN-gamma) derived from Chinese hamster ovary (CHO) cell culture. Sialylation profiles were quantitated by reversed-phase HPLC separations of the site-specific pools of tryptic glycopeptides representing IFN-gamma's two potential N-linked glycosylation sites (i.e., Asn(25) and Asn(97)). Although sialylation at each glycosylation site was found to be incomplete, glycans of Asn(25) were more heavily sialylated than those of Asn(97). Furthermore, Man(alpha1-3) arms of the predominant complex biantennary structures were more favorably sialylated than Man(alpha1-6) branches at each glycosylation site. When the sialylation profile was analyzed throughout a suspension batch culture, sialic acid content at each site and branch was found to be relatively constant until a steady decrease in sialylation was observed coincident with loss of cell viability. The introduction of a competitive inhibitor of sialidase into the culture supernatant prevented the loss of sialic acid after the onset of cell death but did not affect sialylation prior to cell death. This finding indicated that incomplete sialylation prior to loss of cell viability could be attributed to incomplete intracellular sialylation while the reduction in sialylation following loss of cell viability was due to extracellular sialidase activity resulting from cell lysis. Thus, both intracellular and extracellular processes defined the sialic acid content of the final product. (c) 1997 John Wiley & Sons, Inc. Biotechnol Bioeng 55: 390-398, 1977.

833 ||| 19002921 ||| (6, 0.66) ||| 6 ||| 7 ||| Stable expression of recombinant human coagulation factor XIII in protein-free suspension culture of Chinese hamster ovary cells. The recombinant a and bsubunits for human coagulation factor XIII were transfected into Chinese hamster ovary (CHO) cells. CHO cells were amplified and selected with methotrexate in adherent cultures containing serum, and CHO 1-62 cells were later selected in protein-free medium. To develop a recombinant factor XIII production process in a suspension culture, we have investigated the growth characteristics of CHO cells and the maintenance of factor XIII expression in the culture medium. Suspension adaptation of CHO cells was performed in protein-free medium, GC-CHO-PI, by two methods, such as serum weaning and direct switching from serum containing media to protein-free media. Although the growth of CHO cells in suspension culture was affected initially by serum depletion, cell specific productivity of factor XIII showed only minor changes by the direct switching to protein-free medium during a suspension culture. As for the long-term stability of factor XIII, CHO 1-62 cells showed a stable expression of factor XIII in protein-free condition for 1000 h. These results indicate that the CHO 1-62cells can be adapted to express recombinant human factor XIII in a stable maimer in suspension culture using a protein-free medium. Our results demonstrate that enhanced cell growth in a continuous manner is achievable for factor XIII production in a protein-free medium when a perfusion bioreactor culture system with a spin filter is employed.

834 ||| 16233483 ||| (6, 0.65) ||| 6 ||| 7 ||| Repeated addition of insulin for dynamic control of apoptosis in serum-free culture of Chinese hamster ovary cells. Repeated addition of insulin, together with an initial addition of the reduced form of glutathione (GSH) and a chelator, aurintricarboxylic acid (ATA), was proposed as a strategy for the dynamic control of apoptosis in serum-free culture of Chinese hamster ovary (CHO) cells. Insulin suppressed cell death in serum-free culture by means of lowering intracellular reactive oxygen species levels via an increase in the specific glucose consumption rate. This effect of insulin addition was found not only at 0 h but also at 48 h during serum-free cultivation. In consideration of the reduction of insulin by GSH, insulin was again added at 24 h following the initial addition during the serum-free cultivation. The repeated addition of insulin decreased cell death and increased the production of tissue plasminogen activator compared with the single addition at 0 h.

835 ||| 8800692 ||| (6, 0.64) ||| 6 ||| 7 ||| Viable AC-2, a new adult bovine serum- and colostrum-based supplement for the culture of mammalian cells. In this study we have shown that Viable AC-2, a medium based on the ultrafiltrate fraction of bovine colostrum and adult bovine serum, can be used successfully as a fetal bovine serum (FBS) substitute in the culture of several anchorage-dependent and independent cell lines. Of the 15 cell lines cultured in 8% Viable AC-2 in microplates, 10 reached the maximum cell density of 65%-123% of that in 10% FBS, 4 cell lines reached maximum cell density of 35%-65% of that in 10% FBS and only one cell line, a human osteosarcoma G-292, grew slowly in Viable AC-2. In a small-scale suspension culture, 8%-15% Viable AC-2 supports the growth of Chinese hamster ovary cells (CHO-K1) on microcarriers in spinner flasks significantly better than 10% FBS. Shionogi mouse mammary tumour cell line (S115) transfected with human alpha 2-adrenergic receptor subtype C2 was used as a model to study recombinant protein production in Viable AC-2-supplemented medium. The results showed that in cell culture flasks and in an ACUSYST-R bioreactor, the alpha 2-C2 receptor expression level per mg of total protein was similar in both Viable AC-2 and FBS.

836 ||| 20680262 ||| (6, 0.63) ||| 6 ||| 7 ||| Substitution of glutamine by glutamate enhances production and galactosylation of recombinant IgG in Chinese hamster ovary cells. The effect of ammonia on Chinese hamster ovary (CHO) cell growth and galactosylation of recombinant immunoglobulin (rIgG) was investigated using shaking flasks with serum free media containing 0-15 mM NH(4)Cl. The elevated ammonia inhibited cell growth and negatively affected the galactosylation of rIgG. At 15 mM NH(4)Cl, the proportions of monogalactosylated glycan with fucosex (monogalactosylated glycan with fucose) and digalactosylated glycan with fucose (G2F) were 23.9% and 6.3% lower than those at 0 mM NH(4)Cl, respectively. To reduce ammonia formation by cells, glutamate was examined as a substitute for glutamine. The use of glutamate reduced the accumulation of ammonia and enhanced the production of rIgG while depressing cell growth. At 6 mM glutamate, ammonia level did not exceed 2 mM, which is only one third of that at 6 mM glutamine. Also, a 1.7-fold increase in the titer of rIgG and specific rIgG productivity, q (rIgG), was achieved at 6 mM glutamate. The galactosylation of rIgG was favorable at 6 mM glutamate. The proportion of galactosylated glycans, G1F and G2F, at 6 mM glutamate was 59.8%, but it was 50.4% at 6 mM glutamine. The use of glutamate also increased complement-dependent cytotoxicity activity, one of the effector functions of rIgG. Taken together, substitution of glutamine by glutamate can be considered relevant for the production of rIgG in CHO cells since glutamate not only enhances q (rIgG) but also generates a higher galactosylation essential for the effector function of rIgG.

837 ||| 11027158 ||| (6, 0.62) ||| 6 ||| 7 ||| Effects of insulin and LongR(3) on serum-free Chinese hamster ovary cell cultures expressing two recombinant proteins. Insulin is the most commonly used growth factor for sustaining cell growth and viability in serum-free Chinese hamster ovary (CHO) cell cultures. In the present study insulin and IGF-1 analogue (LongR(3)) were compared for their ability to support growth, viability, and production of two serum-free CHO cell lines expressing recombinant protein. The first cell line, VA12, expresses protein B, and the second cell line, CL23, expresses protein C. Both molecules are recombinant cytokine receptors. VA12 will grow in serum-free media lacking growth factor, while CL23 requires either insulin or LongR(3) for cell growth. Both cell lines, however, require a growth factor for optimal performance under production conditions. In this study, LongR(3) was better able to sustain the viability of both cell lines under production conditions than insulin. These data indicate that while insulin and LongR(3) can both serve as growth and viability factors for CHO cells, LongR(3) is the preferred growth factor for cell lines VA12 and CL23.

838 ||| 22358924 ||| (6, 0.61) ||| 6 ||| 7 ||| Super-CHO-A cell line capable of autocrine growth under fully defined protein-free conditions. Chinese Hamster Ovary (CHO) cells are widely used for the large scale production of recombinant biopharmaceuticals. Growth of the CHO-K1 cell line has been demonstrated in serum-free medium containing insulin, transferrin and selenium. In an attempt to get autocrine growth in protein-free medium, DNA coding for insulin and transferrin production was transfected into CHO-K1 cells. Transferrin was expressed well, with clones secreting approximately 1000 ng/10(6) cells/24h. Insulin was poorly expressed, with rates peaking at 5 ng/10(6) cells/24h. Characterisation of the secreted insulin indicated that the CHO cells were incompletely processing the insulin molecule. Site-directed mutagenesis was used to introduce a furin (prohormone converting enzyme) recognition sequence into the insulin molecule, allowing the production of active insulin. However, the levels were still too low to support autocrine growth. Further investigations revealed insulin degrading activity (presumably due to the presence of insulin degrading enzymes) in the cytoplasm of CHO cells. To overcome these problems insulin-like growth factor I (instead of insulin) was transfected into the cells. IGF-1 was completely processed and expressed at rates greater than 500 ng/10(6)cells/24h. In this paper we report autonomous growth of the transfected CHO-K1 cell line expressing transferrin and IGF-1 in protein-free medium without the addition of exogenous growth factors. Growth rates and final cell densities of these cells were identical to that of the parent cell line CHO-K1 growing in insulin, transferrin, and selenium supplemented serum-free media.

839 ||| 11822911 ||| (6, 0.61) ||| 6 ||| 7 ||| Effects of ammonia and glucosamine on the heterogeneity of erythropoietin glycoforms. Recombinant human erythropoietin (EPO) is a glycoprotein produced as a therapeutic agent from mammalian cell cultures for the treatment of anemia associated with severe kidney damage. The EPO structure has a high glycan content which is essential for bioactivity but shows considerable molecular heterogeneity. The cell culture conditions that affect the heterogeneity of the glycoforms of EPO are not well understood. However, the accumulation of ammonia in culture is one factor that has been associated with an enhanced heterogeneity of glycoforms. In this report we investigate the metabolic perturbations associated with ammonia and glucosamine that may give rise to an altered pattern of EPO glycosylation. Recombinant human erythropoietin was synthesized in serum-free cultures of transfected Chinese hamster ovary (CHO) cells. The molecular heterogeneity of erythropoietin was increased by supplementation of cultures with either ammonia or glucosamine. The enhanced molecular heterogeneity was shown to be due to variable glycosylation that resulted in EPO with an enhanced molecular weight and isoelectric point range. Enzymatic removal of the glycan moiety of EPO in all cases resulted in a single molecular form with a molecular weight of 18 000, which corresponded to non-glycosylated EPO. The variable glycosylation was consistent with reduced sialylation and antennarity of the carbohydrate structures present on the three N-linked sites of EPO. In the presence of ammonia (>30 mM) the proportion of tetrasialylated and tetraantennary glycan structures were reduced by 73% and 57%, respectively, as determined by HPLC analysis. Such changes were also observed, although to a lesser extent (41% and 37%), by an increase in the glucosamine concentration (>10 mM) in the medium. The enhanced heterogeneity of the glycan structures coincided with a significant increase in the intracellular UDP-N-acetylhexosamine (UDP-GNAc) pool. The measured UDP-GNAc level was up to 2 orders of magnitude higher in the presence of either glucosamine or ammonia. However, the changes in the glycosylation profiles induced by either glucosamine or ammonia were significantly different even at the same intracellular UDP-GNAc concentration. This suggests that the enhanced EPO heterogeneity could not be mediated solely by the increased UDP-GNAc level. Glucosamine (but not ammonia) was shown to cause significant inhibition of glucose transport into the cells, which could induce a different pattern of primary metabolism.

840 ||| 16253368 ||| (6, 0.59) ||| 6 ||| 7 ||| Adaptation of Chinese hamster ovary cells to low culture temperature: cell growth and recombinant protein production. Recombinant Chinese hamster ovary (rCHO) cells producing erythropoietin (EPO) and rCHO cells producing follicle-stimulating hormone (FSH) showed a significant increase in specific productivity (q) when grown at 32 degrees C compared to 37 degrees C. However, low culture temperature suppressed cell growth, and therefore, did not increase volumetric productivity as much as q. In an attempt to increase the volumetric productivity through improvement of hypothermic growth, EPO producing rCHO (CHO-EPO) cells and FSH producing rCHO (CHO-FSH) cells were adapted at 32 degrees C in a repeated batch mode using spinner flasks. Cell growth of both CHO-EPO and CHO-FSH gradually improved during adaptation at 32 degrees C. Specific growth rates of CHO-EPO and CHO-FSH cells at 32 degrees C, through adaptation, were increased by 73% and 20%, respectively. During adaptation at 32 degrees C, mRNA levels of cold-inducible RNA-binding protein (CIRP) of both rCHO cell lines did not change significantly, suggesting that CIRP expression may not be the only cause for growth suppression at low culture temperature. Unlike cell growth, the recombinant protein production of both rCHO cell lines was not increased during adaptation due to decreased specific productivities. The specific EPO productivity and specific FSH productivity were decreased by 49% and 22%, respectively. Southern blot analyses showed that the decreased specific productivities were not due to the loss of foreign gene copies. Taken together, improvement of hypothermic cell growth by adaptation does not appear to be applicable for enhanced recombinant protein production, since specific productivity decreases during adaptation to the low culture temperature.

841 ||| 10799988 ||| (6, 0.59) ||| 6 ||| 7 ||| Ammonium alters N-glycan structures of recombinant TNFR-IgG: degradative versus biosynthetic mechanisms. The effect of ammonium on the glycosylation pattern of the recombinant immunoadhesin tumor necrosis factor-IgG (TNFR-IgG) produced by Chinese hamster ovary cells is elucidated in this study. TNFR-IgG is a chimeric IgG fusion protein bearing one N-linked glycosylation site in the Fc region and three complex-type N-glycans in the TNF-receptor portion of each monomer. The ammonium concentration of batch suspension cultures was adjusted with glutamine and/or NH(4)Cl. The amount of galactose (Gal) and N-acetylneuraminic acid (NANA) residues on TNFR-IgG correlated in a dose-dependent manner with the ammonium concentration under which the N-linked oligosaccharides were synthesized. As ammonium increased from 1 to 15 mM, a concomitant decrease of up to 40% was observed in terminal galactosylation and sialylation of the molecule. Cell culture supernatants contained measurable beta-galactosidase and sialidase activity, which increased throughout the culture. The beta-galactosidase, but not the sialidase, level was proportional to the ammonium concentration. No loss of N-glycans was observed in incubation studies using beta-galactosidase and sialidase containing cell culture supernatants, suggesting that the ammonium effect was biosynthetic and not degradative. Several biosynthetic mechanisms were investigated. Ammonium (a weak base) is known to affect the pH of acidic intracellular compartments (e.g., trans-Golgi) as well as intracellular nucleotide sugar pools (increases UDP-N-acetylglucosamine and UDP-N-acetylgalactosamine). Ammonium might also affect the expression rates of beta1, 4-galactosyltransferase (beta1,4-GT) and alpha2,3-sialyltransferase (alpha2,3-ST). To separate these mechanisms, experiments were designed using chloroquine (changes intracellular pH) and glucosamine (increases UDP-GNAc pool [sum of UDP-GlcNAc and UDP-GalNAc]). The ammonium effect on TNFR-IgG oligosaccharide structures could be mimicked only by chloroquine, another weak base. No differences in N-glycosylation were found in the product synthesized in the presence of glucosamine. No differences in beta1, 4-galactosyltransferase (beta1,4-GT) and alpha2,3-sialyltransferase (alpha2,3-ST) messenger RNA (mRNA) and enzyme levels were observed in cells cultivated in the presence or absence of 13 mM NH(4)Cl. pH titration of endogenous CHO alpha2,3-ST and beta-1,4-GT revealed a sharp optimum at pH 6.5, the reported trans-Golgi pH. Thus, at pH 7.0 to 7.2, a likely trans-Golgi pH range in the presence of 10 to 15 mM ammonium, activities for both enzymes are reduced to 50% to 60%. Consequently, ammonium seems to alter the carbohydrate biosynthesis of TNFR-IgG by a pH-mediated effect on glycosyltransferase activity.

842 ||| 26019520 ||| (6, 0.57) ||| 6 ||| 7 ||| Poly-gamma-glutamic acid enhances the quality of recombant erythropoietin produced by CHO cells. The effect of poly-gamma-glutamic acid (gammaPGA), which is produced by Bacillus sp., on the production of recombinant erythropoietin (rEPO) by Chinese hamster ovary (CHO) cells in suspension culture was evaluated. The growth, viability, and productivity of recombinant CHO cells were investigated in a chemically defined medium with 50 and 500 kD gammaPGAs at 0.075% or with Pluronic F68. Cell growth with the two gammaPGAs was lower than that with Pluronic F68 but significantly higher than that without any additive (control). The effect of additives on rEPO productivity was 50 kDa gammaPGA > 500 kDa gammaPGA > Pluronic F68 > control. Using EPO-dependent F-36E cells, we found that the effect of additives on rEPO quality was 500 kDa gammaPGA > 50 kDa gammaPGA > control > Pluronic F68. gammaPGA has an enhancement effect on the quality of rEPO produced by CHO cells.

843 ||| 23239488 ||| (6, 0.57) ||| 6 ||| 7 ||| Combination of yeast hydrolysates to improve CHO cell growth and IgG production. Many studies underlined the great benefits of hydrolysates used as additives in animal free media on cell culture performances. However, to precisely define hydrolysate supplementation strategies, a deeper understanding of their effect on cell growth and protein production is required. In the present study, the effect of addition of one yeast extract (YE) and two yeast peptones (named YP.A and YP.B) in a chemically defined medium was first assessed on cell culture performances. Interestingly, specific effects were found depending on the degree of degradation of yeast hydrolysates. The YE at 1 g L(-1) increased the maximal cell density by 70 %, while a mixture of YE (1 g L(-1)) and YP.A (4 g L(-1)) increased IgG production by 180 %. These conditions were then evaluated on the CHO cell kinetics all over cultures. Hydrolysates extended the cell growth phase in Erlenmeyer flask and increased the maximal growth rate in bioreactor up to 20 %. Cell growth stimulation induced by hydrolysates addition was linked with energetic metabolism improvement suggesting that they promote oxidative pathway. Furthermore, hydrolysates provided an additional source of substrate that supported cell growth despite glutamine limitation.

844 ||| 19003275 ||| (6, 0.57) ||| 6 ||| 7 ||| Growth and secretion of erythropoietin of Chinese hamster ovary cells coexpressing epidermal growth factor receptor and erythropoietin genes: Design of cells for cell culture matrix. Chinese hamster ovary (CHO) cells were transfected withboth genes encoding erythropoietin (Epo) and epidermal growthfactor receptor (EGFR). The transfection of the Epo gene wasconfirmed by an enzyme-linked immunoassay. Overexpression ofEGFR was confirmed by Western blotting of EGFR. Thetransfected CHO cells were cultured in serum-free medium inthe presence of soluble epidermal growth factor (EGF) orimmobilized EGF. The CHO cells overexpressing EGFR grew in thepresence of less EGF than the cells not overexpressing EGFR.In addition, the growth of EGFR-overexpressing CHO cells wasenhanced in the presence of immobilized EGF more efficientlythan in the presence of soluble EGF. The amount of Eposecreted from the cells increased linearly with the increaseof growth rate. Consequently, culture of CHO cellscoexpressing Epo and EGFR on EGF-immobilized matrix was themost efficient for Epo production.

845 ||| 16797979 ||| (6, 0.57) ||| 6 ||| 7 ||| Usability of size-excluded fractions of soy protein hydrolysates for growth and viability of Chinese hamster ovary cells in protein-free suspension culture. To investigate the effect of size-excluded fraction of non-animal protein hydrolysate on growth, viability and longevity of Chinese hamster ovary (CHO) cells, several commercially available protein hydrolysates were evaluated as a feed supplement to chemically-defined protein-free suspension culture. Soy protein hydrolysates showed better supporting capability for cell growth and viability than the other types of hydrolysates. Maximal cell growth was not affected greatly by size exclusion of some soy hydrolysates such as bacto soytone and soy hydrolysates. CHO cells supplemented with size-excluded fractions of the two hydrolysates showed viable cell density and viability almost equal to those with their crude hydrolysates, although soy hydrolysates showed a little better performance. This suggested that the size-excluded hydrolysate fractions of some soy hydrolysate might be a potential culture medium additive to achieve better downstream operation in a large-scale production as well as enhanced productivity.

846 ||| 16233042 ||| (6, 0.57) ||| 6 ||| 7 ||| Aurintricarboxylic acid exerts insulin-like growth stimulating effects on Chinese hamster ovary cells under serum-free conditions. When aurintricarboxylic acid (ATA) was added at a concentration of 30 mg/l in DME/F12 medium to Chinese hamster ovary (CHO) NTHU-108 cells in static six-well plates, some of the cells exhibited adherent growth while others grew in suspension. Beyond the critical concentration of ATA, CHO cells grew in single-cell suspension. In the same serum-free medium, insulin at a level of 0.5 mg/l was found to be the most important protein ingredient promoting cell growth. We used the respective kinase inhibitors to investigate their influence on the cell proliferation induces by ATA and insulin. It is interesting that the inhibition by seven kinase inhibitors of ATA-induced proliferation is similar to that of insulin-induced proliferation. It is possible that ATA mimics insulin and influences the mitogen activated signal transduction to induce the proliferation of CHO cells. Although the actual mechanism of the proliferation of CHO cells by ATA is unclear, ATA supported the long-term proliferation of CHO cells under serum-free conditions and thus could be used as a good substitute for insulin in the formulation of protein-free media.

847 ||| 15593097 ||| (6, 0.57) ||| 6 ||| 7 ||| Impact of dynamic online fed-batch strategies on metabolism, productivity and N-glycosylation quality in CHO cell cultures. As we pursue the means to improve yields to meet growing therapy demands, it is important to examine the impact of process control on glycosylation patterns to ensure product efficacy and consistency. In this study, we describe a dynamic on-line fed-batch strategy based on low glutamine/glucose concentrations and its impact on cellular metabolism and, more importantly, the productivity and N-glycosylation quality of a model recombinant glycoprotein, interferon gamma (IFN-gamma). We found that low glutamine fed-batch strategy enabled up to 10-fold improvement in IFN-gamma yields, which can be attributed to reduced specific productivity of ammonia and lactate. Furthermore, the low glutamine concentration (0.3 mM) used in this fed-batch strategy could maintain both the N-glycosylation macro- and microheterogeneity of IFN-gamma. However, very low glutamine (<0.1 mM) or glucose (<0.70 mM) concentrations can lead to decreased sialylation and increased presence of minor glycan species consisting of hybrid and high-mannose types. This shows that glycan chain extension and sialylation can be affected by nutrient limitation. In addition to nutrient limitation, we also found that N-glycosylation quality can be detrimentally affected by low culture viability. IFN-gamma purified at low culture viability had both lower sialylation as well as glycans of lower molecular masses, which can be attributed to extensive degradation by intracellular glycosidases released by cytolysis. Therefore, in order to maintain good N-glycosylation quality, there is a need to consider both culture viability and nutrient control setpoint in a nutrient-limiting fed-batch culture strategy. A greater understanding of these major factors that affect N-glycosylation quality would surely facilitate future development of effective process controls.

848 ||| 11027159 ||| (6, 0.57) ||| 6 ||| 7 ||| Insulin-like growth factor-I and transferrin mediate growth and survival of Chinese hamster ovary cells. The aim of this investigation was to elucidate the roles of insulin-like growth factor-I (IGF-I) and transferrin in the survival and proliferation of Chinese hamster ovary (CHO) cells upon withdrawal of serum. For this purpose, we employed DNA analysis and flow cytometry to compare CHO cell lines expressing either IGF-I alone or IGF-I and transferrin. The ability of cells to cycle and the occurrence of apoptosis were monitored in these cells in serum-free medium. These results indicate that IGF-I alone is able to maintain the viability of CHO cells for an extended length of time in the absence of serum. Transferrin alone does not promote survival or proliferation. Only in the presence of both IGF-I and transferrin do cells survive and proliferate. Therefore, in attached CHO cultures, IGF-I alone does not stimulate cell proliferation but is a requirement for growth in serum-free medium in cooperation with transferrin. We report on the dual role of IGF-I as a survival factor in CHO cells and its interlocking role with transferrin to stimulate cell growth.

849 ||| 25931375 ||| (6, 0.55) ||| 6 ||| 7 ||| Galactose supplementation enhance sialylation of recombinant Fc-fusion protein in CHO cell: an insight into the role of galactosylation in sialylation. Sialic acid levels of therapeutic glycoprotein play an important role in plasma half-life. An undesirable decrease of sialic acid content was observed when we increased Fc-fusion protein productivity fourfold in a GS-CHO cell line by bioprocess optimization. We investigated the potential mechanism for the sialic acid content reduction. We found that limited nucleotide sugar precursor and the extracellular sialidase were not responsible for the reduction of the sialic acid content after titer improvement. Oligosaccharide analysis revealed that the lack of protein galactosylation was the potential cause for the reduction of sialic acid content. Thus we validated this notion by evaluated galactose supplementation in 2 L bioreactors. Cell culture performance was not impacted by addition of up to 40 mM galactose except for the glucose consumption rate. Addition of 20 mM galactose to the bioreactor resulted in the increase of 44 % for total sialic acid content and 20.3 % for sialylated glycans. These data were further validated when the process was run on 200 L scaled bioreactor. These data together show that the galactosylation plays an apparent role in sialylation in our current system.

850 ||| 23701045 ||| (6, 0.55) ||| 6 ||| 7 ||| Growth, metabolic activity, and productivity of immobilized and freely suspended CHO cells in perfusion culture. Chinese hamster ovary (CHO) cells producing beta-galactosidase (beta-gal) were successfully cultured on silicone-based porous microcarriers (ImmobaSil FS) in a 1 L stirred-tank perfusion bioreactor. We studied the growth, metabolism, and productivity of free and immobilized cells to understand cellular activity in immobilized conditions. CHO cells attached to ImmobaSil FS significantly better than to other microcarriers. Scanning electron microscope images showed that the CHO cells thoroughly colonized the porous surfaces of the ImmobaSil FS, exhibiting a spherical morphology with microvilli that extended to anchorage cells on the silicone surface. In perfusion culture, the concentration of the attached cells reached 8 x 10(8) cells/mL of carrier, whereas those that remained freely suspended reached 2 x 10(7) cells/mL medium. The beta-gal concentration reached more than 5 unit/mL in perfusion culture, more than fivefold that of batch culture. The maximum concentration per microcarrier was proportional to the initial cell density. The specific growth rate, the specific beta-gal production rate, the percentage of S phase, and the oxygen uptake rate were all relatively lower for immobilized cells than freely suspended cells in the same bioreactor, indicating that not only do cells survive and grow to a greater extent in a free suspension state, but they are also metabolically more active than viable cells inside the pores of the microcarriers.

851 ||| 18623434 ||| (6, 0.55) ||| 6 ||| 7 ||| Membrane anchored protein production from spheroid, porous, and solid microcarrier chinese hamster ovary cell cultures. The influence of the microcarrier type on the performance of a controlled release process used to produce a recombinant glycosyl-phosphatidylinositol anchored protein was investigated. Chinese hamster ovary (CHO) cells expressing the human melanoma tumor antigen (p97) were cultured in 10% serum on Cultispher-GH porous microcarriers and then, for protein production, maintained in 2% serum. Cells were harvested every 48 h and p97 was recovered at 90 mug/mL and 40% purity. Harvested p97 concentrations were increased by harvestingfrom spheroid (241 mug/mL) and smaller porous microcarrier, Cultispher-G (167 mug/mL) cultures. The low total cell specific p97 production of cells cultured on Cultispher-GH was due to necrosis of cells within the beads, decreased p97 expression of the immobilized cells, dilution by the liquid (up to 40% volume) associated with settled beads, and incomplete recovery of p97 from within the beads. Cells cultured on solid microcarriers, Cytodex-1, had the highest cell viability and cell specific p97 production, It is recommended that a two-stage cyclic harvesting process of cells cultured on small Cultispher-G or on Cytodex-1 beads would minimize protein loss and maximize cell specific protein recovery. (c) 1995 John Wiley & Sons Inc.

852 ||| 24557070 ||| (6, 0.54) ||| 6 ||| 7 ||| Sodium butyrate enhances the acidic isoform content of recombinant human erythropoietin produced by Chinese hamster ovary cells. Sodium butyrate is commonly used in mammalian cell cultures to increase the productivity of recombinant proteins. A Chinese hamster ovary (CHO) cell line producing recombinant human erythropoietin (rhEPO) was cultured in commercial medium. Addition of 0.5 mM butyrate inhibited the over-growth of the cells after the medium was changed from serum-added medium to serum-free medium. At the 6th day, the addition of butyrate lowered the transcriptional level of sialidases I, II, and III compared to that of control groups by 56, 87, and 59%, respectively. Extracellular sialidase activity was decreased by 53% by addition of butyrate. The inhibition of cell over-growth and the decrease of extracellular sialidase activity helped to increase the acidic isoform content of rhEPO expressed by this CHO cell strain.

853 ||| 19194894 ||| (6, 0.54) ||| 6 ||| 7 ||| High yields of monomeric recombinant beta-interferon from macroporous microcarrier cultures under hypothermic conditions. Macroporous microcarriers such as Cytopore entrap mammalian cells in a mesh network allowing growth to high cell concentrations in a protected environment within a stirred culture. Chinese hamster ovary (CHO) cells producing recombinant human beta-interferon (IFN-beta) and grown in Cytopore microcarriers showed a 2.6- to 2.8-fold increase in the volumetric product titer compared with cells grown in an equivalent suspension culture. In an attempt to maximize production of IFN-beta, microcarrier cultures were subjected to a low temperature regime. Low temperature culture conditions (32 degrees C) have been shown previously to enhance cell specific productivity in suspension cultures although at reduced cell growth rates. These conditions can be optimized by a timely shift from physiological to hypothermic conditions during the culture run to maximize volumetric protein production. In the case of IFN-beta production the lower temperature has the added advantage of stabilizing the product and reducing intramolecular aggregation. Using a biphasic temperature-shift regime from 37 to 32 degrees C the volumetric production of IFN-beta was enhanced to 4.2-fold compared with a single temperature suspension culture in a controlled bench-top bioreactor. Furthermore, the degree of intramolecular aggregation of IFN-beta was reduced significantly (59%) compared with control cultures, largely due to the lower temperature but also partially due to the presence of microcarriers. These results indicate that the hypothermic conditions in a Cytopore culture had a combined and possibly synergistic effect of increasing volumetric production of the recombinant protein.

854 ||| 22358914 ||| (6, 0.53) ||| 6 ||| 7 ||| Degradative activities in a recombinant chinese hamster ovary cell culture. Two degradative activities were found in a recombinant Chinese hamster ovary cell culture. These activities became more dominant under high cell density and extended running time, as achieved in a semi-continous perfusion culture. The first, insulin degradative activity caused a growth upset in the 3rd cycle of the perfusion culture and shortened the length of the bioreactor process. The second activity, derived from the neutral pH stable sialidase, was found to affect the integrity of the carbohydrate structure of the recombinant protein, causing increase in heterogeneity in molecular weight and pI of the glycoforms. The most efficient way to overcome these problems may be the use of genetically altered 'designer cells' as the production cell line.

855 ||| 20589456 ||| (6, 0.53) ||| 6 ||| 7 ||| Heparin promotes suspension adaptation process of CHO-TS28 cells by eliminating cell aggregation. While heparin has been shown to eliminate cell aggregation in suspension adaptations of insect and HEK293 cells for virus-based cell cultures, the role of heparin in long period serum-free suspension adaptation of the anchorage-dependent Chinese hamster ovary (CHO) cell lines remains inconclusive. In this paper, we explore the potential application of heparin in suspension adaptation of CHO cell line which produces an anti-human chimeric antibody cHAb18. Heparin showed a concentration-dependent inhibition of CHO-TS28 cell-to-cell adhesion, with a significant inhibitory effect occurring when the concentration exceeded 250 mug/ml (P < 0.001). Heparin also exhibited a cell aggregation elimination role at all concentrations (P < 0.001). Furthermore, heparin promoted cell growth and antibody secretion, with the highest cell density ((99.83 +- 12.21) x 10(4) cells/ml, P = 0.034) and maximum antibody yield ((9.46 +- 0.94) mg/l, P < 0.001) both occurring at 250 mug/ml heparin. When agitated, cell aggregates were effectively dispersed by 250 mug/ml heparin and a single-cell suspension culture process was promoted. In suspension adapted CHO-TS28 cells, cell growth rates and specific antibody productivity were maintained; while antigen-binding activity improved slightly. Together, our results show that heparin may promote suspension adaptation of anchorage-depended CHO cells by resisting cell aggregation without reducing cell growth, antibody secretion, and antigen-binding activity.

856 ||| 19002891 ||| (6, 0.53) ||| 6 ||| 7 ||| Metabolic characteristics of recombinant Chinese hamster ovary cells expressing glutamine synthetase in presence and absence of glutamine. To elucidate the metabolic characteristics of recombinant CHO cells expressing glutamine synthetase (GS) in the medium with or without glutamine, the concentrations of extra- and intracellular metabolites and the activities of key metabolic enzymes involved in glutamine metabolism pathway were determined. In the absence of glutamine, glutamate was utilized for glutamine synthesis, while the production of ammonia was greatly decreased. In addition, the expression of recombinant protein was increased by 18%. Interestingly, the intracellular glutamine maintained almost constant, independent of the presence of glutamine or not. Activities of glutamate-oxaloacetate aminotransferase (GOT), glutamate-pyruvate aminotransferase (GPT), and glutamate dehydrogenase (GDH) increased in the absence of glutamine. On the other hand, intracellular isocitrate and the activities of its downstream isocitrate dehydrogenase in the TCA cycle increased also. In combination with these two factors, a 8-fold increase in the intracellular alpha-ketoglutarate was observed in the culture of CHO-GS cells in the medium without glutamine.

857 ||| 15789755 ||| (6, 0.52) ||| 6 ||| 7 ||| Effect of low adapted temperature and medium composition on growth and erythropoietin (EPO) production by Chinese hamster ovary cells. Temperature and medium composition were changed with the aim of increasing growth and erythropoietin (EPO) production in EPO-producing Chinese hamster ovary (CHO) cells. We used the CHO cell line, IBE, and its derivative, CO5, which over-expresses the first two enzymes of the urea cycle, carbamoyl phosphate synthetase I (CPS I) and ornithine transcarbamoylase (OTC). When supplements were added to the medium at 33 degrees C, the growth of IBE and CO5 cells increased by 27% and 26%, respectively and the maximum yield of EPO was increased by 40% in both cell lines. The absolute EPO concentration in the CO5 cells was always 55-60% higher than in the IBE cells. In addition, when the two cell lines were continuously cultured with supplements at 33 degrees C until their growth rates approached those at 37 degrees C, the growth rates of both IBE and CO5 cells increased by 54% and their maximum EPO levels increased by up to 73% and 56%, respectively. Therefore, the growth and EPO expression levels of CO5 cells increased 2.2-fold and 2.6-fold, respectively, compared to those of the IBE cells. These results indicate that adaptation to lower temperature as well as medium supplementation could be important for improving cell growth and EPO production.

858 ||| 22358555 ||| (6, 0.51) ||| 6 ||| 7 ||| Development of a serum-free culture medium for the large scale production of recombinant protein from a Chinese hamster ovary cell line. A serum-free medium, WCM5, has been developed for the large scale propagation of CHO (Chinese hamster ovary) cells which express recombinant protein using dihydrofolate reductase as a selectable marker. WCM5 was prepared by supplementing Iscoves medium without lecithin, albumin or transferrin with a number of components which were shown to benefit growth. WCM5 medium contained 5 mg l(-1) human recombinant insulin (Nucellin) but was otherwise protein-free. CHO 3D11(\*) cells which had been engineered to express a humanised antibody, CAMPATH(\*)-1H, were routinely grown using serum-containing medium. From a seeding density of 10(5) cells ml(-1), cells grown in static culture with serum reached a maximal cell density of 6.5x10(5) cells ml(-1) after 6 days in culture and produced a maximal antibody concentration of 69 mg l(-1) after 11 days in culture. CHO 3D11(\*) cells grown with serum were washed in serum-free medium then cultured in WCM5 medium. Following a period of adaptation the cell growth and product yield was superior to that achieved with serum-containing medium. CHO cells producing CAMPATH-1H grown in an 8000 l stirred bioreactor seeded with 2x10(5) cells ml(-1) reached a maximal viable cell density of 2.16x10(6) cells ml(-1) after 108 h in culture and a maximal antibody concentration of 131.1 mg l(-1) after 122 h in culture.

859 ||| 21425253 ||| (6, 0.51) ||| 6 ||| 7 ||| Effect of chemical chaperone addition on production and aggregation of recombinant flag-tagged COMP-angiopoietin 1 in Chinese hamster ovary cells. To investigate the effect of chemical chaperones on the production and aggregation of flag-tagged cartilage oligomeric matrix protein-Angiopoietin1 (FCA1) in recombinant Chinese hamster ovary (CHO) cells, CHO cells were cultivated in serum-free media with various chemical chaperones, 1 mM 4-phenylbutyrate (4-PBA), 200 mM proline, 3% glycerol, 2% dimethyl sulfoxide (DMSO), and without chemical chaperone as control. The addition of chemical chaperones enhanced FCA1 production and specific FCA1 productivity, q(FCA)(1). For example, the q(FCA)(1) at 200 mM proline was fourfold higher than that at control. Unlike q(FCA)(1), the aggregation of FCA1 was strongly affected by which chemical chaperone was added. The addition of 2% DMSO and 200 mM proline significantly reduced the proportion of aggregates, but the addition of 1 mM 4-PBA and 3% glycerol was hardly effective. The proportions of aggregates were 29.5 and 55.6% at 2% DMSO and 200 mM proline, respectively, whereas it was 79.6% at control. The exact mechanism how chemical chaperones affected the aggregation of FCA1 was not investigated in this study, and therefore, more extensive works will be needed to clarify why different chemical chaperones behaved differently in reducing the aggregation of FCA1. Among chemical chaperones tested, DMSO was the most effective one in regard to enhancing the production and reducing the aggregation of FCA1 in CHO cells.

860 ||| 19003023 ||| (6, 0.51) ||| 6 ||| 7 ||| Enhanced recombinant M-CSF production in CHO cells by glycerol addition: model and validation. Addition of stimulatory chemical such as glycerol was found to increase recombinant protein production in Chinese hamster ovary (CHO) cells. However, glycerol influenced cell mitosis and reduced cell growth rate. We developed a controlled proliferation strategy to utilize the stimulation of glycerol on recombinant protein production and mitigate the problem of growth inhibition. The approach is to apply a two-stage process, where cells are cultured without glycerol for a period of time in order to obtain enough cell density and then glycerol is added to achieve high specific productivity. In addition, a model for predicting the profiles of cell proliferation and recombinant protein production was developed and validated. A two-stage process, addition of 1% glycerol after 1 day of growth, could increase the final production of macrophage-colony stimulating factor (M-CSF) by 38% compared with the value obtained without addition of glycerol.

861 ||| 17608629 ||| (6, 0.51) ||| 6 ||| 7 ||| Overexpression of cold-inducible RNA-binding protein increases interferon-gamma production in Chinese-hamster ovary cells. Culturing recombinant CHO (Chinese-hamster ovary) cells at low temperatures (30-33 degrees C) increases specific recombinant protein productivity by 2-5-fold. However, even though the specific productivity is increased, cell growth is decreased in low-temperature culture such that the final recombinant protein titre remains unchanged or is even diminished, owing to the lower cell density. Exposing mammalian cells to low temperatures results in a change in the expression of many 'cold-stress' genes. CIRP (cold-inducible RNA-binding protein) is a cold-stress protein that is highly expressed at 32 degrees C, but not at 37 degrees C. In the present study we demonstrated that overexpression of CIRP at 37 degrees C can increase the recombinant-protein titre in CHO cells. Stable overexpression of CIRP at 37 degrees C improved the final titre of CHO IFN-gamma, a recombinant CHO cell line producing human IFN-gamma (interferon-gamma), by 25% in adherent culture and up to 40% in suspension culture. Real-time PCR analysis showed that the increase in the recombinant IFN-gamma titre could be attributed to increased recombinant IFN-gamma mRNA levels, while growth data showed that CIRP overexpression did not result in growth arrest in CHO IFN-gamma cells. Glycan analysis showed that the increase in IFN-gamma titre as a result of CIRP overexpression did not affect the site occupancy, glycan structures or sialic acid content of IFN-gamma. Using this strategy, the final IFN-gamma titre was increased by 40% compared with current temperature-based strategies. Furthermore, there is no decrease in cell growth or recombinant-protein glycosylation quality, as previously observed in low-temperature culture.

862 ||| 23125190 ||| (6, 0.50) ||| 6 ||| 7 ||| Fed-batch CHO cell t-PA production and feed glutamine replacement to reduce ammonia production. Industrial therapeutic protein production has been greatly improved through fed-batch development. In this study, improvement to the productivity of a tissue-plasminogen activator (t-PA) expressing Chinese hamster ovary (CHO) cell line was investigated in shake flask culture through the optimization of the fed-batch feed and the reduction of ammonia generation by glutamine replacement. The t-PA titer was increased from 33 mg/L under batch conditions to 250 mg/L with daily feeding starting after three days of culture. A commercially available fed-batch feed was supplemented with cotton seed hydrolysate and the four depleted amino acids, aspartic acid, asparagine, cysteine, and tyrosine. The fed-batch operation increased the generation of by-products such as lactate and ammonia that can adversely affect the fed-batch performance. To reduce the ammonia production, a glutamine-containing dipeptide, pyruvate, glutamate, and wheat gluten hydrolysate, were investigated as glutamine substitutes. To minimize the lag phase as the cells adjusted to the new energy source, a feed glutamine replacement process was developed where the cells were initially cultured with a glutamine containing basal medium to establish cell growth followed by feeding with a feed containing the glutamine substitutes. This two-step feed glutamine replacement process not only reduced the ammonia levels by over 45% but, in the case of using wheat gluten hydrolysate, almost doubled the t-PA titer to over 420 mg/L without compromising the t-PA product quality or glycosylation pattern. The feed glutamine replacement process combined with optimizing other feed medium components provided a simple, practical, and effective fed-batch strategy that could be applied to the production of other recombinant therapeutic proteins.

863 ||| 19412658 ||| (6, 0.50) ||| 6 ||| 7 ||| The effects of microcarrier culture on recombinant CHO cells under biphasic hypothermic culture conditions. A Chinese hamster ovary (CHO) cell line, producing recombinant secreted human placental alkaline phosphatase (SEAP) was investigated under three different culture conditions (suspension cells, cells attached to Cytodex 3 and Cytopore 1 microcarriers) in a biphasic culture mode using a temperature shift to mild hypothermic conditions (33 degrees C) in a fed-batch bioreactor. The cell viability in both the suspension and the Cytodex 3 cultures was maintained for significantly longer periods under hypothermic conditions than in the single-temperature cultures, leading to higher integrated viable cell densities. For all culture conditions, the specific productivity of SEAP increased after the temperature reduction; the specific productivities of the microcarrier cultures increased approximately threefold while the specific productivity of the suspension culture increased nearly eightfold. The glucose and glutamine consumption rates and lactate and ammonia production rates were significantly lowered after the temperature reduction, as were the yields of lactate from glucose. However, the yield of ammonia from glutamine increased in response to the temperature shift.

864 ||| 18636451 ||| (6, 0.50) ||| 6 ||| 7 ||| Increasing GPI-anchored protein harvest concentrations from suspension and porous microcarrier CHO cell cultures. Chinese hamster ovary (CHO) cells expressing the human melanoma tumour antigen, p97, were used to develop a controlled release process for the production of recombinant glycosyl-phosphatidylinositol (GPI) anchored proteins. The cells were cultured either in suspension or immobilized on porous microcarriers and p97 was selectively cleaved from the cell surface by the bacterial enzyme, phosphatidylinositol-phospholipase C (PI-PLC). The kinetics of p97 cleavage from the cell surface by PI-PLC was shown to be approximated by Michaelis-Menten kinetics. The recovered p97 concentrations were increased by reusing the PI-PLC enzyme solution to harvest multiple batches of cells. A convenient PI-PLC assay was developed to monitor the harvesting process and to determine the stability of PI-PLC under harvesting conditions. Although the Pl-PLC was stable under harvesting conditions, it rapidly adsorbed to the cell surface and was depleted from the reused enzyme solution. In order to maintain PI-PLC activity, it was necessary to add fresh PI-PLC to the reused enzyme solution before harvesting a fresh batch of cells. The maximum p97 concentration that could be obtained from harvesting CHO cells cultured on porous microcarriers was limited by the dilution effects of sample removal, adding fresh PI-PLC and liquid associated with settled microcarriers. A model was developed that adequately predicted the p97 concentration after each harvest and the maximum p97 concentration that could be achieved by this harvesting method. The dilution effects were minimized by harvesting from centrifuged suspension culture cells and the harvested p97 concentration was increased by over sixfold to 0.64 mg/mL.

865 ||| 22535835 ||| (6, 0.49),(1, 0.42) ||| 6 ||| 7 ||| Effect of hydrocortisone on the production and glycosylation of an Fc-fusion protein in CHO cell cultures. Glucocorticoids are known to modulate various cellular functions such as cell proliferation, metabolism, glycosylation, and secretion of many proteins. We tested the effect of hydrocortisone (HC) on cell growth, viability, metabolism, protein production, and glycosylation of an Fc-protein expressing Chinese hamster ovary (CHO) cell culture. HC extended cell viability but impaired cell growth. The inhibitory effect on cell growth was dose-dependent and decreased when the glucocorticoid addition was delayed. When HC was added after 2 or 3 days of culture, an increase in glutamate consumption was observed, which was reversed by the glucocorticoid receptor antagonist mifepristone (Mif). Titer and specific productivity increased in the presence of HC. The increase in titer was only slightly reversed by Mif. On the other hand, Mif by itself induced an increase in titer to a level comparable to or higher than HC. Protein glycosylation was altered by the glucocorticoid in a dose- and time-dependent manner, with a shift to more acidic bands, which correlated with an increase in sialic acid moieties. This increase, which was not linked to a decrease in extracellular sialidase activity in HC-treated cultures, was reversed by Mif. Predictive models based on design of experiments enabled the definition of optimal conditions for process performance in terms of viability and titer and for the quality of the Fc-fusion protein in terms of glycosylation. The data obtained suggest a use of glucocorticoids for commercial production of Fc-fusion proteins expressed in CHO cells.

866 ||| 9222815 ||| (6, 0.49) ||| 6 ||| 7 ||| Bicarbonate-dependent proton extrusion in Chinese hamster ovary (CHO) cells adapted to growth at pH 6.7. A CHO cell model is described for in vitro studies of the mechanisms underlying heat resistance in cells adapted to growth in acidic environments. Adaptation is defined as a loss of pH 6.7-induced sensitization to 42.0 degrees C cytotoxicity and it is accompanied with an elevation of steady-state intracellular pH (pHi). CHO cells cultured between 75 and 100 days at pH 6.7 became fully adapted (6.7G cells), and the adapted phenotype was maintained for at least 100 additional days of culture at pH 6.7. The surviving fraction (SF) of 6.7G cells heated (42.0 degrees C) at pH 6.7 was comparable with that of cells cultured at pH 7.3 (7.3G cells) and heated at pH 7.3, while the SF of 7.3G cells acutely acidified to pH 6.7 and heated was an order of magnitude less. Although this resistance of 6.7G cells to killing was observed at 42.0 degrees C, it was not observed at 43.0 and 45.0 degrees C. Both 6.7G and 7.3G cells were able to develop comparable levels of thermotolerance during 42.0 degrees C at their growth pHs. However, in agreement with the literature, development of thermotolerance was reduced in acutely acidified 7.3G cells. An acute acidification of only 0.2 pH unit from pH 6.7 to 6.5 also reduced the ability of 6.7G cells to develop thermotolerance during heating at 42.0 degrees C. The acquired 6.7G phenotype reverted to the 7.3G phenotype following 17 days of culture at pH 7.3. Amiloride (0.5 mM), an inhibitor of the Na+/H+ exchanger (NHE), did not sensitize 7.3G and 6.7G cells to 42.0 degrees at their growth pHs. However, sensitization was observed for acutely acidified 7.3G cells. This is consistent with the hypothesis that extracellular acute acidification causes a decrease in pHi, and that the recovery from that decrease is achieved in part by activation of the NHE. An elevation of steady-state pHi, measured by analysing intracellular BCECF excitation spectra, was documented in a suspension assay for cells grown at pH 6.7 for 180 days. The elevation was bicarbonate-dependent (negligible in the absence of HCO3-, +0.17 pH units in the presence of HCO3-). These results suggest that the altered regulation of pHi in CHO cells adapted to pHe 6.7 is maintained by bicarbonate-dependent processes.

867 ||| 24721212 ||| (6, 0.49) ||| 6 ||| 7 ||| Effect of glutamine substitution by TCA cycle intermediates on the production and sialylation of Fc-fusion protein in Chinese hamster ovary cell culture. In an effort to reduce the accumulation of ammonia in culture medium, three different TCA cycle intermediates, (alpha-ketoglutarate (alpha-KG), citric acid and succinic acid) along with glutamic acid for a comparison, were examined as a substitute for glutamine with rCHO cell line producing a Fc-fusion glycoprotein. Among them, alpha-KG showed the best production performance. When cells were cultivated with 4 mM alpha-KG, the final ammonia concentration did not exceed 3 mM, which is less than one fourth of that with 4 mM glutamine. The replacement of glutamine increased the lag phase and reduced cell growth. However, it increased the specific productivity by 2.7-fold, resulting in a 1.3-fold increase in the maximum product concentration. Furthermore, the sialic acid content of the Fc-fusion protein with 4 mM alpha-KG was higher than that with 4 mM glutamine in all cultures, most likely due to the lower ammonia concentration. The results of Western blotting and activity assays of intracellular alpha-2,3-sialyltransferase and extracellular sialidases are in good agreement with tests done to assess the sialic acid content of the Fc-fusion protein. Taken together, the data obtained here demonstrate that alpha-KG is a potential substitute for glutamine for improved glycoprotein production in rCHO cells.

868 ||| 24846804 ||| (6, 0.48),(1, 0.33) ||| 6 ||| 7 ||| The bioactivity and fractionation of peptide hydrolysates in cultures of CHO cells. Peptide hydrolysate supplements in mammalian cell cultures provide enhanced growth and productivity. The objective of this study was to compare the bioactivity of ten different commercially available hydrolysates from plant, microbial, and animal sources. The peptide hydrolysates were tested as supplements to cultures of Chinese hamster ovary (CHO) cells that produce human beta interferon (beta-IFN). A soy hydrolysate was shown to support high cell growth but not protein productivity compared to an animal component hydrolysate (Primatone RL). On the other hand, a yeast hydrolysate showed lower cell growth, but comparable productivity of the recombinant protein. Glycosylation analysis showed that the glycan profile of beta-IFN produced in yeast hydrolysate supplemented cultures was equivalent to that from Primatone RL-supplemented cultures. Fractionation of the yeast hydrolysate and Primatone RL produced a similar protein-assayed pattern except for one extra peak at around 1 kDa in the Primatone RL profile. A fraction taken at a molecular weight range of 1.5-1.7 kDa showed the highest growth promoting activity in both samples. However, four other fractions in yeast hydrolysate and two in Primatone RL at lower molecular weights showed some growth promoting activity. In conclusion, the yeast hydrolysates provided a good alternative to the animal sourced Primatone RL for high productivity of beta-IFN from CHO cells with equivalent glycosylation.

869 ||| 23247406 ||| (6, 0.48) ||| 6 ||| 7 ||| The impact of cell adaptation to serum-free conditions on the glycosylation profile of a monoclonal antibody produced by Chinese hamster ovary cells. N-glycosylation is one of the most crucial parameters affecting the biological activity of therapeutic monoclonal antibodies (mAbs), and should therefore be closely monitored and controlled to guarantee a consistent and high-quality product in biopharmaceutical processes. In the present work, the effect of the time-consuming step of gradual cell adaptation to serum-free conditions on the glycosylation profile of a mAb produced by CHO-K1 cells was evaluated. High-performance liquid chromatography analysis revealed important changes in mAb glycosylation patterns in all steps of serum reduction. These changes could be grouped in two distinct phases of the process of adaptation: middle (2.5 to 0.15% serum) and final (0.075 and 0% serum). For intermediate levels of serum, a desirable increase of galactosylation and decrease of fucosylation, but an undesirable increase in sialylation were observed; while the inverse was obtained at the final stages of adaptation. These divergences may be related to the reduction of serum supplementation, to variations in the levels of cell density and viability achieved at these stages, and to the natural shift of the cell growth mode during adaptation from adherent to suspended. The divergent glycan profiles obtained in this study demonstrate a strong influence of the adaptation process on mAb glycosylation, suggesting that control and monitoring of product quality should be implemented at the early stages of process development.

870 ||| 20933553 ||| (6, 0.48) ||| 6 ||| 7 ||| High productivity of human recombinant beta-interferon from a low-temperature perfusion culture. Recombinant human interferon-beta (beta-IFN), used in the therapeutic treatment of multiple sclerosis (MS), can be produced on a large-scale from genetically engineered Chinese hamster ovary (CHO) cells. However, its hydrophobicity causes non-reversible, molecular aggregation in culture. The parameters affecting aggregation were determined to be concentration, culture residence time, temperature and glycosylation. Although the protein can be produced in Escherichia coli in a non-glycosylated form, the addition of glycans confers a reduced rate of aggregation as well as a 10-fold higher bioactivity. We report on the application of a low temperature perfusion culture designed to control the parameters that cause aggregation. In this three-phase culture system there is a transition to a low temperature (32 C) in a batch mode prior to implementing perfusion at 1 volume/day using an acoustic cell separator. Perfusion at the low temperature resulted in a 3.5-fold increase in specific productivity and a 7-fold increase in volumetric productivity compared to the batch culture at 37 C. The percentage aggregation of beta-IFN was reduced from a maximum of 43% in batch culture to a minimum of 5% toward the end of the perfusion phase. The glycosylation profile of all samples showed predominantly sialylated biantennary fucosylated structures. The extent of sialylation, which is important for bioactivity, was enhanced significantly in the perfusion culture, compared to the batch culture.

871 ||| 8987482 ||| (6, 0.47) ||| 6 ||| 7 ||| Sialidase activity in culture fluid of Chinese hamster ovary cells during batch culture and its effects on recombinant human antithrombin III integrity. Sialidase activity in cell-free supernatant of batch-cultivated Chinese hamster ovary (CHO) cells producing human recombinant antithrombin III (rhAT III) was monitored during cultivation using 4-methylumbelliferyl substrate and HPLC for free sialic acid determination. Supernatant sialidase as well as lactate dehydrogenase activity increased significantly during batch growth. The enhanced number of dead cells correlated with increasing sialidase activity which seemed to be principally due to cell lysis, resulting in release of cytosolic sialidase. Loss of terminally alpha (2-->3) bound sialic acids of the oligosaccharides of rhAT III was analyzed in lectin-based Western blot and enzyme-linked lectin assays, using Maackia amurensis and Datura stramonium agglutinins for specific determination of Neu5Ac alpha (2-->3)Gal- and Gal beta (1-->4)-GlcNAc-terminated glycoproteins, respectively. Results show a remarkable loss of terminal sialic acids of rhAT III along with decrease in CHO cell viability and concomitant increase of dead cells throughout long-term batch cultivation. To avoid this degradation effect, process parameters forcing high viability are essential and harvesting of culture at an early time even at suboptimal recombinant protein concentration is highly recommended to avoid product desialylation.

872 ||| 10835230 ||| (6, 0.46) ||| 6 ||| 7 ||| The growth factor inhibitor suramin reduces apoptosis and cell aggregation in protein-free CHO cell batch cultures. We have previously shown that Chinese hamster ovary (CHO) cells capable of growing in medium free of exogenous proteins die by apoptosis during all stages of a batch culture (Zanghi et al., 1999). On the basis of the hypothesis that extracellular death factors might be important in apoptosis under these conditions, we examined the effect of the growth factor inhibitor and antitumor agent suramin on CHO cell growth and apoptosis in serum-free culture. Suramin protected against apoptosis during exponential growth, as indicated by the absence of DNA laddering and an increase in cell viability from roughly 70% to above 95%. Suramin also effectively dispersed cell aggregates so that single-cell suspension culture was possible. However, suramin did not protect against apoptosis during the death phase, in contrast to serum, suggesting that antiapoptotic factors in the serum remain to be discovered. The increased viable cell yield following suramin supplementation resulted in a 40% increase in product yield, based on results with cells expressing recombinant secreted alkaline phosphatase. Polysulfated compounds dextran sulfate and polyvinyl sulfate worked nearly as well as suramin in dispersing cell clumps and increasing viable cell yield, which implies that suramin's high sulfate group density may be responsible for its effects in cell culture. In addition, suramin was beneficial for long-term adaptation of CHO cells to protein-free media suspension culture, and the compound was synergistic with insulin in accelerating this adaptation time.

873 ||| 15504104 ||| (6, 0.45),(5, 0.37) ||| 6 ||| 7 ||| Active hypothermic growth: a novel means for increasing total interferon-gamma production by Chinese-hamster ovary cells. When grown under hypothermic conditions, CHO (Chinese-hamster ovary) cells become growth-arrested in the G0/G1 phase of the cell cycle and also often exhibit increased recombinant-protein production. We have shown in the accompanying paper [Fox, Tan, Tan, Wong, Yap and Wang (2005) Biotechnol. Appl. Biochem. 41, 255-264] that the positive effect of low temperature on recombinant-protein production is due to elevated mRNA levels and not due to G0/G1-phase growth arrest and that a cell line can still show growth-associated productivity at low temperature. This finding led to the hypothesis that improved total production of recombinant protein would be achieved by stimulating cells to actively grow at low temperature, a culture condition previously unreported in the literature. In the present study we have validated this hypothesis by stimulating hypothermic (32 degrees C) growth through the use of different growth factors. Hypothermic growth was stimulated in fetal-bovine-serum-supplemented adherent cultures using basic fibroblast growth factor or insulin. Hypothermic growth was also stimulated in suspension cultures normally grown in protein-free medium by using supplementation with fetal bovine serum. These methods resulted in up to 7.7- and 4.9-fold increases in total recombinant-protein production compared with the 37 and 32 degrees C control cultures respectively. This proof-of-concept study will motivate the creation of cell lines capable of growing at low temperatures for use in industrial processes.

874 ||| 22692846 ||| (6, 0.45),(3, 0.30) ||| 6 ||| 7 ||| Laboratory production of human prolactin from CHO cells adapted to serum-free suspension culture. Human prolactin (hPRL) is a polypeptide with 199 amino acids and a molecular mass of 23 kDa. Previously, a eukaryotic hPRL expression vector was used to transfect Chinese hamster ovary (CHO) cells: this work describes a fast and practical laboratory adaptation of these transfected cells, in ~40 days, to grow in suspension in serum-free medium. High cell densities of up to 4.0 x 10(6) cell/ml were obtained from spinner flask cultures and a stable and continuous production process was developed for at least 30 days. Two harvesting strategies were set up, 50 or 100 % of the total conditioned medium being collected daily and replaced by fresh culture medium. The volumetric productivity was 5-7 mug hPRL/ml, as determined directly in the collected medium via reversed-phase HPLC (RP-HPLC). A two-step process based on a cationic exchanger followed by size exclusion chromatography was applied to obtain purified hPRL from conditioned medium. Two hPRL isoforms, non-glycosylated and glycosylated, could also be separated by high-performance size-exclusion chromatography (HPSEC) and, when analyzed by RP-HPLC, HPSEC, Western blotting, and bioassay, were found to be comparable to the World Health Organization International Reference Reagent of hPRL. These results are useful for the practical scale-up to the pilot and industrial scale of a bioprocess based on CHO cell culture.

875 ||| 22275108 ||| (6, 0.45) ||| 6 ||| 7 ||| Effect of process parameters and product-host-interaction on hVEGFA-production by recombinant Chinese hamster ovary cells. A potential producer clone was identified among recombinant, human vascular endothelial growth factor A (hVEGFA)-producing Chinese Hamster Ovary (CHO) K1 cells, using a recently established screening method. In batch spinner cultivations, the cells showed a maximum growth rate of 0.045 h(-1), a final total cell density of 5.3x10(6) mL(-1) (living cell density: 3.4x10(6) mL(-1)), and a final hVEGFA concentration of 207 mug L(-1). Living cell density and productivity in the spinner cultivations could be increased by glutamine feeding. Transfer of the process to the bioreactor (batch mode, control of pH, T, and O2) resulted in a reduction of the growth rate by roughly 50%, while overall living cell density and productivity increased, largely due to an extension of the production phase. When the bioreactor was run in the fed-batch mode, growth rates were further reduced, while productivity and living cell densities reached a maximum (hVEGFA: 358 mug L(-1), cells: 5.2x10(6) mL(-1)). In addition, the death rate of the hVEGFA-producing cells was considerably reduced compared with the parent cell line, most likely due to product-host-interaction. This hypothesis was corroborated when a second recombinant CHO cell line (antibody producer) was transfected with the hVEGFA gene and afterward consistently showed higher viable cell densities together with a significantly improved antibody titer.

876 ||| 24728980 ||| (6, 0.44),(8, 0.33) ||| 6 ||| 7 ||| Identification of manipulated variables for a glycosylation control strategy. N-linked glycan distribution affects important end-use characteristics such as the bioactivity and efficacy of many therapeutic proteins, (including monoclonal antibodies), in vivo. Yet, obtaining desired glycan distributions consistently during batch-to-batch production can be challenging for biopharmaceutical manufacturers. While an appropriately implemented on-line glycosylation control strategy during production can help to ensure a consistent glycan distribution, to date no such strategies have been reported. Our goal is to develop and validate a comprehensive strategy for effective on-line control of glycosylation, the successful achievement of which requires first identifying appropriate manipulated variables that can be used to direct the glycan distribution to a desired state. While various culture conditions such as bioreactor process variables, media type, and media supplements have been shown to affect the glycan distribution, in this study we focus on the latter. Specifically, we implemented a statistically designed series of experiments to determine the significant main effects (as well as interaction effects) of media supplementation with manganese, galactose, ammonia and found that each had significant effects on certain glycans. We also include data indicating the glycosylation enzyme gene transcript levels as well as the intracellular nucleotide sugar concentrations in the presence of the media supplements to provide insight into the intracellular conditions that may be contributing to the changes in glycan distribution. The acquired experimental data sets were then used to identify which glycans can be controlled by the media supplements and to what degree. We determined that MnCl2 can be used as a manipulated variable to increase the relative abundance of M51 and decrease FA2 simultaneously, and galactose can be used as a manipulated variable to increase the relative abundance of FA2G1 and decrease FA2 and A2 simultaneously.

877 ||| 9841644 ||| (6, 0.44),(5, 0.41) ||| 6 ||| 7 ||| Role of nucleotide sugar pools in the inhibition of NCAM polysialylation by ammonia. Ammonia in animal cell cultures has been shown to specifically inhibit terminal sialylation of N- and O-linked oligosaccharides of glycoproteins. For example, we have previously shown that as little as 2.5 mM NH4Cl can decrease neural cell adhesion molecule (NCAM) polysialylation in both small cell lung cancer (SCLC) and Chinese hamster ovary (CHO) cells. Besides its potential involvement in SCLC metastasis, polysialic acid (PolySia) is a sensitive marker for measuring changes in sialylation. The role of UDP-N-acetylglucosamine (UDP-GlcNAc) in ammonia's inhibition of NCAM polysialylation was examined by adding glucosamine (GlcN) and uridine (Urd) to the cultures. This bypassed feedback inhibition of GlcN-6-P synthase and increased UDP-GlcNAc content by 25-fold in SCLC cells. After 3 days, PolySia levels were reduced to 10% of control with little effect on NCAM protein content. The extensive decrease in PolySia was confirmed in CHO cells. The effects of GlcN or Urd alone were less extensive, lending support to a specific role for UDP-GlcNAc in inhibition by ammonia. By comparison, 20 mM NH4Cl decreased PolySia content by 45% and increased UDP-GlcNAc in SCLC cells by 2-fold. The discrepancy between the ?GlcN+Urd? and NH4Cl effects on UDP-GlcNAc and PolySia suggests that accumulation of UDP-GlcNAc is only partially responsible for decreased polysialylation in response to NH4Cl. In an attempt to increase NCAM polysialylation, N-acetylmannosamine and cytidine were added to cultures in order to circumvent the feedback inhibition of CMP-sialic acid synthesis. However, this only slightly increased PolySia levels and failed to counter ammonia's inhibition of NCAM polysialylation.

878 ||| 20683638 ||| (6, 0.44) ||| 6 ||| 7 ||| Enhanced production and isotope enrichment of recombinant glycoproteins produced in cultured mammalian cells. NMR studies of post-translationally modified proteins are complicated by the lack of an efficient method to produce isotope enriched recombinant proteins in cultured mammalian cells. We show that reducing the glucose concentration and substituting glutamate for glutamine in serum-free medium increased cell viability while simultaneously increasing recombinant protein yield and the enrichment of non-essential amino acids compared to culture in unmodified, serum-free medium. Adding dichloroacetate, a pyruvate dehydrogenase kinase inhibitor, further improves cell viability, recombinant protein yield, and isotope enrichment. We demonstrate the method by producing partially enriched recombinant Thy1 glycoprotein from Lec1 Chinese hamster ovary (CHO) cells using U-13C-glucose and 15N-glutamate as labeled precursors. This study suggests that uniformly 15N,13C-labeled recombinant proteins may be produced in cultured mammalian cells starting from a mixture of labeled essential amino acids, glucose, and glutamate.

879 ||| 18553404 ||| (6, 0.44) ||| 6 ||| 7 ||| The effects of culture conditions on the glycosylation of secreted human placental alkaline phosphatase produced in Chinese hamster ovary cells. The effects of different culture conditions, suspension and microcarrier culture and temperature reduction on the structures of N-linked glycans attached to secreted human placental alkaline phosphatase (SEAP) were investigated for CHO cells grown in a controlled bioreactor. Both mass spectrometry and anion-exchange chromatography were used to probe the N-linked glycan structures and distribution. Complex-type glycans were the dominant structures with small amounts of high mannose glycans observed in suspension and reduced temperature cultures. Biantennary glycans were the most common structures detected by mass spectrometry, but triantennary and tetraantennary forms were also detected. The amount of sialic acid present was relatively low, approximately 0.4 mol sialic acid/mol SEAP for suspension cultures. Microcarrier cultures exhibited a decrease in productivity compared with suspension culture due to a decrease in both maximum viable cell density (15-20%) and specific productivity (30-50%). In contrast, a biphasic suspension culture in which the temperature was reduced at the beginning of the stationary phase from 37 to 33 degrees C, showed a 7% increase in maximum viable cell density, a 62% increase in integrated viable cell density, and a 133% increase in specific productivity, leading to greater than threefold increase in total productivity. Both microcarrier and reduced temperature cultures showed increased sialylation and decreased fucosylation when compared to suspension culture. Our results highlight the importance of glycoform analysis after process modification as even subtle changes (e.g., changing from one microcarrier to another) may affect glycan distributions.

880 ||| 16232793 ||| (6, 0.44) ||| 6 ||| 7 ||| Aggregate formation of rCHO cells and its maintenance in repeated batch culture in the absence of cell adhesion materials. Aggregate formation of recombinant Chinese hamster ovary (rCHO) cells capable of producing granulocyte colony-stimulating factor (G-CSF), using medium lacking cell adhesion materials in a repeated batch culture, was examined together with cell growth, cell viability and G-CSF production. The rCHO culture was conducted in a rotary shaker and the medium was changed every five days. The formation of stable cell aggregates with high reproducibility was observed after the first medium change. The size of the cell aggregates (consisting of several 10s to 40,000 cells) formed during the repeated batch culture ranged from 30 to 600 microm. The cell density of the aggregates reached as high as 2 x 10(6) cells/ml and the viability was maintained at more than 80% for 19 d. Changing the medium to avoid glucose exhaustion effectively maintained the cell density, cell viability and G-CSF productivity at high levels.

881 ||| 15903237 ||| (6, 0.44) ||| 6 ||| 7 ||| Enhanced production of monomeric interferon-beta by CHO cells through the control of culture conditions. The enhancement of recombinant protein expression of a transfected cell line is essential for the development of an efficient large-scale bioprocess. The effect of various media additives and temperature conditions were studied in an attempt to optimize protein production, stability, and protein glycosylation from a Chinese hamster ovary (CHO) cell line producing human beta-interferon (Hu-beta-IFN). We observed a decrease in the ELISA response of the glycoprotein in the later stages of batch cultures, which was attributed to molecular aggregation. Cells were subjected to various concentrations of glycerol, dimethyl sulfoxide (DMSO), and sodium butyrate (NaBu) in a variety of culture systems and conditions. The addition of both NaBu and DMSO resulted in higher specific productivities but reduced growth rates that resulted in a net reduction of interferon produced. Glycerol appeared to stabilize the secreted beta-IFN, resulting in reduced aggregation, despite a decrease in cell growth rate. Glycosylation analysis of isolated beta-IFN showed a time-dependent decrease in sialylation in batch culture that was ameliorated by the presence of glycerol. Low-temperature conditions (30 degrees C) had the greatest effect on productivity with a significant increase in beta-IFN titer as well as a reduction in the degree of molecular aggregation.

882 ||| 10222248 ||| (6, 0.44) ||| 6 ||| 7 ||| Aberrant metabolic sialylation of recombinant proteins expressed in Chinese hamster ovary cells in high productivity cultures. The incorporation of sialic acid into therapeutic recombinant glycoprotein expressed in Chinese hamster ovary (CHO) cells during growth in large bioreactors (10 l) has been monitored under high productivity conditions induced by the presence of sodium butyrate. Samples of the bioreactor culture (approximately 4 x 10(6) cells) were labeled with 3H-N-acetylmannosamine, a metabolic precursor of sialic acid. After 24 h, the recombinant glycoprotein, an immunoadhesion chimeric molecule, was purified and the amount of sialic acid incorporated was determined as radioactive counts. The labeling profile of the protein over the course of the culture was compared with the sialic acid content of the molecule as determined by direct chemical analysis. Early in the culture, the two methods of analysis gave a similar sialylation profile. However, after sodium butyrate was included in the culture, the metabolically incorporated sialic acid rapidly and dramatically decreased to near undetectable levels. In contrast, sialic acid content of the protein, as determined by chemical analysis, decreased only moderately and gradually over the culture period, from a maximum of 6.1 to about 5. 0 mol sialic acid/mole of protein after 10 days in culture. These results suggest that butyrate may enhance reutilization of existing glycoproteins in the culture, generating sialic acid for biosynthesis through lysosomal degradation and thereby bypassing de novo biosynthesis.

883 ||| 22205442 ||| (6, 0.43),(3, 0.39) ||| 6 ||| 7 ||| Enhancement of sialylation on humanized IgG-like bispecific antibody by overexpression of alpha2,6-sialyltransferase derived from Chinese hamster ovary cells. Improvement of glycosylation is one of the most important topics in the industrial production of therapeutic antibodies. We have focused on terminal sialylation with alpha-2,6 linkage, which is crucial for anti-inflammatory activity. In the present study, we have successfully cloned cDNA of beta-galactosyl alpha-2,6 sialyltransferase (ST6Gal I) derived from Chinese hamster ovary (CHO) cells regardless of reports that stated this was not endogenously expressed in CHO cells. After expressing cloned ST6Gal I in Escherichia coli, the transferase activity was confirmed by HPLC and lectin binding assay. Then, we applied ST6Gal I to alpha-2,6 sialylation of the recombinant antibody; the ST6Gal I expression vector was transfected into the CHO cell line producing a bispecific antibody. The N-glycosylation pattern of the antibody was estimated by HPLC and sialidase digestion. About 70% of the total N-linked oligosaccharide was alpha-2,6 sialylated in the transfected cell line whereas no sialylation was observed in the non-transfected cell line. The improvement of sialylation would be of practical importance for the industrial production of therapeutic antibodies.

884 ||| 11005924 ||| (6, 0.42),(7, 0.30) ||| 6 ||| 7 ||| Effect of insulin stimulation on the proliferation and death of Chinese hamster ovary cells. The effect of environmental and genetic parameters on cell death was studied in Chinese hamster ovary cell cultures. Experiments were performed using an anchorage-dependent CHO cell line expressing gamma-IFN, and a second cell line obtained by transfection of the previous one with bcl-2. In serum-free medium the two cell lines showed a considerable degree of growth control entering quiescence while maintaining high viabilities. The addition of transferrin did not have any effect but insulin addition allowed cells arrested by serum withdrawal to reenter the cell cycle. However, insulin supplementation also resulted in cell death, which was possible to avoid through bcl-2 overexpression or in the presence of serum. We propose that the expression of c-myc, shown to be induced by insulin, plays an important role in the cell death observed after insulin addition in an inappropriate environment, deficient in protective factors. This hypothesis is supported by measurements of c-myc expression and cell cycle distribution.

885 ||| 10099468 ||| (6, 0.42),(3, 0.37) ||| 6 ||| 7 ||| Monitoring recombinant human interferon-gamma N-glycosylation during perfused fluidized-bed and stirred-tank batch culture of CHO cells. Chinese hamster ovary cells producing recombinant human interferon-gamma were cultivated for 500 h attached to macroporous microcarriers in a perfused, fluidized-bed bioreactor, reaching a maximum cell density in excess of 3 x 10(7) cells (mL microcarrier)-1 at a specific growth rate (mu) of 0.010 h-1. During establishment of the culture, the N-glycosylation of secreted recombinant IFN-gamma was monitored by capillary electrophoresis of intact IFN-gamma proteins and by HPLC analysis of released N-glycans. Rapid analysis of IFN-gamma by micellar electrokinetic capillary chromatography resolved the three glycosylation site occupancy variants of recombinant IFN-gamma (two Asn sites occupied, one Asn site occupied and nonglycosylated) in under 10 min per sample; the relative proportions of these variants remained constant during culture. Analysis of IFN-gamma by capillary isoelectric focusing resolved at least 11 differently sialylated glycoforms over a pI range of 3.4 to 6.4, enabling rapid quantitation of this important source of microheterogeneity. During perfusion culture the relative proportion of acidic IFN-gamma proteins increased after 210 h of culture, indicative of an increase in N-glycan sialylation. This was confirmed by cation-exchange HPLC analysis of released, fluorophore-labeled N-glycans, which showed an increase in the proportion of tri- and tetrasialylated N-glycans associated with IFN-gamma during culture, with a concomitant decrease in the proportion of monosialylated and neutral N-glycans. Comparative analyses of IFN-gamma produced by CHO cells in stirred-tank culture showed that N-glycan sialylation was stable until late in culture, when a decline in sialylation coincided with the onset of cell death and lysis. This study demonstrates that different modes of capillary electrophoresis can be employed to rapidly and quantitatively monitor the main sources of glycoprotein variation, and that the culture system and operation may influence the glycosylation of a recombinant glycoprotein.

886 ||| 19269599 ||| (6, 0.42),(1, 0.33) ||| 6 ||| 7 ||| Synergistic effects of basic fibroblast growth factor and insulin on Chinese hamster ovary cells under serum-free conditions. Chinese hamster ovary (CHO) cells are commonly used in the biotechnology industry to produce recombinant proteins. The development of serum-free media in order to maintain mammalian cell viability and reduce cell death in the absence of serum is highly desirable. Our aim was to find new growth factors that could maintain viability and enhance the proliferation of CHO cells under serum-free conditions. Macrophage colony-stimulating factor (M-CSF)-secreting CHO cells were used as a model cell line to evaluate the stimulatory effects of different compounds, including two growth factors, on cell proliferation. Flow cytometry and fluorescence microscopy were used to analyze the cell cycle and monitor apoptotic nuclei during serum-free culture. Among the tested compounds, insulin and basic fibroblast growth factor (bFGF), each used alone, prevented CHO cells from dying and helped maintain cell viability, but cells were unable to re-enter the cell cycle and proliferate. Simultaneous supplementation with insulin and bFGF synergistically promoted the growth of CHO cells and recombinant M-CSF synthesis in basal DMEM/F12 medium. The mitogenic effects of bFGF and insulin under serum-free conditions were confirmed in the CHO-K1 cell line. These results provide useful information for the design and development of serum-free media as well as for antiapoptotic studies of CHO cells under serum-free conditions.

887 ||| 20949270 ||| (6, 0.42) ||| 6 ||| 7 ||| Uniform stable-isotope labeling in mammalian cells: formulation of a cost-effective culture medium. Uniform stable-isotope labeling of mammalian cells is achieved via a novel formulation of a serum-free cell culture medium that is based on stable-isotope-labeled autolysates and lipid extracts of various microbiological origin. Yeast autolysates allow complete replacement of individual amino acids and organic acids in a chemically defined medium (DMEM/F12), enabling a cost-effective formulation of a stable-isotope-labeled culture medium for mammalian cells. In addition, biomass-derived hydrolysates, autolysates, and lipid extracts of various classes of algae were explored as cell culture components, both separately and in combination with yeast autolysates. Optimal autolysate concentrations were established. Such novel medium formulations were tested on mammalian cell lines, often used for recombinant protein production, i.e., Chinese hamster ovary (CHO) and human embryonic kidney (HEK 293). Special attention was paid to the adaptation of these mammalian cell lines to serum-free media. Formulation of the novel proprietary cell culture medium PLIm, based on yeastolates instead of individual amino acids and organic acids, allows a four- to eightfold cost reduction for (15)N and (13)C,(15)N stable-isotope-labeling, respectively, in CHO cells and a three- to sixfold cost reduction in HEK 293 cells. A high level of stable-isotope enrichment of mammalian cells (>90%) was achieved within four passages by complete replacement of carbon and nitrogen sources in the medium with their stable-isotope-labeled analogs. These conditions can be used to more cost-effectively produce labeled recombinant proteins in mammalian cells.

888 ||| 21337325 ||| (6, 0.41),(2, 0.37) ||| 6 ||| 7 ||| Enhancement of recombinant human EPO production and sialylation in chinese hamster ovary cells through Bombyx mori 30Kc19 gene expression. Productivity and sialylation are two important factors for the production of recombinant glycoproteins in mammalian cell culture. In our previous study, we found that silkworm hemolymph increased the sialylation of recombinant secreted human placental alkaline phosphatase in the insect cells, promoted the transfer of sialic acids onto the glycoprotein oligosaccharides in an in vitro asialofetuin sialylation system, and enhanced recombinant protein production in the Chinese hamster ovary (CHO) cells. These beneficial effects were mainly due to the 30K proteins, which consist of five isoforms. Among the 30K proteins, 30Kc19 was determined to be the major component. In this study, the 30Kc19 gene was introduced into a CHO cell line producing recombinant human erythropoietin, and its effects on productivity and sialylation were investigated. The transient expression of 30Kc19 significantly improved the production and sialylation of EPO. A stable cell line containing 30Kc19 was also established to investigate the effect of 30Kc19 gene expression. The stable expression of 30Kc19 increased the production and sialylation by 102.6% and 87.1%, respectively. The enhanced productivity from 30Kc19 expression is believed to occur because the 30Kc19 protein suppresses the loss of mitochondrial membrane potential and consequently improves the generation of intracellular ATP. In addition, the positive effect of 30Kc19 expression on sialylation is believed to be due to its ability to maintain sialyltransferase activity. In conclusion, 30Kc19 expression is a novel approach to improve the production and sialylation of recombinant glycoproteins in CHO cells.

889 ||| 20533076 ||| (6, 0.41) ||| 6 ||| 7 ||| Effect of culture temperature on TNFR-Fc productivity in recombinant glutamine synthetase-chinese hamster ovary cells. Lowering the culture temperature is often useful to improve the production of many recombinant proteins in Chinese hamster ovary (CHO) cells. Batch cultivation of GS-CHO cells expressing TNFR-Fc antibody was therefore carried out at 30, 33.5 and 37 degrees C. TNFR-Fc productivity, q(TNFR-Fc), increased as culture temperature decreased; and the maximum q(TNFR-Fc) was 20 mg/(10(9) cells.day) at 30 degrees C which was three times that at 37 degrees C. Increasing the viable cell density (VCD) to above 2.2 x 10(6) cells/ml, however, decreased the q(TNFR-Fc) at 30 degrees C, which was due to a reduction in transcription of the TNFR-Fc gene. Taken together, lowering temperature can improve q(TNFR-Fc) but the negative effect of increasing VCD compromises this effect. Further process development addressing the issue of cell density-dependent TNFR-Fc productivity is therefore needed.

890 ||| 18296044 ||| (6, 0.40),(3, 0.34) ||| 6 ||| 7 ||| Influence of the rapeseed protein hydrolysis process on CHO cell growth. Different protein hydrolysates were prepared from enzymatic hydrolyses of a rapeseed isolate (>90% protein content) using different commercial enzymes of non-animal origin. The extent of hydrolysis was controlled to produce hydrolysates corresponding to various degrees of hydrolysis (DH) from 5 to 30. These hydrolysates were characterized according to their solubility and size peptide pattern. Different growth behaviours of Chinese Hamster Ovary cells were observed when these various hydrolysates were added in serum-free medium containing transferrin, albumin and insulin. Hydrolysates from low degree of hydrolysis generally did not exhibit significant positive effect on cell growth; conversely hydrolysates from extensive hydrolysis, corresponding to a major low molecular size peptides content, usually allowed an increase of the maximal cell density. However, depending on the enzyme used, the supplementation with hydrolysates corresponding to a high degree of hydrolysis and composed of at least 70% peptides with a molecular size under 1kDa, led to different maximal cell density values, indicating the importance of enzyme specificity and consequently the nature of the released peptides. This result showed that the positive influence of the rapeseed hydrolysates on cell growth was not only due to a nutritional support tied to the addition of small peptides but may be related to the presence of peptides exhibiting growth or survival factor effects. Furthermore, total substitution of proteins (transferrin, albumin and insulin) in the cell culture medium by some rapeseed hydrolysates appeared to be a promising alternative to improve the cell growth in protein-free media.

891 ||| 19002822 ||| (6, 0.40) ||| 6 ||| 7 ||| The effects of cell adhesion on the growth and protein productivity of animal cells. We investigated the effect of cell adhesion on cellgrowth and productivity of recombinant protein inChinese hamster ovary (CHO) cells. Cells cultured innormal tissue culture dishes attached to the dishsurfaces and grew as a monolayer, while cells culturedin non-treated dishes proliferated in suspension assingle cells without adhering to the dish surfaces. On an agarose-coated dish surface, cell aggregatesformed without attaching to the dish. Growth rates inboth suspension cultures were slightly lower thanthose in monolayer culture. Cell cycle analysisindicated that the duration of the G(1) phase insuspension cultures was longer than that in monolayerculture, suggesting that attachment to the substratummainly affected the transition from the G(1) to theS phase. Consistent with this, CDK inhibitor p27,that inhibits the G(1)S transition, was induced inthe cells cultured in suspension.To assess the productivity of recombinant proteins,CHO cells were transfected with a plasmid containingmurine interferon gamma (mIFN-gamma) under thecontrol of the cytomegalovirus promoter. Insuspension culture, mIFN-gamma productivity wasslightly lower than that in the monolayer culture. When protein kinase C was activated by phorbol ester,mIFN-gamma production was enhanced in both themonolayer and suspension cultures. However, theproductivity in the suspension culture was lower thanthat in the adherent culture even in the presence ofhigh concentrations of phorbol ester. These resultssuggested that cell adhesion to the substratum affectsvarious features of CHO cells.

892 ||| 24166780 ||| (6, 0.39) ||| 6 ||| 7 ||| Highly sialylated recombinant human erythropoietin production in large-scale perfusion bioreactor utilizing CHO-gmt4 (JW152) with restored GnT I function. Therapeutic glycoprotein drugs require a high degree of sialylation of their N-glycans for a better circulatory half-life that results in greater efficacy. It has been demonstrated that Chinese hamster ovary (CHO) glycosylation mutants lacking N-acetylglucosaminyltransferase I (GnT I), when restored by introduction of a functional GnT I, produced highly sialylated erythropoietin (EPO). We have now further engineered one of such mutants, JW152, by inactivating the dihydrofolate reductase (DHFR) gene to allow for the amplification of the EPO gene with methotrexate (MTX). Several MTX-amplified clones maintained the ability to produce highly sialylated EPO and one was selected for culture in a perfusion bioreactor that is used in an existing industrial EPO-production bioprocess. Extensive characterization of the EPO produced was performed using total sialic quantification, HPAEC-PAD and MALDI-TOF MS analyses. Our results demonstrated that the EPO produced by the mutant line exhibits superior sialylation compared to the commercially used EPO-producing CHO clone cultured under the same conditions. Therefore, this mutant has the industrial potential for producing highly sialylated recombinant EPO and potentially other recombinant glycoprotein therapeutics.

893 ||| 21436238 ||| (6, 0.39) ||| 6 ||| 7 ||| Enhanced sialylation of recombinant human erythropoietin in Chinese hamster ovary cells by combinatorial engineering of selected genes. Therapeutic glycoproteins with exposed galactose (Gal) residues are cleared rapidly from the bloodstream by asialoglycoprotein receptors in hepatocytes. Various approaches have been used to increase the content of sialic acid, which occupies terminal sites of N- or O-linked glycans and thereby increases the half-life of therapeutic glycoproteins. We enhanced sialylation of human erythropoietin (EPO) by genetic engineering of the sialylation pathway in Chinese hamster ovary (CHO) cells. The enzyme GNE (uridine diphosphate-N-acetyl glucosamine 2-epimerase)/MNK (N-acetyl mannosamine kinase), which plays a key role in the initial two steps of sialic acid biosynthesis, is regulated by cytidine monophosphate (CMP)-sialic acid through a feedback mechanism. Since sialuria patient cells fail in regulating sialic acid biosynthesis by feedback mechanism, various sialuria-like mutated rat GNEs were established and subjected to in vitro activity assay. GNE/MNK-R263L-R266Q mutant showed 93.6% relative activity compared with wild type and did not display feedback inhibition. Genes for sialuria-mutated rat GNE/MNK, Chinese hamster CMP-sialic acid transporter and human alpha2,3-sialyltransferase (alpha2,3-ST) were transfected simultaneously into recombinant human (rh) EPO-producing CHO cells. CMP-sialic acid concentration of engineered cells was significantly (>10-fold) increased by sialuria-mutated GNE/MNK (R263L-R266Q) expression. The sialic acid content of rhEPO produced from engineered cells was 43% higher than that of control cells. Ratio of tetra-sialylated glycan of rhEPO produced from engineered cells was increased ~32%, but ratios of asialo- and mono-sialylated glycans were decreased ~50%, compared with control. These findings indicate that sialuria-mutated rat GNE/MNK effectively increases the intracellular CMP-sialic acid level. The newly constructed host CHO cell lines produced more highly sialylated therapeutic glycoproteins through overexpression of sialuria-mutated GNE/MNK, CMP-SAT and alpha2,3-ST.

894 ||| 16233093 ||| (6, 0.39) ||| 6 ||| 7 ||| Use of nonionic surfactants for effective supply of phosphatidic acid in serum-free culture of Chinese hamster ovary cells. We have previously shown [Sakai et al., J. Biosci. Bioeng., 88, 306-309 (1999)] that exogenously supplied phosphatidic acid (PA) and lysophosphatidic acid (LPA) promoted the growth of Chinese hamster ovary (CHO) cells in serum-free culture. However, the direct addition of high concentrations of these phospholipids alone to the culture medium resulted in the formation of precipitates. We therefore examined the use of two nonionic surfactants, Tween 80 and Pluronic F-68, as a means of supplying PA more effectively to CHO cells in a serum-free culture. A clear dispersion of PA from egg yolk lecithin that could be successfully sterile-filtered was obtained by using Tween 80 or Pluronic F-68. When PA prepared with either of the surfactants was added to serum-free media, precipitation was noticeably reduced. Furthermore, the growth-promoting activity of PA was considerably enhanced by the presence of the surfactants. Since Tween 80 and Pluronic F-68 themselves possessed no growth-stimulating property, it was suggested that the enhanced growth-promoting activity results from the improved availability of PA to the cells. The use of Tween 80 with PA analogues having saturated acyl chains also accelerated cell growth, whereas these PAs showed little growth-promoting activity, due to their poor water-solubility, when added alone.

895 ||| 16232616 ||| (6, 0.39) ||| 6 ||| 7 ||| Effects of phospholipids on growth of Chinese hamster ovary cells in serum-free media. The effects of phosphatidic acid (PA) and lysophosphatidic acid (LPA) dispersed in serum-free media on the growth of Chinese hamster ovary (CHO) cells were investigated. After cells were incubated in serum-free media supplemented with PA or LPA for 3 d, the cellular growth was evaluated by the MTT (3-(4,5-dimethylthiazol-2-yl)-2,5-diphenyltetrazolium bromide) assay. Supplementing PA from egg yolk lecithin to basal synthetic media such as alpha-MEM (minimum essential medium, alpha modification) markedly promoted the growth of CHO cells. PA from egg yolk lecithin also enhanced the cell growth in a serum-free medium containing insulin and transferring. When PA analogues with different acyl chains of C14-18 were compared, PA with unsaturated acyl chains, dioleoyl-PA (C18: 1), was most effective for stimulating the growth of CHO cells. Similar results were obtained when LPA was examined. These results suggest that PA and LPA, especially those with unsaturated acyl chains, are promising growth-promoting supplements for use as constituents in a low-protein serum-free medium.

896 ||| 11027171 ||| (6, 0.38),(5, 0.35) ||| 6 ||| 7 ||| Increased t-PA yields using ultrafiltration of an inhibitory product from CHO fed-batch culture. Fed-batch operation for the production of t-PA using Chinese Hamster Ovary (CHO) cells was optimized using serial and parallel experimentation. The feed, an isotonic concentrate, was improved to obtain 2- to 2.5-fold increases in integrated viable cell days versus batch. With a low glucose inoculum train, the viability index was further increased up to 4.5-fold. Hydrolysates were substituted for the amino acid portion of the concentrate with no significant change in fed-batch results. The concentrate addition rate was based on a constant 4 pmol/cell.day glucose uptake rate that maintained a relatively constant glucose concentration (approximately 3 mM). Increased viable cell indices did not lead to concomitant increases in t-PA concentrations compared to batch. The fed-batch concentrate and feeding strategy were shown to be effective in hybridoma culture, where a 4-fold increase in viable cell index yielded a 4-fold increase in antibody concentration. The half-life of t-PA decreased from 43 to 15 days with decreasing cell viability (from 92% to 71%), but this was not sufficient to explain the apparent t-PA threshold. Instead, the CHO results were explained by a reduction in t-PA production at higher extracellular t-PA concentrations that limited the fed-batch maximum at 35 mg/L for the cell line investigated. Analysis of both the total and t-PA mRNA levels revealed no response to increasing extracellular t-PA concentrations upon exogenous additions. Instead, intracellular t-PA levels were increased, revealing a possible secretory pathway limitation. A new reactor configuration was developed using an acoustic filter to retain the cells in the reactor while an ultrafiltration module stripped t-PA from the clarified medium before the permeate was returned to the reactor. By adding this harvesting step, the t-PA fed-batch production was increased over 2-fold, up to a yield of 80 mg/L.

897 ||| 20574835 ||| (6, 0.38),(2, 0.30) ||| 6 ||| 7 ||| Hyperosmolarity enhances transient recombinant protein yield in Chinese hamster ovary cells. The effect of hyperosmolarity on transient recombinant protein production in Chinese hamster ovary (CHO) cells was investigated. Addition of 90 mM NaCl to the production medium ProCHO5 increased the volumetric yield of recombinant antibody up to 4-fold relative to transfection in ProCHO5 alone. Volumetric yields up to 50 mg l(-1) were achieved in a 6 day batch culture of 3 l. In addition, hyperosmolarity reduced cell growth and increased cell size. The addition of salt to cultures of transiently transfected CHO cells is a simple and cost-effective method to increase TGE yields in this host.

898 ||| 22008152 ||| (6, 0.36) ||| 6 ||| 7 ||| Synergizing metabolic flux analysis and nucleotide sugar metabolism to understand the control of glycosylation of recombinant protein in CHO cells. BACKGROUND: The glycosylation of recombinant proteins can be altered by a range of parameters including cellular metabolism, metabolic flux and the efficiency of the glycosylation process. We present an experimental set-up that allows determination of these key processes associated with the control of N-linked glycosylation of recombinant proteins. RESULTS: Chinese hamster ovary cells (CHO) were cultivated in shake flasks at 0 mM glutamine and displayed a reduced growth rate, glucose metabolism and a slower decrease in pH, when compared to other glutamine-supplemented cultures. The N-linked glycosylation of recombinant human chorionic gonadotrophin (HCG) was also altered under these conditions; the sialylation, fucosylation and antennarity decreased, while the proportion of neutral structures increased. A continuous culture set-up was subsequently used to understand the control of HCG glycosylation in the presence of varied glutamine concentrations; when glycolytic flux was reduced in the absence of glutamine, the glycosylation changes that were observed in shake flask culture were similarly detected. The intracellular content of UDP-GlcNAc was also reduced, which correlated with a decrease in sialylation and antennarity of the N-linked glycans attached to HCG. CONCLUSIONS: The use of metabolic flux analysis illustrated a case of steady state multiplicity, where use of the same operating conditions at each steady state resulted in altered flux through glycolysis and the TCA cycle. This study clearly demonstrated that the control of glycoprotein microheterogeneity may be examined by use of a continuous culture system, metabolic flux analysis and assay of intracellular nucleotides. This system advances our knowledge of the relationship between metabolic flux and the glycosylation of biotherapeutics in CHO cells and will be of benefit to the bioprocessing industry.

899 ||| 19003411 ||| (6, 0.36) ||| 6 ||| 7 ||| Recombinant insulin-like growth factor-I (IGF-I) production in Super-CHO results in the expression of IGF-I receptor and IGF binding protein 3. Previously, we described the genetic construction Super- CHO, a cell line capable of autocrine growth under fully defined protein-free conditions. Super-CHO cells constitutively express insulin growth factor-I (IGF-I) and transferrin in sufficient amounts to support long-term, stable growth without the addition of exogenous growth factors, thus making it an ideal host for the production of recombinant biopharmaceuticals. although IGF-I has been successfully expressed in Chinese Hamster Ovary (CHO) cells, the long term effects of recombinant IGF-I expression have not been explored. In particular, the expression of the endogenous IGF-I receptor in response to IGF-I production has not been reported. We report here the transcriptional induction of the type I IGF receptor gene in Super-CHO. In addition, we examined the conditioned medium for the presence of IGF-I binding proteins. Ligand blot analysis reveals the presence of IGF binding proteins present in the medium conditioned by Super-CHO cells as well as CHO cells incubated in the presence of IGF-I. Furthermore, immunoaffinity reveals that Super-CHO expresses IGF binding protein-3 in response to IGF-I production. These results suggest the autocrine growth of Super-CHO involves a complex interaction of cell type specific factors which regulate its utility of IGF-I.

900 ||| 17298900 ||| (6, 0.36) ||| 6 ||| 7 ||| Promotion of recombinant macrophage colony stimulating factor production by dimethyl sulfoxide addition in Chinese hamster ovary cells. A Chinese hamster ovary (CHO) cell line is the primary choice for the production of recombinant protein drugs with glycosylation modification. Recombinant protein productivity was enhanced by the addition of dimethyl sulfoxide (DMSO) in CHO cells. However, DMSO induced G0/G1 phase growth arrest and reduced cell growth rate. A two-stage process was developed to utilize the stimulatory effect of DMSO on recombinant protein production and mitigate the problem of growth inhibition in this study. In the first stage, cells are cultured without DMSO for a certain period in order to obtain a high cell density. Sequentially, DMSO is added to achieve a high specific productivity in the second stage. Using this approach, we found that by adding 1% DMSO after 24 h of growth, macrophage colony-stimulating factor (M-CSF) volumetric productivity is increased by 57% compared with the value obtained without the addition of DMSO.

901 ||| 10397838 ||| (6, 0.36) ||| 6 ||| 7 ||| Effect of glutamine limitation on the death of attached Chinese hamster ovary cells. The effect of glutamine depletion on the death of attached Chinese hamster ovary (CHO) cells was investigated. Experiments were performed using an anchorage dependent CHO cell line expressing gamma-IFN and a second cell line obtained by transfection of that cell line with the human bcl-2 (hbcl-2). Either cell line could grow in media devoid of glutamine with minimal cell death due to endogenous glutamine synthetase activity that allowed cells to synthesize glutamine from glutamic acid in the medium. However, compared to control cultures in glutamine-containing media, the cell growth rate in glutamine-free media was slower with an increased fraction of cells distributed in the G0/G1 phase. The slower rate of cell cycling apparently protected the cells from entering apoptosis when they were stimulated to proliferate in an environment devoid of other protective factors, such as serum or over-expressed hbcl-2. The depletion of both glutamine and glutamic acid did cause cell death, which could be mitigated by hbcl-2 over-expression.

902 ||| 10397845 ||| (6, 0.35),(1, 0.31) ||| 6 ||| 7 ||| Serum protects protein-free competent Chinese hamster ovary cells against apoptosis induced by nutrient deprivation in batch culture. The development of serum- and protein-free Chinese hamster ovary (CHO) cell cultures is a high priority for the production of biopharmaceuticals. Protein-free competent CHO cells lines have been previously constructed by two different methods-metabolic engineering with cell-cycle regulatory proteins and long-term selective adaptation. Apoptosis was present in both cell lines during protein-free, static-batch culture as a result of nutrient deprivation, and glucose deprivation alone was a potent inducer of apoptosis compared to the depletion of other nutrients such as amino acids. By adding back serum to the cultures during batch growth or nutrient deprivation, it was shown that unidentified survival factors in serum can greatly reduce apoptosis in protein-competent cell lines in all phases of the culture. Both observations contrast to previous reports for hybridoma cells, in which amino acids were the key determinants of apoptosis and serum had no additional antiapoptotic effect. Serum's protective effect against CHO cell death in batch culture was multifaceted and complex: (1) 10% FBS increased cell viability to >99% during exponential growth from roughly 75-90%, (2) 5-10% fetal bovine serum (FBS) reduced specific glucose consumption rates in both cell lines by 40%, thereby delaying the onset of apoptosis caused by glucose deprivation, and (3) 5% FBS reduced the specific cell death rate by 65% during a 3-d lactate-consumption phase characterized by substantial abortive proliferation, in which the cells both proliferated and died at a constant rate. The benefit of serum on cell production over the various phases of batch growth was combined into a single parameter by integrating the viable cell concentration vs. time profile (termed here as cumulative volumetric viable cell-time, VCTvol). Despite the ability of both cell lines to grow indefinitely without any exogenous growth factors, the addition of serum resulted in a 2. 3-fold increase in the VCTvol. Thus, it is clear that there is much room for improvement of protein-free CHO cell lines despite their adequate growth competence, and new strategies different from those successfully used for hybridomas may be necessary to combat CHO cell apoptosis.

903 ||| 15862358 ||| (6, 0.33),(5, 0.32) ||| 6 ||| 7 ||| Effects of amino acid additions on ammonium stressed CHO cells. Ammonium is a toxic and inhibitory byproduct of mammalian cell metabolism. At the end of a typical recombinant protein production campaign, the ammonium concentration can be as high as 10 mM, mainly due to glutamine metabolism. Intracellular pH (pH(i)) levels are sensitive to ammonium, which negatively impacts both cell growth and recombinant protein productivity. Ammonium also negatively affects the recombinant protein glycosylation profile, thus altering quality. Many strategies have been adopted to reduce ammonium accumulation, with limited results. This study investigated the addition of amino acids to the growth media for Chinese hamster ovary (CHO) cell cultures as a means of mitigating the negative effects of ammonium. Threonine, proline, and glycine additions improved CHO cell growth and recombinant protein levels. Further, the threonine, proline, and glycine additions positively impacted important metabolic parameters, including glucose consumption, lactate production, glutamine utilization, and final ammonium levels. Additionally, threonine, proline, and glycine increased the level of alpha(2,3)-linked sialic acid, galactose-beta(1,4)-N-acetylglucosamine, and alpha(2,6)-linked sialic acid residues on the recombinant tissue plasminogen activator (t-PA). Thus, threonine, proline, and glycine can be used to mitigate some of the toxic effects of ammonium on cell growth, recombinant protein productivity, and protein quality.

904 ||| 9634806 ||| (6, 0.31) ||| 6 ||| 7 ||| Removal of sialic acid from a glycoprotein in CHO cell culture supernatant by action of an extracellular CHO cell sialidase. We have directly tested the hypothesis that Chinese hamster ovary (CHO) cell-produced glycoproteins are subject to extracellular degradation by a sialidase endogenous to the CHO cell line. Factors important to understanding the potential for extracellular degradation are addressed including the glycoprotein specificity, subcellular source, mechanism of release, and stability of the sialidase activity. The extracellular CHO cell sialidase apparently originates from the cytosol of the cells, and is released to the cell culture supernatant as a result of damage to the cellular membrane. The extracellular sialidase is active toward a variety of CHO cell-produced glycoproteins, and can hydrolyze sialic acid from the recombinant glycoprotein gp120 in the culture supernatant. While measuring the actual degradation of a glycoprotein by extracellular CHO cell sialidase can be difficult, data presented here suggest that the level of degradation can be estimated indirectly by using a more convenient fluorescent substrate, 4-methylumbelliferyl-alpha-D-N-acetylneuraminic acid, to quantify sialidase activity. Degradation by sialidase is minimized through addition of the sialidase inhibitor 2,3-dehydro-2-deoxy-N-acetylneuraminic acid to the culture supernatant. The results in this study suggest additional potential approaches for minimizing degradation by sialidase, including isolation of a sialidase-deficient CHO cell line.

905 ||| 25014402 ||| (6, 0.31) ||| 6 ||| 7 ||| Effects of nutrient levels and average culture pH on the glycosylation pattern of camelid-humanized monoclonal antibody. The impact of operating conditions on the glycosylation pattern of humanized camelid monoclonal antibody, EG2-hFc produced by Chinese hamster ovary (CHO) cells has been evaluated by a combination of experiments and modeling. Cells were cultivated under different levels of glucose and glutamine concentrations with the goal of investigating the effect of nutrient depletion levels and ammonia build up on the cell growth and the glycoprofiles of the monoclonal antibody (Mab). The effect of average pH reduction on glycosylation level during the entire culture time or during a specific time span was also investigated. The relative abundance of glycan structures was quantified by hydrophilic interaction liquid chromatography (HILIC) and the galactosylation index (GI) and the sialylation index (SI) were determined. Lower initial concentrations of glutamine resulted in lower glucose consumption and lower cell yield but increased GI and SI levels when compared to cultures started with higher initial glutamine levels. Similarly, reducing the average pH of culture resulted in lower growth but higher SI and GI levels. These findings indicate that there is a tradeoff between cell growth, resulting Mab productivity and the achievement of desirable higher glycosylation levels. A dynamic model, based on a metabolic flux analysis (MFA), is proposed to describe the metabolism of nutrients, cell growth and Mab productivity. Finally, existing software (GLYCOVIS) that describes the glycosylation pathways was used to illustrate the impact of extracellular species on the glycoprofiles.

# ----------------------------- TOPIC7 -----------------------------

906 ||| 9870524 ||| (7, 0.99) ||| 7 ||| 4 ||| Development of a serum-free medium for the production of humanized antibody from Chinese hamster ovary cells using a statistical design. To develop serum-free (SF) media for the production of humanized antibody from recombinant Chinese hamster ovary (rCHO) cells, a statistical optimization approach based on a Plackett-Burman design was adopted. A basal medium was prepared by supplementing alpha-minimal essential medium (alpha-MEM) with Fe(NO3)3.9H2O, CuCl2, ZnSO4.7H2O, and Na2SeO3 which are generally contained in SF medium formulations. Insulin, transferrin, and ethanolamine were also supplemented to the basal medium to determine their optimal concentrations. From this statistical analysis, serine, phenylalanine, and tyrosine were identified as important determinants for cell growth. Also, putrescine, linoleic acid, and hydrocortisone were shown to be important for both cell growth and antibody production. The SF medium was formulated by supplementing the basal medium with components showing positive effects on cell growth and/or antibody production. Cell growth and antibody production in this SF medium were comparable to those in alpha-MEM supplemented with 5% dialyzed fetal bovine serum. Taken together, the results obtained here show that a Plackett-Burman design facilitates the development of SF media for rCHO cells aimed at producing a humanized antibody.

907 ||| 20014438 ||| (7, 0.99) ||| 7 ||| 4 ||| Hyperosmotic stress induces autophagy and apoptosis in recombinant Chinese hamster ovary cell culture. During recombinant Chinese hamster ovary (rCHO) cell culture, various events, such as feeding with concentrated nutrient solutions or the addition of base to maintain an optimal pH, increase the osmolality of the medium. To determine the effect of hyperosmotic stress on two types of programmed cell death (PCD), apoptosis and autophagy, of rCHO cells, two rCHO cell lines, producing antibody and erythropoietin, were subjected to hyperosmotic stress resulting from NaCl addition (310-610 mOsm/kg). For both rCHO cell lines, hyperosmolality up to 610 mOsm/kg increased cleaved forms of PARP, caspase-3, caspase-7, and fragmentation of chromosomal DNA, confirming the previous observation that apoptosis was induced by hyperosmotic stress. Concurrently, hyperosmolality increased the level of accumulation of LC3-II, a widely used autophagic marker, which was determined by Western blot analysis and confocal microscopy. When glucose and glutamine concentrations were measured during the cultures, glucose and glutamine concentrations in the culture medium at various osmolalities (310-610 mOsm/kg) showed no significant differences. This result suggests that induction of PCD by hyperosmotic stress occurred independently of nutrient depletion. Taken together, autophagy as well as apoptosis was observed in rCHO cells subjected to hyperosmolality.

908 ||| 19484373 ||| (7, 0.99) ||| 7 ||| 4 ||| Effects of heat treatment and concentration of fish serum on cell growth in adhesion culture of Chinese hamster ovary cells. The effects of heat treatment and concentration of fish serum (FS) on cell growth and human granulocyte-macrophage colony-stimulating factor (hGM-CSF) production in an adhesion culture of recombinant Chinese hamster ovary (CHO) cells, DR1000L4N, were investigated. The addition of heat treated FS instead of non-heat-treated FS improved cell growth in terms of cell density, which reached 60% that in 10% fetal calf serum (FCS)-containing medium (FCS medium). A decrease in FS concentration from 10 to 1.25% markedly increased cell density, which was 79% that in 10% FCS medium. The combination of heat treatment at 56 degrees C and the addition of FS at a low concentration (1.25%) showed an additive effect on cell growth and resulted in the same cell density as that in 10% FCS medium, whereas the hGM-CSF concentration in the culture using FS-containing medium (FS medium) was approximately 50% that in 10% FCS medium. The total lipid concentration in FS was more than three fold that in FCS. The effect of decreasing FS concentration on cell growth may be due to the low lipid concentration in FS medium, because addition of the lipids extracted from FS to 10% FCS and 1.25% FS media markedly decreased cell density. Consequently, the addition of heat-treated FS at low concentrations to medium may be useful for the growth of CHO cells without FCS.

909 ||| 17892962 ||| (7, 0.99) ||| 7 ||| 4 ||| Influence of co-down-regulation of caspase-3 and caspase-7 by siRNAs on sodium butyrate-induced apoptotic cell death of Chinese hamster ovary cells producing thrombopoietin. Previously, the expression of caspase-3 siRNA could not effectively inhibit sodium butyrate (NaBu)-induced apoptotic cell death of recombinant Chinese hamster ovary (rCHO) cells producing human thrombopoietin (hTPO). Caspase-3 siRNA expressing cells appeared to compensate for the lack of caspase-3 by increasing active caspase-7 levels. For the successful inhibition of NaBu-induced apoptosis of rCHO cells, both caspase-3 and caspase-7 were down-regulated using the siRNA expression vector system. Co-down-regulation of caspase-3 and caspase-7 increased cell viability and extended culture longevity in serum-free culture in the presence or absence of 1mM NaBu addition. In the cultures with 1mM NaBu addition, the maximum hTPO concentration in rCHO cells with down-regulation of both caspases was approximately 55% higher than that in rCHO cells without down-regulation of caspases and approximately 16% higher than rCHO cells with down-regulation of only caspase-3. However, in the culture with 3mM NaBu, this strategy could not dramatically enhance the culture longevity and hTPO production, compared to Bcl-2 overexpression. The different result in hTPO production between down-regulation of caspases and Bcl-2 overexpression may be because the down-regulation of caspase-3 and caspase-7, unlike Bcl-2 overexpression, could not maintain mitochondrial membrane potential in the presence of 3mM NaBu. Taken together, co-down-regulation of caspase-3 and caspase-7 is effective in regard to extension of culture longevity and enhancement of hTPO production in a serum-free culture in the presence or absence of 1mM NaBu addition.

910 ||| 12513118 ||| (7, 0.99) ||| 7 ||| 4 ||| Biphasic culture strategy based on hyperosmotic pressure for improved humanized antibody production in Chinese hamster ovary cell culture. Hyperosmotic pressure increased specific antibody productivity (q(Ab)) of recombinant Chinese hamster ovary (rCHO) cells (SH2-0.32) and it depressed cell growth. Thus, the use of hyperosmolar medium did not increase the maximum antibody concentration substantially. To overcome this drawback, the feasibility of biphasic culture strategy was investigated. In the biphasic culture, cells were first cultivated in the standard medium with physiological osmolality (294 mOsm/kg) for cell growth. When cells reached the late exponential growth phase, the spent standard medium was replaced with the fresh hyperosmolar medium (522 mOsm/kg) for antibody production. The q(Ab) in growth phase with the standard medium was 2.1 microg per 10(6) cells/d, whereas the q(Ab) in antibody production phase with the hyperosmolar medium was 11.1 microg per 10(6) cells/d. Northern blot analysis showed a positive relationship between the relative contents of intracellular immunoglobulin messenger ribonucleic acid and q(Ab). Because of the enhanced q(Ab) and the increased cell concentration in biphasic culture, the maximum antibody concentration obtained in biphasic culture with 522 mOsm/kg medium exchange was 161% higher than that obtained in batch culture with the standard medium. Taken together, the simple biphasic culture strategy based on hyperosmotic culture is effective in improving antibody production of rCHO cells.

911 ||| 10972928 ||| (7, 0.99) ||| 7 ||| 4 ||| Osmoprotective effect of glycine betaine on foreign protein production in hyperosmotic recombinant chinese hamster ovary cell cultures differs among cell lines. When three recombinant Chinese hamster ovary (rCHO) cell lines, CHO/dhfr-B22-4, CS13-1.00\*, and CS13-0.02\*, were cultivated in hyperosmolar media resulting from NaCl addition, their specific foreign protein productivity increased with medium osmolality. However, due to a simultaneous suppression of cell growth at elevated osmolality, no enhancement in the maximum foreign protein titer was made in batch cultures. To test the feasibility of using glycine betaine, known as a strong osmoprotective compound, for improved foreign protein production in hyperosmotic rCHO cell cultures, hyperosmotic batch cultures were carried out in the presence of 15 mM glycine betaine. Glycine betaine was found to have a strong osmoprotective effect on all three rCHO cell lines. Inclusion of 15 mM glycine betaine in hyperosmolar medium enabled rCHO cell lines to grow at 557 to 573 mOsm/kg, whereas they could not grow in the absence of glycine betaine. However, effect of glycine betaine inclusion in hyperosmolar medium on foreign protein production differed among rCHO cell lines. CHO/dhfr-B22-4 cells retained enhanced specific human thrombopoietin (hTPO) productivity in the presence of glycine betaine, and thereby the maximum hTPO titer obtained at 573 mOsm/kg was increased by 72% over that obtained in the control culture with physiological osmolality (292 mOsm/kg). On the other hand, enhanced specific antibody productivity of CS13-1.00\* and CS13-0.02\* at elevated osmolality was decreased significantly in the presence of glycine betaine. As a result, the maximum antibody titer at 557 mOsm/kg was similar to that obtained in the control culture with physiological osmolality. The mRNA contents per cell determined by northern blot hybridization correlated with q in all three rCHO cell lines, indicating that transcriptional regulation is responsible in part for q enhancement at hyperosmolality in the absence as well as the presence of glycine betaine. Taken together, efficacy of the simultaneous use of hyperosmotic pressure and glycine betaine as a means to improve foreign protein production was variable among different rCHO cell lines.

912 ||| 16233043 ||| (7, 0.94) ||| 7 ||| 4 ||| Effect of antioxidants on the apoptosis of CHO cells and production of tissue plasminogen activator in suspension culture. The effects of antioxidants on the apoptosis of Chinese hamster ovary (CHO) 1-15,500 cells and production of tissue plasminogen activator (tPA) in suspension culture were investigated. After cell growth to 2 x 10(5) cells/ml in Ham's F12 medium containing 10% newborn bovine serum (NBS) in a spinner bottle, CHO cells were maintained for 6 d in Ham's F12 medium containing 0 or 0.4% NBS and 10 mM antioxidants, namely, l-ascorbic acid 2-phosphate (VCP) or the reduced form of glutathione (GSH). The viable cell concentrations at day 6 in the serum-free culture with GSH and in the low-serum culture wiht VCP or GSH were 0.57, 1.04 and 1.69 x 10(5) cells/ml, respectively, while those in the serum-free and low-serum cultures without the antioxidants were only 2.33 and 1.17 x 10(3) cells/ml, respectively. The percentages of apoptotic cells in the serum-free and low-serum cultures with VCP (73.2, 44.6%) and GSH (76.9, 38.6%) measured using a flowcytometer after annexin V-FITC/propidium iodide staining were markedly lower than those in the cultures without antioxidants (96.3, 92.5%). The percentage of cells having a high mitochondrial membrane potential among the viable cells in the cultures with antioxidants determined using a flowcytometer after 5,5',6,6'-tetrachloro-1,1',3,3'-tetraethylbenzimidazolylcarbocyanine iodide staining was clearly higher than those in the cultures without the antioxidants. The production of tPA in the serum-free and low-serum media with VCP (0.282, 2.92 mg/l) or GSH (1.01, 1.61 mg/l) was markedly higher than that in the cultures without the antioxidants (0.275, 0.689 mg/l). Consequently, the suppression of apoptosis through the maintenance of the membrane potential of mitochondria by VCP or GSH resulted in a marked increase in tPA production by CHO cells in the serum-free and low-serum cultures.

913 ||| 22492702 ||| (7, 0.92) ||| 7 ||| 4 ||| Effect of sodium butyrate on autophagy and apoptosis in Chinese hamster ovary cells. Sodium butyrate (NaBu), which is widely used in recombinant Chinese hamster ovary cell (rCHO) cultures for high-level expression of therapeutic proteins, is known to induce apoptosis in a dose-dependent manner. Lately, the significance of autophagy has increased in the field of CHO cell culture due to the fact that autophagy is related to the programmed cell death mechanism. To determine the effect of NaBu on autophagy as well as apoptosis of rCHO cells, rCHO cells producing erythropoietin were subjected to NaBu treatment. NaBu treatment up to 5 mM increased cleaved forms of PARP, caspase-3, and Annexin V positive population, confirming the previous results that NaBu induces apoptosis. Concurrently, NaBu treatment increased the level of accumulation of the autophagic marker, LC3-II, independently of nutrient depletion, suggesting that NaBu induces autophagy. To elucidate the potential role of autophagy induced by NaBu, a representative autophagy inducer (rapamycin) or an inhibitor (bafilomycin A1) was added to cultures together with NaBu. It was found that autophagy had the potential role of a positive cell survival mechanism under NaBu treatment. Furthermore, gradual reduction in mitochondrial membrane potential/mass and recruitment of a mitophagy protein, Parkin, to the mitochondria were observed under NaBu treatment, suggesting that this positive function of autophagy might be mediated by the autophagic removal of damaged mitochondria. Taken together, autophagy was observed in rCHO cell culture under NaBu treatments and the results obtained here support the positive effects of autophagy induced by NaBu treatments.

914 ||| 12007864 ||| (7, 0.92) ||| 7 ||| 4 ||| Response of recombinant Chinese hamster ovary cells to hyperosmotic pressure: effect of Bcl-2 overexpression. In an attempt to use the hyperosmotic pressure for improved foreign protein production in recombinant Chinese hamster ovary (rCHO) cells, the response of rCHO cells producing a humanized antibody (SH2-0.32-(Delta)bcl-2 cells) to hyperosmotic pressure was determined in regard to cell growth and death, and antibody production. Further, the feasibility of Bcl-2 overexpression in improving rCHO cell viability under hyperosmotic pressure was also determined by comparing control cells (SH2-0.32-(Delta)bcl-2) with Bcl-2 overexpressing cells (14C6-bcl-2). After 3 days of cultivation in the standard medium (294 mOsm x kg(-1)), the spent medium was exchanged with the fresh media with various osmolalities (294-640 mOsm x kg(-1)). The results obtained show that hyperosmotic pressure inhibited cell growth in a dose-dependent manner, though 14C6-bcl-2 cells were less susceptible to hyperosmotic pressure than SH2-0.32-(Delta)bcl-2 cells. At 522 mOsm x kg(-1), SH2-0.32-(Delta)bcl-2 cells underwent a gradual cell death mainly through apoptosis due to the cytotoxic effect of hyperosmotic pressure. In contrast, Bcl-2 overexpression in 14C6-bcl-2 cells could delay the apoptosis induced by 522 mOsm x kg(-1) by inhibiting caspase-3 activation. Bcl-2 overexpression could also improve the cellular membrane integrity of 14C6-bcl-2 cells. When subjected to hyperosmotic pressure, the specific antibody productivity of SH2-0.32-(Delta)bcl-2 cells and 14C6-bcl-2 cells was increased in a similar extent. As a result, the final antibody concentration achieved in 14C6-bcl-2 cells at 522 mOsm x kg(-1) was 2.5-fold higher than that at 294 mOsm x kg(-1). At 580 mOsm x kg(-1), acute hyperosmotic pressure induced the rapid loss of viability in both SH2-0.32-(Delta)bcl-2 and 14C6-bcl-2 cells through necrosis rather than through apoptosis. Taken together, Bcl-2 overexpression and optimized hyperosmotic pressure could improve the antibody production of rCHO cells.

915 ||| 17334756 ||| (7, 0.89) ||| 7 ||| 4 ||| Fetal calf serum-free culture of Chinese hamster ovary cells employing fish serum. The effects of fish serum on cell growth and human granulocyte-macrophage colony-stimulating factor (hGM-CSF) production in an adhesion culture of Chinese hamster ovary (CHO) cells DR1000L4N were investigated and compared with those of fetal calf serum (FCS). Although fish serum did not stimulate the initial adhesion of CHO cells to culture dishes, it prompted cell growth after cell adhesion with FCS for 24 h. The cell density in the fish serum medium reached 75% that in the FCS medium. Fish serum promoted cell adhesion to and cell growth on collagen-coated dishes. The cell-specific production rate of hGM-CSF in the fish serum medium on collagen-coated dishes was almost the same as that in the FCS medium.

916 ||| 21875625 ||| (7, 0.86) ||| 7 ||| 4 ||| Bcl-x(L) overexpression delays the onset of autophagy and apoptosis in hyperosmotic recombinant Chinese hamster ovary cell cultures. Hyperosmolality in recombinant Chinese hamster ovary (rCHO) cell cultures induces autophagy and apoptosis. To investigate the effect of Bcl-x(L) overexpression on autophagy and apoptosis in hyperosmotic rCHO cell cultures, an erythropoietin (EPO)-producing rCHO cell line with regulated Bcl-x(L) overexpression was subjected to hyperosmolality resulting from NaCl addition in a batch culture and nutrient supplementation in a fed-batch culture. In the batch culture, Bcl-x(L) overexpression suppressed apoptosis, as evidenced by a decreased amount of cleaved caspase-7 and PARP. Concurrently, Bcl-x(L) overexpression also delayed autophagy, as indicated by reduced LC3 conversion, from LC3-I to LC3-II. As a result, the cell viability and EPO production were improved by Bcl-x(L) overexpression. In the fed-batch culture, the simultaneous application of Bcl-x(L) overexpression and nutrient feeding increased the culture longevity and maximum EPO concentration. Taken together, Bcl-x(L) overexpression delayed autophagy and apoptosis in hyperosmotic rCHO cell cultures, resulting in increased EPO production.

917 ||| 21495016 ||| (7, 0.85) ||| 7 ||| 4 ||| Autophagy and apoptosis of recombinant Chinese hamster ovary cells during fed-batch culture: effect of nutrient supplementation. Upon nutrient depletion during recombinant Chinese hamster ovary (rCHO) cell batch culture, cells are subjected to apoptosis, type I programmed cell death (PCD), and autophagy which can be type II PCD or a cell survival mechanism. To investigate the effect of nutrient supplementation on the two PCDs and protein production in rCHO cells, an antibody-producing rCHO cell line was cultivated in batch and fed-batch modes. The feed medium containing glucose, amino acids, and vitamins was determined through flask culture tests and used in bioreactor cultures. In the bioreactor cultures, the nutrient feedings extended the culture longevity and enhanced antibody production. In addition, cells in the fed-batch culture showed delayed onset of both apoptosis and autophagy, compared with those in the batch culture. The inhibition of apoptosis was demonstrated by a decreased amount of cleaved caspase-7 protein and less fragmentation of chromosomal DNA. Concurrently, reduced LC3 conversion, from LC3-I to LC3-II, was observed in cells that received the feeds. Cultivation with pharmacological autophagy inducer (rapamycin) or inhibitor (bafilomycin A1) indicated that autophagy is necessary for the cells to survive under nutrient depletion. Taken together, the delayed and relieved cell death by nutrient supplementation could improve antibody production.

918 ||| 10478796 ||| (7, 0.85) ||| 7 ||| 4 ||| Development of a serum-free medium for dihydrofolate reductase-deficient Chinese hamster ovary cells (DG44) using a statistical design: beneficial effect of weaning of cells. To develop serum-free (SF) medium for dihydrofolate reductase-deficient Chinese hamster ovary cells (DG44), a statistical optimization approach based on a Plackett-Burman design was adopted. DG44 cells which were normally maintained in 10 serum medium were gradually weaned to 0.5% serum medium to increase the probability of successful growth in SF medium. A basal medium was prepared by supplementing Dulbecco's modified Eagle's medium and Ham's nutrient mixture F12 with hypoxanthine (10 mg/l) and thymidine (10 mg/l). Twenty-eight different supplements were selected as variables on the basis of their growth-promoting abilities. From statistical analysis, leucine, tryptophan, lysine, proline, histidine, hydrocortisone, ethanolamine, and phosphatidylcholine were identified as important components showing positive effects on cell growth. A new SF medium (SF-DG44) was formulated by supplementing the basal medium with these components. When the weaned cells were inoculated at 1.0 x 10(5) cells/ml, a maximum viable cell concentration of 6.4 x 10(3)) cells/ ml was achieved in SF-DG44 medium. In contrast, when the unweaned cells were used, a concentration of only 4.1 x 10(5) cells/ml was reached under the same culture conditions, indicating that weaning of cells improves cell growth in SF medium. In summary, we found that development of a novel SF medium for DG44 cells was facilitated using a Plackett-Burman design technique and weaning of cells.

919 ||| 21723342 ||| (7, 0.84) ||| 7 ||| 4 ||| Growth factor withdrawal in combination with sodium butyrate addition extends culture longevity and enhances antibody production in CHO cells. The effect of growth factor (GF) and sodium butyrate (NaBu) on Chinese hamster ovary (CHO) cell growth, cell viability and antibody production was investigated using shaking flasks in GF-containing and GF-deficient medium containing 0, 1 and 3mM NaBu. The withdrawal of GF and the addition of NaBu suppressed cell growth, but they significantly increased specific antibody productivity, q(Ab). Interestingly, the withdrawal of GF in combination with the addition of NaBu markedly retarded cell death, leading to extended culture longevity. For instance, at 3mM NaBu, cell viability fell below 80% after day 4 in GF-containing medium, but it remained over 80% until day 18 in GF-deficient medium. Due to the enhanced q(Ab) and the extended culture longevity, approximately 2-fold increase in total antibody production was achieved in pseudo-perfusion culture with 1mM NaBu in GF-deficient medium, compared to the culture in GF-containing medium. The effect of GF and NaBu on the change in the expression and activity of cellular proteins, c-Myc, Bcl-2 and pyruvate dehydrogenase (PDH), was also investigated. Both the withdrawal of GF and the addition of NaBu decreased the expression of c-Myc. The expression of Bcl-2 was enhanced by the addition of NaBu in a dose-dependent manner while it was not affected by the withdrawal of GF. In addition, both the withdrawal of GF and the addition of NaBu reduced metabolic rates, q(Glc), q(Lac) and Y(Lac/Glc), and increased PDH activity while not affecting PDH expression, suggesting that they may reduce the glycolytic rates, but enhance the conversion rates of pyruvate to TCA intermediates. Taken together, the withdrawal of GF in combination with the addition of NaBu can be considered as a relevant strategy for alleviating NaBu-induced cell apoptosis and enhancing antibody production since it can be easily implemented as well as enhance q(Ab) and extend culture longevity.

920 ||| 22627923 ||| (7, 0.81) ||| 7 ||| 4 ||| Rapamycin treatment inhibits CHO cell death in a serum-free suspension culture by autophagy induction. Rapamycin, a specific mTOR inhibitor, has been used as a chemical activator in autophagy research both in vitro and in vivo. Recently, autophagy has received attention as an anti-cell death engineering target in addition to apoptosis in the Chinese hamster ovary (CHO) cell engineering field. Here, the effect of rapamycin and the subsequent autophagy induction is investigated on two CHO cell lines, DG44 host and an antibody-producing recombinant CHO (rCHO), in a serum-free suspension culture. In both cell lines, the rapamycin treatment delayed the viability drop and apoptosis induction. In particular, the improved cell viability of the antibody-producing rCHO cell line resulting from the rapamycin treatment led to a 21% increase in the maximum antibody concentration. From observations that a rapamycin derivative, everolimus, demonstrated similar positive effects in both cell lines, but not FK-506, which forms the same complex as rapamycin, but does not inhibit mTOR, it was demonstrated that the positive effects of rapamycin appear to be mTOR-dependent. In addition, the cultivation with rapamycin and/or an autophagy inhibitor, bafilomycin A1, indicated that the autophagy induction is related to the positive effects of rapamycin. The genetic perturbation of the autophagy pathway through the regulation of the expression level of Beclin-1, an important autophagy regulator, resulted in a delayed autophagy induction and apoptosis inhibition in response to the rapamycin treatment in the DG44 host cell line. Taken together, the results obtained in this study imply a positive role for autophagy and predict the usefulness of pro-autophagy engineering in CHO cell cultures.

921 ||| 10361720 ||| (7, 0.79) ||| 7 ||| 4 ||| Development of a serum-free medium for the production of erythropoietin by suspension culture of recombinant Chinese hamster ovary cells using a statistical design. In order to develop a serum-free (SF) medium for the production of erythropoietin (EPO) by suspension culture of recombinant Chinese hamster ovary (rCHO) cells, a statistical optimization approach based on a Plackett-Burman design was adopted. A basal medium was prepared by supplementing Iscove's modified Dulbecco's medium (IMDM) with Fe(NO3)3.9H2O, CuCl2 and ZnSO4.7H2O which are generally contained in SF medium formulations. Insulin, transferrin and ethanolamine were also supplemented to the basal medium to determine their optimal concentrations. From this statistical analysis, glutamate, serine, methionine, phosphatidylcholine, hydrocortisone and pluronic F68 were identified as positive determinants for cell growth. The SF medium was formulated by supplementing the basal medium with components showing positive effects on cell growth in suspension culture. An EPO titer in this optimized SF medium was 79% of that in IMDM supplemented with 5% dialyzed fetal bovine serum (dFBS). Furthermore, the in vitro and in vivo biological activities of EPO produced in the SF medium were comparable to those produced in the serum-supplemented medium. Taken together, the results obtained here show that a Plackett-Burman design facilitates the development of SF media for the production of EPO by suspension culture of rCHO cells.

922 ||| 18983878 ||| (7, 0.76) ||| 7 ||| 4 ||| Effect of Akt overexpression on programmed cell death in antibody-producing Chinese hamster ovary cells. Upon nutrient deprivation, Chinese hamster ovary (CHO) cells are subjected to two types of programmed cell death, apoptosis and autophagy. CHO cell engineering, as a means to improve foreign protein production, has focused mainly on anti-apoptosis. In this study, to determine the effect of Akt, which is known to regulate both apoptosis and autophagy, on cell survival and foreign protein production, constitutively active Akt was overexpressed in antibody-producing cells. Compared with the control cells, Akt overexpressing cells showed delayed onset of apoptosis and autophagy during batch culture. The inhibition of apoptosis was demonstrated by reduced amount of cleaved poly(ADP-ribose) polymerase and caspase 3 proteins and less fragmentation of chromosomal DNA. Moreover, under nutrient-limiting conditions, decreased level of autophagosome accumulation was observed in Akt overexpressing cells by the less accumulation of the 16kDa form of LC3-II and autophagic vacuoles. Taken together, the overexpression of constitutively active Akt in CHO cells could delay the onset of both types of programmed cell death during batch culture.

923 ||| 19266576 ||| (7, 0.75) ||| 7 ||| 4 ||| Effect of Bcl-xL overexpression on apoptosis and autophagy in recombinant Chinese hamster ovary cells under nutrient-deprived condition. Upon nutrient deprivation during culture, recombinant Chinese hamster ovary (rCHO) cells are subjected to two types of programmed cell death (PCD), apoptosis and autophagy. To investigate the effect of Bcl-x(L) overexpression on apoptosis and autophagy in rCHO cells, an erythropoietin (EPO)-producing rCHO cell line with regulated Bcl-x(L) overexpression (EPO-off-Bcl-x(L)) was established using the Tet-off system. The expression level of Bcl-x(L) in EPO-off-Bcl-x(L) cells was tightly regulated by doxycycline in a dose-dependent manner. Bcl-x(L) overexpression enhanced cell viability and extended culture longevity in batch culture. Upon nutrient depletion in the later stage of batch culture, Bcl-x(L) overexpression suppressed apoptosis by inhibiting the activation of caspase-3 and -7. Simultaneously, Bcl-x(L) overexpression also delayed autophagy, characterized by LC3-II accumulation. Immunoprecipitation analysis with a Flag-tagged Bcl-x(L) revealed that Bcl-x(L) interacts with Bax and Bak, essential mediators of caspase-dependent apoptosis, as well as with Beclin-1, an essential mediator of autophagy, and may inhibit their pro-cell death function. Taken together, it was found that Bcl-x(L) overexpression inhibits both apoptosis and autophagy in rCHO cell culture.

924 ||| 10068450 ||| (7, 0.74) ||| 7 ||| 4 ||| Enhancement of cytotoxicity of hydrogen peroxide by hyperthermia in chinese hamster ovary cells: role of antioxidant defenses. Regional hyperthermia has potential for human cancer treatment, particularly in combination with systemic chemotherapy or radiotherapy. The mechanisms involved in heat-induced cell killing are currently unknown. Hyperthermia may increase oxidative stress in cells, and thus, oxidative stress could have a role in the mechanism of cell death. We use hydrogen peroxide as a model oxidant to improve understanding of interactions between heat and oxidative stress. Heat increased cytotoxicity of hydrogen peroxide in Chinese hamster ovary cells. Altered levels of cellular antioxidants should create an imbalance between prooxidant and antioxidant systems, thus modifying cytotoxic responses to heat and to oxidants. We determine the involvement of the two cellular antioxidant defenses against peroxides, catalase and the glutathione redox cycle, in cellular sensitivity to heat, to hydrogen peroxide, and to heat combined with the oxidant. Defense systems were either inhibited or increased. For inhibition studies, intracellular glutathione was diminished to less than 15% of its initial level by treatment with L-buthionine sulfoximine (1 mM, 24 h). Inhibition of catalase was achieved with 3-amino-1,2,4-triazole (20 mM, 2 h), which caused a 80% decrease in endogenous enzyme activity. To increase antioxidants, cells were pretreated with the thiol-containing reducing agents, N-acetyl-L-cysteine, 2-oxo-4-thiazolidine carboxylate, and 2-mercaptoethane sulfonate. These compounds increased intracellular glutathione levels by 30%. Catalase activity was increased by addition of exogenous enzyme to cells. We show that levels of glutathione and catalase affect cellular cytotoxic responses to heat and hydrogen peroxide, either used separately or in combination. These findings are relevant to mechanisms of cell killing at elevated temperatures and suggest the involvement of oxidative stress.

925 ||| 22652083 ||| (7, 0.73) ||| 7 ||| 4 ||| Biphasic addition strategy of hypoxanthine and thymidine for improving monoclonal antibody production. In our previous study, we demonstrated that combinatorial addition of hypoxanthine (10 mg/L) and thymidine (2 mg/L) was able to stimulate initial cell growth and elevate volumetric concentration of antibody by 22% (Chen et al., Appl. Microbiol. Biotechnol., 93, 169-178, 2012). In this study, a systematic study was carried out to investigate the effects of hypoxanthine and thymidine (H&T) on cell growth and antibody production in a much wider range of concentration. In addition, we pursued to establish a highly productive fed-batch culture via rationally designing H&T addition regime. It was found that both cell growth and antibody production in batch cultures were H&T concentration-dependent. Specifically, a low concentration stimulated cell growth while exerting no influence on specific productivity (q(mAb)), and a high concentration inhibited cell growth, however, significantly enhancing q(mAb). Subsequent experiments with fed-batch shaking flasks demonstrated the feasibility of improving antibody production using a biphasic addition strategy for H&T: supplementing a low concentration of H&T during initial cell growth phase and a high concentration of H&T at the production phase. By applying the optimized feeding regime, a maximum viable cell density (VCD) of 6.45 x 10(6)cells/mL and volumetric antibody production of 632 mg/L were achieved in a 2 L-B.Braun bioreactor. Taken together, in this study, a biphasic H&T addition strategy for cell culture was developed, which hold great promise to improve antibody production.

926 ||| 21695809 ||| (7, 0.73) ||| 7 ||| 4 ||| Extracellular purine and pyrimidine catabolism in cell culture. The presence of purines and pyrimidines bases, nucleosides, and nucleotides in the culture medium has shown to differently affect the growth of a Chinese hamster ovary (CHO) cell line producing the secreted form of the human placental alkaline phosphatase enzyme (SEAP; Carvalhal et al., Biotech Prog. 2003;19:69-83). CHO, BHK, as well as Sf9 cell growth was clearly reduced in the presence of purines but was not affected by pyrimidines at the concentrations tested. The knowledge about the mechanisms by which nucleotides exert their effect when present outside the cells remains very incomplete. The catabolism of both extracellular purines and pyrimidines was followed during the culture of CHO cells. Purines/pyrimidines nucleotides added at a concentration of 1 mM to the culture medium decreased to negligible concentrations in the first 2 days. Purine and pyrimidine catabolism originated only purinic and pyrimidic end-products, respectively. The comparison between AMP catabolism in serum-free cultures (CHO cells expressing Factor VII and Sf9 cells) and in cultures containing serum (CHO cells expressing SEAP and BHK cells expressing Factor VII) showed that AMP extracellular catabolism is mediated by both cells and enzymes present in the serum. This work shows that the quantification of purines and pyrimidines in the culture medium is essential in animal cell culture optimization. When using AMP addition as a chemical cell growth strategy for recombinant protein production improvement, AMP extracellular concentration monitoring allows the optimization of the multiple AMP addition strategy for a prolonged cell culture duration with high specific productivity.

927 ||| 19003316 ||| (7, 0.73) ||| 7 ||| 4 ||| Effect of hypoosmotic pressure on cell growth and antibody production in recombinant Chinese hamster ovary cell culture. To determine the response of recombinant Chinese hamster ovary (rCHO) cells subjected to hypoosmotic pressure, rCHO cells (CS13\*-1.0) producing a chimeric antibody were cultivated in the hypoosmolar medium resulting from NaCl subtraction. At hypoosmotic pressure, CS13\*-1.0 cells displayed decreased specific growth rate (mu) and increased specific antibody productivity (q (Ab)).When the medium osmolality was decreased from 300 mOsm kg(-1)(physiological osmolality) to 150 mOsm kg(-1), mu was decreased by 68% and q (Ab) was increased by 128%. To understand the mechanism of enhanced q (Ab) resulting from hypoosmotic pressure, cellular responses of cells in the exponential phase of growth were observed at the transcription level. Total cytoplasmic RNA content per cell at 150 mOsm kg(-1) was increased by 140%, compared with that at 300 mOsm kg(-1). On a per mug RNA basis, immunoglobulin (Ig) mRNA levels at 150 mOsm kg(-1) were comparable to those at 300 mOsm kg(-1), indicating that hypoosmotic pressure did not lead to the preferential transcription of Ig mRNAs. Taken together, the data obtained here suggest that the increase in total RNA pool is primarily responsible for the enhanced q (Ab) of CS13\*-1.0 cells subjected to hypoosmotic pressure.

928 ||| 11027169 ||| (7, 0.73) ||| 7 ||| 4 ||| Osmoprotective effect of glycine betaine on thrombopoietin production in hyperosmotic Chinese hamster ovary cell culture: clonal variations. When 23 recombinant Chinese hamster ovary (rCHO) cell clones were cultivated in hyperosmolar medium resulting from NaCl addition (533 mOsm/kg), their specific thrombopoietin (TPO) productivity (q(TPO)) was increased. However, due to depressed cell growth at elevated osmolality, no enhancement in the maximum TPO titer was made in batch cultures of all 23 clones. To test the feasibility of using glycine betaine, known as a strong osmoprotective compound, for improved TPO production in hyperosmotic rCHO cell cultures, hyperosmotic batch cultures of 23 clones were performed in the presence of 15 mM glycine betaine. Glycine betaine was found to have a strong osmoprotective effect on all 23 clones. Inclusion of 15 mM glycine betaine in hyperosmolar medium enabled 22 clones to grow at 542 mOsm/kg, where most clones could not grow in the absence of glycine betaine, but at a cost of reduced q(TPO). However, the relative decrease in q(TPO) varied significantly among clones. Thus, efficacy of the simultaneous use of hyperosmotic pressure and glycine betaine as a means to improve foreign protein production was variable among clones. Six out of 23 clones displayed more than a 40% increase in the maximum TPO titer in the hyperosmolar medium containing glycine betaine, compared with that in the standard medium with a physiological osmolality. Taken together, the results obtained here emphasize the importance of selection of clones for the successful use of hyperosmotic pressure and glycine betaine as an economical means to improve TPO production.

929 ||| 11870612 ||| (7, 0.72) ||| 7 ||| 4 ||| Inhibition of sodium butyrate-induced apoptosis in recombinant Chinese hamster ovary cells by constitutively expressing antisense RNA of caspase-3. Sodium butyrate (NaBu) can enhance the expression of genes controlled by some of the mammalian promoters, but it can also inhibit cell growth and induce cellular apoptosis. Thus, the beneficial effect of using a higher concentration of NaBu on a foreign protein expression is compromised by its cytotoxic effect on cell growth. To overcome this cytotoxic effect of NaBu, the expression vector of antisense RNA of caspase-3 was constructed and transfected to recombinant Chinese hamster ovary (rCHO) cells producing a humanized antibody. Using this antisense RNA strategy, rCHO cells (B3) producing a low level of caspase-3 proenzyme were established. When batch cultures of both B3 cells and control cells transfected with antisense RNA-deficient plasmid were performed in the absence of NaBu, both cells showed similar profiles of cell growth and antibody production. Compared with control cell culture, under the condition of 5 mM NaBu addition at the exponential growth phase, expression of antisense RNA of caspase-3 significantly suppressed the NaBu-induced apoptosis of B3 cells and extended culture longevity by >2 days if the culture was terminated at cell viability of 50%. However, compared with control cell culture, the final antibody concentration of B3 cell culture was not increased in the presence of NaBu, which may be due to the loss of cellular metabolic capability resulted from the depolarization of mitochondrial membrane. Taken together, this study suggests that, although expression of antisense RNA of caspase-3 does not improve antibody productivity of rCHO cells, it can suppress NaBu-induced apoptotic cell death of rCHO cells and thereby may reduce problems associated with cellular disintegration.

930 ||| 11291028 ||| (7, 0.69) ||| 7 ||| 4 ||| Overexpression of bcl-2 inhibits sodium butyrate-induced apoptosis in Chinese hamster ovary cells resulting in enhanced humanized antibody production. Sodium butyrate (NaBu) can enhance the expression of genes from some of the mammalian promoters including cytomegalovirus (CMV) and simian virus 40 (SV40), but it can also inhibit cell growth and induce cellular apoptosis. Thus, the beneficial effect of using a higher concentration of NaBu on a foreign protein expression is compromised by its cytotoxic effect on cell growth. To overcome this cytotoxic effect of NaBu, a survival protein, human Bcl-2, was overexpressed in recombinant Chinese hamster ovary (CHO) cells (SH2-0.32), producing a humanized antibody directed against the S surface antigen of hepatitis B virus. When batch cultures of both control cells transfected with bcl-2-deficient plasmid (SH2-0.32-Deltabcl-2) and cells transfected with bcl-2 expression plasmid (14C6-bcl-2) were performed in the absence of NaBu, both cells showed similar profiles of cell viability and antibody production. Compared with the SH2-0.32-Deltabcl-2 culture, under the condition of NaBu addition at the exponential growth phase, overexpression of the bcl-2 gene considerably suppressed the NaBu-induced apoptosis of 14C6-bcl-2 by inhibiting caspase 3 activity and extending culture longevity by >2 days. As a result, the final antibody concentration of 14C6-bcl-2 culture was twofold higher than that of SH2-0.32-Deltabcl-2 culture in the presence of NaBu and threefold higher than that of SH2-0.32-Deltabcl-2 and 14C6-bcl-2 cultures in the absence of NaBu.

931 ||| 25132065 ||| (7, 0.67) ||| 7 ||| 4 ||| Effect of lithium chloride on the production and sialylation of Fc-fusion protein in Chinese hamster ovary cell culture. Lithium chloride (LiCl), which is a specific inhibitor of glycogen synthase kinase-3beta, is known to induce cell cycle arrest at the G2/M phase and to regulate apoptosis. To determine the potential of LiCl as a chemical additive to enhance specific productivity (q p) of recombinant Chinese hamster ovary (rCHO) cells through cell cycle arrest at G2/M phase, rCHO cells producing Fc-fusion protein were cultivated in serum-free media with LiCl concentrations ranging from 0 to 20 mM. The addition of LiCl induced cell cycle arrest at G2/M phase and thereby decreased the specific cell growth rate. However, LiCl increased q p in a dose-dependent manner. The beneficial effect of LiCl on q p outweighed its detrimental effect on mu, resulting in improved maximum Fc-fusion protein concentration (MFPC) at 10 mM LiCl. The q p and MFPC in the bioreactor culture with 10 mM LiCl were 5.0 and 2.1 times higher than those without LiCl, respectively. In addition, the presence of LiCl at 10 mM did not significantly affect either intracellular alpha2,3-ST or extracellular sialidase activity. LiCl also inhibited apoptosis of cells in the decline phase of growth by increasing Bcl-2 expression. Taken together, the results obtained in this study demonstrate the potential of LiCl as a q p-enhancing additive in CHO cell culture for improved recombinant protein production.

932 ||| 18663604 ||| (7, 0.67) ||| 7 ||| 4 ||| Calnexin overexpression sensitizes recombinant CHO cells to apoptosis induced by sodium butyrate treatment. Sodium butyrate (NaBu) can enhance the expression of foreign genes in recombinant Chinese hamster ovary (rCHO) cells, but it can also inhibit cell growth and induce cellular apoptosis. In this study, the potential role of calnexin (Cnx) expression in rCHO cells treated with 5 mM NaBu was investigated for rCHO cells producing tumor necrosis factor receptor FC. To regulate the Cnx expression level, a tetracycline-inducible system was used. Clones with different Cnx expression levels were selected and investigated. With regard to productivity per cell (q (p)), NaBu enhanced the q (p) by over twofold. Under NaBu treatment, Cnx overexpression further enhanced the q (p) by about 1.7-fold. However, under NaBu stress, the cells overexpressing Cnx showed a poorer viability profile with a consistent difference of over 25% in the viability when compared to the Cnx-repressed condition. This drop in the viability was attributed to increased apoptosis seen in these cells as evidenced by enhanced poly (ADP-ribose) polymerase cleavage and cytochrome C release. Ca(2+) localization staining and subsequent confocal imaging revealed elevated cytosolic Ca(2+) ([Ca(2+)](c)) in the Cnx-overexpressing cells when compared to the Cnx-repressed condition, thus endorsing the increased apoptosis observed in these cells. Taken together, Cnx overexpression not only improved the q (p) of cells treated with NaBu, but it also sensitized cells to apoptosis.

933 ||| 19739093 ||| (7, 0.64) ||| 7 ||| 4 ||| A DIGE approach for the assessment of differential expression of the CHO proteome under sodium butyrate addition: Effect of Bcl-x(L) overexpression. Bcl-x(L), a member of the Bcl-2 family, is known to inhibit apoptosis of recombinant Chinese hamster ovary (rCHO) cells induced by the addition of sodium butyrate (NaBu), which is used for the elevated expression of recombinant protein. In order to understand the intracellular effects of Bcl-x(L) overexpression on CHO cells treated with NaBu, changes to the proteome caused by controlled Bcl-x(L) expression in rCHO cells producing erythropoietin (EPO) in the presence of 3 mM NaBu were evaluated using two-dimensional differential in-gel electrophoresis (2D-DIGE) and MS analysis. The consequences of Bcl-x(L) overexpression were not limited to the apoptotic signaling pathway. Out of eight proteins regulated significantly by Bcl-x(L) overexpression in 3 mM NaBu addition culture, four proteins were related to cell survival (Iq motif-containing GTPase-activating protein 1), cell proliferation (dihydrolipoamide-S-acetyltransferase, guanine nucleotide binding protein alpha interacting 2), and repair of DNA damage (BRCA and CDKN1A interacting protein). Taken together, a DIGE approach reveals that overexpression of Bcl-x(L) not only inhibits apoptosis in the presence of NaBu but also affects cell proliferation and survival in various aspects.

934 ||| 23436561 ||| (7, 0.62) ||| 7 ||| 4 ||| Anti-cell death engineering of CHO cells: co-overexpression of Bcl-2 for apoptosis inhibition, Beclin-1 for autophagy induction. Genetic engineering approaches to inhibit cell death in Chinese hamster ovary (CHO) cell cultures have been limited primarily to anti-apoptosis engineering. Recently, autophagy has received attention as a new anti-cell death engineering target in addition to apoptosis. In order to achieve a more efficient protection of cells from the stressful culture conditions, the simultaneous targeting of anti-apoptosis and pro-autophagy in CHO cells (DG44) was attempted by co-overexpressing an anti-apoptotic protein, Bcl-2, and a key regulator of autophagy pathway, Beclin-1, respectively. Co-overexpression of Bcl-2 and Beclin-1 exhibited a longer culture period as well as higher viability during serum-free suspension culture, compared with the control (without co-overexpression of Bcl-2 and Beclin-1) and Bcl-2 overexpression only. In addition to the efficient inhibition of apoptosis by Bcl-2 overexpression, Beclin-1 overexpression successfully induced the increase in the autophagic marker protein, LC3-II, and autophagosome formation with the decrease in mTOR activity. Co-immunoprecipitation and qRT-PCR experiments revealed that the enforced expression of Beclin-1 increased Ulk1 expression and level of free-Beclin-1 that did not bind to the Bcl-2 despite the Bcl-2 overexpression. Under other stressful culture conditions such as treatment with sodium butyrate and hyperosmolality, co-overexpression of Bcl-2 and Beclin-1 also protected the cells from cell death more efficiently than Bcl-2 overexpression only, implying the potential of autophagy induction. Taken together, the data obtained here provide the evidence that pro-autophagy engineering together with anti-apoptosis engineering yields a synergistic effect and successfully enhances the anti-cell death engineering of CHO cells.

935 ||| 9652551 ||| (7, 0.60) ||| 7 ||| 4 ||| Cadmium suppresses apoptosis induced by chromium. Cadmium and chromium are both well-known human carcinogens, and common exposures to these metals are not infrequent. Recent studies have shown that hexavalent chromium induces apoptosis in Chinese hamster ovary (CHO) cells, suggesting an association of apoptosis with carcinogenesis. In contrast, induction of apoptosis by cadmium has been inconsistently observed. The present study was designed to determine if cadmium could induce apoptosis in CHO cells and if common exposure to cadmium and chromium would modify any apoptotic response. Apoptosis was evaluated by both agarose gel and in situ end-labeling methods. Apoptosis was observed at 48 h after treatment with 300 microM chromium (Na2CrO4) for 2 h. Cadmium alone at concentrations of 1, 5, or 10 microM (as CdCl2) did not induce apoptosis in these cells even at times up to 72 h after treatment. However, when CHO cells were concurrently exposed to cadmium and chromium, chromium-induced apoptosis was markedly suppressed in a cadmium concentration-related fashion. Cadmium did not consistently modify the cytotoxic effects of chromium, and significant increases in metallothionein were not induced by these metal treatments. These findings indicate that cadmium can block chromium-induced apoptosis. The suppression of apoptosis by cadmium may be a significant aspect of its carcinogenic mechanism.

936 ||| 8644118 ||| (7, 0.59) ||| 7 ||| 4 ||| N-actylcysteine protects Chinese hamster ovary (CHO) cells from lead-induced oxidative stress. In vitro administration of lead acetate (PbA) to cultures of Chinese hamster ovary (CHO) cells had a concentration-dependent inhibitory effect on colony formation. Colony formation was returned to control levels in lead-treated cultures that were supplemented with 1 mM N-actylcysteine (NAC), a well-documented synthetic antioxidant. In order to investigate the nature of NAC's protective effect, we measured L-gamma-glutamyl-L-cysteinylglycine (GSH), oxidized glutathione (GSSG), malondialdehyde (MDA) and catalase activity both in the presence and absence of NAC in lead-exposed CHO cells. Increases in both MDA levels (p < 0.05) and catalase activity (P < 0.05) were observed in cultures that received only PbA, but supplementation with NAC returned these measures to pretreatment levels. The ratio of GSH to GSSG increased in lead-exposed cells incubated in NAC-enhanced media, but declined in cultures treated with PbA only. Our results suggest that NAC can confer protection against lead-induced oxidative stress to CHO cells, possibly through the enhancement of the cell's own antioxidant defense mechanisms.

937 ||| 12884404 ||| (7, 0.58) ||| 7 ||| 4 ||| Apoptosis induced by different doses of caffeine on Chinese hamster ovary cells. Caffeine has been investigated for its potential mutagenic activity to bacteria, fungi and mammalian cells in culture, and at high concentrations it is also an inducer of apoptosis. Caffeine can exert acute cellular toxicity, including inhibition of cell growth and cell death, in Chinese hamster ovary cells. The aim of this study was to evaluate the cell survival and apoptotic or non-apoptotic effects of caffeine to different concentrations in Chinese hamster ovary cells (CHO-K1). These effects were evaluated by measuring cell viability, caspase 8 activity and fragmented DNA. This study suggests that the concentration of caffeine is of critical importance because high doses of caffeine induce apoptosis and low concentrations can act as an antioxidant. Previously, the cytotoxicity of caffeine was evaluated using a wide range of concentrations by the neutral red test. From this screening, adequate doses were selected to perform the caspase activity and fragmentation DNA studies. The potential antioxidant effect of caffeine was studied using tert-butyl-hydroperoxide as a free-radical generator. The repeatability was checked through three separate tests with the same concentration.

938 ||| 10193576 ||| (7, 0.58) ||| 7 ||| 4 ||| N-acetylcysteine increases the biosynthesis of recombinant EPO in apoptotic Chinese hamster ovary cells. Sodium butyrate (NaBu) is known to enhance the rate of biosynthesis of recombinant proteins in Chinese hamster ovary cells (CHO). Here we demonstrate that supplementation with NaBu during rapid growth brings about abrupt death of the cells. The death of the cells is due to apoptosis, as assessed by intranucleosomal DNA fragmentation. The promotion of apoptotic death of the cells could be partially blocked by treatment with the well-known antioxidant, N-acetylcysteine (NAC). Strikingly, the NAC treatment enhanced the production of recombinant EPO two-fold compared with that of the culture without NAC supplementation. These results showed that NaBu treatment supplemented with NAC not only inhibits apoptosis, but also exerts a synergistic effect on the biosynthesis of recombinant EPO.

939 ||| 16232949 ||| (7, 0.55) ||| 7 ||| 4 ||| Pentanoic acid, a novel protein synthesis stimulant for Chinese Hamster Ovary (CHO) cells. Nine carboxylic acids were tested to evaluate their effects on recombinant fusion protein production and cell growth of Chinese Hamster Ovary (CHO) cells. Pentanoic acid was demonstrated to have the highest enhancement effect on the protein biosynthesis of CHO cells among the acids tested. Pentanoic acid also had less growth suppression effects compared with butyrate. The optimal induction time and concentration of pentanoic acid for a 120-h batch culture were 72 h and 1 mM, respectively. Apoptosis (programmed cell death) was observed in the serum-free batch culture of CHO cells using a cell death detection ELISA kit. The addition of butyrate accelerated the rate of apoptosis of CHO cells whereas the addition of pentanoate did not. These results confirmed that pentanoic acid was a better stimulant for protein biosynthesis in animal cell culture than butyrate.

940 ||| 9666463 ||| (7, 0.52) ||| 7 ||| 4 ||| Alleviation of apoptosis by serum in Chinese hamster ovary cells ectopically expressing human Fas antigen. Fas-mediated apoptosis is an important regulatory mechanism for the development of T-cells and prevention of oncogenesis. Here, we establish Chinese hamster ovary (CHO) cell lines which stably express Fas antigen, and analyzed apoptosis induced by anti-Fas IgM. While Fas-transfected hamster cells did not undergo apoptosis when stimulated with anti-Fas antibody in the presence of medium containing 10% serum, in reduced serum concentrations, anti-Fas antibody caused these cells to round up and detach from the culture dish. Analysis of the DNA content by a flow cytometry demonstrated a significant increase of cells with sub-G1 amount of DNA upon Fas stimulation in the low serum concentrations. The increase in the number of apoptosis cells was inhibited by an apopain (CPP32, caspase 3) inhibitor or insulin-like growth factor-I. In contrast, apoptosis in a Fas-transfected mouse T-cell line occurred in the presence of 10% serum. these results suggest that factors including insulin-like growth factor-I in fetal bovine serum protect CHO cells from apopain-dependent apoptosis mediated by Fas-antigen stimulation.

941 ||| 22714280 ||| (7, 0.52) ||| 7 ||| 4 ||| Poly-gamma-glutamic acid enhances the growth and viability of Chinese hamster ovary cells in serum-free medium. The protective effects of polymer additives, including a group of viscosity-enhancing polymer poly-gamma-glutamic acid (gammaPGA; 10, 50, and 500 kDa) and surface-active polymer Pluronic F68, on Chinese hamster ovary cells against damage due to shear stress were investigated in shake-flask cultures. The level of protection was dependent upon the molecular weight of gammaPGA and its concentration. When 0.05 or 0.075 % of 500 kDa gammaPGA was added, the cell growth and viability were almost equal to those of Pluronic F68 supplementation and were much higher than those of the control without additives. For the first time, we show that gammaPGA is another environmentally-friendly medium additive that can be used in place of Pluronic F68.

942 ||| 23881315 ||| (7, 0.51) ||| 7 ||| 4 ||| Autophagy and its implication in Chinese hamster ovary cell culture. Chinese hamster ovary (CHO) cells, that are widely used for production of therapeutic proteins, are subjected to apoptosis and autophagy under the stresses induced by conditions such as nutrient deprivation, hyperosmolality and addition of sodium butyrate. To achieve a cost-effective level of production, it is important to extend the culture longevity. Until now, there have been numerous studies in which apoptosis of recombinant CHO (rCHO) cells was inhibited, resulting in enhanced production of therapeutic proteins. Recently, autophagy in rCHO cells has drawn attention because it can be genetically and chemically controlled to increase cell survival and productivity. Autophagy is a global catabolic process which involves multiple pathways and genes that regulate the lysosomal degradation of intracellular components. A simultaneous targeting of anti-apoptosis and pro-autophagy could lead to more efficient protection of cells from stressful culture conditions. In this regard, it is worthwhile to have a detailed understanding of the autophagic pathway, in order to select appropriate genes and chemical targets to manage autophagy in rCHO cells, and thus to enhance the production of therapeutic proteins.

943 ||| 11735431 ||| (7, 0.51) ||| 7 ||| 4 ||| Effects of cloned gene dosage on the response of recombinant CHO cells to hyperosmotic pressure in regard to cell growth and antibody production. The effect of cloned gene dosage on growth and product formation under hyperosmotic conditions has been studied using recombinant Chinese hamster ovary (rCHO) cell lines producing chimeric antibody. Batch cultures of four rCHO cell lines carrying different numbers of antibody gene copies were carried out using the hyperosmolar medium. Depending on cloned gene dosage, hyperosmotic pressure decreased specific growth rate (mu) and increased specific antibody productivity (q(Ab)) to a different degree. The cell line with lower cloned gene dosage displayed more significant enhancement in q(Ab) and less reduction in mu at hyperosmolalities. However, the cell line with higher cloned gene dosage still yielded higher maximum antibody concentration at hyperosmolality up to 469 mOsm/kg. Northern blot analysis showed a positive relationship between immunoglobulin mRNA level per cell and q(Ab), indicating that transcriptional regulation was involved in the response of rCHO cells to hyperosmotic pressure. Cell cycle analysis showed that hyperosmotic pressure induced G(1)-phase arrest, suggesting that the increase of cell population in G(1)-phase may contribute in part to enhanced q(Ab) at hyperosmolality. Taken together, although the cell line with lower cloned gene dosage displayed more significant enhancement in q(Ab) at hyperosmolality, the factor that determined the maximum antibody concentration in hyperosmotic rCHO cell cultures was almost exclusively the gene dosage.

944 ||| 19003018 ||| (7, 0.50) ||| 7 ||| 4 ||| Developement of serum-free media in CHO-DG44 cells using a central composite statistical design. A serum free medium was developed for the production of recombinant antibody against Botulinum A (BoNTA) using dihydrofolate reductase deficient Chinese Hamster Ovary Cells (CHO-DG44) in suspension culture. An initial control basal medium was prepared, which was similar in composition to HAM's F12: IMDM (1:1) supplemented with insulin, transeferrin, selenium and a lipid mixture. The vitamin concentration of the basal medium was twice that of HAM's F12: IMDM (1:1). CHO-DG44 cells expressing S25 antibody grew from 2 x 10(5) cells to maximum cell density of 1.04 x 10(6) cells/ml after 5 days in this control medium. A central composite design was used to identify optimal levels and interaction among five groups of medium components. These five groups were glutamine, Essential Amino Acids (EAA), Non Essential Amino Acids (NEAA), Insulin, Transferrin, Selenium (ITS), and lipids. Fifty experiments were carried out in four batches, with two controls in each batch. There was little effect of ITS and Lipid concentrations over the range studied, and glutamine concentration showed a strong interaction with EAA. The optimal concentrations of the variables studied were 2.5 mM Glutamine, 7.4 mM (2x) EAA, 1.4 mM (0.5x) NEAA, 1x ITS supplement, 0.7x Lipids supplement. The maximum viable cell density attained in the optimized medium was 1.4 x 10(6) cells/ml, a 35% improvement over the control culture, while the final antibody titer attained was 22 +/- 3.4 mug/mL, a 50% improvement.

945 ||| 19224599 ||| (7, 0.47),(6, 0.39) ||| 7 ||| 4 ||| Bcl-xL overexpression does not enhance specific erythropoietin productivity of recombinant CHO cells grown at 33 degrees C and 37 degrees C. Overexpression of bcl-xL in recombinant Chinese hamster ovary (rCHO) cells has been known to suppress apoptotic cell death and thereby extend culture longevity during batch culture. However, its effect on specific productivity (q) of rCHO cells is controversial. This study attempts to investigate the effect of bcl-xL overexpression on q of rCHO cells producing erythropoietin (EPO). To regulate the bcl-xL expression level, the Tet-off system was introduced in rCHO cells producing EPO (EPO-off-bcl-xL). The bcl-xL expression level was tightly controlled by doxycycline concentration. To evaluate the effect of bcl-xL overexpression on specific EPO productivity (q(EPO)) at different levels, EPO-off-bcl-xL cells were cultivated at the two different culture temperatures, 33 degrees C and 37 degrees C. The q(EPO) at 33 degrees C and 37 degrees C in the presence of 100 ng/mL doxycycline (without bcl-xL overexpression) were 4.89 +/- 0.21 and 3.18 +/- 0.06 microg/10(6)cells/day, respectively. In the absence of doxycycline, bcl-xL overexpression did not affect q(EPO) significantly, regardless of the culture temperature, though it extended the culture longevity. Taken together, bcl-xL overexpression showed no significant effect on the q(EPO) of rCHO cells grown at 33 degrees C and 37 degrees C.

946 ||| 16233032 ||| (7, 0.47),(5, 0.34) ||| 7 ||| 4 ||| Effects of high concentrations of energy sources and metabolites on suspension culture of Chinese hamster ovary cells producing tissue plasminogen activator. A high-concentration medium was developed for the high-density cultivation of Chinese hamster ovary (CHO) cells to produce tissue plasminogen activator (tPA). First, the effects of the concentrations of several medium components on the growth and metabolism of CHO cells were studied particularly in relation to the serum concentration, in order to develop a suitable concentrated medium. High concentrations of serum, glucose, lactate, and glutamine and a low ammonia concentration increased the specific growth rate and decreased the specific tPA production rate. Among these components, the effect of the serum concentration was the most significant. There was an inverse correlation between the specific rates of cell growth and tPA production. Using a medium containing glutamine at twice the concentration of the original medium (F-12) allowed the medium volume to be reduced to seven-ninths that of the original, without any reduction in tPA productivity. When the F-12 medium was supplemented five-fold with amino acids and vitamins, the total amount of tPA produced, specific rate of tPA production, and yield of tPA per unit volume of medium employed were respectively enhanced 1.7, 1.5, and 3.6 times. As a consequence, the frequency of medium exchange could be reduced. The results strongly indicate that the supplementation of nutrient amino acids and vitamins to a medium with a low concentration of serum are the most critical factors for cost-effective tPA production in high-density cell cultivation.

947 ||| 9951521 ||| (7, 0.47) ||| 7 ||| 4 ||| Apoptosis in batch cultures of Chinese hamster ovary cells. One of the main problems in the culture of Chinese Hamster Ovary (CHO) cells continues to be the inability to maintain the viability of the cultures over an extended period of time. The rapid decline in viability at the end of the culture is exacerbated by the absence of serum. In trying to reduce the extent of death in these cultures, we first tried to determine the mode of death. We found that more than 80% of the cells in a standard serum-free batch culture of CHO cells in suspension died via apoptosis--as evidenced by condensed chromatin and the appearance of a characteristic DNA ladder. Furthermore, when protein synthesis was inhibited using cycloheximide, the cells underwent rapid apoptosis indicating that death proteins were present in greater abundance than survival proteins in our CHO cells. Cell lysate from CHO cells showed evidence of cysteine protease (caspase) activity. Caspases of the Interleukin-1-beta-Converting Enzyme (ICE) family, e.g., CPP32, Mch-1, etc., have been implicated in the apoptotic process. Surprisingly, a caspase peptide inhibitor, N-benzyloxycarbonyl-Val-Ala-Asp-fluoro-methyl-ketone (z-VAD.fmk), was unable to substantially extend the life of a serum-free batch culture of CHO cells. In addition, z-VAD.fmk was only marginally able to extend viability in response to withdrawal of growth and survival factors, insulin and transferrin. In both these instances, z-VAD.fmk was able to prevent cleavage of caspase substrates, but not protect cells from death. However, we found that bcl-2 expression was able to significantly extend viabilities in CHO batch culture. Bcl-2 expression also substantially extended the viability of cultures in response to insulin and transferrin withdrawal. These results provide interesting insights into the pathways of death in a CHO cell.

948 ||| 18164778 ||| (7, 0.46),(1, 0.37) ||| 7 ||| 4 ||| Limitations to the comparative proteomic analysis of thrombopoietin producing Chinese hamster ovary cells treated with sodium butyrate. Sodium butyrate (NaBu) is known to enhance the specific productivity of Chinese hamster ovary cells expressing human thrombopoietin. In order to better understand the intracellular responses of these cells resulting from NaBu treatment, the proteomic profiles of cells treated with various concentrations of NaBu (0-3mM) were compared using two-dimensional electrophoresis (2-DE). Based on spot intensities, 80 high intensity protein spots were selected. Fifty-six of the 80 protein spots, which represent 28 different kinds of proteins, were identified by MALDI-TOF-MS and MS/MS. Compared to control without NaBu treatment, the expression levels of 2 proteins (glucose regulated protein 78 (GRP 78) and peroxiredoxin 4) were increased over two fold with NaBu treatment and the expression level of phosphopyruvate hydratase was decreased over two fold with NaBu treatment. Due to multiplicity (multiple spots for one protein), a change in one single spot intensity from a 2-DE gel image may not represent the total change in expression level for that protein. Western blot analyses of GRP78, HSC70 and ERp57 confirmed the results of the MS analyses. However, a degree of change in expression level differed between the two methods, suggesting the necessity of a validating method to determine the total amount of the protein.

949 ||| 10699869 ||| (7, 0.40) ||| 7 ||| 4 ||| Influence of bcl-2 on cell death during the cultivation of a Chinese hamster ovary cell line expressing a chimeric antibody. The influence of Bcl-2 expression on the robustness of a CHO cell line (22H11) developed for the industrial production of a chimeric antibody was evaluated. Western blot analysis following transfection with the expression vector unexpectedly revealed upregulation of endogenous Bcl-2 expression in the control (Neo) cell line in response to exposure to the selection drug G418. This indicated that geneticin may function by inducing apoptosis in cells not carrying the control plasmid or expressing very low levels of survival genes. Thus, exposure to the drug enriched the culture for a population of cells which expressed enhanced levels of endogenous Bcl-2. In batch cultures, ectopic bcl-2 expression resulted in a 75% increase in maximum viable cell density over control cultures. Moreover, the rate of decrease in viability in the Bcl-2 cultures was significantly lower than that in the control cultures. After 18 days, the Bcl-2 viability was around 90%, compared to 20% in the control cultures. Evaluation of the mechanism of cell death revealed very few cells with classical apoptotic morphology. Around 10% were clearly necrotic, but the majority of dead cells were seen as chromatin free but otherwise relatively intact structures. Because of the relatively low rate of cell death in both cell lines, few cells were observed in the transitional, easily identifiable early stages of apoptosis. However, DNA gel electrophoresis revealed a clear ladder-pattern, but only in the control cultures, thus confirming high levels of apoptotic death. Antibody concentrations during both sets of cultures were very similar, both during the growth and death phases, with a maximum titer of around 40 microgram/ml. Analysis of Bcl-2 expression by flow cytometry revealed that the cultures contained two populations of cells: a large population which expressed high levels of Bcl-2 and a relatively smaller low-expressing population. During the course of the batch, the smaller, low-expressing population declined in frequency, suggesting that these cells were more sensitive to cell death. In addition, the mean level of Bcl-2 expression in the overexpressing population also declined significantly, presumably reflecting the exhaustion of precursors for protein synthesis following nutrient depletion. Importantly, when cells were taken from day 40 of the significantly extended Bcl-2 batch cultures, they immediately proliferated, confirming that they had retained their replicative potential. Cultivation of the cells in basal medium lacking (individually) serum, all amino acids, glutamate/asparagine, and, finally, glucose, resulted in relatively lower viable cell numbers and viability in the control cell line compared to the Bcl-2 cell line. Exposure of cells to ammonia toxicity also revealed the relative robustness of the bcl-2 transfected cells. When growth was arrested by treatment with 4 mM thymidine, Bcl-2 overexpressing cells exhibit a viability of over 80% after 5 days in culture, compared to only 40% in the control cell line. However, under growth-arrested conditions, there was no major difference in antibody titer between the two cell lines.

950 ||| 9562255 ||| (7, 0.39) ||| 7 ||| 4 ||| Enhanced NOR-1 gene expression by exposure of Chinese hamster cells to high-density 50 Hz magnetic fields. Enhanced expression of neuron derived orphan receptor (NOR-1) gene was observed by exposure of Chinese hamster ovary K1 (CHO-K1) cells to an extremely low frequency magnetic field (ELFMF) of 50 Hz at 400 mT, but not at 5 mT. The enhanced expression, reaching the maximum at 6 h, was transient and reduced to the control level after exposure to 400 mT ELFMF for 24 h. The NOR-1 expression induced by treatment with forskolin and TPA was further enhanced by the simultaneous treatment with 400 mT ELFMF, in which the maximum response was at 3 h. The NOR-1 expression by these treatments was induced more earlier than that by 400 mT ELFMF alone. When cells were treated with an inhibitor of the protein kinase C (calphostin C or crocetin) and Ca2+ entry blockers (nifedipin and dantrolen) during the 400 mT ELFMF exposure, the enhanced NOR-1 expression was not observed. Exposure of CHO-K1 cells to the high-density 400 mT ELFMF may affect the signal transduction in the cells, resulting in the enhanced NOR-1 gene expression.

951 ||| 11745139 ||| (7, 0.39) ||| 7 ||| 4 ||| Expression of the transcription factor GADD153 is an indicator of apoptosis for recombinant chinese hamster ovary (CHO) cells. Loss of cell viability, through engagement of apoptotic cell death, represents a limitation to maintenance of high levels of productivity of recombinant animal cells in culture. The ability to monitor the status of recombinant cells, and to define indicators of their "well-being," would present a valuable approach to permit a rational intervention at appropriate times during culture. Growth arrest and DNA damage gene 153 (GADD153) is a member of the CCAAT/enhancer-binding protein (C/EBP) family of transcription factors and has been associated with apoptosis. We have examined the expression of GADD153 in conditions associated with apoptosis of recombinant CHO cells in batch culture. GADD153 expression is very low in CHO cells growing in the exponential phase of batch culture but is activated as cells enter the decline phase. Depletion of nutrients (glucose or glutamine) causes activation of GADD153 expression as does the imposition of endoplasmic reticulum stress. In all cases, there is a good relationship between the extent of apoptosis that occurs in response to each stress and the degree of GADD153 expression. In addition, nutrient refeeding or reversal of stress produces a concomitant decrease in expression of GADD153 and the susceptibility to apoptosis. Thus, GADD153 appears to offer a valid indicator of apoptosis and illustrates the potential for definition of monitors of cellular status related to the likelihood of apoptosis of cell populations.

952 ||| 19559063 ||| (7, 0.38),(6, 0.36) ||| 7 ||| 4 ||| Use of NaCl prevents aggregation of recombinant COMP-angiopoietin-1 in Chinese hamster ovary cells. To investigate the effect of hyperosmotic medium on production and aggregation of the variant of Angiopoietin-1 (Ang1), cartilage oligomeric matrix protein (COMP)-Ang1, in recombinant Chinese hamster ovary (CHO) cells, CHO cells were cultivated in shaking flasks. NaCl and/or sorbitol were used to raise medium osmolality in the range of 300-450mOsm/kg. The specific productivity of COMP-Ang1, q(COMP-Ang1), increased as medium osmolality increased. At NaCl-450mOsm/kg, the q(COMP-Ang1) was 7.7-fold higher than that at NaCl-300mOsm/kg, while, at sorbitol-450mOsm/kg, it was 2.9-fold higher than that at sorbitol-300mOsm/kg. This can be attributed to the increased relative mRNA level of COMP-Ang1 at NaCl-450mOsm/kg which was approximately 2.4-fold higher than that at sorbitol-450mOsm/kg. Western blot analysis showed that COMP-Ang1 aggregates started to occur in the late-exponential phase of cell growth. When sorbitol was used to raise the medium osmolality, a severe aggregation of COMP-Ang1 was observed. On the other hand, when NaCl was used, the aggregation of COMP-Ang1 was drastically reduced at NaCl-400mOsm/kg. At NaCl-450mOsm/kg, the aggregation of COMP-Ang1 was hardly observed. This suggests that environmental conditions are critical for the aggregation of COMP-Ang1. Taken together, the use of NaCl-induced hyperosmotic medium to cell culture process turns out to be an efficient strategy for enhancing COMP-Ang1 production and reducing COMP-Ang1 aggregation.

953 ||| 12892507 ||| (7, 0.38),(5, 0.36) ||| 7 ||| 4 ||| Effect of low culture temperature on specific productivity and transcription level of anti-4-1BB antibody in recombinant Chinese hamster ovary cells. Lowering the culture temperature has been suggested as a useful tool for improving the production of recombinant proteins in Chinese hamster ovary (CHO) cells. In an effort to improve anti-4-1BB antibody production in recombinant CHO (rCHO) cells, rCHO cells producing anti-4-1BB antibody (LGA31-56) were cultivated at three different temperatures, 30, 33, and 37 degrees C. Lowering the culture temperature led to suppressed cell growth, cell cycle arrest in G(0)/G(1) phase, and improved cell viability for a longer period. However, antibody production and q(Ab) were not increased at low culture temperature. The maximum antibody concentration and q(Ab) at 37 degrees C were 110.6 +/- 2.6 microg mL(-)(1) and 0.43 +/- 0.03 microg (10(6) cells h)(-)(1), respectively, whereas those at 30 degrees C were 28.3 +/- 3.8 microg mL(-)(1) and 0.44 +/- 0.07 (10(6) cells h)(-)(1), respectively. Northern blot analysis revealed that lowering the culture temperature did not increase the transcription level of heavy and light chains. These results were quite in contrast with the improved production of erythropoietin, which is expressed in the same CHO host and driven by the same CMV promoters, by lowering the temperature. Taken together, the results obtained imply that the beneficial effect of low culture temperature on recombinant protein production in rCHO cells is cell-line-specific.

954 ||| 24557571 ||| (7, 0.38) ||| 7 ||| 4 ||| Effect of sodium butyrate on the assembly, charge variants, and galactosylation of antibody produced in recombinant Chinese hamster ovary cells. Sodium butyrate (NaBu) is known to increase the specific productivity of recombinant Chinese hamster ovary (rCHO) cells. To understand the effects of NaBu on the product quality, rCHO cells producing monoclonal antibody (Mab) were cultivated at various concentrations of NaBu (0 to 4 mM). NaBu increased correctly assembled Mab. In the absence of NaBu, the proportions of intact Mab (2H2L) and heavy chain dimer (2H) were 81 and 15 %. At 1 mM NaBu, the proportion of 2H2L increased to 93 %, whereas the proportion of 2H decreased to 2 %. No further increase in the proportion of 2H2L was obtained at a higher NaBu concentration. NaBu also affected the charge heterogeneity of Mab, which may affect the efficacy of Mab. The basic charge variants of Mabs increased with an increase in the NaBu concentration. In addition, NaBu affected the galactosylation of Mab negatively. Overall, the data obtained here show that NaBu used in rCHO cell cultures for improved Mab production affects certain quality aspects of Mab, in this case, the charge heterogeneity and galactosylation.

955 ||| 16598795 ||| (7, 0.38) ||| 7 ||| 4 ||| Combining caspase and mitochondrial dysfunction inhibitors of apoptosis to limit cell death in mammalian cell cultures. Apoptosis is now recognized as a significant problem in mammalian cell culture. Therefore, in this study, a single gene and multigene approach to inhibit apoptosis has been examined. Stable Chinese hamster ovary (CHO) cell lines were generated to overexpress different single, dual, and triple combinations of three apoptosis inhibitor genes. Two upstream inhibitors involved in the mitochondrial pathway, Bcl-X(L) and Aven, were expressed in addition to a downstream inhibitor of caspases. The caspase inhibitor, a variant of XIAP containing only the caspase inhibitory BIR domains (XIAP-BIRs), has been shown previously to enhance viabilities in mammalian cultures. Stable clonal cell lines were generated and tested for three apoptotic insults: Sindbis virus infection, the chemical reagent etoposide, and spent medium. For all single gene experiments, the Bcl-X(L)-containing cell lines provided superior protection to either the Aven- or XIAP-BIRs-containing cell lines following initial exposure to the insults. However, the cell lines expressing two or more anti-apoptosis proteins were more effective at inhibiting cell death than those expressing just one anti-apoptosis gene. The cell lines overexpressing Bcl-X(L) in combination with XIAP-BIRs were especially effective in delaying cell death for all three apoptotic insults. Expression of all three anti-apoptosis genes in concert was only slightly more effective than using Bcl-X(L) and XIAP-BIRs for some insults. During exposure to spent medium, CHO-BIRS + Aven + BclX(L) was the best inhibitor of apoptosis (IAP) initially, whereas CHO-BIRs + BclX(L) was particularly effective at later times of the experiment. In conclusion, the utilization of a mitochondrial dysfunction inhibitor used in combination with a caspase inhibitor was more effective in thwarting the progression of apoptosis than either inhibitor expressed individually. Thus, the concurrent expression of multiple apoptosis inhibitors may be the most effective strategy to increase survival of mammalian cells in culture.

956 ||| 20625486 ||| (7, 0.37),(3, 0.36) ||| 7 ||| 4 ||| Enhancement of human prolactin synthesis by sodium butyrate addition to serum-free CHO cell culture. Sodium butyrate (NaBu) has been used as a productivity enhancer for the synthesis of recombinant proteins in Chinese hamster ovary (CHO) cells. Thus, the influence of NaBu on the production of recombinant human prolactin (hPRL) from CHO cells was investigated for the first time. CHO cell cultures were submitted to a treatment with different concentrations of NaBu (0.25 to 4 mM). Quantitative and qualitative analyses by reverse-phase high-performance liquid chromatography (RP-HPLC) and Western blot or SDS-PAGE, carried out directly on CHO-conditioned medium, showed that the highest hPRL expression was obtained with 1 mM NaBu. In vitro biological assays based on noble rat lymphoma (Nb2) and mouse pro-B lymphoma (Ba/F3-LLP) cells were carried out on purified hPRL. Its bioactivity in the presence of NaBu was not apparently different from that of the First International Reference Reagent of recombinant hPRL (WHO 97/714). Our results show that NaBu increased the synthesis of recombinant hPRL in CHO cells, apparently without compromising either its structure or function.

957 ||| 25079388 ||| (7, 0.36) ||| 7 ||| 4 ||| Effects of glutamine and asparagine on recombinant antibody production using CHO-GS cell lines. A unique and nontraditional approach using glutamine and asparagine supplements for CHO-glutamine synthetase (GS) cell lines was studied. In our experiments, we found that a decrease in pH and an increase in cell death occurred in production phase of a GS cell line, leading to reduced antibody expression and lower antibody yields. The experimental results and the statistical analysis (ANOVA) indicated that additions of glutamine and asparagine in the basal and feed media were effective to buffer the cell culture pH, reduce lactate generation, maintain a higher cell viability profile, and improve antibody productivity. In bench-top bioreactors, glutamine and asparagine supplementation helped to prevent cell death, improve antibody yield, and reduce base usage. Glutamine is normally excluded from culture media for GS cell lines to prevent the bypass of selection pressure. In this study, however, the addition of glutamine did not affect cell population homogeneity, protein quality, or decrease antibody yield of two GS cell lines.

958 ||| 17658284 ||| (7, 0.36) ||| 7 ||| 4 ||| Specific inhibition of caspase-8 and -9 in CHO cells enhances cell viability in batch and fed-batch cultures. In an attempt to investigate the molecular mechanism that leads to apoptotic death in Chinese hamster ovary (CHO) cells in batch and fed-batch cultures, we cloned caspase-2, -8 and -9 from a CHO cDNA library. Recombinant Chinese hamster caspase-2 and -9 expressed in Escherichia coli show highest activities towards commercial peptide substrates Ac-VDVAD-pNA and Ac-LEHD-pNA, the designated commercial substrates for human caspase-2 and -9, respectively. However, Chinese hamster caspase-8 shows a broad specificity profile and it cleaves the caspase-9 substrate more efficiently than it cleaves the caspase-8 substrate. The commercially available fluoromethyl ketone type of caspase inhibitors, such as Z-LEHD-fmk, Z-IETD-fmk, Z-VDVAD-fmk and Z-DEVD-fmk, were shown to completely lack specificity in inhibiting these caspases. The reversible aldehyde form of inhibitors for human caspase-8 and -9, Ac-LEHD-CHO and Ac-IETD-CHO, are equally efficient in inhibiting Chinese hamster caspase-8. Therefore, the wildly used method of utilizing the "caspase-specific" inhibitors to track the role of individual caspases in dying cells can be inaccurate and thus misleading. As an alternative, we stably expressed dominant negative (DN) mutants of Chinese hamster caspase-2, -8 and -9 to specifically inhibit these enzymes in CHO cells. Our results showed that inhibition of either endogenous caspase-8 or caspase-9 enhanced the viability of the CHO cells in both batch and fed-batch suspension cultures, but the inhibition of caspase-2 had minimal effects. These results suggest that caspase-8 and -9 are possibly involved in the apoptotic cell death in batch and fed-batch cultures of CHO cells, whereas caspase-2 is not. These findings can be valuable in the development of strategies for genetically engineering CHO cells to counter apoptotic death in batch and fed-batch cultures.

959 ||| 17650954 ||| (7, 0.35),(6, 0.35) ||| 7 ||| 4 ||| Insulin and IGF-1 mediated inhibition of apoptosis in CHO cells grown in suspension in a protein-free medium. When Chinese hamster ovary (CHO) cells were grown in suspension and deprived of serum, 40% of them became apoptotic after 72 hours, as determined by flow cytometry analysis of TUNEL-labelled cells. Cell viability, assessed by erythrocin B staining, decreased correspondingly. An increase in the total fraction of cells expressing interleukin converting enzyme (ICE; caspase 1), B-cell lymphoma 2 protein (Bcl-2,) and Bcl-2 associated x protein (Bax) was shown by antibody probing and subsequent flow cytometry. The p53 tumour suppressor gene product level remained low within the cell population. Insulin-like growth factor-1 (IGF-1) inhibited cell death in a concentration-dependent manner, and at 20 ng/ml, cell viability was maintained close to 100% and no apoptotic cells were detected. Also, insulin was shown to inhibit cell death - at 1.0 microg/ml, cell viability was 95%, whereas 10% of the cells stained for apoptosis. At the highest concentrations of IGF-1 and insulin, the expression of ICE, Bcl-2 and Bax was fully suppressed, whereas the p53 product level increased, despite still being detectable in a minority of cells. Under these conditions, IGF-1 may increase p53 expression to restrain abnormal cell proliferation. It is concluded that special attention should be paid to exposure and culture conditions that induce acquired susceptibility to a toxic insult, during the development and validation of cell-based assays.

960 ||| 24333461 ||| (7, 0.35),(6, 0.33) ||| 7 ||| 4 ||| Digital mRNA profiling of N-glycosylation gene expression in recombinant Chinese hamster ovary cells treated with sodium butyrate. To understand the effects of sodium butyrate (NaBu) on protein glycosylation, recombinant Chinese hamster ovary (rCHO) cells producing Fc-fusion glycoprotein were subjected to 3mM NaBu. The addition of NaBu to the cultures reduced the relative proportion of acidic isoforms and sialic acid content of the glycoprotein. Fifty-two N-glycosylation-related gene expressions were also assessed by the NanoString nCounter system, which can provide a direct digital readout using custom-designed color-coded probes. Among them, ten genes (ugp, slc35a2, ganc, man1a, man1c, mgat5a, st3gal5, glb1, neu1, and neu3) were up-regulated and three genes (b4galt2, st3gal3, and neu2) were down-regulated significantly. Altered expression patterns in st3gal3, neu1, and neu3, which have roles in the sialic acid biosynthesis pathway, correlated with reduced sialic acid content of the glycoprotein by NaBu. Taken together, the results obtained in this study provide a better understanding of the detrimental effect of NaBu on N-glycosylation in rCHO cells.

961 ||| 21805168 ||| (7, 0.35),(2, 0.31) ||| 7 ||| 4 ||| Insight into the roles of hypoxanthine and thymidine [corrected] on cultivating antibody-producing CHO cells: cell growth, antibody production and long-term stability. The potential of hypoxanthine and thymidine (H&T) to promote growth of CHO cells and production of monoclonal antibody (mAb) was explored in this study. It was demonstrated that H&T stimulated the initial cell growth and enhanced volumetric production of anti-human CD20 mAb by 22%, mainly through the elevated integrated viable cell concentration (IVCC). The moderate alteration in cell cycle distribution might partially account for the increased cell growth. Subsequent long-term stability studies indicated that H&T did not accelerate decay kinetics in mAb productivity. Specifically, cells under both nucleic acids-replete (H&T supplementation) and nucleic acids-hungry (methotrexate treatment) culture conditions showed similar stable mAb production during the first 2 months, followed by a gradual decline in the specific production rate (q(mAb)) with a 40% drop at the fourth month. In addition, the decreased transcript level of intracellular heavy chain (HC) of anti-human CD20 mAb correlated well with the decreased q(mAb). Furthermore, genomic mutation rate regarding the loss-of-function occurrence of the hypoxanthine-guanine phosphoribosyltransferase (HPRT) gene was determined, showing that H&T repressed the HPRT spontaneous mutation rate while methotrexate (MTX) provoked the mutation rate. Collectively, our data illustrated that H&T as potential medium additives promoted both initial cell growth and volumetric production of mAb, while not affecting the long-term stability of antibody-producing CHO cells.

962 ||| 14706639 ||| (7, 0.34),(4, 0.34),(5, 0.30) ||| 7 ||| 4 ||| Modification of glycolysis affects cell sensitivity to apoptosis induced by oxidative stress and mediated by mitochondria. The effect of alteration of the glycolytic pathway on cell damage induced by oxidative stress was investigated with dihydrofolate reductase-deficient Chinese hamster ovary (CHO) cells that either overexpress cytosolic glycerol-3-phosphate dehydrogenase (CHO/cGPDH cells) or are depleted of the A subunit of lactate dehydrogenase as a result of anti-sense RNA expression (CHO/anti-LDH cells). The extent of oxidative phosphorylation in CHO/anti-LDH and CHO/cGPDH cells was increased and decreased, respectively, relative to that in parental CHO cells, as revealed by measurement of the intracellular content of ATP, the rate of cellular O(2) consumption, the mitochondrial membrane potential (DeltaPsi(m)), and the generation of reactive oxygen species. The sensitivity of these cell lines to cell death induced by the exogenous oxidant tert-butyl hydroperoxide decreased according to the rank order CHO/anti-LDH>CHO>CHO/cGPDH. Exogenous pyruvate markedly increased the sensitivity of CHO/cGPDH cells to oxidant-induced death. The differences among the three cell lines in susceptibility to oxidant-induced death were reflected in the proportion of oxidant-treated cells with a subdiploid DNA content, with a collapsed DeltaPsi(m), and with cytochrome c in the cytosol, indicating that death was mediated by apoptosis. These results demonstrate that the influx of respiratory substrate into mitochondria is an important determinant of cell sensitivity to oxidant-induced apoptosis.

963 ||| 19002966 ||| (7, 0.33),(1, 0.30) ||| 7 ||| 4 ||| Regulated overexpression of the survival factor bcl-2 in CHO cells increases viable cell density in batch culture and decreases DNA release in extended fixed-bed cultivation. Using multicistronic expression technology we generated a stable Chinese hamster ovary (CHO) cell line (MG12) expressing a model secreted heterologous glycoprotein, the secreted form of the human placental alkaline phosphatase (SEAP), and bcl-2, best known as an apoptosis inhibitor, in a tetracycline-repressible dicistronic configuration. In batch cultivations in serum-containing medium, MG12 cells reached twice the final viable cell density when Bcl-2 was overexpressed (in the absence oftetracycline) compared to MG12 populations culturedunder tetracycline-containing conditions (bcl-2repressed). However, bcl-2-expressing MG12 cellsshowed no significant retardation of the decline phasecompared to batch cultures in which the dicistronicexpression unit was repressed.Genetic linkage of bcl-2 expression with the reporter protein SEAP in our multicistronic construct allowed online monitoring of Bcl-2 expression over an extended, multistage fixed-bed bioreactor cultivation. The cloned multicistronic expression unit proved to be stable over a 100 day bioreactor run. CHO MG12 cells in the fixed-bed reactor showed a drastic decrease in the release of DNA into the culture supernatant under conditions of reduced tetracycline (and hencederepressed SEAP and bcl-2 overexpression). This observation indicated enhanced robustness associated with bcl-2 overexpression, similar to recent findings for constitutive Bcl-2-overexpressing hybridoma cells under the same bioprocess conditions. These findings indicate, in these serum-containing CHO cell cultures, that overexpression of Bcl-2 results in desirable modifications in culture physiology.

964 ||| 8905168 ||| (7, 0.33) ||| 7 ||| 4 ||| Heat shock proteins protect cultured fibroblasts from the cytotoxic effects of MPP+. 1-Methyl-4-phenylpyridinium (MPP+), the cytotoxic metabolite of 1-methyl-4-phenyl-1,2,3,6-tetrahydropyridine (MPTP), has been shown to be toxic to a variety of cell types in culture. The addition of media containing 1 mM MPP+ to cultures of Chinese hamster ovary (CHO) fibroblasts led to the gradual depletion of cellular ATP stores and subsequent cell death. A 12-min heat shock of the cells at 45 degrees C, 3 h prior to the addition of MPP(+)-containing media, significantly attenuated cell death. Heat shock pretreatment led to an increased synthesis of all the major heat shock proteins (HSPs) in CHO cells. Further, the addition of the protein synthesis inhibitor, cycloheximide, prevented the protective effect of heat shock pretreatment, indicating that this protection was dependent upon new protein synthesis. In additional experiments, a rat fibroblast cell line which has been stably transfected with, and constitutively expresses a cloned human HSP-70 gene, was found to be more resistant to the cytotoxic effects of MPP+ than the parental fibroblast cell line. These results indicate that HSPs are protective toward the deleterious effects of MPP+ and that their synthesis represents an important parameter in the neurotoxicity of MPTP.

965 ||| 11257603 ||| (7, 0.33) ||| 7 ||| 4 ||| Comparison of Bcl-2 to a Bcl-2 deletion mutant for mammalian cells exposed to culture insults. Apoptosis has been found to occur in bioreactors as a result of environmental stresses. The overexpression of bcl-2 is a widely used strategy to limit the induction of apoptosis in mammalian cell cultures. In this study, the effectiveness of wild-type Bcl-2 was compared to a Bcl-2 mutant lacking the nonstructured loop domain in two commercially prominent cell lines, Chinese hamster ovary (CHO) and baby hamster kidney (BHK) cells. The generation of a DNA "ladder" and condensation of chromatin indicated that apoptosis occurred in these cell lines following Sindbis virus infection and serum deprivation. When cells were engineered to overexpress the bcl-2 mutant, cell death due to Sindbis virus was inhibited in a concentration-dependent manner. Furthermore, the Bcl-2 mutant provided increased protection as compared to wild-type Bcl-2 following two model insults, Sindbis virus infection and serum deprivation. Total production for a heterologous protein encoded on the Sindbis virus was increased in cell lines expressing the Bcl-2 variants compared to the parental cell line. In order to understand the reasons for the improved anti-apoptosis properties of the mutant, wild-type Bcl-2 and mutant Bcl-2 were examined by Western blot following each model insult. Wild-type Bcl-2 was observed to degrade into a 23 kDa fragment following both Sindbis virus infection and serum withdrawal in both cell lines, while the mutant Bcl-2 protein was not degraded during the same period. The processing of Bcl-2 was found to correlate with reduced cell viabilities following the two external insults to suggest that Bcl-2 degradation may limit its ability to inhibit apoptosis. These studies indicate that the cells regulate anti-apoptosis protein levels and these processing events can limit the effectiveness of cell death inhibition strategies in mammalian cell culture systems.

966 ||| 23108857 ||| (7, 0.32) ||| 7 ||| 4 ||| Eliminating tyrosine sequence variants in CHO cell lines producing recombinant monoclonal antibodies. Amino acid sequence variants are defined as unintended amino acid sequence changes that contribute to product variation with potential impact to product safety, immunogenicity, and efficacy. Therefore, it is important to understand the propensity for sequence variant (SV) formation during the production of recombinant proteins for therapeutic use. During the development of clinical therapeutic products, several monoclonal antibodies (mAbs) produced from Chinese Hamster Ovary (CHO) cells exhibited SVs at low levels (<=3%) in multiple locations throughout the mAbs. In these examples, the cell culture process depleted tyrosine, and the tyrosine residues in the recombinant mAbs were replaced with phenylalanine or histidine. In this work, it is demonstrated that tyrosine supplementation eliminated the tyrosine SVs, while early tyrosine starvation significantly increased the SV level in all mAbs tested. Additionally, it was determined that phenylalanine is the amino acid preferentially misincorporated in the absence of tyrosine over histidine, with no other amino acid misincorporated in the absence of tyrosine, phenylalanine, and histidine. The data support that the tyrosine SVs are due to mistranslation and not DNA mutation, most likely due to tRNA(Tyr) mischarging due to the structural similarities between tyrosine and phenylalanine.

967 ||| 9113384 ||| (7, 0.31) ||| 7 ||| 4 ||| Stress dose-dependent suppression of heat shock protein gene expression by inhibiting protein synthesis during heat shock treatment. When a clone of Chinese hamster ovary (CHO) cells transfected with a plasmid containing a luciferase reporter gene under the control of the human heat shock protein (hsp) 70 gene promoter was treated with cycloheximide during heat exposure at 42 and 43 degrees C for 15 to 100 minutes and then incubated at 37 degrees C after removal of cycloheximide, reporter gene expression was suppressed by the protein synthesis inhibitor only at small heat shock doses (i.e., heat shock of less than 40 minutes at 42 degrees C and 15 minutes at 43 degrees C). A similar stress dose-dependent suppression of reporter gene expression by cycloheximide was also demonstrated by treatment with sodium arsenite at 37 degrees C. However, dexamethasone-dependent reporter gene expression in a different CHO clone was not inhibited after the inducer treatment for different times in the presence of cycloheximide. In addition, synthesis of most cellular proteins (except for hsp) was not affected after heat shock treatment with cycloheximide. The results suggested that the cycloheximide inhibition of gene expression is specific to hsp gene expression induced by limited stress doses. Furthermore, a prior 42 degrees C heat shock treatment for 30 minutes induced a decreased responsiveness (tolerance) to a second 42 degrees C heat treatment for hsp gene expression, but tolerance did not develop in cells exposed to the first heat shock in the presence of cycloheximide. These results confirm previous findings that induction of hsp gene expression by stress is balanced by the severity of stress and rate of protein synthesis. They also support the proposed model of autoregulation of hsp gene expression by levels of free hsp70.

# ----------------------------- TOPIC8 -----------------------------

968 ||| 8845100 ||| (8, 0.99) ||| 8 ||| 1 ||| Control of long-term perfusion Chinese hamster ovary cell culture by glucose auxostat. The strategies for control of the feed rate in high-density perfusion cultures of animal cells are limited to several simple schemes. While in an industrial environment simplicity is seen as a major advantage, the need for more elaborate closed-loop control methods that can improve process stability in long-term continuous cultures is also well understood. What has prevented the application of the advanced control strategies known from theory is the lack of reliable real-time information that can be used to close the feedback loop. Among the variables that are appropriate for direct feedback control of the perfusion rate, high priority should be given to the glucose concentration. Unlike some other environmental variables, such as dissolved oxygen and pH, it provides unambiguous information which facilitates the selection of the right feed rate. The present paper describes the application of a closed loop control scheme, known as a "glucose-stat", to the long-term cultivation of Chinese hamster ovary cells in a high-density (35-40 million cells/mL) perfusion process. The monitoring and control system worked successfully for more than 2.5 months without any signs of performance degradation. In targeting industrial application, issues such as reliability, sterility, and accuracy, are given high priority. The implementation of the glucose monitoring system, which is the main part of the control complex, is addressed in details. The performance of the perfusion culture was evaluated at four different glucose set points, providing essential information about process optimization. It became evident that the perfusion culture was operated in the so-called "high-gain" zone (where the system is highly sensitive to the dilution rate), which justifies the application of a feedback control. The on-line glucose concentration was also used by an embedded expert system which drove the process through the batch and the perfusion phase, achieving total computer control of the feed rate. In summary, the proposed glucose monitoring and control technique proved to be a reliable biotechnology tool which can be applied with confidence at an industrial scale to either microbial or mammalian cell cultures.

969 ||| 25716416 ||| (8, 0.99) ||| 8 ||| 1 ||| A consensus approach for estimating the predictive accuracy of dynamic models in biology. Mathematical models that predict the complex dynamic behaviour of cellular networks are fundamental in systems biology, and provide an important basis for biomedical and biotechnological applications. However, obtaining reliable predictions from large-scale dynamic models is commonly a challenging task due to lack of identifiability. The present work addresses this challenge by presenting a methodology for obtaining high-confidence predictions from dynamic models using time-series data. First, to preserve the complex behaviour of the network while reducing the number of estimated parameters, model parameters are combined in sets of meta-parameters, which are obtained from correlations between biochemical reaction rates and between concentrations of the chemical species. Next, an ensemble of models with different parameterizations is constructed and calibrated. Finally, the ensemble is used for assessing the reliability of model predictions by defining a measure of convergence of model outputs (consensus) that is used as an indicator of confidence. We report results of computational tests carried out on a metabolic model of Chinese Hamster Ovary (CHO) cells, which are used for recombinant protein production. Using noisy simulated data, we find that the aggregated ensemble predictions are on average more accurate than the predictions of individual ensemble models. Furthermore, ensemble predictions with high consensus are statistically more accurate than ensemble predictions with large variance. The procedure provides quantitative estimates of the confidence in model predictions and enables the analysis of sufficiently complex networks as required for practical applications.

970 ||| 23711520 ||| (8, 0.99) ||| 8 ||| 1 ||| Evaluation of macroporous and microporous carriers for CHO-K1 cell growth and monoclonal antibody production. The emergence of microcarrier technology has brought a renewed interest in anchorage-dependent cell culture for high-yield processes. Well-known in vaccine production, microcarrier culture also has potential for application in other fields. In this work, two types of microcarriers were evaluated for small-scale monoclonal antibody (mAb) production by CHOK1 cells. Cultures (5 ml) of microporous Cytodex 3 and macroporous CultiSpher-S carriers were performed in vented conical tubes and subsequently scaled-up (20 ml) to shake-flasks, testing combinations of different culture conditions (cell concentration, microcarrier concentration, rocking methodology, rocking speed, and initial culture volume). Culture performance was evaluated by considering the mAb production and cell growth at the phases of initial adhesion and proliferation. The best culture performances were obtained with Cytodex 3, regarding cell proliferation (average 1.85 +- 0.11 x 10(6) cells/ml against 0.60 +- 0.08 x 10(6) cells/ ml for CultiSpher-S), mAb production (2.04 +- 0.41 mug/ml against 0.99 +- 0.35 mug/ml for CultiSpher-S), and culture longevity (30 days against 10-15 days for CultiSpher-S), probably due to the collagen-coated dextran matrix that potentiates adhesion and prevents detachment. The culture conditions of greater influence were rocking mechanism (Cytodex 3, pulse followed by continuous) and initial cell concentration (CultiSpher-S, 4 x 10(5) cells/ml). Microcarriers proved to be a viable and favorable alternative to standard adherent and suspended cultures for mAb production by CHO-K1 cells, with simple operation, easy scale-up, and significantly higher levels of mAb production. However, variations of microcarrier culture performance in different vessels reiterate the need for optimization at each step of the scale-up process.

971 ||| 17013615 ||| (8, 0.98) ||| 8 ||| 1 ||| Metabolic design of macroscopic bioreaction models: application to Chinese hamster ovary cells. The aim of this paper is to present a systematic methodology to design macroscopic bioreaction models for cell cultures based upon metabolic networks. The cell culture is seen as a succession of phases. During each phase, a metabolic network represents the set of reactions occurring in the cell. Then, through the use of the elementary flux modes, these metabolic networks are used to derive macroscopic bioreactions linking the extracellular substrates and products. On this basis, as many separate models are obtained as there are phases. Then, a complete model is obtained by smoothly switching from model to model. This is illustrated with batch cultures of Chinese hamster ovary cells.

972 ||| 22698821 ||| (8, 0.86) ||| 8 ||| 1 ||| Dynamic metabolic models of CHO cell cultures through minimal sets of elementary flux modes. The concept of Elementary Flux Modes (EFMs) has been of central importance in a number of studies involving the analysis of metabolism. In Provost and Bastin (2007) this concept is used to translate the metabolic networks of the different phases of CHO cell cultures into macroscopic bioreactions linking extracellular substrates to products. However, a critical issue concerns the calculation of these elementary flux vectors, as their number combinatorially increases with the size of the metabolic network. In this study, a detailed metabolic network of CHO cells is considered, where the above-mentioned combinatorial explosion makes the computation of the elementary flux modes impossible. To alleviate this problem, a methodology proposed in Jungers et al. (2011) is used to compute a decomposition of admissible flux vectors in a minimal number of elementary flux modes without explicitly enumerating all of them. As a result, a set of macroscopic bioreactions linking the extracellular measured species is obtained at a very low computational expense. The procedure is repeated for the several cell culture phases and a global model is built using a multi-model approach, which is able to successfully predict the evolution of experimental data.

973 ||| 23548040 ||| (8, 0.78) ||| 8 ||| 1 ||| Probabilistic strain optimization under constraint uncertainty. BACKGROUND: An important step in strain optimization is to identify reactions whose activities should be modified to achieve the desired cellular objective. Preferably, these reactions are identified systematically, as the number of possible combinations of reaction modifications could be very large. Over the last several years, a number of computational methods have been described for identifying combinations of reaction modifications. However, none of these methods explicitly address uncertainties in implementing the reaction activity modifications. In this work, we model the uncertainties as probability distributions in the flux carrying capacities of reactions. Based on this model, we develop an optimization method that identifies reactions for flux capacity modifications to predict outcomes with high statistical likelihood. RESULTS: We compare three optimization methods that select an intervention set comprising up- or down-regulation of reaction flux capacity: CCOpt (Chance constrained optimization), DetOpt (Deterministic optimization), and MCOpt (Monte Carlo-based optimization). We evaluate the methods using a Monte Carlo simulation-based method, MCEval (Monte Carlo Evaluations). We present two case studies analyzing a CHO cell and an adipocyte model. The flux capacity distributions required for our methods were estimated from maximal reaction velocities or elementary mode analysis. The intervention set selected by CCOpt consistently outperforms the intervention set selected by DetOpt in terms of tolerance to flux capacity variations. MCEval shows that the optimal flux predicted based on the CCOpt intervention set is more likely to be obtained, in a probabilistic sense, than the flux predicted by DetOpt. The intervention sets identified by CCOpt and MCOpt were similar; however, the exhaustive sampling required by MCOpt incurred significantly greater computational cost. CONCLUSIONS: Maximizing tolerance to variable engineering outcomes (in modifying enzyme activities) can identify intervention sets that statistically improve the desired cellular objective.

974 ||| 24519447 ||| (8, 0.71) ||| 8 ||| 1 ||| Continuous flow micro-bioreactors for the production of biopharmaceuticals: the effect of geometry, surface texture, and flow rate. We used continuous flow micro-devices as bioreactors for the production of a glycosylated pharmaceutical product (a monoclonal antibody). We cultured CHO cells on the surface of PMMA/PDMS micro-channels that had been textured by micromachining and coated with fibronectin. Three different micro-channel geometries (a wavy channel, a zigzag channel, and a series of donut-shape reservoirs) were tested in a continuous flow regime in the range of 3 to 6 muL min(-1). Both the geometry of the micro-device and the flow rate had a significant effect on cell adhesion, cell proliferation, and monoclonal antibody production. The most efficient configuration was a series of donut-shaped reservoirs, which yielded mAb concentrations of 7.2 mg L(-1) at residence times lower than one minute and steady-state productivities above 9 mg mL(-1) min(-1). These rates are at about 3 orders of magnitude higher than those observed in suspended-cell stirred tank fed-batch bioreactors.

975 ||| 23143896 ||| (8, 0.68) ||| 8 ||| 1 ||| Process model comparison and transferability across bioreactor scales and modes of operation for a mammalian cell bioprocess. A Monod kinetic model, logistic equation model, and statistical regression model were developed for a Chinese hamster ovary cell bioprocess operated under three different modes of operation (batch, bolus fed-batch, and continuous fed-batch) and grown on two different bioreactor scales (3 L bench-top and 15 L pilot-scale). The Monod kinetic model was developed for all modes of operation under study and predicted cell density, glucose glutamine, lactate, and ammonia concentrations well for the bioprocess. However, it was computationally demanding due to the large number of parameters necessary to produce a good model fit. The transferability of the Monod kinetic model structure and parameter set across bioreactor scales and modes of operation was investigated and a parameter sensitivity analysis performed. The experimentally determined parameters had the greatest influence on model performance. They changed with scale and mode of operation, but were easily calculated. The remaining parameters, which were fitted using a differential evolutionary algorithm, were not as crucial. Logistic equation and statistical regression models were investigated as alternatives to the Monod kinetic model. They were less computationally intensive to develop due to the absence of a large parameter set. However, modeling of the nutrient and metabolite concentrations proved to be troublesome due to the logistic equation model structure and the inability of both models to incorporate a feed. The complexity, computational load, and effort required for model development has to be balanced with the necessary level of model sophistication when choosing which model type to develop for a particular application.

976 ||| 25869484 ||| (8, 0.64) ||| 8 ||| 1 ||| Fluorescence dye-based detection of mAb aggregates in CHO culture supernatants. Product yields, efficacy, and safety of monoclonal antibodies (mAbs) are reduced by the formation of higher molecular weight aggregates during upstream processing. In-process characterization of mAb aggregate formation is a challenge since there is a lack of a fast detection method to identify mAb aggregates in cell culture. In this work, we present a rapid method to characterize mAb aggregate-containing Chinese hamster ovary (CHO) cell culture supernatants. The fluorescence dyes thioflavin T (ThT) and 4-4-bis-1-phenylamino-8-naphthalene sulfonate (Bis-ANS) enabled the detection of soluble as well as large mAb aggregates. Partial least square (PLS) regression models were used to evaluate the linearity of the dye-based mAb aggregate detection in buffer down to a mAb aggregate concentration of 2.4 mug mL(-1). Furthermore, mAb aggregates were detected in bioprocess medium using Bis-ANS and ThT. Dye binding to aggregates was stable for 60 min, making the method robust and reliable. Finally, the developed method using 10 mumol L(-1) Bis-ANS enabled discrimination between CHO cell culture supernatants containing different levels of mAb aggregates. The method can be adapted for high-throughput screening, e.g., to screen for cell culture conditions influencing mAb product quality, and hence can contribute to the improvement of production processes of biopharmaceuticals in mammalian cell culture.

977 ||| 10099282 ||| (8, 0.64) ||| 8 ||| 1 ||| Specific heat flow rate: an on-line monitor and potential control variable of specific metabolic rate in animal cell culture that combines microcalorimetry with dielectric spectroscopy. One of the requirements for enhanced productivity by the animal culture systems used in biotechnology is the direct assessment of the metabolic rate by on-line biosensors. Based on the fact that cell growth is associated with an enthalpy change, it is shown that the specific heat flow rate is stoichiometrically related to the net specific rates of substrates, products, and indeed to specific growth rate, and therefore a direct reflection of metabolic rate. Heat flow rate measured by conduction calorimetry has a technical advantage over estimates for many material flows which require assays at a minimum of two discrete times to give the rate. In order to make heat flow rate specific to the amount of the living cellular system, it would be advantageous to divide it by viable biomass. This requirement has been fulfilled by combining a continuous flow microcalorimeter ex situ with a dielectric spectroscope in situ, the latter measuring the viable cell mass volume fraction. The quality of the resulting biosensor for specific heat flow rate was illustrated using batch cultures of Chinese hamster ovary cells (CHO 320) producing recombinant human interferon-gamma (IFN-gamma) during growth in a stirred tank bioreactor under fully aerobic conditions. The measuring scatter of the probe was decreased significantly by applying the moving average technique to the two participant signals. It was demonstrated that the total metabolic rate of the cells, as indicated by the specific heat flow rate sensor, decreased with increasing time in batch culture, coincident with the decline in the two major substrates, glucose and glutamine, and the accumulation of the by-products, ammonia and lactate. Furthermore, the specific heat flow rate was an earlier indicator of substrate depletion than the flow rate alone. The calorimetric-respirometric ratio showed the intensive participation of anaerobic processes during growth and the related IFN-gamma production. Specific heat flow rate was monotonically related to specific cell growth rate and associated with specific IFN-gamma production. Specific heat flow rate is potentially a valid control variable for the growth of genetically engineered cell lines producing target proteins.

978 ||| 23436783 ||| (8, 0.63) ||| 8 ||| 1 ||| Very high density of Chinese hamster ovary cells in perfusion by alternating tangential flow or tangential flow filtration in WAVE Bioreactor -part II: Applications for antibody production and cryopreservation. A high cell density perfusion process of monoclonal antibody (MAb) producing Chinese hamster ovary (CHO) cells was developed in disposable WAVE Bioreactor using external hollow fiber (HF) filter as cell separation device. Tangential flow filtration (TFF) and alternating tangential flow (ATF) systems were compared and process applications of high cell density perfusion were studied here: MAb production and cryopreservation. Operations by perfusion using microfiltration (MF) or ultrafiltration (UF) with ATF or TFF and by fed-batch were compared. Cell densities higher than 10(8) cells/mL were obtained using UF TFF or UF ATF. The cells produced comparable amounts of MAb in perfusion by ATF or TFF, MF or UF. MAbs were partially retained by the MF using ATF or TFF but more severely using TFF. Consequently, MAbs were lost when cell broth was discarded from the bioreactor in the daily bleeds. The MAb cell-specific productivity was comparable at cell densities up to 1.3 x 10(8) cells/mL in perfusion and was comparable or lower in fed-batch. After 12 days, six times more MAbs were harvested using perfusion by ATF or TFF with MF or UF, compared to fed-batch and 28x more in a 1-month perfusion at 10(8) cells/mL density. Pumping at a recirculation rate up to 2.75 L/min did not damage the cells with the present TFF settings with HF short circuited. Cell cryopreservation at 0.5 x 10(8) and 10(8) cells/mL was performed using cells from a perfusion run at 10(8) cells/mL density. Cell resuscitation was very successful, showing that this system was a reliable process for cell bank manufacturing.

979 ||| 19003344 ||| (8, 0.63) ||| 8 ||| 1 ||| High cell density and productivity culture of Chinese hamster ovary cells in a fluidized bed bioreactor. A recombinant Chinese hamster ovary clone was cultivated in a 2L Cytopilot Mini fluidized bed bioreactor using Cytoline 1 microcarriers and a 10L B. Braun stirred tank bioreactor with Cytodex 1 microcarriers. Cytoline 1 is a macroporous polyethylene microcarrier and Cytodex 1 is a solid DEAE-dextran microcarrier. Cytoline 1 microcarriers in the fluidized bed bioreactor were gently mixed by an uplifting flow. Circulation and sparging in Cytopilot Mini were separated from the fluidized microcarrier bed. Cytopilot Mini bioreactor with Cytoline 1 microcarriers offered 2.3 times more surface area than the stirred tank bioreactor. The 2L fluidized bed bioreactor accommodated approximately half the cells in the 10L stirred tank bioreactor. Moreover, Cytopilot Mini had approximately three times more product output rate and 5.5 times higher specific productivity than the stirred tank bioreactor.

980 ||| 24036437 ||| (8, 0.61) ||| 8 ||| 1 ||| An optimized method for extraction and quantification of nucleotides and nucleotide sugars from mammalian cells. Glycosylation is a critical attribute of therapeutic proteins given its impact on the clinical safety and efficacy of these molecules. The biochemical process of glycosylation is inextricably dependent on metabolism and ensuing availability of nucleotides and nucleotide sugars (NSs) during cell culture. Herein, we present a comprehensive methodology to extract and quantify these metabolites from cultured cells. To establish the full protocol, two methods for the extraction of these compounds were evaluated for efficiency, and the requirement for quenching and washing the sample was assessed. A chromatographic method based on anion exchange has been optimized to separate and quantify eight nucleotides and nine NSs in less than 30 min. Degradation of nucleotides and NSs under extraction conditions was evaluated to aid in selection of the most efficient extraction protocol. We conclude that the optimized chromatographic method is quick, robust, and sensitive for quantifying nucleotides and NSs. Furthermore, our results show that samples taken from cell culture should be treated with 50% v/v acetonitrile and do not require quenching or washing for reliable extraction of nucleotides and NSs. This comprehensive protocol should prove useful in determining the impact of nucleotide and NS metabolism on protein glycosylation.

981 ||| 16080690 ||| (8, 0.60) ||| 8 ||| 1 ||| Logistic equations effectively model Mammalian cell batch and fed-batch kinetics by logically constraining the fit. A four-parameter logistic equation was used to fit batch and fed-batch time profiles of viable cell density in order to estimate net growth rates from the inoculation through the cell death phase. Reduced three-parameter forms were used for nutrient uptake and metabolite/product formation rate calculations. These logistic equations constrained the fits to expected general concentration trends, either increasing followed by decreasing (four-parameter) or monotonic (three-parameter). The applicability of this approach was first verified for Chinese hamster ovary (CHO) cells cultivated in 15-L batch bioreactors. Cell density, metabolite, and nutrient concentrations were monitored over time and used to estimate the logistic parameters by nonlinear least squares. The logistic models fit the experimental data well, supporting the validity of this approach. Further evidence to this effect was obtained by applying the technique to three previously published batch studies for baby hamster kidney (BHK) and hybridoma cells in bioreactors ranging from 100 mL to 300 L. In 27 of the 30 batch data sets examined, the logistic models provided a statistically superior description of the experimental data than polynomial fitting. Two fed-batch experiments with hybridoma and CHO cells in benchtop bioreactors were also examined, and the logistic fits provided good representations of the experimental data in all 25 data sets. From a computational standpoint, this approach was simpler than classical approaches involving Monod-type kinetics. Since the logistic equations were analytically differentiable, specific rates could be readily estimated. Overall, the advantages of the logistic modeling approach should make it an attractive option for effectively estimating specific rates from batch and fed-batch cultures.

982 ||| 25377169 ||| (8, 0.59) ||| 8 ||| 1 ||| Representative mammalian cell culture test materials for assessment of primary recovery technologies: a rapid method with industrial applicability. Mammalian cell culture material is often difficult to produce accurately and reproducibly for downstream studies. This article presents a methodology for the creation of a set of cell culture test materials where key variables including cell density, cell viability, product, and the host cell protein (HCP) load can be manipulated individually. The methodology was developed using a glutamine synthetase Chinese hamster ovary cell line cultured at 5-L and 70-L scales. Cell concentration post-cell growth was manipulated using tangential flow filtration to generate a range of target cell densities of up to 100 x 10(6) cells/mL. A method to prepare an apoptotic cell stock to achieve target viabilities of 40-90% is also described. In addition, a range of IgG1 and HCP concentrations was achieved. The results illustrate that the proposed methodology is able to mimic different cell culture profiles by decoupling the control of the key variables. The cell culture test materials were shown to be representative of typical cell culture feed material in terms of particle size distribution and HCP population. This provides a rapid method to create the required feeds for assessing the feasibility of primary recovery technologies designed to cope with higher cell density cultures.

983 ||| 21618458 ||| (8, 0.59) ||| 8 ||| 1 ||| Development of a mathematical model for evaluating the dynamics of normal and apoptotic Chinese hamster ovary cells. A metabolic flux based methodology was developed for modeling the metabolism of a Chinese hamster ovary cell line. The elimination of insignificant fluxes resulted in a simplified metabolic network which was the basis for modeling the significant metabolites. Employing kinetic rate expressions for growing and non-growing subpopulations, a logistic model was developed for cell growth and dynamic models were formulated to describe culture composition and monoclonal antibody (MAb) secretion. The model was validated for a range of nutrient concentrations. Good agreement was obtained between model predictions and experimental data. The ultimate goal of this study is to establish a comprehensive dynamic model which may be used for model-based optimization of the cell culture for MAb production in both batch and fed-batch systems.

984 ||| 23810769 ||| (8, 0.56) ||| 8 ||| 1 ||| Modeling the coupled extracellular and intracellular environments in mammalian cell culture. The regulation of metabolism in mammalian cell culture is closely linked to the process of apoptosis-programmed cell death. Apoptosis negatively impacts culture viability, product yield, and quality. An improved understanding of the interaction between apoptosis and metabolism will give rise to better control over the culture process, and thus improvements in product yield. This study presents a mathematical model that describes both the metabolic fluxes involving the extracellular metabolites and the progression of apoptosis in terms of intracellular caspases, and thus highlights the interactions between these two processes. The model is trained and validated against experimental observations of Chinese Hamster Ovary cell culture producing monoclonal antibody. Importantly, the model describes the continued production of monoclonal antibody in post exponential phase by incorporating different rates of antibody production for separate sub-populations within the culture. A parameter estimability test was applied on the combined model to assess the confidence in parameter estimates.

985 ||| 25097916 ||| (8, 0.55) ||| 8 ||| 1 ||| Fluorescence-based soft sensor for at situ monitoring of Chinese hamster ovary cell cultures. Multi-wavelength fluorescence spectroscopy was investigated as a potential tool for use in monitoring key process variables that include: viable and dead cells, recombinant protein, glucose, and ammonia concentrations for Chinese hamster ovary (CHO) cells during cultivation.For the purpose of calibrating the fluorescence-based empirical model, cells were grown in batch mode with different initial glucose and glutamine concentrations.Spectrofluorometer settings were optimized to ensure reproducibility and accuracy of the acquired spectra. With the purpose of gaining qualitative insight into the evolution of the spectra, the trajectories of individual fluorophore peaks were studied during the cultivation process. Spectral changes related to biomass and secreted proteins were investigated by comparing the spectra at various stages during the downstream processing. A partial least square regression (PLSR) was used to formulate empirical models that related the input data set, i.e., the fluorescence excitation-emission matrix, to the actual state of the system including viable cell and dead cells and recombinant protein, glucose, and ammonia concentrations. The models exhibited accurate prediction ability for the process variables of interest.

986 ||| 24292563 ||| (8, 0.53) ||| 8 ||| 1 ||| Analysis of Chinese hamster ovary cell metabolism through a combined computational and experimental approach. Optimization of cell culture processes can benefit from the systematic analysis of experimental data and their organization in mathematical models, which can be used to decipher the effect of individual process variables on multiple outputs of interest. Towards this goal, a kinetic model of cytosolic glucose metabolism coupled with a population-level model of Chinese hamster ovary cells was used to analyse metabolic behavior under batch and fed-batch cell culture conditions. The model was parameterized using experimental data for cell growth dynamics, extracellular and intracellular metabolite profiles. The results highlight significant differences between the two culture conditions in terms of metabolic efficiency and motivate the exploration of lactate as a secondary carbon source. Finally, the application of global sensitivity analysis to the model parameters highlights the need for additional experimental information on cell cycle distribution to complement metabolomic analyses with a view to parameterize kinetic models.

987 ||| 23420794 ||| (8, 0.52) ||| 8 ||| 1 ||| Stimuli-responsive magnetic nanoparticles for monoclonal antibody purification. Monoclonal antibodies (mAbs) are important therapeutic proteins. One of the challenges facing large-scale production of monoclonal antibodies is the capacity bottleneck in downstream processing, which can be circumvented by using magnetic stimuli-responsive polymer nanoparticles. In this work, stimuli-responsive magnetic particles composed of a magnetic poly(methyl methacrylate) core with a poly(N-isopropylacrylamide-co-acrylic acid) (P(NIPAM-co-AA)) shell cross-linked with N, N'-methylenebisacrylamide were prepared by miniemulsion polymerization. The particles were shown to have an average hydrodynamic diameter of 317 nm at 18 C, which decreased to 277 nm at 41 C due to the collapse of the thermo-responsive shell. The particles were superparamagnetic in behavior and exhibited a saturation magnetization of 12.6 emu/g. Subsequently, we evaluated the potential of these negatively charged stimuli-responsive magnetic particles in the purification of a monoclonal antibody from a diafiltered CHO cell culture supernatant by cation exchange. The adsorption of antibodies onto P(NIPAM-co-AA)-coated nanoparticles was highly selective and allowed for the recovery of approximately 94% of the mAb. Different elution strategies were employed providing highly pure mAb fractions with host cell protein (HCP) removal greater than 98%. By exploring the stimuli-responsive properties of the particles, shorter magnetic separation times were possible without significant differences in product yield and purity.

988 ||| 25805268 ||| (8, 0.51) ||| 8 ||| 1 ||| Revisiting Verhulst and Monod models: analysis of batch and fed-batch cultures. The paper re-evaluates Verhulst and Monod models. It has been claimed that standard logistic equation cannot describe the decline phase of mammalian cells in batch and fed-batch cultures and in some cases it fails to fit somatic growth data. In the present work Verhulst, population-based mechanistic growth model was revisited to describe successfully viable cell density (VCD) in exponential and decline phases of batch and fed-batch cultures of three different CHO cell lines. Verhulst model constants, K, carrying capacity (VCD/ml or mug/ml) and r, intrinsic growth factor (h(-1)) have physical meaning and they are of biological significance. These two parameters together define the course of growth and productivity and therefore, they are valuable in optimisation of culture media, developing feeding strategies and selection of cell lines for productivity. The Verhulst growth model approach was extended to develop productivity models for batch and fed-batch cultures. All Verhulst models were validated against blind data (R(2) > 0.95). Critical examination of theoretical approaches concluded that Monod parameters have no physical meaning. Monod-hybrid (pseudo-mechanistic) batch models were validated against specific growth rates of respective bolus and continuous fed-batch cultures (R(2) 0.90). The reduced form of Monod-hybrid model CL/(KL + CL) describes specific growth rate during metabolic shift (R(2) 0.95). Verhulst substrate-based growth models compared favourably with Monod-hybrid models. Thus, experimental evidence implies that the constants in the Monod-hybrid model may not have physical meaning but they behave similarly to the biological constants in Michaelis-Menten enzyme kinetics, the basis of the Monod growth model.

989 ||| 24498415 ||| (8, 0.49) ||| 8 ||| 1 ||| Controllability analysis of protein glycosylation in CHO cells. To function as intended in vivo, a majority of biopharmaceuticals require specific glycan distributions. However, achieving a precise glycan distribution during manufacturing can be challenging because glycosylation is a non-template driven cellular process, with the potential for significant uncontrolled variability in glycan distributions. As important as the glycan distribution is to the end-use performance of biopharmaceuticals, to date, no strategy exists for controlling glycosylation on-line. However, before expending the significant amount of effort and expense required to develop and implement on-line control strategies to address the problem of glycosylation heterogeneity, it is imperative to assess first the extent to which the very complex process of glycosylation is controllable, thereby establishing what is theoretically achievable prior to any experimental attempts. In this work, we present a novel methodology for assessing the output controllability of glycosylation, a prototypical example of an extremely high-dimensional and very non-linear system. We first discuss a method for obtaining the process gain matrix for glycosylation that involves performing model simulations and data analysis systematically and judiciously according to a statistical design of experiments (DOE) scheme and then employing Analysis of Variance (ANOVA) to determine the elements of process gain matrix from the resulting simulation data. We then discuss how to use the resulting high-dimensional gain matrix to assess controllability. The utility of this method is demonstrated with a practical example where we assess the controllability of various classes of glycans and of specific glycoforms that are typically found in recombinant biologics produced with Chinese Hamster Ovary (CHO) cells. In addition to providing useful insight into the extent to which on-line glycosylation control is achievable in actual manufacturing processes, the results also have important implications for genetically engineering cell lines design for enhanced glycosylation controllability.

990 ||| 24852214 ||| (8, 0.48),(1, 0.36) ||| 8 ||| 1 ||| Modeling of cell culture damage and recovery leads to increased antibody and biomass productivity in CHO cell cultures. The development of an efficient and productive cell-culture process requires a deep understanding of intracellular mechanisms and extracellular conditions for optimal product synthesis. Mathematical modeling provides an effective strategy to predict, control, and optimize cell performance under a range of culture conditions. In this study, a mathematical model is proposed for the investigation of cell damage of a Chinese hamster ovary cell culture secreting recombinant anti-RhD monoclonal antibody (mAb). Irreversible cell damage was found to be correlated with a reduction in pH. This irreversible damage to cellular function is described mathematically by a Tessier-based model, in which the actively growing fraction of cells is dependent on an intracellular metabolic product acting as a growth inhibitor. To further verify the model, an offline model-based optimization of mAb production in the cell culture was carried out, with the goal of minimizing cell damage and thereby enhancing productivity through intermittent refreshment of the culture medium. An experimental implementation of this model-based strategy resulted in a doubling of the yield as compared to the batch operation and the resulting biomass and productivity profiles agreed with the model predictions.

991 ||| 25203722 ||| (8, 0.47),(3, 0.47) ||| 8 ||| 1 ||| Evaluation of a multimodal resin for selective capture of CHO-derived monoclonal antibodies directly from harvested cell culture fluid. This proof-of-concept study examines the applicability of using multimodal chromatography to selectively capture recombinantly produced monoclonal antibodies (mAb) directly from harvested mammalian cell culture fluid (HCCF) with minimal optimization. Capto MMC is a multimodal resin that contains a ligand with the potential to participate in ionic, hydrophobic, and hydrogen boding interactions with proteins and is coupled to a highly cross-linked agarose bead matrix. Twelve mAb HCCF feedstocks were examined for dynamic binding capacity (DBC) and then two representative feedstocks were selected to develop a systematic approach for elution buffer development. A range of dynamic binding capacities was observed for 10 feedstocks (24-53g/L) and two feedstocks had poor binding properties (<10g/L) despite load conditioning towards a more favorable pH. Analysis of the DBC versus molecular properties showed that the mAb-ligand binding interaction was predominantly charge based. Four separate elution strategies were identified to selectively recover the mAb and could be applied with minimal optimization to other mAb feedstocks. Downstream processing of the Capto MMC pools showed that it is feasible to produce material with comparable purity to a process with affinity capture after two chromatography steps.

992 ||| 23890554 ||| (8, 0.47) ||| 8 ||| 1 ||| Isolation of monoclonal antibody from a Chinese hamster ovary supernatant. II: dynamics of the integrated separation on ion exchange and hydrophobic interaction chromatography media. Dynamics of the purification process of a CHO derived monoclonal antibody by ion exchange chromatography (IEC), hydrophobic interaction chromatography (HIC) and their integration has been investigated. To quantify the adsorption behavior of the target protein (IgG1) and impurities contained in the supernatant, their elution course on IEC and HIC columns has been analyzed versus pH and/or the salt concentration in the mobile phase. A short-cut method has been proposed for mathematical modeling and determining underlying kinetic and thermodynamic parameters. The accuracy of the model predictions has been verified by comparing the simulated and experimental band profiles recorded in both chromatographic processes. After verification, the model was used to optimize operating conditions for the column loading and chromatographic elution in the integrated process IEC/HIC. Two alternative loading techniques based on the upstream and downstream feed dilution were taken into account in the optimization routine. In the first one the feed stream was diluted with the loading buffer prior to the column loading, while in the latter one the feed dilution was realized inside the column using the multiple-injection technique. It was shown that the downstream dilution allowed significant reduction of the contact time between the protein and the loading buffer.

993 ||| 24016587 ||| (8, 0.46),(1, 0.46) ||| 8 ||| 1 ||| Performance monitoring of a mammalian cell based bioprocess using Raman spectroscopy. Being able to predict the final product yield at all stages in long-running, industrial, mammalian cell culture processes is vital for both operational efficiency, process consistency, and the implementation of quality by design (QbD) practices. Here we used Raman spectroscopy to monitor (in terms of glycoprotein yield prediction) a fed-batch fermentation from start to finish. Raman data were collected from 12 different time points in a Chinese hamster ovary (CHO) based manufacturing process and across 37 separate production runs. The samples comprised of clarified bioprocess broths extracted from the CHO cell based process with varying amounts of fresh and spent cell culture media. Competitive adaptive reweighted sampling (CoAdReS) and ant colony optimization (ACO) variable selection methods were used to enhance the predictive ability of the chemometric models by removing unnecessary spectral information. Using CoAdReS accurate prediction models (relative error of predictions between 2.1% and 3.3%) were built for the final glycoprotein yield at every stage of the bioprocess from small scale up to the final 5000 L bioreactor. This result reinforces our previous studies which indicate that media quality is one of the most significant factors determining the efficiency of industrial CHO-cell processes. This Raman based approach could thus be used to manage production in terms of selecting which small scale batches are progressed to large-scale manufacture, thus improving process efficiency significantly.

994 ||| 23737295 ||| (8, 0.45),(6, 0.34) ||| 8 ||| 1 ||| CHO cell line specific prediction and control of recombinant monoclonal antibody N-glycosylation. Here we demonstrate that it is possible to predict and control N-glycan processing of a secreted recombinant monoclonal antibody during manufacturing process development using a combination of statistical modelling and comparative measurement of cell surface glycans using fluorescent lectins. Using design of experiments--response surface modelling (DoE-RSM) methodology to adjust the relative media concentrations of known metabolic effectors of galactosylation (manganese, galactose, and uridine) we have shown that beta1,4-galactosylation of the same recombinant IgG4 monoclonal antibody produced by different CHO cell lines can be precisely controlled in a cell line specific manner. For two cell lines, monoclonal antibody galactosylation could be increased by over 100% compared to control, non-supplemented cultures without a reduction in product titre and with minimal effect on cell growth. Analysis of galactosylation effector interactions by DoE-RSM indicated that Mn2+ alone was necessary but not sufficient to improve galactosylation, and that synergistic combinations of Gal and Urd were necessary to maximize galactosylation, whilst minimizing the deleterious effect of Urd on cell growth. To facilitate rapid cell culture process development we also tested the hypothesis that substrate-level control of cellular galactosylation would similarly affect both cell surface and secreted monoclonal antibody glycans, enabling facile indirect prediction of product glycan processing. To support this hypothesis, comparative quantitation of CHO cell surface beta1,4-galactosylation by flow cytometry using fluorescent derivatives of RCA and ConA lectins revealed that substrate-controlled variation in monoclonal antibody galactosylation and cell surface galactosylation were significantly correlated. Taken together, these data show that precision control of a complex, dynamic cellular process essential for the definition of protein product molecular heterogeneity and bioactivity is possible. Moreover, real-time, or near real-time control can be enabled by facile, rapid measurement of cell surface biomarkers of cellular biosynthetic capability.

995 ||| 12889793 ||| (8, 0.43),(5, 0.38) ||| 8 ||| 1 ||| Estimation of Chinese hamster ovary cell density in packed-bed bioreactor by lactate production rate. A method is described for estimating recombinant Chinese hamster ovary (rCHO) cell density in a packed-bed bioreactor by lactate production rate. The lactate production rate, which depended on both the cell numbers and cell growth rate, was modeled by segregating the cell population into two parts: one growing at a maximum specific growth rate and another non-growing. The individual cell in each part had the same lactate production rate. The established rate equation of lactate production matched the experimental data reasonably well and could be used to estimate the cell growth in the batch culture with microcarriers. Furthermore, in the perfusion culture of rCHO cells in a packed-bed bioreactor, the final cell density, 1.3 x 10(10) cells l(-1), estimated by lactate production rate, was comparable to the direct sample counting of 1.2 x 10(10) cells l(-1), showing that lactate production rate method would be useful in tracing the cell growth in packed-bed bioreactors.

996 ||| 23436789 ||| (8, 0.43),(1, 0.39) ||| 8 ||| 1 ||| Very high density of CHO cells in perfusion by ATF or TFF in WAVE bioreactor . Part I. Effect of the cell density on the process. High cell density perfusion process of antibody producing CHO cells was developed in disposable WAVE Bioreactor using external hollow fiber filter as cell separation device. Both "classical" tangential flow filtration (TFF) and alternating tangential flow system (ATF) equipment were used and compared. Consistency of both TFF- and ATF-based cultures was shown at 20-35 x 10(6) cells/mL density stabilized by cell bleeds. To minimize the nutrients deprivation and by-product accumulation, a perfusion rate correlated to the cell density was applied. The cells were maintained by cell bleeds at density 0.9-1.3 x 10(8) cells/mL in growing state and at high viability for more than 2 weeks. Finally, with the present settings, maximal cell densities of 2.14 x 10(8) cells/mL, achieved for the first time in a wave-induced bioreactor, and 1.32 x 10(8) cells/mL were reached using TFF and ATF systems, respectively. Using TFF, the cell density was limited by the membrane capacity for the encountered high viscosity and by the pCO2 level. Using ATF, the cell density was limited by the vacuum capacity failing to pull the highly viscous fluid. Thus, the TFF system allowed reaching higher cell densities. The TFF inlet pressure was highly correlated to the viscosity leading to the development of a model of this pressure, which is a useful tool for hollow fiber design of TFF and ATF. At very high cell density, the viscosity introduced physical limitations. This led us to recommend cell densities under 1.46 x 10(8) cell/mL based on the analysis of the theoretical distance between the cells for the present cell line.

997 ||| 10553663 ||| (8, 0.42),(3, 0.30) ||| 8 ||| 1 ||| Expanded bed protein A affinity chromatography of a recombinant humanized monoclonal antibody: process development, operation, and comparison with a packed bed method. We show that expanded bed protein A affinity chromatography using Streamline rProtein A media is an efficient method for purifying a recombinant humanized monoclonal antibody from unclarified Chinese hamster ovary cell culture fluid and that it provides purification performance comparable to using a packed bed. We determined that the dynamic capacity of the expanded bed media is related to flow rate (measured in column volumes per hour) by a power function, which allows a high capacity at a low flow rate. At 250 cm h-1 with a 25 cm bed height (10 column volumes h-1), the dynamic capacity is 30 g l-1. The yield and purity (measured by the amount of host cell proteins, DNA, SDS-PAGE, and turbidity) of the antibody purified by expanded bed is comparable to the yield and purity obtained on a standard packed bed method using Prosep A media.

998 ||| 19834967 ||| (8, 0.42),(1, 0.33) ||| 8 ||| 1 ||| Modeling kinetics of a large-scale fed-batch CHO cell culture by Markov chain Monte Carlo method. Markov chain Monte Carlo (MCMC) method was applied to model kinetics of a fed-batch Chinese hamster ovary cell culture process in 5,000-L bioreactors. The kinetic model consists of six differential equations, which describe dynamics of viable cell density and concentrations of glucose, glutamine, ammonia, lactate, and the antibody fusion protein B1 (B1). The kinetic model has 18 parameters, six of which were calculated from the cell culture data, whereas the other 12 were estimated from a training data set that comprised of seven cell culture runs using a MCMC method. The model was confirmed in two validation data sets that represented a perturbation of the cell culture condition. The agreement between the predicted and measured values of both validation data sets may indicate high reliability of the model estimates. The kinetic model uniquely incorporated the ammonia removal and the exponential function of B1 protein concentration. The model indicated that ammonia and lactate play critical roles in cell growth and that low concentrations of glucose (0.17 mM) and glutamine (0.09 mM) in the cell culture medium may help reduce ammonia and lactate production. The model demonstrated that 83% of the glucose consumed was used for cell maintenance during the late phase of the cell cultures, whereas the maintenance coefficient for glutamine was negligible. Finally, the kinetic model suggests that it is critical for B1 production to sustain a high number of viable cells. The MCMC methodology may be a useful tool for modeling kinetics of a fed-batch mammalian cell culture process.

999 ||| 24794799 ||| (8, 0.41),(5, 0.33) ||| 8 ||| 1 ||| Reduced quenching and extraction time for mammalian cells using filtration and syringe extraction. In order to preserve the in vivo metabolite levels of cells, a quenching protocol must be quickly executed to avoid degradation of labile metabolites either chemically or biologically. In the case of mammalian cell cultures cultivated in complex media, a wash step previous to quenching is necessary to avoid contamination of the cell pellet with extracellular metabolites, which could distort the real intracellular concentration of metabolites. This is typically achieved either by one or multiple centrifugation/wash steps which delay the time until quenching (even harsh centrifugation requires several minutes for processing until the cells are quenched) or filtration. In this article, we describe and evaluate a two-step optimized protocol based on fast filtration by use of a vacuum pump for quenching and subsequent extraction of intracellular metabolites from CHO (Chinese hamster ovary) suspension cells, which uses commercially available components. The method allows transfer of washed cells into liquid nitrogen within 10-15s of sampling and recovers the entire extraction solution volume. It also has the advantage to remove residual filter filaments in the final sample, thus preventing damage to separation columns during subsequent MS analysis. Relative to other methods currently used in the literature, the resulting energy charge of intracellular adenosine nucleotides was increased to 0.94 compared to 0.90 with cold PBS quenching or 0.82 with cold methanol/AMBIC quenching.

1000 ||| 10512791 ||| (8, 0.41),(3, 0.36) ||| 8 ||| 1 ||| Performance comparison of protein A affinity-chromatography sorbents for purifying recombinant monoclonal antibodies. We describe the performance characteristics of five Protein A affinity-chromatography sorbents (Sepharose Fast Flow, Poros 50, Poros LP, Prosep and Streamline) for purifying a recombinant humanized monoclonal antibody from clarified Chinese hamster ovary cell culture fluid. We measured the dynamic capacity at varying flow rates, maximum capacity, pressure drop and production rate. For purified antibody, we measured yield and purity (by SDS/PAGE, the amount of DNA, the amount of host-cell proteins and the amount of Protein A). We found that, whereas all sorbents provided significant and essentially equivalent antibody purification, there were differences in capacity and pressure drop, which affected the production rate and had implications for process applications.

1001 ||| 22358551 ||| (8, 0.40),(6, 0.40) ||| 8 ||| 1 ||| Long-term stable production of monocyte-colony inhibition factor (M-CIF) from CHO microcarrier perfusion cultures. Monocyte-colony inhibition factor (M-CIF) was produced in microcarrier perfusion cultures from engineered Chinese hamster ovary (CHO) cells. Three and fifteen liter microcarrier perfusion bioreactors equipped with internal spin filters were operated for over two months. Approximately 60 L and 300 L of culture filtrate were harvested from the 3L and 15L microcarrier perfusion bioreactors respectively. During the perfusion operation, cell density reached 2-6 x 10(6) cells/ml. Importantly, stable expression of M-CIF from the CHO cells under non-selection condition was maintained at a level of 4-10 mg/L. Specific productivity was maintained at 1.8-3.4 mg/billion cells/day. The ability of the recombinant CHO cells to migrate from microcarrier to microcarrier under our proprietary HGS-CHO-3 medium greatly facilitated microcarrier culture scale-up and microcarrier replenishment. Future directions for microcarrier perfusion system scale-up and process development are highlighted.

1002 ||| 25403790 ||| (8, 0.40) ||| 8 ||| 1 ||| High-throughput mAb expression and purification platform based on transient CHO. A high-cell-density transient transfection system was recently developed in our laboratory based on a CHO-GS-KO cell line. This method yields monoclonal antibody titers up to 350 mg/L from a simple 7-day process, in volumes ranging from 2 mL to 2 L. By performing transfections in 24-deep-well plates, a large number of mAbs can be expressed simultaneously. We coupled this new high-throughput transfection process to a semiautomated protein A purification process. Using a Biomek FX(p) liquid handling robot, up to 72 unique mAbs can be simultaneously purified. Our primary goal was to obtain >0.25 mg of purified mAb at a concentration of >0.5 mg/mL, without any concentration or buffer-exchange steps. We optimized both the batch-binding and the batch elution steps. The length of the batch-binding step was important to minimize mAb losses in the flowthrough fraction. The elution step proved to be challenging to simultaneously maximize protein recovery and protein concentration. We designed a variable volume elution strategy based on the average supernatant titer. Finally, we present two case studies. In the first study, we produced 56 affinity maturation mAb variants at an average yield of 0.33 +- 0.05 mg (average concentration of 0.65 +- 0.10 mg/mL). In a second study, we produced 42 unique mAbs, from an early-stage discovery effort, at an average yield of 0.79 +- 0.31 mg (average concentration of 1.59 +- 0.63 mg/mL). The combination of parallel high-yielding transient transfection and semiautomated high-throughput protein A purification represents a valuable mAb drug discovery tool.

1003 ||| 22976819 ||| (8, 0.40) ||| 8 ||| 1 ||| A kinetic-metabolic model based on cell energetic state: study of CHO cell behavior under Na-butyrate stimulation. A kinetic-metabolic model approach describing and simulating Chinese hamster ovary (CHO) cell behavior is presented. The model includes glycolysis, pentose phosphate pathway, TCA cycle, respiratory chain, redox state and energetic metabolism. Growth kinetic is defined as a function of the major precursors for the synthesis of cell building blocks. Michaelis-Menten type kinetic is used for metabolic intermediates as well as for regulatory functions from energy shuttles (ATP/ADP) and cofactors (NAD/H and NADP/H). Model structure and parameters were first calibrated using results from bioreactor cultures of CHO cells expressing recombinant t-PA. It is shown that the model can simulate experimental data for all available experimental data, such as extracellular glucose, glutamine, lactate and ammonium concentration time profiles, as well as cell energetic state. A sensitivity analysis allowed identifying the most sensitive parameters. The model was then shown to be readily adaptable for studying the effect of sodium butyrate on CHO cells metabolism, where it was applied to the cases with sodium butyrate addition either at mid-exponential growth phase (48 h) or at the early plateau phase (74 h). In both cases, a global optimization routine was used for the simultaneous estimation of the most sensitive parameters, while the insensitive parameters were considered as constants. Finally, confidence intervals for the estimated parameters were calculated. Results presented here further substantiate our previous findings that butyrate treatment at mid-exponential phase may cause a shift in cellular metabolism toward a sustained and increased efficiency of glucose utilization channeled through the TCA cycle.

1004 ||| 18636599 ||| (8, 0.39),(3, 0.32) ||| 8 ||| 1 ||| A mathematical model of N-linked glycoform biosynthesis. Metabolic engineering of N-linked oligosaccharide biosynthesis to produce novel glycoforms or glycoform distributions of a recombinant glycoprotein can potentially lead to an improved therapeutic performance of the glycoprotein product. Effective engineering of this pathway to maximize the fractions of beneficial glycoforms within the glycoform population of a target glycoprotein can be aided by a mathematical model of the N-linked glycosylation process. A mathematical model is presented here, whose main function is to calculate the expected qualitative trends in the N-linked oligosaccharide distribution resulting from changes in the levels of one or more enzymes involved in the network of enzyme-catalyzed reactions that accomplish N-linked oligosaccharide biosynthesis. It consists of mass balances for 33 different oligosaccharide species N-linked to a specified protein that is being transported through the different compartments of the Golgi complex. Values of the model parameters describing Chinese hamster ovary (CHO) cells were estimated from literature information. A basal set of kinetic parameters for the enzyme-catalyzed reactions acting on free oligosaccharide substrates was also obtained from the literature. The solution of the system for this basal set of parameters gave a glycoform distribution consisting mainly of complex-galactosylated oligosaccharides distributed in structures with different numbers of antennae in a fashion similar to that observed for various recombinant proteins produced in CHO cells. Other simulations indicate that changes in the oligosaccharide distribution could easily result from alteration in glycoprotein productivity within the range currently attainable in industry. The overexpression of N-acetylglucosaminyltransferase III in CHO cells was simulated under different conditions to test the main function of the model. These simulations allow a comparison of different strategies, such as simultaneous overexpression of several enzymes or spatial relocation of enzymes, when trying to optimize a particular glycoform distribution.

1005 ||| 22857861 ||| (8, 0.38) ||| 8 ||| 1 ||| Fishing human monoclonal antibodies from a CHO cell supernatant with boronic acid magnetic particles. In this work we have evaluated the potential of boronic acid functionalized magnetic particles for the one-step capture of a human monoclonal antibody (mAb) from a Chinese hamster ovary (CHO) cell culture supernatant. For comparison, Protein A coated magnetic particles were also used. The most important factor influencing the overall process yield and product purity in boronic acid particles was found to be the binding pH. Basic pH values promoted higher purities while resulting in decreased yields due to the competing effects of molecules such as glucose and lactate present in the cell culture supernatant. After optimization, the particles were successfully used in a multi-cycle purification process of the mAb from the CHO feedstock. Boronic acid particles were able to achieve an average overall yield of 86% with 88% removal of CHO host cell proteins (HCP) when the binding was performed at pH 7.4, while at pH 8.5 these values were 58% and 97%, respectively. In both cases, genomic DNA removal was in excess of 97%. Comparatively, Protein A particles recorded an average overall yield of 80% and an HCP removal greater than 99%. The adsorption of the mAb to the boronic acid particles was shown to be mediated by strong affinity interactions. Overall, boronic acid based purification processes can offer a cost-effective alternative to Protein A as the direct capturing step from the mammalian cell culture.

1006 ||| 19002842 ||| (8, 0.38) ||| 8 ||| 1 ||| Separation of CHO cells using hydrocyclones. Hydrocyclones are simple and robust separation devices with no moving parts. In the past few years, their use in animal cell separation has been proposed. In this work, the use of different hydrocyclone configurations for Chinese hamster ovary (CHO) cell separation was investigated following an experimental design. It was shown that cell separation efficiencies for cultures of the wild-type CHO.K1 cell line and of a recombinant CHO cell line producing granulocyte-macrophage colony stimulating factor (GM-CSF) were kept above 97%. Low viability losses were observed, as measured by trypan blue exclusion and by determination of intracellular lactate dehydrogenase (LDH) released to the culture medium. Mathematical models were proposed to predict the flow rate, flow ratio and separation efficiency as a function of hydrocyclone geometry and pressure drop. When cells were monitored for any induction of apoptosis upon passage through the hydrocyclones, no increase in apoptotic cell concentration was observed within 48 h of hydrocycloning. Thus, based on the high separation efficiencies, the robustness of the equipment, and the absence of apoptosis induction, hydrocyclones seem to be specially suited for use as cell retention devices in long-term perfusion runs.

1007 ||| 11879713 ||| (8, 0.36),(1, 0.34) ||| 8 ||| 1 ||| Instantaneous evaluation of mammalian cell culture growth rates through analysis of the mitotic index. Since a culture increases in cell number when dividing cells separate into two newborn cells, the fraction of mitotic cells in a growing cell population directly reflects the overall growth behavior of a cell culture. To rapidly assess the effects of growth conditions on the fraction of mitotic cells we have employed an antibody specific for the phosphorylated form of histone H3 for the identification of mitotic cells using flow cytometry. The phosphorylation of histone H3 closely correlates with the chromosomal condensation that accompanies the onset of mitosis, and, therefore, it represents a convenient marker for dividing cells. We have optimized the protocol for the staining of mitotic cells for both Chinese hamster ovary and hybridoma cell cultures. Fluorescence micrographs taken of stained cells show that cells in the various stages of mitosis can be detected based on the morphological characteristics of the chromosomes. The variation in the mitotic cell fraction has been determined throughout the batch growth phases of cultures under different growth conditions. The dynamics of the mitotic index show that balanced growth was never truly reached and that the growth rate is in fact quite variable for these cultures since large variations in the mitotic index are observed. In addition, a large increase in the fraction of mitotic cells just prior to the exponential growth phase for all cultures indicates that they are partially synchronized at the exit from the lag phase. According to a two-staged, age structured population balance model, the mitotic index is directly proportional to the growth rate of a culture. The proportionality constant for this case is shown to be the time required for cells to progress through mitosis. This time is believed to be constant for a particular cell line, as shown by experimental data. Thus, growth rates can be determined solely by measurement of the fraction of cells in mitosis. The mitotic index measurements were then used to calculate the growth in cell number of the cultures, and these simulations accurately reflect observed cell counts. Other simulations also show that changes in cell growth can be predicted before they are reflected in the cell count data. This technique can be used as a sensitive indicator of cell growth and could be useful as a process monitoring technique and for developing better feeding strategies for animal cell cultures.

1008 ||| 18676154 ||| (8, 0.36) ||| 8 ||| 1 ||| Cell survival and chromosomal aberrations in CHO-K1 cells irradiated by carbon ions. Chinese hamster ovary CHO-K1 cells were exposed to high LET (12)C-beam (LET: 830 keV/microm) in the dose range of 0-6 Gy and to (60)Co irradiation and the RBE value was obtained. Effects of (12)C-beam exposure on cell survival and chromosomal aberrations were calculated. The chromosomal aberration data were fitted with linear equation. The distribution of aberration in cells was examined with a standard u-test and used to evaluate the data according to Poisson probabilities. The variance to the mean ratio sigma(2)/Y and the dispersion index (u) were determined. Overdispersion was significant (p<0.05) when the value of u exceeded 1.96.

1009 ||| 24081924 ||| (8, 0.35),(2, 0.30) ||| 8 ||| 1 ||| Model-directed engineering of "difficult-to-express" monoclonal antibody production by Chinese hamster ovary cells. Despite improvements in volumetric titer for monoclonal antibody (MAb) production processes using Chinese hamster ovary (CHO) cells, some "difficult-to-express" (DTE) MAbs inexplicably reach much lower process titers. These DTE MAbs require intensive cell line and process development activity, rendering them more costly or even unsuitable to manufacture. To rapidly and rationally identify an optimal strategy to improve production of DTE MAbs, we have developed an engineering design platform combining high-yielding transient production, empirical modeling of MAb synthesis incorporating an unfolded protein response (UPR) regulatory loop with directed expression and cell engineering approaches. Utilizing a panel of eight IgG1 lambda MAbs varying >4-fold in volumetric titer, we showed that MAb-specific limitations on folding and assembly rate functioned to induce a proportionate UPR in host CHO cells with a corresponding reduction in cell growth rate. Derived from comparative empirical modeling of cellular constraints on the production of each MAb we employed two strategies to increase production of DTE MAbs designed to avoid UPR induction through an improvement in the rate/cellular capacity for MAb folding and assembly reactions. Firstly, we altered the transfected LC:HC gene ratio and secondly, we co-expressed a variety of molecular chaperones, foldases or UPR transactivators (BiP, CypB, PDI, and active forms of ATF6 and XBP1) with recombinant MAbs. DTE MAb production was significantly improved by both strategies, although the mode of action was dependent upon the approach employed. Increased LC:HC ratio or CypB co-expression improved cell growth with no effect on qP. In contrast, BiP, ATF6c and XBP1s co-expression increased qP and reduced cell growth. This study demonstrates that expression-engineering strategies to improve production of DTE proteins in mammalian cells should be product specific, and based on rapid predictive tools to assess the relative impact of different engineering interventions.

1010 ||| 24630065 ||| (8, 0.35) ||| 8 ||| 1 ||| Development of a phenyl membrane chromatography-based process yielding pharmaceutical grade plasmid deoxyribonucleic acid for mammalian cells transfection. Production of plasmid DNA pharmaceuticals requires fast, robust and cost effective methodologies able to deliver high amounts of the target molecule in short periods of time. Membrane adsorbers can be tailored and operated to suit such criteria. This study focuses on the impact of pDNA samples produced by a membrane chromatography-based purification methodology on the transfection efficiency of CHO cells. Chromatographies were performed with 5mL of plasmid-containing clarified bacterial lysate each on a Sartorius Phenyl 3mL spiral cartridge using a bind and elute mode to purify the GFP expressing pVAX1/GFP model plasmid. The developed methodology could deliver up to 285mug pDNA samples per run that were virtually RNA free (over 99% removal) and chromatographic step yields of 85%. The purified samples had a reduced content of OC pDNA (~15% less in average). Additionally, robustness of the process was assessed up to nine chromatographic runs without noticing any relevant loss in chromatographic performance and transfection capabilities. The increase of productivity was also studied by increasing the flow rate 5 fold-a maximum productivity of 100mug pDNA/(hmL-BV) was achieved. The pDNA samples produced led to transfection efficiencies that were comparable among all experiments-72% and within 4% relative standard deviation when samples were produced using a lower throughput. Transfection efficiencies obtained by the membrane process were comparable to a combined HIC/SEC bead-based purification process, with values ranging within 74-113% of the values obtained from the latter.

1011 ||| 17691812 ||| (8, 0.35) ||| 8 ||| 1 ||| Application of a cell-once-through perfusion strategy for production of recombinant antibody from rCHO cells in a Centritech Lab II centrifuge system. Based upon the results of scale-down intermittent perfusion processes, a cell-once-through (COT) perfusion concept was applied to a dual bioreactor system coupled to a Centritech Lab II centrifuge for culture of recombinant Chinese hamster ovary (rCHO) cells for monoclonal antibody production. In this new culture mode, i.e., the COT perfusion process, total spent medium was transferred to the centrifuge and a fixed percentage was removed. Approximately 99% of the viable cells are transferred to another bioreactor filled with fresh medium by single operation of the Centritech Lab II centrifuge system for about 30 min. Accordingly, a significant reduction of the cell-passage frequency to the centrifuge led to minimization of cell damage caused by mechanical shear stress, oxygen limitation, nutrient limitation, and low temperature outside the bioreactor. The effects of culture temperature shift and fortified medium on cell growth and recombinant antibody production in the COT perfusion process were investigated. Although the suppressive effects of low culture temperature on cell growth led to a loss of stability in a long-term COT perfusion culture system, the average antibody concentration at 33 degrees C was 157.8 mg/L, approximately 2.4-fold higher than that at 37 degrees C. By the use of a fortified medium at 37 degrees C, rCHO cells were maintained at high density above 1.2 x 10(7) cells/mL, and antibody was produced continuously in a range of 260-280 mg/L in a stable long-term COT perfusion culture. The proposed new culture mode, the COT perfusion approach, guarantees the recovery of rCHO cells damaged by lowered temperature or high lactate and ammonium concentration. It will be an attractive choice for minimization of cell damage and stable long-term antibody production with high cell density.

1012 ||| 23943087 ||| (8, 0.31),(1, 0.31) ||| 8 ||| 1 ||| Enhancement of monoclonal antibody production in CHO cells by exposure to He-Ne laser radiation. This study tested the effectiveness of laser biostimulation in small-scale cultures in vitro. We investigated the response of recombinant CHO cells, which are used for the production of monoclonal antibody, to low level laser radiation. The cells were irradiated using a 632.8 nm He-Ne laser in a continuous wave mode at different energy doses. We incubated the irradiated cells in small batch cultures and assessed their proliferation and productivity at various time intervals. Compared to untreated cells, the irradiated cells showed a significant increase in antibody production. Moreover, the results showed that laser irradiation did not affect viability and slightly enhanced proliferation rate.

# ----------------------------- TOPIC9 -----------------------------

1013 ||| 9794806 ||| (9, 0.99) ||| 9 ||| 6 ||| Activation of dihydrofolate reductase following thiol modification involves a conformational change at the active site. Compared with the activation of dihydrofolate reductase (DHFR) by protein denaturants and inorganic salts, activation of the enzyme by thiol modification is relatively slow. Thus it is an ideal system for kinetic study of the activation mechanism. We describe here a kinetic study of the activation of DHFRs from chicken liver and Chinese hamster ovary by p-hydroxymercuribenzoate (p-HMB). The conformational changes in the enzyme molecule that result from the modification were monitored by measuring fluorescence enhancement due to the binding of 2-p-toluidinylnaphthalene-6-sulphonate (TNS), and by monitoring changes in the intrinsic fluorescence of the enzyme. Both activation and the conformational change probed by TNS followed pseudo-first-order kinetics, and the rate constants obtained are in good agreement with each other. The change in intrinsic fluorescence is a biphasic process. The rate of the fast phase, which may reflect a change in the microenvironment of Trp-24 at the active site, coincides with the rate of activation and the conformational change probed by TNS. The rate of the slow phase, which reflects a global conformational change, is about one order of magnitude lower than that of activation. The results indicate that the activation of DHFR by p-HMB is due to modification-induced conformational changes at its active site, rather than the modification of the thiol group itself, which is almost complete within the dead-time of the experiment. This study provides kinetic evidence for the proposal that flexibility at the active site is essential for full expression of catalytic activity.

1014 ||| 10764830 ||| (9, 0.99) ||| 9 ||| 6 ||| Remodeling of sugar chain structures of human interferon-gamma. Natural human interferon (IFN)-gamma has mainly biantennary complex-type sugar chains and scarcely has multiantennary structures. We attempted to remodel the sugar chain structures using IFN-gamma as a model glycoprotein. To obtain the branching glycoforms of IFN-gamma, we introduced the genes for GnT-IV (UDP-N-acetylglucosamine:alpha-1,3-D-mannoside beta-1, 4-N-acetylglucosaminyltransferase) and/or GnT-V (UDP-N-acetylglucosamine:alpha-1,6-D-mannoside beta-1, 6-N-acetylglucosaminyltransferase) into Chinese hamster ovary (CHO) cells producing human IFN-gamma. The parental CHO cells produced IFN-gamma with biantennary sugar chains mainly. When the GnT-IV activity was increased, triantennary sugar chains with a branch produced by GnT-IV increased up to 66.9% of the total sugar chains. When the GnT-V activity was increased, triantennary sugar chains with a corresponding branch increased up to 55.7% of the total sugar chains. When the GnT-IV and -V activities were increased at a time, tetraantennary sugar chains increased up to 56.2% of the total sugar chains. The proportion of these multiantennary sugar chains corresponded to the intracellular activities of GnT-IV and -V. What is more, lectin blot and flow cytometric analysis indicated that the multi-branch structure of the sugar chains was increased not only on IFN-gamma, one of the secretory glycoproteins, but also on almost CHO cellular proteins by introducing either or both of the GnT genes. The results suggest that the branching structure of sugar chains of glycoproteins could be controlled by cellular GnT-IV and GnT-V activities. This technology can produce glycoforms out of natural occurrence, which should enlarge the potency of glycoprotein therapeutics.

1015 ||| 10606765 ||| (9, 0.99) ||| 9 ||| 6 ||| The influence of inorganic phosphate on the activity of adenosine kinase. The enzyme adenosine kinase (AK; EC 2.7.1.20) shows a dependence upon inorganic phosphate (Pi) for activity. The degree of dependence varies among enzyme sources and the pH at which the activity is measured. At physiological pH, recombinant AK from Chinese hamster ovary (CHO) cells and AK from beef liver (BL) show higher affinities for the substrate adenosine (Ado), larger maximum velocities and lower sensitivities to substrate inhibition in the presence of Pi. At pH 6.2, both BL and CHO AK exhibit almost complete dependence on the presence of Pi for activity. The data show that both enzymes exhibit increasing relief from substrate inhibition upon increasing Pi and the inhibition of BL AK is almost completely alleviated by the addition of 50 mM Pi. The affinity of CHO AK for Ado increases asymptotically from K(m) 6.4 microM to a limit of 0.7 microM upon the addition of increasing Pi from 1 to 50 mM. The concentration of Ado necessary to invoke substrate inhibition also increases asymptotically from K(i) 32 microM to a limit of 69 microM at saturating concentrations of phosphate. In the presence of increasing amounts of Pi, the maximal velocity of activity increases hyperbolically. The effect that phosphate exerts on AK may be either to protect the enzyme from inactivation at high adenosine and H(+) concentrations or to stabilize substrate binding at the active site.

1016 ||| 8671719 ||| (9, 0.98) ||| 9 ||| 6 ||| Lowering extracellular calcium content protects cells from arsenite-induced killing and micronuclei formation. The present study demonstrated that calcium ions were accumulated in nuclei of Chinese hamster ovary (CHO)-K1 cells after arsenite treatment. This process was enhanced by verapamil (a calcium channel blocker). Verapamil also significantly increased the cytotoxic effects of arsenite. In contrast, ethylene glycol-bis[beta-aminoethylether] N,N, N1,N1,-tetraacetic acid (EGTA, a calcium-specific chelator), or calcium-free conditions significantly reduced the cytotoxicity or arsenite. Similarly, the strategy of lowering extracellular calcium concentration by modifying the medium or lowering intracellular calcium concentration by administration of the intracellular calcium chelator quin 2, protected cells from arsenite-induced micronuclei formation. These data indicate that the disturbances in intracellular calcium homeostasis maybe involved in arsenite-induced cytotoxicity and micronuclei formation.

1017 ||| 10501230 ||| (9, 0.96) ||| 9 ||| 6 ||| Physical, chemical and immunological stability of CHO-derived hepatitis B surface antigen (HBsAg) particles. Recombinant hepatitis B surface antigen (HBsAg) particles derived from Chinese hamster ovary (CHO) cells were stored at various conditions for 12-18 months in their naked form or adsorbed to alum (HBV vaccine). The physical, chemical and immunological parameters during storage at -20 degrees C, 4 degrees C, room temperature and 37 degrees C were investigated. HBsAg particles fully retained the original peptide composition when stored for 6 months, as a dispersion, at -20 degrees C and 4 degrees C; and as lyophilized powder, at all four temperatures. Ten percent sucrose preserved the size, shape and protein content of the naked particles stored at 4 degrees C and -20 degrees C for 18 months as a dispersion or lyophilized. Lyophilization in the presence of glucose, sucrose and trehalose, but not mannitol, further improved the 4 degrees C stability of size, shape and the protein content during 2 years of storage. Stability of the particle's lipid components was inferior to that of the protein components. Dispersions of naked HBsAg and of particles lyophilized in the presence of sucrose and stored at -20 degrees C were the only forms in which the lipid content and composition, including the lipid polyunsaturated acyl chains, were preserved for at least 18 months storage. The level of phospholipid and free cholesterol were most stable in the HBV vaccine which was stored at 4 degrees C; they did not change after 1 year of storage. Preservation of immunogenicity was evaluated according to dose-dependent changes in S-specific antibody titers in sera obtained from immunized BALB/c mice. The ED50 for achieving seroconversion was 0.07 microg/ml/mouse, indicating that the vaccine is very immunogenic. Freezing or freeze-drying of the HBV vaccine results in the total loss of vaccine immunogenicity (in spite of the good chemical stability), while full immunological potency was retained for at least 2.5 years at 4 degrees C. Storing formulated vaccine at 25 and 37 degrees C for 4 and 2 weeks, respectively, did not alter the vaccine potency. This study suggests that the vaccine's physical, chemical and immunological characteristics are sufficiently stable at high temperatures to reduce the need for 'cold chain' transportation.

1018 ||| 10734572 ||| (9, 0.68) ||| 9 ||| 6 ||| Direct coupling of expanded bed adsorption with a downstream purification step. In the course of developing a cost-effective, scaleable process for the purification of a recombinant protein from Chinese hamster ovary (CHO) suspension cell culture, we investigated direct capture of this molecule using expanded bed adsorption (EBA). EBA combines clarification, purification, and concentration of the product into a single step. The unclarified bioreactor material was directly applied to a STREAMLINE 25 column containing an affinity STREAMLINE adsorbent. This work focused on simplifying the EBA operations and minimizing the overall processing time by running the EBA column unidirectionally, eluting in the expanded bed mode, and coupling the EBA column directly with ion exchange or hydrophobic interaction chromatography. Unidirectional EBA was clearly a simpler unit operation and did not require the use of specialized equipment. The increase in the elution pool volume was insignificant, especially when the EBA column was eluted directly onto the downstream column. Scale-down was simple and could be automated. Coupling of unidirectional EBA with a downstream purification step reduced processing time, equipment requirements and cost.

1019 ||| 24033810 ||| (9, 0.66) ||| 9 ||| 6 ||| Evaluation of butyrate-induced production of a mannose-6-phosphorylated therapeutic enzyme using parallel bioreactors. Bioreactor process changes can have a profound effect on the yield and quality of biotechnology products. Mannose-6-phosphate (M6P) glycan content and the enzymatic catalytic kinetic parameters are critical quality attributes (CQAs) of many therapeutic enzymes used to treat lysosomal storage diseases (LSDs). Here, we have evaluated the effect of adding butyrate to bioreactor production cultures of human recombinant beta-glucuronidase produced from CHO-K1 cells, with an emphasis on CQAs. The beta-glucuronidase produced in parallel bioreactors was quantified by capillary electrophoresis, the catalytic kinetic parameters were measured using steady-state analysis, and mannose-6-phosphorylation status was assessed using an M6P-specific single-chain antibody fragment. Using this approach, we found that butyrate treatment increased beta-glucuronidase production up to approximately threefold without significantly affecting the catalytic properties of the enzyme. However, M6P content in beta-glucuronidase was inversely correlated with the increased enzyme production induced by butyrate treatment. This assessment demonstrated that although butyrate dramatically increased beta-glucuronidase production in bioreactors, it adversely impacted the mannose-6-phosphorylation of this LSD therapeutic enzyme. This strategy may have utility in evaluating manufacturing process changes to improve therapeutic enzyme yields and CQAs.

1020 ||| 12928114 ||| (9, 0.52) ||| 9 ||| 6 ||| Monitoring biodistribution of glycoproteins with modified sugar chains. Natural human interferon (hIFN)-gamma has mainly biantennary complex-type sugar chains. Previously, we successfully remodeled its sugar chain structure into: (a) highly branched types; or (b) highly sialylated types, by overexpression of: (a) N-acetylglucosaminyltransferase (GnT)-IV and/or GnT-V; or (b) sialyltransferases, in Chinese hamster ovary (CHO) cells. In addition, we prepared asialo hIFN-gammas by treatment with sialidase in vitro. In the present study, we assessed the bioactivity of remodeled hIFN-gamma in terms of antiviral activity, anticellular activity, and biodistribution. Structural changes to the sugar chains did not have a significant influence on the antiviral and anticellular activities of hIFN-gamma, although the attachment of the sugar chain itself affected both activities. However, the biodistribution differed significantly; the number of exposed galactose residues was the major determinant of the specific distribution to the liver and blood clearance rate of hIFN-gamma. This phenomenon was considered to be mediated by the hepatic asialoglycoprotein receptor (ASGP-R), and we showed a linear, not exponential, enhancement of the distribution to the liver with an increase in the number of exposed galactose residues. We also confirmed this tendency using fibroblast growth factor (FGF). Our observation is not the same as the "glycoside cluster effect." We thus provide important information on the character of modified recombinant glycoproteins.

1021 ||| 11027569 ||| (9, 0.49),(2, 0.34) ||| 9 ||| 6 ||| Correction of glycogen storage disease type II by enzyme replacement with a recombinant human acid maltase produced by over-expression in a CHO-DHFR(neg) cell line. Inherited genetic deficiency of lysosomal acid alpha glucosidase or acid maltase (GAA) results in the autosomal recessive glycogen storage disease type II (GSD II). To investigate whether we could generate a functional recombinant human GAA (rhGAA) for enzyme replacement therapy, we subcloned the cDNAs for human GAA and mouse dihydrofolate reductase (DHFR) into DHFR(neg) Chinese hamster ovary cells and established a stable cotransformant that expressed rhGAA. We cultured the recombinant cells in media with progressively increasing concentrations of methotrexate and found that human GAA enzyme activity increased to over 2,000 IU per gram protein. Importantly, the human GAA enzyme activity correlated to equivalent amounts of human GAA protein by rocketimmunoelectrophoresis. We confirmed that the human GAA enzyme activity corresponded to an amplification in human GAA mRNA by Northern analysis and human GAA cDNA copy number by Southern analysis. Exposing the rhGAA to human GSDII fibroblast cells or patient's lymphocytes or monocytes resulted in uptake of the rhGAA and reversal of the enzymatic defect. Mannose-6-phosphate in the media blocked uptake. GAA -/- mice were treated with the rhGAA at 1 mg/kg, which resulted in heterozygous levels of GAA in tissues, most notably skeletal muscle, heart and diaphragm after two infusions. More importantly, after multiple infusions, hind, and fore-limb muscle weakness was reversed. This rhGAA would be ideal for enzyme replacement therapy in GSD II.

1022 ||| 11237686 ||| (9, 0.41),(3, 0.38) ||| 9 ||| 6 ||| Expression and characterization of human recombinant and alpha-N-acetylglucosaminidase. Mucopolysaccharidosis type IIIB (MPS-IIIB, Sanfilippo type B Syndrome) is a heterosomal, recessive lysosomal storage disorder resulting from a deficiency of [alpha]-N-acetylglucosaminidase (NAGLU). To characterize this enzyme further and evaluate its potential for enzyme replacement studies we expressed the NAGLU-encoding cDNA in Chinese hamster ovary cells (CHO-K1 cells) and purified the recombinant enzyme from the medium of stably transfected cells by a two-step affinity chromatography. Two isoforms of recombinant NAGLU with apparent molecular weights of 89 and 79 kDa were purified and shown to differ in their glycosylation pattern. The catalytic parameters of both forms of the recombinant enzyme were indistinguishable from each other and similar to those of NAGLU purified from various tissues. However, compared to other recombinant lysosomal enzymes expressed from CHO-K1 cells, the mannose-6-phosphate receptor mediated uptake of the secreted form of recombinant NAGLU into cultured skin fibroblasts was considerably reduced. A small amount of phosphorylated NAGLU present in purified enzyme preparations was shown to be endocytosed by MPS-IIIB fibroblasts via the mannose-6-phosphate receptor-mediated pathway and transported to the lysosomes, where they corrected the storage phenotype. Direct metabolic labeling experiments with Na(2) (32)PO(4) confirmed that the specific phosphorylation of recombinant NAGLU secreted from transfected CHO cells is significantly lower when compared with a control lysosomal enzyme. These results suggest that the use of secreted NAGLU in future enzyme and gene replacement therapy protocols will be severely limited due to its small degree of mannose-6-phosphorylation.

1023 ||| 11350179 ||| (9, 0.36),(3, 0.31) ||| 9 ||| 6 ||| Purification and characterization of recombinant human lysosomal alpha-mannosidase. Lysosomal alpha-mannosidase (EC 3.2.1.24) is required in the degradation of the asparagine-linked carbohydrates of glycoproteins. Deficiency of this enzyme leads to the lysosomal storage disorder alpha-mannosidosis. As an initial step toward enzyme replacement therapy for alpha-mannosidosis, the human lysosomal alpha-mannosidase cDNA was cloned into the pcDNA 3.1 vector and expressed in Chinese hamster ovary cells. Dimethyl sulfoxide (DMSO) added to the cell culture media to induce growth arrest led to a 4-fold increase in the enzyme production, with an average yield of 3.2 mg L(-1) day(-1). alpha-Mannosidase was secreted as an active homodimer of a 130-kDa precursor that was proteolyzed into two polypeptides of 55 and 72 kDa during the subsequent purification of the enzyme. N-terminal sequence analysis of the purified enzyme revealed that the proteolysis occurred close to a cleavage site previously identified in the intracellular form of lysosomal alpha-mannosidase. Generation of monoclonal antibodies against the recombinant enzyme made it possible to develop a single-step immunoaffinity purification procedure for alpha-mannosidase. The immunoaffinity-purified enzyme which mainly consisted of the 130-kDa precursor, displayed specific activity and kinetics similar to those of the processed form. Recombinant alpha-mannosidase was taken up by cultured alpha-mannosidosis fibroblasts and was trafficked to the lysosomes via the mannose 6-phosphate pathway where it reduced the amounts of stored mannose-containing oligosaccharides.

1024 ||| 8752306 ||| (9, 0.34) ||| 9 ||| 6 ||| Heat-induced changes in intracellular sodium and membrane potential: lack of a role in cell killing and thermotolerance. Hyperthermia induces transient changes in intracellular free sodium levels and membrane potential. The possible role of these changes in cell killing by hyperthermia and thermotolerance has been evaluated using Chinese hamster ovary IS1 and HeLa cells. Intracellular sodium was measured with Sodium Green and SBFI, while membrane potential was measured with the oxonol dye diBAC4(3). Heating at either 42.0 or 45.0 degrees C caused nearly the same decrease in free [Na+]i from about 20 mM in unheated cells to 5-7 mM in heated cells. However, survival differed by over two orders of magnitude after heating for 30 min at these two temperatures. In addition, blockage of the heat-induced decrease in [Na+]i using ouabain and/or amiloride did not affect the survival curves for heated cells. Hyperthermia also induced a membrane hyperpolarization of 15 mV after 15 min at 42.0 degrees C or 35 mV after 15 min at 45.0 degrees C which could be blocked with ouabain and amiloride. Both the free [Na+]i and membrane potential recovered to near baseline levels within 30-40 min after heating. Induction of thermotolerance using a 45.0 degrees C, 10-min heat treatment also was not affected by ouabain and/or amiloride. Finally, thermotolerant cells experienced the same heat-induced changes in free [Na+]i and membrane potential as non-thermotolerant cells. We conclude that the heat-induced changes in free [Na+]i and membrane potential are not directly related to cell killing by hyperthermia or thermotolerance.

# ----------------------------- TOPIC - NA -----------------------------

1025 ||| 8928771 ||| NA ||| NA ||| NA ||| Glycogen: a carbohydrate source for GLUT-1 glycosylation during glucose deprivation of 3T3-L1 adipocytes. In 3T3-L1 adipocytes, the glycosylation of the GLUT-1 transporter is altered beyond 12 h of glucose deprivation. To determine whether glycogen degradation provides substrate for normal protein glycosylation during this delay, we measured the glycogen content of 3T3-L1 adipocytes. From an initial value of 0.537 +/- 0.097 mumol glucose/10(6) cells, glycogen was depleted in a time-dependent manner in response to glucose deprivation, exhibiting a half-time of 6 h. Surprisingly, fructose did not prevent glycogen depletion. However, in such glycogen-depleted adipocytes, the alteration of GLUT-1 glycosylation in response to glucose deprivation was more rapid than in normal adipocytes. Chinese hamster ovary (CHO) cells, which synthesize abbreviated dolichol-linked oligosaccharides within minutes of glucose deprivation (J. I. Rearick, A. Chapman, and S. Kornfeld. J. Biol. Chem. 256: 6255-6261, 1981), contained only 1% of the level of glycogen found in 3T3-L1 adipocytes. Glycosylation of GLUT-1 was altered in CHO cells within 3 h of glucose deprivation. These data demonstrate that, during glucose stress, glycogen may serve as a buffer for oligosaccharide biosynthesis and provide a potential explanation for varying sensitivities of different cell types to glucose deprivation.

1026 ||| 8866824 ||| NA ||| NA ||| NA ||| Isolation and preliminary characterization of a Chinese hamster ovary cell line with high-degree resistance to hydrogen peroxide. We have isolated and conducted preliminary characterization of a cell line derived from the Chinese hamster ovary cell line AA8, which we have designated AG8 and which is highly resistant to the cytotoxic effects of H2O2 (approximately 17-fold when the H2O2 treatment was at 37 degrees; approximately 11-fold when the H2O2 treatment was at 4 degrees). AG8 cells were moderately (but significantly; P < 0.05) cross-resistant to CdCl2 (approximately 4-fold), NaAsO2 (approximately 2.3-fold), t-butyl hydroperoxide (approximately 2.9-fold), cumene hydroperoxide (approximately 3-fold), menadione (approximately 1.7-fold) and HgCl2 (approximately 1.5-fold), but were not significantly cross-resistant to hyperthermia (43 degrees), 254 nm UV light, 137Cs gamma-rays, and 42-MeV (p-->Be+) fast neutrons. As regards their biochemical status, AG8 and AA8 cells contain similar non-protein sulfhydryl levels per milligram of protein. Catalase activity (assessed by both spectrophotometry and polarography) was significantly higher in AG8 than in AA8 cells irrespective of whether enzyme activity was expressed per 10(6) cells (approximately 3.6-fold increase) or per milligram of protein (approximately 1.6-fold increase). AG8 cells also exhibited significantly greater glutathione reductase activity than wild-type cells when the data were expressed per 10(6) cells (approximately 2.9-fold) or per milligram of protein (approximately 1.3-fold). Glutathione peroxidase activity was immeasurably low in both cell lines. The susceptibility of the two cell lines to H2O2-mediated generation of DNA single-strand breaks (as measured by alkaline elution) indicated a slightly (approximately 1.5-fold) decreased yield in the resistant AG8 cell line. The two cell lines repaired these breaks with similar kinetics. In contrast, no measurable induction of DNA double-strand breaks (as measured by pulsed-field gel electrophoresis) was apparent in either cell line after survival-curve range concentrations of H2O2. On the basis of these data, it appears that the AG8 phenotype involves two previously identified resistance mechanisms, namely an adaptive component that may or may not involve increased antioxidant capacity, and a second component that does involve increased antioxidant (primarily catalase) capacity.

1027 ||| 8834362 ||| NA ||| NA ||| NA ||| Wood-drying condensate from Eastern white pine induced cytotoxicity and genotoxicity in vitro. Eastern white pine is one of the most important commercial species of wood in the Northeast. Condensates extracted from this wood were tested to detect potential cytotoxicity and genotoxicity in Chinese Hamster Ovary (CHO) cells in the absence of S-9 activation. Cytotoxicity was measured by the Trypan blue exclusion assay, mitotic index (MI) and proliferative rate index (PRI). Genotoxicity was measured by the chromosome aberration (CA) assay and sister chromatid exchange (SCE) analysis. Both cytotoxic and genotoxic effects were observed. Laboratory-generated Eastern white pine condensate reduced the viability of CHO cells. The number of viable cells was roughly inversely proportional to dosage over a range of 91 percent to 58 percent survival in treated groups as compared to 2.4 x 10(5) viable cells (100 percent) in the control. The mitotic index (MI) data also showed an inverse correlation with dosage. The highest scorable dose limited by toxicity was determined to be 1 ml of Eastern white pine condensate in a total of 10 ml of medium. Lastly, a dose response curve was observed using the CA assay and also with the SCE analysis. The present findings support results obtained from Ames testing of Eastern white pine condensate and also corroborate results derived from human peripheral-blood lymphocytes.

1028 ||| 8625473 ||| NA ||| NA ||| NA ||| Relationship between the adaptive response to oxidants and stable menadione-resistance in Chinese hamster ovary cell lines. To study the genetic changes that generate resistance to oxidants in mammalian cells, we isolated cell lines that are resistant to the naphthoquinone, menadione, from a Chinese hamster ovary cell line (CHO-K1). Corss-resistance to other oxidants (H2O2 and sodium arsenite) was observed. The IC50 of menadione (measured using a clonogenic assay) was 7.8-fold greater for one menadione-resistant cell line (MRc40) than for CHO-K1. Acquisition of resistance was associated with elevations of 2- and 3.2-fold in the low molecular weight thiols, glutathione and cysteine, respectively. Further, characterization demonstrated significant changes in the expression of enzymes associated with the oxidative stress response and with protection against oxidizing agents. The expressions of glutathione S-transferase pi (GST pi), glutathione peroxidase (GPX) and heme oxygenase mRNAs were all increased. Accompanying these changes the enzyme activity of GST pi, GPX and gamma-glutamyl transpeptidase (gamma-GT) were also elevated. Interestingly, in a revertant cell line heme oxygenase overexpression approached wild-type levels. Intriguingly, similar changes in gene expression seen in the menadione-resistant cells were also observed in wild-type cells following transient oxidative stress, indicating that the observed changes in the resistant line may be due to the immortalization of a normally transient adaptive stress response.

1029 ||| 7656585 ||| NA ||| NA ||| NA ||| Chromosomal localization of the reduced folate transporter gene (SLC19A1) in Chinese hamster ovary cells.

1030 ||| 25545631 ||| NA ||| NA ||| NA ||| How does mild hypothermia affect monoclonal antibody glycosylation? The application of mild hypothermic conditions to cell culture is a routine industrial practice used to improve recombinant protein production. However, a thorough understanding of the regulation of dynamic cellular processes at lower temperatures is necessary to enhance bioprocess design and optimization. In this study, we investigated the impact of mild hypothermia on protein glycosylation. Chinese hamster ovary (CHO) cells expressing a monoclonal antibody (mAb) were cultured at 36.5 C and with a temperature shift to 32 C during late exponential/early stationary phase. Experimental results showed higher cell viability with decreased metabolic rates. The specific antibody productivity increased by 25% at 32 C and was accompanied by a reduction in intracellular nucleotide sugar donor (NSD) concentrations and a decreased proportion of the more processed glycan structures on the mAb constant region. To better understand CHO cell metabolism at 32 C, flux balance analysis (FBA) was carried out and constrained with exometabolite data from stationary phase of cultures with or without a temperature shift. Estimated fluxomes suggested reduced fluxes of carbon species towards nucleotide and NSD synthesis and more energy was used for product formation. Expression of the glycosyltransferases that are responsible for N-linked glycan branching and elongation were significantly lower at 32 C. As a result of mild hypothermia, mAb glycosylation was shown to be affected by both NSD availability and glycosyltransferase expression. The combined experimental/FBA approach generated insight as to how product glycosylation can be impacted by changes in culture temperature. Better feeding strategies can be developed based on the understanding of the metabolic flux distribution.

1031 ||| 24021764 ||| NA ||| NA ||| NA ||| Polyethyleneimine-based transient gene expression processes for suspension-adapted HEK-293E and CHO-DG44 cells. Transient gene expression (TGE) from mammalian cells is an increasingly important tool for the rapid production of recombinant proteins for research applications in biochemistry, structural biology, and biomedicine. Here we review methods for the transfection of human embryo kidney (HEK-293) and Chinese hamster ovary (CHO) cells in suspension culture using the cationic polymer polyethylenimine (PEI) for gene delivery.

1032 ||| 23851271 ||| NA ||| NA ||| NA ||| Inhibition of tumor growth in a mouse xenograft model by the humanized anti-HGF monoclonal antibody YYB-101 produced in a large-scale CHO cell culture. The humanized anti-hepatocyte growth factor (HGF) monoclonal antibody (mAb) YYB-101 is a promising therapeutic candidate for treating various cancers. In this study, we developed a bioprocess for large-scale production of YYB-101 and evaluated its therapeutic potential for tumor treatment using a xenograft mouse model. By screening diverse chemically defined basal media formulations and by assessing the effects of various feed supplements and feeding schedules on cell growth and antibody production, we established an optimal medium and feeding method to produce 757 mg/l of YYB-101 in flask cultures, representing a 7.5-fold increase in titer compared with that obtained under non-optimized conditions. The optimal dissolved oxygen concentration for antibody production was 70% pO2. A pH shift from 7.2 to 7.0, rather than controlled pH of either 7.0 or 7.2, resulted in productivity improvement in 5 L and 200 L bioreactors, yielding 737 and 830 mg/ml of YYB-101, respectively. The YYB-101 mAb highly purified by affinity chromatography using a Protein A column and two-step ion exchange chromatography effectively neutralized HGF in a cell-based assay and showed potent tumor suppression activity in a mouse xenograft model established with human glioblastoma cells.

1033 ||| 23556132 ||| NA ||| NA ||| NA ||| Recombinant human butyrylcholinesterase as a new-age bioscavenger drug: development of the expression system. Butyrylcholinesterase (BChE) is a serine hydrolase (EC 3.1.1.8) which can be found in most animal tissues. This enzyme has a broad spectrum of efficacy against organophosphorus compounds, which makes it a prime candidate for the role of stoichiometric bioscavenger. Development of a new-age DNA-encoded bioscavenger is a vival task. Several transgenic expression systems of human BChE were developed over the past 20 years; however, none of them has been shown to make economic sense or has been approved for administration to humans. In this study, a CHO-based expression system was redesigned, resulting in a significant increase in the production level of functional recombinant human butyrylcholinesterase as compared to the hitherto existing systems. The recombinant enzyme was characterized with Elman and ELISA methods.

1034 ||| 23412111 ||| NA ||| NA ||| NA ||| A biopharmaceutical plant on a chip: continuous micro-devices for the production of monoclonal antibodies. We report a proof-of-principle for the use of micro-devices as continuous bioreactors for the production of monoclonal antibodies. We culture CHO cells on the surface of PMMA "zigzag" channels textured with semi-spherical cavities coated with fibronectin, observing steady-state productivities 100 times higher than those observed in full scale systems.

1035 ||| 22989990 ||| NA ||| NA ||| NA ||| CHO-gmt5, a novel CHO glycosylation mutant for producing afucosylated and asialylated recombinant antibodies. Engineered zinc-finger nucleases (ZFNs) are powerful tools for creating double-stranded-breaks (DSBs) in genomic DNA in a site-specific manner. These DSBs generated by ZFNs can be repaired by homology-directed repair or nonhomologous end joining, in which the latter can be exploited to generate insertion or deletion mutants. Based on published literature, we designed a pair of zinc-finger nucleases and inactivated the GDP-fucose transporter gene (Slc35c1) in a previously reported CHO mutant that has a dysfunctional CMP-sialic acid transporter gene (Slc35a1). The resulting mutant cell line, CHO-gmt5, lacks functional GDP-fucose transporter and CMP-sialic acid transporter. As a result, these cells can only produce asialylated and afucosylated glycoproteins. It is now widely recognized that removal of the core fucose from the N-glycans attached to Asn(297) of human IgG1 significantly enhances its binding to its receptor, FcgammaRIIIa, and thereby dramatically improves antibody-dependent cellular cytotoxicity (ADCC). Recent reports showed that removal of sialic acid from IgG1 also enhances ADCC. Therefore, CHO-gmt5 may represent a more advantageous cell line for the production of recombinant antibodies with enhanced ADCC. These cells show comparable growth rate to wild type CHO-K1 cells and uncompromised transfection efficiency, which make them desirable for use as a production line.

1036 ||| 22695857 ||| NA ||| NA ||| NA ||| Advantages of AlaGln as an additive to cell culture medium: use with anti-CD20 chimeric antibody-producing POTELLIGENT CHO cell lines. L-alanyl-L-glutamine (AlaGln) is dipeptide that has better solubility and stability than Glutamine (Gln). In this study, we evaluated the utility of this dipeptide during culture of POTELLIGENT Chinese hamster ovary (CHO) cells expressing anti-CD20 chimeric antibody. Although AlaGln in the culture medium lowered the specific growth rate, the MAb titer was maximized when Gln was completely replaced by AlaGln in both the basal and feed media. Moreover, AlaGln augmented production of antibody not only at flask scale but also at spinner scale, although the extent of this effect was dependent on the cell clone. To explore the mechanism responsible for the effect of AlaGln on cell growth, we measured apoptosis in the early phase of cell culture on days 8, 9, and 10. The apoptotic ratio was reduced in medium containing AlaGln. Ammonia was generated in medium containing Gln when it was maintained at 37 C, which impeded the growth and productivity of the cells. In contrast, AlaGln produced less ammonia under these conditions, which may have been one of the properties associated with its beneficial effects. We conclude that certain dipeptides can serve as superior alternative sources of amino acids in cell culture and antibody production.

1037 ||| 22473810 ||| NA ||| NA ||| NA ||| Probing of C-terminal lysine variation in a recombinant monoclonal antibody production using Chinese hamster ovary cells with chemically defined media. C-terminal lysine (C-K) variants are commonly observed in therapeutic monoclonal antibodies and recombinant proteins. Heterogeneity of C-K residues is believed to result from varying degree of proteolysis by endogenous carboxypeptidase(s) during cell culture production. The achievement of batch-to-batch culture performance and product quality reproducibility is a key cell culture development criterion. Understanding the operational parameters affecting C-K levels provides valuable insight into the cell culture process. A CHO cell line X expressing a recombinant antibody was selected as the model cell line due to the exhibited sensitivity of its C-K level to the process conditions. A weak cation exchange chromatography (WCX) method with or without carboxypeptidase B (CpB) treatment was developed to monitor the C-K level for in-process samples. The effects of operating conditions (i.e., temperature and culture duration) and media trace elements (copper and zinc) on C-K variants were studied. The dominant effect on C-K level was identified as the trace elements concentration. Specifically, increased C-K levels were observed with increase of copper concentration and decrease of zinc concentration in chemically defined medium. Further, a hypothesis for C-K processing with intracellular and extracellular carboxypeptidase activity was proposed, based on preliminary intracellular carboxypeptidase Western blot results and the extracellular HCCF holding study.

1038 ||| 22138638 ||| NA ||| NA ||| NA ||| Thioredoxin 1 is responsible for antibody disulfide reduction in CHO cell culture. During large-scale manufacturing of an IgG1 monoclonal antibody in Chinese hamster ovary (CHO) cells, reduction of the antibody's disulfide bonds was observed. We present evidence that mammalian thioredoxin 1 (TXN1) is the terminal enzyme responsible for this reduction event. We demonstrate a marked prevention of IgG1 disulfide bond reduction in a cell-density dependent manner by knocking down expression of TXN1 via lentivirus transduction of short hairpin RNA.

1039 ||| 22125188 ||| NA ||| NA ||| NA ||| Inhibition of glutamine-dependent autophagy increases t-PA production in CHO cell fed-batch processes. Understanding the cellular responses caused by metabolic stress is crucial for the design of robust fed-batch bioprocesses that maximize the expression of recombinant proteins. Chinese hamster ovary cells were investigated in chemically defined, serum-free cultures yielding 10(7) cells/mL and up to 500 mg/L recombinant tissue-plasminogen activator (t-PA). Upon glutamine depletion increased autophagosome formation and autophagic flux were observed, along with decreased proliferation and high viability. Higher lysosomal levels correlated with decreased productivity. Chemical inhibition of autophagy with 3-methyl adenine (3-MA) increased the t-PA yield by 2.8-fold. Autophagy-related MAP1LC3 and LAMP2 mRNA levels increased continuously in all cultures. Analysis of protein quality revealed that 3-MA treatment did not alter glycan antennarity while increasing fucosylation, galactosylation, and sialylation. Taken together, these findings indicate that inhibition of autophagy can considerably increase the yield of biotechnology fed-batch processes, without compromising the glycosylation capacity of cells. Monitoring or genetic engineering of autophagy provides novel avenues to improve the performance of cell culture-based recombinant protein production.

1040 ||| 21822681 ||| NA ||| NA ||| NA ||| Effects of clonal variation on growth, metabolism, and productivity in response to trophic factor stimulation: a study of Chinese hamster ovary cells producing a recombinant monoclonal antibody. The growth, metabolism, and productivity of five Chinese hamster ovary (CHO) clones were explored in response to stimulation with insulin (5 mg/L) and LONG( )R(3)IGF-I (20 mug/L or 100 mug/L). All five clones were derived from the same parental CHO cell line (DG44) and produced the same recombinant monoclonal antibody, with varying specific productivities. There was no uniform response among the clones to stimulation with the different trophic factors. One of the high productivity clones (clone D) exhibited significantly better growth in response to LONG( )R(3)IGF-I; whereas the other clones showed equivalent or slightly better growth in the presence of insulin. Three out of the five clones had higher specific productivities in the presence of insulin (although not statistically significant); one was invariant, and the final clone exhibited slightly higher specific productivity in the presence of LONG( )R(3)IGF-I. Total product titers exhibited moderate variation between culture conditions, again with neither trophic factor being clearly superior. Overall product titers were affected by variations in both integrated viable cell density and specific productivity. Nutrient uptake and metabolite generation patterns varied strongly between clones and much less with culture conditions. These results point to the need for careful clonal analysis when selecting clones, particularly for platform processes where media and culture conditions are predetermined.

1041 ||| 20661907 ||| NA ||| NA ||| NA ||| Overexpression of taurine transporter in Chinese hamster ovary cells can enhance cell viability and product yield, while promoting glutamine consumption. Transporters mediate the uptake of nutrients such as amino acids and the excretion of metabolites. The fact that transporters play crucial roles in regulating cell metabolism suggests that they might be useful targets for cell engineering to enhance the yield and/or quality of monoclonal antibody (MAb) produced by CHO cells. The taurine transporter (TAUT) is stably expressed in CHO-DXB11 cells and is upregulated late in the culture period. We found that forcing the overexpression of TAUT delayed apoptotic cell death, extending the culture period. Thus, under fed-batch small-culture conditions, CHO cells that expressed pHyg-TAUT plasmid (TAUT/CHO cells), but not those that contained the null plasmid pHyg (HYG/CHO cells), produced more MAb (P < 0.01) and less lactate (P < 0.05). In a 1-L bioreactor, a representative high-yield TAUT/CHO cell line (T10) showed >80% viability for more than 1 month and a 47% increase in medium MAb concentration. In T10 cells, the upregulation of TNF-alpha mRNA (an apoptosis marker) and the accumulation of ammonia late in the culture period were suppressed. Moreover, if an excess of taurine was added, T10 cells efficiently consumed glutamine but not other amino acids, so T10 cells may have gained a glutamine transporter-like function. Because a considerable amount of metabolic energy is derived from glutamine, this active glutamine consumption in T10 cells might be a reason for the improved cell viability and MAb concentration. These results demonstrate that forcing the overexpression of TAUT in CHO cells can enhance cell culture performance and increase MAb titer.

1042 ||| 20159583 ||| NA ||| NA ||| NA ||| Fetal calf serum-free suspension culture of Chinese hamster ovary cells employing fish serum. The effects of heat treatment and concentration of fish serum (FS) on cell growth in a suspension culture of recombinant Chinese hamster ovary (CHO) 1-15(500) (ATCC CRL-9606) cells were investigated. An increase in FS concentration from 1% to 4% markedly increased cell density. On the other hand, heat treatment of FS showed nearly no effect on cell density.

1043 ||| 19770009 ||| NA ||| NA ||| NA ||| Transcriptome and proteome analysis of Chinese hamster ovary cells under low temperature and butyrate treatment. Recombinant Chinese hamster ovary (CHO) cells selected for high productivity are capable of secreting immunoglobulin G (IgG) molecules at a level that rivals plasma cells in vivo. Following butyrate treatment at 33 degrees C, further increases in productivity are observed. To better understand the mechanisms by which this increased productivity is incurred, the transcriptional response of an antibody-producing cell line undergoing these treatments was investigated using oligo-DNA microarrays. Using distance calculations, more than 900 genes were identified as kinetically differentially expressed between the butyrate-treated 33 degrees C culture and the untreated culture. Furthermore, transcript levels of the heavy and light chain IgG genes increased following treatment. Using stable isotope labeling (SILAC), the secretion rate of IgG was investigated by tracking the decay of the isotope label upon switching to unlabeled medium. Both treated and untreated cultures exhibited very similar IgG secretion kinetics. In contrast, the intracellular IgG content was found to be elevated following treatment. This result suggests that increased productivity under treatment is attributable to elevated cellular secretory capacity, rather than shorter holding times in the secretory pathway. This hypothesis is further supported by the results of gene set enrichment analysis (GSEA), which revealed that elements of the secretory pathway, including Golgi apparatus, cytoskeleton protein binding and small GTPase-mediated signal transduction are enriched and thus may play a role in the increased recombinant protein production observed under butyrate treatment at 33 degrees C.

1044 ||| 19623580 ||| NA ||| NA ||| NA ||| CHO cells adhering to nitrogen-rich plasma-polymerised ethylene exhibit high production of a specific recombinant protein. In many industrial applications, inadequate cell attachment can be a limitation, especially when serum-free media are used. Nitrogen-rich plasma-polymerised ethylene (PPE:N) exhibits high concentrations of polar groups that can help to promote the attachment of weakly adherent cell types. Tissue plasminogen activator-producing Chinese hamster ovary (CHO) cells, adapted to suspension, were grown in the presence PPE:N flakes and were found to adhere to them. The growth rate was reduced, but cell viability was enhanced and their metabolism was more efficient, with generally higher recombinant protein productivity. Finally, cell adhesion on PPE:N surfaces was found to be independent of integrins, and was probably mediated by certain non-specific interactions with the PPE:N surface.

1045 ||| 18215639 ||| NA ||| NA ||| NA ||| Effect of static pressure on intracellular pH of adhesive Chinese hamster ovary cells. The effect of static pressure on the intracellular pH of the Chinese hamster ovary (CHO) cell line DR1000L4N was investigated. In cultivation of CHO cells at 0.9 MPa, two distinct populations were observed in the histogram of a flow cytometer, while single population was observed in cultivation at 0.1 MPa. The intracellular pH of the major population at 0.9 MPa was markedly lower than that of the single population at 0.1 MPa.

1046 ||| 15801797 ||| NA ||| NA ||| NA ||| Effect of copper sulfate on performance of a serum-free CHO cell culture process and the level of free thiol in the recombinant antibody expressed. A recombinant Chinese hamster ovary (CHO) cell line was used to express a humanized antibody. Product quality analysis of this humanized antibody showed the presence of free thiol, due to unpaired cysteine residues in the Fab region. Decreased potency of this thiol Fab made it critical to minimize the levels of free thiol. In an effort to do this, we evaluated the effect of copper sulfate addition to the cell culture production medium. As a component of the production medium, copper sulfate can act as an oxidizing agent, thereby facilitating disulfide bond formation. Four concentrations of copper sulfate were added at the beginning of 2-L benchtop production cultures of the recombinant CHO cell line: 0, 5, 50, and 100 microM. We found that these copper sulfate additions had no effect on cell growth or antibody production. However, a slight dose-dependent depression in culture viability was observed. Analysis of the purified antibody showed that either the 50 or 100 microM copper sulfate additions reduced the level of free thiol by more than 10-fold.

1047 ||| 11415435 ||| NA ||| NA ||| NA ||| Production and characterization of recombinant human CLN2 protein for enzyme-replacement therapy in late infantile neuronal ceroid lipofuscinosis. Late infantile neuronal ceroid lipofuscinosis (LINCL) is a fatal recessive childhood disease caused by mutations in the CLN2 gene, which encodes the lysosomal enzyme tripeptidyl peptidase I. As a step towards understanding the protein and developing therapeutics for the disease, we have produced and characterized recombinant human CLN2 (ceroid lipofuscinosis, neuronal 2) protein from Chinese-hamster ovary cells engineered to secrete high levels of the enzyme. The protein was secreted as an inactive soluble proenzyme of approximately 65 kDa that appears as a monomer by gel filtration. Upon acidification, the protein is processed to mature form and acquires activity. The enzyme is efficiently delivered to the lysosomes of LINCL fibroblasts by mannose 6-phosphate-receptor-mediated endocytosis (EC(50) approximately 2 nM), where it remains active for long periods of time (t(1/2) approximately 12 days). In addition, the enzyme is taken up by rat cerebellar granule neurons by mannose 6-phosphate-dependent and -independent mechanisms. Treatment of LINCL fibroblasts with recombinant CLN2 protein restores normal enzyme activity and ameliorates accumulation of the major storage protein, mitochondrial ATP synthase subunit c.

1048 ||| 10745191 ||| NA ||| NA ||| NA ||| Engineering of coordinated up- and down-regulation of two glycosyltransferases of the O-glycosylation pathway in Chinese hamster ovary (CHO) cells. Production of O-linked oligosaccharides that interact with selectins to mediate cell-cell adhesion occurs in one segment of a branched glycan biosynthesis network. Prior efforts to direct the branched pathway towards selectin-binding oligosaccharides by amplifying enzymes in this branch of the network have had limited success, suggesting that metabolic engineering to simultaneously inhibit the competing pathway may also be required. We report here the partial cloning of the CMP-sialic acid:Galbeta1,3GalNAcalpha2, 3-sialyltransferase (ST3Gal I) gene from Chinese hamster ovary (CHO) cells and the simultaneous inhibition of expression of CHO cell ST3Gal I gene and overexpression of the human UDP-GlcNAc:Galbeta1, 3GalNAc-R beta1,6-N-acetylglucosaminyltransferase (C2GnT) gene. A tetracycline-regulated system adjoined to tricistronic expression technology allowed "one-step" transient manipulation of multiple enzyme activities in the O-glycosylation pathway of a previously established CHO cell line already engineered to express alpha1, 3-fucosyltransferase VI (alpha1,3-Fuc-TVI). Tetracycline-regulated co-expression of a ST3Gal I fragment, cloned in the antisense orientation, and of C2GnT cDNA resulted in inhibition of the ST3Gal I enzymatic activity and increase in C2GnT activity which varied depending on the extent of tetracycline reduction in the cell culture medium. This simultaneous regulated inhibition and activation of the two key enzyme activities in the O-glycosylation pathway of mammalian cells is an important addition to the metabolic engineering field.

1049 ||| 10099358 ||| NA ||| NA ||| NA ||| Antisense strategies for glycosylation engineering of Chinese hamster ovary (CHO) cells. Novel glycoproteins, inaccessible by other techniques, can be obtained by metabolic engineering of the oligosaccharide biosynthesis pathway. Furthermore, alteration of cell-surface oligosaccharides can change the properties of receptors involved in cell-cell adhesion. Sialyl Lewis X (sLex) is a cell-surface oligosaccharide determinant which is specifically expressed on granulocytes and monocytes and which interacts with selectins to influence leukocyte trafficking, thrombosis, inflammation, and cancer. Antisense technology targeting fucosyltransferase VI (Fuc-TVI), an enzyme necessary for the synthesis of the sLex in engineered Chinese hamster ovary (CHO) cells, has reduced Fuc-TVI activity, sLex synthesis, and adhesion to endothelial cells. Antisense methodology to reduce targeted activity in oligosaccharide biosynthesis or other pathways is an important addition to CHO cell metabolic engineering capabilities.
